# Supplementary material for: Tuning the reactivity of alkoxyl radicals from 1,5-hydrogen atom transfer to 1,2-silyl transfer
Source: Nat Commun. 2021 Apr 9;12:2131. doi: 10.1038/s41467-021-22382-y (PMC8035221; doi:10.1038/s41467-021-22382-y)
Supplement: Supplementary file 1 — Supplementary Information [file 41467_2021_22382_MOESM1_ESM.pdf]

SUPPLEMENTARY INFORMATION

Tuning the Reactivity of Alkoxyl Radicals from 1,5-Hydrogen Atom Transfer to 1,2-Silyl Transfer

Yang *et al*

1. General Information

Chromatography: Hai Lang Silica Flash P60 size 40–63 µm (200–300 mesh), TLC: HaiLang silica gel 60 (0.25mm). Visualization of the chromatogram was performed by UV, phosphomolybdic acid. ^{1}H, ^{13}C, ^{19}F were recorded on Bruker 600 and JNM–ECZ 400 using $CDCl_3$ as solvent. Chemical shift values are reported in ppm with the solvent resonance as the internal standard ($CDCl_3$: δ 7.26 for ^{1}H, δ 77.16 for ^{13}C). Data are reported as follows: chemical shifts, multiplicity (s = singlet, bs = broad singlet, d = doublet, dd = doublet of doublets, t = triplet, td = triplet of doublets, m = multiplet), coupling constants (Hz), and integration. Infrared spectra were recorded on an Agilent Technologies Cary 630 FTIR and wavelengths are reported in cm^{-1}. Melting point was measured by INESA SGW X–4. Mass spectra were recorded on Bruker UltiMate3000 & Compact. All reagents were purchased from commercial suppliers (TCI, Aldrich, Alfa, Adamas, energy) and used without further purification and solvents were dried and degassed according to standard procedure.

2. Synthesis of α-alkoxylimino alcohols via radical 1,2-silyl transfer

N-(Benzyloxy)-2-hydroxyoctanimidoyl cyanide(3a)

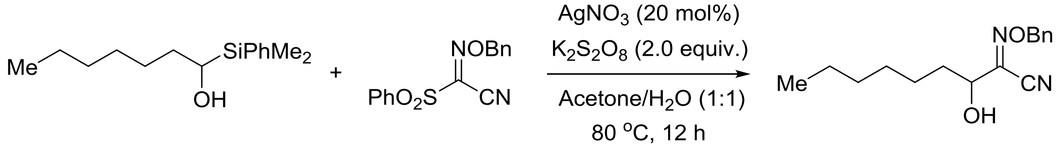

1a **2** **3a**

In an Ar protected glove box, $AgNO_3$ (6.8 mg, 0.04mmol, 20 mol%), **2** (90.0 mg, 0.30mmol, 1.5 equiv.) and **1a** (50.0 mg, 0.20 mmol) were added into a reaction tube. After that, the tube was taken out of the box, acetone/H_2O (0.75 mL / 0.75mL) and $K_2S_2O_8$ (108.0 mg, 0.40mmol, 2.0 equiv.) were added under N_2. The tube was then sealed, and the resulting mixture was kept stirring at 80 °C in heating block for 12 h. The reaction mixture was quenched with water (5 mL), extracted with ethyl acetate (3×10 mL) and organic phase was combined and washed with brine, dried over anhydrous Na_2SO_4, concentrated under reduced pressure. The crude product was purified with column chromatography on silica gel (200–300 mesh) with PE/EA (8/1, v/v) as eluent to afford 37.7 mg of the title compound as a faint yellow oil (69% yield).

R_f = 0.70 (PE/EA = 4/1, v/v). NMR Spectroscopy: ^{1}H NMR (400 MHz, $CDCl_3$, 25 °C) δ 7.41–7.31 (m, 5H), 5.26 (s, 2H), 4.38 (td, J = 6.8, 4.4 Hz, 2H), 2.51 (d, J = 4.9 Hz, 1H), 1.79–1.70 (m, 2H), 1.32–1.26 (m, 8H), 0.89 (t, J = 6.8 Hz, 3H); ^{13}C NMR (101 MHz, $CDCl_3$, 25 °C) δ 135.8, 135.6, 128.6, 128.6, 128.5, 109.0, 78.3, 70.6, 34.5, 31.7, 28.9, 24.8, 22.6, 14.1. IR (ATR): 3392, 2922, 2855, 1707, 1554, 1364, 1297, 1017, 746, 701 cm^{-1}. HRMS (ESI, m/z): calcd for $C_{16}H_{23}N_2O_2^+$ $(M+H)^+$: 275.1759; Found: 275.1754.

N-(Benzyloxy)-2-hydroxyundecanimidoyl cyanide(3b)

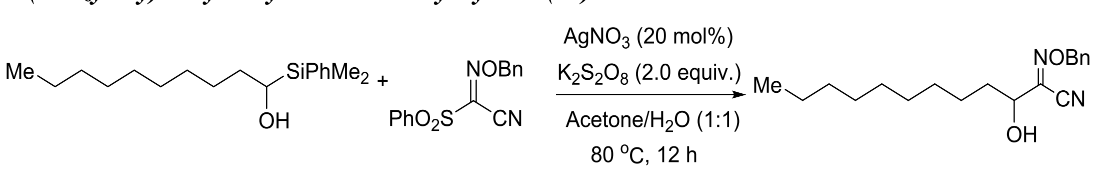

1b **2** **3b**

In an Ar protected glove box, $AgNO_3$ (6.8 mg, 0.04 mmol, 20 mol%), **2** (90.0 mg, 0.30 mmol, 1.5 equiv.) and **1b** (58.4 mg, 0.20 mmol) were added into a reaction tube. After that, the tube was taken out of the box, acetone/H_2O (0.75 mL / 0.75 mL) and $K_2S_2O_8$ (108.0 mg, 0.40 mmol, 2.0 equiv.) were added under N_2. The tube was then sealed, and the resulting mixture was kept stirring at 80 °C in heating block for 12 h. The reaction mixture was quenched with water (5 mL), extracted with ethyl acetate (3×10 mL) and organic phase was combined and washed with brine, dried over anhydrous Na_2SO_4, concentrated under reduced pressure. The crude product was purified with column chromatography on silica gel (200–300 mesh) with PE/EA (10/1, v/v) as eluent to afford 38.0 mg of the title compound as a faint yellow oil (60% yield).

R_f = 0.47 (PE/EA = 4/1, v/v). NMR Spectroscopy: ^{1}H NMR (400 MHz, $CDCl_3$, 25 °C) δ 7.53–7.28 (m, 5 H), 5.27 (s, 2H), 4.40 (t, J = 6.8 Hz, 1H), 2.19 (s, 1H), 1.87–1.61 (m, 2H), 1.48–1.14 (m, 14 H), 0.89 (t, J = 6.9 Hz, 3H); ^{13}C NMR (101 MHz, $CDCl_3$, 25 °C) δ 135.8, 135.6, 128.7, 128.7, 128.6, 109.0, 78.4, 70.8, 34.6, 32.0, 29.6, 29.54, 29.4, 29.3, 24.9, 22.8, 14.3. IR (ATR): 3444, 2926, 2855, 1707, 1453, 1364, 1211, 1006, 734, 697 cm^{-1}. HRMS (ESI, m/z): calcd for $C_{19}H_{28}N_2O_2K^+$ $(M+K)^+$: 355.1788; Found: 355.1782.

N-(Benzyloxy)-2-hydroxyheptanimidoyl cyanide (3c)

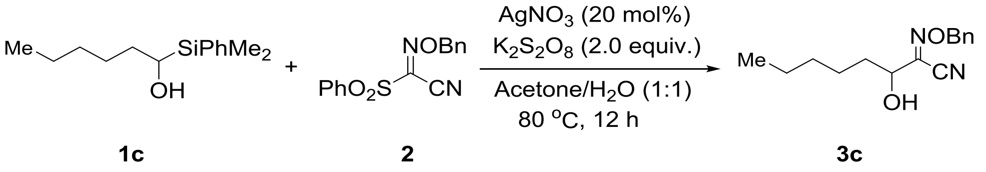

In an Ar protected glove box, $AgNO_3$ (6.8 mg, 0.04 mmol, 20 mol%), **2** (90.0 mg, 0.30 mmol, 1.5 equiv.) and **1c** (47.2 mg, 0.20 mmol) were added into a reaction tube. After that, the tube was taken out of the box, acetone/H_2O (0.75 mL / 0.75 mL) and $K_2S_2O_8$ (108.0 mg, 0.40 mmol, 2.0 equiv.) were added under N_2. The tube was then sealed, and the resulting mixture was kept stirring at 80 °C in heating block for 12 h. The reaction mixture was quenched with water (5 mL), extracted with ethyl acetate (3×10 mL) and organic phase was combined and washed with brine, dried over anhydrous Na_2SO_4, concentrated under reduced pressure. The crude product was purified with column chromatography on silica gel (200–300 mesh) with PE/EA (10/1, v/v) as eluent to afford 32.8 mg of the title compound as a faint yellow oil (63% yield).

R_f = 0.46 (PE/EA = 4/1, v/v). NMR Spectroscopy: ^{1}H NMR (400 MHz, $CDCl_3$, 25 °C) δ 7.39–7.32 (m, 5H), 5.27 (s, 2H), 4.40 (dd, J = 12.1, 6.8 Hz, 1H), 2.27 (d, J = 5.3 Hz, 1H), 1.75 (dd, J = 13.8, 6.8 Hz, 2H), 1.40–1.17 (m, 6 H), 0.95–0.84 (m, 3 H); ^{13}C NMR (101 MHz, $CDCl_3$, 25 °C) δ 135.9, 135.7, 128.7, 128.7, 128.6, 109, 78.4, 70.8, 34.6, 31.4, 24.6, 22.6, 14.1. IR (ATR): 3452, 2930, 2859, 1700, 1454, 1361, 1211, 1010, 738, 697 cm^{-1}. HRMS (ESI, m/z): calcd for $C_{15}H_{20}N_2O_2Na^+$ $(M+Na)^+$: 283.1417; Found: 283.1417.

N-(Benzyloxy)-2-hydroxytridecanimidoyl cyanide (3d)

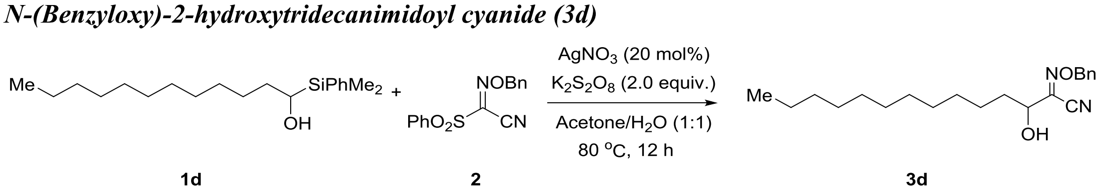

In an Ar protected glove box, $AgNO_3$ (3.4mg, 0.02 mmol, 20 mol%), **2** (45.0 mg, 0.15 mmol, 1.5 equiv.)

and **1d** (31.0 mg, 0.10 mmol) were added into a reaction tube. After that, the tube was taken out of the box, acetone/H_2O (0.375 mL / 0.375 mL) and $K_2S_2O_8$ (54.0 mg, 0.20 mmol, 2.0 equiv.) were added under N_2. The tube was then sealed, and the resulting mixture was kept stirring at 80 °C in heating block for 12 h. The reaction mixture was quenched with water (5 mL), extracted with ethyl acetate (3×10 mL) and organic phase was combined and washed with brine, dried over anhydrous Na_2SO_4, concentrated under reduced pressure. The crude product was purified with column chromatography on silica gel (200–300 mesh) with PE/EA (10/1, v/v) as eluent to afford 19.6 mg of the title compound as a faint yellow oil (57% yield).

R_f = 0.5 (PE/EA = 4/1, v/v). NMR Spectroscopy: ^{1}H NMR (400 MHz, $CDCl_3$, 25 °C) δ 7.41–7.32 (m, 5H), 5.27 (s, 2H), 4.39 (dd, J = 10.5, 6.6 Hz, 1H), 2.23 (d, J = 4.6 Hz, 1H), 1.75 (q, J = 6.9 Hz, 2H), 1.43–1.17 (m, 18H), 0.89 (t, J = 6.8 Hz, 3H); ^{13}C NMR (101 MHz, $CDCl_3$, 25 °C) δ 135.9, 135.6, 128.7, 128.7, 128.6, 109.0, 78.4, 70.8, 34.6, 32.1, 29.7, 29.6, 29.5, 29.5, 29.3, 24.9, 22.8, 14.3. IR (ATR): 3407, 2922, 2855, 1707, 1454, 1364, 1211, 1006, 738, 697 cm^{-1}. HRMS (ESI, m/z): calcd for $C_{21}H_{32}N_2O_2Na^+$ $(M+Na)^+$: 367.2349; Found: 367.2356.

6-Azido-N-(benzyloxy)-2-hydroxyhexanimidoyl cyanide (3e)

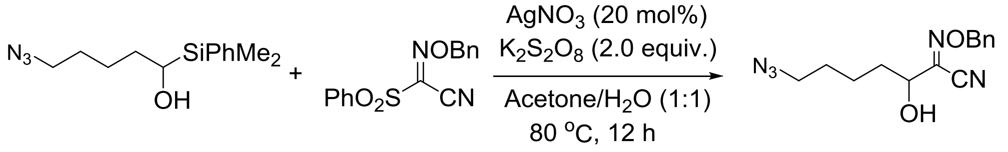

1e **2** **3e**

In an Ar protected glove box, $AgNO_3$ (6.8 mg, 0.04 mmol, 20 mol%), **2** (90.0 mg, 0.30 mmol, 1.5 equiv.) and **1e** (55.4 mg, 0.20 mmol) were added into a reaction tube. After that, the tube was taken out of the box, acetone/H_2O (0.75 mL / 0.75 mL) and $K_2S_2O_8$ (108.0 mg, 0.40 mmol, 2.0 equiv.) were added under N_2. The tube was then sealed, and the resulting mixture was kept stirring at 80 °C in heating block for 12 h. The reaction mixture was quenched with water (5 mL), extracted with ethyl acetate (3×10 mL) and organic phase was combined and washed with brine, dried over anhydrous Na_2SO_4, concentrated under reduced pressure. The crude product was purified with column chromatography on silica gel (200–300 mesh) with PE/EA (10/1, v/v) as eluent to afford 27.0 mg of the title compound as a faint yellow oil (47% yield).

R_f = 0.36 (PE/EA = 4/1, v/v). NMR Spectroscopy: ^{1}H NMR (400 MHz, $CDCl_3$, 25 °C) δ 7.41–7.32 (m, 5H), 5.27 (s, 2H), 4.41 (t, J = 6.8 Hz, 1H), 3.26 (t, J = 6.7 Hz, 2H), 1.98 (s, 1H), 1.85–1.71 (m, 2H), 1.66–1.57 (m, 2H), 1.55–1.37 (m, 2H); ^{13}C NMR (101 MHz, $CDCl_3$, 25 °C) δ 135.8, 135.3, 128.7, 128.6, 108.9, 78.4, 70.3, 51.2, 33.9, 28.5, 22.2. IR (ATR): 3422, 2930, 2863, 2095, 1454, 1364, 1260, 1006, 738, 701 cm^{-1}. HRMS (ESI, m/z): calcd for $C_{14}H_{17}N_5O_2Na^+$ $(M+Na)^+$: 310.1274; Found: 310.1275.

N-(Benzyloxy)-7-bromo-2-hydroxyheptanimidoyl cyanide (3f)

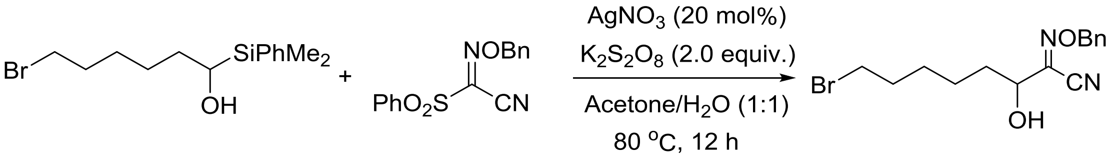

1f **2** **3f**

In an Ar protected glove box, $AgNO_3$ (6.8 mg, 0.04 mmol, 20 mol%), **2** (90.0 mg, 0.30 mmol, 1.5 equiv.)

and **1f** (67.6 mg, 0.20 mmol) were added into a reaction tube. After that, the tube was taken out of the box, acetone/H_2O (0.75 mL / 0.75 mL) and $K_2S_2O_8$ (108.0 mg, 0.40 mmol, 2.0 equiv.) were added under N_2. The tube was then sealed, and the resulting mixture was kept stirring at 80 °C in heating block for 12 h. The reaction mixture was quenched with water (5 mL), extracted with ethyl acetate (3×10 mL) and organic phase was combined and washed with brine, dried over anhydrous Na_2SO_4, concentrated under reduced pressure. The crude product was purified with column chromatography on silica gel (200–300 mesh) with PE/EA (10/1, v/v) as eluent to afford 40.5 mg of the title compound as a faint yellow oil (60% yield).

R_f = 0.22 (PE/EA = 4/1, v/v). NMR Spectroscopy: ^1H NMR (400 MHz, $CDCl_3$, 25 °C) δ 7.46–7.25 (m, 5H), 5.26 (s, 2H), 4.39 (dd, J = 9.6, 6.5 Hz, 1 H), 3.37 (t, J = 6.7 Hz, 2H), 2.34 (d, J = 4.1 Hz, 1H), 1.88–1.67 (m, 4H), 1.52–1.30 (m, 4H); ^{13}C NMR (101 MHz, $CDCl_3$, 25 °C) δ 135.8, 135.4, 128.7, 128.6, 108.9, 78.4, 70.5, 34.3, 33.7, 32.6, 27.7, 24.1. IR (ATR): 3451, 2937, 2863, 1454, 1211, 1081, 1003, 738, 697 cm^{-1}. HRMS (ESI, m/z): calcd for $C_{15}H_{23}BrN_3O_2^+$ $(M+NH_4)^+$: 356.0968; Found: 356.0968.

N,5-Bis(benzyloxy)-2-hydroxypentanimidoyl cyanide (3g)

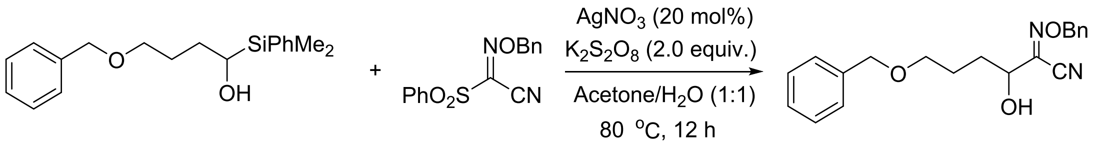

1g **2** **3g**

In an Ar protected glove box, $AgNO_3$ (6.8 mg, 0.04 mmol, 20 mol%), **2** (90.0 mg, 0.30 mmol, 1.5 equiv.) and **1g** (62.8 mg, 0.20 mmol) were added into a reaction tube. After that, the tube was taken out of the box, acetone/H_2O (0.75 mL / 0.75 mL) and $K_2S_2O_8$ (108.0 mg, 0.40 mmol, 2.0 equiv.) were added under N_2. The tube was then sealed, and the resulting mixture was kept stirring at 80 °C in heating block for 12 h. The reaction mixture was quenched with water (5 mL), extracted with ethyl acetate (3×10 mL) and organic phase was combined and washed with brine, dried over anhydrous Na_2SO_4, concentrated under reduced pressure. The crude product was purified with column chromatography on silica gel (200–300 mesh) with PE/EA (8/1, v/v) as eluent to afford 37.0 mg of the title compound as a faint yellow oil (55% yield).

R_f = 0.27 (PE/EA = 4/1, v/v). NMR Spectroscopy: ^1H NMR (400 MHz, $CDCl_3$, 25 °C) δ 7.4–7.29 (m, 10H), 5.26 (s, 2H), 4.53 (s, 2H), 4.41 (dd, J = 8.0, 4.9 Hz, 1H), 3.65–3.30 (m, 2H), 1.96–1.83 (m, 2H), 1.81–1.72 (m, 2H); ^{13}C NMR (101 MHz, $CDCl_3$, 25 °C) δ 137.7, 135.9, 135.7, 128.7, 128.6, 128.6, 128.0, 128.0, 109.2, 78.3, 73.4, 70.4, 69.9, 32.6, 25.7. IR (ATR): 3362, 2926, 2859, 1495, 1454, 1364, 1092, 1003, 738, 697 cm^{-1}. HRMS (ESI, m/z): calcd for $C_{20}H_{22}N_2O_3Na^+$ $(M+Na)^+$: 361.1523; Found: 361.1523.

N-(Benzyloxy)-2-hydroxy-6-phenoxyhexanimidoyl cyanide (3h)

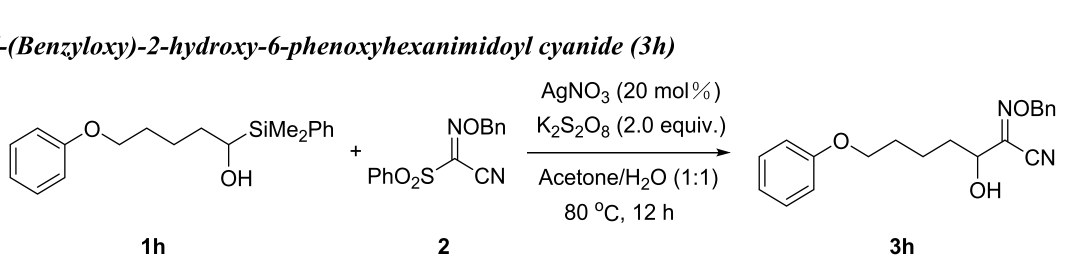

1h **2** **3h**

In an Ar protected glove box, $AgNO_3$ (6.8 mg, 0.04 mmol, 20 mol%), **2** (90.0 mg, 0.30 mmol, 1.5 equiv.) and **1h** (62.8 mg, 0.20 mmol) were added into a reaction tube. After that, the tube was taken out of the box, acetone/H_2O (0.75 mL / 0.75 mL) and $K_2S_2O_8$ (108.0 mg, 0.40 mmol, 2.0 equiv.) were added under

N_2. The tube was then sealed, and the resulting mixture was kept stirring at 80 °C in heating block for 12 h. The reaction mixture was quenched with water (5 mL), extracted with ethyl acetate (3×10 mL) and organic phase was combined and washed with brine, dried over anhydrous Na_2SO_4, concentrated under reduced pressure. The crude product was purified with column chromatography on silica gel (200–300 mesh) with PE/EA (8/1, v/v) as eluent to afford 40.0 mg of the title compound as a faint yellow oil (60% yield).

R_f =0.24 (PE/EA = 4/1, v/v). NMR Spectroscopy: ^{1}H NMR (400 MHz, $CDCl_3$, 25 °C) δ 7.41–7.32 (m, 5 H), 7.32–7.24 (m, 2H), 6.97–6.93 (m, 1H), 6.91–6.86 (m, 2H), 5.27 (s, 2H), 4.44 (t, J = 6.8 Hz, 1H), 3.95 (t, J = 6.3 Hz, 2H), 1.98 (s, 1H), 1.89–1.74 (m, 4H), 1.70–1.43 (m, 2H); ^{13}C NMR (101 MHz, $CDCl_3$, 25 °C) δ 159.0, 135.8, 135.4, 129.6, 128.8, 128.7, 128.6, 120.8, 114.6, 109.0, 78.5, 70.6, 67.5, 28.9, 28.3, 21.8. IR (ATR): 3422, 2930, 2863, 2095, 1454, 1364, 1260, 1006, 738, 701 cm^{-1}. HRMS (ESI, m/z): calcd for $C_{20}H_{23}N_2O_3^+$ (M+H)$^+$: 339.1703; Found: 339.1700.

N,5-Bis(benzyloxy)-2-hydroxypentanimidoyl cyanide (3i)

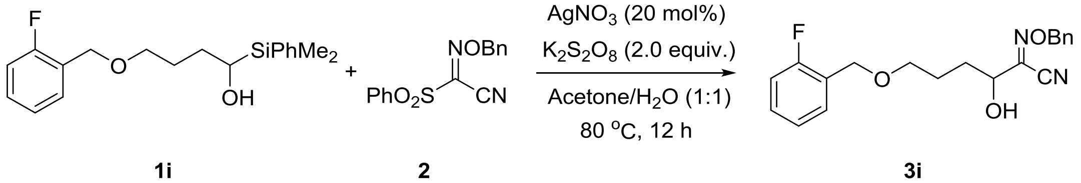

In an Ar protected glove box, $AgNO_3$ (13.6 mg, 0.08mmol, 20 mol%), **2** (180.0 mg, 0.60 mmol, 1.5 equiv.) and **1i** (132.8 mg, 0.40 mmol) were added into a reaction tube. After that, the tube was taken out of the box, aetone/H_2O (1.5 mL / 1.5 mL) and $K_2S_2O_8$ (216.0 mg, 0.80mmol, 2.0 equiv.) were added under N_2. The tube was then sealed, and the resulting mixture was kept stirring at 80 °C in heating block for 12 h. The reaction mixture was quenched with water (10 mL), extracted with ethyl acetate (3×20 mL) and organic phase was combined and washed with brine, dried over anhydrous Na_2SO_4, concentrated under reduced pressure. The crude product was purified with column chromatography on silica gel (200–300 mesh) with PE/EA (4/1, v/v) as eluent to afford 88.3 mg of the title compound as a faint yellow oil (62% yield).

R_f = 0.25 (PE/EA = 4/1, v/v). NMR Spectroscopy: ^{1}H NMR (400 MHz, $CDCl_3$, 25 °C) δ 7.45–7.27 (m, 6H), 7.14–6.92 (m, 3H), 5.26 (s, 2H), 4.50 (s, 2H), 4.43 (t, J = 6.2 Hz, 1H), 3.59–3.45 (m, 2H), 3.33 (s, 1H), 1.95–1.83 (m, 2H), 1.82–1.71 (m, 2H); ^{13}C NMR (101 MHz, $CDCl_3$, 25 °C) δ 160.9 (d, J = 246.9 Hz), 135.9, 135.6, 130.3 (d, J = 4.2 Hz), 129.8 (d, J = 8.2 Hz), 128.7, 128.7, 128.6, 124.8 (d, J = 14.7 Hz), 124.3 (d, J = 3.5 Hz), 115.5 (d, J = 21.4 Hz), 109.1, 78.3, 70.4, 70.1, 66.7 (d, J = 3.7 Hz), 32.4, 25.6. ^{19}F NMR (375 MHz, $CDCl_3$) δ −114.5 (m, 1F). IR (ATR): 3452, 2937, 2863, 1454, 1364, 1211, 1081, 1003, 734, 697 cm^{-1}. HRMS (ESI, m/z): calcd for $C_{20}H_{22}FN_2O_3^+$ (M+H)$^+$: 357.1614; Found: 357.1609.

N-(Benzyloxy)-5-((3-fluorobenzyl)oxy)-2-hydroxypentanimidoyl cyanide (3j)

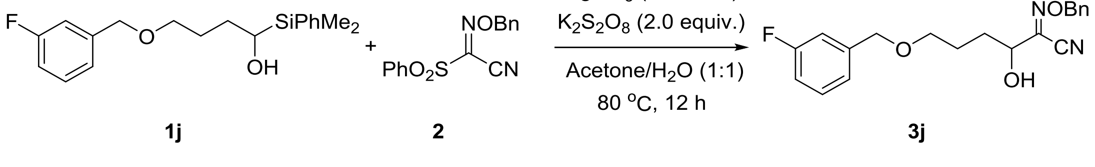

In an Ar protected glove box, $AgNO_3$ (13.6 mg, 0.08mmol, 20 mol%), **2** (132.8 mg, 0.60 mmol, 1.5 equiv.) and **1j** (132.8 mg, 0.40 mmol) were added into a reaction tube. After that, the tube was taken out

of the box, acetone/H_2O (1.5 mL / 1.5 mL), $K_2S_2O_8$ (216.0 mg, 0.80mmol, 2.0 equiv.) were added under N_2. The tube was then sealed, and the resulting mixture was kept stirring at 80 °C in heating block for 12 h. The reaction mixture was quenched with water (10 mL), extracted with ethyl acetate (3×20 mL) and organic phase was combined and washed with brine, dried over anhydrous Na_2SO_4, concentrated under reduced pressure. The crude product was purified with column chromatography on silica gel (200–300 mesh) with PE/EA (4/1, v/v) as eluent to afford 96.0 mg of the title compound as a faint yellow oil (67% yield).

R_f = 0.18 (PE/EA = 4/1, v/v). NMR Spectroscopy: ^{1}H NMR (400 MHz, $CDCl_3$, 25 °C) δ 7.42–7.26 (m, 7H), 7.14 (td, J = 7.5, 1.2 Hz, 1H), 7.08–7.02 (m, 1H), 5.26 (s, 2H), 4.58 (s, 2H), 4.41 (dd, J = 7.8, 5.1 Hz, 1H), 3.60–3.50 (m, 2H), 3.41 (s, 1H), 1.89 (m, 2H), 1.80–1.72 (m, 2H); ^{13}C NMR (101 MHz, $CDCl_3$, 25 °C) δ 160.9 (d, J = 246.8 Hz), 135.9, 135.6, 130.3 (d, J = 4.3 Hz), 129.8 (d, J = 8.2 Hz), 128.7 (d, J = 1.2 Hz), 128.7, 128.6, 124.9 (d, J = 15.0 Hz), 124.4 (d, J = 3.8 Hz), 115.5 (d, J = 21.5 Hz), 109.1, 78.3, 70.4, 70.1, 66.7 (d, J = 3.9 Hz), 32.4, 25.6. ^{19}F NMR (375 MHz, $CDCl_3$) δ –118.69– –118.87 (m, 1F). IR (ATR): 3452, 2937, 2863, 1454, 1364, 1211, 1081, 1003, 734, 697 cm^{-1}. HRMS (ESI, m/z): calcd for $C_{20}H_{22}FN_2O_3^+$ (M+H)$^+$: 357.1609; Found: 357.1609.

N-(Benzyloxy)-5-((4-fluorobenzyl)oxy)-2-hydroxypentanimidoyl cyanide (3k)

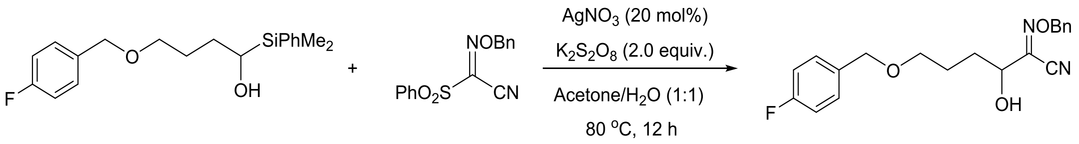

1k **2** **3k**

In an Ar protected glove box, $AgNO_3$ (13.6 mg, 0.08mmol, 20 mol%), **2** (180.0 mg, 0.60 mmol, 1.5 equiv.) and **1k** (132.8 mg, 0.40 mmol) were added into a reaction tube. After that, the tube was taken out of the box, acetone/H_2O (1.5 mL / 1.5 mL) and $K_2S_2O_8$ (216.0 mg, 0.80mmol, 2.0 equiv.) were added under N_2. The tube was then sealed, and the resulting mixture was kept stirring at 80 °C in heating block for 12 h. The reaction mixture was quenched with water (10 mL), extracted with ethyl acetate (3×20 mL) and organic phase was combined and washed with brine, dried over anhydrous Na_2SO_4, concentrated under reduced pressure. The crude product was purified with column chromatography on silica gel (200–300 mesh) with PE/EA (8/1, v/v) as eluent to afford 97.0 mg of the title compound as a faint yellow oil (68% yield).

R_f = 0.2 (PE/EA = 4/1, v/v). NMR Spectroscopy: ^{1}H NMR (400 MHz, $CDCl_3$, 25 °C) δ 7.41–7.32 (m, 5H), 7.33–7.27 (m, 2H), 7.08–7.00 (m, 2H), 5.26 (s, 2H), 4.48 (s, 2H), 4.41 (dd, J = 7.8, 5.0 Hz, 1H), 3.59–3.43 (m, 2H), 1.97–1.70 (m, 4H); ^{13}C NMR (101 MHz, $CDCl_3$, 25 °C) δ 162.6 (d, J = 246.0 Hz), 135.9, 135.7, 133.5 (d, J = 3.3 Hz), 129.73 (d, J = 8.2 Hz), 128.7, 128.7, 128.6, 115.53 (d, J = 21.5 Hz), 109.1, 78.4, 72.6, 70.4, 69.9, 32.6, 25.6. ^{19}F NMR (375 MHz, $CDCl_3$) δ –114.5 (m, 1F). IR (ATR): 3384, 2933, 2866, 1603, 1510, 1223, 1088, 1003, 734, 697 cm^{-1}. HRMS (ESI, m/z): calcd for $C_{20}H_{21}FN_2O_3Na^+$ (M+ Na)$^+$: 379.1428; Found: 379.1428.

N-(Benzyloxy)-5-((3-chlorobenzyl)oxy)-2-hydroxypentanimidoyl cyanide (3l)

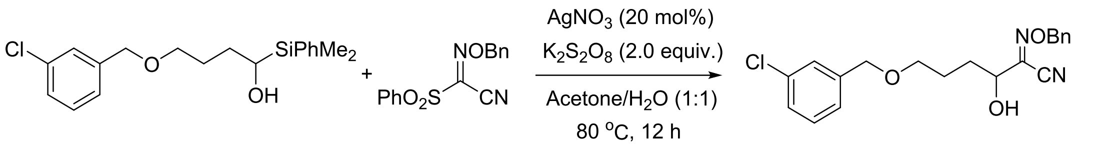

1l **2** **3l**

In an Ar protected glove box, $AgNO_3$ (13.6 mg, 0.08mmol, 20 mol%), **2** (180.0 mg, 0.60 mmol, 1.5 equiv.) and **1l** (139.2 mg, 0.40 mmol) were added into a reaction tube. After that, the tube was taken out of the box, Acetone/H_2O (1.5 mL / 1.5 mL) and $K_2S_2O_8$ (216.0 mg, 0.80mmol, 2.0 equiv.) were added under N_2. The tube was then sealed, and the resulting mixture was kept stirring at 80 °C in heating block for 12 h. The reaction mixture was quenched with water (10 mL), extracted with ethyl acetate (3×20 mL) and organic phase was combined and washed with brine, dried over anhydrous Na_2SO_4, concentrated under reduced pressure. The crude product was purified with column chromatography on silica gel (200–300 mesh) with PE/EA (8/1, v/v) as eluent to afford 98.2 mg of the title compound as a faint yellow oil (66% yield).

R_f = 0.24 (PE/EA = 4/1, v/v). NMR Spectroscopy: ^{1}H NMR (400 MHz, $CDCl_3$, 25 °C) δ 7.42–7.33 (m, 5H), 7.32–7.30 (m, 1H), 7.29–7.26 (m, 2H), 7.24–7.14 (m, 1H), 5.26 (s, 2H), 4.48 (s, 2H), 4.43 (dd, J = 7.9, 5.2 Hz, 1H), 3.62–3.26 (m, 2H), 1.95–1.83 (m, 2H), 1.81–1.71 (m, 2H); ^{13}C NMR (101 MHz, $CDCl_3$, 25 °C) δ 134.0, 135.9, 135.6, 134.5, 130.0, 128.7, 128.7, 128.6, 128.1, 127.9, 125.8, 109.1, 78.4, 72.5, 70.4, 70.1, 32.3, 25.5. IR (ATR): 3392, 2930, 2863, 1576, 1476, 1361, 1077, 1003, 734, 697 cm^{-1}. HRMS (ESI, m/z): calcd for $C_{20}H_{21}ClN_2O_3Na^+$ $(M+Na)^+$: 395.1133 ; Found: 395.1132.

N-(Benzyloxy)-5-((3-bromobenzyl)oxy)-2-hydroxypentanimidoyl cyanide (3m)

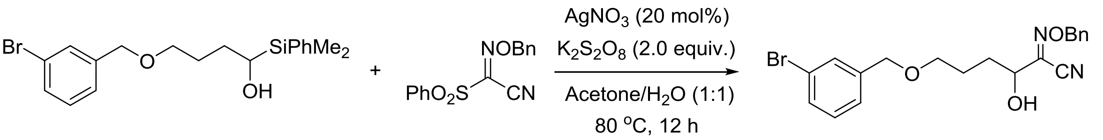

1m **2** **3m**

In an Ar protected glove box, $AgNO_3$ (6.8 mg, 0.04 mmol, 20 mol%), **2** (90.0 mg, 0.30 mmol, 1.5 equiv.) and **1m** (78.4 mg, 0.20 mmol) were added into a reaction tube. After that, the tube was taken out of the box, acetone/H_2O (0.75 mL / 0.75 mL) and $K_2S_2O_8$ (108.0 mg, 0.40 mmol, 2.0 equiv.) were added under N_2. The tube was then sealed, and the resulting mixture was kept stirring at 80 °C in heating block for 12 h. The reaction mixture was quenched with water (5 mL), extracted with ethyl acetate (3×10 mL) and organic phase was combined and washed with brine, dried over anhydrous Na_2SO_4, concentrated under reduced pressure. The crude product was purified with column chromatography on silica gel (200–300 mesh) with PE/EA (10/1, v/v) as eluent to afford 51.0 mg of the title compound as a faint yellow oil (61% yield).

R_f = 0.23 (PE/EA = 4/1, v/v). NMR Spectroscopy: ^{1}H NMR (400 MHz, $CDCl_3$, 25 °C) δ 7.47 (t, J = 1.4 Hz, 1H), 7.43 (m, 1H), 7.39–7.30 (m, 5H), 7.30–7.18 (m, 2H), 5.26 (s, 2H), 4.47 (s, 2H), 4.43 (m, 1H), 3.56–3.44 (m, 2H), 3.30 (s, 1H), 1.95–1.80 (m, 2H), 1.80–1.68 (m, 2H); ^{13}C NMR (101 MHz, $CDCl_3$, 25 °C) δ 140.3, 135.9, 135.6, 131.0, 130.8, 130.2, 128.71, 128.7, 128.6, 126.3, 122.7, 109.1, 78.4, 72.4, 70.4, 70.1, 32.3, 25.5. IR (ATR): 3399, 2926, 2859, 1573, 1454, 1208, 1070, 999, 738, 697 cm^{-1}. HRMS (ESI, m/z): calcd for $C_{20}H_{21}BrN_2O_3Na^+$ $(M+Na)^+$: 439.0628; Found: 439.0628.

N-(Benzyloxy)-5-((2-bromobenzyl)oxy)-2-hydroxypentanimidoyl cyanide (3n)

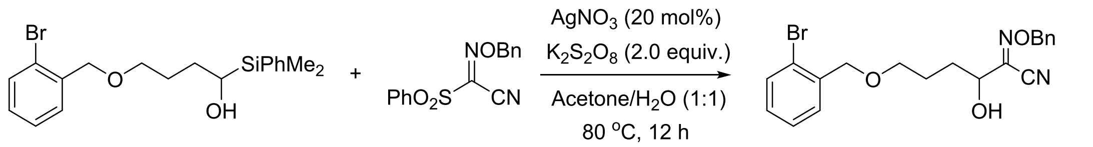

1n **2** **3n**

In an Ar protected glove box, $AgNO_3$ (6.8 mg, 0.04 mmol, 20 mol%), **2** (90.0 mg, 0.30 mmol, 1.5 equiv.) and **1n** (78.4 mg, 0.20 mmol) were added into a reaction tube. After that, the tube was taken out of the box, acetone/H_2O (0.75 mL / 0.75 mL) and $K_2S_2O_8$ (108.0 mg, 0.40 mmol, 2.0 equiv.) were added under N_2. The tube was then sealed, and the resulting mixture was kept stirring at 80 °C in heating block for 12 h. The reaction mixture was quenched with water (5 mL), extracted with ethyl acetate (3×10 mL) and organic phase was combined and washed with brine, dried over anhydrous Na_2SO_4, concentrated under reduced pressure. The crude product was purified with column chromatography on silica gel (200–300 mesh) with PE/EA (10/1, v/v) as eluent to afford 47.3 mg of the title compound as a faint yellow oil (57% yield).

R_f = 0.25 (PE/EA = 4/1, v/v). NMR Spectroscopy: ^{1}H NMR (400 MHz, $CDCl_3$, 25 °C) δ 7.47 (s, 1H), 7.44–7.40 (m, 1H), 7.40–7.31 (m, 5H), 7.27–7.18 (m, 2H), 5.26 (s, 2H), 4.48 (s, 2H), 4.43 (dd, J = 12.8, 4.8 Hz, 1H), 3.58–3.45 (m, 2H), 3.24 (d, J = 4.8 Hz, 1H), 1.98–1.82 (m, 2H), 1.82–1.70 (m, 2H); ^{13}C NMR (101 MHz, $CDCl_3$, 25 °C) δ 140.23, 135.9, 135.6, 131.0, 130.8, 130.3, 128.7, 128.7, 128.6, 126.3, 125.2, 109.1, 78.4, 72.5, 70.5, 70.1, 32.4, 25.5. IR (ATR): 3384, 2926, 2855, 1662, 1361, 1260, 1085, 1006, 910, 731 cm^{-1}. HRMS (ESI, m/z): calcd for $C_{20}H_{22}BrN_2O_3^+$ $(M+H)^+$: 417.0808; Found: 417.0808.

N-(Benzyloxy)-5-((2-cyanobenzyl)oxy)-2-hydroxypentanimidoyl cyanide (3o)

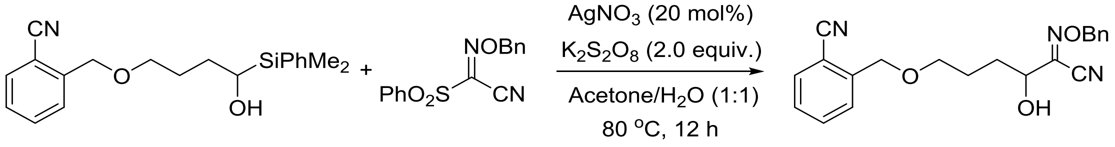

1o **2** **3o**

In an Ar protected glove box, $AgNO_3$ (13.6 mg, 0.08mmol, 20 mol%), **2** (180.0 mg, 0.60 mmol, 1.5 equiv.) and **1o** (135.6 mg, 0.40 mmol) were added into a reaction tube. After that, the tube was taken out of the box, acetone/H_2O (1.5 mL / 1.5 mL), $K_2S_2O_8$ (216.0 mg, 0.80mmol, 2.0 equiv.) were added under N_2. The tube was then sealed, and the resulting mixture was kept stirring at 80 °C in heating block for 12 h. The reaction mixture was quenched with water (10 mL), extracted with ethyl acetate (3×20 mL) and organic phase was combined and washed with brine, dried over anhydrous Na_2SO_4, concentrated under reduced pressure. The crude product was purified with column chromatography on silica gel (200–300 mesh) with PE/EA (4/1, v/v) as eluent to afford 74.0 mg of the title compound as a faint yellow oil (51% yield).

R_f = 0.33 (PE/EA = 2/1, v/v). NMR Spectroscopy: ^{1}H NMR (400 MHz, $CDCl_3$, 25 °C) δ 7.66-7.63 (m, 1H) 7.60-7.56 (m, 1H), 7.48-7.45 (m, 1H), 7.42-7.34 (m, 6H), 5.32 (s, 2H), 4.61 (d, J = 1.2 Hz, 2H), 4.24-4.20 (m, 1H), 3.69-3.61 (m, 2H), 2.07-1.98 (m, 1H), 1.87-1.77 (m, 1H), 1.68-1.57 (m, 2H).^{13}C NMR (101 MHz, $CDCl_3$, 25 °C) δ 140.6, 135.8, 133.7, 133.2, 133.0, 129.3, 128.8, 128.7, 128.7, 117.4, 111.9, 108.7, 78.6, 77.9, 69.0, 62.2, 29.7, 28.0. IR (ATR): 3437, 2922, 2855, 1718, 1454, 1364, 1211, 1084, 1002, 764 cm^{-1}. HRMS (ESI, m/z): calcd for $C_{21}H_{21}N_3O_3Na^+$ $(M+ Na)^+$: 386.1475; Found: 386.1475.

N-(Benzyloxy)-2-hydroxy-7-phenoxyheptanimidoyl cyanide (3p)

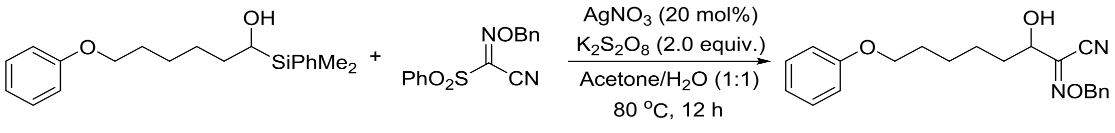

1p **2** **3p**

In an Ar protected glove box, $AgNO_3$ (6.8 mg, 0.04mmol, 20 mol%), **2** (90.0 mg, 0.30mmol, 1.5 equiv.) and **1p** (70.4 mg, 0.20 mmol) were added into a reaction tube. After that, the tube was taken out of the box, acetone/H_2O (0.75 mL / 0.75 mL) and $K_2S_2O_8$ (108.0 mg, 0.40mmol, 2.0 equiv.) were added under N_2. The tube was then sealed, and the resulting mixture was kept stirring at 80 °C in heating block for 12 h. The reaction mixture was quenched with water (5 mL), extracted with ethyl acetate (3×10 mL) and organic phase was combined and washed with brine, dried over anhydrous Na_2SO_4, concentrated under reduced pressure. The crude product was purified with column chromatography on silica gel (200–300 mesh) with PE/EA (8/1, v/v) as eluent to afford 40.0 mg of the title compound as a faint yellow oil (57% yield).

R_f = 0.26 (PE/EA = 4/1, v/v). NMR Spectroscopy: ^{1}H NMR (400 MHz, $CDCl_3$, 25 °C) δ 7.40–7.33 (m, 5H), 7.31–7.25 (m, 2H), 6.96–6.94 (m, 1H), 6.92–6.86 (m, 2H), 5.27 (s, 2H), 4.42 (t, J = 6.7 Hz, 1H), 3.95 (t, J = 6.4 Hz, 2H), 2.29 (s, 1H), 1.84–1.72 (m, 4H), 1.56–1.35 (m, 4H); ^{13}C NMR (101 MHz, $CDCl_3$, 25 °C) δ 159.1, 135.8, 135.5, 129.5, 128.7, 128.6, 120.7, 114.6, 108.9, 78.4, 70.6, 67.6, 34.4, 29.2, 25.8, 24.6. IR (ATR): 3459, 2937, 2863, 1599, 1495, 1245, 1003, 813, 753, 693 cm^{-1}. HRMS (ESI, m/z): calcd for $C_{21}H_{24}N_2O_3Na^+$ $(M+Na)^+$: 375.1679; Found: 375.1679.

7-((Benzyloxy)imino)-7-cyano-6-hydroxyheptyl benzoate (3q)

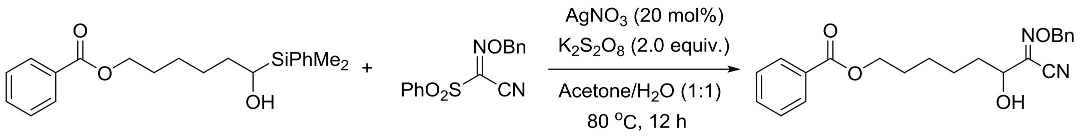

1q **2** **3q**

In an Ar protected glove box, $AgNO_3$ (3.4 mg, 0.02 mmol, 20 mol%), **2** (45.0 mg, 0.15 mmol, 1.5 equiv.) and **1q** (35.6 mg, 0.10 mmol) were added into a reaction tube. After that, the tube was taken out of the box, acetone/H_2O (0.375 mL / 0.375 mL) and $K_2S_2O_8$ (54.0 mg, 0.20 mmol, 2.0 equiv.) were added under N_2. The tube was then sealed, and the resulting mixture was kept stirring at 80 °C in heating block for 12 h. The reaction mixture was quenched with water (5 mL), extracted with ethyl acetate (3×10 mL) and organic phase was combined and washed with brine, dried over anhydrous Na_2SO_4, concentrated under reduced pressure. The crude product was purified with column chromatography on silica gel (200–300 mesh) with PE/EA (10/1, v/v) as eluent to afford 22.0 mg of the title compound as a faint yellow oil (58% yield).

R_f = 0.24 (PE/EA = 4/1, v/v). NMR Spectroscopy: ^{1}H NMR (400 MHz, $CDCl_3$, 25 °C) δ 8.04 (m, 2H), 7.60–7.51 (m, 1H), 7.48–7.40 (m, 2H), 7.40–7.28 (m, 5H), 5.25 (s, 2H), 4.41 (t, J = 6.7 Hz, 1H), 4.30 (t, J = 6.6 Hz, 2H), 2.59 (s, 1H), 1.88–1.66 (m, 4H), 1.59–1.35 (m, 4H); ^{13}C NMR (101 MHz, $CDCl_3$, 25 °C) δ 166.9, 135.8, 135.6, 133.0, 130.4, 129.7, 128.7, 128.7, 128.6, 128.5, 109.0, 78.4, 70.5, 64.9, 34.4, 28.7, 25.7, 24.6. IR (ATR): 3444, 2937, 2863, 1715, 1454, 1275, 1118, 1069, 1003, 716 cm^{-1}. HRMS (ESI, m/z): calcd for $C_{22}H_{24}N_2O_4Na^+$ $(M+Na)^+$: 403.1628; Found: 403.1628.

N,7-Bis(benzyloxy)-2-hydroxyheptanimidoyl cyanide (3r)

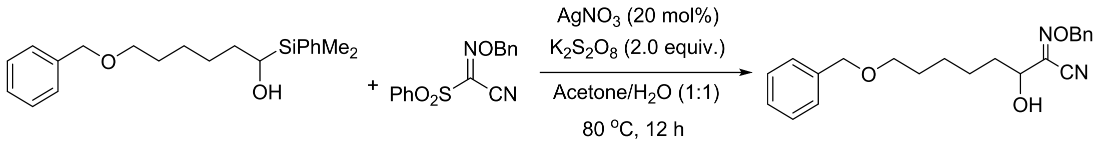

1r **2** **3r**

In an Ar protected glove box, and $AgNO_3$ (6.8 mg, 0.04 mmol, 20 mol%), **2** (90.0 mg, 0.30 mmol, 1.5 equiv.) and **1r** (68.4 mg, 0.20 mmol) were added into a reaction tube. After that, the tube was taken out of the box, acetone/H_2O (0.75 mL / 0.75 mL) and $K_2S_2O_8$ (108.0 mg, 0.40 mmol, 2.0 equiv.) were added under N_2. The tube was then sealed, and the resulting mixture was kept stirring at 80 °C in heating block for 12 h. The reaction mixture was quenched with water (5 mL), extracted with ethyl acetate (3×10 mL) and organic phase was combined and washed with brine, dried over anhydrous Na_2SO_4, concentrated under reduced pressure. The crude product was purified with column chromatography on silica gel (200–300 mesh) with PE/EA (8/1, v/v) as eluent to afford 48.0 mg of the title compound as a faint yellow oil (66% yield).

R_f = 0.25 (PE/EA = 4/1, v/v). NMR Spectroscopy: ^{1}H NMR (400 MHz, $CDCl_3$, 25 °C) δ 7.40–7.30 (m, 10H), 5.26 (s, 2H), 4.49 (s, 2H), 4.38 (t, J = 6.8 Hz, 1H), 3.46 (t, J = 6.5 Hz, 2H), 2.2 (s, 1H) 1.80–1.72 (m, 2H), 1.61 (m, 2H), 1.46–1.36 (m, 4H); ^{13}C NMR (101 MHz, $CDCl_3$, 25 °C) δ 138.7, 135.8, 135.6, 128.8, 128.7, 128.6, 128.5, 127.8, 127.7, 109.0, 78.4, 73.1, 70.6, 70.2, 34.5, 29.6, 25.9, 24.7. IR (ATR): 3392, 2937, 2863, 1454, 1364, 1201, 1073, 1003, 734, 697 cm^{-1}. HRMS (ESI, m/z): calcd for $C_{22}H_{27}N_2O_3^+$ (M+H)$^+$: 389.1841; Found: 389.1835.

***N*-(Benzyloxy)-2-hydroxy-5-(3-phenylpropoxy)pentanimidoyl cyanide (3s)**

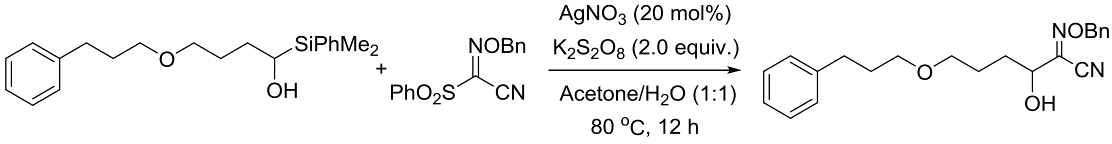

1s **2** **3s**

In an Ar protected glove box, and $AgNO_3$ (13.6 mg, 0.08mmol, 20 mol%), **2** (180.0 mg, 0.60 mmol, 1.5 equiv.) and **1s** (136.8 mg, 0.40 mmol) was added into a reaction tube. After that, the tube was taken out of the box, acetone/H_2O (1.5 mL / 1.5 mL) and $K_2S_2O_8$ (216.0 mg, 0.80mmol, 2.0 equiv.) were added under N_2. The tube was then sealed, and the resulting mixture was kept stirring at 80 °C in heating block for 12 h. The reaction mixture was quenched with water (10 mL), extracted with ethyl acetate (3×20 mL) and organic phase was combined and washed with brine, dried over anhydrous Na_2SO_4, concentrated under reduced pressure. The crude product was purified with column chromatography on silica gel (200–300 mesh) with PE/EA (8/1, v/v) as eluent to afford 89.0 mg of the title compound as a faint yellow oil (61% yield).

R_f = 0.24 (PE/EA = 4/1, v/v). NMR Spectroscopy: ^{1}H NMR (400 MHz, $CDCl_3$, 25 °C) δ 7.40–7.32 (m, 5H), 7.32–7.26 (m, 2H), 7.23–7.14 (m, 3H), 5.26 (s, 2H), 4.43 (dd, J = 8.3, 4.4 Hz, 1H), 4.25–3.90 (m, 1H), 3.67–3.33 (m, 4H), 2.71–2.66 (m, 2H), 1.99–1.85 (m, 4H), 1.81–1.68 (m, 2H); ^{13}C NMR (101 MHz, $CDCl_3$, 25 °C) δ 141.7, 135.9, 135.8, 128.7, 128.6, 128.6, 128.6, 128.5, 126.0, 109.2, 78.3, 70.6, 70.5, 70.5, 33.1, 32.4, 31.1, 25.9. IR (ATR): 3377, 2933, 2863, 1454, 1364, 1289, 1111, 1003, 745, 697 cm^{-1}. HRMS (ESI, m/z): calcd for $C_{22}H_{27}N_2O_3^+$ (M+H)$^+$: 367.2016; Found: 367.2016.

***N*-(Benzyloxy)-2-hydroxy-7-((4-methylbenzyl)oxy)heptanimidoyl cyanide (3t)**

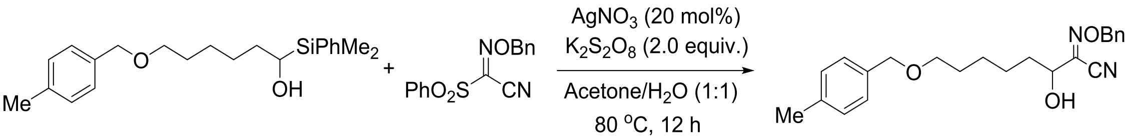

1t **2** **3t**

In an Ar protected glove box, $AgNO_3$ (13.6 mg, 0.08mmol, 20 mol%), **2** (160.0 mg, 0.60 mmol, 1.5 equiv.) and **1t** (151.4 mg, 0.40 mmol) were added into a reaction tube. After that, the tube was taken out of the box, acetone/H_2O (0.75 mL / 0.75 mL) and $K_2S_2O_8$ (108.0 mg, 0.40 mmol, 2.0 equiv.) were added under N_2. The tube was then sealed, and the resulting mixture was kept stirring at 80 °C in heating block for 12 h. The reaction mixture was quenched with water (5 mL), extracted with ethyl acetate (3×10 mL) and organic phase was combined and washed with brine, dried over anhydrous Na_2SO_4, concentrated under reduced pressure. The crude product was purified with column chromatography on silica gel (200–300 mesh) with PE/EA (8/1, v/v) as eluent to afford 44.8 mg of the title compound as a faint yellow oil (57% yield).

R_f = 0.31 (PE/EA = 4/1, v/v). NMR Spectroscopy: ^{1}H NMR (400 MHz, $CDCl_3$, 25 °C) δ 7.43–7.30 (m, 5H), 7.19 (m, 4H), 5.26 (s, 2H), 4.45 (s, 2H), 4.38 (t, J = 6.8 Hz, 1H), 3.43 (t, J = 6.4 Hz, 2H), 2.34 (s, 3H), 1.79–1.72 (m, 2H), 1.59 (m, 2H), 1.49–1.28 (m, 4H); ^{13}C NMR (101 MHz, $CDCl_3$, 25 °C) δ 137.4, 135.8, 135.6, 135.6, 129.2, 128.8, 128.7, 128.6, 127.9, 109.0, 78.4, 72.9, 70.6, 70.1, 34.5, 29.6, 25.9, 24.7, 21.3. IR (ATR): 3384, 2933, 2859, 1454, 1364, 1207, 1080, 1003, 734, 697 cm^{-1}. HRMS (ESI, m/z): calcd for $C_{23}H_{28}N_2O_3Na^+$ $(M+Na)^+$: 403.1992; Found: 403.1992.

N,6-Bis(benzyloxy)-2-hydroxyhexanimidoyl cyanide (3u)

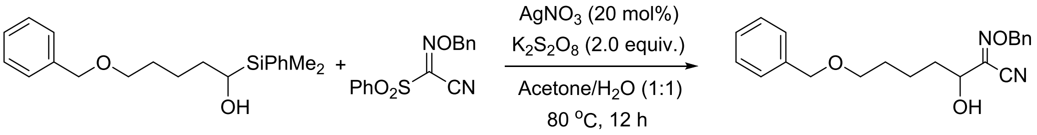

1u **2** **3u**

In an Ar protected glove box, $AgNO_3$ (6.8 mg, 0.04 mmol, 20 mol%), **2** (90.0 mg, 0.30 mmol, 1.5 equiv.) and **1u** (65.6 mg, 0.20 mmol) were added into a reaction tube. After that, the tube was taken out of the box, acetone/H_2O (0.75 mL / 0.75 mL) and $K_2S_2O_8$ (108.0 mg, 0.40 mmol, 2.0 equiv.) were added under N_2. The tube was then sealed, and the resulting mixture was kept stirring at 80 °C in heating block for 12 h. The reaction mixture was quenched with water (5 mL), extracted with ethyl a cetate (3×10 mL) and organic phase was combined and washed with brine, dried over anhydrous Na_2SO_4, concentrated under reduced pressure. The crude product was purified with column chromatography on silica gel (200–300 mesh) with PE/EA (8/1, v/v) as eluent to afford 48.0 mg of the title compound as a faint yellow oil (68% yield).

R_f = 0.25 (PE/EA = 4/1, v/v). NMR Spectroscopy: ^{1}H NMR (400 MHz, $CDCl_3$, 25 °C δ 7.41–7.29 (m, 10H), 5.26 (s, 2H), 4.50 (s, 2H), 4.39 (t, J = 6.2 Hz, 1H), 3.46 (t, J = 6.3 Hz, 2H), 2.50 (s, 1H), 1.82–1.74 (m, 2H), 1.71–1.60 (m, 2H), 1.59–1.36 (m, 2H); ^{13}C NMR (101 MHz, $CDCl_3$, 25 °C) δ 138.5, 135.8, 135.6, 128.7, 128.7, 128.6, 128.5, 127.8, 127.8, 109.0, 78.4, 73.1, 70.6, 70.0, 34.3, 29.2, 21.8. IR (ATR): 3377, 2937, 2863, 1454, 1364, 1260, 1092, 1006, 738, 697 cm^{-1}. HRMS (ESI, m/z): calcd for $C_{21}H_{24}N_2O_3Na^+$ $(M+Na)^+$: 375.1685; Found: 375.1860.

N-(Benzyloxy)-2-hydroxy-5-(isopentyloxy)pentanimidoyl cyanide(3v)

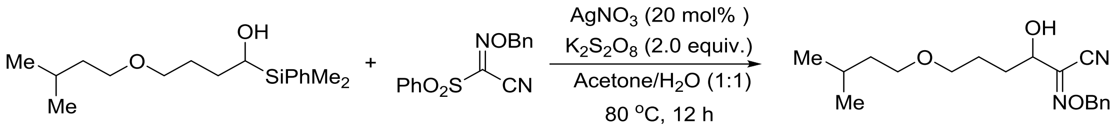

In an Ar protected glove box, $AgNO_3$ (6.8 mg, 0.04 mmol, 20 mol%), **2** (90.0 mg, 0.30 mmol, 1.5 equiv.) and **1v** (58.8 mg, 0.20 mmol) were added into a reaction tube. After that, the tube was taken out of the box, acetone/H_2O (0.75 mL / 0.75 mL) and $K_2S_2O_8$ (108.0 mg, 0.40 mmol, 2.0 equiv.) were added under N_2. The tube was then sealed, and the resulting mixture was kept stirring at 80 °C in heating block for 12 h. The reaction mixture was quenched with water (5 mL), extracted with ethyl acetate (3×10 mL) and organic phase was combined and washed with brine, dried over anhydrous Na_2SO_4, concentrated under reduced pressure. The crude product was purified with column chromatography on silica gel (200–300 mesh) with PE/EA (8/1, v/v) as eluent to afford 45.0 mg of the title compound as a faint yellow oil (70% yield).

R_f = 0.31 (PE/EA = 4/1, v/v). NMR Spectroscopy: ^{1}H NMR (400 MHz, $CDCl_3$, 25 °C) δ 7.39–7.31 (m, 5H), 5.26 (s, 2H), 4.40 (dd, J = 8.4, 4.2 Hz, 1H), 4.23 (s, 1H), 3.54–3.40 (m, 4H), 1.98–1.81 (m, 2H), 1.81–1.71 (m, 2H), 1.68 (m, 1H), 1.48 (m, 2H), 0.90 (d, J = 6.6 Hz, 6H); ^{13}C NMR (101 MHz, $CDCl_3$, 25 °C) δ 136.0, 135.9, 128.7, 128.6, 128.6, 109.2, 78.3, 70.6, 70.5, 70.0, 38.3, 33.3, 26.0, 25.2, 22.74, 22.70. IR (ATR): 3358, 2870, 2952, 1454, 1364, 1096, 1006, 921, 738, 697 cm^{-1}. HRMS (ESI, m/z): calcd for $C_{18}H_{26}N_2O_3Na^+$ $(M+Na)^+$: 341.1834; Found: 341.1835.

N-(Benzyloxy)-2-hydroxy-7-((4-(trifluoromethoxy)benzyl)oxy)heptanimidoyl cyanide(3w)

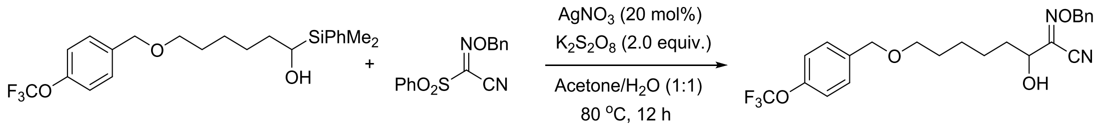

In an Ar protected glove box, and $AgNO_3$ (13.6 mg, 0.08mmol, 20 mol%), **2** (180.0 mg, 0.60mmol, 1.5 equiv.) and **1w** (170.4 mg, 0.40 mmol) were added into a reaction tube. After that, the tube was taken out of the box, acetone/H_2O (1.5 mL / 1.5 mL) and $K_2S_2O_8$ (216.0 mg, 0.80mmol, 2.0 equiv.) were added under N_2. The tube was then sealed, and the resulting mixture was kept stirring at 80 °C in heating block for 12 h. The reaction mixture was quenched with water (10 mL), extracted with ethyl acetate (3×20 mL) and organic phase was combined and washed with brine, dried over anhydrous Na_2SO_4, concentrated under reduced pressure. The crude product was purified with column chromatography on silica gel (200–300 mesh) with PE/EA (8/1, v/v) as eluent to afford 75.0 mg of the title compound as a faint yellow oil (50% yield).

R_f = 0.38 (PE/EA = 4/1, v/v). NMR Spectroscopy: ^{1}H NMR (400 MHz, $CDCl_3$, 25 °C) δ 7.43–7.30 (m, 7H), 7.22–7.16 (m, 2H), 5.26 (s, 2H), 4.48 (s, 2H), 4.39 (t, J = 6.8 Hz, 1H), 3.46 (t, J = 6.4 Hz, 2H), 1.82–1.71 (m, 2H), 1.61 (dt, J = 12.8, 6.5 Hz, 2H), 1.45–1.33 (m, 4H); ^{13}C NMR (101 MHz, $CDCl_3$, 25 °C) δ 148.71 (dd, J = 4.2, 2.1 Hz), 137.4, 135.84, 135.6, 129.0, 128.7, 128.7, 128.5, 121.0 (dd, J = 2.3, 1.0 Hz), 120.61 (q, J = 256.8 Hz), 109.0, 78.3, 72.1, 70.5, 70.5, 34.4, 29.5, 25.9, 24.7. ^{19}F NMR (375 MHz, $CDCl_3$) δ –57.8 (s, 3F). IR (ATR): 3388, 2930, 2855, 1613, 1509, 1256, 1021, 920, 816, 752 cm^{-1}. HRMS (ESI, m/z): calcd for $C_{23}H_{26}F_3N_2O_4^+$ $(M+H)^+$: 451.1838; Found: 451.1839.

N-(Benzyloxy)-5-(2-(benzyloxy)ethoxy)-2-hydroxypentanimidoyl cyanide (3x)

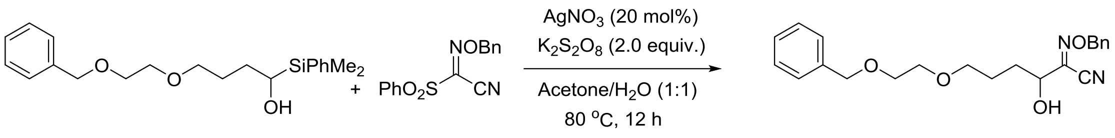

1x **2** **3x**

In an Ar protected glove box, $AgNO_3$ (13.6 mg, 0.08 mmol, 20 mol%), **2** (180 mg, 0.60 mmol, 1.5 equiv.) and **1x** (143.2 mg, 0.40 mmol) were added into a reaction tube. After that, the tube was taken out of the box, acetone/H_2O (1.5 mL / 1.5 mL) and $K_2S_2O_8$ (216.0 mg, 0.80mmol, 2.0 equiv.) were added under N_2. The tube was then sealed, and the resulting mixture was kept stirring at 80 °C in heating block for 12 h. The reaction mixture was quenched with water (10 mL), extracted with ethyl acetate (3×20 mL) and organic phase was combined and washed with brine, dried over anhydrous Na_2SO_4, concentrated under reduced pressure. The crude product was purified with column chromatography on silica gel (200–300 mesh) with PE/EA (8/1, v/v) as eluent to afford 99.0 mg of the title compound as a faint yellow oil (65% yield).

R_f = 0.17 (PE/EA = 4/1, v/v). NMR Spectroscopy: ^1H NMR (400 MHz, $CDCl_3$, 25 °C) δ 7.39–7.30 (m, 10H), 5.26 (s, 2H), 4.56 (s, 2H), 4.45 (dd, J = 7.3, 5.6 Hz, 1H), 3.66–3.59 (m, 4H), 3.59–3.47 (m, 2H), 1.92–1.84 (m, 2H), 1.81–1.66 (m, 2H); ^{13}C NMR (101 MHz, $CDCl_3$, 25 °C) δ 138.1, 136.0, 135.9, 128.7, 128.6, 128.5, 128.5, 128.0, 127.8, 109.2, 78.2, 73.5, 71.0, 70.3, 70.3, 69.3, 32.8, 25.6. IR (ATR): 3384, 2922, 2866, 1454, 1357, 1084, 1003, 909, 730, 697 cm^{-1}. HRMS (ESI, m/z): calcd for $C_{22}H_{27}N_2O_4^+$ $(M+H)^+$: 383.1971; Found: 383.1965.

N-(benzyloxy)-2-hydroxy-3-methylpentanimidoyl cyanide (3y)

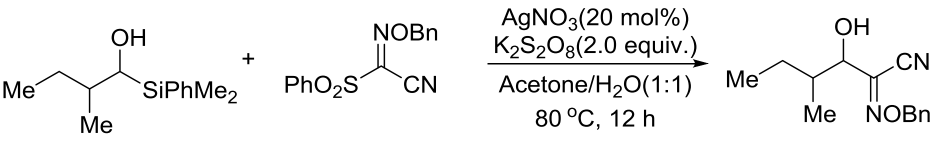

1y 2 3y

In an Ar protected glove box, $AgNO_3$ (6.8 mg, 0.04 mmol, 20 mol%), **2** (90 mg, 0.30 mmol, 1.5 equiv.) and **1y** (44.4 mg, 0.20 mmol) were added into a reaction tube. After that, the tube was taken out of the box, acetone/H_2O (0.75 mL / 0.75 mL) and $K_2S_2O_8$ (108.0 mg, 0.40mmol, 2.0 equiv.) were added under N_2. The tube was then sealed, and the resulting mixture was kept stirring at 80 °C in heating block for 12 h. The reaction mixture was quenched with water (10 mL), extracted with ethyl acetate (3×20 mL) and organic phase was combined and washed with brine, dried over anhydrous Na_2SO_4, concentrated under reduced pressure. The crude product was purified with column chromatography on silica gel (200–300 mesh) with PE/EA (10/1, v/v) as eluent to afford 32.0 mg of the title compound as a yellow oil (65% yield).

R_f = 0.47 (PE/EA = 4/1, v/v). NMR Spectroscopy: ^1H NMR (400 MHz, $CDCl_3$, 25 °C) δ 7.39–7.32 (m, 5H), 5.28 (d, J = 2.4 Hz, 2H), 4.24 (t, J = 5.7 Hz, 0.61H), 4.16 (dd, J = 7.7, 5.1 Hz, 0.39H), 2.33 (dd, J = 11.1, 5.5 Hz, 1H), 1.87–1.76 (m, 1H), 1.68–1.40 (m, 1H), 1.27–1.15 (m, 1H), 0.96–0.88 (m, 6H); ^{13}C NMR (151 MHz, $CDCl_3$, 25 °C) δ 135.9, 135. 3, 128.8, 128.7, 128.7, 128.6, 128.6, 109.2, 78.4, 78.37, 74.8, 74.2, 38.7, 38.6, 25.6, 24.3, 14.9, 13.6, 11.4, 11.0. IR (ATR): 3037, 2963, 2333, 1945, 1453, 1412, 1259, 1080 cm^{-1}. HRMS (ESI, m/z): calcd for C14H19N2O2+ (M+H)+: 247.1441; Found: 247.1434.

N-(Benzyloxy)-2-ethyl-2-hydroxyhexanimidoyl cyanide (3z)

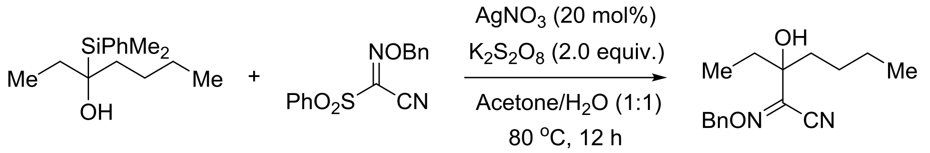

1z 2 3z

In an Ar protected glove box, and $AgNO_3$ (6.8 mg, 0.04mmol, 20 mol%), **2** (90.0 mg, 0.30mmol, 1.5 equiv.), **1a** (50.0 mg, 0.20 mmol) was added into a reaction tube. After that, the tube was taken out of the box, and Acetone/H_2O (0.75 mL / 0.75mL), $K_2S_2O_8$ (108.0 mg, 0.40mmol, 2.0 equiv.) was added under N_2. The tube was then sealed, and the resulting mixture was kept stirring at 80 °C in heating block for 12 h. The reaction mixture was quenched with water (5 mL), extracted with ethyl acetate (3×10 mL) and organic phase was combined and washed with brine, dried over anhydrous Na_2SO_4, concentrated under reduced pressure. The crude product was purified with column chromatography on silica gel (200–300 mesh) with PE/EA (20/1, v/v) as eluent to afford 11.0 mg of the title compound as a faint yellow oil (20% yield).

R_f = 0.46 (PE/EA = 4/1, v/v). NMR Spectroscopy: ^{1}H NMR (400 MHz, $CDCl_3$, 25 °C) δ 7.40–7.32 (m, 5H), 5.30 (s, 2H), 2.58 (s, 1H), 1.79–1.69 (m, 4H), 1.31–1.24 (m, 4H), 0.89–0.81 (m, 6H); ^{13}C NMR (151 MHz, $CDCl_3$, 25 °C) δ 137.8, 135.9, 128.7, 128.7, 128.5, 109.2, 78.4, 38.6, 32.0, 25.3, 22.9, 14.1, 7.4. IR (ATR): 3481, 2930, 2859, 2356, 1722, 1457, 1364, 1275, 1126, 1010, 742 cm^{-1}. HRMS (ESI, m/z): calcd for $C_{16}H_{22}N_2O_2Na+$ $(M+ Na)^+$: 297.1574; Found: 297.1566.

N-(benzyloxy)-2-hydroxy-2,5-dimethylhexanimidoyl cyanide (3aa)

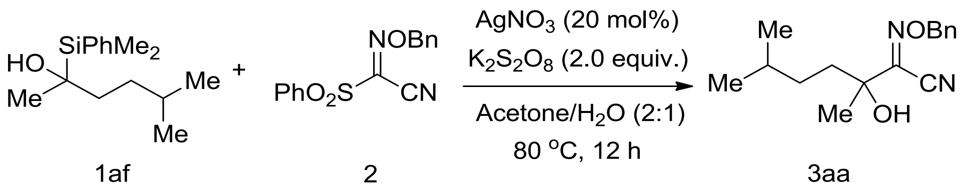

1af 2 3aa

In an Ar protected glove box, and $AgNO_3$ (6.8 mg, 0.04mmol, 20 mol%), **2** (90.0 mg, 0.30mmol, 1.5 equiv.), **1aa** (50.0 mg, 0.20 mmol) was added into a reaction tube. After that, the tube was taken out of the box, and Acetone/H_2O (1.0 mL / 0.5 mL), $K_2S_2O_8$ (108.0 mg, 0.40mmol, 2.0 equiv.) was added under N_2. The tube was then sealed, and the resulting mixture was kept stirring at 80 °C in heating block for 12 h. The reaction mixture was quenched with water (5 mL), extracted with ethyl acetate (3×10 mL) and organic phase was combined and washed with brine, dried over anhydrous Na_2SO_4, concentrated under reduced pressure. The crude product was purified with column chromatography on silica gel (200–300 mesh) with PE/EA (20/1, v/v) as eluent to afford 17.0 mg of the title compound as a faint yellow oil (31% yield).

R_f = 0.70 (PE/EA = 9/1, v/v). yellow oil, (17 mg, 31% yield). ^{1}H NMR (400 MHz, $CDCl_3$, 25 °C) δ 7.40–7.33 (m, 5H), 5.28 (s, 2H), 2.44 (s, 1H), 1.73 (dd, J = 9.5, 7.5 Hz, 2H), 1.53–1.48 (m, 1H), 1.46 (s, 3H), 1.20–1.02 (m, 2H), 0.86 (d, J = 4.0 Hz, 6H)； ^{13}C NMR (151 MHz, $CDCl_3$, 25 °C) δ 138.5, 135.9, 128.7, 128.7, 128.6, 109.2, 78.4, 74.2, 38.4, 32.3, 28.2, 26.3, 22.6, 22.6. IR (ATR): 3440, 2956, 2870, 1454, 1368, 1133, 1006, 913, 865, 734 cm^{-1}. HRMS (ESI, m/z): calcd for $C_{16}H_{20}N_2O^+$ $(M+H-H_2O)^+$: 257.1648; Found:257.1641.

(E)-N-(benzyloxy)-2-hydroxy-2-methylhexanimidoyl cyanide (3ab)

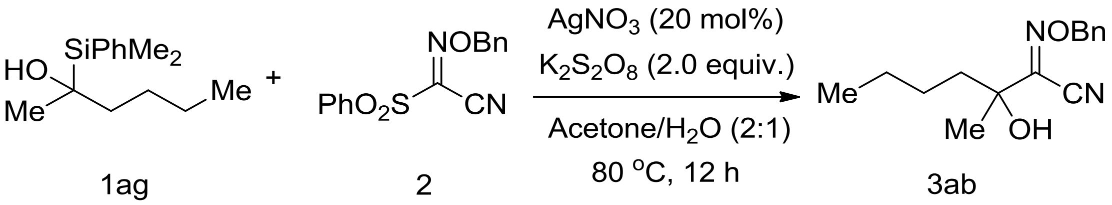

In an Ar protected glove box, and $AgNO_3$ (6.8 mg, 0.04mmol, 20 mol%), **2** (90.0 mg, 0.30mmol, 1.5 equiv.), **1ab** (47.2 mg, 0.20 mmol) was added into a reaction tube. After that, the tube was taken out of the box, and Acetone/H_2O (1.0 mL / 0.5 mL), $K_2S_2O_8$ (108.0 mg, 0.40mmol, 2.0 equiv.) was added under N_2. The tube was then sealed, and the resulting mixture was kept stirring at 80 ºC in heating block for 12 h. The reaction mixture was quenched with water (5 mL), extracted with ethyl acetate (3×10 mL) and organic phase was combined and washed with brine, dried over anhydrous Na_2SO_4, concentrated under reduced pressure. The crude product was purified with column chromatography on silica gel (200–300 mesh) with PE/EA (20/1, v/v) as eluent to afford 21.0 mg of the title compound as yellow oil (41% yield). R_f = 0.70 (PE/EA = 9/1, v/v). (21 mg, 41% yield). ^{1}H NMR (400 MHz, $CDCl_3$, 25 ºC) δ 7.40–7.32 (m, 5H), 5.28 (s, 1H), 2.44 (s, 1H), 1.73 (t, J = 8.0 Hz, 2H), 1.46 (s, 3H), 1.33–1.17 (m, 4H), 0.88 (t, J = 8.0 Hz, 3H); ^{13}C NMR (151 MHz, $CDCl_3$, 25 ºC) δ 138.5, 135.8, 128.7, 128.7, 128.6, 109.2, 78.4, 74.2, 40.2, 26.3, 25.6, 22.9, 14.1. IR (ATR): 3362, 2960, 1700, 1454, 1252, 1118, 1044, 910, 831, 731 cm^{-1}. HRMS (ESI, m/z): calcd for $C_{15}H_{18}N_2O^+$ (M+H-H_2O)$^+$: 243.1492; Found:243.1484

3. Mechanism study

a) Radical scavenger experiment with TEMPO

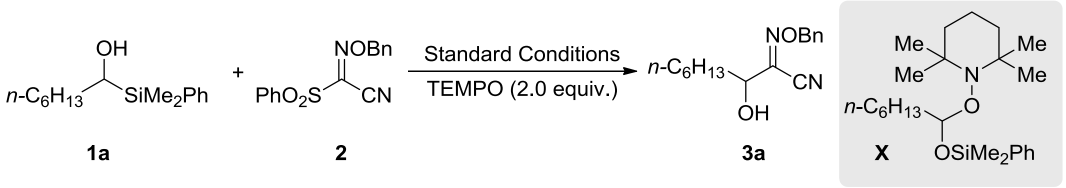

Entry	variation from standard conditions	conversion (**1a**)	conversion (**2**)	Yield (**3a**)
1	no variation	68%	17%	7%
2	without $K_2S_2O_8$	7%	11%	0%
3	without $AgNO_3$	48%	20%	13%
4	without $K_2S_2O_8$ and $AgNO_3$	2%	2%	0%

Reaction conditions: the mixture of **1a** (0.2 mmol), $AgNO_3$ (0.04 mmol), $K_2S_2O_8$ (0.4 mmol), **2** (0.3 mmol) in acetone/H_2O (v/v = 1/1, 1.5 mL) was stirred at 80 °C under N_2 for 12 h. The yield was determined by ^{1}H NMR using mesitylene as an internal standard.

Figure S-1

Conditions for entry 1: in an Ar protected glove box, $AgNO_3$ (6.8 mg, 0.04 mmol, 20 mol%), **2** (90.0 mg, 0.30 mmol, 1.5 equiv.), **1a** (50 mg, 0.20 mmol) was added into a reaction tube. After that, the tube was taken out of the box, Acetone/H_2O (0.75 mL / 0.75 mL), $K_2S_2O_8$ (108.0 mg, 0.40 mmol, 2.0 equiv.) and Tempo (62.4 mg, 0.40mmol, 2.0 equiv.) were added under N_2. The tube was then sealed, and the resulting

mixture was kept stirring at 80 °C in heating block for 12 h. The reaction mixture was quenched with water (5 mL), extracted with ethyl acetate (3×10 mL) and organic phase was combined and washed with brine, dried over anhydrous Na_2SO_4, concentrated under reduced pressure. 7% yield of **3a**. The yield was determined by ^1H NMR spectroscopy using mesitylene as internal standard. Compound X was detected by HRMS: calcd for $C_{24}H_{44}NO_2Si^+$ $(M+H)^+$: 406.3141; Found: 406.3136.
Three control experiments were also performed (Figure S-1, entries 2~4). Without $AgNO_3$ and $K_2S_2O_8$, there were only 2% conversion of **1a** and **2**, without formation of any **3a** (Figure S-1, entry 4), indicating that the addition of TEMPO is less likely to change the mechanism.

b) Reaction of heptyldimethyl(phenyl)silane 1a-1 under standard conditions.

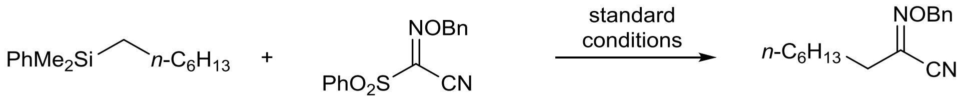

1a-1 **2** **3a-1**

In an Ar protected glove box, $AgNO_3$ (6.8 mg, 0.04mmol, 20 mol%), **1a-1** (46.8 mg, 0.20 mmol, 1.0 equiv.) and **2** (90.0 mg, 0.30 mmol) were added into a reaction tube. After that, the tube was taken out of the box, acetone/H_2O (0.75 mL / 0.75 mL) and $K_2S_2O_8$ (108.0 mg, 0.80mmol, 2.0 equiv.) were added under N_2. The tube was then sealed, and the resulting mixture was kept stirring at 80 °C in heating block for 12 h. The reaction mixture was quenched with water (5 mL), extracted with ethyl acetate (3×10 mL) and organic phase was combined and washed with brine, dried over anhydrous Na_2SO_4, concentrated under reduced pressure. 0% yield of **3a-1**. The yield was determined by ^1H NMR spectroscopy with Mesitylene as internal standard.

c) Reaction of protected α-silyl alcohol 1a-2 under standard conditions.

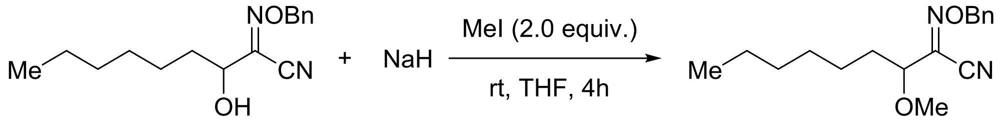

3a **3a-2**

Under N_2 atmosphere, **3a** (54.8 mg, 0.2 mmol), NaH (9.6 mg, 0.24 mmol, 1.2 equiv., 60 % in oil) , MeI (56.8 mg, 0.4 mmol, 2.0 equiv.) was added into a reaction tube. The tube was then sealed, and the resulting mixture was kept stirring at room temperature for 4 h. The reaction mixture was quenched with water (10 mL) , extracted with ether (3×10 mL) and organic phase was combined and washed with brine, dried over anhydrous Na_2SO_4, concentrated under reduced pressure. The crude product was purified with column chromatography on silica gel (200–300 mesh) with PE as eluent to afford 33.0 mg of the title compound as a faint yellow oil (57% yield). Two isomer (5.55:1).
R_f = 0.9 (PE). NMR Spectroscopy: ^1H NMR (400 MHz, $CDCl_3$, 25 °C) δ 7.58–7.26 (m, 5 H), 5.29 (s, 2H), 3.82 (dd, J = 7.4, 6.9 Hz, 1H), 3.31 (s, 3H), 3.25 (s, 3H), 1.38–1.17 (m, 10H), 0.91–0.81 (m, 3H); ^{13}C NMR (101 MHz, $CDCl_3$, 25 °C) δ 136.0, 134.5, 128.7, 128.6, 128.5, 108.8, 79.6, 78.3, 57.1, 33.0, 31.7, 29.0, 24.9, 22.6, 14.2. IR (ATR): 2930, 2859, 2863, 1454, 1364, 1260, 1096, 1003, 738, 697 cm^{-1}. HRMS (ESI, m/z): calcd for $C_{17}H_{25}N_2O_2^+$ $(M+H)^+$: 289.1911; Found: 289.1910

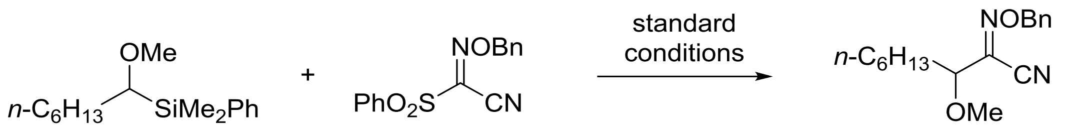

1a-2 **2** **3a-2**

In an Ar protected glove box, $AgNO_3$ (6.8 mg, 0.04mmol, 20 mol%), **2** (90.0 mg, 0.30 mmol, 1.5equiv.) and **1a-2** (158.5 mg, 0.60 mmol, 1.5 equiv.) were added into a reaction tube. After that, the tube was taken out of the box, acetone/H_2O (0.75 mL / 0.75 mL) and $K_2S_2O_8$ (108.0 mg, 0.40mmol, 2.0 equiv.) were added under N_2. The tube was then sealed, and the resulting mixture was kept stirring at 80 °C in heating block for 12 h. The reaction mixture was quenched with water (10 mL), extracted with ethyl acetate (3×20 mL) and organic phase was combined and washed with brine, dried over anhydrous Na_2SO_4, concentrated under reduced pressure. By comparing the crude spectrum of the reaction system with the standard spectrum, it can be konw that the peak at 3.82(dd, J = 7.4, 6.9 Hz, 1 H) is the characteristic peak and the yield is 5%. The yield was determined by ^{1}H NMR spectroscopy using Mesitylene as internal standard.

d) Reaction of α-silyl alcohol containing OSiMe₂tBu group 1a-3 under standard conditions.

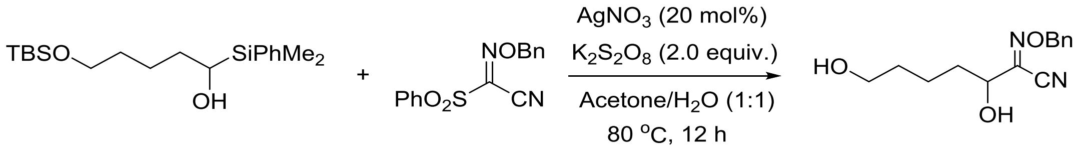

1a-3 **2** **3a-3**

In an Ar protected glove box, $AgNO_3$ (13.6 mg, 0.08mmol, 20 mol%), **1a–3(**180.0 mg, 0.60 mmol, 1.5 equiv.), **2** (140.8 mg, 0.40 mmol) was added into a reaction tube. After that, the tube was taken out of the box, and Acetone/H_2O (1.5 mL / 1.5 mL), and $K_2S_2O_8$ (216.0 mg, 0.80mmol, 2.0 equiv.) was added under N_2. The tube was then sealed, and the resulting mixture was kept stirring at 80 °C in heating block for 12 h. The reaction mixture was quenched with water (10 mL), extracted with ethyl acetate (3×20 mL) and organic phase was combined and washed with brine, dried over anhydrous Na_2SO_4, concentrated under reduced pressure. The crude product was purified with column chromatography on silica gel (200–300 mesh) with PE/EA (2/1, v/v) as eluent to afford 46.0 mg of the title compound as a faint yellow oil (44% yield).

R_f = 0.57 (EA). NMR Spectroscopy: ^{1}H NMR (400 MHz, $CDCl_3$, 25 °C) δ 7.42–7.31 (m, 5H), 5.26 (s, 2H), 4.41 (dd, J = 7.5, 6.0 Hz, 1H), 3.63 (t, J = 6.2 Hz, 2H), 2.00 (s, 2H), 1.86–1.70 (m, 2H), 1.65–1.35 (m, 4H); ^{13}C NMR (101 MHz, $CDCl_3$, 25 °C) δ 135.9, 135.6, 128.8, 128.7, 128.6, 109.0, 78.4, 70.5, 62.6, 34.2, 32.0, 21.3. IR (ATR): 3340, 2933, 2863, 1454, 1364, 1293, 1006, 917, 738, 701 cm^{-1}. HRMS (ESI, m/z): calcd for $C_{14}H_{19}N_2O_3^+$ $(M+H)^+$: 263.1390; Found: 263.1390.

e) Reaction of α-triphenylsilyl alcohol 1a-4 under conditions without H₂O.

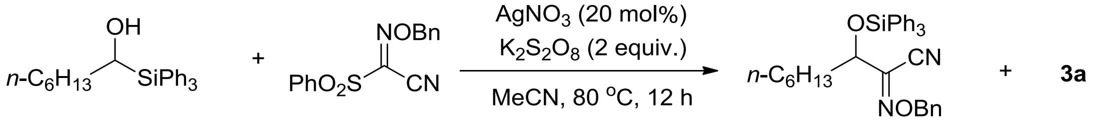

1a-4 **2** **3a-4**

In an Ar protected glove box, $AgNO_3$ (6.8 mg, 0.04mmol, 20 mol%), **2** (90.0 mg, 0.30 mmol, 1.5 equiv.), **1a–4** (74.8 mg, 0.20 mmol) was added into a reaction tube. After that, the tube was taken out of the box, and MeCN (1.5 mL), $K_2S_2O_8$ (108.0 mg, 0.40mmol, 2.0 equiv.) was added under N_2. The tube was then sealed, and the resulting mixture was kept stirring at 80 °C in heating block for 12 h. The reaction mixture was quenched with water (10 mL), extracted with ethyl acetate (3×20 mL) and organic phase was combined and washed with brine, dried over anhydrous Na_2SO_4, concentrated under reduced pressure. The crude product was purified with column chromatography on silica gel (300–400 mesh) with PE/EA (10/1, v/v) as eluent to afford 13.0 mg of the **3a–4** as a faint colorless oil (10% yield) and 1 mg of **3a** as yellow oil (2% yield).

R_f = 0.52 (PE/EA = 4/1, v/v). Colorless oil (13.0 mg, 0.02 mmol, 12 % yield). NMR spectroscopy: ^1H NMR (400 MHz, $CDCl_3$, 25 °C) δ 7.60–7.55 (m, 7H), 7.46–7.43 (m, 4H), 7.38–7.31 (m, 9H), 5.03 (d, J = 2.7 Hz, 2H), 4.50 (t, J = 7.0 Hz, 1H), 1.90–1.68 (m, 2H), 1.23–1.12 (m, 8H), 0.83 (t, J = 7.0 Hz, 3H); ^{13}C NMR (151 MHz, $CDCl_3$, 25 °C) δ 135.6, 133.4, 130.4, 128.6, 128.58, 128.4, 128.1, 128.1, 109.1, 78.0, 72.0, 35.5, 31.6, 29.8, 28.8, 24.6, 22.6, 14.2. IR (ATR): 3071, 2926, 2855, 1487, 1417, 1114, 1013, 916, 738, 700 cm^{-1}. HRMS (ESI, m/z): calcd for $C_{34}H_{36}SiN_2O_2Na^+$ $(M+Na)^+$: 555.2444; Found: 555.2438.

f) Reaction of heptan-1-ol 1a-5 under standard conditions

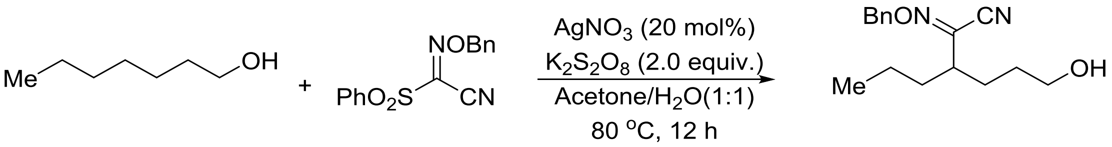

1a-5 **2** **3a-5**

In an Ar protected glove box, $AgNO_3$ (6.8 mg, 0.04mmol, 20 mol%), **2** (90.0 mg, 0.30 mmol, 1.5 equiv.) and **1a–5** (23.2 mg, 0.20 mmol) were added into a reaction tube. After that, the tube was taken out of the box, acetone/H_2O (0.75 mL / 0.75 mL) and $K_2S_2O_8$ (108.0 mg, 0.40 mmol, 2.0 equiv.) were added under N_2. The tube was then sealed, and the resulting mixture was kept stirring at 80 °C in heating block for 12 h. The reaction mixture was quenched with water (10 mL), extracted with ethyl acetate (3×10 mL) and organic phase was combined and washed with brine, dried over anhydrous Na_2SO_4, concentrated under reduced pressure. The crude product was purified with column chromatography on silica gel (200–300 mesh) with PE/EA (4/1, v/v) as eluent to afford 22.0 mg of the title compound as a faint yellow oil (40% yield).

R_f = 0.54 (PE/EA = 4/1, v/v). NMR Spectroscopy: ^1H NMR (400 MHz, $CDCl_3$, 25 °C) δ 7.51–7.28 (m, 5H), 5.24 (s, 2H), 3.57 (t, J = 6.4 Hz, 2H), 2.54–2.49 (m, 1H), 1.58–1.42 (m, 6H), 1.33–1.16 (m, 2H), 0.87 (t, J = 7.3 Hz, 3H); ^{13}C NMR (101 MHz, $CDCl_3$, 25 °C) δ 137.0, 136.4, 128.7, 128.6, 128.4, 109.5, 77.7, 62.3, 41.9, 34.7, 30.0, 28.7, 20.2, 13.8. IR (ATR): 3381, 2933, 2870, 1454, 1364, 1245, 1002, 917, 738, 697 cm^{-1}. HRMS (ESI, m/z): calcd for $C_{16}H_{23}N_2O_2^+$ $(M+H)^+$: 275.1745; Found: 275.1754.

g) Reaction of α-silyl alcohol containing remote OH group 1a-6 under standard conditions.

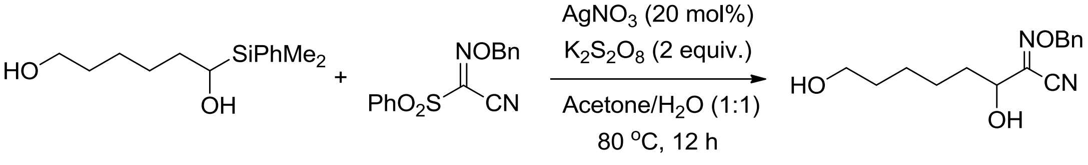

1a-6 **2** **3a-3**

In an Ar protected glove box, $AgNO_3$ (6.8 mg, 0.04 mmol, 20 mol%), **2** (90.0 mg, 0.30 mmol, 1.5 equiv.) and **1a–6**(47.6 mg, 0.20 mmol) were added into a reaction tube. After that, the tube was taken out of the box, acetone/H_2O (0.75 mL / 0.75 mL) and $K_2S_2O_8$ (108.0 mg, 0.40 mmol, 2.0 equiv.) were added under N_2. The tube was then sealed, and the resulting mixture was kept stirring at 80 °C in heating block for 12 h. The reaction mixture was quenched with water (5 mL), extracted with ethyl acetate (3×10 mL) and organic phase was combined and washed with brine, dried over anhydrous Na_2SO_4, concentrated under reduced pressure. The crude product was purified with column chromatography on silica gel (200–300 mesh) with PE/EA (10/1, v/v) as eluent to afford 20.0 mg of the title compound as a faint yellow oil (38% yield).

R_f = 0.19 (PE/EA = 2/1, v/v). NMR Spectroscopy: ^{1}H NMR (600 MHz, $CDCl_3$, 25 °C) δ 7.39–7.33 (m, 5H), 5.26 (s, 0H), 4.41–4.38 (m, 1H), 3.62 (t, J = 6.3 Hz, 2H), 3.09 (s, 1H), 1.83–1.72 (m, 4H), 1.62–1.53 (m, 2H); ^{13}C NMR (151 MHz, $CDCl_3$, 25 ℃) δ 135.8, 135.5, 128.6, 128.6, 128.5, 109.0, 78.3, 70.3, 62.4, 34.0, 31.8, 21.2. IR (ATR): 3366, 2922, 2855, 1662, 1580, 1495, 1453, 1367, 1010 cm^{-1}. HRMS (ESI, m/z): calcd for $C_{14}H_{18}BrN_2O_3Na^+$ $(M+Na)^+$: 286.1243 Found: 286.1243.

h) By-product monitoring experiments

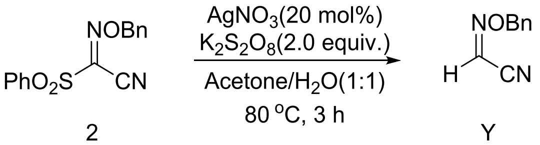

2 Y

In an Ar protected glove box, $AgNO_3$ (3.4 mg, 0.02 mmol, 20 mol%), **2** (45.0 mg, 0.15 mmol, 1.5 equiv.) was added into a reaction tube. After that, the tube was taken out of the box, Acetone/H_2O (1:1, 0.75 mL), $K_2S_2O_8$ (54.0 mg, 0.20 mmol, 2.0 equiv.) was added under N_2. The tube was then sealed, and the resulting mixture was kept stirring at 80 °C in heating block for 3 h. The reaction mixture was quenched with water (5 mL), extracted with ethyl acetate (3×10 mL) and organic phase was combined and washed with brine, dried over anhydrous Na_2SO_4, concentrated under reduced pressure. <3% yield of **Y**. The yield was determined by ^{1}H NMR spectroscopy using 2-bromotrimethylbenzene as internal standard. Compound **Y** was also confirmed by HRMS: calcd for $C_9H_9N_2O^+$ $(M+H)^+$: 161.0709; Found: 161.0718.

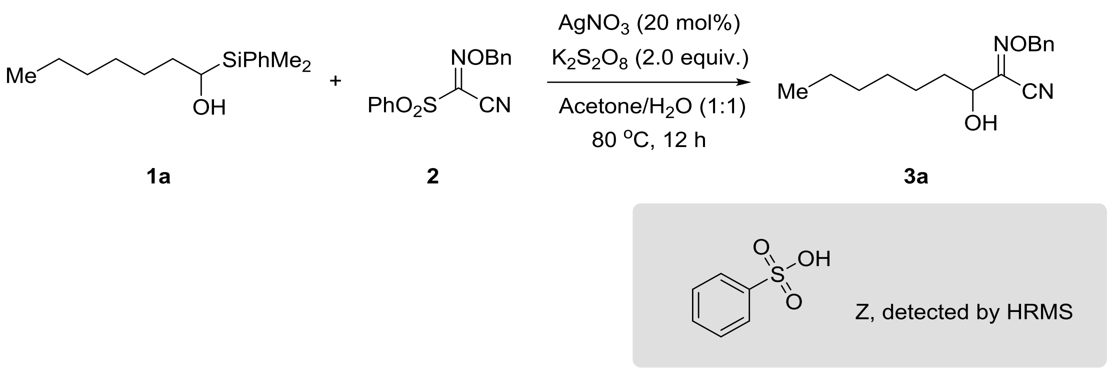

In an Ar protected glove box, $AgNO_3$ (6.8 mg, 0.04mmol, 20 mol%), **2** (90.0 mg, 0.30mmol, 1.5 equiv.) and **1a** (50.0 mg, 0.20 mmol) were added into a reaction tube. After that, the tube was taken out of the box, acetone/H_2O (0.75 mL / 0.75mL) and $K_2S_2O_8$ (108.0 mg, 0.40mmol, 2.0 equiv.) were added under N_2. The tube was then sealed, and the resulting mixture was kept stirring at 80 °C in heating block for 12 h. The reaction mixture was quenched with water (5 mL), extracted with ethyl acetate (3×10 mL) and organic phase was combined and washed with brine, dried over anhydrous Na_2SO_4, concentrated under reduced pressure. The formation of $PhSO_3H$ was confirmed by HRMS: calcd for $C_6H_5O_3S^-$ $(M{-}H)^-$: 156.9965; Found: 156.9962.

i) Computational methods

All of the DFT calculations were performed with the Gaussian 09 series of programs.[1] The M06-2X [2] functional and standard def2-TZVP [3] basis set were used for geometry optimization in the solvent phase. Harmonic vibrational frequency calculations were performed for all of the stationary points to confirm whether they are local minima or transition structures and to derive the enthalpies and thermochemical corrections for the free energies.

j) Complete reference for Gaussian 09

Frisch, M. J.; Trucks, G. W.; Schlegel, H. B.; Scuseria, G. E.; Robb, M. A.; Cheeseman, J. R.; Scalmani, G.; Barone, V.; Mennucci, B.; Petersson, G. A.; Nakatsuji, H.; Caricato, M.; Li, X.; Hratchian, H. P.; Izmaylov, A. F.; Bloino, J.; Zheng, G.; Sonnenberg, J. L.; Hada, M.; Ehara, M.; Toyota, K.; Fukuda, R.; Hasegawa, J.; Ishida, M.; Nakajima, T.; Honda, Y.; Kitao, O.; Nakai, H.; Vreven, T.; Montgomery, Jr., J. A.; Peralta, J. E.; Ogliaro, F.; Bearpark, M.; Heyd, J. J.; Brothers, E.; Kudin, K. N.; Staroverov, V. N.; Keith, T.; Kobayashi, R.; Normand, J.; Raghavachari, K.; Rendell, A.; Burant, J. C.; Iyengar, S. S.; Tomasi, J.; Cossi, M.; Rega, N.; Millam, J. M.; Klene, M.; Knox, J. E.; Cross, J. B.; Bakken, V.; Adamo, C.; Jaramillo, J.; Gomperts, R.; Stratmann, R. E.; Yazyev, O.; Austin, A. J.; Cammi, R.; Pomelli, C.; Ochterski, J. W.; Martin, R. L.; Morokuma, K.; Zakrzewski, V. G.; Voth, G. A.; Salvador, P.; Dannenberg, J. J.; Dapprich, S.; Daniels, A. D.; Farkas, O.; Foresman, J. B.; Ortiz, J. V.; Cioslowski, J.; and Fox, D. J. Gaussian 09, revision D.01; Gaussian, Inc.: Wallingford, CT, **2013**.

k) M06-2X/def2-TZVP calculated absolute energies, enthalpies, and free energies of all structures

Table S1. M06-2X/def2-TZVP calculated absolute energies, enthalpies, and free energies of all structures

Geometry	$E_{(elec\text{-}M06\text{-}2X)}$[1]	$H_{(corr\text{-}M06\text{-}2X)}$[2]	$G_{(corr\text{-}M06\text{-}2X)}$[3]	IF[4]
**A**	-951.296797	0.387945	0.314753	-

**B-ts**	-951.293482	0.386801	0.313463	-244.78
**C-ts**	-951.266657	0.381218	0.307324	-1642.74
**D**	-951.338050	0.387487	0.313685	-
**E**	-951.287505	0.386754	0.313282	-

[1]The electronic energy calculated by M06-2X in acetonitrile solvent. [2]The thermal correction to enthalpy calculated by M06-2X in acetonitrile solvent. [3]The thermal correction to Gibbs free energy calculated by M06-2X in acetonitrile solvent. [4]The M06-2X calculated imaginary frequencies for the transition states.

l) Single Point Calculation at the Pathways for 1,2-SiT vs 1,5-H AT (various methods)

Table S2. Single Point Calculation at the Pathways for 1,2-SiT vs 1,5-H AT(various methods)

**Entry**	**Δ*G*(solv) by M06-2X/ def2-TZVP**	**Δ*G*(solv) by M06-2X-D3/ def2-TZVP**	**Δ*G*(solv) By ωB97XD/ def2-TZVP**	**Δ*G*(solv) by B3-PW91/ def2-TZVP**	**Δ*G*(solv) by PBE/ def2-TZVP**
**A**	0.0	0.0	0.0	0.0	0.0
**B-ts**	1.3	1.3	2.3	2.0	1.8
**C-ts**	14.3	14.1	14.1	12.2	12.1
**D**	-26.6	-26.6	-22.5	-21.5	-21.6
**E**	4.9	4.8	6.7	7.7	7.1

m) Single Point Calculation at the Pathways for 1,2-SiT vs 1,5-H AT (various basis set)

Table S3. Single Point Calculation at the Pathways for 1,2-SiT vs 1,5-H AT(various basis set)

**Entry**	**Δ*G*(solv) by M06-2X/ def2-TZVP**	**Δ*G*(solv) by M06-2X/ def2-SVP**	**Δ*G*(solv) by M06-2X/ 6-311+g(d,p)**	**Δ*G*(solv) by M06-2X/ 6-31+g(d,p)**	**Δ*G*(solv) by M06-2X/ def2-QZVPP**
**A**	0.0	0.0	0.0	0.0	0.0
**B-ts**	1.3	2.1	3.0	2.4	1.2
**C-ts**	14.3	12.6	14.0	13.7	14.4
**D**	-26.6	-24.9	-22.3	-22.5	-26.6
**E**	4.9	3.8	4.2	5.0	4.7

n) M06-2X geometries for all the optimized compounds and transition states

A

C -0.64706400 -0.02239400 -1.37998100

H	-0.69091700	0.76779300	-2.14766700
H	0.24849900	-0.63246600	-1.58804800
C	-0.51819900	0.63447000	-0.00027300
H	-1.07512900	1.60176700	0.07983200
C	-1.88769200	-0.91730700	-1.44827800
H	-1.77839500	-1.67833900	-0.65889100
H	-1.89021100	-1.46069700	-2.40517700
C	-3.21524600	-0.16359400	-1.24957100
H	-3.78223500	-0.13263100	-2.19439700
O	-0.62960300	-0.13919100	1.05267700
H	-3.01512600	0.88977600	-0.98858200
C	-4.08993400	-0.77324800	-0.15738400
H	-4.29154000	-1.83220300	-0.39911300
H	-3.51547200	-0.77987100	0.78683200
C	-5.41084600	-0.04213600	0.05376800
H	-5.20309400	1.01500200	0.29193700
H	-5.97649900	-0.03627100	-0.89356200
C	-6.26304600	-0.65860700	1.15654800
H	-7.20963400	-0.11505400	1.29156100
H	-6.50856500	-1.70713100	0.92610600
H	-5.72836400	-0.64817100	2.11912200
Si	1.30522800	1.40016500	0.25139500
C	1.30501000	2.22143800	1.92678700
H	1.12645800	1.48154900	2.72012600
H	2.27686900	2.70492600	2.11208500
H	0.52113500	2.99213100	1.98490000
C	1.57807000	2.61093200	-1.14787700
H	0.77216400	3.36060500	-1.16245500
H	2.53182700	3.14374600	-1.00437600
H	1.60695700	2.10706400	-2.12509500
C	2.52136200	-0.03201200	0.16584600
C	3.69138800	0.06685400	-0.60527400
C	2.29858900	-1.21827100	0.88662400
C	4.61442300	-0.98034100	-0.64831600
H	3.89447100	0.97230600	-1.18391800
C	3.21662600	-2.26710300	0.84285600
H	1.38324300	-1.32610600	1.47427800
C	4.37776100	-2.14851800	0.07609900
H	5.51896600	-0.88530500	-1.25237100
H	3.02424400	-3.18272600	1.40569800
H	5.09676700	-2.96945600	0.04079300

B-ts

C	-0.65470100	-0.00090900	-1.04694200

H	-0.37853400	1.04031600	-0.85687600
H	-0.11799800	-0.32996900	-1.94225100
C	-0.22389300	-0.83598900	0.13324400
H	-0.39460500	-0.38323500	1.12332000
C	-2.16436600	-0.10709700	-1.29686900
H	-2.41055100	-1.14784700	-1.52872700
H	-2.41307600	0.48116300	-2.18483200
C	-3.01273700	0.36577400	-0.12227700
H	-2.70121100	1.37593400	0.16709500
O	-0.30693500	-2.15679700	0.06493600
H	-2.82902000	-0.27556700	0.74621900
C	-4.50487700	0.36696500	-0.43080600
H	-4.69972600	1.03273300	-1.27913300
H	-4.81066600	-0.63630500	-0.74852100
C	-5.36016700	0.79984100	0.75401600
H	-5.17750300	0.12207400	1.59370700
H	-5.04049200	1.79365300	1.08282200
C	-6.84763600	0.82211500	0.42934600
H	-7.44311400	1.12013800	1.29420700
H	-7.05991100	1.52424800	-0.38050400
H	-7.19219800	-0.16483100	0.11118700
Si	1.68761000	-1.41765100	0.22574500
C	2.00421100	-2.17909900	1.88845400
H	1.70425800	-3.22788100	1.89127900
H	3.06637300	-2.12141700	2.13831000
H	1.44501700	-1.66486700	2.67334800
C	2.22863900	-2.42636200	-1.23511900
H	1.88233900	-3.45568000	-1.13694900
H	1.82984900	-2.02337600	-2.16878600
H	3.31922400	-2.42968100	-1.30672600
C	2.54268300	0.26519400	0.13516500
C	2.62719600	1.08157800	1.26830200
C	3.05405300	0.75765400	-1.06958200
C	3.21135200	2.34010400	1.20487200
H	2.23661800	0.73005700	2.21831000
C	3.63853800	2.01709800	-1.13947400
H	3.00410700	0.15134300	-1.96812800
C	3.71750000	2.81046300	-0.00176200
H	3.27092200	2.95468900	2.09507000
H	4.03218300	2.37949400	-2.08158700
H	4.17188900	3.79231800	-0.05516700

C-ts

C	-0.67968100	-1.14982400	0.33413100

H	0.21630400	-1.72014300	0.59022400
H	-1.04970700	-0.67590900	1.24809500
C	-0.35573000	-0.07867500	-0.70572900
H	0.06367600	-0.57210600	-1.59992800
C	-1.76103300	-2.07879300	-0.24561500
H	-2.05521900	-2.80695500	0.52009900
H	-1.33129500	-2.63573400	-1.08080900
C	-2.96901800	-1.29781100	-0.70297000
H	-3.43886200	-1.67644800	-1.61217100
O	-1.48964500	0.66278300	-1.02787100
H	-2.39810700	-0.19769000	-1.06346700
C	-3.96289100	-0.88596600	0.35434400
H	-4.45059000	-1.78754000	0.74998900
H	-3.43603500	-0.42845500	1.19898300
C	-5.02836500	0.07285300	-0.16411100
H	-4.54092300	0.97429100	-0.54768000
H	-5.54013400	-0.38990900	-1.01343900
C	-6.04341700	0.45319400	0.90444100
H	-6.79499100	1.14181800	0.51453800
H	-6.56240700	-0.43101700	1.28202200
H	-5.55361600	0.93870700	1.75186900
Si	0.95184900	1.17442900	-0.08118200
C	1.13073700	2.52170100	-1.35921800
H	0.19117300	3.06705500	-1.47162700
H	1.90749200	3.23297100	-1.06846500
H	1.39875100	2.10626100	-2.33363900
C	0.41976300	1.85852100	1.56961200
H	-0.58274400	2.28598100	1.48756900
H	0.39471500	1.08640500	2.34139900
H	1.09485500	2.65037000	1.90330500
C	2.56481000	0.22317400	0.08378500
C	3.17257300	-0.32583400	-1.05201500
C	3.18513700	0.01777400	1.31965500
C	4.35228400	-1.05219500	-0.95838500
H	2.71974200	-0.18628600	-2.02913300
C	4.36874500	-0.70551100	1.42036700
H	2.74373300	0.42874700	2.22160100
C	4.95317600	-1.24297600	0.28101800
H	4.80421000	-1.46919300	-1.85032600
H	4.83421100	-0.84961500	2.38812000
H	5.87451400	-1.80767100	0.35652800

D

C	-0.71829400	-1.29012700	0.09550100

H	-0.88884700	-2.35048200	-0.10913600
H	0.08666100	-1.23559500	0.83936000
C	-0.27779500	-0.63700900	-1.16657400
H	-0.67135900	-0.94407800	-2.12829100
C	-1.99473500	-0.67877100	0.70109900
H	-1.83045600	0.38867200	0.87765600
H	-2.17084300	-1.13544200	1.67987600
C	-3.22705000	-0.86395800	-0.17509600
H	-3.37120400	-1.93186400	-0.37507500
O	0.12441300	0.66466500	-1.13871000
H	-3.06097100	-0.38638300	-1.14651200
C	-4.49264100	-0.29299400	0.45231100
H	-4.66127500	-0.76873500	1.42502200
H	-4.34739100	0.77467100	0.65205200
C	-5.73156000	-0.47659800	-0.41598700
H	-5.56628300	0.00417200	-1.38515200
H	-5.87277800	-1.54277100	-0.61922900
C	-6.98892500	0.09115600	0.22837500
H	-7.86550200	-0.05160200	-0.40612200
H	-7.18790900	-0.39373500	1.18715000
H	-6.88179200	1.16245900	0.41484500
Si	1.24876400	1.34116900	-0.10555500
C	1.78312400	2.90254400	-0.95335300
H	0.93742700	3.58052800	-1.09205300
H	2.53883000	3.42132600	-0.35858300
H	2.21296900	2.68665900	-1.93418800
C	0.54733000	1.71991400	1.57480100
H	-0.33037600	2.36454300	1.48398900
H	0.26285200	0.82327400	2.12864100
H	1.29058800	2.26240900	2.16623200
C	2.66736000	0.12465000	0.04297900
C	3.08965600	-0.59709600	-1.07965000
C	3.34508700	-0.08008000	1.24759700
C	4.15062600	-1.48984600	-1.00266000
H	2.57993600	-0.46331300	-2.02909600
C	4.41070200	-0.96968100	1.33105700
H	3.04013300	0.45847700	2.13900000
C	4.81329800	-1.67659400	0.20549200
H	4.45961100	-2.04234100	-1.88187700
H	4.92252500	-1.11429600	2.27479000
H	5.64035900	-2.37331000	0.26879200

E

C	-0.78073000	-0.92731200	0.37099700

H	0.12450300	-1.43332300	0.72284000
H	-1.23232200	-0.44517600	1.24349000
C	-0.38604200	0.14960800	-0.63465900
H	-0.06450500	-0.34456300	-1.56493000
C	-1.73161600	-1.99545600	-0.19989100
H	-1.87824000	-2.75822300	0.57938600
H	-1.22998900	-2.49309000	-1.03314700
C	-3.06516000	-1.48658800	-0.63900400
H	-3.44335400	-1.78498800	-1.61151400
O	-1.46717700	1.04681200	-0.92359300
H	-2.22705000	0.51841100	-1.20919800
C	-4.05036200	-0.97026500	0.35211500
H	-4.53218100	-1.82129100	0.86121000
H	-3.53368900	-0.41488700	1.14301700
C	-5.13964100	-0.09303300	-0.25883400
H	-4.67802800	0.78807800	-0.71547700
H	-5.62905000	-0.64272500	-1.06839500
C	-6.17524300	0.34410400	0.76716900
H	-6.94055200	0.97934500	0.31813000
H	-6.67447600	-0.52117100	1.20948400
H	-5.70636600	0.90704800	1.57789000
Si	1.04382400	1.23905600	-0.02210300
C	1.32251100	2.61272400	-1.25803400
H	0.44873000	3.26619700	-1.30864100
H	2.18839700	3.21988500	-0.98398700
H	1.49616900	2.20626800	-2.25770900
C	0.63206400	1.91736300	1.66779100
H	-0.31419200	2.46275100	1.62770200
H	0.53026200	1.12352100	2.41098800
H	1.40208300	2.61000600	2.01632700
C	2.58293500	0.15848000	0.07182400
C	3.11009100	-0.41236100	-1.09320500
C	3.23622700	-0.10674200	1.27869300
C	4.24190800	-1.21654600	-1.05563000
H	2.63187800	-0.22772200	-2.05065100
C	4.37180700	-0.90887700	1.32433200
H	2.85809300	0.31787700	2.20274100
C	4.87652500	-1.46507700	0.15627800
H	4.63184300	-1.64667400	-1.97040600
H	4.86294800	-1.09768000	2.27145200
H	5.76130800	-2.08927600	0.18793900

4. Further application

(Z)-2-((benzyloxy)imino)-3-hydroxynonanamide(3a-8)

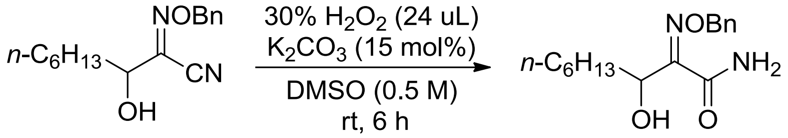

To a stirred solution of **3a** (0.2 mmol, 58.4 mg) in DMSO (1 mL), cooled in an ice bath, are added 30% H_2O_2 (24 uL) and anhydrous K_2CO_3(15 mol%, 4.14mg). The mixture is allowed to warm up to room temperature and the resulting mixture was kept stirring for 6 h. The reaction mixture was quenched with water (10 mL), extracted with ethyl acetate (3×10 mL) and organic phase was combined and washed with brine, dried over anhydrous Na_2SO_4, concentrated under reduced pressure. The crude product was purified with column chromatography on silica gel (200−300 mesh) with PE/EA (1/1, v/v) as eluent to afford 42.0 mg of the title compound as a white solid (72% yield).

R_f = 0.30 (PE/EA = 1/1, v/v), White solid, mp: 59 °C–62 °C. NMR Spectroscopy: ^{1}H NMR (400 MHz, $CDCl_3$, 25 °C) δ 7.40–7.31 (m, 6H), 6.26 (s, 1H), 5.22 (s, 2H), 4.46–4.40 (m, 1H), 3.56 (d, J = 8.2 Hz, 1H), 1.79–1.68 (m, 2H), 1.46–1.38 (m, 1H), 1.33–1.20 (m, 7H), 0.88 (t, J = 6.9 Hz, 3H); ^{13}C NMR (151 MHz, $CDCl_3$, 25 °C) δ 162.2, 151.4, 136.0, 128.8, 128.8, 128.7, 78.3, 72.5, 35.1, 31.9, 29.2, 25.6, 22.7, 14.2. IR (ATR): 3388, 3298, 3183, 2929, 2855, 1684, 1643, 1453, 1394, 1006 cm^{-1}. HRMS (ESI, m/z): calcd for $C_{16}H_{25}N_2O_3^+$ (M+H)$^+$:293.1860 ; Found: 293.1853.

5. Minisci Reaction

Investigation of reaction conditions for catalytic Minisci reaction[a]

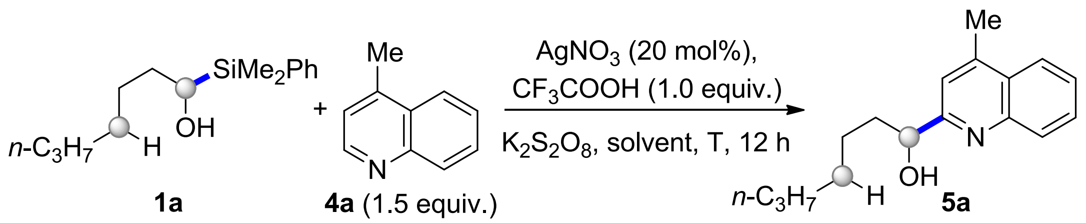

entry	1a/4a	solvent	T/°C	conv. of **1a** (%)	conv. of **4a** (%)	yield of **5a** (%)
1	1/1.5	MeCN:H_2O (1:1)	70	65	80	7
2	1/1.5	MeCN	70	94	70	2
3	1/1.5	acetone	70	92	95	0
4	1/1.5	MeOH	70	66	83	0
5	1/1.5	H_2O	70	90	69	0
6	1/1.5	MeCN:H_2O (2:1)	80	100	62	25
7	1/1.5	MeCN:H_2O (5:1)	80	100	69	40
8	2/1	MeCN:H_2O (5:1)	80	100	76	51
9	1/1	MeCN:H_2O (5:1)	80	100	76	28
10[b]	2/1	MeCN:H_2O (5:1)	80	100	100	55 (53)

[a] Reaction conditions: The mixture of **1a** (0.2 mmol), $AgNO_3$ (0.04 mmol), $K_2S_2O_8$ (0.4 mmol), **4a** (0.3 mmol), CF_3COOH (0.2 mmol) and solvent (2 mL) was stirred at 80 °C under N_2 for 12 h, the conversion of compounds **1a** and **4a** and the yield of **5a** was determined by ^1H NMR with $BrCH_2CH_2Br$ as the internal standard. [b] 2.2 equiv. of $K_2S_2O_8$ was used and the yield in the parenthesis refers to the isolated yield of **5a**.

1-(4-Methylquinolin-2-yl)heptan-1-ol (5a)

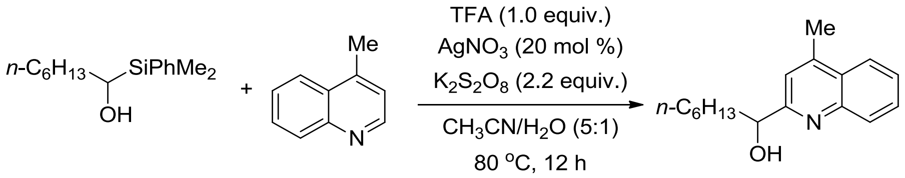

1a **4a** **5a**

Under N_2 atmosphere, $AgNO_3$ (6.8 mg, 0.04 mmol, 20 mol%), CH_3CN/H_2O (1.67 mL / 0.33 mL), **4a** (28.6 mg, 0.20 mmol), TFA (22.8 mg, 0.2 mmol, 1.0 equiv.), **1a** (100 mg, 0.40 mmol, 2.0 equiv.) , $K_2S_2O_8$ (118.8 mg, 0.44 mmol, 2.2 equiv.) was added into a reaction tube. The tube was then sealed, and the resulting mixture was kept stirring at 80 °C in heating block for 12 h. The reaction mixture was quenched with saturated $NaHCO_3$ aqueous solution (10 mL), extracted with ethyl acetate (3×10 mL) and organic phase was combined and washed with brine, dried over anhydrous Na_2SO_4, concentrated under reduced pressure. The crude product was purified with column chromatography on silica gel (200–300 mesh) with PE/EA (10/1, v/v) as eluent to afford 27.0 mg of the title compound as a faint yellow oil (53% yield).

R_f = 0.31 (PE/EA = 4/1, v/v). NMR Spectroscopy: ^1H NMR (400 MHz, $CDCl_3$, 25 °C) δ 8.07–8.05 (m, 1 H), 7.98–7.95 (m, 1 H), 7.62–7.67 (m, 1 H), 7.55–7.52 (m, 1 H), 7.17 (s, 1H), 5.01(s, 1 H), 4.85 (dd, *J*

= 7.7, 3.9 Hz, 1 H), 2.70 (s, 3 H), 1.99–1.63 (m, 2 H), 1.58–1.22 (m, 8 H), 0.91–0.81 (m, 3 H); ^{13}C NMR (101 MHz, $CDCl_3$, 25 °C) δ 162.1, 146.3, 145.2, 129.5, 129.4, 127.6, 126.2, 123.8, 119.0, 72.7, 38.4, 31.9, 29.5, 25.4, 22.7, 19.0, 14.2. IR (ATR): 3380, 2926, 2855, 1602, 1509, 1446, 1249, 1069, 880, 760 cm^{-1}. HRMS (ESI, m/z): calcd for $C_{17}H_{23}NONa^+$ $(M+Na)^+$: 280.1672; Found: 280.1672.

1-(2-Methylquinolin-4-yl)heptan-1-ol (5b)

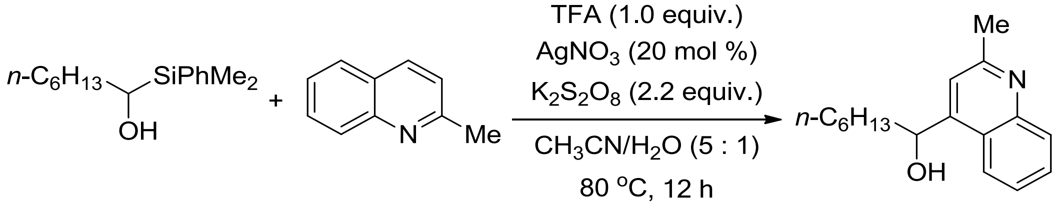

1a **4b** **5b**

Under N_2 atmosphere, $AgNO_3$ (6.8 mg, 0.04 mmol, 20 mol%), CH_3CN/H_2O (1.67 mL / 0.33 mL), **4b** (28.6 mg, 0.20 mmol), TFA (22.8 mg, 0.2 mmol, 1.0 equiv.), **1a** (100 mg, 0.40 mmol, 2.0 equiv.), $K_2S_2O_8$ (118.8 mg, 0.44 mmol, 2.2 equiv.) was added into a reaction tube. The tube was then sealed, and the resulting mixture was kept stirring at 80 °C in heating block for 12 h. The reaction mixture was quenched with saturated $NaHCO_3$ aqueous solution (10 mL), extracted with ethyl acetate (3×10 mL) and organic phase was combined and washed with brine, dried over anhydrous Na_2SO_4, concentrated under reduced pressure. The crude product was purified with column chromatography on silica gel (200–300 mesh) with PE/EA (10/1, v/v) as eluent to afford 32.0 mg of the title compound as a faint yellow oil (62% yield). R_f = 0.15 (PE/EA = 4/1, v/v). NMR Spectroscopy: ^{1}H NMR (400 MHz, $CDCl_3$, 25 °C) δ 7.98–7.96 (m, 1H), 7.89–7.86(m, 1H), 7.62–7.58 (m, 1H), 7.45–7.40 (m, 1H), 7.38 (s, 1H), 5.37 (dd, J = 8.2, 4.1 Hz, 1H), 3.42 (s, 1H), 2.61 (s, 3H), 1.91–1.72 (m, 2H), 1.45–1.18 (m, 8H), 0.85 (t, J = 6.9 Hz, 3H); ^{13}C NMR (101 MHz, $CDCl_3$,25 °C) δ 159.0, 151.0, 147.8, 129.2, 129.1, 125.6, 123.9, 122.9, 118.4, 69.9, 38.6, 31.9, 29.2, 26.2, 25.3, 22.7, 14.2. IR (ATR): 3067, 2922, 2855, 1603, 1457, 1334, 1069, 969, 879, 760 cm^{-1}. HRMS (ESI, m/z): calcd for $C_{17}H_{23}NONa^+$ $(M+Na)^+$: 280.1672; Found: 280.1671.

1-(2-Phenylquinolin-4-yl)heptan-1-ol (5c)

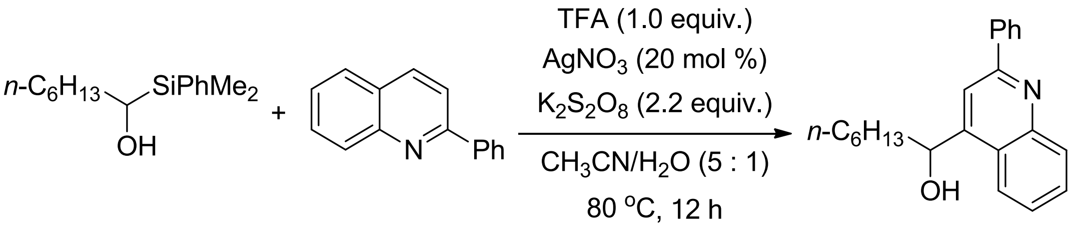

1a **4c** **5c**

Under N_2 atmosphere, $AgNO_3$ (6.8 mg, 0.04 mmol, 20 mol%), CH_3CN/H_2O (1.67 mL / 0.33 mL), **4c** (41.0 mg, 0.20 mmol), TFA (22.8 mg, 0.2 mmol, 1.0 equiv.), **1a** (100 mg, 0.40 mmol, 2.0 equiv.), $K_2S_2O_8$ (118.8 mg, 0.44 mmol, 2.2 equiv.) was added into a reaction tube. The tube was then sealed, and the resulting mixture was kept stirring at 80 °C in heating block for 12 h. The reaction mixture was quenched with saturated $NaHCO_3$ aqueous solution (10 mL), extracted with ethyl acetate (3×10 mL) and organic phase was combined and washed with brine, dried over anhydrous Na_2SO_4, concentrated under reduced pressure. The crude product was purified with column chromatography on silica gel (200–300 mesh) with PE/EA (10/1, v/v) as eluent to afford 41.0 mg of the title compound as a colourless oil (64% yield).

R_f = 0.52 (PE/EA = 4/1, v/v). NMR Spectroscopy: ^1H NMR (400 MHz, $CDCl_3$, 25 °C) δ 8.17–8.15 (m, 1H), 8.00–7.97 (m, 2H), 7.79 (s, 1H), 7.75–7.74 (m, 1H), 7.68–7.64 (m, 1H), 7.45–7.40 (m, 4H), 5.23 (dd, J = 8.2, 4.1 Hz, 1H), 3.21 (s, 1H), 1.87–1.66 (m, 2H), 1.53–1.18 (m, 8H), 0.87 (t, J = 7.0 Hz, 3H); ^{13}C NMR (101 MHz, $CDCl_3$, 25 °C) δ 157.1, 151.6, 148.2, 139.4, 130.3, 129.4, 129.3, 128.8, 127.6, 126.1, 124.5, 122.9, 115.1, 70.1, 38.5, 31.9, 29.2, 26.1, 22.7, 14.2. IR (ATR): 3355, 2926, 2855, 1595, 1494, 1349, 1237, 1066, 909, 768 cm^{-1}. HRMS (ESI, m/z): calcd for $C_{22}H_{25}NONa^+$ $(M+Na)^+$: 342.1828; Found: 342.1828.

1-(2-(4-Fluorophenyl)quinolin-4-yl)heptan-1-ol (5d)

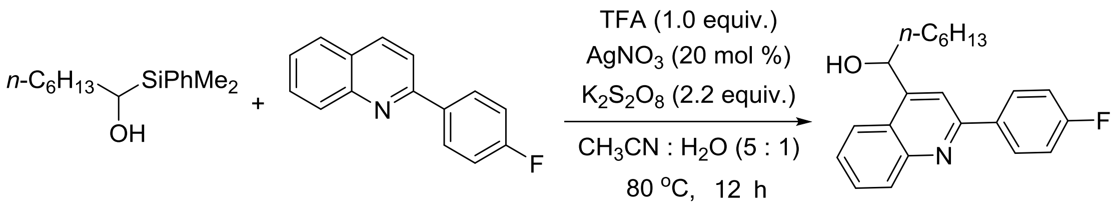

1a **4d** **5d**

Under N_2 atmosphere, $AgNO_3$ (6.8 mg, 0.04 mmol, 20 mol%), CH_3CN/H_2O (1.67 mL / 0.33 mL), **4d** (44.6 mg, 0.20 mmol), TFA (22.8 mg, 0.2 mmol, 1.0 equiv.), **1a** (100.0 mg, 0.40 mmol, 2.0 equiv.), $K_2S_2O_8$ (118.8 mg, 0.44 mmol, 2.2 equiv.) was added into a reaction tube. The tube was then sealed, and the resulting mixture was kept stirring at 80 °C in heating block for 12 h. The reaction mixture was quenched with saturated $NaHCO_3$ aqueous solution (10 mL), extracted with ethyl acetate (3×10 mL) and organic phase was combined and washed with brine, dried over anhydrous Na_2SO_4, concentrated under reduced pressure. The crude product was purified with column chromatography on silica gel (200–300 mesh) with PE/EA (10/1, v/v) as eluent to afford 53.4 mg of the title compound as a colourless oil (79% yield).

R_f = 0.67 (PE/EA = 4/1, v/v). NMR Spectroscopy: ^1H NMR (400 MHz, $CDCl_3$, 25 °C) δ 8.14 (d, J = 8.5 Hz, 1H), 8.00–7.94 (m, 2H), 7.78–7.72 (m, 2H), 7.69–7.65 (m, 1H), 7.46–7.42 (m, 1H), 7.13–7.07 (m, 2H), 5.25 (dd, J = 8.3, 4.0 Hz, 1H), 3.11 (s, 1H), 1.87–1.68 (m, 2H), 1.57–1.38 (m, 2H), 1.36–1.21 (m, 6H), 0.87 (t, J = 6.7 Hz, 3H); ^{13}C NMR (101 MHz, $CDCl_3$, 25 °C) δ 163.9 (d, J = 249.2 Hz), 156.0, 151.7, 148.1, 135.5 (d, J = 2.0 Hz), 130.2, 129.5 (d, J = 3.3 Hz), 129.4, 126.2, 124.4, 122.8, 115.7 (d, J = 21.5 Hz), 114.7, 70.1, 38.5, 31.8, 29.2, 26.1, 22.7, 14.2. ^{19}F NMR (375 MHz, $CDCl_3$) δ –112.5 (m, 1F). IR (ATR): 3068, 2926, 2855, 1599, 1506, 1234, 1155, 1070, 839, 760 cm^{-1}. HRMS (ESI, m/z): calcd for $C_{22}H_{25}FNO^+$ $(M+H)^+$: 338.1915; Found: 338.1915.

1-(2-(4-Chlorophenyl)quinolin-4-yl)heptan-1-ol (5e)

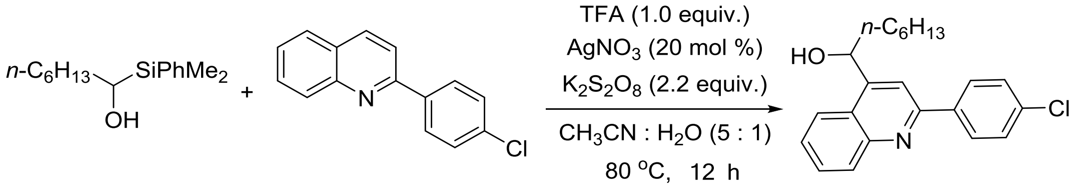

1a **4e** **5e**

Under N_2 atmosphere, $AgNO_3$ (6.8 mg, 0.04 mmol, 20 mol%), CH_3CN/H_2O (1.67 mL / 0.33 mL), **4e** (47.8 mg, 0.20 mmol), TFA (22.8 mg, 0.2 mmol, 1.0 equiv.), **1a** (100.0 mg, 0.40 mmol, 2.0 equiv.), $K_2S_2O_8$ (118.8 mg, 0.44 mmol, 2.2 equiv.) was added into a reaction tube. The tube was then sealed, and

the resulting mixture was kept stirring at 80 °C in heating block for 12 h. The reaction mixture was quenched with saturated $NaHCO_3$ aqueous solution (10 mL), extracted with ethyl acetate (3×10 mL) and organic phase was combined and washed with brine, dried over anhydrous Na_2SO_4, concentrated under reduced pressure. The crude product was purified with column chromatography on silica gel (200–300 mesh) with PE/EA (10/1, v/v) as eluent to afford 56.0 mg of the title compound as a colourless oil (79% yield).

R_f = 0.61 (PE/EA = 4/1, v/v). NMR Spectroscopy: ^{1}H NMR (400 MHz, $CDCl_3$, 25 °C) δ 8.14 (d, J = 8.3 Hz, 1H), 7.92 (d, J = 8.2 Hz, 2H), 7.78–7.76 (m, 2H), 7.70–7.67 (m, 1H), 7.48–7.44 (m, 1H), 7.38 (d, J = 8.1 Hz, 2H), 5.26 (dd, J = 8.3, 3.9 Hz, 1H), 3.06 (s, 1H), 1.86–1.69 (m, 2H), 1.56–1.40 (m, 2H), 1.32 –1.23 (m, 6H), 0.87 (t, J = 6.6 Hz, 3H); ^{13}C NMR (101 MHz, $CDCl_3$, 25 °C) δ 155.9, 151.9, 148.3, 137.9, 135.8, 130.4, 129.7, 129.1, 128.9, 126.5, 124.6, 123.0, 114.8, 70.2, 38.6, 32.0, 29.3, 26.2, 22.8, 14.3. IR (ATR): 3068, 2922, 2855, 1595, 1490, 1423, 1349, 1092, 834, 760 cm^{-1}. HRMS (ESI, m/z): calcd for $C_{22}H_{25}ClNO^+$ $(M+H)^+$: 354.1619; Found: 354.1619.

1-(2-(4-Bromophenyl)quinolin-4-yl)heptan-1-ol (5f)

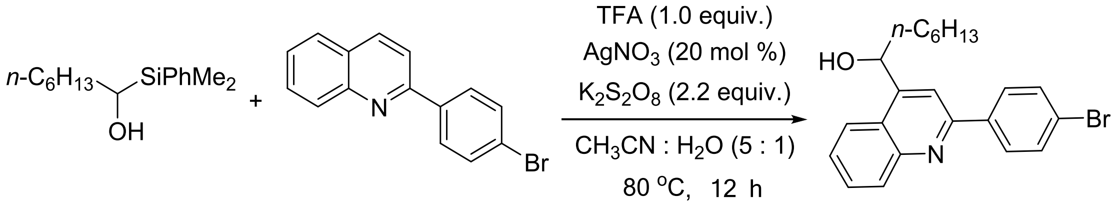

1a **4f** **5f**

Under N_2 atmosphere, $AgNO_3$ (6.8 mg, 0.04 mmol, 20 mol%), CH_3CN/H_2O (1.67 mL / 0.33 mL), **4f** (56.6 mg, 0.20 mmol), TFA (22.8 mg, 0.2 mmol, 1.0 equiv.), **1a** (100.0 mg, 0.40 mmol, 2.0 equiv.), $K_2S_2O_8$ (118.8 mg, 0.44 mmol, 2.2 equiv.) was added into a reaction tube. The tube was then sealed, and the resulting mixture was kept stirring at 80 °C in heating block for 12 h. The reaction mixture was quenched with saturated $NaHCO_3$ aqueous solution (10 mL), extracted with ethyl acetate (3×10 mL) and organic phase was combined and washed with brine, dried over anhydrous Na_2SO_4, concentrated under reduced pressure. The crude product was purified with column chromatography on silica gel (200–300 mesh) with PE/EA (10/1, v/v) as eluent to afford 58.0 mg of the title compound as a colourless oil (73% yield).

R_f = 0.52 (PE/EA = 4/1, v/v). NMR Spectroscopy: ^{1}H NMR (400 MHz, $CDCl_3$, 25 °C) δ 8.14 (d, J = 7.2 Hz, 1H), 7.86 (d, J = 8.4 Hz, 2H), 7.78–7.77 (m, 2H), 7.70–7.67 (m, 1H), 7.54 (d, J = 8.4 Hz, 2H), 7.47 (m, 1H), 5.27 (dd, J = 8.5, 3.9 Hz, 1H), 2.97 (s, 1H), 1.85–1.79 (m, 1H), 1.77–1.71 (m, 1H), 1.56–1.49 (m, 1H), 1.48–1.41 (m, 1H), 1.36–1.23 (m, 6H), 0.87 (t, J = 6.9 Hz, 3H); ^{13}C NMR (101 MHz, $CDCl_3$, 25 °C) δ 155.8, 151.9, 148.2, 138.2, 132.0, 130.3, 129.6, 129.1, 126.4, 124.5, 124.1, 122.9, 114.6, 70.1, 38.5, 31.9, 29.2, 26.2, 22.7, 14.2. IR (ATR): 3068, 2922, 2855, 1595, 1550, 1490, 1349, 1091, 1073, 1010, 910, 831 cm^{-1}. HRMS (ESI, m/z): calcd for $C_{22}H_{24}BrNOH^+$ $(M+H)^+$: 398.1114; Found: 398.1114

4-(4-(1-Hydroxyheptyl)quinolin-2-yl)benzonitrile (5g)

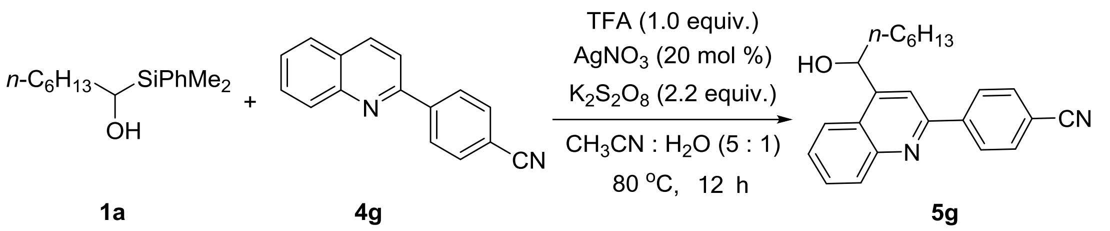

Under N_2 atmosphere, $AgNO_3$ (6.8 mg, 0.04 mmol, 20 mol%), CH_3CN/H_2O (1.67 mL / 0.33 mL), **4g** (46.0 mg, 0.20 mmol), TFA (22.8 mg, 0.2 mmol, 1.0 equiv.), **1a** (100.0 mg, 0.40 mmol, 2.0 equiv.), $K_2S_2O_8$ (118.8 mg, 0.44 mmol, 2.2 equiv.) was added into a reaction tube. The tube was then sealed, and the resulting mixture was kept stirring at 80 °C in heating block for 12 h. The reaction mixture was quenched with saturated $NaHCO_3$ aqueous solution (10 mL), extracted with ethyl acetate (3×10 mL) and organic phase was combined and washed with brine, dried over anhydrous Na_2SO_4, concentrated under reduced pressure. The crude product was purified with column chromatography on silica gel (200–300 mesh) with PE/EA (10/1, v/v) as eluent to afford 48.0 mg of the title compound as a yellow solid (71% yield).

R_f = 0.44 (PE/EA = 4/1, v/v). mp: 83 °C–85 °C. NMR Spectroscopy: ^{1}H NMR (400 MHz, $CDCl_3$, 25 °C) δ 8.20–8.13 (m, 3H), 7.93 (s, 1H), 7.87 (d, J = 8.6 Hz, 1H), 7.76–7.68 (m, 3H), 7.54 (m, 1H), 5.39 (dd, J = 8.5, 3.7 Hz, 1H), 2.86 (s, 1H), 1.87 (m, 1H), 1.81–1.75 (m, 1H), 1.61–1.43 (m, 2H), 1.37–1.22 (m, 6H), 0.92–0.82 (m, 3H); ^{13}C NMR (101 MHz, $CDCl_3$, 25 °C) δ 154.8, 152.2, 148.3, 143.7, 132.6, 130.7, 129.9, 128.1, 127.1, 124.8, 122.9, 118.9, 114.9, 112.7, 70.1, 38.7, 31.8, 29.2, 26.1, 22.7, 14.17. IR (ATR): 3068, 2922, 2855, 2228, 1595, 1234, 1502, 1069, 909, 842 cm^{-1}. HRMS (ESI, m/z): calcd for $C_{23}H_{25}N_2O^+$ $(M+H)^+$: 345.1967; Found: 345.1961.

1-(2-(4-Methoxyphenyl)quinolin-4-yl)heptan-1-ol (5h)

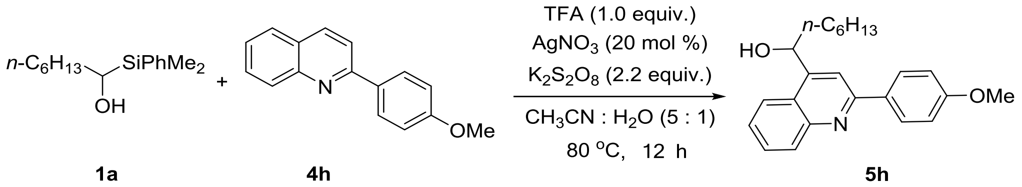

Under N_2 atmosphere, $AgNO_3$ (6.8 mg, 0.04 mmol, 20 mol%), CH_3CN/H_2O (1.67 mL / 0.33 mL), **4h** (47.0 mg, 0.20 mmol), TFA (22.8 mg, 0.2 mmol, 1.0 equiv.), **1a** (100.0 mg, 0.40 mmol, 2.0 equiv.), $K_2S_2O_8$ (118.8 mg, 0.44 mmol, 2.2 equiv.) was added into a reaction tube. The tube was then sealed, and the resulting mixture was kept stirring at 80 °C in heating block for 12 h. The reaction mixture was quenched with saturated $NaHCO_3$ aqueous solution (10 mL), extracted with ethyl acetate (3×10 mL) and organic phase was combined and washed with brine, dried over anhydrous Na_2SO_4, concentrated under reduced pressure. The crude product was purified with column chromatography on silica gel (200–300 mesh) with PE/EA (10/1, v/v) as eluent to afford 50.0 mg of the title compound as a colourless oil (72% yield).

R_f = 0.28 (PE/EA = 4/1, v/v). NMR Spectroscopy: ^{1}H NMR (400 MHz, $CDCl_3$, 25 °C) δ 8.12 (d, J = 8.5, 1H), 8.00–7.92 (m, 2H), 7.74–7.70 (m, 2H), 7.66–7.62 (m, 1H), 7.40–7.38 (m, 1H), 6.97–6.91 (m, 2H), 5.21 (dd, J = 8.2, 4.1 Hz, 1H), 3.86 (s, 3H), 3.15 (s, 1H), 1.87–1.66 (m, 2H), 1.56–1.38 (m, 2H), 1.34–1.21 (m, 6H), 0.88–0.84 (m, 3H); ^{13}C NMR (101 MHz, $CDCl_3$, 25 °C) δ 160.9, 156.6, 151.3, 148.2, 131.9, 130.0, 129.2, 128.9, 125.7, 124.2, 122.9, 114.6, 114.2, 70.1, 55.4, 38.4, 31.9, 29.2, 26.1, 22.7, 14.2. IR (ATR): 3071, 3004, 2926, 2855, 1599, 1505, 1252, 1174, 1032, 834 cm^{-1}. HRMS (ESI, m/z): calcd for

$C_{23}H_{28}NO_2^+$ (M+H)$^+$: 350.2115; Found: 350.2114.

1-(2-(3-Methoxyphenyl)quinolin-4-yl)heptan-1-ol (5i)

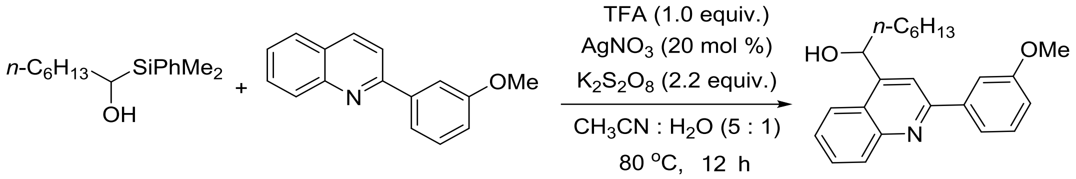

Under N_2 atmosphere, $AgNO_3$ (6.8 mg, 0.04 mmol, 20 mol%), CH_3CN/H_2O (1.67 mL / 0.33 mL), **4i** (47.0 mg, 0.20 mmol), TFA (22.8 mg, 0.2 mmol, 1.0 equiv.), **1a** (100.0 mg, 0.40 mmol, 2.0 equiv.), $K_2S_2O_8$ (118.8 mg, 0.44 mmol, 2.2 equiv.) was added into a reaction tube. The tube was then sealed, and the resulting mixture was kept stirring at 80 °C in heating block for 12 h. The reaction mixture was quenched with saturated $NaHCO_3$ aqueous solution (10 mL), extracted with ethyl acetate (3×10 mL) and organic phase was combined and washed with brine, dried over anhydrous Na_2SO_4, concentrated under reduced pressure. The crude product was purified with column chromatography on silica gel (200–300 mesh) with PE/EA (10/1, v/v) as eluent to afford 49.0 mg of the title compound as a colourless oil (72% yield).

R_f = 0.36 (PE/EA = 4/1, v/v). NMR Spectroscopy: ^1H NMR (400 MHz, $CDCl_3$, 25 °C) δ 8.17 (d, J = 8.5 Hz, 1H), 7.85 (d, J = 8.3 Hz, 2H), 7.71–7.65 (m, 2H), 7.61–7.58 (m, 1H), 7.48–7.44 (m, 1H), 7.38–7.34 (m, 1H), 6.99–6.96 (m, 1H), 5.33 (dd, J = 8.2, 4.0 Hz, 1H), 3.90 (s, 3H), 2.83 (s, 1H), 1.90–1.73 (m, 2H), 1.57–1.42 (m, 2H), 1.35–1.22 (m, 6H), 0.86 (t, J = 6.8 Hz, 3H); ^{13}C NMR (101 MHz, $CDCl_3$, 25 °C) δ 160.1, 157.0, 151.4, 148.3, 141.0, 130.5, 129.8, 129.3, 126.2, 124.6, 122.9, 120.1, 115.5, 115.3, 112.7, 70.3, 55.5, 38.5, 31.9, 29.2, 26.1, 22.7, 14.2. IR (ATR): 3071, 2922, 2855, 1595, 1461, 1253, 1047, 876, 760 cm^{-1}. HRMS (ESI, m/z): calcd for $C_{23}H_{28}NO_2^+$ (M+H)$^+$: 350.2115; Found: 350.2114.

1-(2-(2-Methoxyphenyl)quinolin-4-yl)heptan-1-ol (5j)

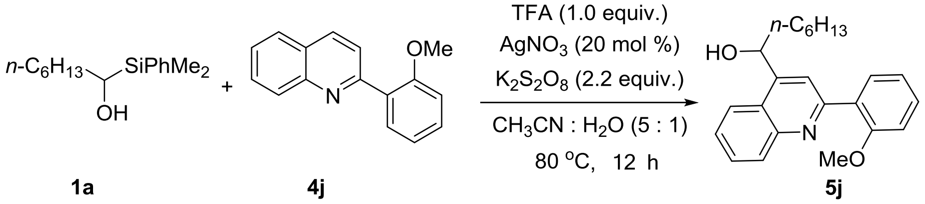

Under N_2 atmosphere, $AgNO_3$ (6.8 mg, 0.04 mmol, 20 mol%), CH_3CN/H_2O (1.67 mL / 0.33 mL), **4j** (47.0 mg, 0.20 mmol), TFA (22.8 mg, 0.2 mmol, 1.0 equiv.), **1a** (100.0 mg, 0.40 mmol, 2.0 equiv.), $K_2S_2O_8$ (118.8 mg, 0.44 mmol, 2.2 equiv.) was added into a reaction tube. The tube was then sealed, and the resulting mixture was kept stirring at 80 °C in heating block for 12 h. The reaction mixture was quenched with saturated $NaHCO_3$ aqueous solution (10 mL), extracted with ethyl acetate (3×10 mL) and organic phase was combined and washed with brine, dried over anhydrous Na_2SO_4, concentrated under reduced pressure. The crude product was purified with column chromatography on silica gel (200–300 mesh) with PE/EA (10/1, v/v) as eluent to afford 40.0 mg of the title compound as a colourless oil (58% yield).

R_f = 0.29 (PE/EA = 4/1, v/v). NMR Spectroscopy: ^1H NMR (400 MHz, $CDCl_3$, 25 °C) 8.19 (d, J = 8.5 Hz, 1H), 7.99 (d, J = 8.6 Hz, 2H), 7.76–7.74 (m, 1H), 7.69–7.65 (m, 1H), 7.54–7.49 (m, 1H), 7.40–7.36

(m, 1H), 7.09–7.05 (m, 1H), 6.69–6.97 (m, 1H), 5.41 (dd, J = 7.9, 4.3 Hz, 1H), 3.80 (s, 3H), 1.99–1.80 (m, 2H), 1.54–1.43 (m, 2H), 1.31–1.26 (m, 6H), 0.87 (t, J = 6.6 Hz, 3H); ^{13}C NMR (101 MHz, $CDCl_3$, 25 °C) δ 157.2, 149.4, 148.4, 131.4, 130.4, 130.3, 129.7, 129.0, 126.2, 124.5, 123.0, 121.2, 120.0, 115.8, 111.5, 70.4, 55.7, 38.29, 31.9, 29.3, 26.0, 22.7, 14.2. IR (ATR): 3071, 2926, 2855, 1599, 1494, 1245, 1162, 1077, 1029 cm^{-1}. HRMS (ESI, m/z): calcd for $C_{23}H_{28}NO_2^+$ (M+H)$^+$: 350.2115; Found: 350.2114.

1-(2-(3,5-Dimethylphenyl)quinolin-4-yl)heptan-1-ol (5k)

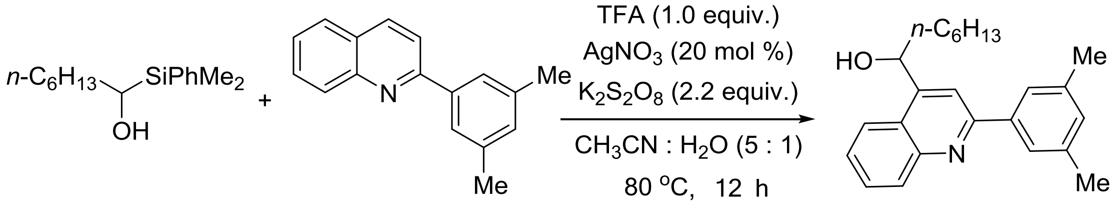

1a **4k** **5k**

Under N_2 atmosphere, $AgNO_3$ (6.8 mg, 0.04 mmol, 20 mol%), CH_3CN/H_2O (1.67 mL / 0.33 mL), **4k** (46.6 mg, 0.20 mmol), TFA (22.8 mg, 0.2 mmol, 1.0 equiv.), **1a** (100.0 mg, 0.40 mmol, 2.0 equiv.), $K_2S_2O_8$ (118.8 mg, 0.44 mmol, 2.2 equiv.) was added into a reaction tube. The tube was then sealed, and the resulting mixture was kept stirring at 80 °C in heating block for 12 h. The reaction mixture was quenched with saturated $NaHCO_3$ aqueous solution (10 mL), extracted with ethyl acetate (3×10 mL) and organic phase was combined and washed with brine, dried over anhydrous Na_2SO_4, concentrated under reduced pressure. The crude product was purified with column chromatography on silica gel (200–300 mesh) with PE/EA (10/1, v/v) as eluent to afford 50.0 mg of the title compound as a colourless oil (72% yield).

R_f = 0.61 (PE/EA = 4/1, v/v). NMR Spectroscopy: ^{1}H NMR (400 MHz, $CDCl_3$, 25 °C) δ 8.18 (d, J = 8.5 Hz, 1H), 7.90–7.83 (m, 2H), 7.72–7.65 (m, 3H), 7.49–7.44 (m, 1H), 7.08 (s, 1H), 5.36 (dd, J = 8.4, 4.0 Hz, 1H), 2.61 (s, 1H), 2.41 (s, 6H), 1.92–1.75 (m, 2H), 1.59–1.42 (m, 2H), 1.36–1.24 (m, 6H), 0.89–0.85 (m, 3H); ^{13}C NMR (101 MHz, $CDCl_3$, 25 °C) δ 157.5, 151.1, 148.3, 139.5, 138.4, 131.2, 130.5, 129.2, 126.0, 125.4, 124.5, 122.8, 115.3, 70.4, 38.5, 31.9, 29.2, 26.1, 22.7, 21.5, 14.2. IR (ATR): 3064, 2922, 2855, 1595, 1554, 1353, 1069, 891, 850 cm^{-1}. HRMS (ESI, m/z): calcd for $C_{24}H_{30}NO^+$ (M+H)$^+$: 348.2322; Found: 348.2322.

1-(Isoquinolin-1-yl)heptan-1-ol (5l)

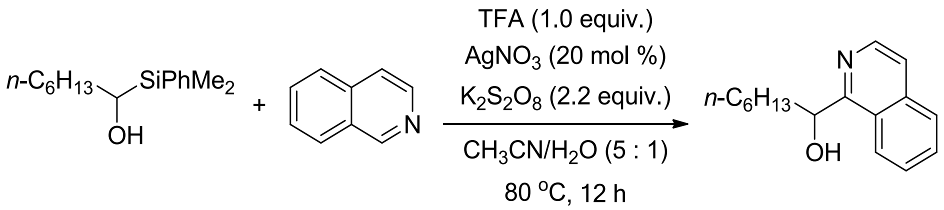

1a **4l** **5l**

Under N_2 atmosphere, $AgNO_3$ (6.8 mg, 0.04 mmol, 20 mol%), CH_3CN/H_2O (1.67 mL / 0.33 mL), **4l** (25.8 mg, 0.20 mmol), TFA (22.8 mg, 0.2 mmol, 1.0 equiv.), **1a** (100 mg, 0.40 mmol, 2.0 equiv.), $K_2S_2O_8$ (118.8 mg, 0.44 mmol, 2.2 equiv.) was added into a reaction tube. The tube was then sealed, and the resulting mixture was kept stirring at 80 °C in heating block for 12 h. The reaction mixture was quenched with saturated $NaHCO_3$ aqueous solution (10 mL), extracted with ethyl acetate (3×10 mL) and organic

phase was combined and washed with brine, dried over anhydrous Na_2SO_4, concentrated under reduced pressure. The crude product was purified with column chromatography on silica gel (200–300 mesh) with PE/EA (10/1, v/v) as eluent to afford 26.0 mg of the title compound as a faint yellow oil (53% yield). R_f = 0.64 (PE/EA = 4/1, v/v). NMR Spectroscopy: ^{1}H NMR (400 MHz, $CDCl_3$, 25 °C) δ 8.45 (d, J = 5.7 Hz, 1H), 8.04 (dd, J = 8.4, 0.9 Hz, 1H), 7.87 (d, J = 8.2 Hz, 1H), 7.73–7.65 (m, 1H), 7.64–7.60 (m, 1H), 7.59 (d, J = 5.7 Hz, 1H), 5.52–5.35 (m, 1H), 5.10 (s, 1H), 1.74–1.53 (m, 2H), 1.53–1.21 (m, 8H), 0.92–0.75 (m, 3H); ^{13}C NMR (101 MHz, $CDCl_3$, 25 °C) δ 161.8, 140.5, 136.6, 130.4, 127.7, 127.4, 124.9, 124.3, 120.5, 69.8, 39.5, 31.9, 29.4, 25.8, 22.8, 14.2. IR (ATR): 3388, 2922, 2855, 1587, 1502, 1375, 1334, 1073, 1006, 746 cm^{-1}. HRMS (ESI, m/z): calcd for $C_{16}H_{21}NONa^+$ $(M+Na)^+$: 266.1515; Found: 266.1515.

1-(5-Bromoisoquinolin-1-yl)heptan-1-ol (5m)

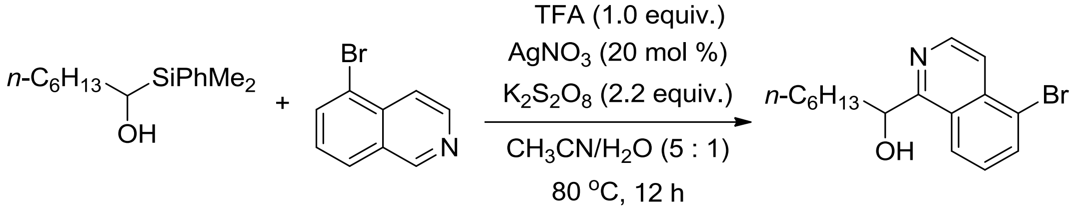

1a **4m** **5m**

Under N_2 atmosphere, $AgNO_3$ (6.8 mg, 0.04 mmol, 20 mol%), CH_3CN/H_2O (1.67 mL / 0.33 mL), **4m** (41.6 mg, 0.20 mmol), TFA (22.8 mg, 0.2 mmol, 1.0 equiv.), **1a** (100 mg, 0.40 mmol, 2.0 equiv.), $K_2S_2O_8$ (118.8 mg, 0.44 mmol, 2.2 equiv.) was added into a reaction tube. The tube was then sealed, and the resulting mixture was kept stirring at 80 °C in heating block for 12 h. The reaction mixture was quenched with saturated $NaHCO_3$ aqueous solution (10 mL), extracted with ethyl acetate (3×10 mL) and organic phase was combined and washed with brine, dried over anhydrous Na_2SO_4, concentrated under reduced pressure. The crude product was purified with column chromatography on silica gel (200–300 mesh) with PE/EA (20/1, v/v) as eluent to afford 45.0 mg of the title compound as a colourless oil (70% yield). R_f = 0.46 (PE/EA = 9/1, v/v). NMR Spectroscopy: ^{1}H NMR (400 MHz, $CDCl_3$, 25 °C) ^{1}H NMR (400 MHz,) δ 8.54 (d, J = 6.0 Hz, 1H), 8.03-8.00 (m, 1H), 7.98 (dd, J = 7.5, 1.0 Hz, 1H), 7.97–7.95 (m, 1H), 7.47 (dd, J = 8.4, 7.5 Hz, 1H), 5.45 (d, J = 6.2 Hz, 1H), 4.99 (s, 1H), 1.97–1.90 (m, 1H), 1.68–1.53 (m, 2H), 1.48–1.41 (m, 1H), 1.39–1.24 (m, 6H), 0.88–0.83 (m, 3H).^{13}C NMR (101 MHz, $CDCl_3$, 25 °C) δ 162.2, 141.9, 135.7, 134.1, 127.7, 126.1, 123.9, 122.7, 119.4, 69.9, 39.6, 31.9, 29.4, 25.7, 22.7, 14.2. IR (ATR): 3399, 2926, 2855, 1580, 1487, 1368, 1327, 1244, 1073, 902 cm^{-1}. HRMS (ESI, m/z): calcd for $C_{16}H_{20}BrNONa^+$ $(M+Na)^+$: 344.0626; Found: 344.0620.

1-(5-Methoxyisoquinolin-1-yl)heptan-1-ol (5n)

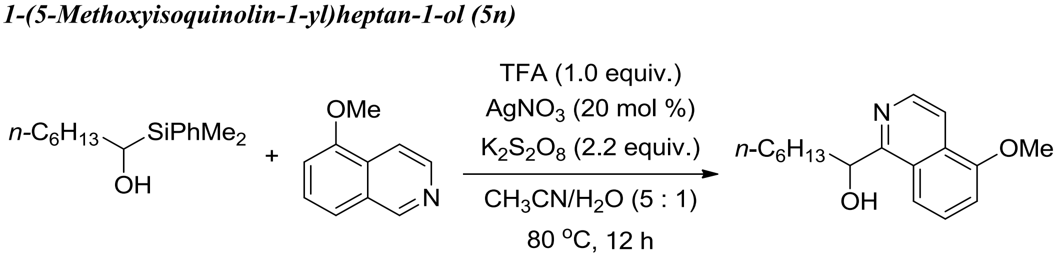

1a **4n** **5n**

Under N_2 atmosphere, $AgNO_3$ (6.8 mg, 0.04 mmol, 20 mol%), CH_3CN/H_2O (1.67 mL / 0.33 mL), **4n**

(31.8 mg, 0.20 mmol), TFA (22.8 mg, 0.2 mmol, 1.0 equiv.), **1a** (100 mg, 0.40 mmol, 2.0 equiv.), $K_2S_2O_8$ (118.8 mg, 0.44 mmol, 2.2 equiv.) was added into a reaction tube. The tube was then sealed, and the resulting mixture was kept stirring at 80 °C in heating block for 12 h. The reaction mixture was quenched with saturated $NaHCO_3$ aqueous solution (10 mL), extracted with ethyl acetate (3×10 mL) and organic phase was combined and washed with brine, dried over anhydrous Na_2SO_4, concentrated under reduced pressure. The crude product was purified with column chromatography on silica gel (200–300 mesh) with PE/EA (10/1, v/v) as eluent to afford 29.0 mg of the title compound as a colourless oil (53% yield). R_f = 0.50 (PE/EA = 4/1, v/v). NMR Spectroscopy: ^1H NMR (400 MHz, $CDCl_3$, 25 °C) δ 8.44 (d, *J* = 5.8 Hz, 1H), 7.98 (d, *J* = 5.8 Hz, 1H), 7.54 (m, 2H), 7.01 (m, 1H), 5.47–5.36 (m, 1H), 5.11 (s, 1H), 4.02 (s, 3H), 1.67–1.58 (m, 3H), 1.30–1.25 (m, 7H), 0.84 (t, J = 6.9 Hz, 3H); 13C NMR (101 MHz, $CDCl_3$, 25 °C) *δ* 161.0, 155.2, 140.1, 129.2, 127.5, 125.9, 116.0, 114.8, 107.6, 69.9, 55.8, 39.4, 32.0, 29.4, 25.7, 22.8, 14.2. IR (ATR): 3388, 2930, 2855, 1587, 1499, 1387, 1264, 1029, 838, 746 cm^{-1}. HRMS (ESI, m/z): calcd for $C_{17}H_{23}NO_2Na^+$ $(M+Na)^+$: 296.1621; Found: 296.1621.

1-(5-(4-Methoxyphenyl)isoquinolin-1-yl)heptan-1-ol (5o)

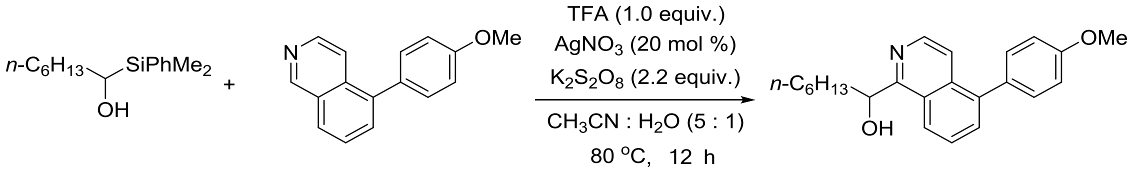

1a **4o** **5o**

Under N_2 atmosphere, $AgNO_3$ (6.8 mg, 0.04 mmol, 20 mol%), CH_3CN/H_2O (1.67 mL / 0.33 mL), **4o** (47.0 mg, 0.20 mmol), TFA (22.8 mg, 0.2 mmol, 1.0 equiv.), **1a** (100.0 mg, 0.40 mmol, 2.0 equiv.), $K_2S_2O_8$ (118.8 mg, 0.44 mmol, 2.2 equiv.) was added into a reaction tube. The tube was then sealed, and the resulting mixture was kept stirring at 80 °C in heating block for 12 h. The reaction mixture was quenched with saturated $NaHCO_3$ aqueous solution (10 mL), extracted with ethyl acetate (3×10 mL) and organic phase was combined and washed with brine, dried over anhydrous Na_2SO_4, concentrated under reduced pressure. The crude product was purified with column chromatography on silica gel (200–300 mesh) with PE/EA (10/1, v/v) as eluent to afford 51.0 mg of the title compound as a colourless oil (74% yield).

R_f = 0.58 (PE/EA = 4/1, v/v). NMR Spectroscopy: ^1H NMR (400 MHz, $CDCl_3$, 25 °C) *δ* 8.38 (d, *J* = 6.0 Hz, 1H), 8.03–8.00 (m, 1H), 7.67–7.61 (m, 3H), 7.38 (d, *J* = 8.7 Hz, 2H), 7.04 (d, *J* = 8.6 Hz, 2H), 5.51 (s, 1H), 5.16–5.15 (m, 1H), 3.89 (s, 3H), 2.04–1.98 (m, 1H), 1.72–1.58 (m, 3H), 1.33–1.23 (m, 6H), 0.88–0.84 (m, 3H); ^{13}C NMR (101 MHz, $CDCl_3$, 25 °C) *δ* 161.9, 159.5, 140.6, 140.0, 135.2, 131.8, 131.2, 130.9, 126.9, 125.2, 123.2, 118.6, 114.1, 69.9, 55.5, 39.6, 32.0, 29.4, 25.8, 22.8, 14.2. IR (ATR): 3041, 3000, 2926, 2855, 1610, 1513, 1244, 1177, 1036, 820 cm^{-1}. HRMS (ESI, m/z): calcd for $C_{23}H_{28}NO_2^+$ $(M+H)^+$: 350.2115; Found: 350.2114.

1-(5-(4-Chlorophenyl)isoquinolin-1-yl)heptan-1-ol (5p)

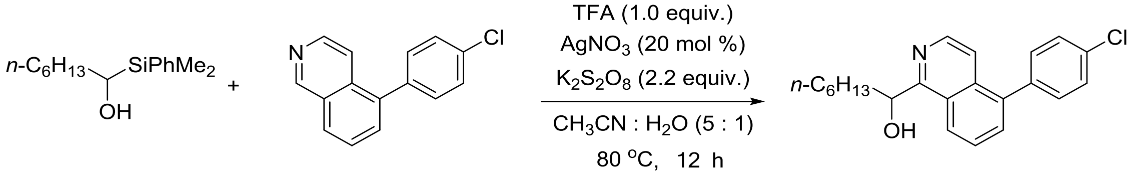

1a **4p** **5p**

Under N_2 atmosphere, $AgNO_3$ (6.8 mg, 0.04 mmol, 20 mol%), CH_3CN/H_2O (1.67 mL / 0.33 mL), **4p** (47.8 mg, 0.20 mmol), TFA (22.8 mg, 0.2 mmol, 1.0 equiv.), **1a** (100.0 mg, 0.40 mmol, 2.0 equiv.), $K_2S_2O_8$ (118.8 mg, 0.44 mmol, 2.2 equiv.) was added into a reaction tube. The tube was then sealed, and the resulting mixture was kept stirring at 80 °C in heating block for 12 h. The reaction mixture was quenched with saturated $NaHCO_3$ aqueous solution (10 mL), extracted with ethyl acetate (3×10 mL) and organic phase was combined and washed with brine, dried over anhydrous Na_2SO_4, concentrated under reduced pressure. The crude product was purified with column chromatography on silica gel (200–300 mesh) with PE/EA (10/1, v/v) as eluent to afford 45.0 mg of the title compound as a colourless oil (64% yield).

R_f = 0.55 (PE/EA = 4/1, v/v). NMR Spectroscopy: ^{1}H NMR (400 MHz, $CDCl_3$, 25 °C) δ 8.41 (d, J = 6.0 Hz, 1H), 8.07 (d, J = 8.2 Hz, 1H), 7.68–7.62 (m, 2H), 7.58 (d, J = 6.0 Hz, 1H), 7.51–7.48 (m, 2H), 7.41 7.39 (m, 2H), 5.50 (s, 1H), 5.12–5.10(m, 1H), 2.04–1.97 (m, 1H), 1.70–1.59 (m, 3H), 1.33–1.25 (m, 6H), 0.88–0.85 (m, 3H); ^{13}C NMR (101 MHz, $CDCl_3$, 25 °C) δ 162.1, 140.9, 139.1, 137.9, 134.8, 134.2, 131.4, 131.0, 128.9, 126.9, 125.2, 124.0, 118.2, 69.9, 39.6, 32.0, 29.4, 25.8, 22.8, 14.2. IR (ATR): 3049, 2926, 2855, 1487, 1416, 1368, 1092, 1017, 816, 731 cm^{-1}. HRMS (ESI, m/z): calcd for $C_{22}H_{24}ClNOH^+$ $(M+H)^+$: 354.1619; Found: 354.1619.

1-(5-([1,1'-Biphenyl]-4-yl)isoquinolin-1-yl)heptan-1-ol (5q)

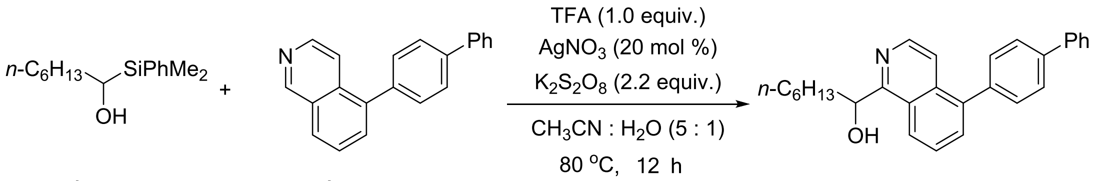

1a **4q** **5q**

Under N_2 atmosphere, $AgNO_3$ (6.8 mg, 0.04 mmol, 20 mol%), CH_3CN/H_2O (1.67 mL / 0.33 mL), **4q** (56.2 mg, 0.20 mmol), TFA (22.8 mg, 0.2 mmol, 1.0 equiv.), **1a** (100.0 mg, 0.40 mmol, 2.0 equiv.), $K_2S_2O_8$ (118.8 mg, 0.44 mmol, 2.2 equiv.) was added into a reaction tube. The tube was then sealed, and the resulting mixture was kept stirring at 80 °C in heating block for 12 h. The reaction mixture was quenched with saturated $NaHCO_3$ aqueous solution (10 mL), extracted with ethyl acetate (3×10 mL) and organic phase was combined and washed with brine, dried over anhydrous Na_2SO_4, concentrated under reduced pressure. The crude product was purified with column chromatography on silica gel (200–300 mesh) with PE/EA (10/1, v/v) as eluent to afford 50.0 mg of the title compound as a colourless oil (63% yield).

R_f = 0.55 (PE/EA = 4/1, v/v). NMR Spectroscopy: ^{1}H NMR (400 MHz, $CDCl_3$, 25 °C) δ 8.42 (d, J = 6.0 Hz, 1H), 8.12–8.04 (m, 1H), 7.82–7.66 (m, 7H), 7.61–7.46 (m, 4H), 7.45–7.37 (m, 1H), 5.53 (dd, J = 8.4, 4.0 Hz, 1H), 5.18 (s, 1H), 2.08–1.98 (m, 1H), 1.75–1.59 (m, 3H), 1.34–1.27 (m, 6H), 0.90–0.87 (m, 3H); ^{13}C NMR (101 MHz, $CDCl_3$, 25 °C) δ 161.9, 140.8, 140.7, 140.6, 139.9, 138.4, 134.9, 130.9, 130.5, 129.0, 127.7, 127.4, 127.2, 126.9, 125.2, 123.6, 118.5, 69.9, 39.6, 31.9, 29.4, 25.8, 22.7, 14.2. IR (ATR): 3053, 2922, 2855, 1610, 1487, 1368, 1073, 846, 760, 697 cm^{-1}. HRMS (ESI, m/z): calcd for $C_{28}H_{30}NO^+$ $(M+H)^+$: 396.2322; Found: 396.2322.

1-(4-Bromoisoquinolin-1-yl)dodecan-1-ol (5r)

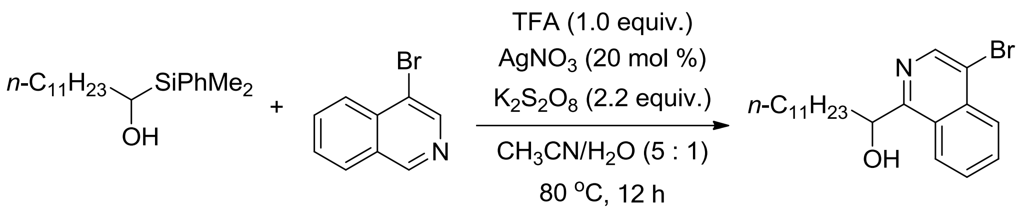

1d **4r** **5r**

Under N_2 atmosphere, $AgNO_3$ (6.8 mg, 0.04 mmol, 20 mol%), CH_3CN/H_2O (1.67 mL / 0.33 mL), **4r** (41.6 mg, 0.20 mmol), TFA (22.8 mg, 0.2 mmol, 1.0 equiv.), **1d** (100 mg, 0.40 mmol, 2.0 equiv.), $K_2S_2O_8$ (118.8 mg, 0.44 mmol, 2.2 equiv.) was added into a reaction tube. The tube was then sealed, and the resulting mixture was kept stirring at 80 °C in heating block for 12 h. The reaction mixture was quenched with saturated $NaHCO_3$ aqueous solution (10 mL), extracted with ethyl acetate (3×10 mL) and organic phase was combined and washed with brine, dried over anhydrous Na_2SO_4, concentrated under reduced pressure. The crude product was purified with column chromatography on silica gel (200–300 mesh) with PE/EA (6/1, v/v) as eluent to afford 50.0 mg of the title compound as a white solid (64% yield).
R_f = 0.71 (PE/EA = 4/1, v/v), White solid, mp: 59 °C–63 °C NMR Spectroscopy: ^1H NMR (400 MHz, $CDCl_3$, 25 °C) δ 8.64 (s, 1H), 8.24–8.22 (m, 1H), 8.04 (d, J = 8.4 Hz, 1H), 7.85–7.80 (m, 1H), 7.71–7.67 (m, 1H), 5.43 (m, J = 1H), 4.75 (s, 1H), 1.23 (s, 20H), 0.87 (t, J = 6.9 Hz, 3H); ^{13}C NMR (101 MHz, $CDCl_3$, 25 °C) δ 161.4, 142.3, 135.1, 131.6, 128.4, 127.1, 126.1, 124.6, 119.0, 69.8, 39.5, 32.1, 29.8, 29.8, 29.7, 29.7, 29.7, 29.5, 25.7, 22.8, 14.3. IR (ATR): 3403, 2922, 2851, 1569, 1498, 1364, 1271, 1080, 928, 760 cm^{-1}. HRMS (ESI, m/z): calcd for $C_{21}H_{30}BrNONa^+$ $(M+Na)^+$: 414.1403; Found: 414.1403.

1-(Phenanthridin-6-yl)heptan-1-ol (5s)

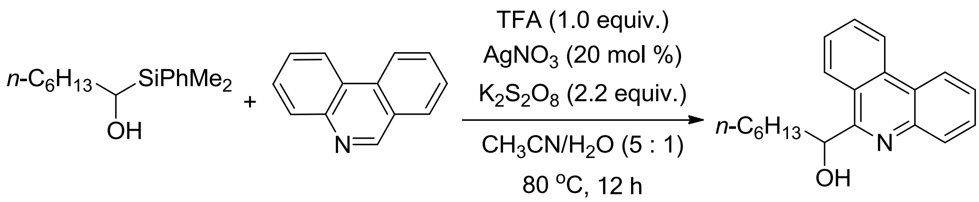

1a **4s** **5s**

Under N_2 atmosphere, $AgNO_3$ (6.8 mg, 0.04 mmol, 20 mol%), CH_3CN/H_2O (1.67 mL / 0.33 mL), **4s** (35.8 mg, 0.20 mmol), TFA (22.8 mg, 0.2 mmol, 1.0 equiv.), **1a** (100 mg, 0.40 mmol, 2.0 equiv.) , $K_2S_2O_8$ (118.8 mg, 0.44 mmol, 2.2 equiv.) was added into a reaction tube. The tube was then sealed, and the resulting mixture was kept stirring at 80 °C in heating block for 12 h. The reaction mixture was quenched with saturated $NaHCO_3$ aqueous solution (10 mL) , extracted with ethyl acetate (3×10 mL) and organic phase was combined and washed with brine, dried over anhydrous Na_2SO_4, concentrated under reduced pressure. The crude product was purified with column chromatography on silica gel (200–300 mesh) with PE/EA (6/1, v/v) as eluent to afford 30.0 mg of the title compound as a colourless oil (51% yield).
R_f = 0.46 (PE/EA = 10/1, v/v). NMR Spectroscopy: ^1H NMR (400 MHz, $CDCl_3$, 25 °C) δ 8.66 (d, J = 8.3 Hz, 1H), 8.57–8.54 (m, 1H), 8.16–8.14 (m, 1H), 8.12–8.10 (m, 1H), 7.88–7.84 (m, 1H), 7.77–7.70 (m, 2H), 7.70–7.64 (m, 1H), 5.52–5.49 (m, 1H), 1.76–1.61 (m, 2H), 1.44–1.23 (m, 8H), 0.90–0.82 (m, 3H); ^{13}C NMR (101 MHz, $CDCl_3$, 25 °C) δ 161.6, 142.2, 133.4, 130.9, 129.6, 129.0, 127.5, 127.0, 125.3, 124.2, 123.3, 122.9, 122.2, 69.9, 39.2, 31.9, 29.4, 25.9, 22.8, 14.2. IR (ATR): 3384, 2922, 2855, 1584, 1461, 1353, 1238, 1129, 1077, 760 cm^{-1}. HRMS (ESI, m/z): calcd for $C_{20}H_{23}NONa^+$ $(M+Na)^+$: 316.1672;

Found: 316.1672.

1-(5-Bromoisoquinolin-1-yl)dodecan-1-ol (5t)

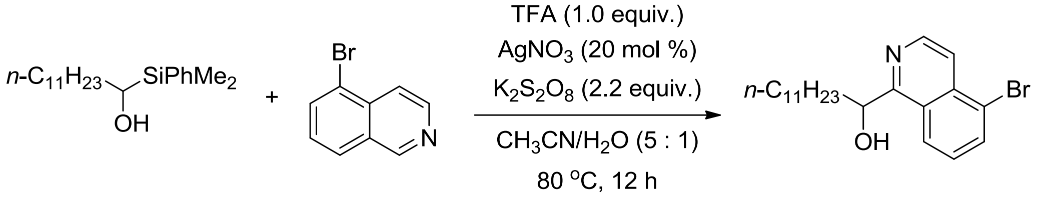

1d **4m** **5t**

Under N_2 atmosphere, $AgNO_3$ (6.8 mg, 0.04 mmol, 20 mol%), CH_3CN/H_2O (1.67 mL / 0.33 mL), **4m** (41.6 mg, 0.20 mmol), TFA (22.8 mg, 0.2 mmol, 1.0 equiv.), **1d** (128.0 mg, 0.40 mmol, 2.0 equiv.), $K_2S_2O_8$ (118.8 mg, 0.44 mmol, 2.2 equiv.) was added into a reaction tube. The tube was then sealed, and the resulting mixture was kept stirring at 80 °C in heating block for 12 h. The reaction mixture was quenched with saturated $NaHCO_3$ aqueous solution (10 mL), extracted with ethyl acetate (3×10 mL) and organic phase was combined and washed with brine, dried over anhydrous Na_2SO_4, concentrated under reduced pressure. The crude product was purified with column chromatography on silica gel (200–300 mesh) with PE/EA (20/1, v/v) as eluent to afford 47.0 mg of the title compound as a white solid (60% yield). R_f = 0.47 (PE/EA = 9/1, v/v), White solid, mp: 48 °C–52 °C. NMR Spectroscopy: ^1H NMR (400 MHz, $CDCl_3$, 25 °C) δ 8.54 (d, J = 6.0 Hz, 1H), 8.03–7.95 (m, 3H), 7.50–7.44 (m, 1H), 5.45–5.44 (m, 1H), 4.99 (s, 1H), 1.71–1.49 (m, 2H), 1.23 (s, 18H), 0.87 (t, J = 6.9 Hz, 3H); ^{13}C NMR (101 MHz, $CDCl_3$, 25 °C) δ 162.2, 141.9, 135.7, 134.1, 127.7, 126.1, 123.9, 122.7, 119.5, 69.9, 39.6, 32.0, 29.8, 29.7, 29.7, 29.7, 29.5, 25.7, 22.8, 14.3. IR (ATR): 3414, 2922, 2855, 1580, 1491, 1327, 1245, 1085, 816, 753 cm^{-1}. HRMS (ESI, m/z): calcd for $C_{21}H_{30}BrNONa^+$ $(M+Na)^+$: 414.1400 ; Found: 414.1403.

1-(4-Bromoisoquinolin-1-yl)heptan-1-ol (5u)

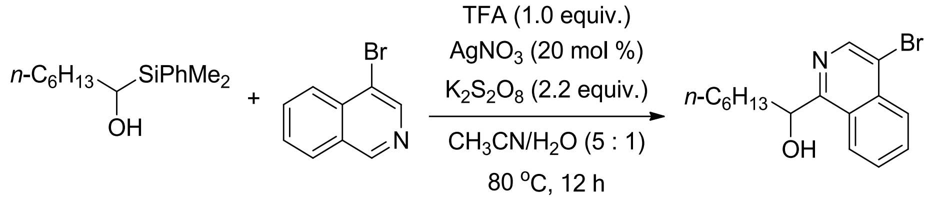

1a **4r** **5u**

Under N_2 atmosphere, $AgNO_3$ (6.8 mg, 0.04 mmol, 20 mol%), CH_3CN/H_2O (1.67 mL / 0.33 mL), **4r** (41.6 mg, 0.20 mmol), TFA (22.8 mg, 0.2 mmol, 1.0 equiv.), **1a** (100 mg, 0.40 mmol, 2.0 equiv.), $K_2S_2O_8$ (118.8 mg, 0.44 mmol, 2.2 equiv.) was added into a reaction tube. The tube was then sealed, and the resulting mixture was kept stirring at 80 °C in heating block for 12 h. The reaction mixture was quenched with saturated $NaHCO_3$ aqueous solution (10 mL), extracted with ethyl acetate (3×10 mL) and organic phase was combined and washed with brine, dried over anhydrous Na_2SO_4, concentrated under reduced pressure. The crude product was purified with column chromatography on silica gel (200–300 mesh) with PE/EA (10/1, v/v) as eluent to afford 43.0 mg of the title compound as a colourless oil (67% yield). R_f = 0.66 (PE/EA = 4/1, v/v). NMR Spectroscopy: ^1H NMR (400 MHz, $CDCl_3$, 25 °C) δ 8.62 (s, 1H), 8.21 (d, J = 8.3 Hz, 1H), 8.02 (d, J = 8.4 Hz, 1H), 7.84–7.80 (m, 1H), 7.69–7.67 (m, 1H), 5.44–5.40 (m, 1H), 4.75 (d, J = 6.7 Hz, 1H), 1.93 (m, 2H), 1.50–1.15 (m, 8H), 0.84 (t, J = 6.9 Hz, 3H); ^{13}C NMR (101 MHz, $CDCl_3$, 25 °C) δ 161.4, 142.3, 135.1, 131.6, 128.3, 127.1, 126.1, 124.6, 118.9, 69.8, 39.5, 31.9,

29.4, 25.7, 22.7, 14.2. IR (ATR): 3422, 2922, 2855, 1569, 1498, 1368, 1238, 1074, 902, 760 cm^{-1}. HRMS (ESI, m/z): calcd for $C_{16}H_{20}BrNONa^+$ $(M+Na)^+$: 344.0620; Found: 344.0620.

1-(4-Bromoisoquinolin-1-yl)-4-((4-fluorobenzyl)oxy)butan-1-ol (5v)

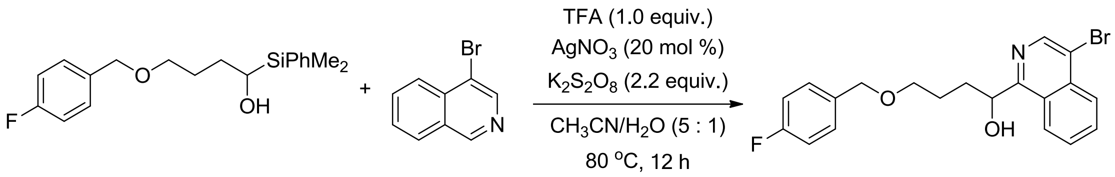

1k **4r** **5v**

Under N_2 atmosphere, $AgNO_3$ (6.8 mg, 0.04 mmol, 20 mol%), CH_3CN/H_2O (1.67 mL / 0.33 mL), **4r** (41.6 mg, 0.20 mmol), TFA (22.8 mg, 0.2 mmol, 1.0 equiv.), **1k** (132.8 mg, 0.40 mmol, 2.0 equiv.) , $K_2S_2O_8$ (118.8 mg, 0.44 mmol, 2.2 equiv.) was added into a reaction tube. The tube was then sealed, and the resulting mixture was kept stirring at 80 °C in heating block for 12 h. The reaction mixture was quenched with saturated $NaHCO_3$ aqueous solution (10 mL), extracted with ethyl acetate (3×10 mL) and organic phase was combined and washed with brine, dried over anhydrous Na_2SO_4, concentrated under reduced pressure. The crude product was purified with column chromatography on silica gel (200–300 mesh) with PE/EA (10/1, v/v) as eluent to afford 45.0 mg of the title compound as a colourless oil (58% yield).

R_f = 0.29 (PE/EA = 4/1, v/v). NMR Spectroscopy: ^1H NMR (400 MHz, $CDCl_3$, 25 °C) δ 8.54 (d, J = 6.0 Hz, 1H), 8.02 (d, J = 8.5 Hz, 1H), 7.98–7.95 (m, 2H), 7.39–7.35 (m, 1H), 7.29–7.23 (m, 2H), 7.03–6.96 (m, 2H), 5.50 (dd, J = 8.4, 2.9 Hz, 1H), 5.06 (s, 1H), 4.44 (s, 2H), 3.64–3.46 (m, 2H), 2.22–1.87 (m, 2H), 1.87–1.59 (m, 2H); ^{13}C NMR (101 MHz, $CDCl_3$, 25 °C) δ 162.4 (d, J = 245.4 Hz), 161.9, 141.9, 135.7, 134.4 (d, J = 3.3 Hz), 134.2, 129.5 (d, J = 8.1 Hz), 127.8, 126.0, 124.0, 122.7, 119.6, 115.3 (d, J = 21.4 Hz), 72.3, 70.0, 69.3, 35.9, 25.6. ^{19}F NMR (375 MHz, $CDCl_3$) δ –114.9 (m, 1F). IR (ATR): 3391, 2919, 2855, 1580, 1509, 1323, 1223, 1092, 820, 753 cm^{-1}. HRMS (ESI, m/z): calcd for $C_{20}H_{19}BrFNO_2Na^+$ $(M+Na)^+$: 426.0500 ; Found: 426.0475.

4-((3-Chlorobenzyl)oxy)-1-(5-(4-methoxyphenyl)quinolin-2-yl)butan-1-ol (5w)

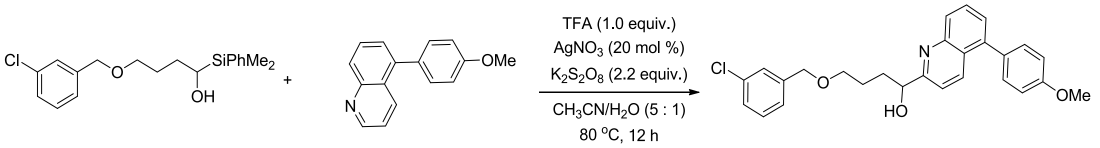

1l **4o** **5w**

Under N_2 atmosphere, $AgNO_3$ (6.8 mg, 0.04 mmol, 20 mol%), CH_3CN/H_2O (1.67 mL / 0.33 mL), **4o** (47.0 mg, 0.20 mmol), TFA (22.8 mg, 0.2 mmol, 1.0 equiv.), **1l** (132.8 mg, 0.40 mmol, 2.0 equiv.), $K_2S_2O_8$ (118.8 mg, 0.44 mmol, 2.2 equiv.) was added into a reaction tube. The tube was then sealed, and the resulting mixture was kept stirring at 80 °C in heating block for 12 h. The reaction mixture was quenched with saturated $NaHCO_3$ aqueous solution (10 mL), extracted with ethyl acetate (3×10 mL) and organic phase was combined and washed with brine, dried over anhydrous Na_2SO_4, concentrated under reduced pressure. The crude product was purified with column chromatography on silica gel (200–300 mesh) with PE/EA (4/1, v/v) as eluent to afford 43.0 mg of the title compound as a colourless oil (50% yield).

R_f = 0.54 (PE/EA = 2/1, v/v). NMR Spectroscopy: ^1H NMR (400 MHz, $CDCl_3$, 25 °C) δ 8.39 (d, J = 6.0 Hz, 1H), 8.06 (d, J = 8.1 Hz, 1H), 7.71–7.69 (m, 1H), 7.66–7.58 (m, 2H), 7.40–7.36 (m, 2H), 7.33–7.32 (m, 1H), 7.26–7.24 (m, 2H), 7.21–7.17 (m, 1H), 7.07–7.03 (m, 2H), 5.58 (dd, J = 8.3, 3.0 Hz, 1H), δ

4.46 (d, J = 2.0 Hz, 2H)., 3.90 (s, 3H), 3.67–3.51 (m, 2H), 2.25–2.17 (m, 1H), 2.03–1.93 (m, 1H), 1.90–1.73 (m, 2H); ^{13}C NMR (101 MHz, $CDCl_3$, 25 °C) δ 161.6, 159.5, 140.8, 140.1, 140.0, 135.3, 134.4, 131.6, 131.2, 131.2, 129.8, 127.7, 127.7, 127.2, 125.7, 125.2, 123.4, 118.9, 114.1, 72.2, 70.4, 69.3, 55.5, 35.7, 25.7. IR (ATR): 3034, 3000, 2926, 2855, 1610, 1513, 1245, 1036, 820, 782 cm^{-1}. HRMS (ESI, m/z): calcd for $C_{27}H_{27}ClNO_3^+$ $(M+H)^+$: 448.1674 ; Found: 448.1674.

1-(5-Bromoisoquinolin-1-yl)-4-(3-phenylpropoxy)butan-1-ol (5x)

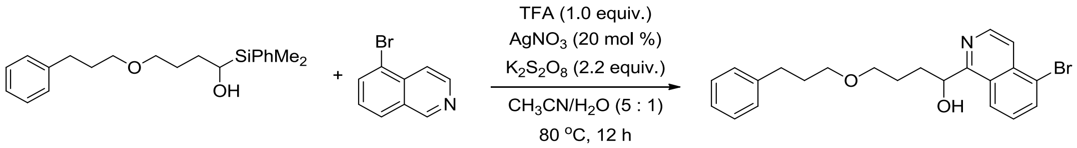

Under N_2 atmosphere, $AgNO_3$ (6.8 mg, 0.04 mmol, 20 mol%), CH_3CN/H_2O (1.67 mL / 0.33 mL), **4m** (41.6 mg, 0.20 mmol), TFA (22.8 mg, 0.2 mmol, 1.0 equiv.), **1s** (136.8 mg, 0.40 mmol, 2.0 equiv.) , $K_2S_2O_8$ (118.8 mg, 0.44 mmol, 2.2 equiv.) was added into a reaction tube. The tube was then sealed, and the resulting mixture was kept stirring at 80 °C in heating block for 12 h. The reaction mixture was quenched with saturated $NaHCO_3$ aqueous solution (10 mL), extracted with ethyl acetate (3×10 mL) and organic phase was combined and washed with brine, dried over anhydrous Na_2SO_4, concentrated under reduced pressure. The crude product was purified with column chromatography on silica gel (200–300 mesh) with PE/EA (10/1, v/v) as eluent to afford 51.0 mg of the title compound as a colourless oil (62% yield).

R_f = 0.33 (PE/EA = 4/1, v/v). NMR Spectroscopy: ^{1}H NMR (400 MHz, $CDCl_3$, 25 °C) δ 8.56 (d, J = 6.0 Hz, 1H), 8.09 (d, J = 8.5 Hz, 1H), 8.0–7.96 (m, 2H), 7.46–7.44 (m, 1H), 7.29–7.22 (m, 2H), 7.20–7.12 (m, 3H), 5.52 (dd, J = 8.0, 2.5 Hz, 1H), 5.06 (s, 1H), 3.59–3.35 (m, 4H), 2.69–2.61 (m, 2H), 1.97–1.83 (m, 3H), 1.83–1.65 (m, 3H); ^{13}C NMR (101 MHz, $CDCl_3$, 25 °C) δ 162.0, 142.1, 141.9, 135.8, 134.2, 128.6, 128.4, 127.8, 126.1, 125.9, 124.1, 122.7, 119.6, 70.5, 70.1, 69.5, 36.0, 32.5, 31.5, 25.8. IR (ATR): 3396, 2922, 2855, 1580, 1490, 1364, 1207, 1111, 816, 749 cm^{-1}. HRMS (ESI, m/z): calcd for $C_{22}H_{24}BrNO_2Na^+$ $(M+Na)^+$: 436.0883 ; Found: 436.0883.

1-(4-Bromoisoquinolin-1-yl)-6-((4-(trifluoromethoxy)benzyl)oxy)hexan-1-ol (5y)

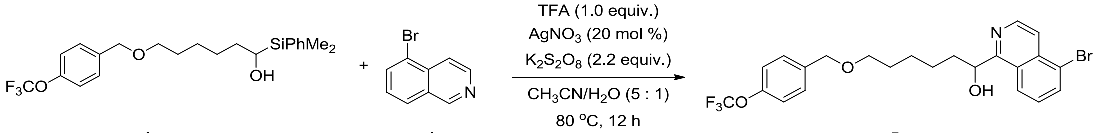

Under N_2 atmosphere, $AgNO_3$ (6.8 mg, 0.04 mmol, 20 mol%), CH_3CN/H_2O (1.67 mL / 0.33 mL), **4m** (41.6 mg, 0.20 mmol), TFA (22.8 mg, 0.2 mmol, 1.0 equiv.), **1w** (159.2 mg, 0.40 mmol, 2.0 equiv.) , $K_2S_2O_8$ (118.8 mg, 0.44 mmol, 2.2 equiv.) was added into a reaction tube. The tube was then sealed, and the resulting mixture was kept stirring at 80 °C in heating block for 12 h. The reaction mixture was quenched with saturated $NaHCO_3$ aqueous solution (10 mL) , extracted with ethyl acetate (3×10 mL) and organic phase was combined and washed with brine, dried over anhydrous Na_2SO_4, concentrated under reduced pressure. The crude product was purified with column chromatography on silica gel (200–300 mesh) with PE/EA (10/1, v/v) as eluent to afford 56.0 mg of the title compound as a colourless oil (56% yield).

R_f = 0.25 (PE/EA = 4/1, v/v). NMR Spectroscopy: ^{1}H NMR (400 MHz, $CDCl_3$, 25 °C) δ 8.55 (d, J = 5.9

Hz, 1H), 8.03–7.96 (m, 3H), 7.48–7.46 (m, 1H), 7.36–7.31 (m, 2H), 7.20–7.14 (m, 2H), 5.49–5.41 (m, 2H), 5.03 (s, 1H), 4.46 (s, 2H), 3.45 (t, J = 6.5 Hz, 2H), 2.01–1.90 (m, 1H), 1.71–1.56 (m, 4H), 1.54–1.32 (m, 3H); ^{13}C NMR (101 MHz, $CDCl_3$, 25 °C) δ 162.0, 148.7 (d, J = 1.9 Hz), 141.9, 137.6, 135.8, 134.2, 129.0, 127.8, 126.0, 123.8, 122.8, 121.0 (q, J = 1.2 Hz), 120.6 (q, J = 256.8 Hz), 119.5, 72.1, 70.7, 69.8, 39.4, 29.8, 26.3, 25.5. ^{19}F NMR (375 MHz, $CDCl_3$) δ –57.8 (s, 3F). IR (ATR): 3388, 2930, 2855, 1613, 1509, 1256, 1021, 920, 816, 752 cm^{-1}. HRMS (ESI, m/z): calcd for $C_{23}H_{23}BrF_3NO_3Na^+$ (M+Na)$^+$: 520.0700 ; Found: 520.0705.

6-(Benzyloxy)-1-(2-(4-chlorophenyl)quinolin-4-yl)hexan-1-ol (5z)

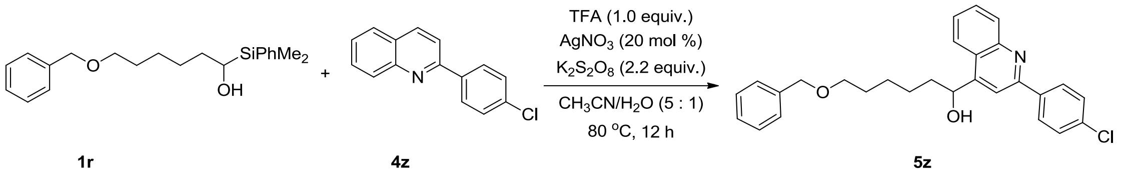

Under N_2 atmosphere, $AgNO_3$ (6.8 mg, 0.04 mmol, 20 mol%), CH_3CN/H_2O (1.67 mL / 0.33 mL), **4z** (47.8 mg, 0.20 mmol), TFA (22.8 mg, 0.2 mmol, 1.0 equiv.), **1r** (136.8 mg, 0.40 mmol, 2.0 equiv.) , $K_2S_2O_8$ (118.8 mg, 0.44 mmol, 2.2 equiv.) was added into a reaction tube. The tube was then sealed, and the resulting mixture was kept stirring at 80 °C in heating block for 12 h. The reaction mixture was quenched with saturated $NaHCO_3$ aqueous solution (10 mL), extracted with ethyl acetate (3×10 mL) and organic phase was combined and washed with brine, dried over anhydrous Na_2SO_4, concentrated under reduced pressure. The crude product was purified with column chromatography on silica gel (200–300 mesh) with PE/EA (4/1, v/v) as eluent to afford 45.0 mg of the title compound as a colourless oil (50% yield).

R_f = 0.35 (PE/EA = 4/1, v/v). NMR Spectroscopy: ^{1}H NMR (400 MHz, $CDCl_3$, 25 °C) δ 8.40 (d, J = 5.9 Hz, 1H), 8.06 (d, J = 8.1 Hz, 1H), 7.70–7.62 (m, 2H), 7.60–7.58 (m, 1H), 7.51–7.48 (m, 2H), 7.41–7.38 (m, 2H), 7.33 (d, J = 4.0 Hz, 4H), 7.29–7.25 (m, 1H), 5.52–5.50 (m, 1H), 5.14 (s, 1H), 4.49 (s, 2H), 3.47 (t, J = 6.6 Hz, 2H), 1.73–1.53 (m, 6H), 1.52–1.36 (m, 2H); ^{13}C NMR (101 MHz, $CDCl_3$, 25 °C) δ 161.9, 140.9, 139.0, 138.8, 137.8, 134.8, 134.2, 131.3, 131.0, 128.9, 128.5, 127.8, 127.6, 126.9, 125.1, 123.9, 118.2, 73.0, 70.5, 69.8, 39.4, 29.9, 26.3, 25.6. IR (ATR): 3034, 3030, 2933, 2855, 1487, 1368, 1092, 1017, 910, 816 cm^{-1}. HRMS (ESI, m/z): calcd for $C_{28}H_{29}ClNO_2^+$ (M+H)$^+$: 446.1881 ; Found: 446.1881.

1-(2-(4-(Hydroxymethyl)phenyl)quinolin-4-yl)heptan-1-ol (5aa)

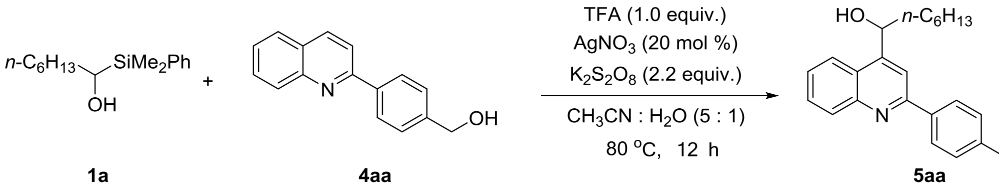

Under N_2 atmosphere, $AgNO_3$ (6.8 mg, 0.04 mmol, 20 mol%), CH_3CN/H_2O (1.67 mL / 0.33 mL), **4aa** (47.0 mg, 0.20 mmol), TFA (22.8 mg, 0.2 mmol, 1.0 equiv.), **1a** (159.2 mg, 0.40 mmol, 2.0 equiv.), $K_2S_2O_8$ (118.8 mg, 0.44 mmol, 2.2 equiv.) was added into a reaction tube. The tube was then sealed, and the resulting mixture was kept stirring at 80 °C in heating block for 12 h. The reaction mixture was quenched with saturated $NaHCO_3$ aqueous solution (10 mL) , extracted with ethyl acetate (3×10 mL) and organic phase was combined and washed with brine, dried over anhydrous Na_2SO_4, concentrated under reduced pressure. The crude product was purified with column chromatography on silica gel

(200–300 mesh) with PE/EA (1/1, v/v) as eluent to afford 35.0 mg of the title compound as a white oil (50% yield).

R_f = 0.42 (PE/EA = 1/1, v/v). NMR Spectroscopy: ^{1}H NMR (600 MHz, $CDCl_3$, 25 °C) δ 8.17 (dd, J = 8.5, 1.2 Hz, 1H), 7.92–7.82 (m, 2H), 7.80 (d, J = 9.0 Hz, 2H), 7.69–7.66 (m, 1H), 7.46–1.43 (m, 1H), 7.23 (d, J = 8.0 Hz, 2H), 5.29 (dd, J = 8.5, 3.9 Hz, 1H), 4.61 (s, 2H), 3.65 (s, 1H), 3.16 (s, 1H), 1.91−1.65 (m, 2H), 1.60−1.39 (m, 2H), 1.35−1.20 (m, 5H), 0.85 (t, J = 6.9 Hz, 3H); ^{13}C NMR (101 MHz, $CDCl_3$, 25 °C) δ 157.0, 152.0, 148.1, 142.4, 138.4, 130.1, 129.4, 127.7, 127.1, 126.2, 124.6, 122.9, 115.4, 70.0, 64.7, 38.6, 31.9, 29.2, 26.2, 22.7, 14.2. IR (ATR): 3332, 2866, 1595, 1505, 1423, 1353, 1203, 1110, 1107, 909 cm^{-1}. HRMS (ESI, m/z): calcd for $C_{23}H_{28}NO_2^+$ (M+H)$^+$: 350.2111 ; Found: 350.2115.

4-((3-Chlorobenzyl)oxy)-1-(2-(4-(hydroxymethyl)phenyl)quinolin-4-yl)butan-1-ol (5ab)

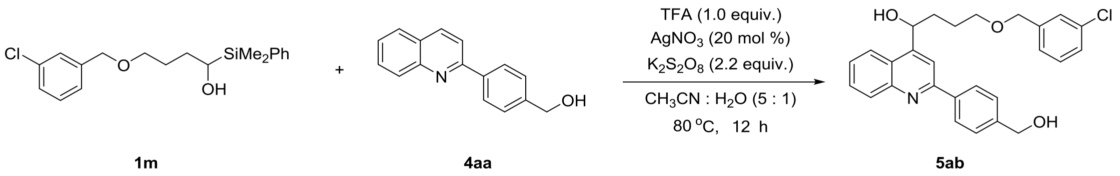

Under N_2 atmosphere, $AgNO_3$ (6.8 mg, 0.04 mmol, 20 mol%), CH_3CN/H_2O (1.67 mL / 0.33 mL), **4aa** (47.0 mg, 0.20 mmol), TFA (22.8 mg, 0.2 mmol, 1.0 equiv.), **1m** (139.2 mg, 0.40 mmol, 2.0 equiv.), $K_2S_2O_8$ (118.8 mg, 0.44 mmol, 2.2 equiv.) was added into a reaction tube. The tube was then sealed, and the resulting mixture was kept stirring at 80 °C in heating block for 12 h. The reaction mixture was quenched with saturated $NaHCO_3$ aqueous solution (10 mL) , extracted with ethyl acetate (3×10 mL) and organic phase was combined and washed with brine, dried over anhydrous Na_2SO_4, concentrated under reduced pressure. The crude product was purified with column chromatography on silica gel (200–300 mesh) with EA (EA=100%) as eluent to afford 46.0 mg of the title compound as a white oil (51% yield)

R_f = 0.24 (PE/EA = 1/1, v/v). NMR Spectroscopy: ^{1}H NMR (600 MHz, $CDCl_3$, 25 °C) δ 8.17 (dd, J = 8.5, 1.2 Hz, 1H), 7.96–7.93 (m, 2H), 7.90 (s, 1H), 7.83 (dd, J = 8.5, 1.4 Hz, 1H), 7.69–7.66 (m, 1H), 7.45–7.42 (m, 1H), 7.34–7.28 (m, 3H), 7.25 (dd, J = 5.8, 4.5 Hz, 2H), 7.21–7.16 (m, 1H), 5.42–5.34 (m, 1H), 4.65 (s, 2H), 4.47 (s, 2H), 3.59–3.48 (m, 2H), 2.81 (s, 1H), 2.09–2.01 (m, 1H), 1.88–1.81 (m, 3H); ^{13}C NMR (151 MHz, $CDCl_3$, 25 °C) δ 157.0, 151.4, 148.2, 142.3, 140.2, 138.6, 134.5, 130.2, 129.9, 129.4, 128.0, 127.8, 127.7, 127.2, 126.2, 125.8, 124.5, 122.9, 115.4, 72.4, 70.5, 69.6, 64.9, 35.7, 26.3. IR (ATR): 3339, 2926, 2855, 1599, 1550, 1457, 1423, 1121, 1051, 760 cm^{-1}. HRMS (ESI, m/z): calcd for $C_{27}H_{27}ClNO_3^+$ (M+H)$^+$: 448.1668 ; Found: 448.1674.

1-(5-bromoisoquinolin-1-yl)-2-methylbutan-1-ol (5ac)

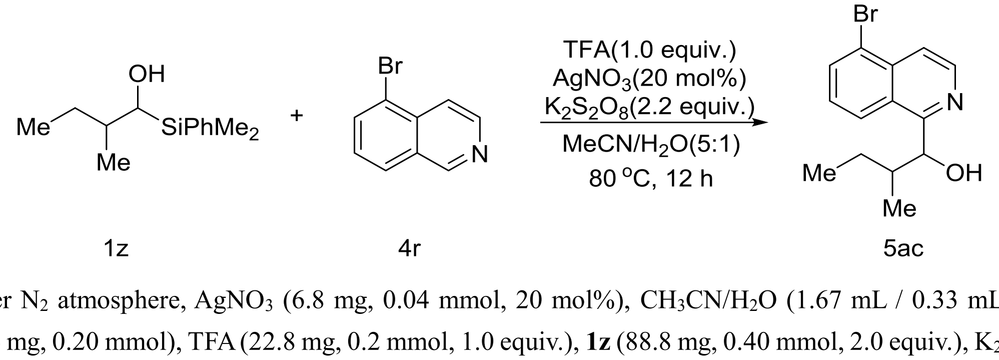

Under N_2 atmosphere, $AgNO_3$ (6.8 mg, 0.04 mmol, 20 mol%), CH_3CN/H_2O (1.67 mL / 0.33 mL), **4r** (41.4 mg, 0.20 mmol), TFA (22.8 mg, 0.2 mmol, 1.0 equiv.), **1z** (88.8 mg, 0.40 mmol, 2.0 equiv.), $K_2S_2O_8$

(118.8 mg, 0.44 mmol, 2.2 equiv.) was added into a reaction tube. The tube was then sealed, and the resulting mixture was kept stirring at 80 °C in heating block for 12 h. The reaction mixture was quenched with saturated $NaHCO_3$ aqueous solution (10 mL) , extracted with ethyl acetate (3×10 mL) and organic phase was combined and washed with brine, dried over anhydrous Na_2SO_4, concentrated under reduced pressure. The crude product was purified with column chromatography on silica gel (200–300 mesh) with EA (EA=100%) as eluent to afford 36.0 mg of the title compound as a yellow oil (62% yield)

R_f = 0.68 (PE/EA = 4/1, v/v). NMR Spectroscopy: Isomer I: ^{1}H NMR (600 MHz, $CDCl_3$, 25 °C) δ 8.56 (d, J = 5.9 Hz, 1H), 8.04–7.96 (m, 3H), 7.47 (dd, J = 8.5, 7.5 Hz, 1H), 5.54–5.49 (m, 1H), 4.98 (s, 1H), 1.91–1.87 (m, 1H), 1.84–1.77 (m, 1H), 1.59–1.53 (m, 1H), 1.12 (t, J = 7.4 Hz, 3H), 0.55 (d, J = 6.7 Hz, 3H). ^{13}C NMR (151 MHz, $CDCl_3$, 25 °C) δ 161.5, 141.6, 135.8, 134.1, 127.7, 126.2, 124.0, 122.7, 119.4, 71.7, 42.0, 27.8, 12.4, 12.3; Isomer II: ^{1}H NMR (600 MHz, $CDCl_3$, 25 °C) δ 8.56 (d, J = 5.9 Hz, 1H), 8.04 (d J = 8.5, 1H), 8.02–7.96 (m, 2H), 7.47 (dd, J = 8.5, 7.4 Hz, 1H), 5.35 (d, J = 3.5 Hz, 1H), 4.82 (s, 1H), 1.93–1.89 (m, 1H), 1.22–1.18 (m, 1H), 1.16 (d, J = 6.9 Hz, 3H), 1.13–1.03 (m, 1H), 0.66 (t, J = 7.5 Hz, 3H). ^{13}C NMR (151 MHz, $CDCl_3$, 25 °C) δ 161.6, 141.8, 135.7, 134.1, 127.7, 126.5, 124.2, 122.7, 119.4, 74.2, 42.4, 22.1, 17.3, 11.8. IR (ATR): 3444, 2963, 2933, 1613, 1580, 1490, 1461, 1371, 1326, 1125, 1051 cm^{-1}. HRMS (ESI, m/z): calcd for $C_{14}H_{17}BrNO^+$ $(M+H)^+$: 294.0488 ; Found: 294.0486.

1-(5,6-diphenylpyrazin-2-yl)heptan-1-ol(5ad)

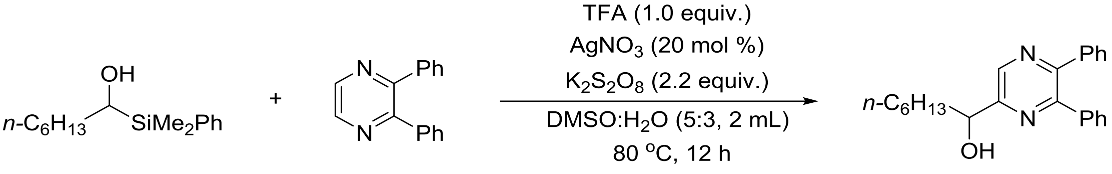

1a 4ad 5ad

Under N_2 atmosphere, $AgNO_3$ (6.8 mg, 0.04 mmol, 20 mol%), DMSO/H_2O (1.25 mL / 0.75 mL), **4ad** (46.4 mg, 0.20 mmol), TFA (22.8 mg, 0.2 mmol, 1.0 equiv.), **1a** (100.0 mg, 0.40 mmol, 2.0 equiv.), $K_2S_2O_8$ (118.8 mg, 0.44 mmol, 2.2 equiv.) was added into a reaction tube. The tube was then sealed, and the resulting mixture was kept stirring at 80 °C in heating block for 12 h. The reaction mixture was quenched with saturated $NaHCO_3$ aqueous solution (10 mL) , extracted with ethyl acetate (3×10 mL) and organic phase was combined and washed with brine, dried over anhydrous Na_2SO_4, concentrated under reduced pressure. The crude product was purified with column chromatography on silica gel (200–300 mesh) with EA (EA=100%) as eluent to afford 24.1 mg of the title compound as a yellow oil (35% yield)

R_f = 0.20 (PE/EA = 10/1, v/v). NMR Spectroscopy: ^{1}H NMR (400 MHz, $CDCl_3$, 25 °C) δ 8.62 (s, 1H), 7.48–7.43 (m, 4H), 7.36–7.29 (m, 6H), 4.93–4.89 (m, J = 7.9, 5.9, 4.8 Hz, 1H), 3.68 (d, J = 6.0 Hz, 1H), 1.98–1.77 (m, 2H), 1.57–1.45 (m, 2H), 1.44–1.28 (m, 6H), 0.89 (t, J = 6.9 Hz, 3H). ^{13}C NMR (151 MHz, $CDCl_3$, 25 °C) δ 155.0, 151.4, 150.9, 139.9, 138.6, 138.4, 129.9, 129.8, 128.9, 128.8, 128.4, 128.4, 71.6, 38.6, 31.9, 29.4, 25.5, 22.8, 14.2. IR (ATR): 3377, 3060, 2926, 2855, 1729, 1453, 1379, 1297, 1155, 1073 cm^{-1}. HRMS (ESI, m/z): calcd for $C_{23}H_{27}N_2O^+$ $(M+H)^+$: 347.2118 ; Found: 347.2106.

Other heterocyclic compounds

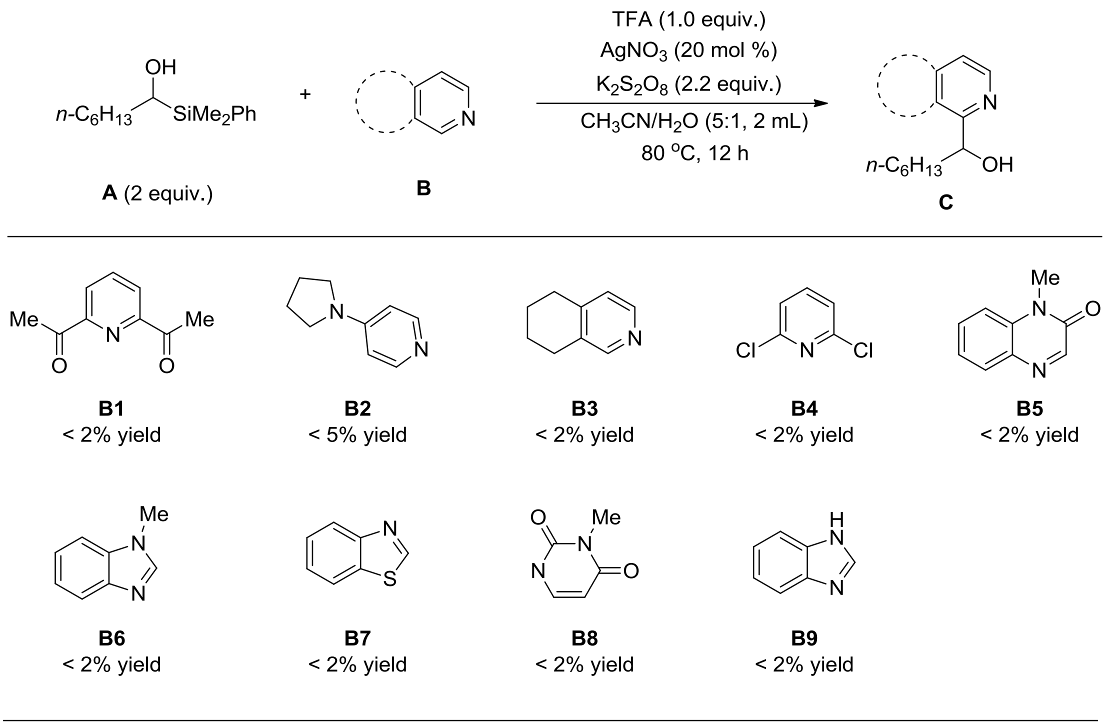

The yield was determined by ^{1}H NMR using mesitylene as an internal standard.

6. Plausible mechanism of Minisci Reaction

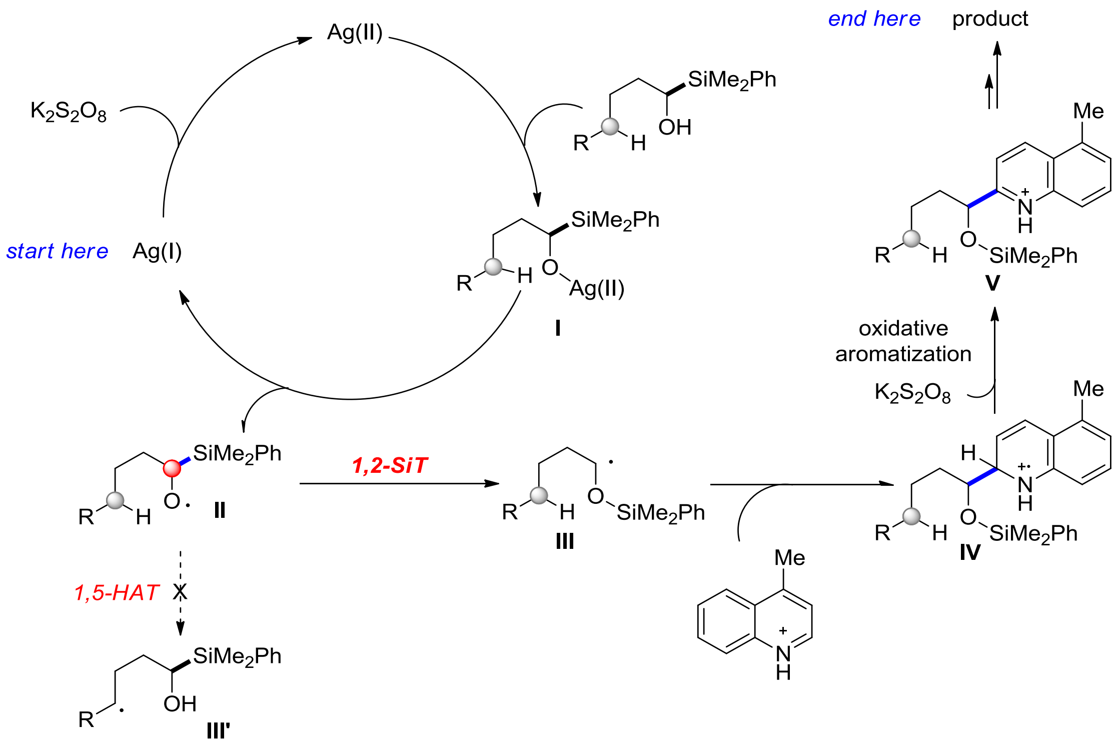

7. Synthesis of α-silyl alcohols

The α-silyl alcohols used in this study are synthesized following the reported methods from corresponding aldehydes (**General procedures A, B and C**).[4,5] Aldehydes that are not commercially available were prepared according to a literature procedure. [6,7]

General procedure A : A solution of $Me_2PhSiLi$ (1.5 equiv.) in THF (1 M) was added dropwise to a stirred solution of the corresponding aldehyde (1.0 equiv.) in THF (1 M) via cannula at –78 ℃. The reaction mixture was allowed to warm to 0 °C slowly before being poured into saturated aqueous NH_4Cl and extracted with ethyl acetate. The combined extracts were dried (Na_2SO_4) and the solvent was removed under reduced pressure to afford the correspond α-silyl alcohol, which was purified by column chromatography on silica gel using PE/EA (v/v) as eluent to afford desired product.

General procedure B: In a glove box, a dry Schlenk tube was successively charged with CuCN (4.5 mg, 0.05 mmol, 5.0 mol%), NaOMe (5.5 mg, 0.10 mmol, 10 mol%) and THF (2.5 mL). The reaction vessel was then capped, removed from the glove box and allowed to stir at room temperature for 1 h. At 0 °C, the indicated corresponding aldehyde (1.0 mmol, 1.0 equiv.), $Me_2PhSiBpin$ (315 mg, 1.2 mmol, 1.2 equiv.), MeOH (128 mg, 4.0 mmol, 4.0 equiv.), and THF (2.5 mL) were added in this order. The reaction was subsequently maintained at 0 °C for 2 h. Filtration through a small pad of silica gel using ethyl acetate (30 mL) and evaporation of the solvents under reduced pressure afforded the corresponding α-silyl alcohol as the crude product. The product was purified by column chromatography on silica gel using PE/EA (v/v) as eluent to afford desired product.

General procedure C: A solution of $Me_2PhSiLi$ (1.5 equiv.) in THF (1 M) was added dropwise to a stirred solution of the corresponding ketone (1.0 equiv.) in Toluene (0.33 M) via cannula at -78 °C. The resulting mixture was kept stirring at -78 °C for 8 h. The reaction mixture was quenched with water (10 mL), extracted with ethyl acetate (3×20 mL) and organic phase was combined and washed with brine, dried over anhydrous Na_2SO_4, concentrated under reduced pressure to afford the correspond α-silyl alcohol, which was purified by column chromatography on silica gel using PE/EA (v/v) as eluent to afford desired product.

1-(Dimethyl(phenyl)silyl)dodecan-1-ol (1d)

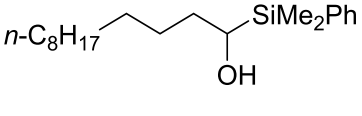

General procedure A: R_f = 0.60 (PE/EA = 10/1, v/v). Yellow oil, (2.24 g, 7.0 mmol, 70% yield). NMR spectroscopy: ^{1}H NMR (400 MHz, $CDCl_3$, 25 °C) δ 7.60–7.54 (m, 2H), 7.38 (m, 3H), 3.52–3.49 (m, 1H), 1.54–1.50 (m, 2H), 1.32–1.25 (m, 18H), 0.88 (t, J = 6.9 Hz, 3H), 0.34 (s, 3H), 0.33 (s, 3H); ^{13}C NMR (101 MHz, $CDCl_3$, 25 °C) δ 137.0, 134.3, 129.4, 128.0, 65.6, 33.6, 32.1, 29.8, 29.8, 29.8, 29.6, 29.5, 27.0, 22.8, 14.3, –5.2, –5.5. IR (ATR): 2922, 2851, 1461, 1427, 1248, 1110, 812, 775, 734, 700 cm^{-1}. HRMS (ESI, m/z): calcd for $C_{20}H_{36}SiONa^+$ $(M+Na)^+$: 343.2433; Found: 343.2428.

4-(Benzyloxy)-1-(dimethyl(phenyl)silyl)butan-1-ol (1g)

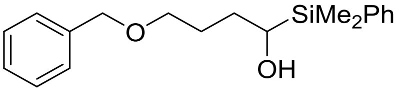

General procedure B: R_f = 0.40 (PE/EA = 4/1, v/v). Colorless oil (249 mg, 0.79 mmol, 79% yield).

NMR spectroscopy: ^{1}H NMR (400 MHz, $CDCl_3$, 25 ºC) δ 7.60–7.55 (m, 2H), 7.39–7.37 (m, 3H), 7.36–7.27 (m, 5H), 4.51 (s, 2H), 3.52–3.48 (m, 3H), 1.99 (s, 1H), 1.88–1.77 (m, 1H), 1.74–1.66 (m, 2H), 1.62–1.51 (m, 1H), 0.35 (s, 3H), 0.34 (s, 3H); ^{13}C NMR (101 MHz, $CDCl_3$, 25 ºC) δ 138.3, 137.1, 134.3, 129.4, 128.5, 128.0, 127.8, 127.5, 73.1, 70.3, 65.1, 30.8, 27.8, -5.3, -5.3. IR (ATR): 3067, 3026, 2922, 2855, 1729, 1494, 1427, 1364, 1248, 1107cm^{-1}. HRMS (ESI, m/z): calcd for $C_{19}H_{26}SiO_2Na^+$ $(M+Na)^+$: 337.1600; Found: 337.1594.

1-(Dimethyl(phenyl)silyl)-5-phenoxypentan-1-ol (1h)

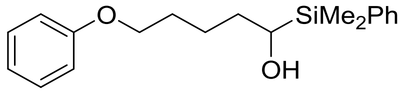

General procedure B: R_f = 0.40 (PE/EA = 4/1, v/v). Yellow oil (251.2 mg, 0.80 mmol, 80% yield). NMR spectroscopy: ^{1}H NMR (400 MHz, $CDCl_3$, 25 ºC) δ 7.59–7.55 (m, 2H), 7.42–7.35 (m, 3H), 7.30–7.24 (m, 2H), 6.96–6.90 (m, 1H), 6.90–6.86 (m, 2H), 3.96–3.92 (m, 2H), 3.54 (dd, J = 7.6, 6.5 Hz, 1H), 1.93–1.69 (m, 3H), 1.66–1.57 (m, 2H), 1.56–1.39 (m, 2H), 0.36 (s, 3H), 0.35 (s, 3H); ^{13}C NMR (101 MHz, $CDCl_3$, 25 ºC) δ 159.2, 136.7, 134.3, 129.5, 129.5, 128.1, 120.6, 114.6, 67.0, 65.5, 33.2, 29.2, 23.6, –5.3, –5.5. IR (ATR): 3440, 3067, 2937, 2855, 1599, 1494, 1300, 1244, 1110, 812 cm^{-1}. HRMS (ESI, m/z): calcd for $C_{19}H_{26}SiO_2Na^+$ $(M+Na)^+$: 337.1590; Found: 337.1594.

1-(Dimethyl(phenyl)silyl)-4-((2-fluorobenzyl)oxy)butan-1-ol (1i)

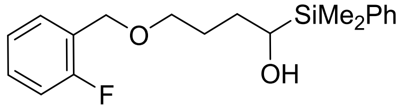

General procedure B: R_f = 0.40 (PE/EA = 4/1, v/v). Colorless oil, (265.6 mg, 0.80 mmol, 80% yield). NMR spectroscopy: ^{1}H NMR (400 MHz, $CDCl_3$, 25 ºC) δ 7.58–7.56 (m, 2H), 7.40–7.35 (m, 3H), 7.33–7.26 (m, 1H), 7.09–6.93 (m, 3H), 4.49 (s, 2H), 3.55–3.46 (m, 3H), 1.89–1.77 (m, 2H), 1.74–1.67 (m, 2H), 1.59–1.53 (m, 1H), 0.35 (s, 3H), 0.34 (s, 3H); ^{13}C NMR (101 MHz, $CDCl_3$, 25 ºC) δ 163.1 (d, J = 246.0 Hz), 141.1 (d, J = 7.2 Hz), 137.0, 134.3, 130.0 (d, J = 8.2 Hz), 129.4, 128.0, 123.1 (d, J = 3.1 Hz), 114.6 (d, J = 7.9 Hz), 114.4 (d, J = 8.3 Hz), 72.3 (d, J = 2.2 Hz), 70.6, 65.2, 30.7, 27.8, –5.3, –5.4. IR (ATR): 3421, 3071, 2918, 2855, 1718, 1591, 1487, 1449, 1356, 1252 cm^{-1}. HRMS (ESI, m/z): calcd for $C_{19}H_{25}SiO_2Na^+$ $(M+Na)^+$: 355.1506; Found: 355.1500.

1-(Dimethyl(phenyl)silyl)-4-((3-fluorobenzyl)oxy)butan-1-ol (1j)

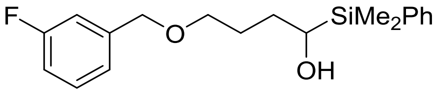

General procedure B: R_f = 0.40 (PE/EA = 4/1, v/v). Colorless oil, (229.1 mg, 0.69 mmol, 69% yield). NMR spectroscopy: ^{1}H NMR (400 MHz, $CDCl_3$, 25 ºC) δ 7.60–7.54 (m, 2H), 7.42–7.34 (m, 4H), 7.32–7.24 (m, 1H), 7.15–7.11 (m, 1H), 7.07–7.00 (m, 1H), 4.57 (s, 2H), 3.57–3.47 (m, 3H), 1.90–1.78 (m, 2H), 1.78–1.64 (m, 2H), 1.62–1.50 (m, 1H), 0.35 (s, 3H), 0.34 (s, 3H); ^{13}C NMR (101 MHz, $CDCl_3$, 25 ºC) δ 160.9 (d, J = 246.6 Hz), 137.0, 134.3, 130.1 (d, J = 4.5 Hz), 129.5 (d, J = 8.2 Hz), 129.4, 128.0, 125.4 (d, J = 14.8 Hz), 124.2 (d, J = 3.8 Hz), 115.3 (d, J = 21.6 Hz), 70.6, 66.4 (d, J = 3.9 Hz), 65.0, 30.7, 27.7, –5.3, –5.4. IR (ATR): 3429, 3071, 2952, 2862, 1718, 1617, 1587, 1490, 1364, 1244 cm^{-1}. HRMS (ESI, m/z): calcd for $C_{19}H_{25}SiO_2Na^+$ $(M+Na)^+$:355.1506; Found: 355.1500.

1-(Dimethyl(phenyl)silyl)-4-((4-fluorobenzyl)oxy)butan-1-ol (1k)

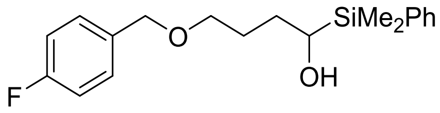

General procedure B: R_f = 0.40 (PE/EA = 4/1, v/v). Colorless oil, (202.5mg, 0.61 mmol, 61% yield). NMR spectroscopy: ^{1}H NMR (400 MHz, $CDCl_3$, 25 °C) δ 7.59–7.55 (m, 2H), 7.40–7.35 (m, 3H), 7.29–7.25 (m, 2H), 7.04–6.99 (m, 2H), 4.46 (s, 2H), 3.52–3.45 (m, 3H), 1.87 (s, 1H), 1.84–1.76 (m, 1H), 1.76–1.64 (m, 2H), 1.58–1.53 (m, 1H), 0.35 (s, 3H), 0.34 (s, 3H); ^{13}C NMR (101 MHz, $CDCl_3$, 25 °C) δ 162.5 (d, J = 245.5 Hz), 134.1 (d, J = 3.4 Hz), 137.0, 134.27, 129.6 (d, J = 8.2 Hz), 129.4, 128.0, 115.4 (d, J = 21.4 Hz), 72.38, 70.4, 65.1, 30.8, 27.8, –5.4, –5.4. IR (ATR): 3444, 3071 2922, 2855, 1722, 1602, 1509, 1155, 1110, 823 cm^{-1}. HRMS (ESI, m/z): calcd for $C_{19}H_{25}SiO_2Na^+$ (M+Na)$^+$: 355.1506; Found: 355.1500.

4-((3-Chlorobenzyl)oxy)-1-(dimethyl(phenyl)silyl)butan-1-ol (1l)

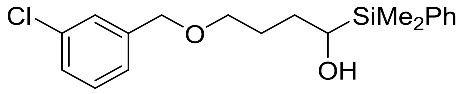

General procedure B: R_f = 0.35 (PE/EA = 4/1, v/v). Colorless oil, (254.0 mg, 0.73 mmol,73% yield). NMR spectroscopy: ^{1}H NMR (400 MHz, $CDCl_3$, 25 °C) δ 7.59–7.55 (m, 2H), 7.40–7.35 (m, 3H), 7.32–7.31 (m, 1H), 7.27– 7.25 (m, 2H), 7.21–7.16 (m, 1H), 4.47 (s, 2H), 3.52–3.46 (m, 3H), 1.89–1.79 (m, 1H), 1.74–1.65 (m, 2H), 1.61–1.51 (m, 1H), 0.35 (s, 3H), 0.34 (s, 3H); ^{13}C NMR (101 MHz, $CDCl_3$, 25 °C) δ 140.5, 137.0, 134.4, 134.3, 129.8, 129.4, 128.0, 127.9, 127.8, 125.7, 72.3, 70.6, 65.2, 30.7, 27.7, –5.3, –5.4. IR (ATR): 3067, 3022, 2922, 2855, 1722, 1699, 1476, 1427, 1356, 1110 cm^{-1}. HRMS (ESI, m/z): calcd for $C_{19}H_{25}SiO_2Na^+$ (M+Na)$^+$: 371.1210; Found: 371.1205.

4-((3-Bromobenzyl)oxy)-1-(dimethyl(phenyl)silyl)butan-1-ol (1m)

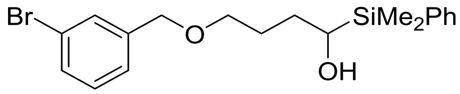

General procedure B: R_f = 0.36 (PE/EA = 4/1, v/v). Yellow oil (229.1 mg, 0.69 mmol, 69% yield). NMR spectroscopy: ^{1}H NMR (400 MHz, $CDCl_3$, 25 °C) δ 7.59–7.55 (m, 2H), 7.48–7.46 (m, 1H), 7.43–7.35 (m, 4H), 7.25–7.17 (m, 2H), 4.46 (s, 2H), 3.53–3.46 (m, 3H), 1.86–1.78 (m, 2H), 1.75–1.65 (m, 2H), 1.61–1.51 (m, 1H), 0.35 (s, 3H), 0.34 (s, 3H); ^{13}C NMR (101 MHz, $CDCl_3$, 25 °C) δ 140.8, 136.9, 134.3, 130.8, 130.7, 130.1, 129.4, 128.0, 126.2, 122.7, 72.2, 70.6, 65.2, 30.7, 27.7, –5.3, –5.4. IR (ATR): 3440, 3067, 2952, 2922, 2855, 1722, 1572, 1427, 1356, 1252 cm^{-1}. HRMS (ESI, m/z): calcd for $C_{19}H_{26}SiBrO_2^+$ (M+H)$^+$: 393.0885; Found: 393.0880.

4-((2-Bromobenzyl)oxy)-1-(dimethyl(phenyl)silyl)butan-1-ol (1n)

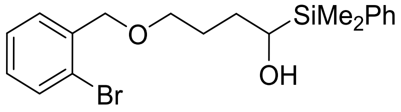

General procedure B: R_f = 0.35 (PE/EA = 4/1, v/v). Yellow oil (278.4 mg, 0.71 mmol, 71% yield). NMR spectroscopy: ^{1}H NMR (400 MHz, $CDCl_3$, 25 °C) δ 7.61–7.51 (m, 3H), 7.45–7.34 (m, 4H),

7.32–7.28 (m, 1H), 7.17–7.12 (m, 1H), 4.57 (s, 2H), 3.58–3.51(m, 3H), 1.93–1.82 (m, 1H), 1.77–1.69 (m, 3H), 1.66–1.54 (m, 1H), 0.35 (s, 3H), 0.34 (s, 3H); ^{13}C NMR (101 MHz, $CDCl_3$, 25 °C) δ 137.7, 137.0, 134.3, 132.7, 129.4, 129.2, 129.0, 128.0, 127.5, 122.9, 72.4, 70.9, 65.2, 30.7, 27.7, –5.3, –5.4. IR (ATR): 3421, 3067, 2922, 2855, 1718, 1591, 1468, 1427, 1356, 1103 cm^{-1}. HRMS (ESI, m/z): calcd for $C_{19}H_{26}SiBrO_2^+$ $(M+H)^+$: 393.0885; Found: 393.0880.

2-((4-(Dimethyl(phenyl)silyl)-4-hydroxybutoxy)methyl)benzonitrile (1o)

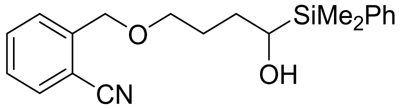

General procedure B: R_f = 0.50 (PE/EA = 1/1, v/v). Yellow oil (271.2 mg, 0.80 mmol, 80% yield). NMR spectroscopy: ^{1}H NMR (400 MHz, $CDCl_3$, 25 °C) δ 7.66–7.63 (m, 1H), 7.60–7.51 (m, 4H), 7.42–7.32 (m, 4H), 4.68 (s, 2H), 3.59 (dd, J = 6.7, 5.4 Hz, 2H), 3.52 (dd, J = 11.1, 3.0 Hz, 1H), 1.90–1.84 (m, 1H), 1.76–1.67 (m, 3H), 1.66–1.53 (m, 1H), 0.35 (s, 3H), 0.34 (s, 3H); ^{13}C NMR (101 MHz, $CDCl_3$, 25 °C) δ 142.2, 136.9, 134.3, 133.0, 132.8, 129.4, 128.7, 128.2, 128.0, 117.4, 111.6, 71.3, 70.6, 65.2, 30.5, 27.5, –5.3, –5.4. IR (ATR): 3444, 3067, 2952, 2225, 1483, 1449, 1364, 1248, 1110, 764 cm^{-1}. HRMS (ESI, m/z): calcd for $C_{20}H_{25}SiNO_2Na^+$ $(M+Na)^+$: 362.1552; Found: 362.1546.

1-(Dimethyl(phenyl)silyl)-6-phenoxyhexan-1-ol (1p)

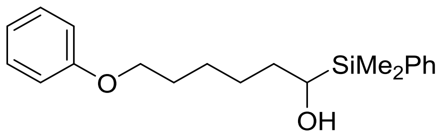

General procedure B: R_f = 0.50 (PE/EA = 4/1, v/v). Yellow oil (233.0 mg, 0.71 mmol, 71% yield). NMR spectroscopy: ^{1}H NMR (400 MHz, $CDCl_3$, 25 °C) δ 7.58–7.55 (m, 2H), 7.39–7.28 (m, 3H), 7.28–7.26 (m. 2H), 6.95–6.88 (m, 3H), 3.96–3.92 (t, 2H), 3.53 (dd, J = 7.6, 6.4 Hz, 1H), 1.81–1.73 (m, 2H), 1.68–1.38 (m, 7H), 0.35 (s, 3H), 0.34 (s, 3H); ^{13}C NMR (101 MHz, $CDCl_3$, 25 °C) δ 159.2, 136.8, 134.2, 129.5, 129.5, 128.1, 120.6, 114.6, 67.8, 65.5, 33.4, 29.4, 26.8, 26.1, –5.3, –5.5. IR (ATR): 3432, 3067, 2929, 2855, 1599, 1494, 1390, 1300, 1244, 1110 cm^{-1}. HRMS (ESI, m/z): calcd for $C_{20}H_{28}SiO_2Na^+$ $(M+Na)^+$: 351.1756; Found: 351.1751.

6-(Dimethyl(phenyl)silyl)-6-hydroxyhexyl benzoate (1q)

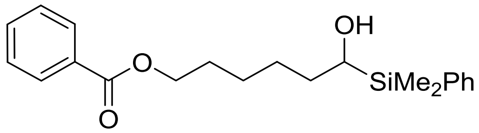

General procedure B: R_f = 0.52 (PE/EA = 4/1, v/v). Colorless oil (199.5 mg, 0.56 mmol, 56% yield). NMR spectroscopy: ^{1}H NMR (400 MHz, $CDCl_3$, 25 °C) δ 8.06–8.02 (m, 2H), 7.60–7.52 (m, 3H), 7.47–7.41 (m, 2H), 7.40–7.34 (m, 3H), 4.30 (t, J = 6.7 Hz, 2H), 3.56–3.46 (m, 1H), 1.77–1.72 (m, 2H), 1.68–1.60 (m, 1H), 1.59–1.51 (m, 2H), 1.50–1.30 (m, 4H), 0.34 (s, 3H), 0.33 (s, 3H); ^{13}C NMR (101 MHz, $CDCl_3$, 25 °C) δ 166.8, 136.8, 134.2, 133.0, 130.6, 129.7, 129.5, 128.5, 128.1, 65.5, 65.1, 33.4, 28.9, 26.7, 26.1, -5.3, -5.5. IR (ATR): 3444, 3067, 2929, 2855, 1718, 1453, 1386, 1315, 1274, 1110 cm^{-1}. HRMS (ESI, m/z): calcd for $C_{21}H_{28}SiO_3Na^+$ $(M+Na)^+$: 379.1705; Found: 379.1700.

6-(Benzyloxy)-1-(dimethyl(phenyl)silyl)hexan-1-ol (1r)

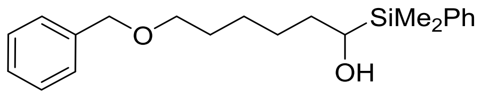

General procedure B: R_f = 0.48 (PE/EA = 4/1, v/v). Colorless oil (287.4, 0.84 mmol, 84% yield). NMR spectroscopy: ^{1}H NMR (400 MHz, $CDCl_3$, 25 ºC) δ 7.59–7.54 (m, 2H), 7.40–7.36 (m, 3H), 7.36–7.26 (m, 5H), 4.49 (s, 2H), 3.53–3.48 (m, 1H), 3.45 (t, J = 6.6 Hz, 2H), 1.65–1.50 (m, 5H), 1.42–1.29 (m, 4H), 0.34 (s, 3H), 0.33 (s, 3H); ^{13}C NMR (101 MHz, $CDCl_3$, 25 ºC) δ 138.8, 136.9, 134.2, 129.4, 128.5, 128.0, 127.8, 127.6, 73.0, 70.5, 65.5, 33.4, 29.8, 26.8, 26.2, -5.2, -5.5. IR (ATR) 3436, 3049, 2922, 2855, 1513, 1457, 1360, 1248, 1099, 812 cm^{-1}. HRMS (ESI, m/z): calcd for $C_{21}H_{30}SiO_2Na^+$ $(M+Na)^+$: 365.1916; Found: 365.1907.

1-(Dimethyl(phenyl)silyl)-4-(3-phenylpropoxy)butan-1-ol (1s)

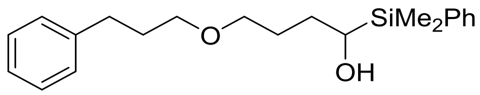

General procedure B: R_f = 0.48 (PE/EA = 4/1, v/v). Colorless oil (205.2 mg, 0.60 mmol, 60% yield). NMR spectroscopy: ^{1}H NMR (400 MHz, $CDCl_3$, 25 ºC) δ 7.63–7.56 (m, 2H), 7.40–7.33 (m, 3H), 7.31–7.25 (m, 2H), 7.22–7.15 (m, 3H), 3.53–3.49 (m, 1H), 3.47–3.39 (m, 4H), 2.73–2.63 (m, 2H), 2.10 (s, 1H), 1.94–1.85 (m, 2H), 1.80–1.65 (m, 3H), 1.62–1.49 (m, 1H), 0.36 (s, 3H), 0.35 (s, 3H); ^{13}C NMR (101 MHz, $CDCl_3$, 25 ºC) δ 142.0, 137.2, 134.3, 129.3, 128.6, 128.5, 128.0, 125.9, 71.0, 70.2, 65.1, 32.4, 31.3, 31.1, 28.2, -5.3, -5.4. IR (ATR): 3421, 3067, 2922, 2855, 1494, 1248, 1110, 1043, 812, 700 cm^{-1}. HRMS (ESI, m/z): calcd for $C_{21}H_{30}SiO_2Na^+$ $(M+Na)^+$: 365.1913; Found: 365.1907.

1-(Dimethyl(phenyl)silyl)-6-((4-methylbenzyl)oxy)hexan-1-ol (1t)

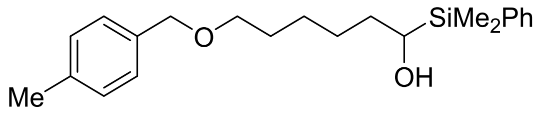

General procedure B: R_f = 0.45 (PE/EA = 4/1, v/v). Colorless oil (303.0, 0.86 mmol, 86% yield). NMR spectroscopy: ^{1}H NMR (400 MHz, $CDCl_3$, 25 ºC) δ 7.59–7.53 (m, 2H), 7.42–7.34 (m, 3H), 7.23–7.14 (m, 4H), 4.45 (s, 2H), 3.53–3.48 (m, 1H), 3.43 (t, J = 6.6 Hz, 2H), 2.35 (s, 3H), 1.65–1.49 (m, 5H), 1.43–1.26 (m, 4H), 0.34 (s, 3H), 0.33 (s, 3H); ^{13}C NMR (101 MHz, $CDCl_3$, 25 ºC) δ 137.3, 136.9, 135.7, 134.2, 129.4, 129.2, 128.0, 127.9, 72.9, 70.3, 65.5, 33.4, 29.8, 26.8, 26.2, 21.3, -5.2, -5.5. IR (ATR): 3436, 3049, 2922, 2855, 1513, 1457, 1360, 1248, 1099, 812 cm^{-1}. HRMS (ESI, m/z): calcd for $C_{22}H_{32}SiO_2Na^+$ $(M+Na)^+$: 379.2069; Found: 379.2064.

5-(Benzyloxy)-1-(dimethyl(phenyl)silyl)pentan-1-ol (1u)

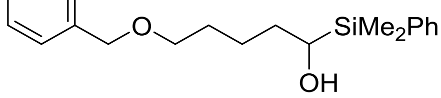

General procedure B: R_f = 0.40 (PE/EA = 4/1, v/v). Yellow oil (269.0 mg, 0.82 mmol, 82% yield). NMR spectroscopy: ^{1}H NMR (400 MHz, $CDCl_3$, 25 ºC) δ 7.57–7.55 (m, 2H), 7.41–7.36 (m, 3H), 7.36–7.26 (m, 5H), 4.49 (s, 2H), 3.54–3.49 (m, 1H), 3.47–3.44 (m, 2H), 1.69–1.61 (m, 2H), 1.60–1.51

(m, 3H), 1.43–1.37 (m, 2H), 0.34 (s, 3H), 0.33 (s, 3H); ^{13}C NMR (101 MHz, CDCl$_3$) δ 138.7, 136.8, 134.2, 129.4, 128.5, 128.0, 127.8, 127.6, 73.0, 70.4, 65.4, 33.2, 29.6, 23.6, −5.3, −5.5. IR (ATR): 3440, 3067, 2933, 2855, 1722, 1453, 1427, 1364, 1248, 1110 cm^{-1}. HRMS (ESI, m/z): calcd for $C_{21}H_{32}SiO_3Na^+$ $(M+Na)^+$: 351.1756; Found: 351.1751.

1-(Dimethyl(phenyl)silyl)-4-(isopentyloxy)butan-1-ol(1v)

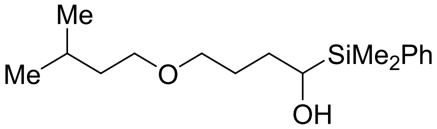

General procedure A: R$_f$ = 0.52 (PE/EA = 5/1, v/v). Colorless oil (132.3 mg, 2.28 mmol, 45% yield). NMR spectroscopy: ^{1}H NMR (400 MHz, CDCl$_3$, 25 °C) δ 7.59–7.55 (m, 2H), 7.39–7.34 (m, 3H), 3.51–3.40 (m, 5H), 1.79–1.61 (m, 5H), 1.59–1.51 (m, 1H), 1.49–1.43 (m, 2H), 0.90 (s, 3H), 0.88 (s, 3H), 0.34 (s, 3H), 0.33 (s, 3H); ^{13}C NMR (101 MHz, CDCl$_3$) δ 137.3, 134.3, 129.3, 127.9, 71.0, 69.7, 65.1, 38.5, 31.2, 28.2, 25.2, 22.8, 22.7, -5.3, -5.4. IR (ATR): 3421, 2955, 2866, 1464, 1427, 1367, 1249, 1110, 812, 700 cm^{-1}. HRMS (ESI, m/z): calcd for $C_{17}H_{30}SiO_2Na^+$ $(M+Na)^+$: 317.1913; Found: 317.1907.

1-(Dimethyl(phenyl)silyl)-6-((4-(trifluoromethoxy)benzyl)oxy)hexan-1-ol (1w)

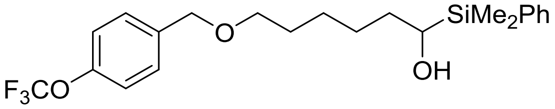

General procedure B: R$_f$ = 0.45 (PE/EA = 4/1, v/v). Colorless oil (287.4, 0.84 mmol, 84% yield). NMR spectroscopy: ^{1}H NMR (400 MHz, CDCl$_3$, 25 °C) 7.57–7.55 (m, 2H), 7.39–7.34 (m, 5H), 7.29–7.17 (m, 2H), 4.47 (s, 2H), 3.52–3.44 (m, 3H), 1.62–1.52 (m, 5H), 1.36–1.31 (m, 4H), 0.34 (s, 3H), 0.33 (s, 3H); ^{13}C NMR (101 MHz, CDCl$_3$, 25 °C) δ 148.7 (q, J = 2.1 Hz), 137.6, 136.8, 134.3, 129.4, 129.0, 128.1, 120.63 (q, J = 256.6 Hz), 121.0, 72.1, 70.8, 65. 6, 33.4, 29.8, 26.8, 26.2, -5.3, -5.5. IR (ATR): 3049, 2933, 2858, 1720, 1509, 1427, 1364, 1256, 1162, 1118 cm^{-1}. HRMS (ESI, m/z): calcd for $C_{22}H_{29}SiF_3O_3Na^+$ $(M+Na)^+$:449.1736; Found: 449.1730.

4-(2-(Benzyloxy)ethoxy)-1-(dimethyl(phenyl)silyl)butan-1-ol (1x)

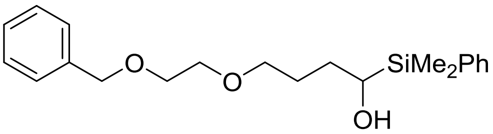

General procedure B: R$_f$ = 0.50 (PE/EA = 3/1, v/v). Yellow oil (251.2 mg, 0.80 mmol, 80% yield). NMR spectroscopy: ^{1}H NMR (400 MHz, CDCl$_3$, 25 °C) δ 7.60–7.55 (m, 2H), 7.37–7.27 (m, 8H), 4.56 (s, 2H), 3.61 (s, 4H), 3.54–3.48 (m, 3H), 2.07 (s, 1H), 1.80–1.65 (m, 3H), 1.62–1.51 (m, 1H), 0.35 (s, 3H), 0.34 (s, 3H); ^{13}C NMR (101 MHz, CDCl$_3$, 25 °C) δ 138.3, 137.2, 134.3, 129.3, 128.5, 128.0, 127.9, 127.7, 73.4, 71.6, 70.3, 69.4, 65.1, 31.0, 28.0, –5.3, –5.4. IR (ATR): 3436, 3067, 2903, 2858, 1722, 1453, 1353, 1249, 1110, 812 cm^{-1}. HRMS (ESI, m/z): calcd for $C_{21}H_{32}SiO_3Na^+$ $(M+Na)^+$: 381.1862; Found: 381.1856.

6-Chloro-1-(dimethyl(phenyl)silyl)hexan-1-ol (1y)

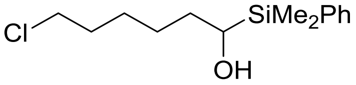

General procedure A: R_f = 0.50 (PE/EA = 4/1, v/v). Yellow oil, (743.0 mg, 2.75 mmol, 68% yield). NMR spectroscopy: ^{1}H NMR (400 MHz, $CDCl_3$, 25 °C) δ 7.58–7.54 (m, 2H), 7.40–7.36 (m, 3H), 3.53-3.49 (m, 3H), 1.80–1.70 (m, 2H), 1.60–1.49 (m, 3H), 1.49–1.28 (m, 4H), 0.34 (s, 3H), 0.34 (s, 3H); ^{13}C NMR (101 MHz, $CDCl_3$) δ 136.7, 134.2, 129.5, 128.1, 65.5, 45.2, 33.3, 32.7, 26.8, 26.3, -5.3, -5.6. IR (ATR): 3440, 2929, 2855, 1714, 1461, 1252, 1114, 1043, 831, 700 cm^{-1}. HRMS (ESI, m/z): calcd for $C_{14}H_{23}ClSiONa^+$ $(M+Na)^+$: 293.1104; Found: 293.1099.

3-(Dimethyl(phenyl)silyl)heptan-3-ol (1z)

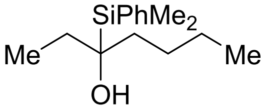

General procedure A: R_f = 0.75 (PE/EA = 10/1, v/v). yellow oil, (1.62 g, 6.4 mmol, 32% yield) NMR spectroscopy: ^{1}H NMR (400 MHz, $CDCl_3$, 25 °C) δ 7.61–7.57 (m, 2H), 7.39–7.34 (m, 3H), 1.63–1.48 (m,4H), 1.32–1.17 (m, 4H), 0.89–0.81 (m, 6H), 0.38 (s, 6H); ^{13}C NMR (151 MHz, $CDCl_3$, 25 °C) δ 137.4, 134.7, 129.3, 127.9, 69.3, 36.3, 29.4, 25.6, 23.6, 14.2, 7.7, -4.3, -4.3. IR (ATR): 3474, 3071, 3049, 2960, 2870, 1461, 1428, 1111, 813, 734 cm^{-1}. HRMS (ESI, m/z): calcd for $C_{15}H_{26}SiONa^+$ $(M+Na)^+$: 273.1645; Found: 273.1637.

2-(dimethyl(phenyl)silyl)-5-methylhexan-2-ol(1af)

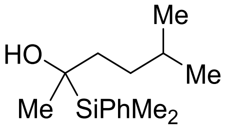

General procedure C: R_f = 0.75 (PE/EA = 10/1, v/v). yellow oil, (333 mg, 1.33 mmol, 67% yield). ^{1}H NMR (400 MHz, $CDCl_3$, 25 °C) δ 7.60–7.57 (m, 2H), 7.39–7.37 (m, 3H), 1.55–1.38 (m, 3H), 1.26–1.10 (m, 5H), 0.85 (dd, J = 6.6, 4.6 Hz, 6H), 0.36 (s, 6H). ^{13}C NMR (151 MHz, $CDCl_3$, 25 °C) δ 136.9, 134.7, 129.4, 127.9, 66.4, 37.7, 31.8, 28.8, 24.1, 22.9, 22.7, -5.2, -5.3. IR (ATR): 3444, 2952, 2870, 1759, 1465, 1427, 1249, 1111, 857, 775 cm-1. HRMS (ESI, m/z): calcd for $C_{15}H_{26}OSiNa^+$ $(M+Na)^+$: 273.1645; Found:273.1638.

2-(dimethyl(phenyl)silyl)hexan-2-ol(1ag)

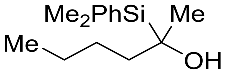

General procedure C: R_f = 0.75 (PE/EA = 10/1, v/v). yellow oil, (281 mg, 1.20 mmol, 60% yield). ^{1}H NMR (400 MHz, $CDCl_3$, 25 °C) δ 7.60–7.57 (m, 2H), 7.39–7.37 (m, 3H), 1.56–1.43 (m, 2H), 1.33–1.23 (m, 4H), 1.18 (s, 3H), 0.88 (t, J = 6.3 Hz, 3H), 0.36 (s, 6H) ; ^{13}C NMR (151 MHz, $CDCl_3$, 25 °C) δ 136.9, 134.6, 129.3, 127.9, 66.4, 39.5, 24.9, 24.0, 23.5, 14.2, -5.4, -5.4. IR (ATR): 3440, 2955, 2862, 1763, 1461, 1428, 1249, 1111, 813, 738 cm^{-1}. HRMS (ESI, m/z): calcd for $C_{14}H_{24}OSiNa^+$ $(M+Na)^+$: 259.1489; Found: 259.1481

5-Azido-1-(dimethyl(phenyl)silyl)pentan-1-ol (1e)

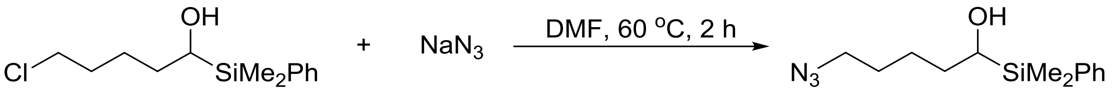

1e

To a solution of 5-chloro-1-(dimethyl(phenyl)silyl)pentan-1-ol (405 g, 1.5 mmol) in DMF (20 mL) was added NaN_3 (486.5 mg, 7.5 mmol). After being stirred at 60 °C for 2 h. The mixture was cooled to room temperature then diluted with ethyl acetate (50 mL) and washed with water (50×3 mL). The organic phase was dried over (Na_2SO_4) and the solvents were removed under reduced pressure to afford the crude product. The product was purified by column chromatography on silica gel using PE/EA (10:1, v/v) as eluent to afford **1e**.

R_f = 0.42 (PE/EA = 4/1, v/v). Yellow oil, (232.3 mg, 0.9 mmol, 58% yield). NMR spectroscopy: ^{1}H NMR (400 MHz, $CDCl_3$, 25 °C) δ 7.59–7.54 (m, 2H), 7.42–7.36 (m, 3H), 3.53–3.47 (m, 1H), 3.24 (t, J = 6.7 Hz, 2H), 1.66–1.59 (m, 2H), 1.59–1.50 (m, 3H), 1.42–1.34 (m, 1H), 0.35 (s, 3H), 0.34 (s, 3H); ^{13}C NMR (101 MHz, $CDCl_3$, 25 °C) δ 136.6, 134.2, 129.6, 128.1, 65.4, 51.5, 33.0, 28.8, 24.2, –5.3, –5.6. IR (ATR): 3425, 2917, 2091, 1453, 1427, 1110, 1028, 879, 812, 700 cm^{-1}. HRMS (ESI, m/z): calcd for $C_{13}H_{21}SiONa^+$ $(M+Na)^+$: 286.1352; Found: 286.1346.

6-Bromo-1-(dimethyl(phenyl)silyl)hexan-1-ol (1f)

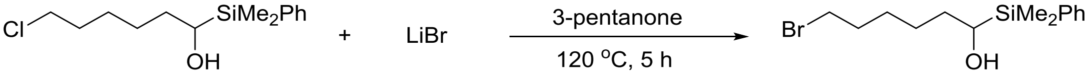

1y **1f**

To a solution of **1y** (920 mg, 3.4 mmol) in 3-pentanone (34 mL) was added lithium bromide (2.9 g, 34 mmol, 10.0 equiv.) and the mixture was allowed to refluxed for 2 h at 120 °C. The reaction mixture was then cooled to room temperature and the solvents were removed under reduced pressure. The residue was dissolved in ethyl acetate (30 mL) and washed with water (30×3 mL). The organic phase was dried over (Na_2SO_4) and the solvents were evaporated under reduced pressure afforded the mixture of **1y** and **1f** which was once more treated with lithium bromide (2.9 g, 34 mmol, 10.0 equiv.) in 30 mL of 3-pentanone. The mixture was subsequently refluxed for 3 h at 120 °C. The reaction mixture was then cooled to room temperature and the solvents were removed under reduced pressure. The resulting residue was dissolved in ethyl acetate (30 mL) and washed with water (30 X 3 mL). The organic phase was dried over (Na_2SO_4) and the solvents were evaporated under reduced pressure to afford **1f** which was purified by column chromatography on silica gel using PE/EA (10:1, v/v) as eluent to afford desired product. [8]

R_f = 0.52 (PE/EA = 4/1, v/v). Yellow oil, (715.5 mg, 2.28 mmol, 67% yield). NMR spectroscopy: ^{1}H NMR (400 MHz, $CDCl_3$, 25 °C) δ 7.57–7.55 (m, 2H), 7.40–7.36 (m, 3H), 3.50 (dd, J = 7.3, 6.5 Hz, 1H), 3.39 (t, J = 6.8 Hz, 2H), 1.97–1.74 (m, 2H), 1.66–1.26 (m, 7H), 0.35 (s, 3H), 0.34 (s, 3H); ^{13}C NMR (101 MHz, $CDCl_3$, 25 °C) δ 136.7, 134.2, 129.5, 128.1, 65.5, 34.0, 33.3, 32.8, 28.1, 26.2, –5.3, –5.5. IR (ATR): 2922, 2851, 1461, 1427, 1248, 1110, 812, 775, 734, 700 cm^{-1}. HRMS (ESI, m/z): calcd for $C_{14}H_{23}BrSiONa^+$ $(M+Na)^+$: 337.0599; Found: 337.0594.

1-(Tert-butyldimethylsilyl)heptan-1-ol (1ac)

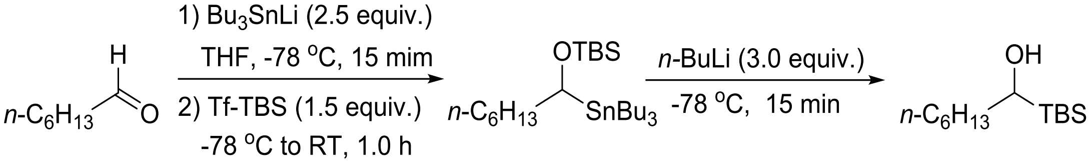

THF solution of the stannyl anion (0.95 equiv, 0.1 M concentration) was cooled to −78 ℃ and the aldehyde (20 mmol, 2.28g) was added dropwise. The reaction mixture was stirred at −78 ℃ for 15 min and then quenched at −78 ℃ by the dropwise addition of the desired trialkylsilyl cyanide or triflate (30 mmol, 7.92g). The mixture was allowed to gradually warm to room temperature and then stirred for 1 h. The solution was diluted with 100 mL of petroleum ether, washed with 20 mL of saturated queous sodium chloride solution, and dried over anhydrous sodium sulfate. The solvents were removed under reduced pressure to provide the crude stannyl silyl ether. Then the crude stannyl silyl ether was dissolved in 25 mL of dry THF and cooled to −78 ℃ (under Ar). A hexane solution of n-butyllithium (3.0 equiv.) was added dropwise via syringe. The reaction mixture was then allowed to stir at −78 ℃ for 15 min and quenched at −78 ℃ by the rapid addition of 5 mL of water. After warming to room temperature, the reaction mixture was worked up by extraction using petroleum ether. The 1-(Tert-butyldimethylsilyl)heptan-1-ol product was purified with column chromatography on silica gel (200–300 mesh) with PE as eluent to afford 2.0 g of the title compound as a colourless oil (43% yield).

R_f = 0.65 (PE/EA = 10/1, v/v). NMR Spectroscopy: ^{1}H NMR (600 MHz, $CDCl_3$, 25 °C) δ 3.46 (m, 1H), 1.58–1.49 (m, 2H), 1.34–1.26 (m, 8H), 0.93 (s, 9H), 0.89–0.87 (m, 3H), 0.00 (s, 3H), –0.06 (s, 3H) ; ^{13}C NMR (151 MHz, $CDCl_3$, 25 °C) δ 64.6, 34.5, 32.0, 29.4, 27.2, 27.0, 22.8, 16.9, 14.2, -7.4, -8.5. IR (ATR): 3463, 2956, 2926, 2855, 1465, 1364, 1249, 1006, 828, 772 cm^{-1}. HRMS (ESI, m/z): calcd for $C_{13}H_{30}O$ SiK^+ $(M+K)^+$: 269.1703; Found: 269.1700.

1-(Triphenylsilyl)heptan-1-ol (1ae)

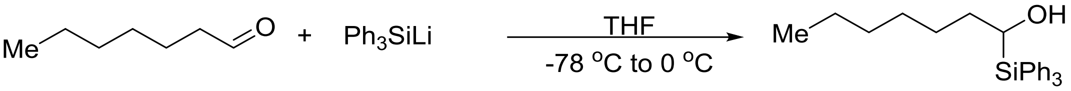

A solution of Ph_3SiLi (30 mmol, 1.5 equiv.) in THF (30 mL) was added dropwise to a stirred solution of the heptanal (15 mmol, 1.0 equiv.) in THF (15 mL) via cannula at −78 ℃. The reaction mixture was allowed to warm to 0 °C slowly before being poured into saturated aqueous NH_4Cl and extracted with ethyl acetate (3×100 mL). The combined extracts were dried (Na_2SO_4) and the solvent was removed under reduced pressure to afford the crude product, which was purified by column chromatography on silica gel using PE/EA (20:1, v/v) as eluent to afford **1ae**.

R_f = 0.55 (PE/EA = 20/1, v/v). Yellow oil, (2.40 g, 8.25 mmol, 55% yield). NMR spectroscopy: ^{1}H NMR (400 MHz, $CDCl_3$, 25 °C) δ 7.63–7.60 (m, 6H), 7.49–7.34 (m, 9H), 4.32–4.01 (m, 1H), 1.79–1.66 (m, 2H), 1.64 (s, 1H), 1.41–1.10 (m, 8H), 0.85 (t, J = 6.9 Hz, 3H); ^{13}C NMR (151 MHz, $CDCl_3$, 25 °C) δ 136.3, 133.3, 129.9, 128.2, 64.8, 33.8, 32.0, 29.2, 27.2, 22.8, 14.2. IR (ATR): 3440, 3071, 2929, 2855, 1427, 1107, 1028, 868, 700 cm^{-1}. HRMS (ESI, m/z): calcd for $C_{25}H_{30}OSiNa^+$ $(M+Na)^+$: 397.1956; Found: 397.1958.

5-((Tert-butyldimethylsilyl)oxy)-1-(dimethyl(phenyl)silyl)pentan-1-ol (1a-3)

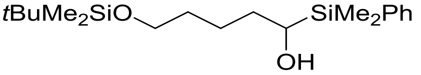

Prepared following General procedure A. R_f = 0.60 (EA=100%). Yellow oil, (1.0 g, 2.93 mmol, 43% yield). NMR spectroscopy: ^{1}H NMR (400 MHz, $CDCl_3$, 25 °C) δ 7.58–7.55 (m, 2H), 7.41–7.36 (m, 3H), 3.62–3.56 (m, 2H), 3.51 (dd, 1H), 1.61–1.45 (m, 5H), 1.39-1.2 (m, 2H), 0.89 (s, 9H), 0.34 (s, 3H), 0.33 (s, 3H), 0.03 (s, 6H); ^{13}C NMR (101 MHz, $CDCl_3$, 25 °C) δ 136.9, 134.3, 129.4, 128.0, 65.5, 63.3, 33.2, 32.6, 26.1, 23.2, 18.5, –5.2, –5.2, –5.2, –5.5. IR (ATR): 2952, 2855, 1472, 1427, 1360, 1252, 1095, 834, 775, 700 cm^{-1}. HRMS (ESI, m/z): calcd for $C_{19}H_{36}Si_2O_2Na^+$ $(M+Na)^+$:372.2152; Found: 375.2146.

1-(Dimethyl(phenyl)silyl)pentane-1,5-diol (1a-6)

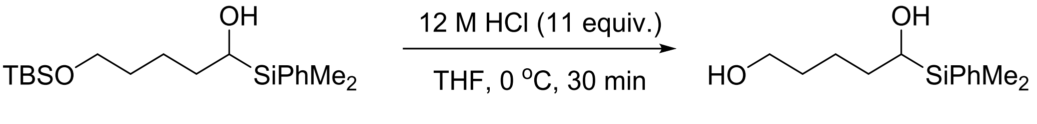

1a-3 **1a-6**

1a-3 (1.5 mmol, 528 mg) was dissolved in THF (18 mL) and the mixture was cooled to 0 °C. Then 12 M HCl (1.4 mL) was added dropwise, and the mixture was Stir at 0°C. After 30 min, Saturated sodium bicarbonate solution was added and extracted with EA (3×20 mL), and the combined organic layers were dried over Na_2SO_4, filtered, and the solvent was removed in vacuo. The **1a-6** product was purified with column chromatography on silica gel (200–300 mesh) with PE/EA (2/1, v/v) as eluent to afford 318 mg of the title compound as a colourless oil (89% yield).

R_f = 0.44 (PE/EA = 10/1, v/v). NMR Spectroscopy: ^{1}H NMR (600 MHz, $CDCl_3$, 25 □) δ 7.56–7.55 (m, 2H), 7.39–7.35 (m, 3H), 3.59 (td, J = 6.3, 1.5 Hz, 2H), 3.50 (dd, J = 10.1, 4.0 Hz, 1H), 1.94 (s, 1H), 1.66–1.46 (m, 6H), 0.34 (s, 3H), 0.33 (s, 3H); ^{13}C NMR (151 MHz, $CDCl_3$, 25 □) δ 136.8, 134.2, 129.5, 128.0, 65.3, 62.7, 32.9, 32.3, 23.1, -5.4, -5.5. IR (ATR): 3325, 2926, 2855, 1666, 1427, 1249, 1110, 1043, 913, 813 cm^{-1}. HRMS (ESI, m/z): calcd for $C_{13}H_{22}O_2SiNa^+$ $(M+Na)^+$: 261.1279; Found: 261.1281.

7. Synthesis of isoquinoline 4e

5-(4-Chlorophenyl)isoquinoline (4e)

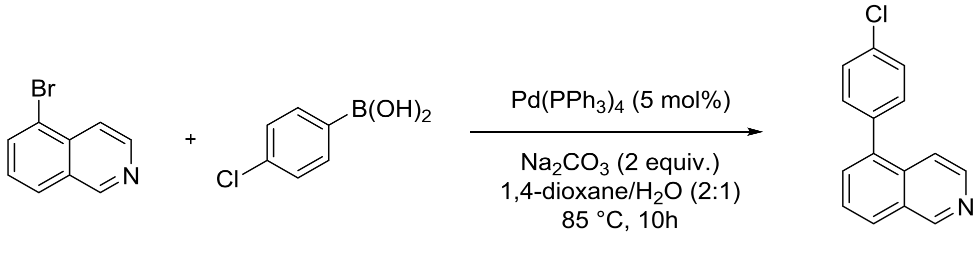

4e

A mixture of 5-bromoisoquinoline (1.04 g, 5.0 mmol, 1.0 equiv.), (4-chlorophenyl)boronic acid (1.17g, 7.5 mmol, 1.5 equiv.), $Pd(PPh_3)_4$ (288.89 mg, 0.25 mmol, 5 mmol%), and Na_2CO_3 (1.06 g, 10.0 mmol, 2.0 equiv.) in 1,4-dioxane (20 mL) and water (10 mL) was stirred at 85 °C for 10 h. The resulting mixture was diluted with water (30 mL) and then extracted three with EtOAc (60 mL). The combined organic layer was dried with Na_2SO_4 and then evaporated under reduced pressure. The crude product was purified with column chromatography on silica gel (200–300 mesh) with PE/EA (8/1, v/v) as eluent to afford 1.09 g of the title compound as yellow solid (91% yield).

R_f = 0.28 (PE/EA = 4/1, v/v), mp: 59 °C–63 °C. NMR Spectroscopy: ^{1}H NMR (400 MHz, $CDCl_3$, 25 □)

δ 9.31 (s, 1H), 8.49 (d, J = 6.0 Hz, 1H), 8.03–7.96 (m, 1H), 7.70–7.61 (m, 3H), 7.49 (d, J = 8.4 Hz, 2H), 7.44–7.38 (m, 2H); ^{13}C NMR (101 MHz, $CDCl_3$, 25 □) δ 153.1, 143.7, 138.0, 137.5, 134.08, 134.05, 131.3, 131.0, 129.0, 128.9, 127.7, 126.9, 118.3; IR (ATR): 3403, 2922, 2851, 1569, 1498, 1364, 1271, 1080, 928, 760 cm^{-1}. HRMS (ESI, m/z): calcd for $C_{15}H_{11}ClN^+$ $(M+H)^+$: 240.0575; Found: 240.0567.

8. Supplementary References

[1] Frisch, M. J. Gaussian 09, Revision D.01, Gaussian, Inc., Wallingford, CT, (2013). The full author list is shown in the ESI 3.j).

[2] Zhao, Y. & Truhlar, D. G. The M06 suite of density functionals for main group thermochemistry, thermochemical kinetics, noncovalent interactions, excited states, and transition elements: two new functionals and systematic testing of four M06-class functionals and 12 other functionals, *Theor. Chem. Account.*, **120**, 215 (2007).

[3] Hellweg, A., Hättig, C. & Höfener, S. et al. Optimized accurate auxiliary basis sets for RI-MP2 and RI-CC2 calculations for the atoms Rb to Rn, *Theoritical Chemistry Accounts* **117**: 587–597 (2007).

[4] Yi, H., Mao, W. & Oestreich, M. Enantioselective Construction of alpha-Chiral Silanes by Nickel-Catalyzed C(sp^3)-C(sp^3) Cross-Coupling. *Angew. Chem. Int. Ed.* **58**, 3575–3578 (2019).

[5] Kleeberg, C., Feldmann, E., Hartmann, E., Vyas, D. J. & Oestreich, M. Copper-Catalyzed 1,2-Addition of Nucleophilic Silicon to Aldehydes: Mechanistic Insight and Catalytic Systems. *Chem. Eur. J.* **17**, 13538–13543 (2011).

[6] Srihari, P., Kumaraswamy, B., Somaiah, R. & Yadav, J. The Stereoselective Total Synthesis of (+)-Stagonolide B. *Synthesis* **2010**, 1039–1045 (2010).

[7] Prabhakar, P., Rajaram, S., Reddy, D. K., Shekar, V. & Venkateswarlu, Y. Total Synthesis of the Phytotoxic Stagonolides A and B. *Tetrahedron: Asym.* **21**, 216–221 (2010).

[8] Li, X., Singh, S. M. & Labrie, F. A Convenient and Practical Method for Conversion of Primary Alkyl Chlorides to Highly Pure Bromides (⩾99%). *Syn. Comm.* **24**, 733–743 (1994).

9. NMR Spectra of the Products

N-(Benzyloxy)-2-hydroxyoctanimidoyl cyanide(3a)

^{1}H NMR of **3a** ($CDCl_3$, 400 MHz, 25 ºC)

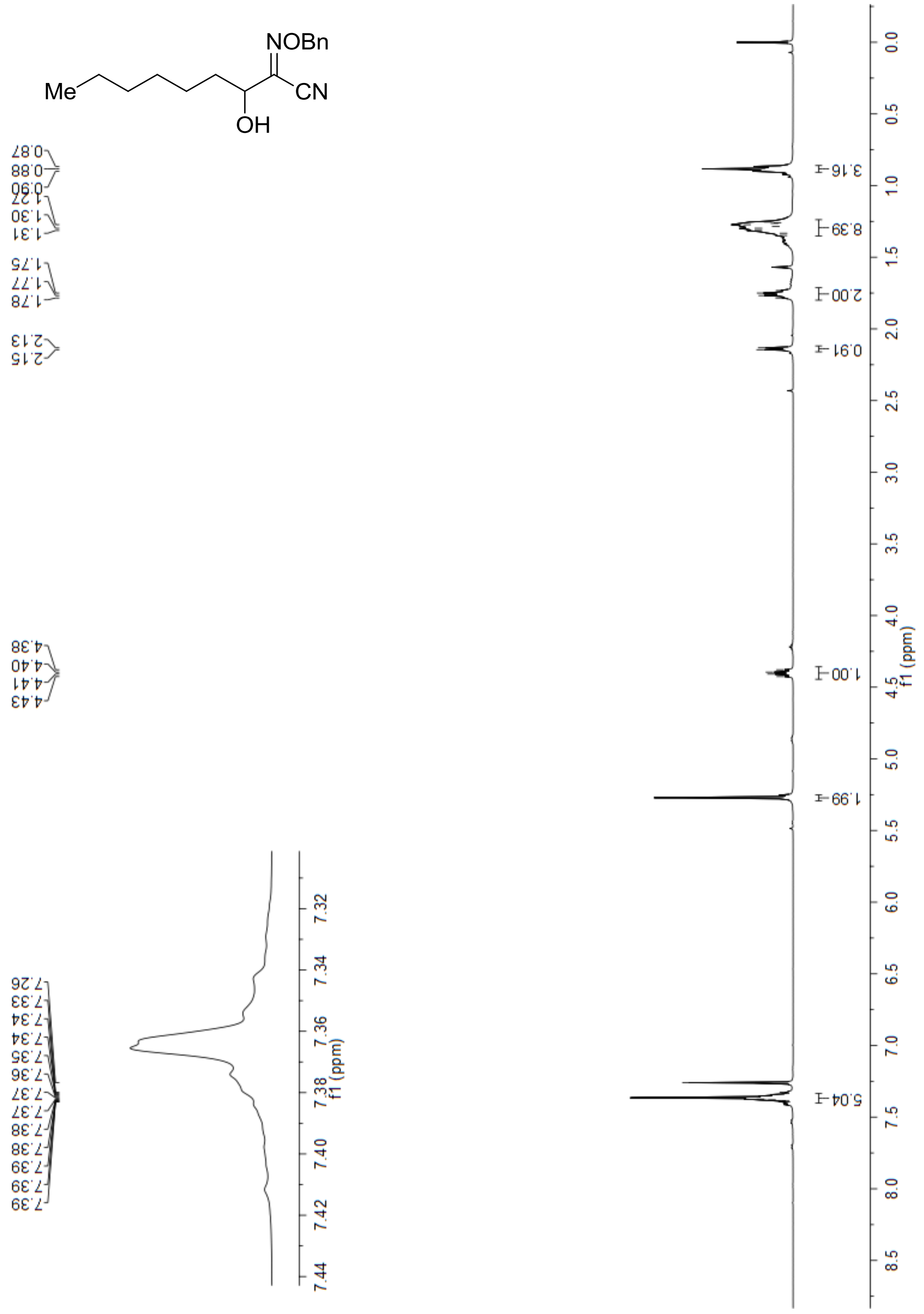

^{13}C NMR of **3a** ($CDCl_3$, 101 MHz, 25 ºC)

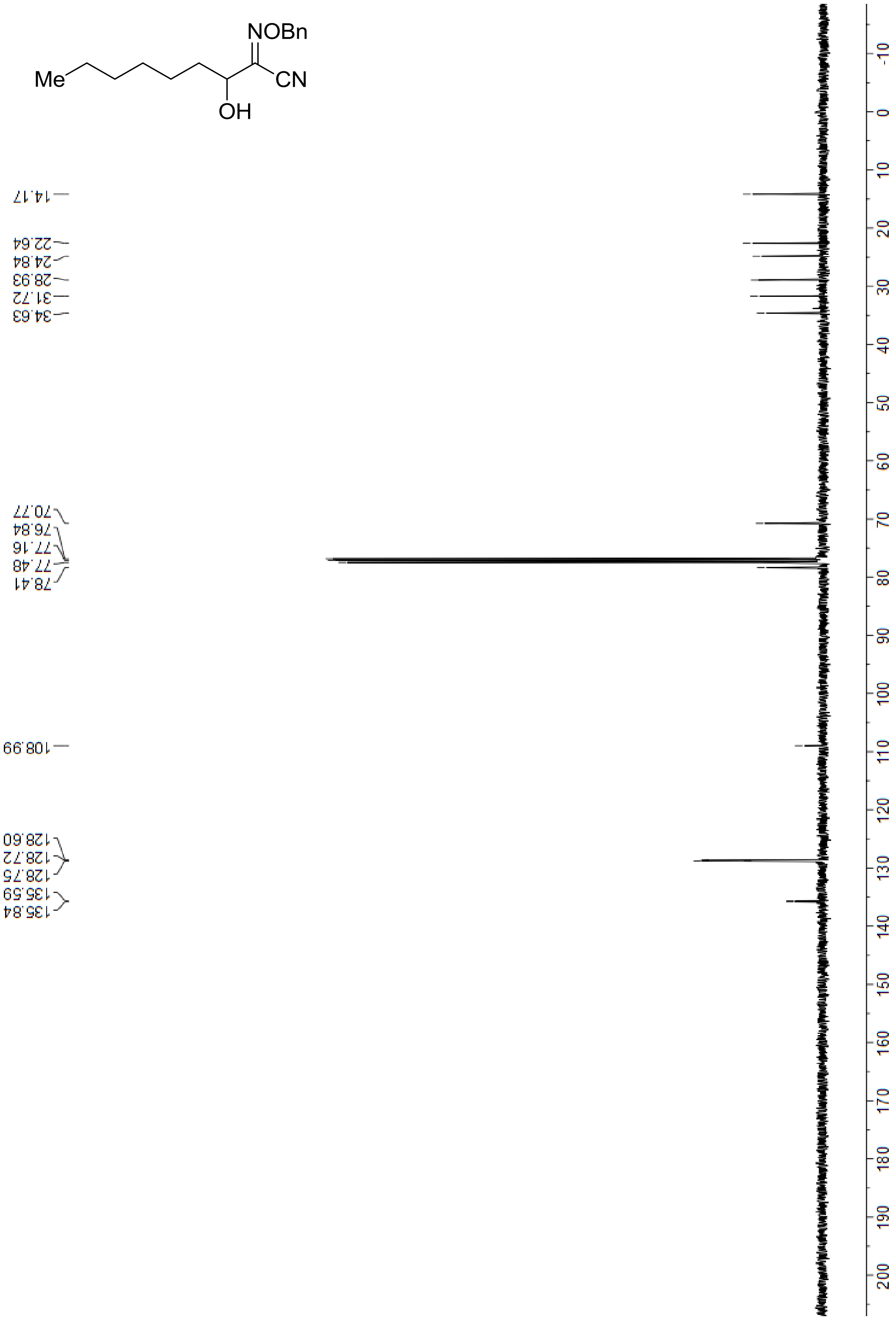

N-(Benzyloxy)-2-hydroxyundecanimidoyl cyanide(3b)

^{1}H NMR of **3b** ($CDCl_3$, 400 MHz, 25 ºC)

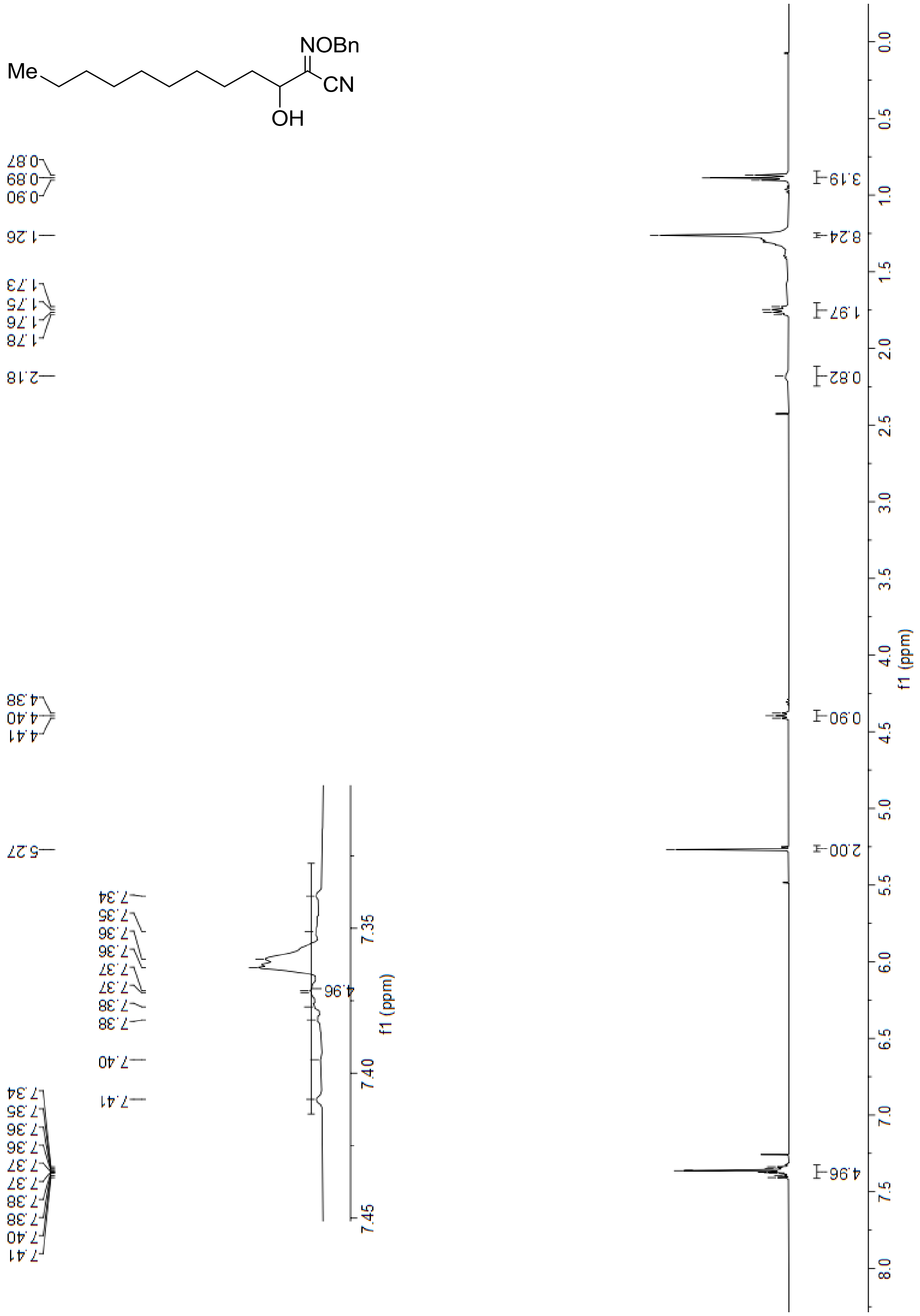

^{13}C NMR of **3b** ($CDCl_3$, 101 MHz, 25 ºC)

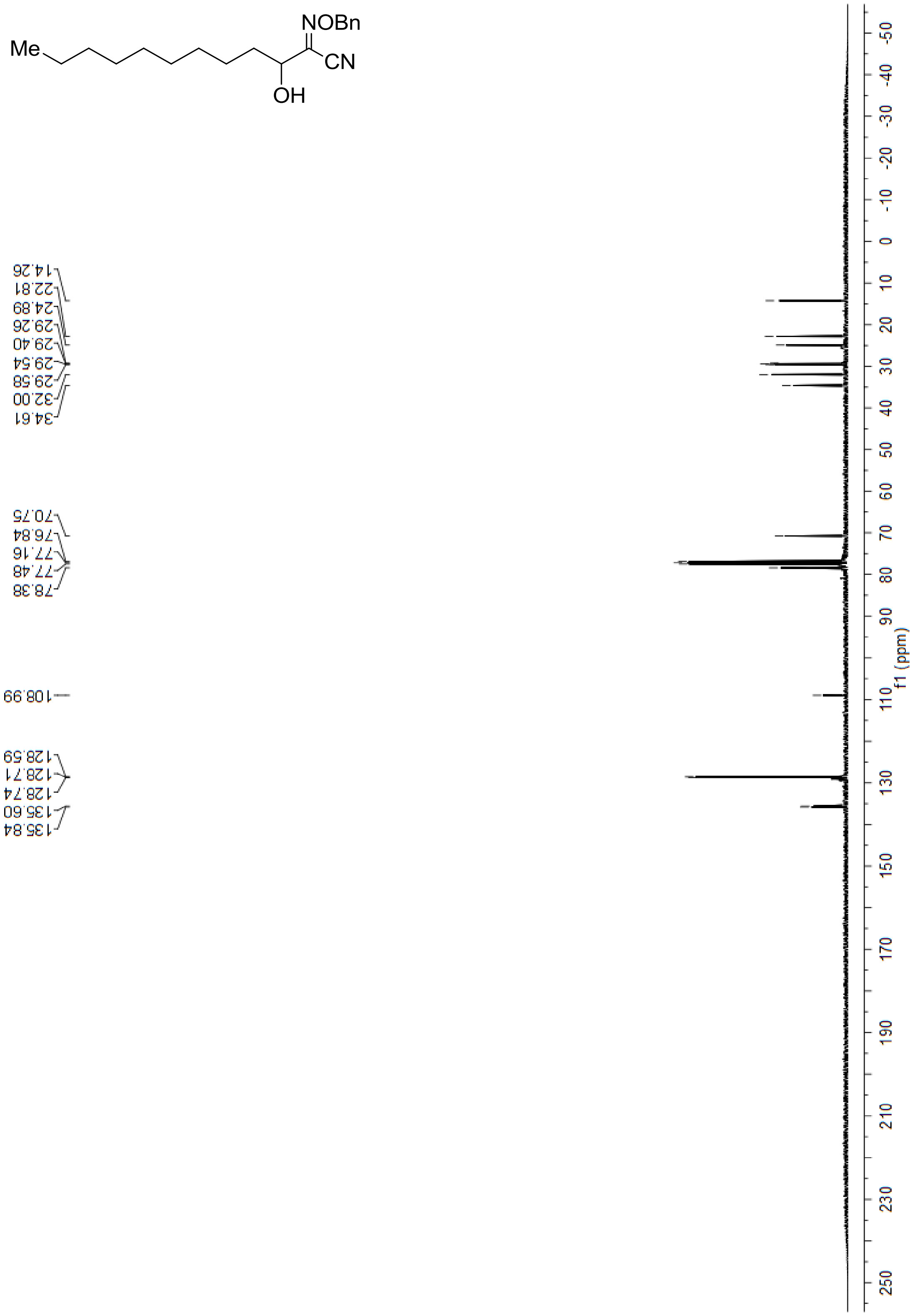

N-(Benzyloxy)-2-hydroxyheptanimidoyl cyanide(3c)

^{1}H NMR of **3c** ($CDCl_3$, 400 MHz, 25 ºC)

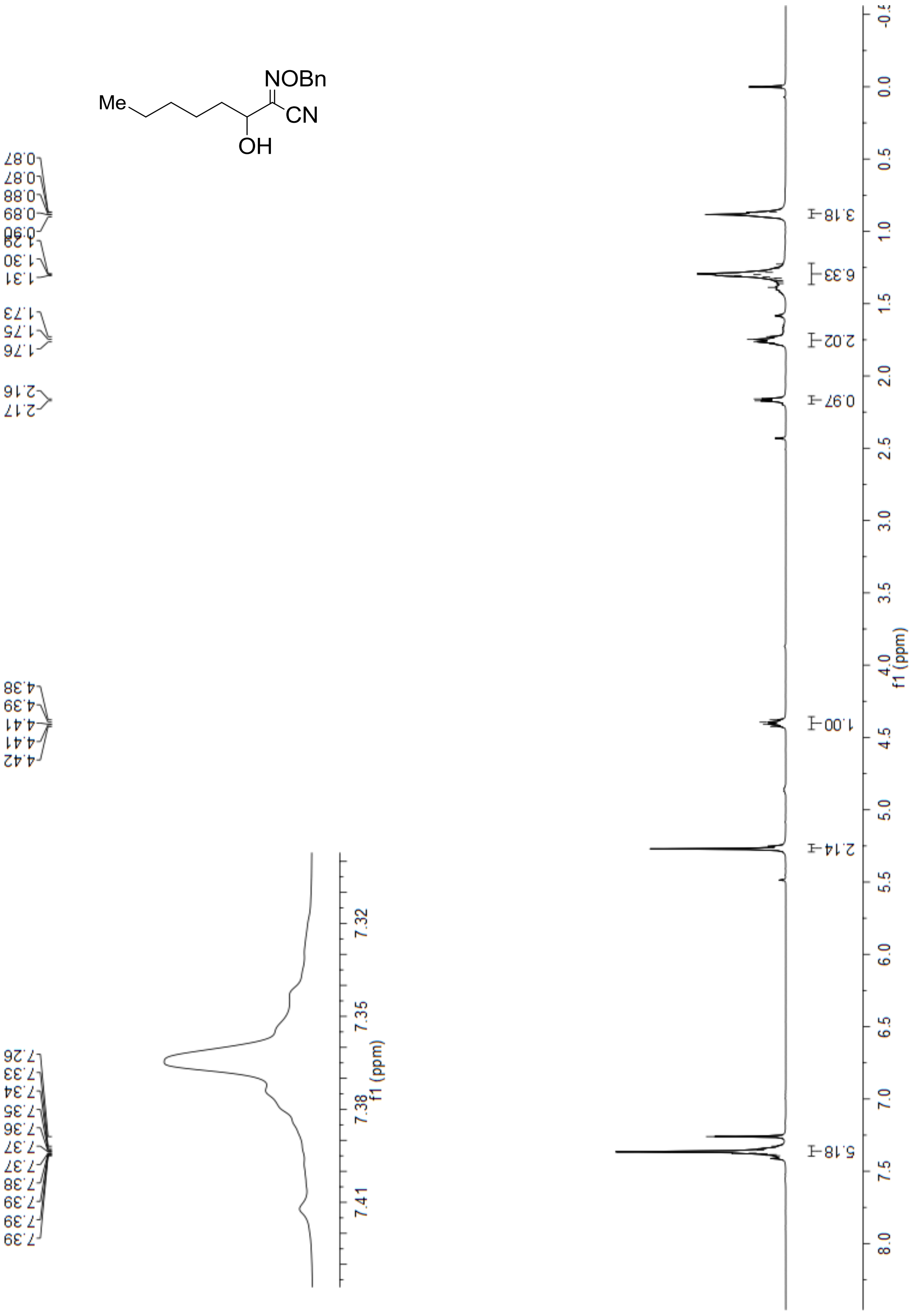

^{13}C NMR of **3c** ($CDCl_3$, 101 MHz, 25 ºC)

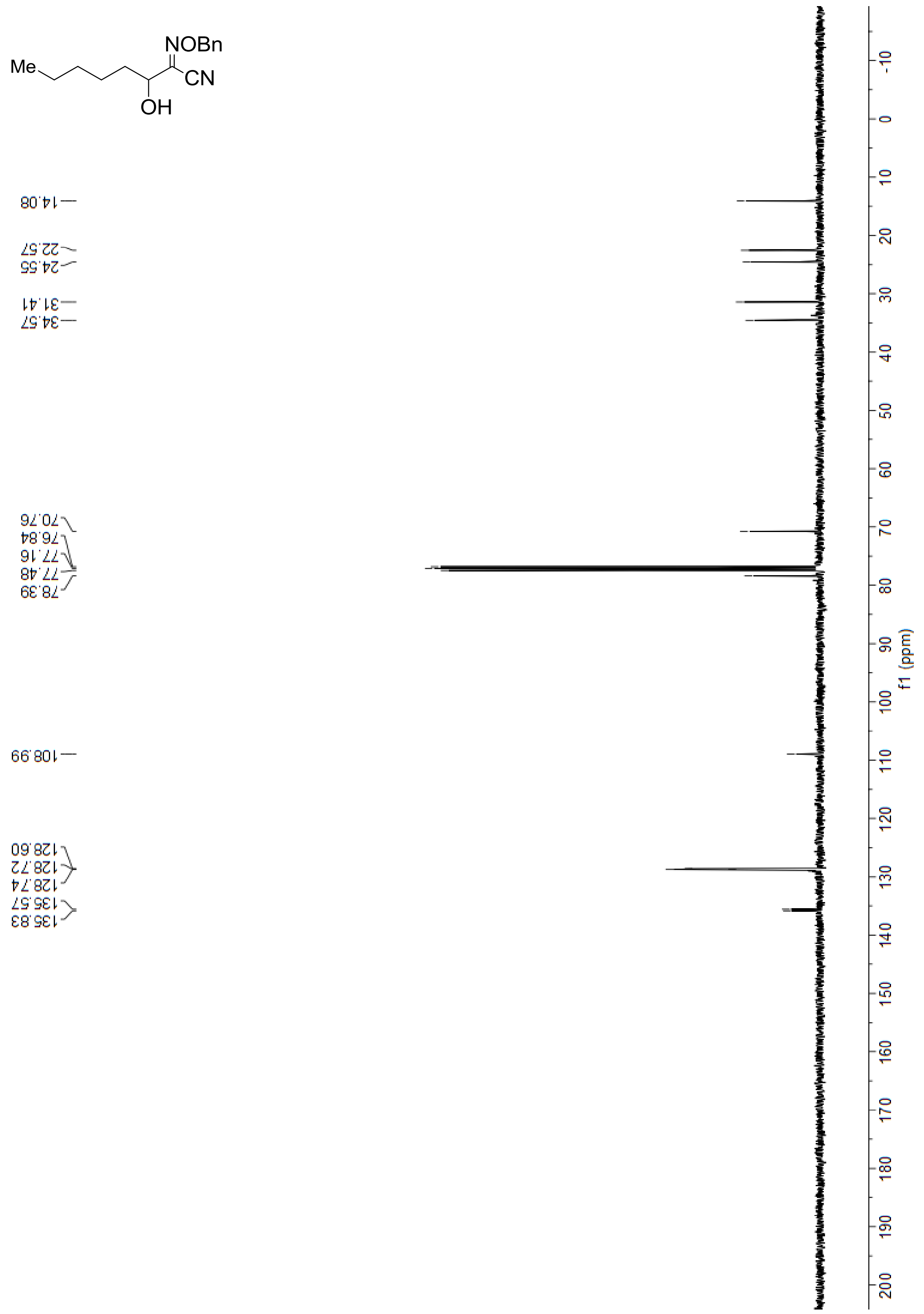

***N*-(Benzyloxy)-2-hydroxytridecanimidoyl cyanide (3d)**

^{1}H NMR of **3d** ($CDCl_3$, 400 MHz, 25 ºC)

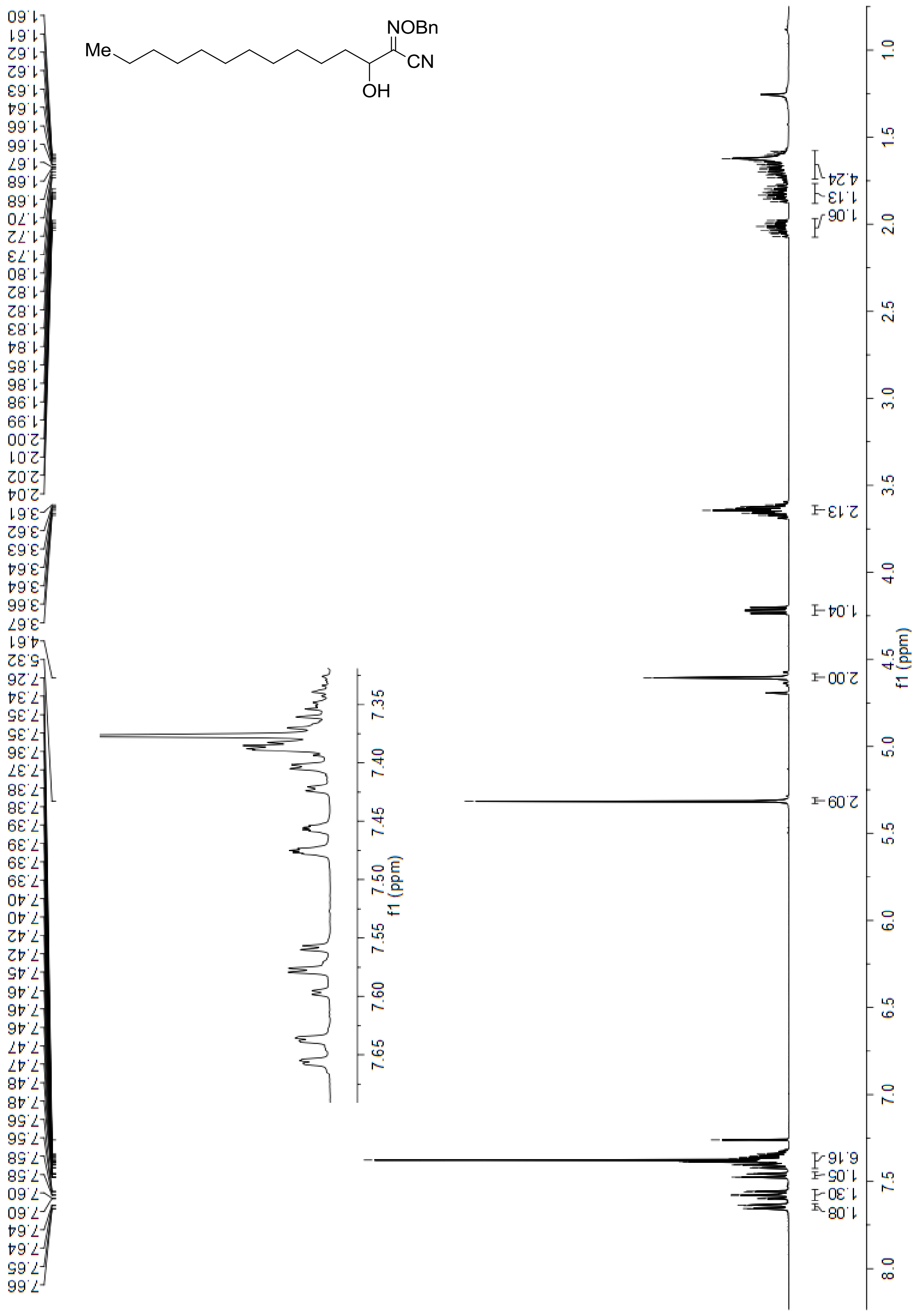

^{13}C NMR of **3d** ($CDCl_3$, 101 MHz, 25 ºC)

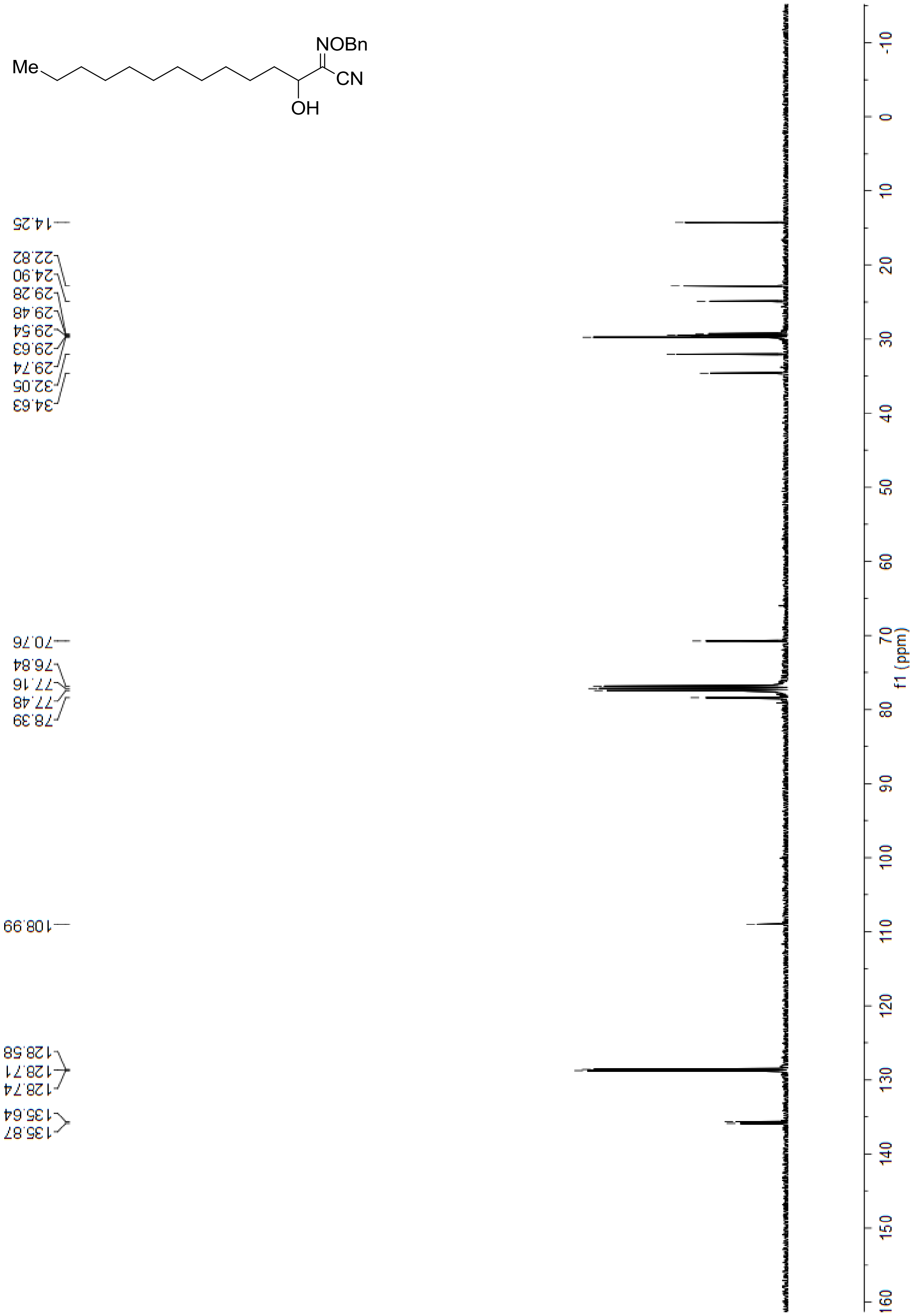

6-Azido-N-(benzyloxy)-2-hydroxyhexanimidoyl cyanide (3e)

^{1}H NMR of **3e** ($CDCl_3$, 400 MHz, 25 ºC)

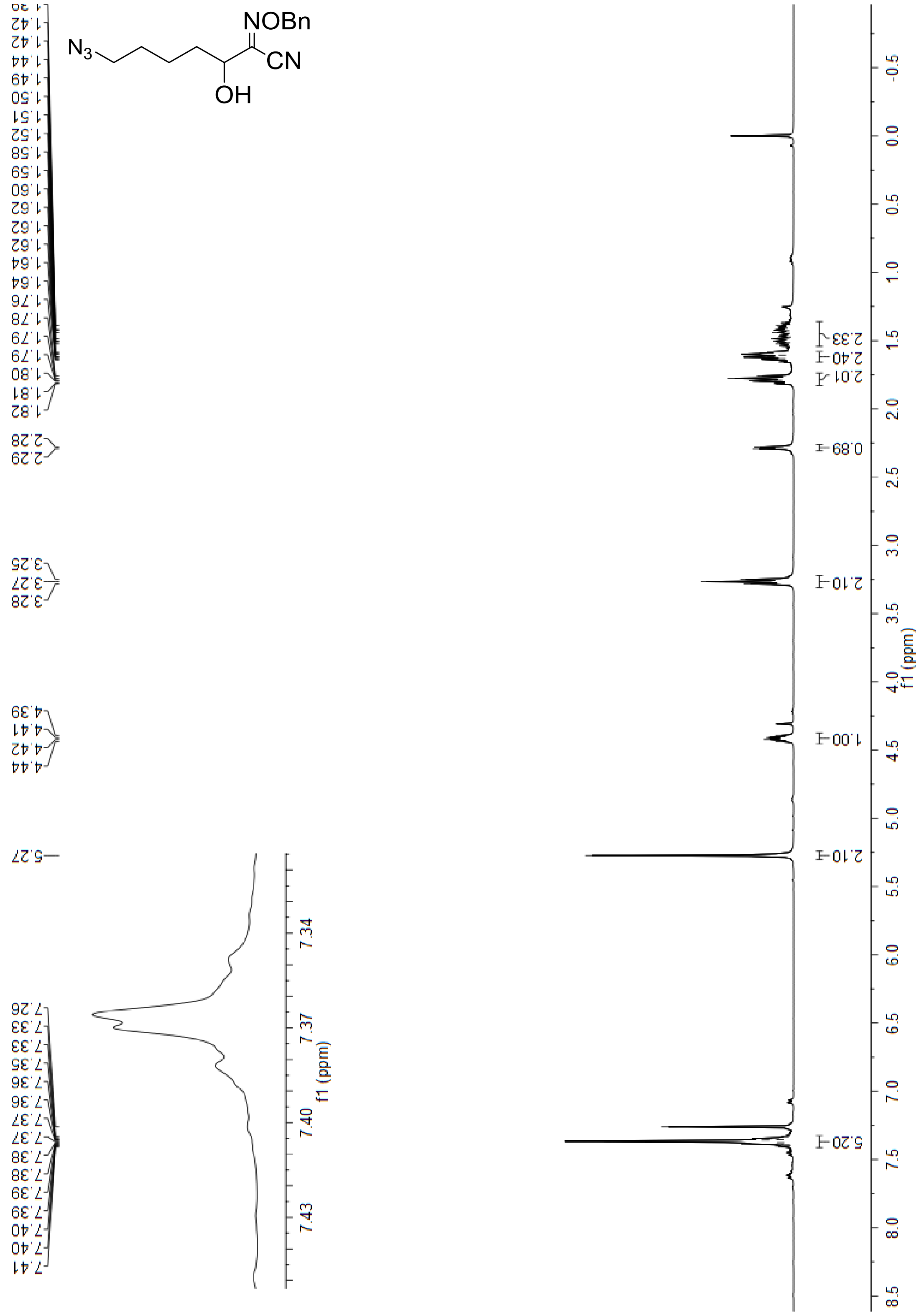

^{13}C NMR of **3e** ($CDCl_3$, 101 MHz, 25 ºC)

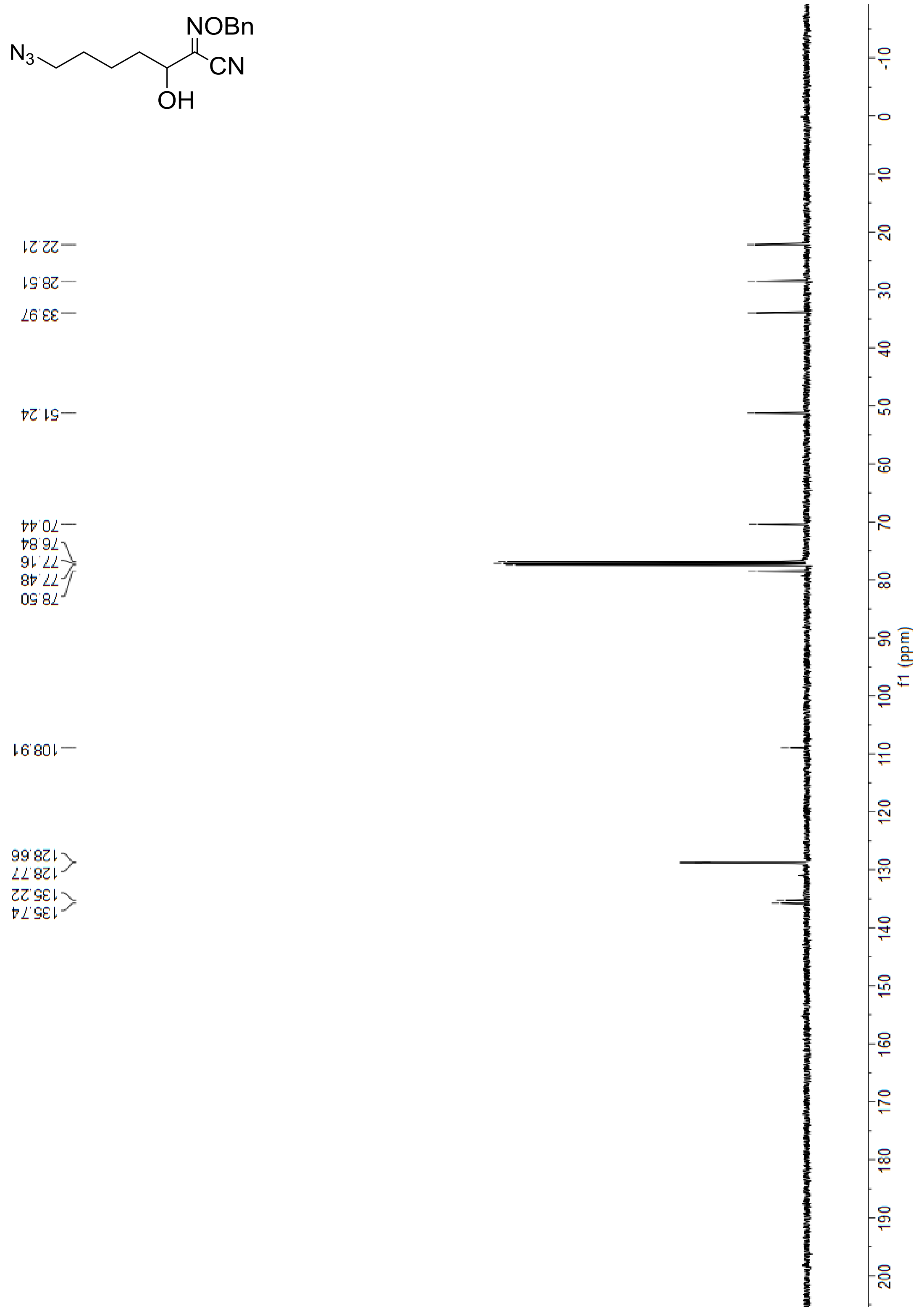

N-(Benzyloxy)-7-bromo-2-hydroxyheptanimidoyl cyanide(3f)

^{1}H NMR of **3f** ($CDCl_3$, 400 MHz, 25 ºC)

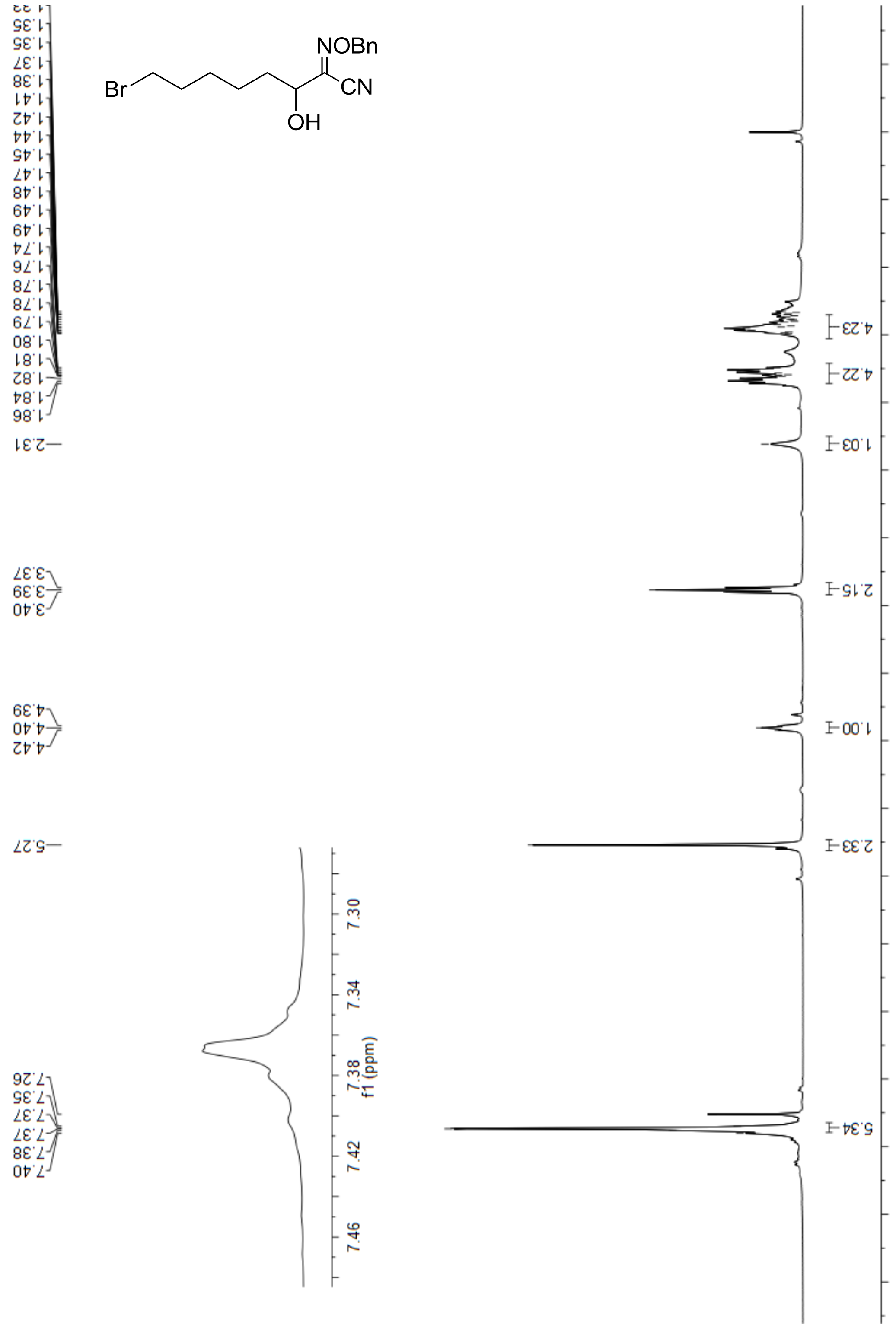

^{13}C NMR of **3f** ($CDCl_3$, 101 MHz, 25 ºC)

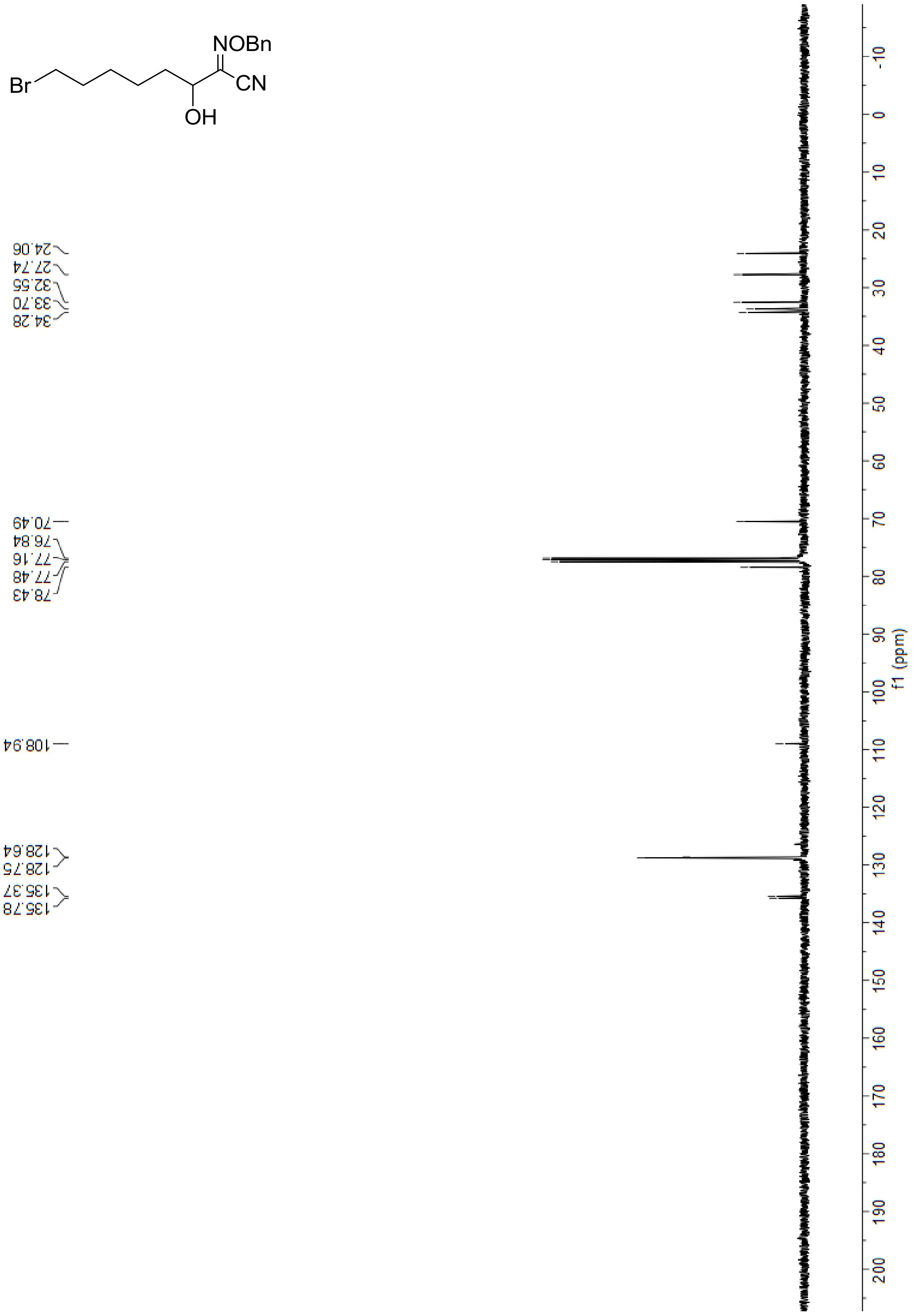

N,5-Bis(benzyloxy)-2-hydroxypentanimidoyl cyanide (3g)

^{1}H NMR of **3g** ($CDCl_3$, 400 MHz, 25 ºC)

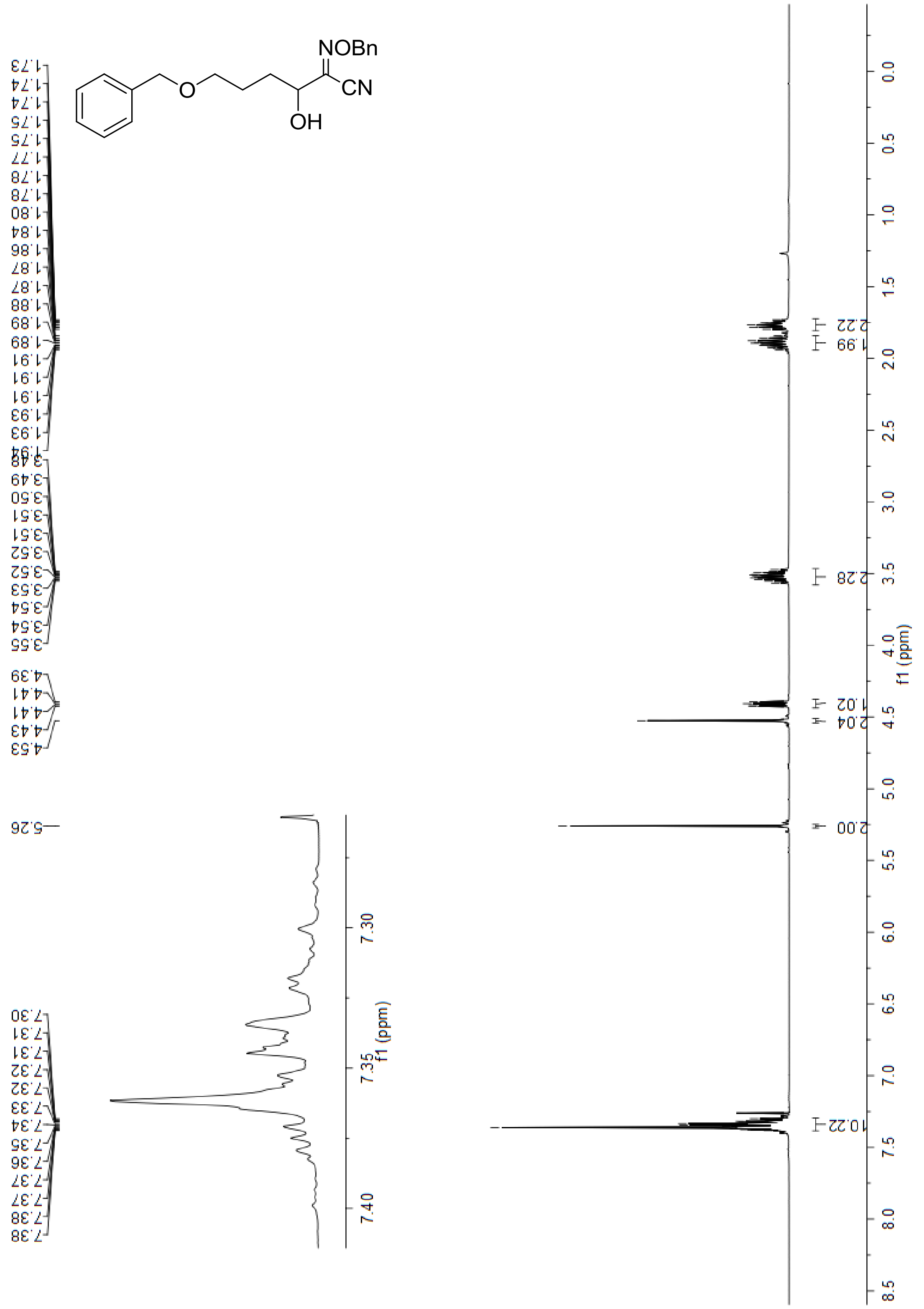

^{13}C NMR of **3g** ($CDCl_3$, 101 MHz, 25 ºC)

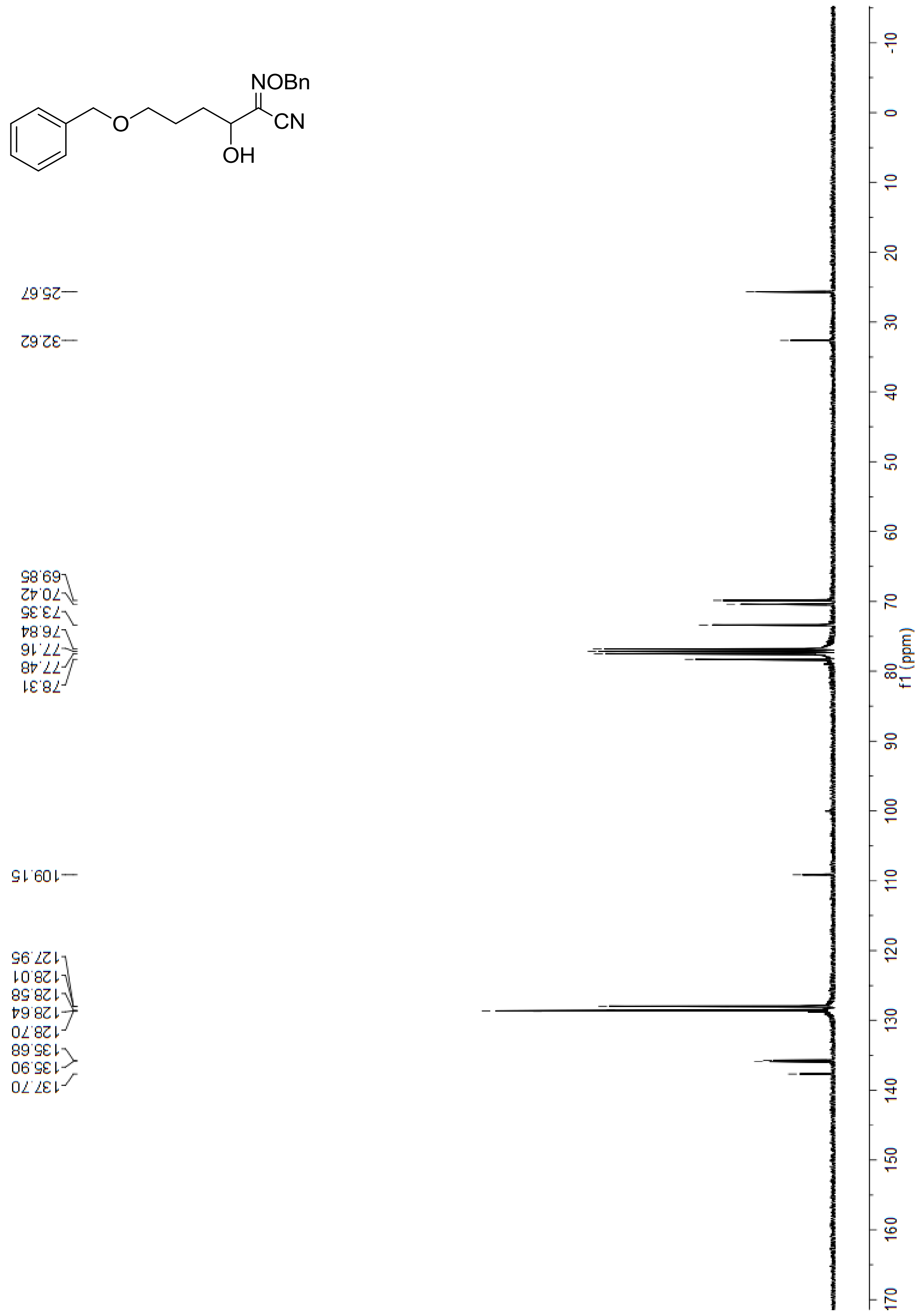

***N*-(Benzyloxy)-2-hydroxy-6-phenoxyhexanimidoyl cyanide(3h)**

^{1}H NMR of **3h** ($CDCl_3$, 400 MHz, 25 ºC)

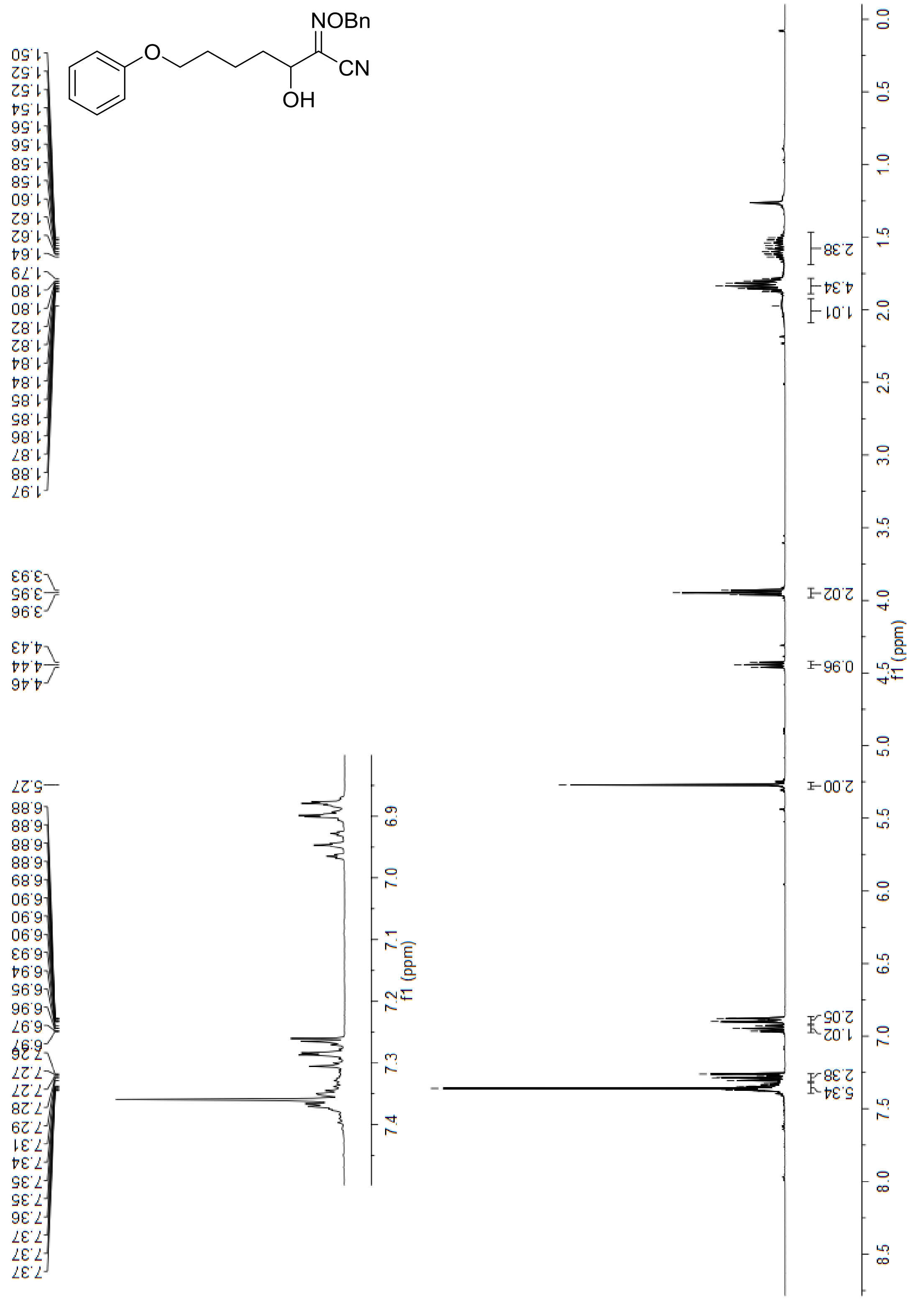

^{13}C NMR of **3h** ($CDCl_3$, 101 MHz, 25 ºC)

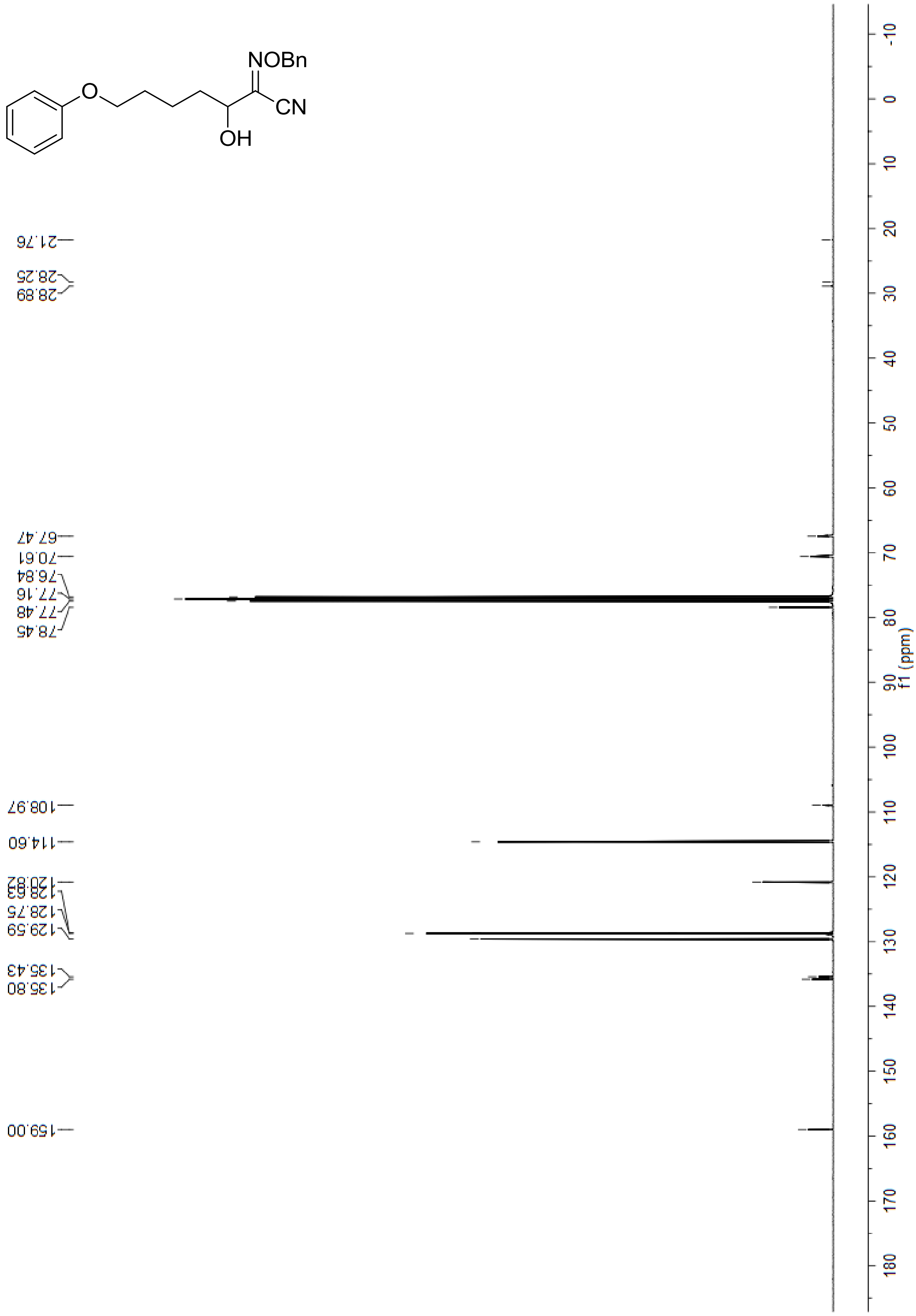

N,5-Bis(benzyloxy)-2-hydroxypentanimidoyl cyanide (3i)

^{1}H NMR of **3i** ($CDCl_3$, 400 MHz, 25 ºC)

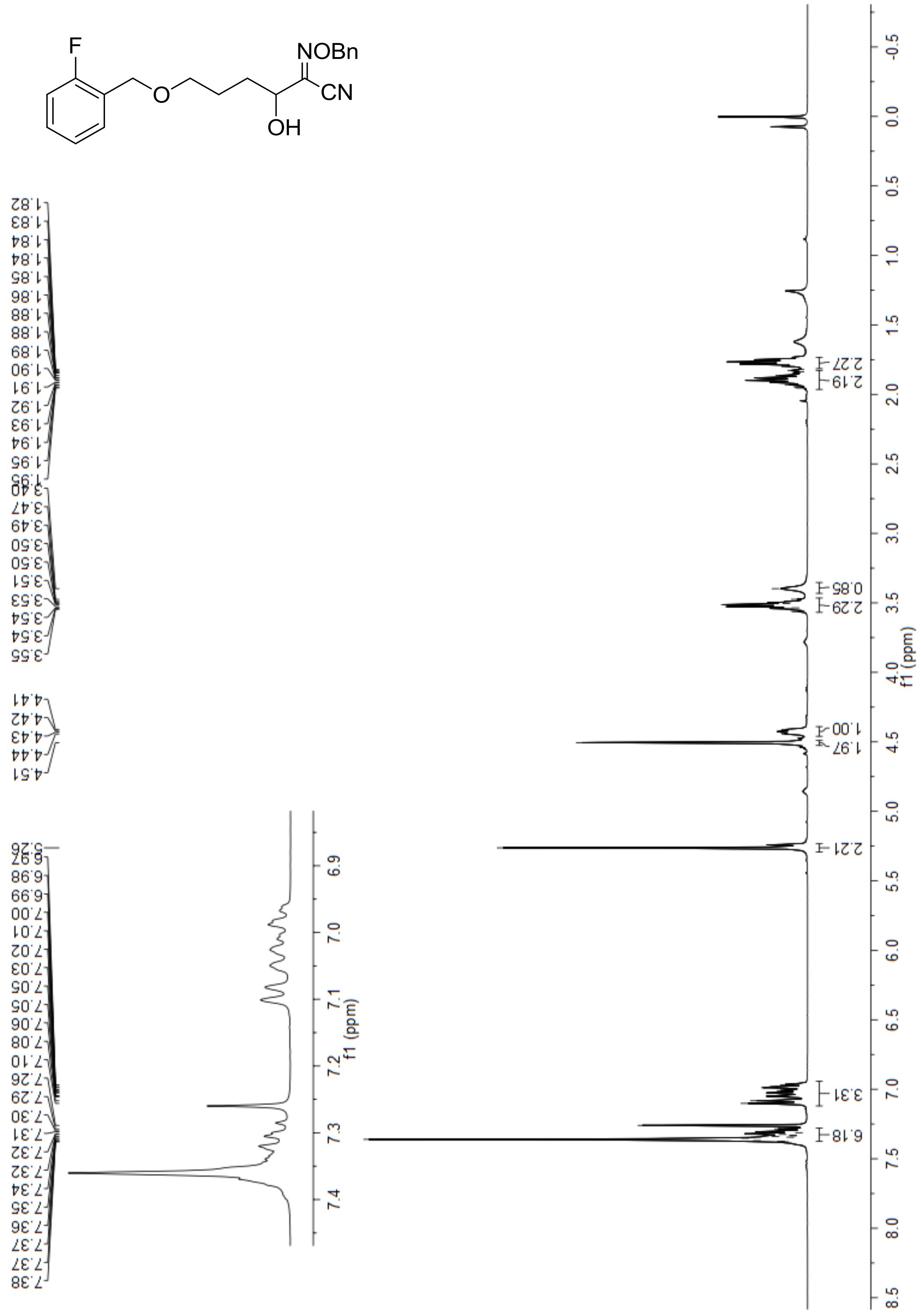

^{13}C NMR of **3i** ($CDCl_3$, 101 MHz, 25 ºC)

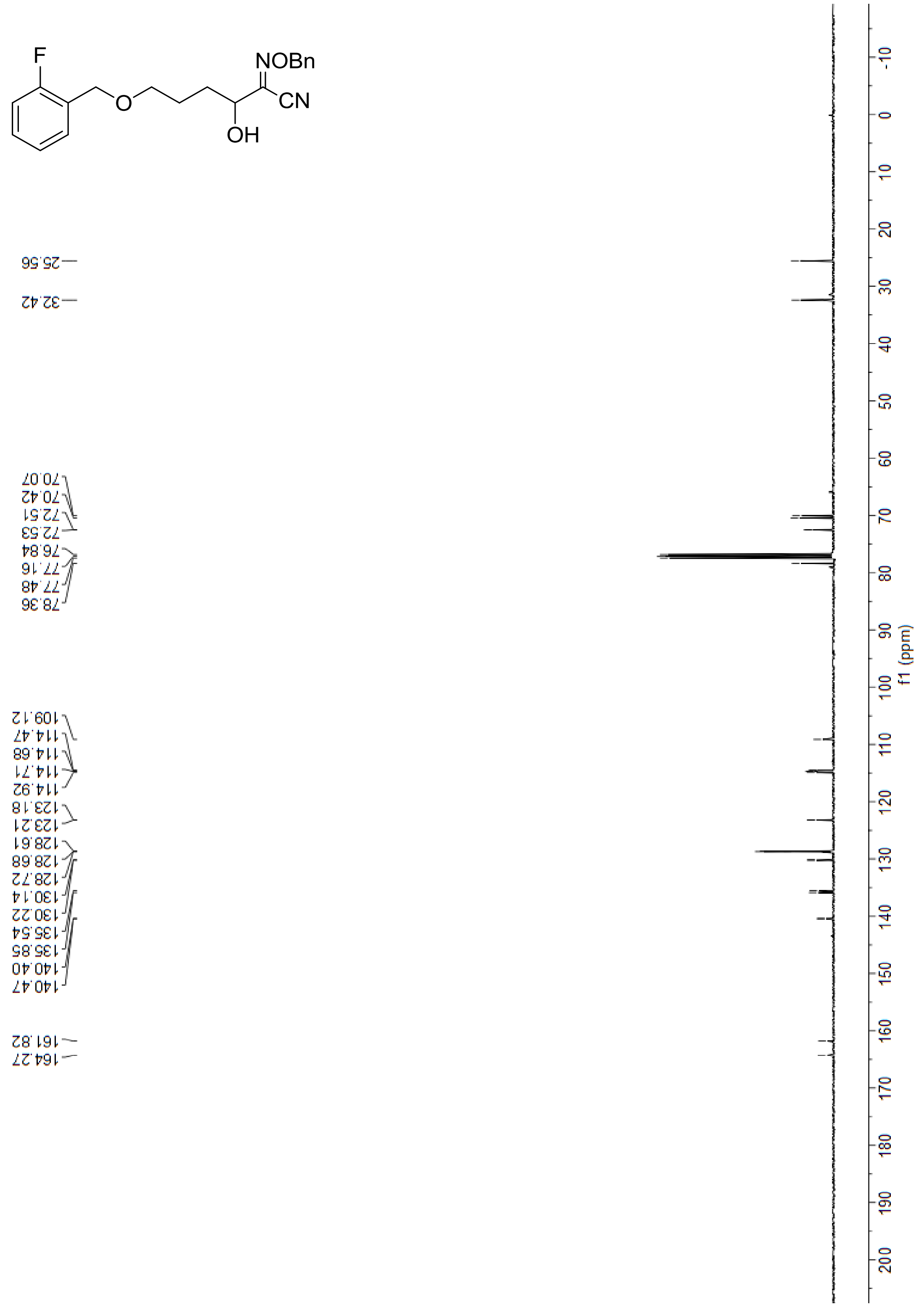

^{19}F NMR of **3i** ($CDCl_3$, 375 MHz, 25 ºC)

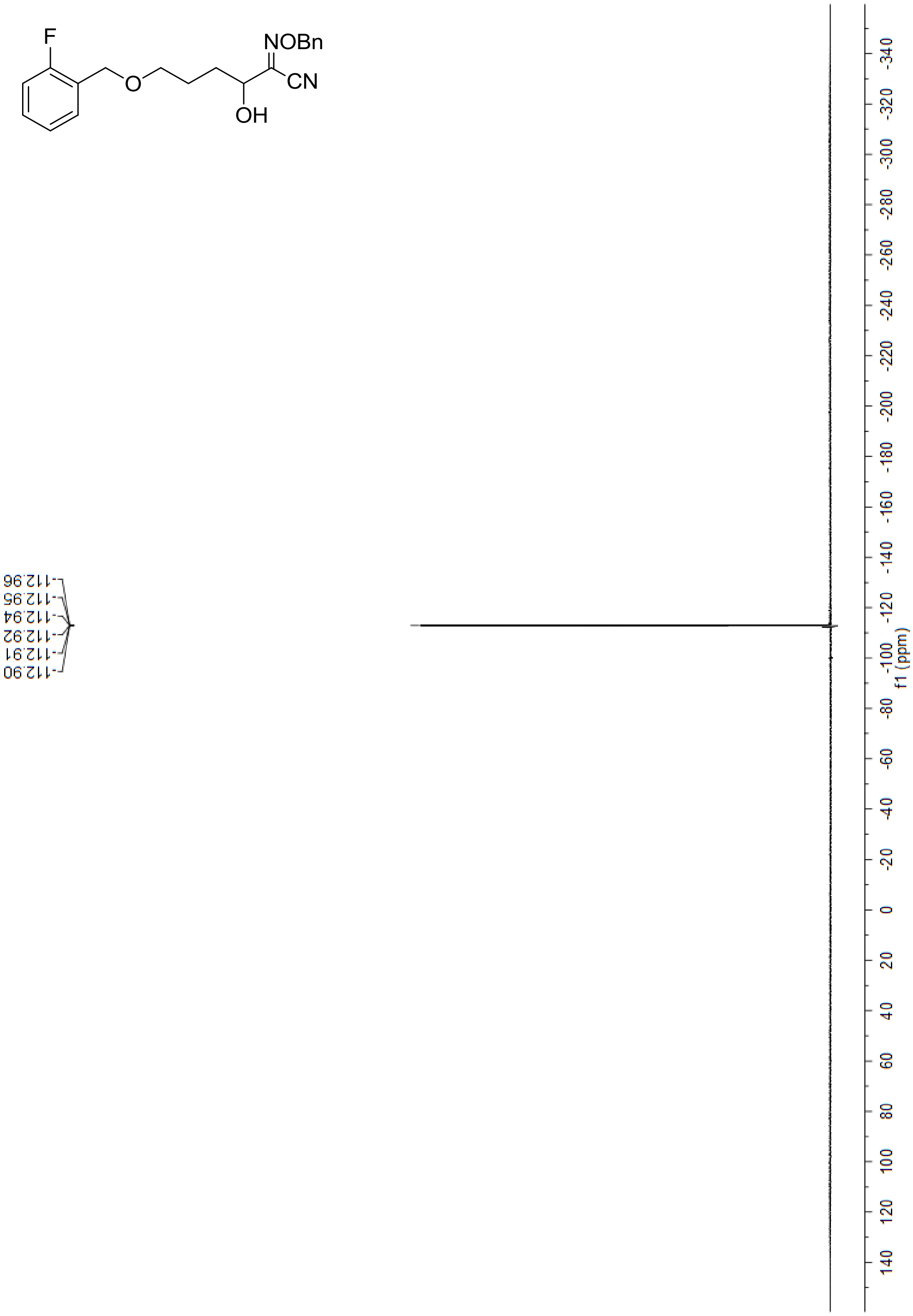

***N*-(Benzyloxy)-5-((3-fluorobenzyl)oxy)-2-hydroxypentanimidoyl cyanide (3j)**

^{1}H NMR of **3j** ($CDCl_3$, 400 MHz, 25 ºC)

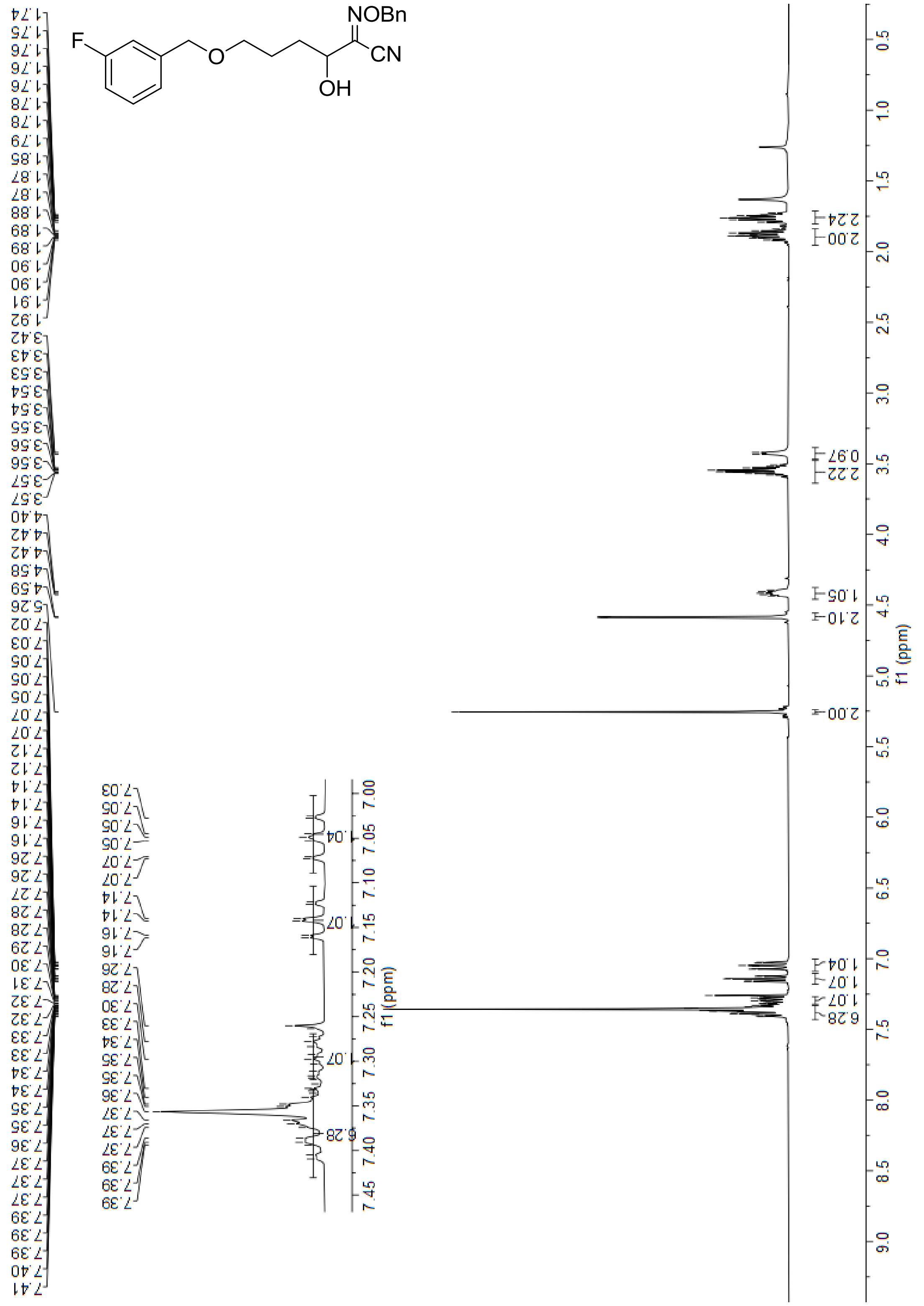

^{13}C NMR of **3j** ($CDCl_3$, 101 MHz, 25 ºC)

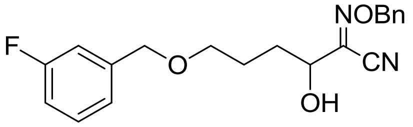

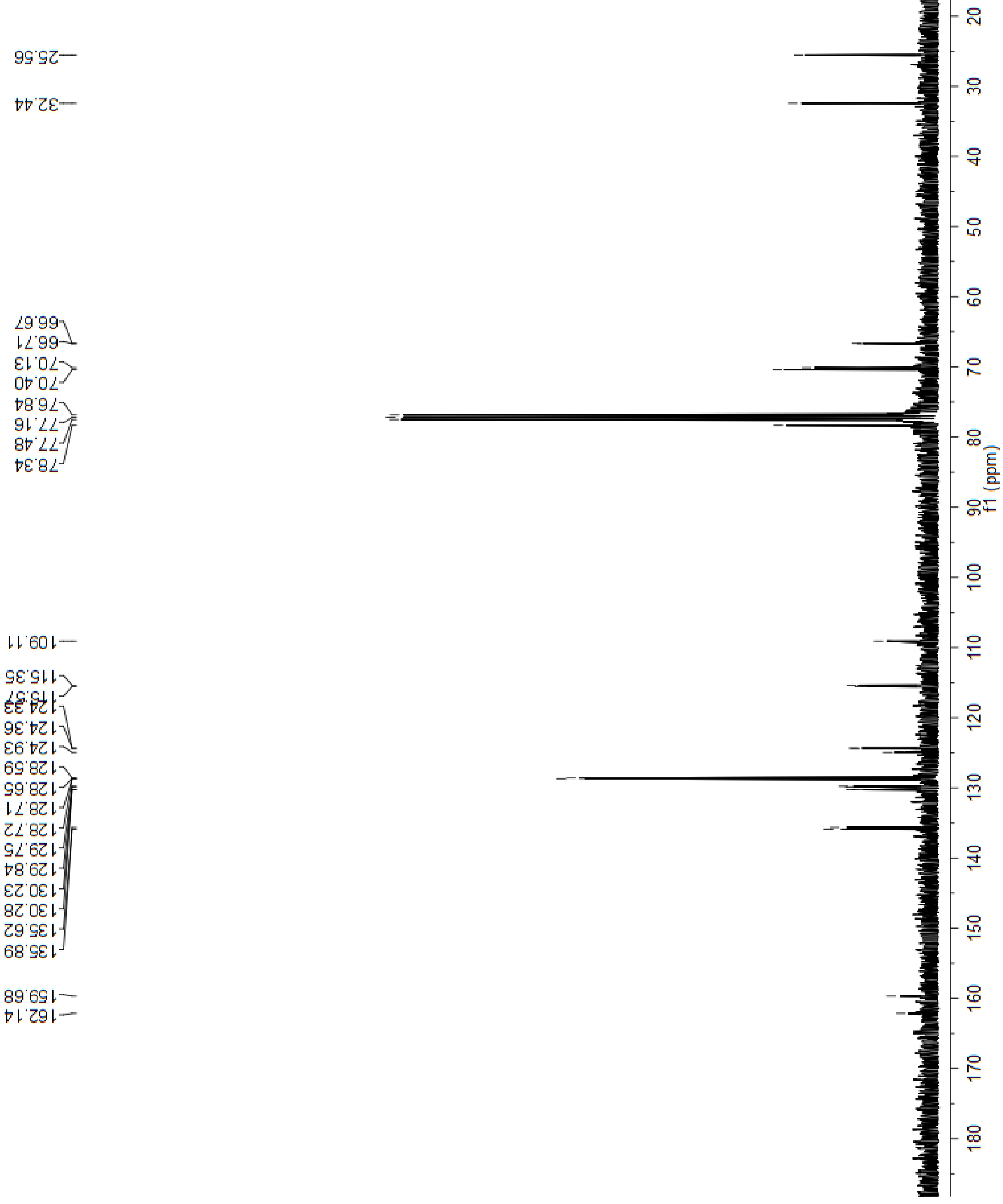

^{19}F NMR of **3j** ($CDCl_3$, 375 MHz, 25 ºC)

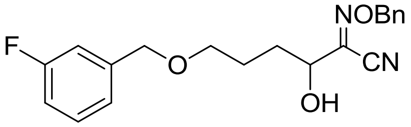

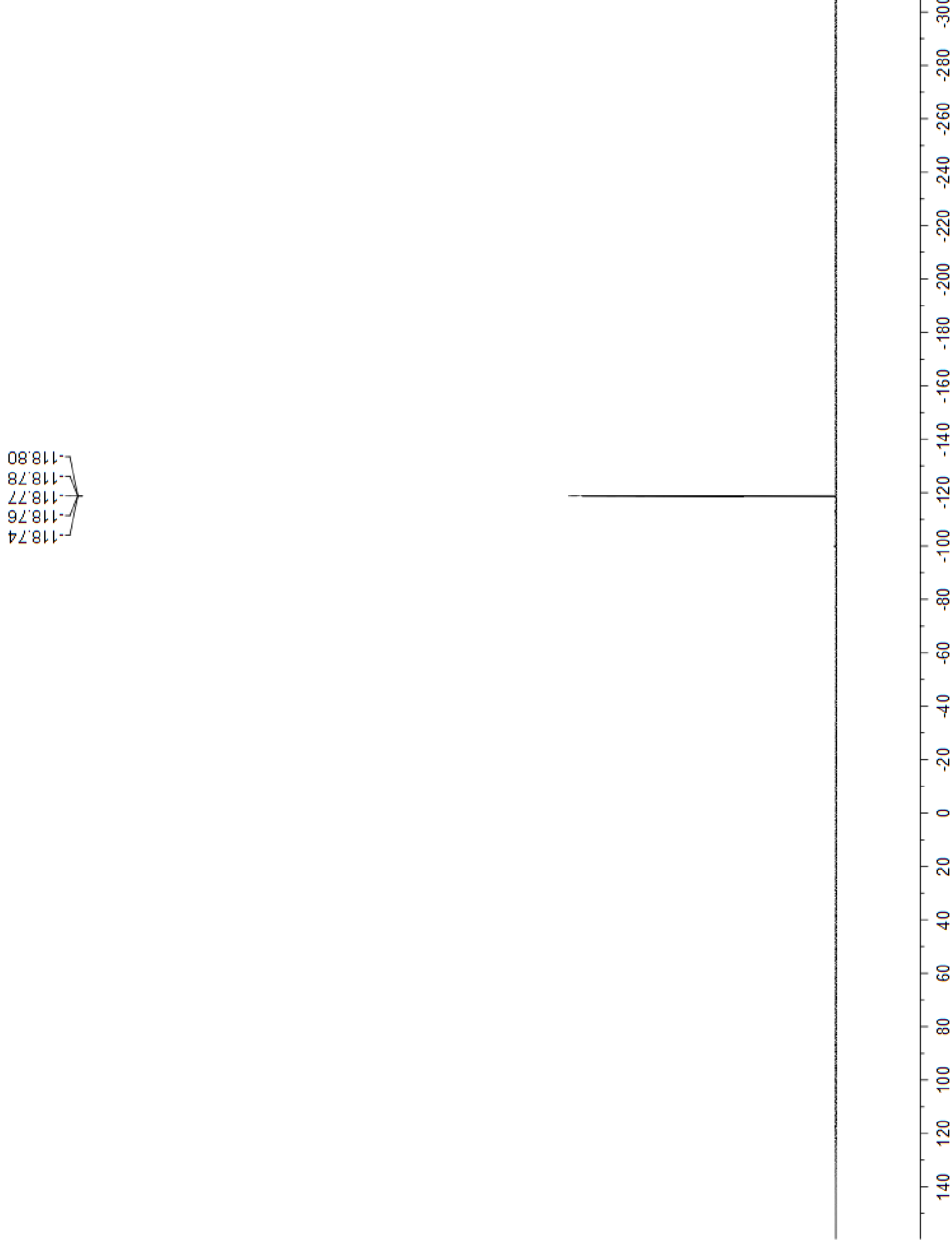

***N*-(Benzyloxy)-5-((4-fluorobenzyl)oxy)-2-hydroxypentanimidoyl cyanide (3k)**

^{1}H NMR of **3k** ($CDCl_3$, 400 MHz, 25 ºC)

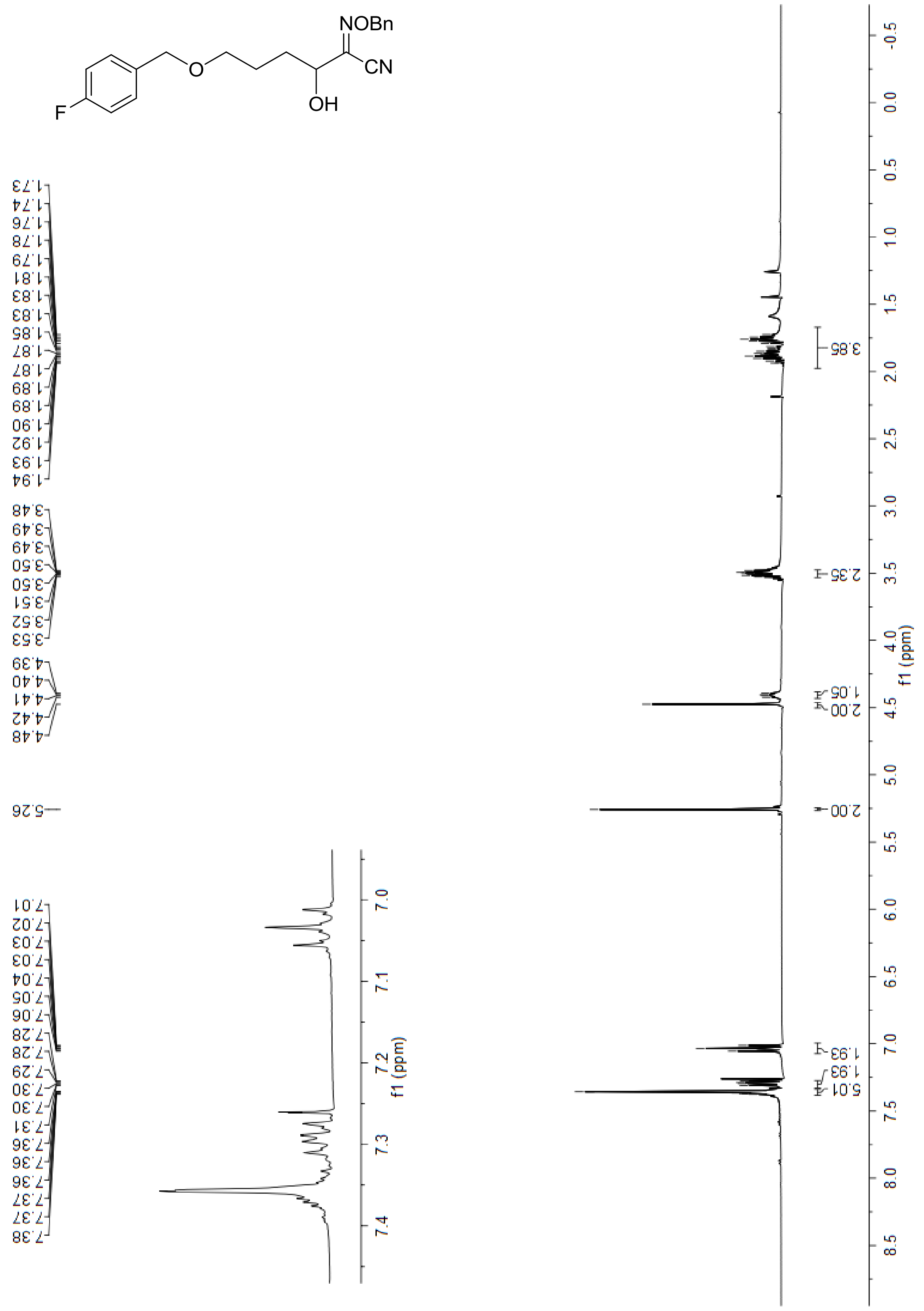

^{13}C NMR of **3k** ($CDCl_3$, 101 MHz, 25 ºC)

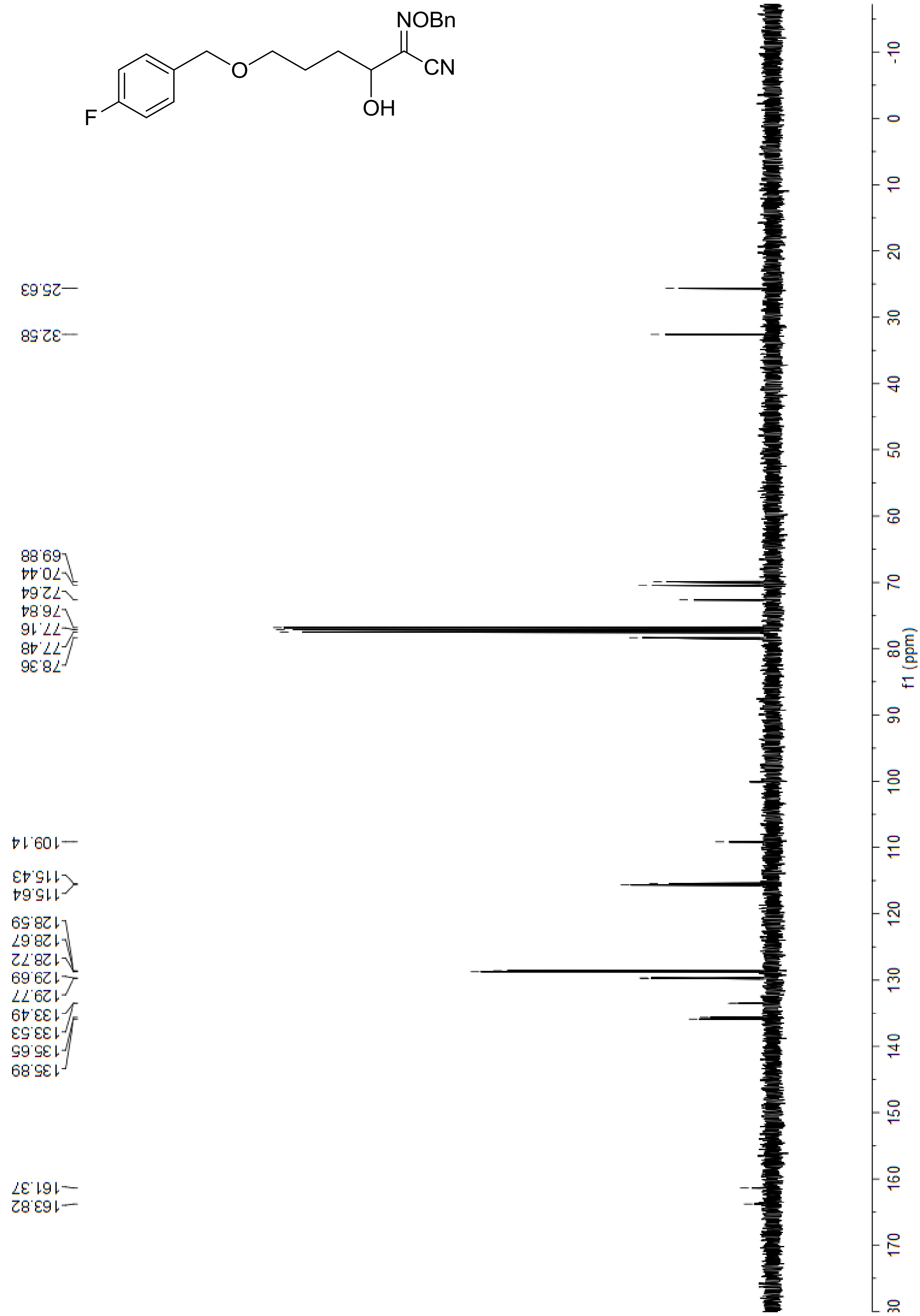

^{19}F NMR of **3k** ($CDCl_3$, 375 MHz, 25 ºC)

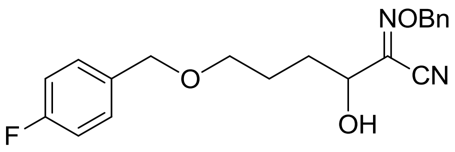

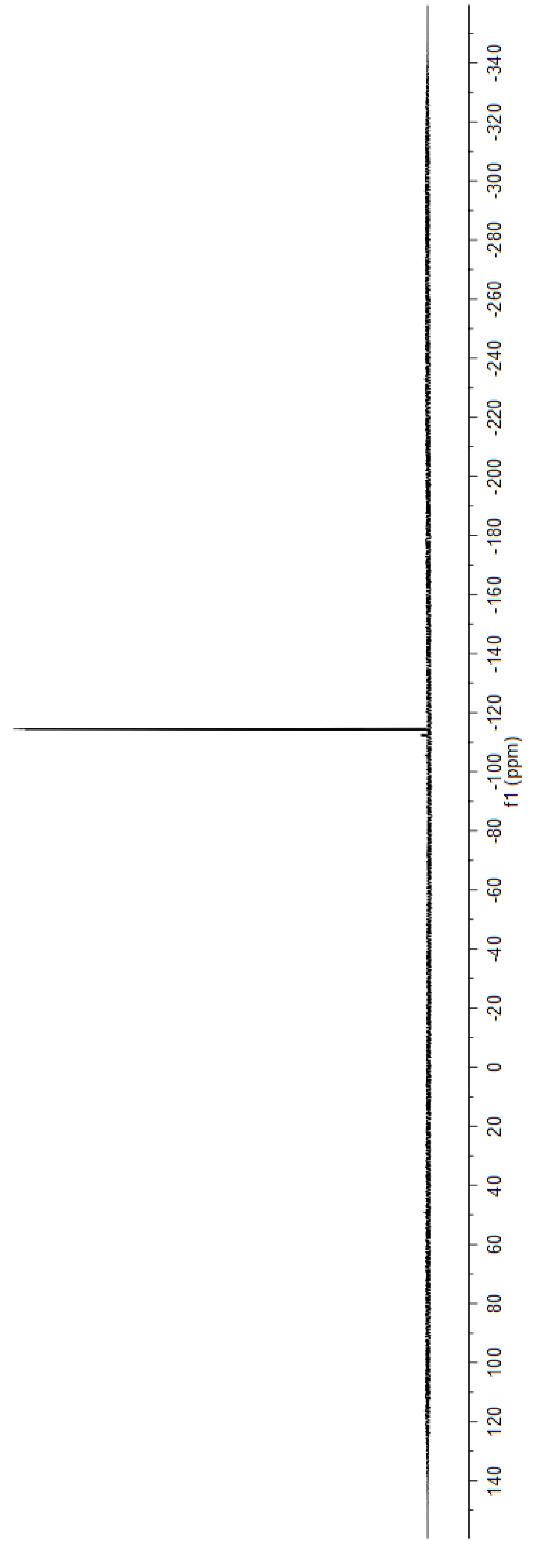

***N*-(Benzyloxy)-5-((3-chlorobenzyl)oxy)-2-hydroxypentanimidoyl cyanide (3l)**

^{1}H NMR of **3l** ($CDCl_3$, 400 MHz, 25 ºC)

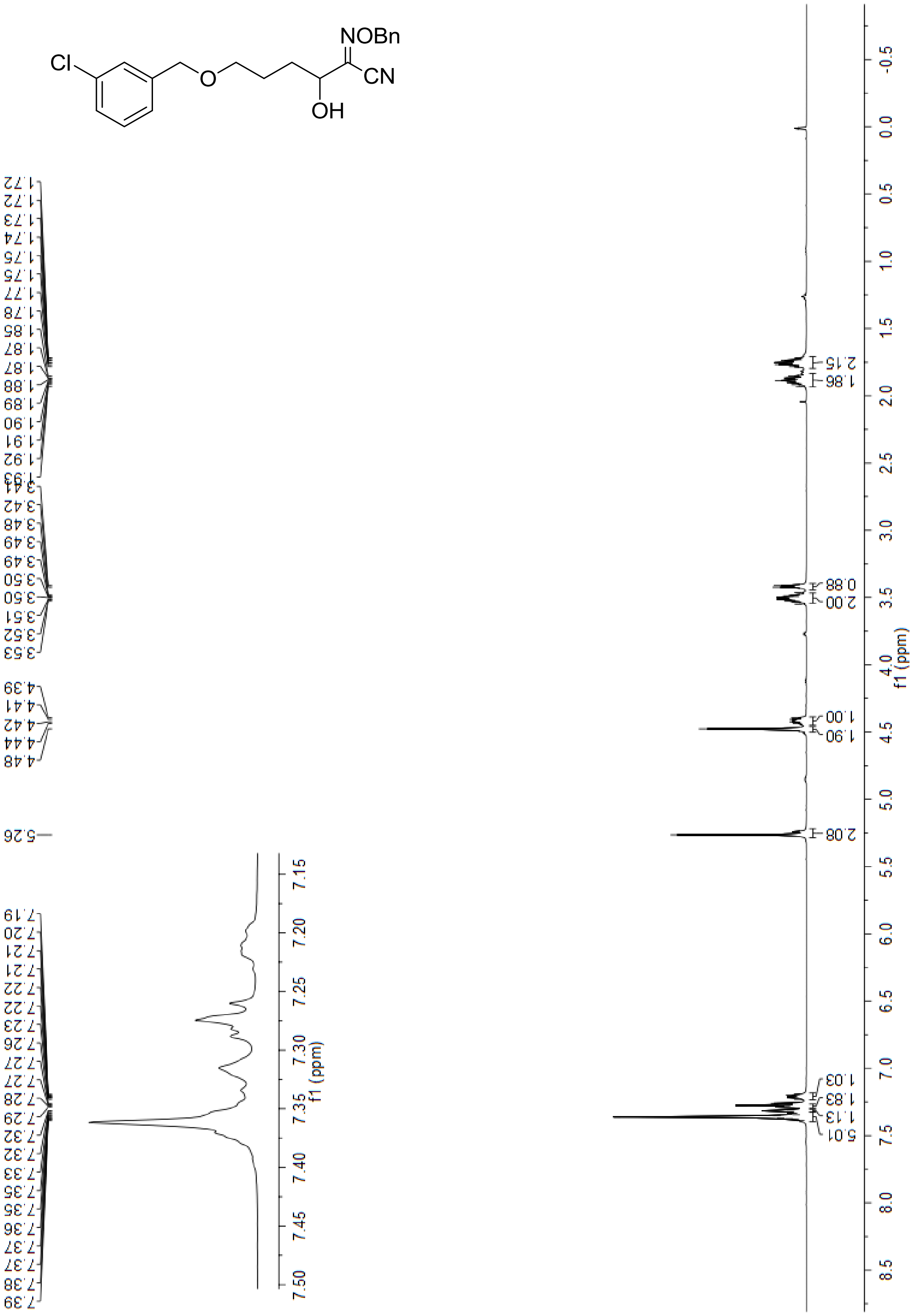

^{13}C NMR of **3l** ($CDCl_3$, 101 MHz, 25 ºC)

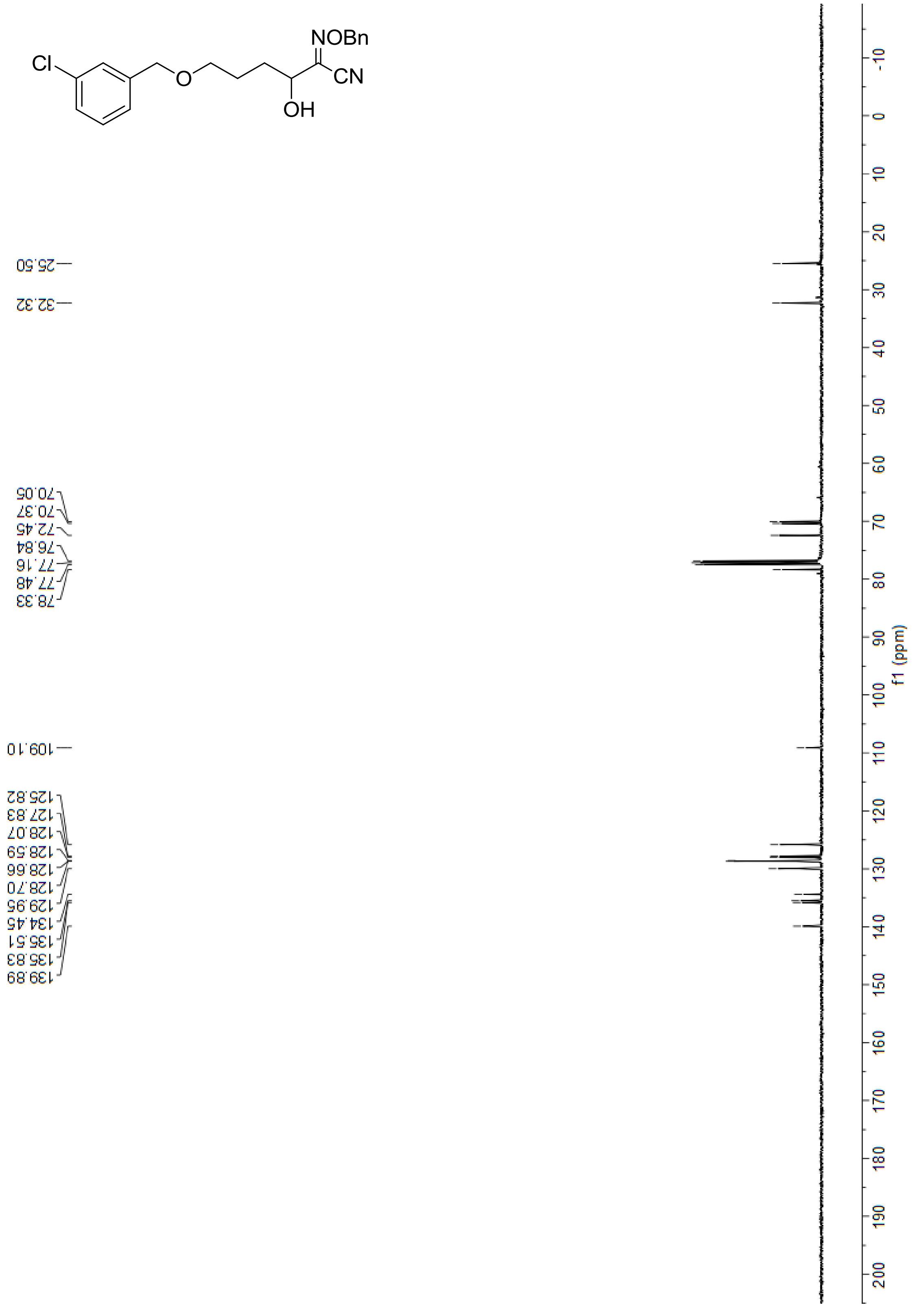

N*-(Benzyloxy)-5-((3-bromobenzyl)oxy)-2-hydroxypentanimidoyl cyanide (3m)

^{1}H NMR of **3m** ($CDCl_3$, 400 MHz, 25 ºC)

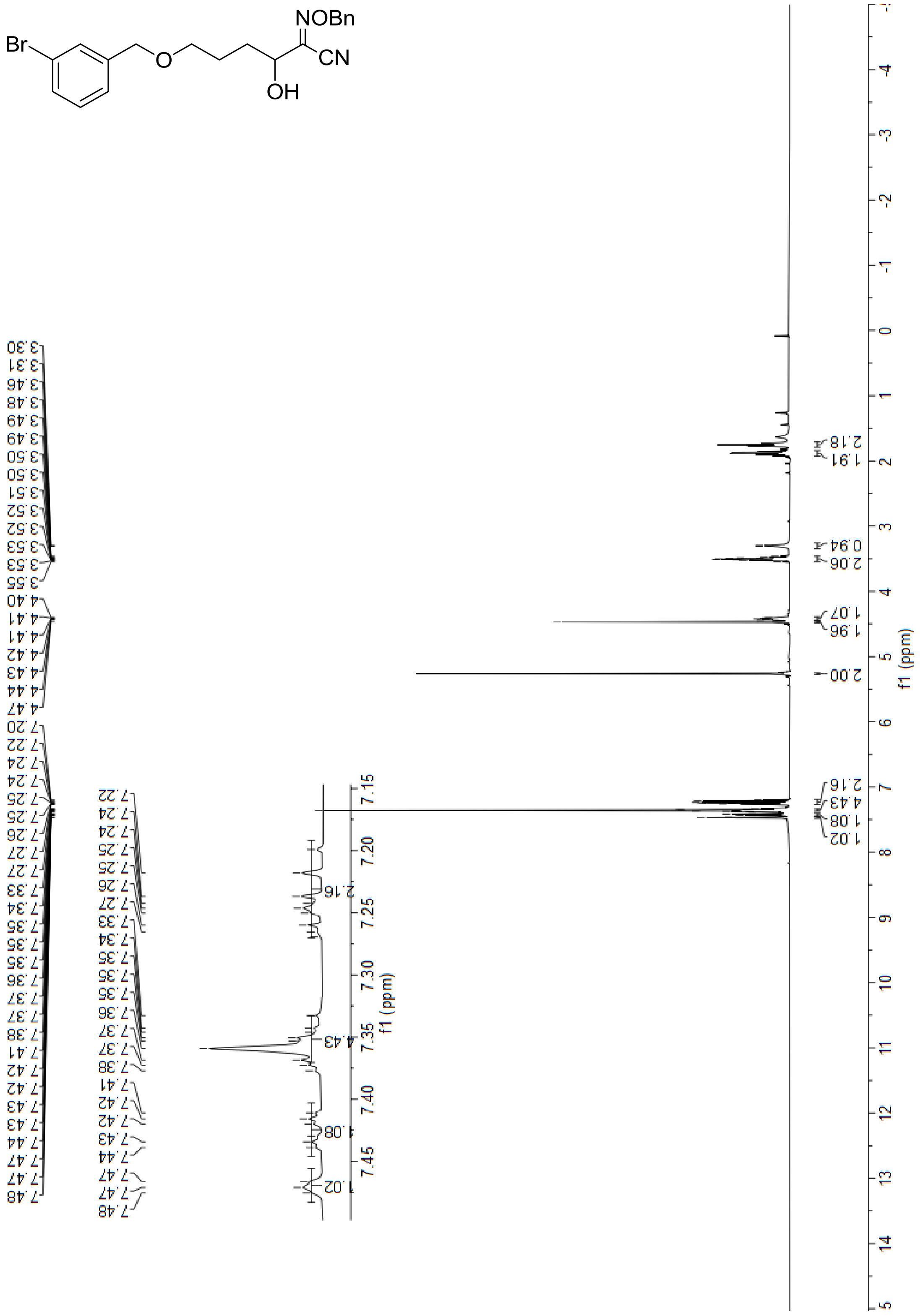

^{13}C NMR of **3m** ($CDCl_3$, 101 MHz, 25 ºC)

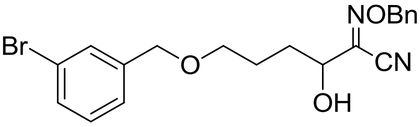

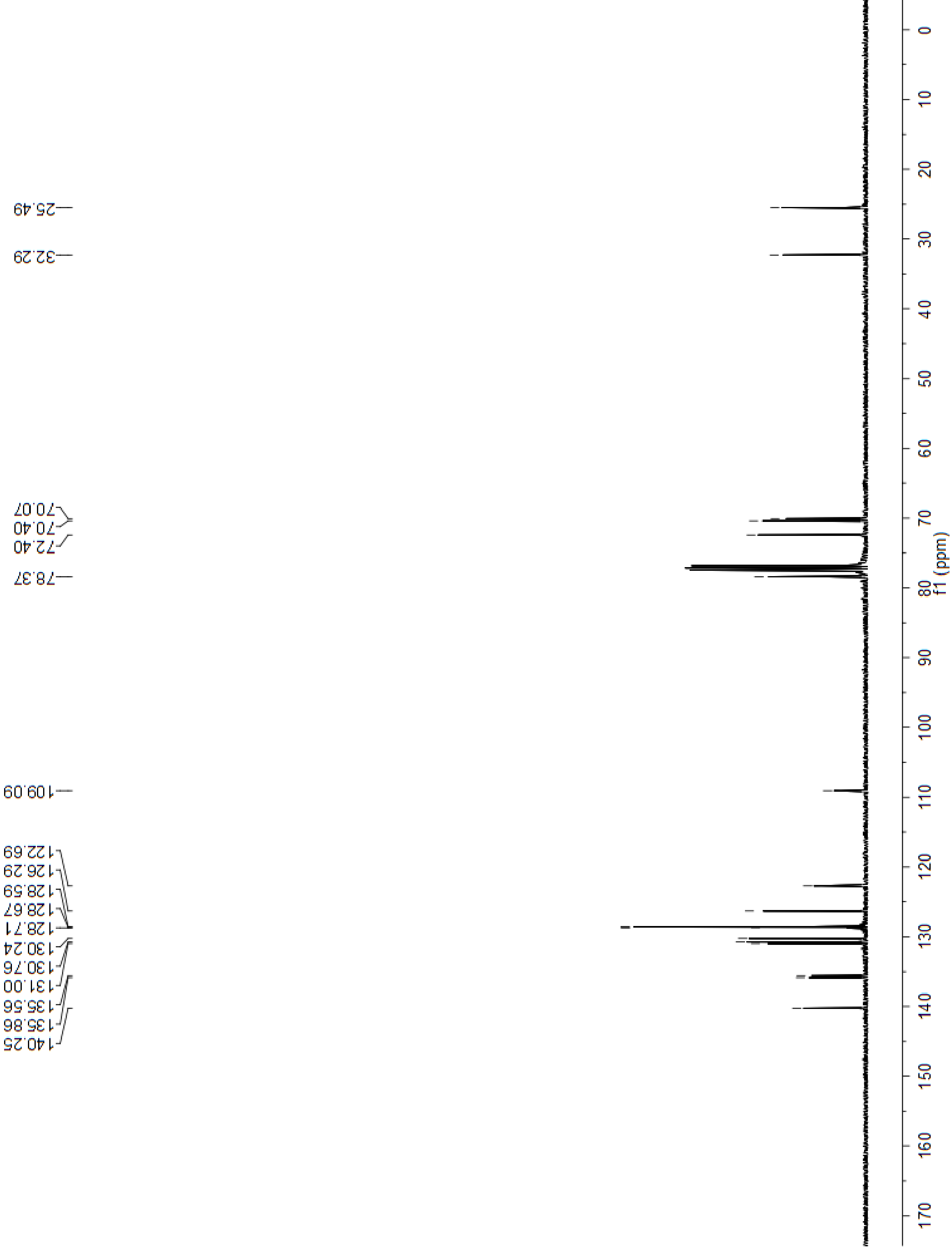

***N*-(Benzyloxy)-5-((2-bromobenzyl)oxy)-2-hydroxypentanimidoyl cyanide (3n)**

^{1}H NMR of **3n** ($CDCl_3$, 400 MHz, 25 ºC)

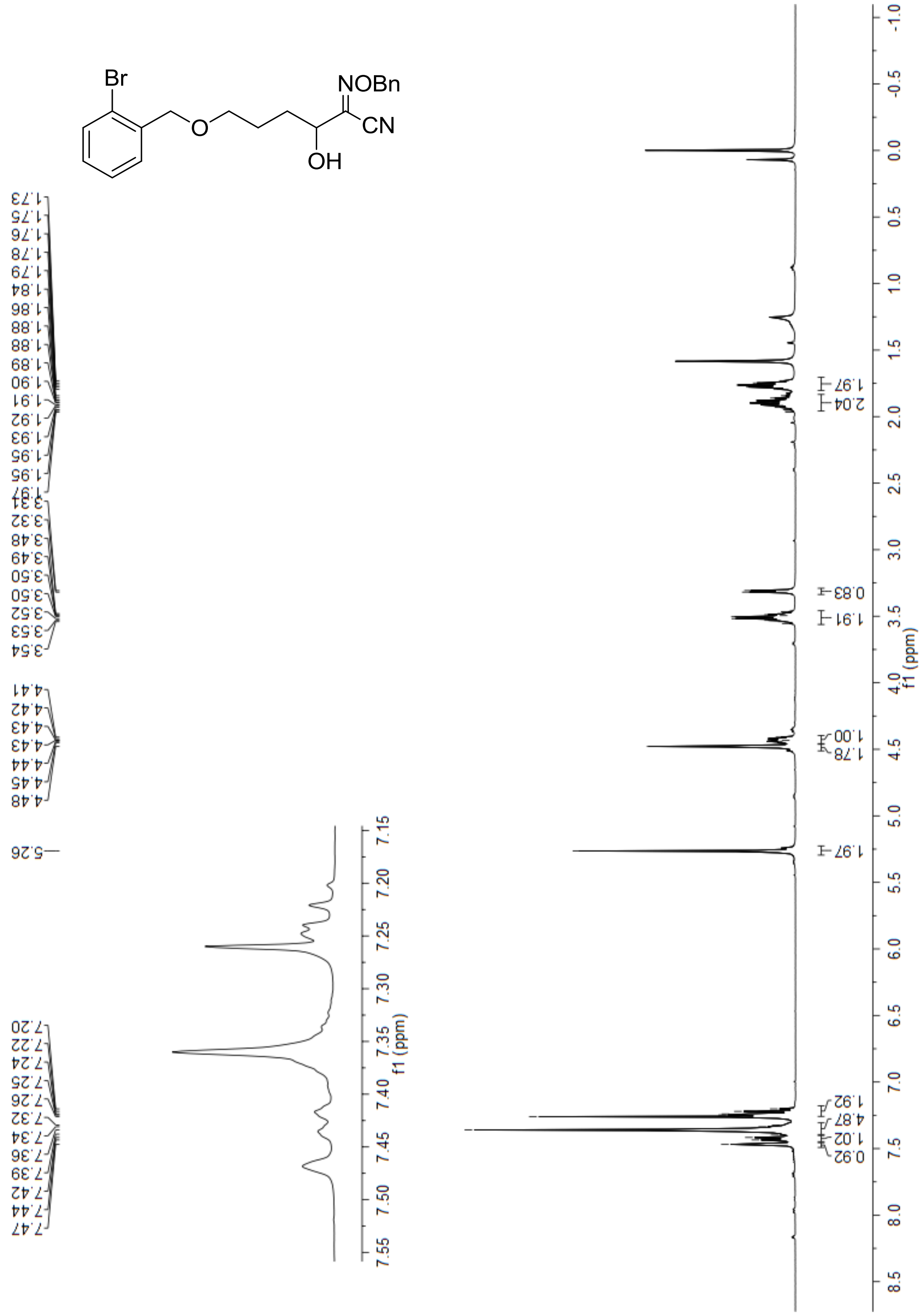

^{13}C NMR of **3n** ($CDCl_3$, 101 MHz, 25 ºC)

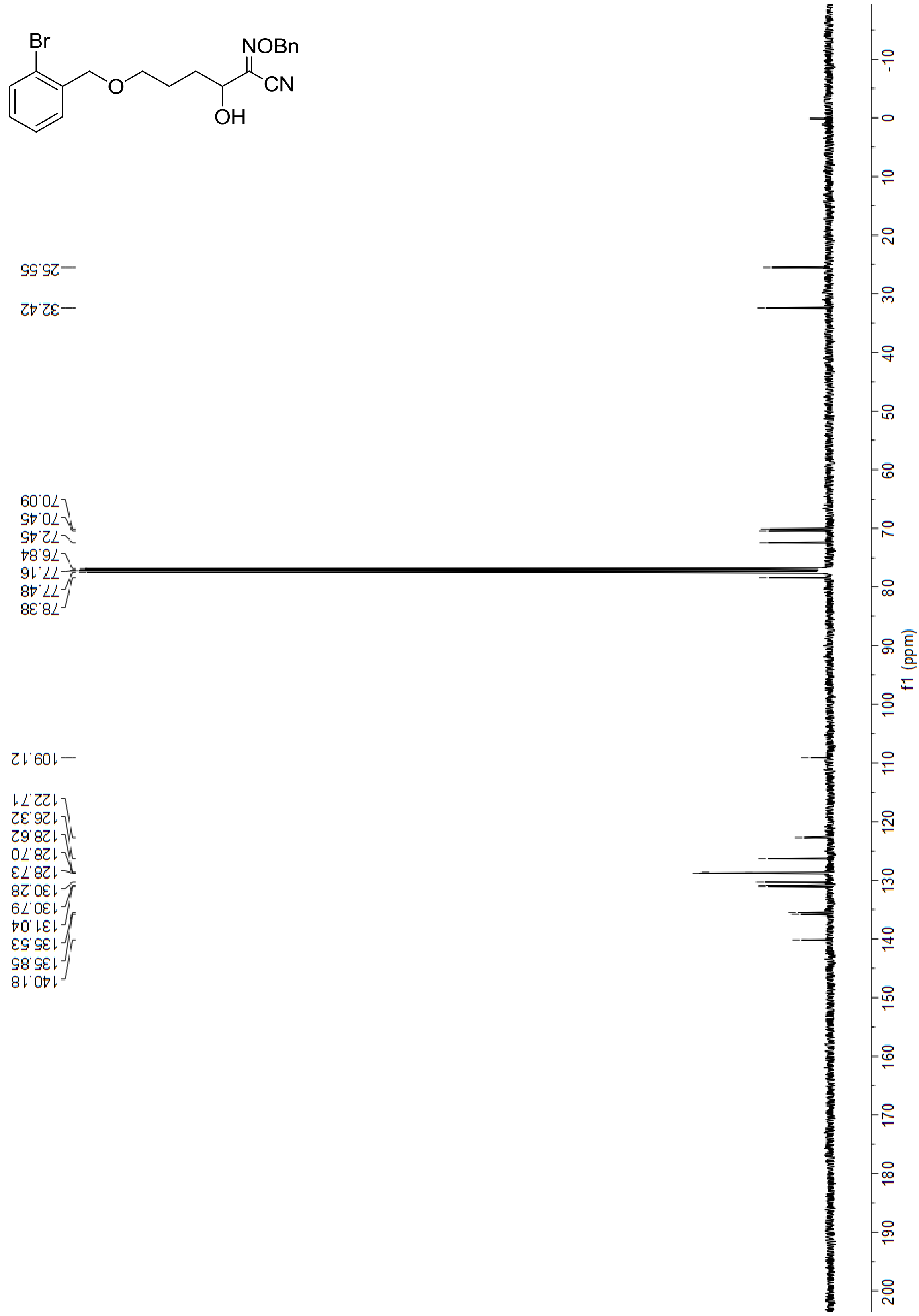

***N*-(Benzyloxy)-5-((2-cyanobenzyl)oxy)-2-hydroxypentanimidoyl cyanide (3o)**

^{1}H NMR of **3o** ($CDCl_3$, 400 MHz, 25 ºC)

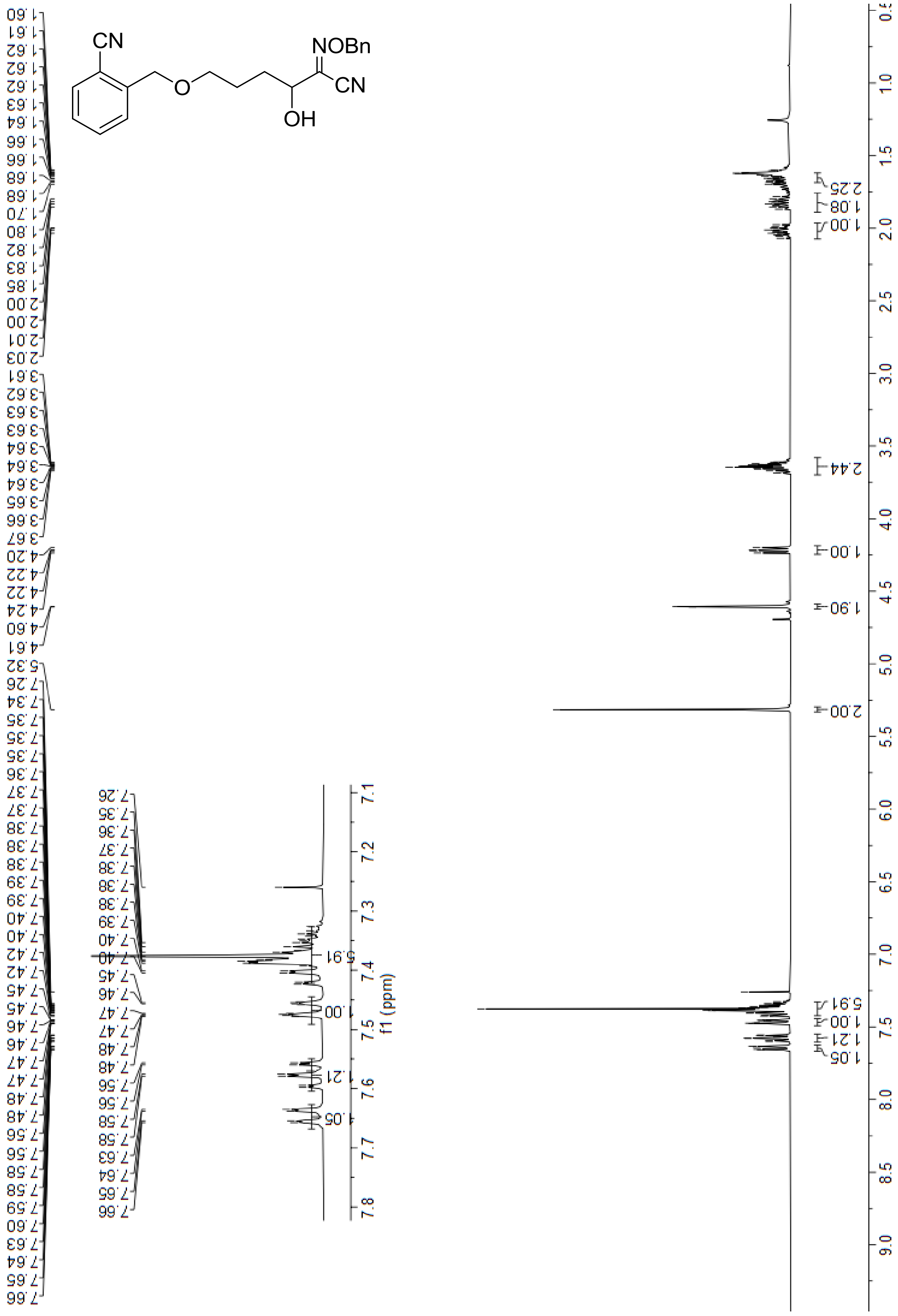

^{13}C NMR of **3o** ($CDCl_3$, 101 MHz, 25 ºC)

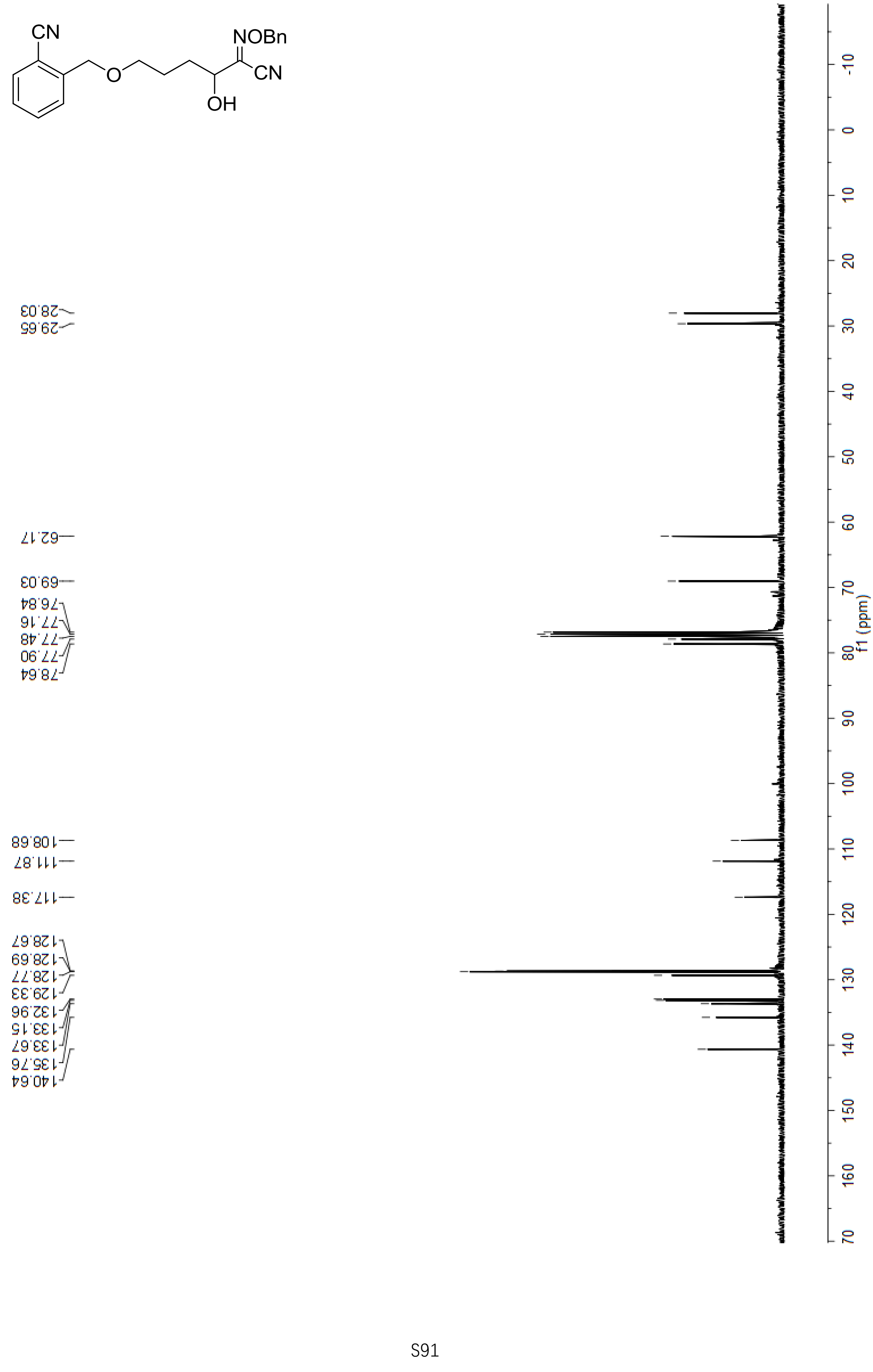

***N*-(Benzyloxy)-2-hydroxy-7-phenoxyheptanimidoyl cyanide (3p)**

^{1}H NMR of **3p** ($CDCl_3$, 400 MHz, 25 ºC)

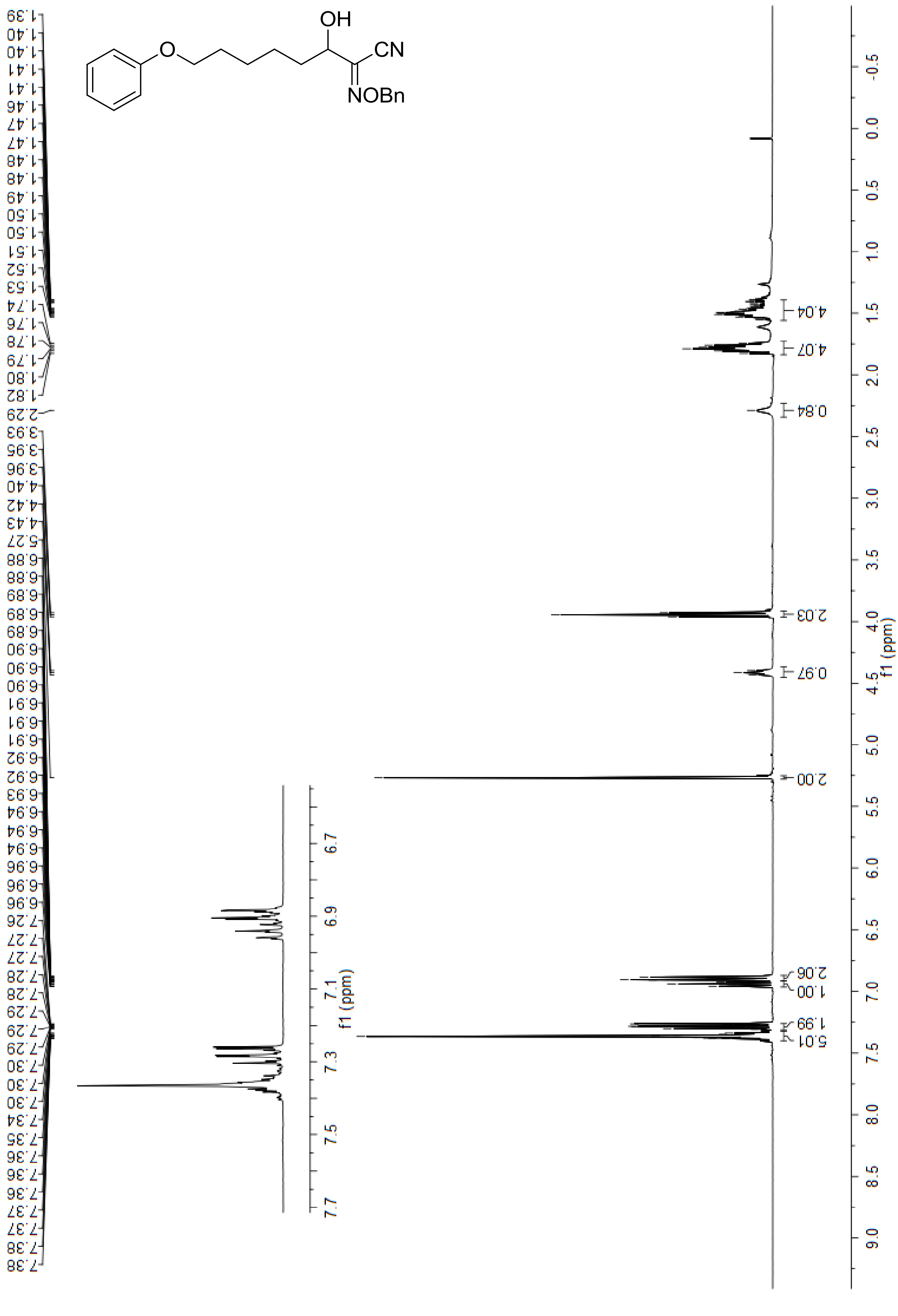

^{13}C NMR of **3p** ($CDCl_3$, 101 MHz, 25 ºC)

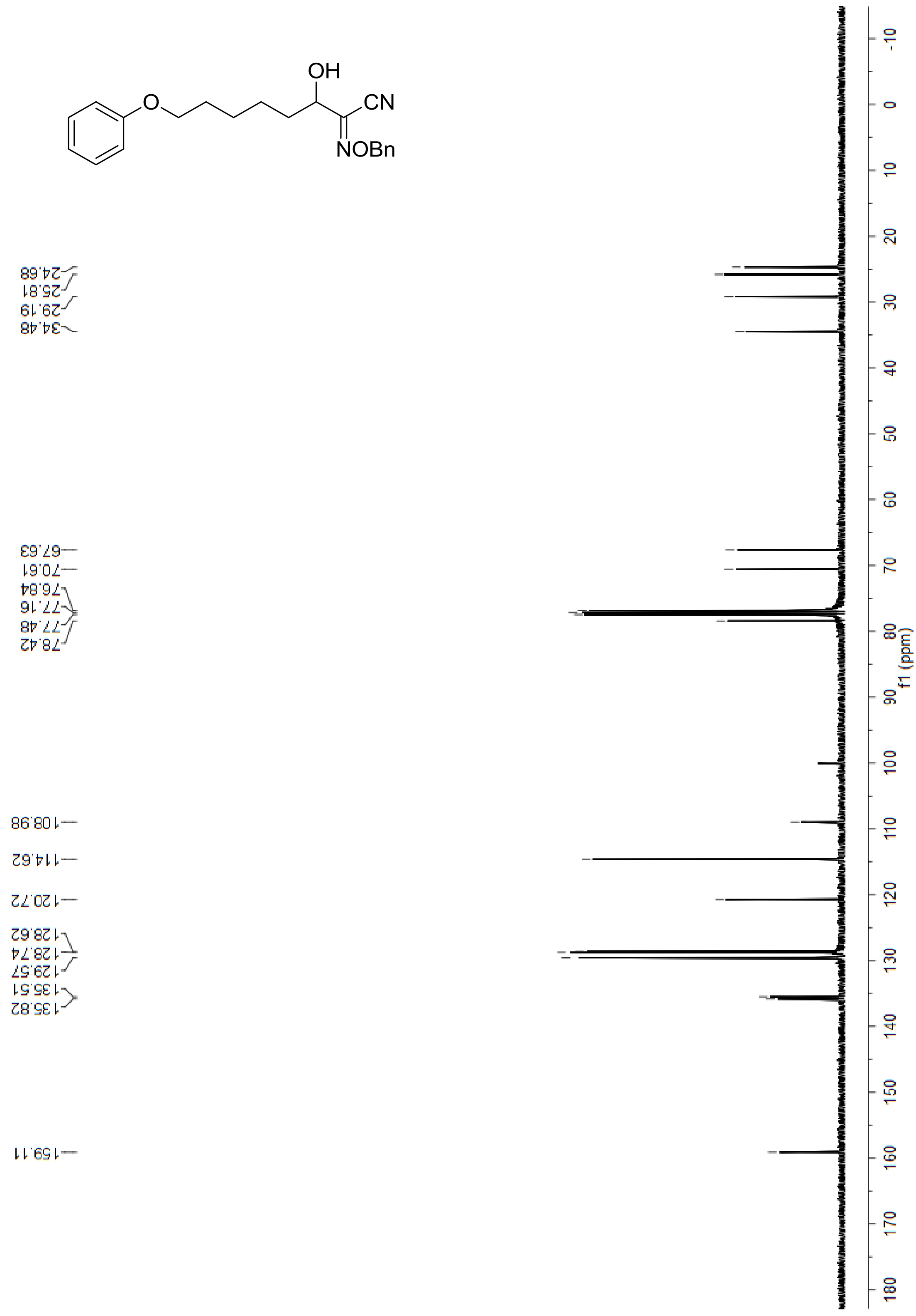

7-((Benzyloxy)imino)-7-cyano-6-hydroxyheptyl benzoate(3q)

^{1}H NMR of **3q** ($CDCl_3$, 400 MHz, 25 ºC)

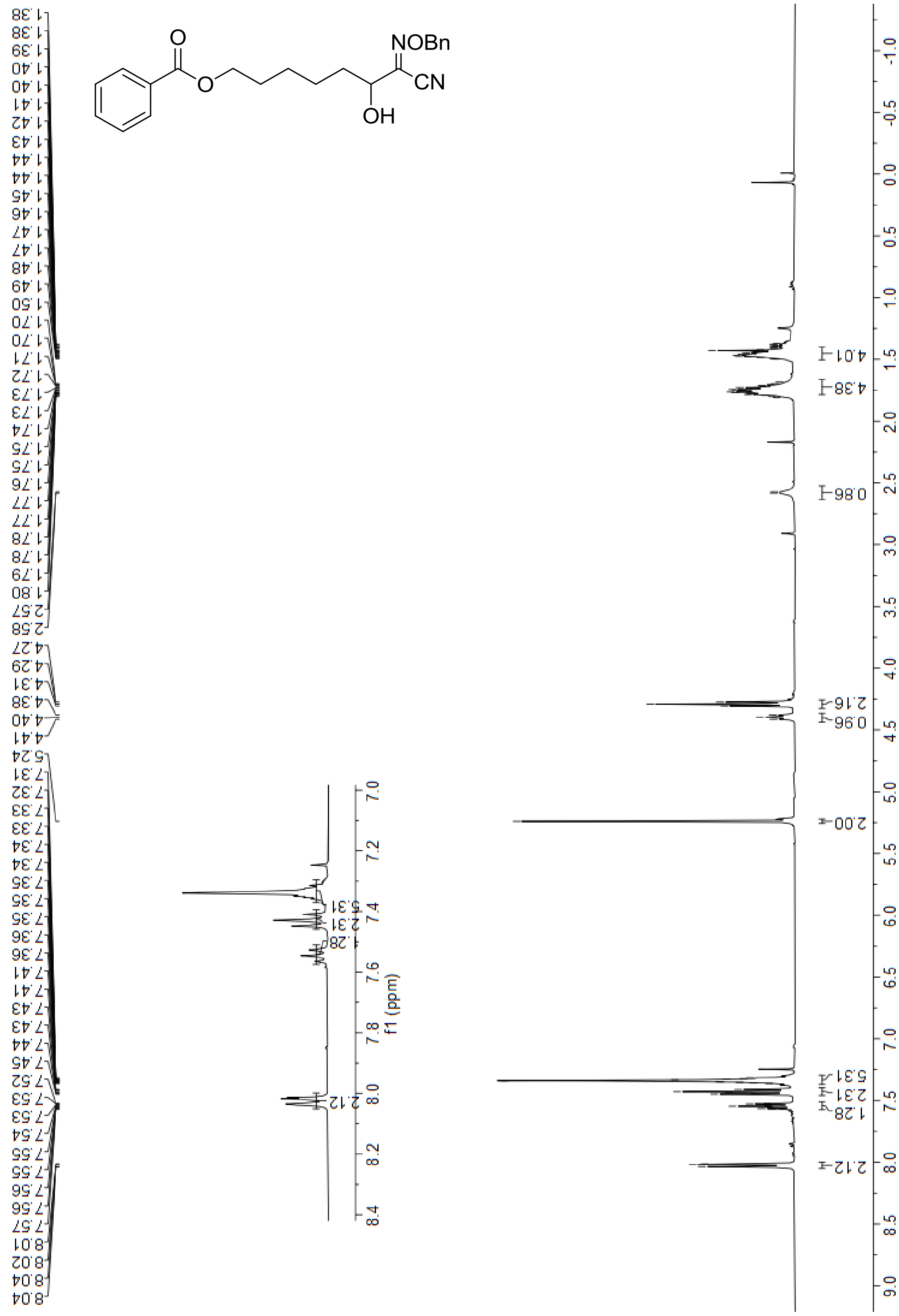

^{13}C NMR of **3q** ($CDCl_3$, 101 MHz, 25 ºC)

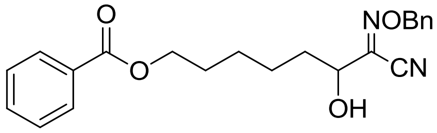

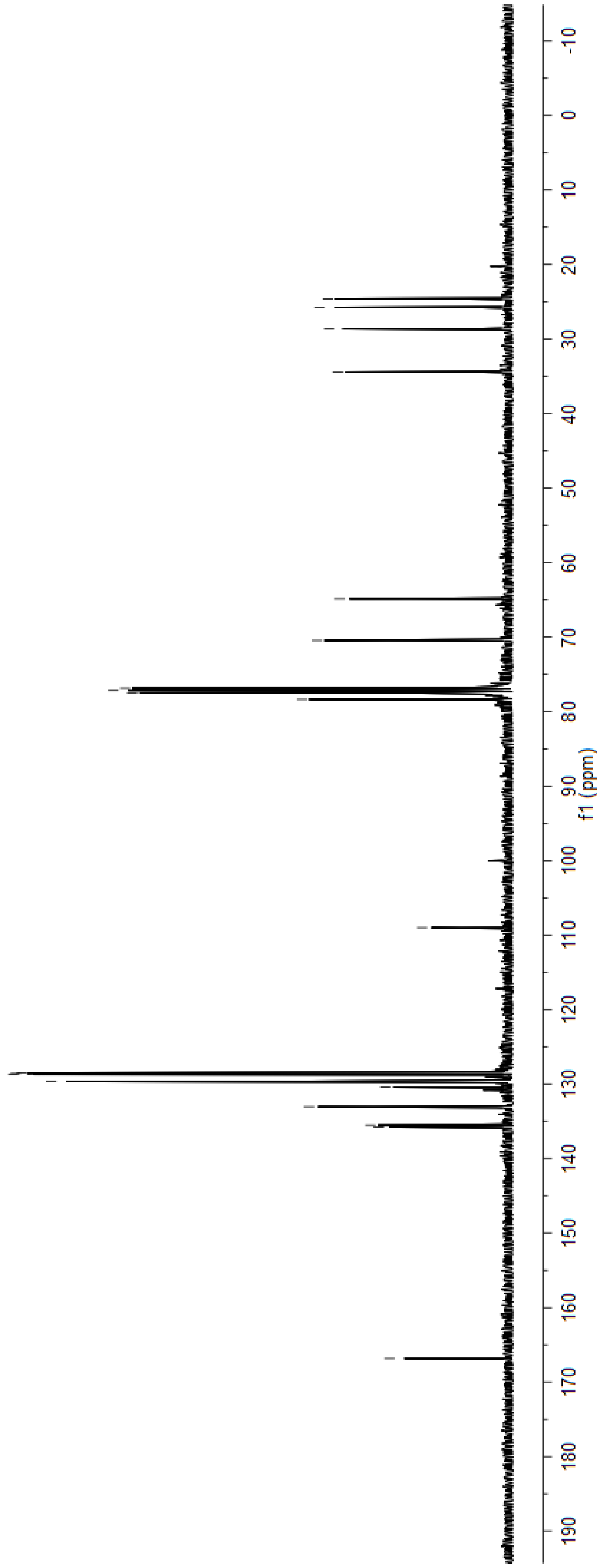

N,7-Bis(benzyloxy)-2-hydroxyheptanimidoyl cyanide (3r)

^{1}H NMR of **3r** ($CDCl_3$, 400 MHz, 25 ºC)

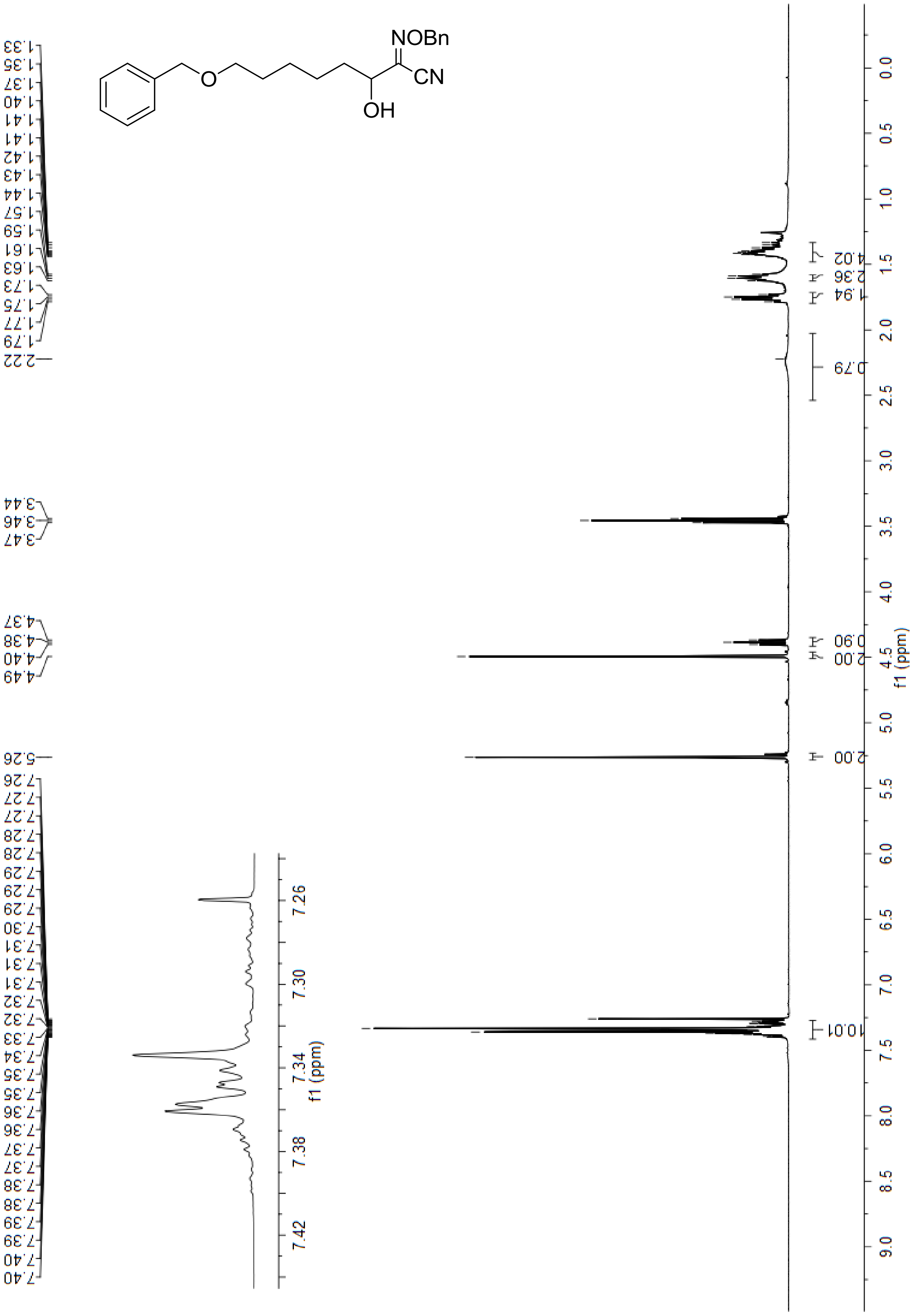

^{13}C NMR of **3r** ($CDCl_3$, 101 MHz, 25 ºC)

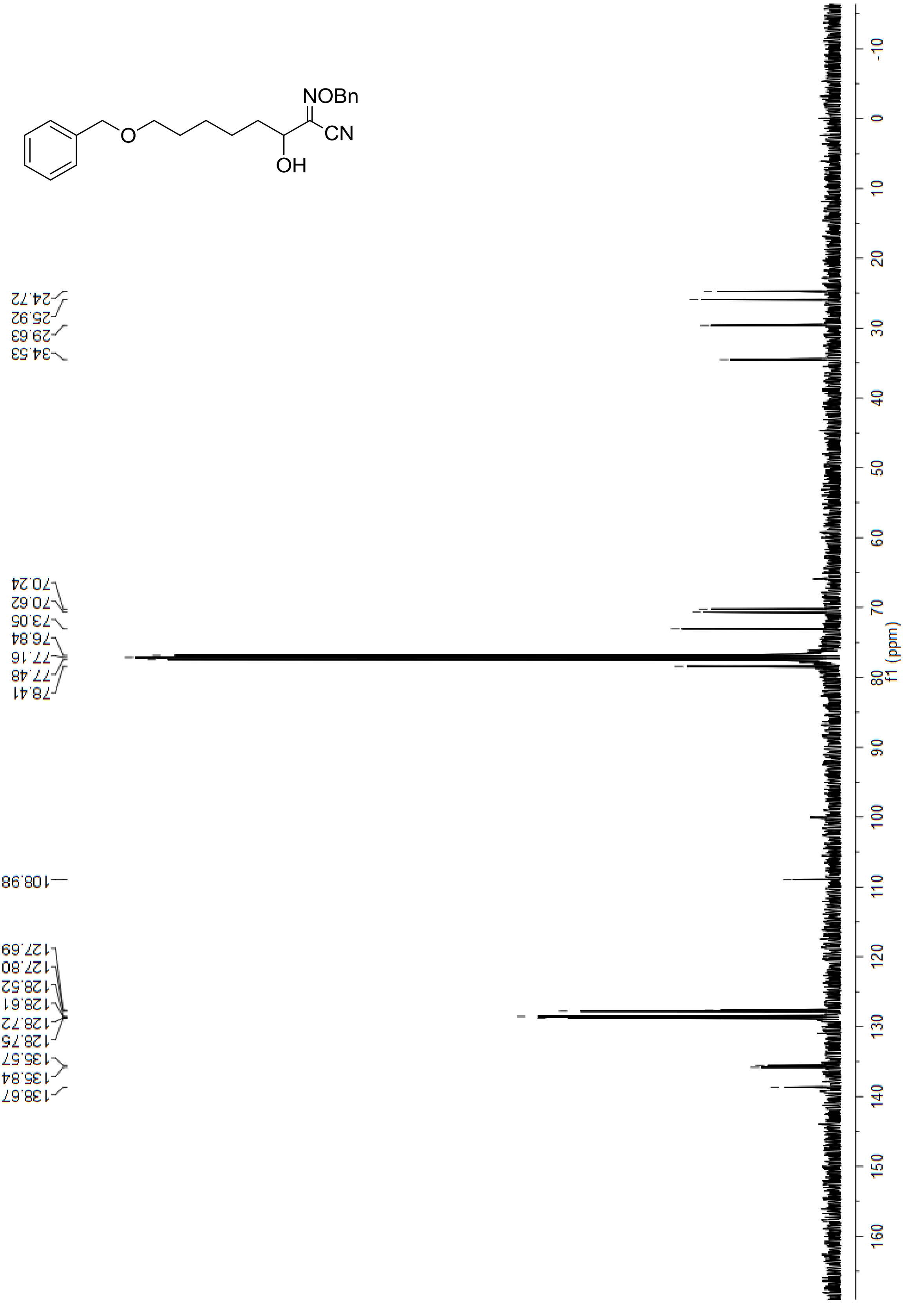

N-(benzyloxy)-2-hydroxy-5-(3-phenylpropoxy)pentanimidoyl cyanide (3s)

^{1}H NMR of **3s** ($CDCl_3$, 400 MHz, 25 ºC)

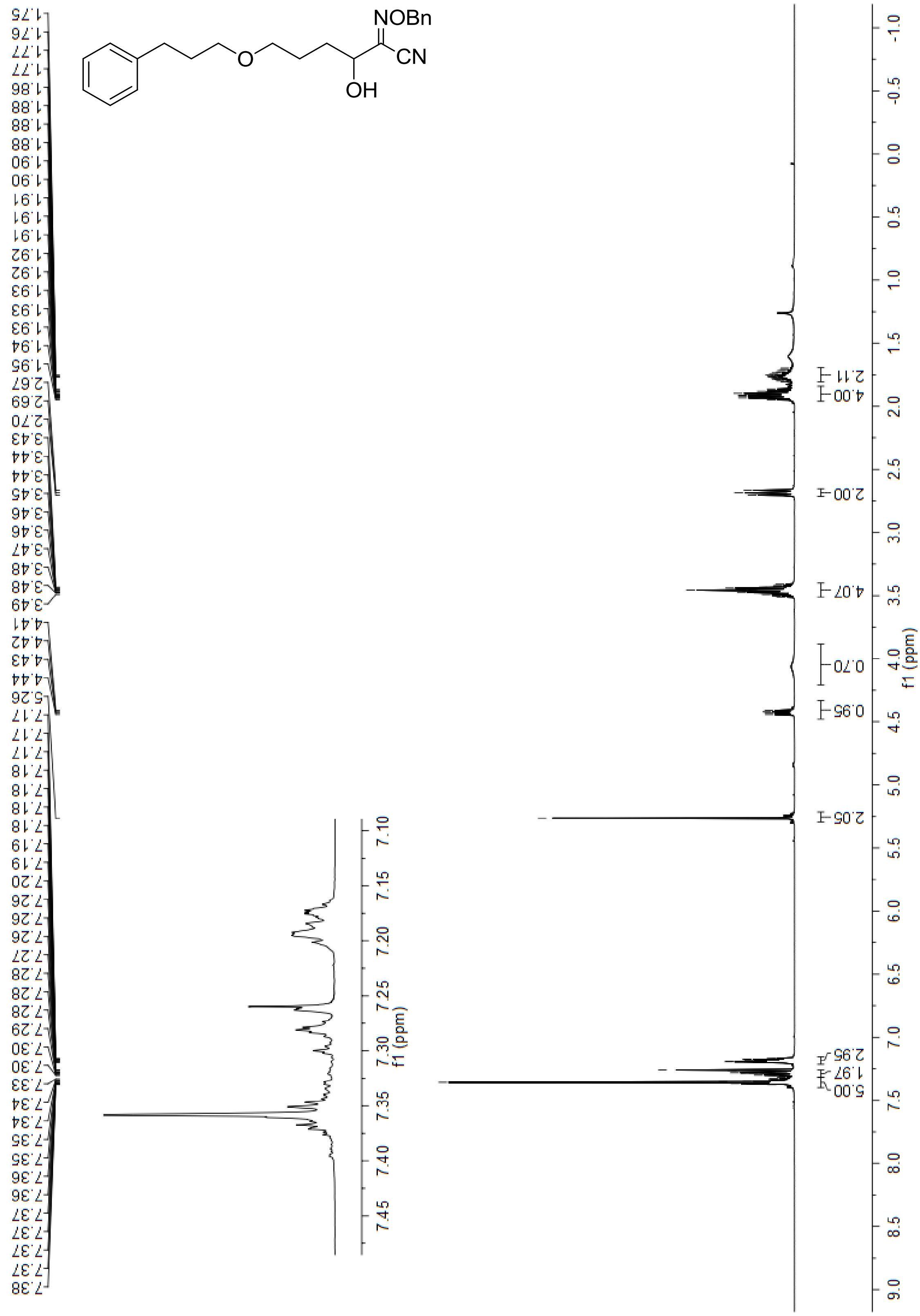

^{13}C NMR of **3s** (CDCl$_3$, 101 MHz, 25 ºC)

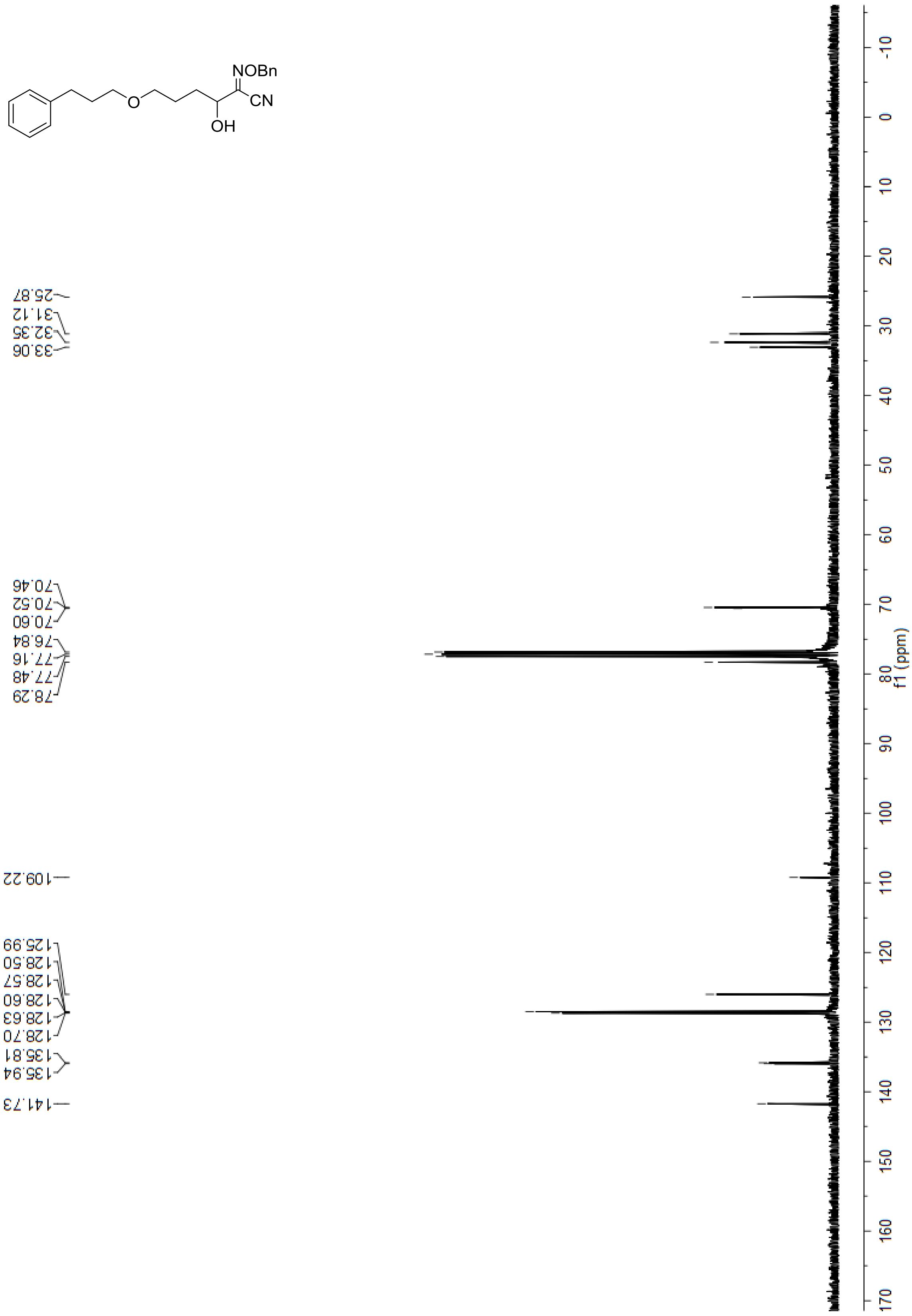

***N*-(Benzyloxy)-2-hydroxy-7-((4-methylbenzyl)oxy)heptanimidoyl cyanide (3t)**

^{1}H NMR of **3t** ($CDCl_3$, 400 MHz, 25 ºC)

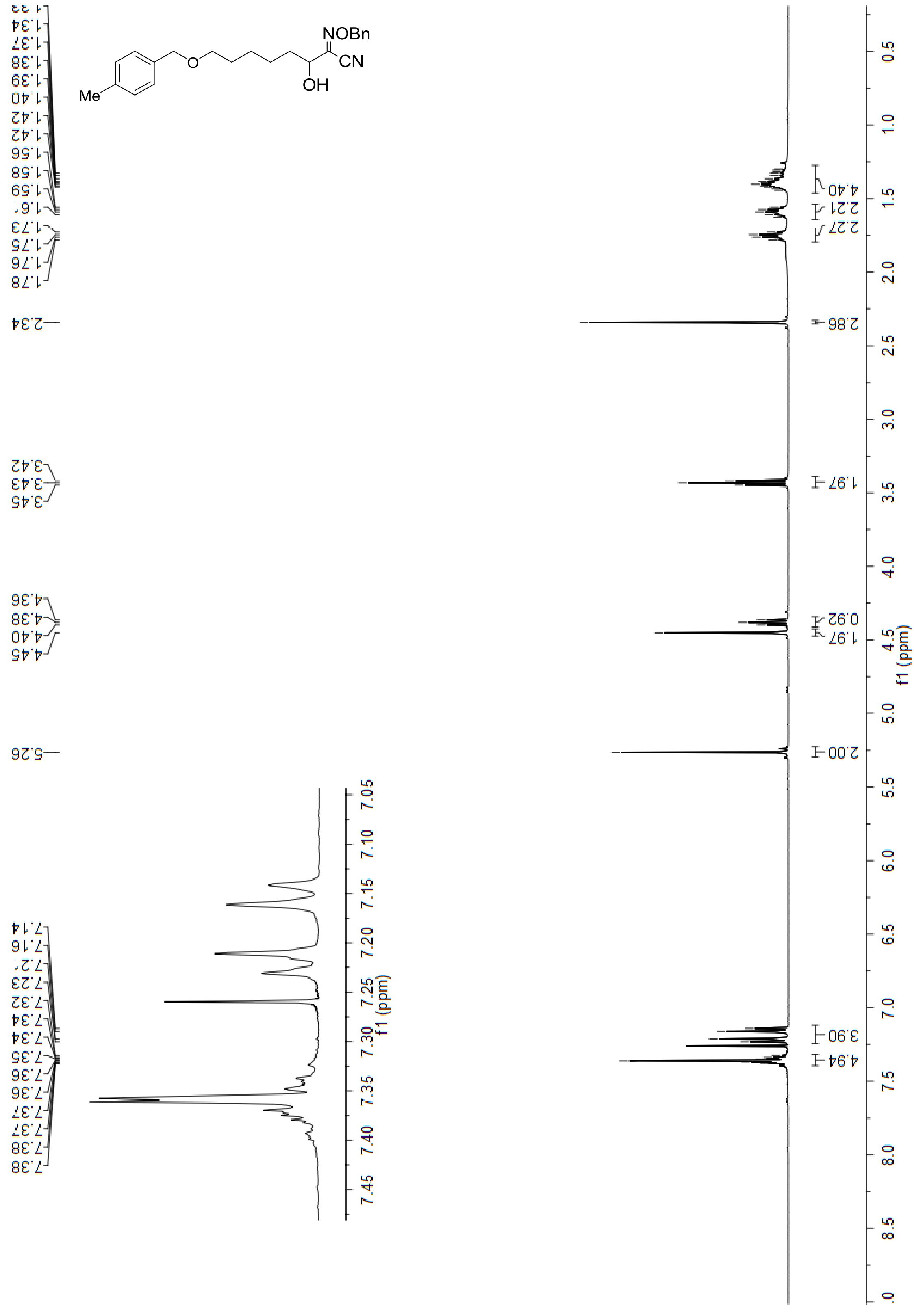

^{13}C NMR of **3t** ($CDCl_3$, 101 MHz, 25 ºC)

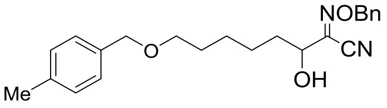

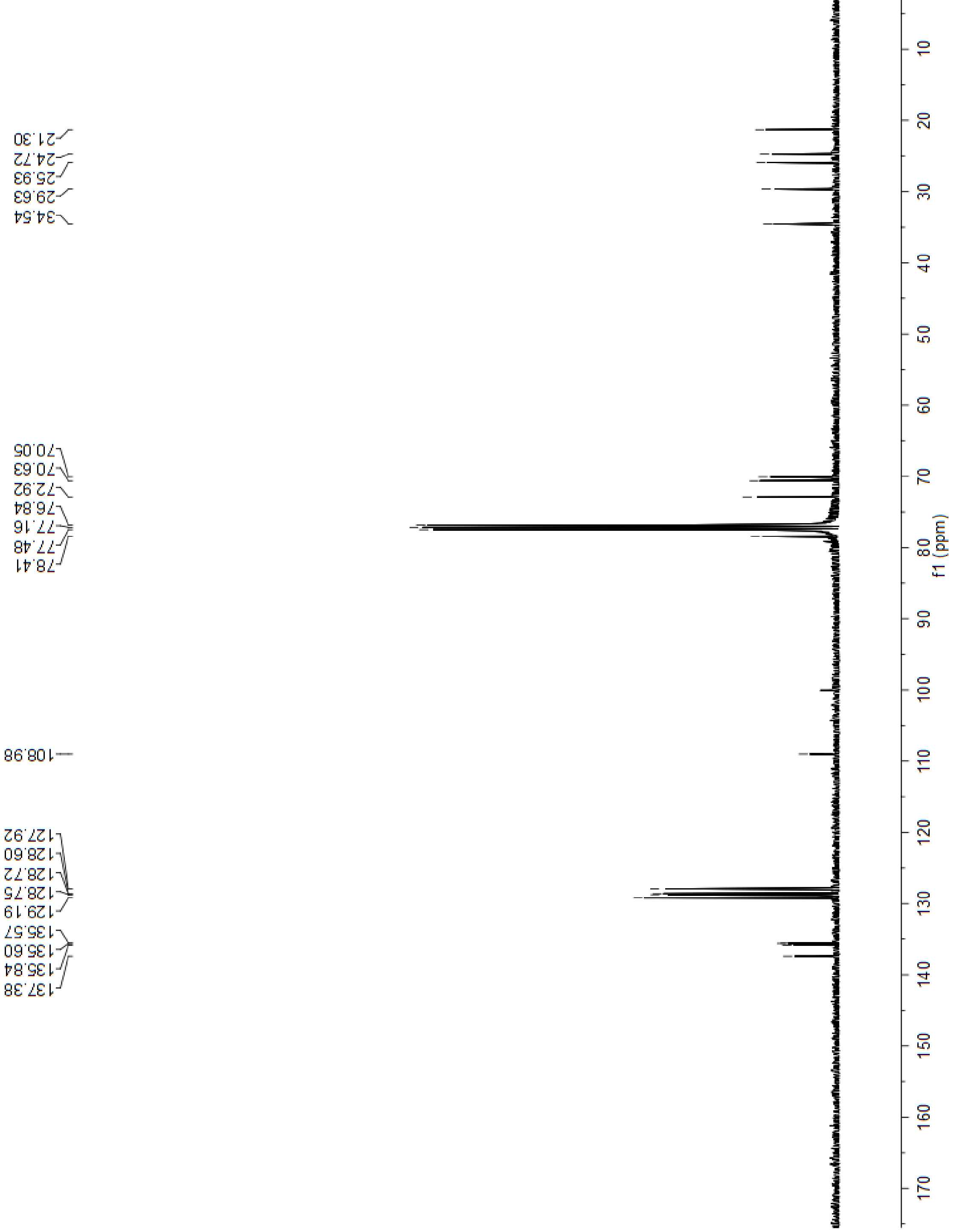

N,6-Bis(benzyloxy)-2-hydroxyhexanimidoyl cyanide (3u)

^{1}H NMR of **3u** ($CDCl_3$, 400 MHz, 25 ºC)

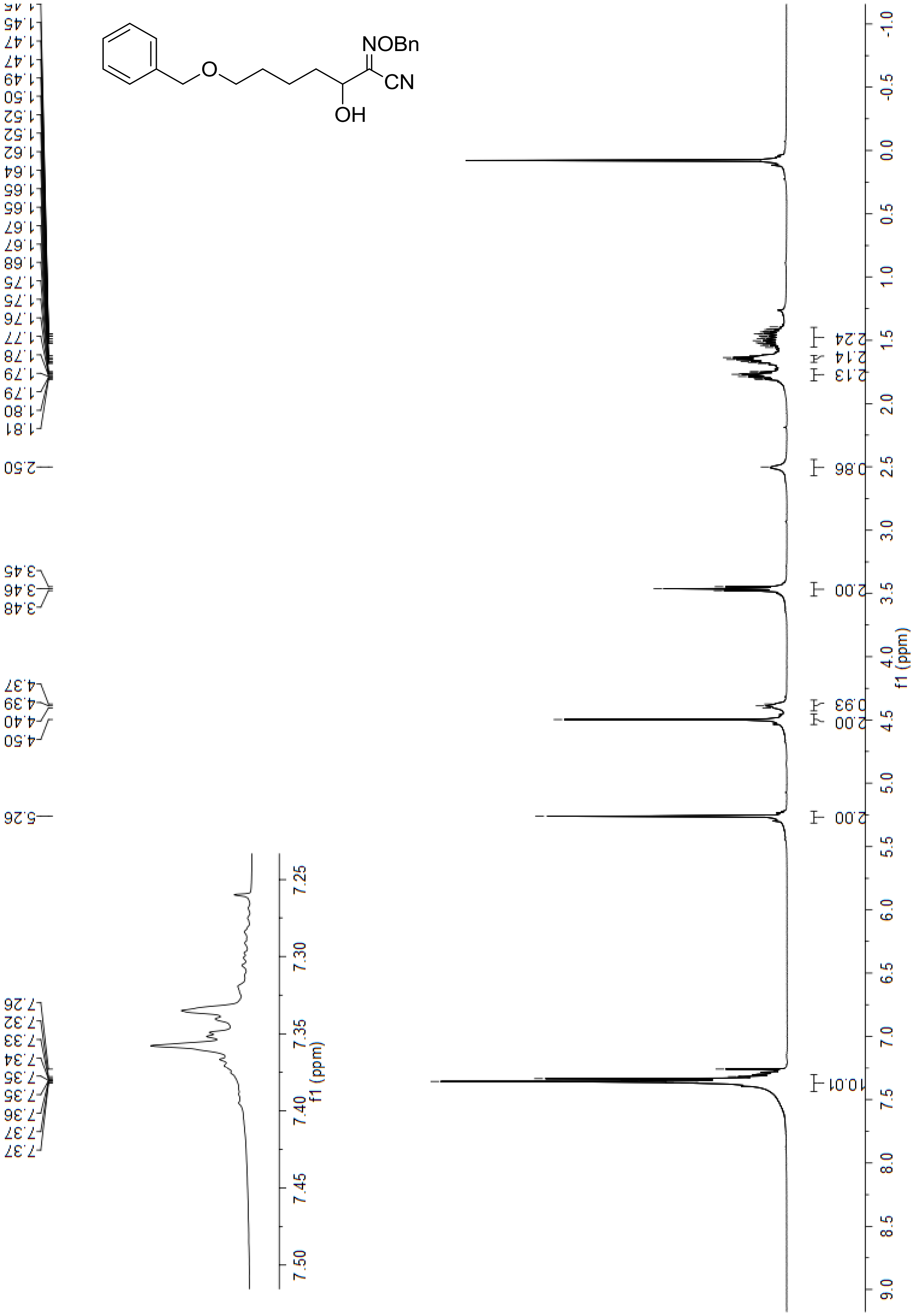

^{13}C NMR of **3u** ($CDCl_3$, 101 MHz, 25 ºC)

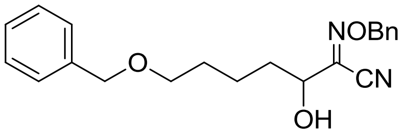

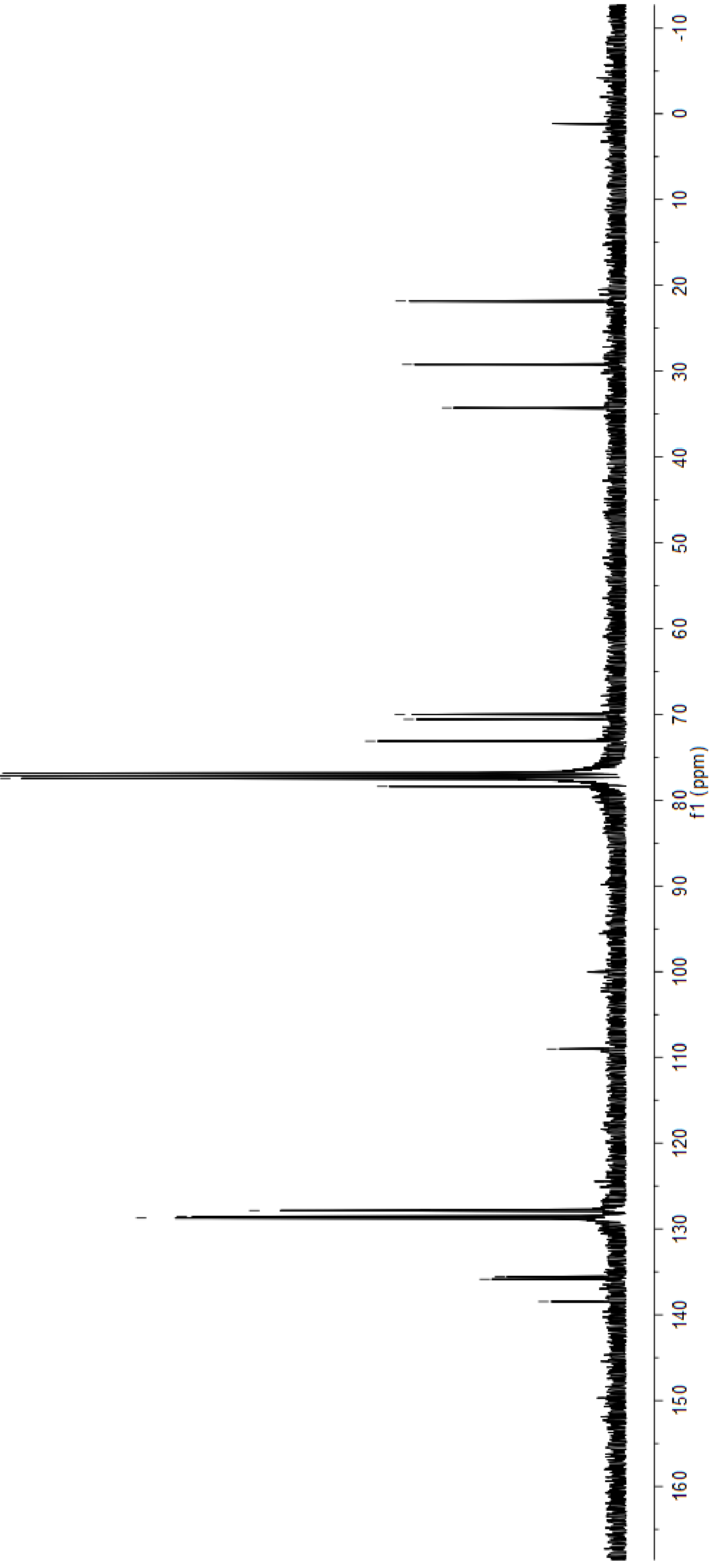

***N*-(Benzyloxy)-2-hydroxy-5-(isopentyloxy)pentanimidoyl cyanide(3v)**

^{1}H NMR of **3v** ($CDCl_3$, 400 MHz, 25 ºC)

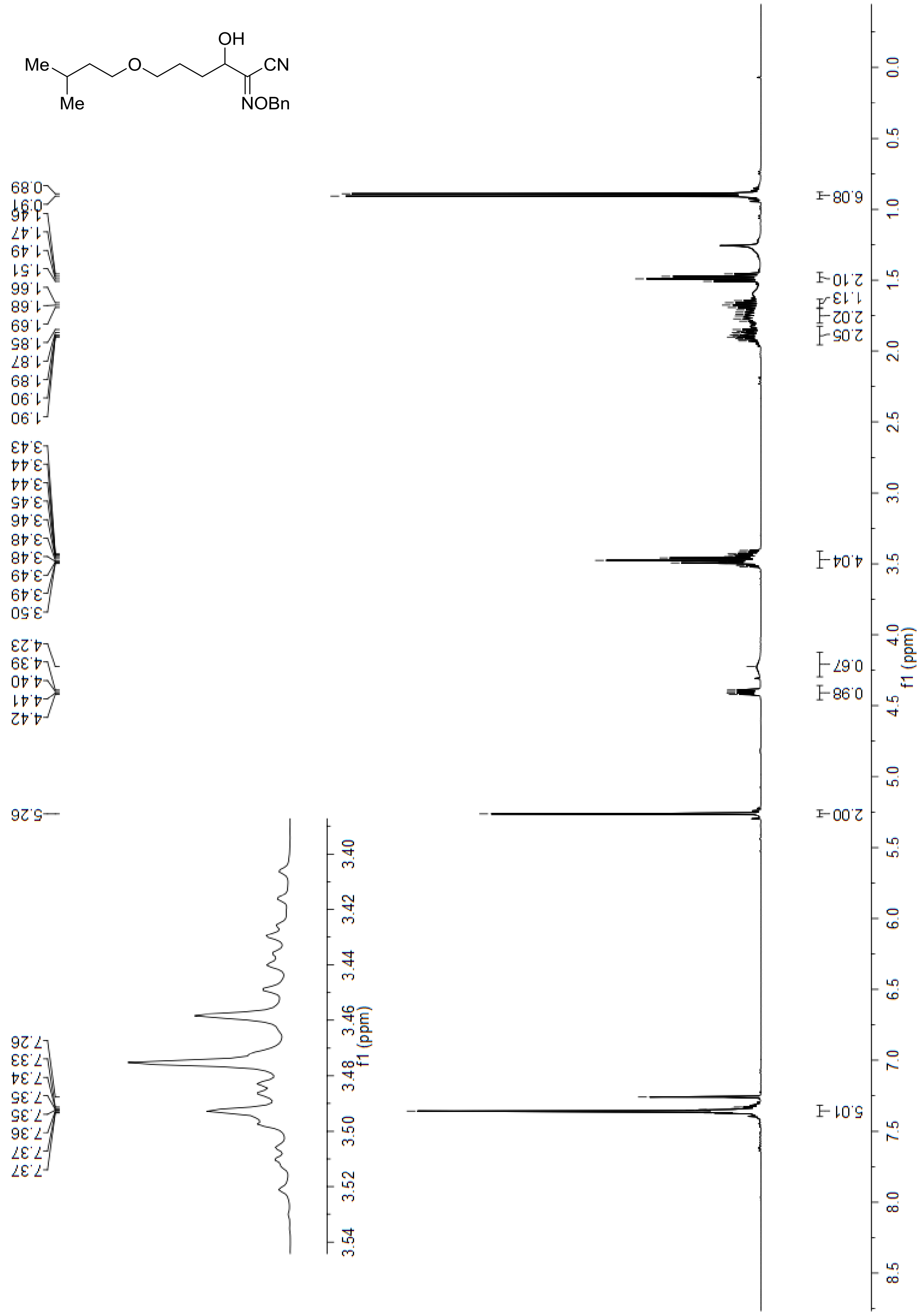

^{13}C NMR of **3v** ($CDCl_3$, 101 MHz, 25 ºC)

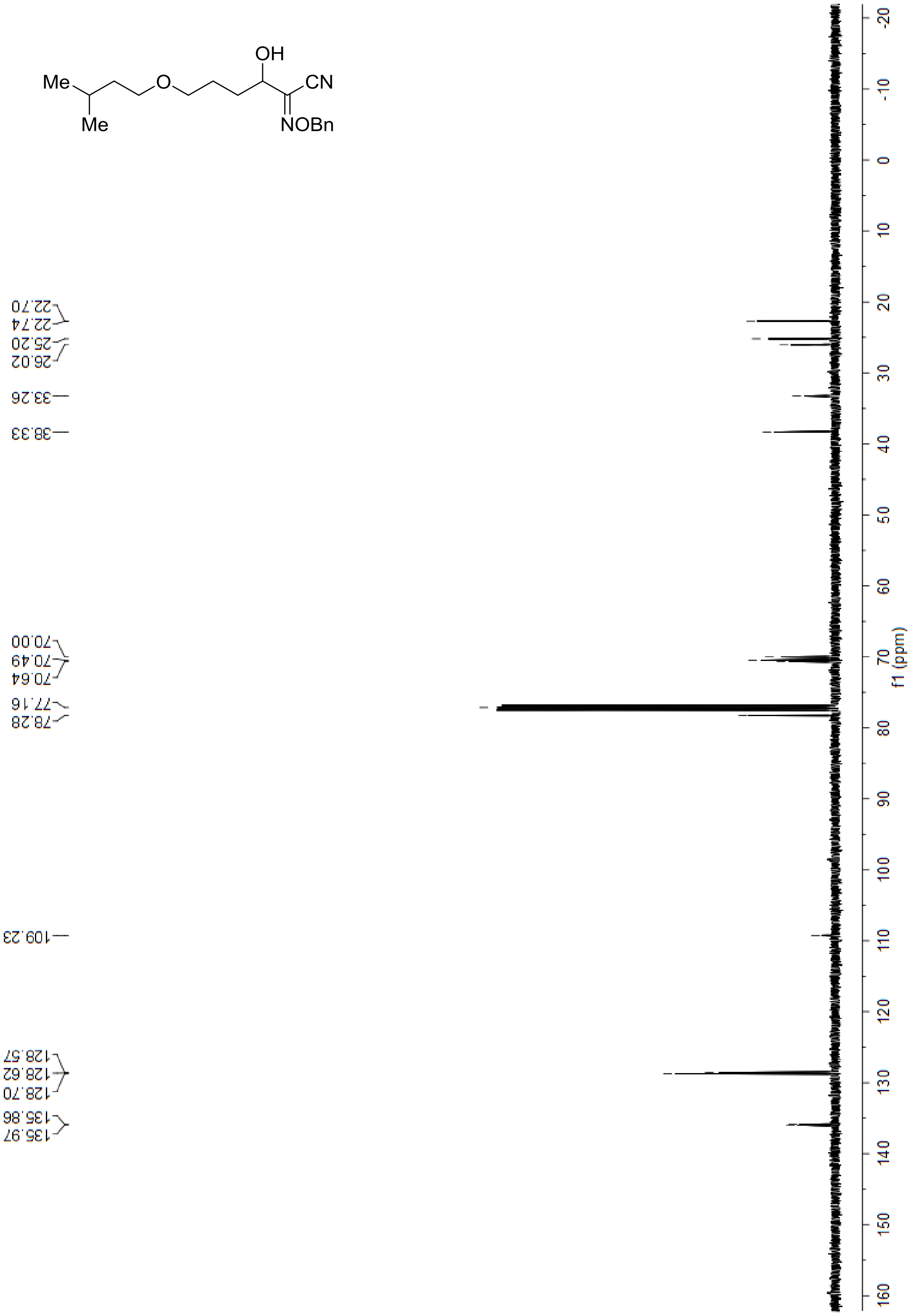

***N*-(Benzyloxy)-2-hydroxy-7-((4-(trifluoromethoxy)benzyl)oxy)heptanimidoyl cyanide (3w)**

^{1}H NMR of **3w** ($CDCl_3$, 400 MHz, 25 ºC)

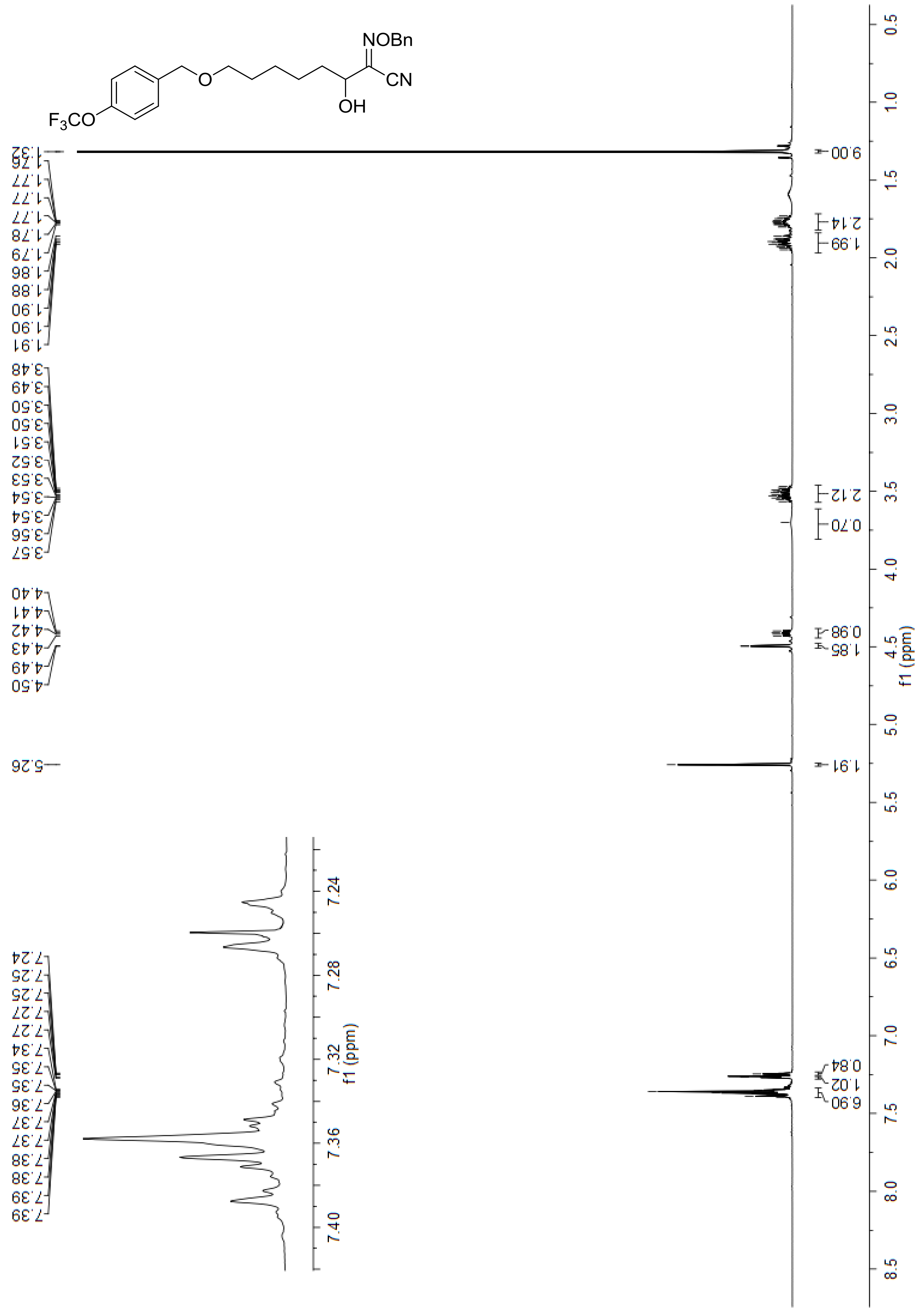

^{13}C NMR of **3w** ($CDCl_3$, 101 MHz, 25 ºC)

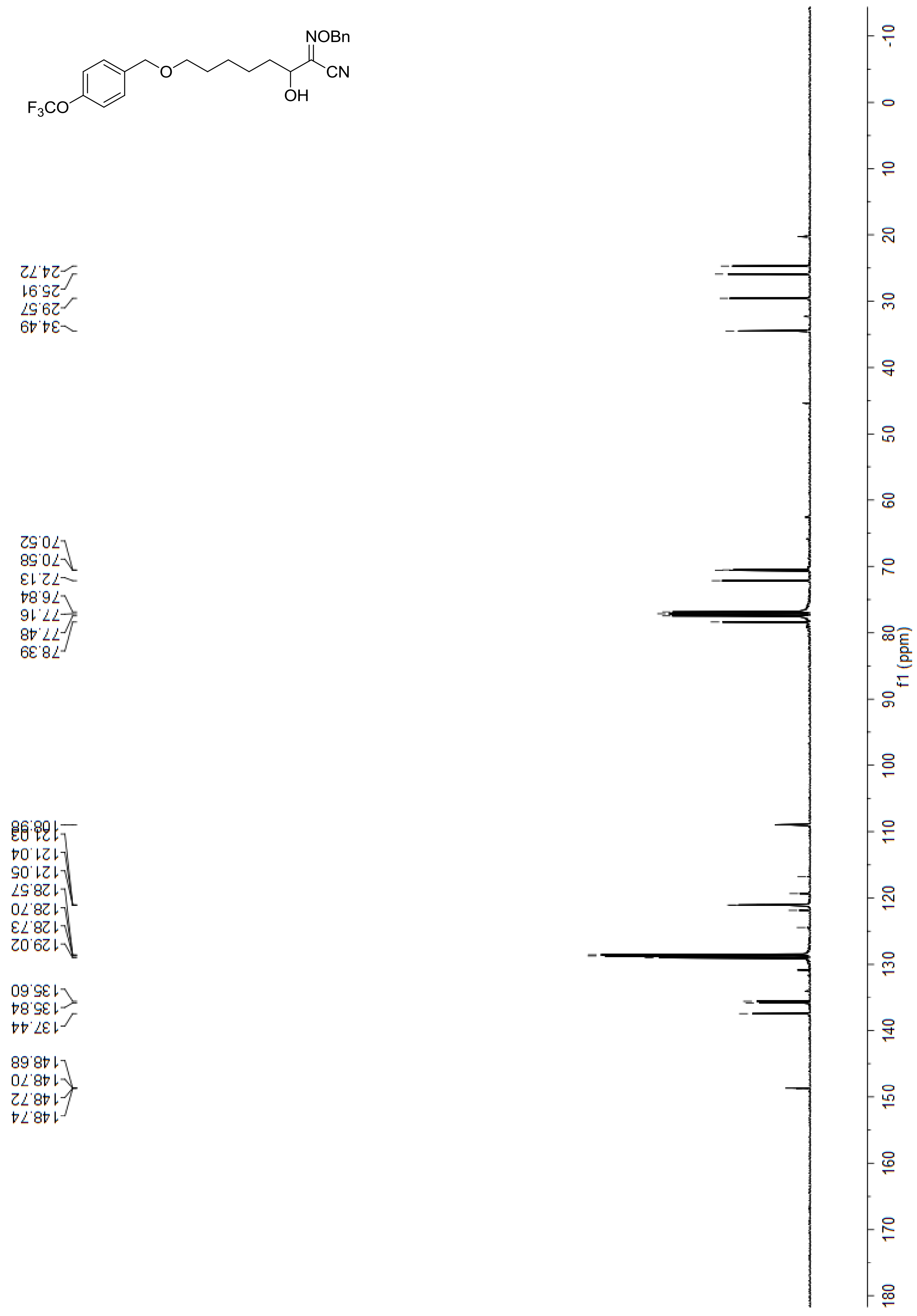

^{19}F NMR of **3w** ($CDCl_3$, 375 MHz, 25 ºC)

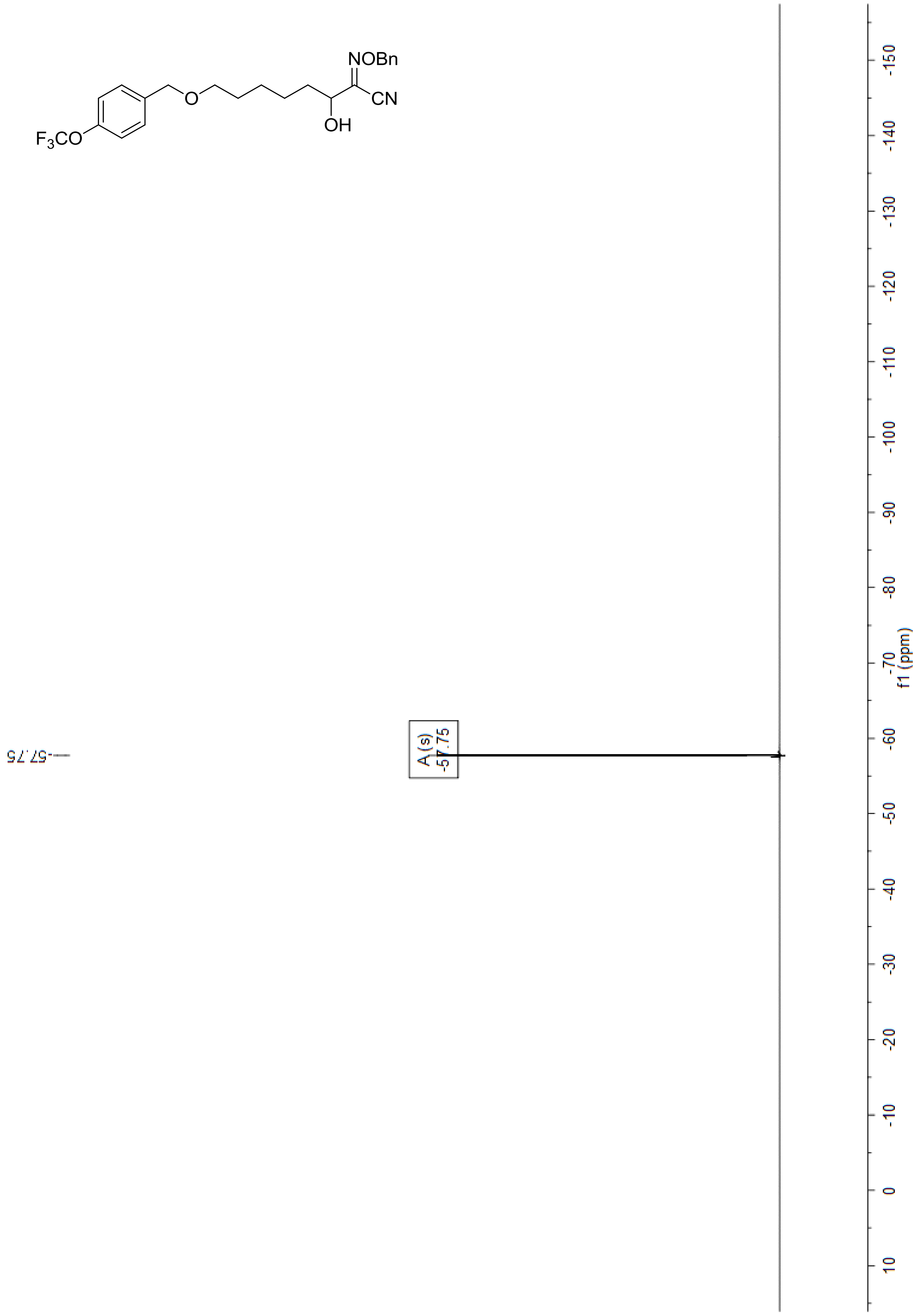

N-(Benzyloxy)-5-(2-(benzyloxy)ethoxy)-2-hydroxypentanimidoyl cyanide (3x)

^{1}H NMR of **3x** ($CDCl_3$, 400 MHz, 25 ºC)

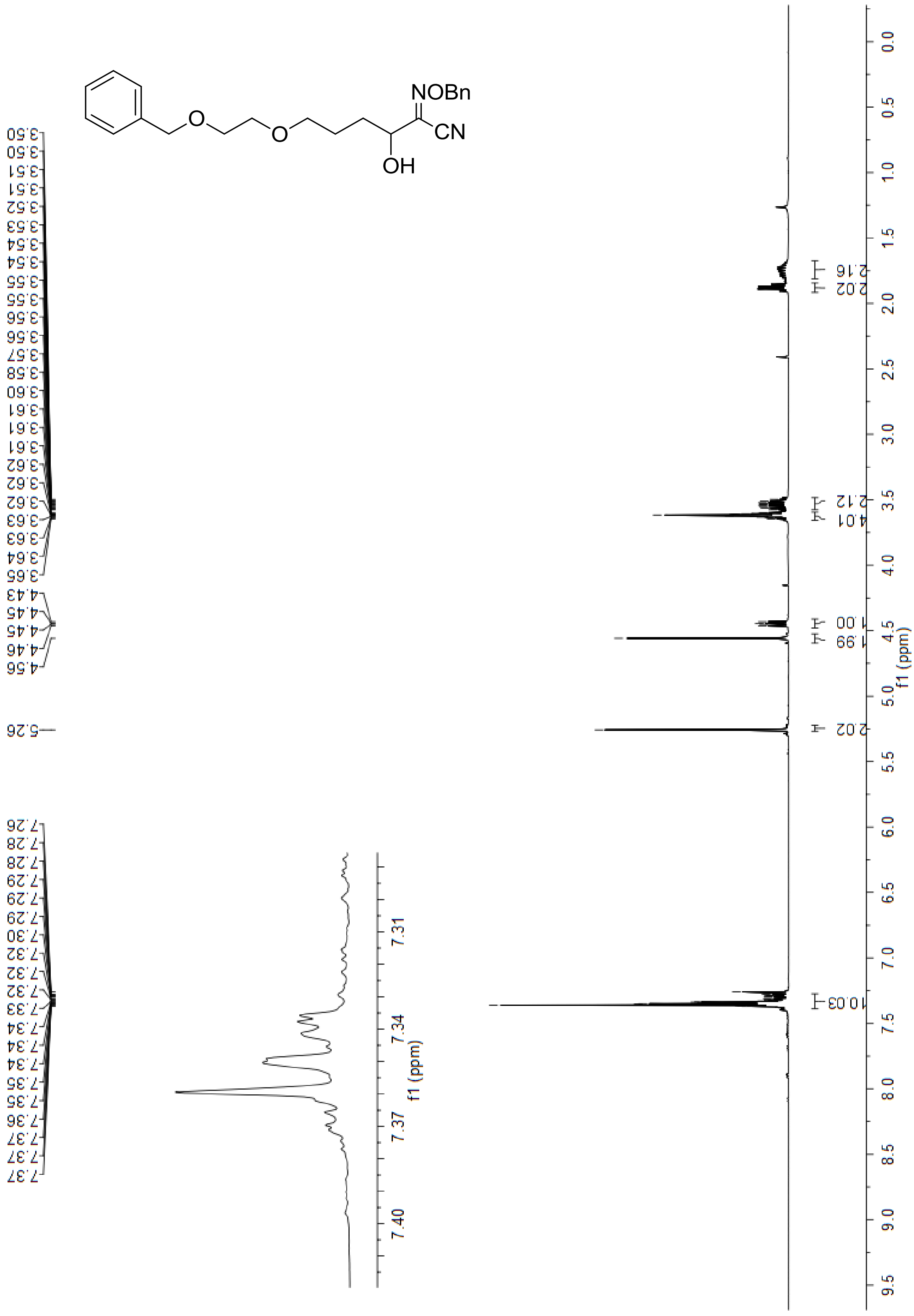

^{13}C NMR of **3x** ($CDCl_3$, 101 MHz, 25 ºC)

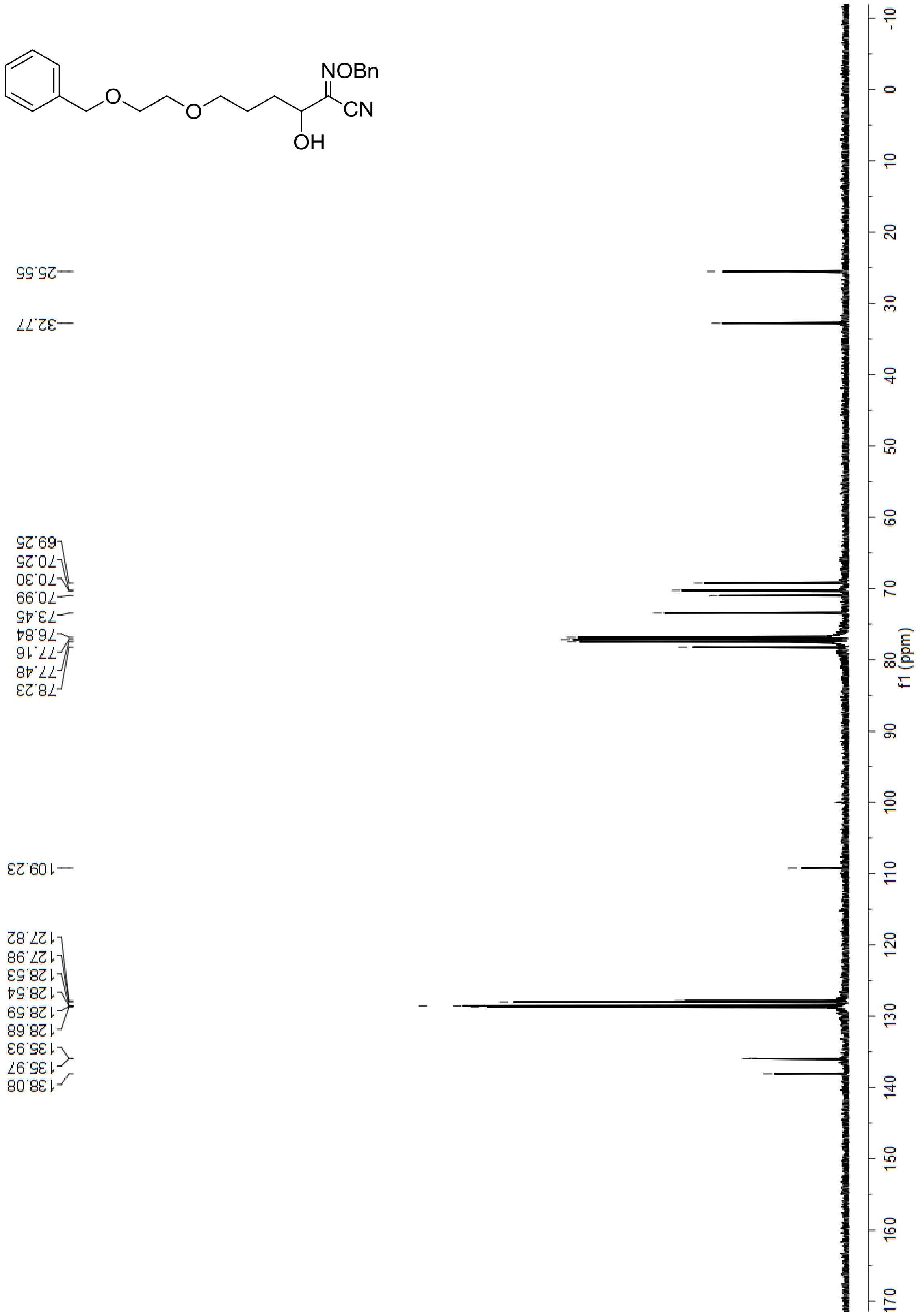

N-(benzyloxy)-2-hydroxy-3-methylpentanimidoyl cyanide (3y)

^{1}H NMR of **3y** ($CDCl_3$, 400 MHz, 25 ºC)

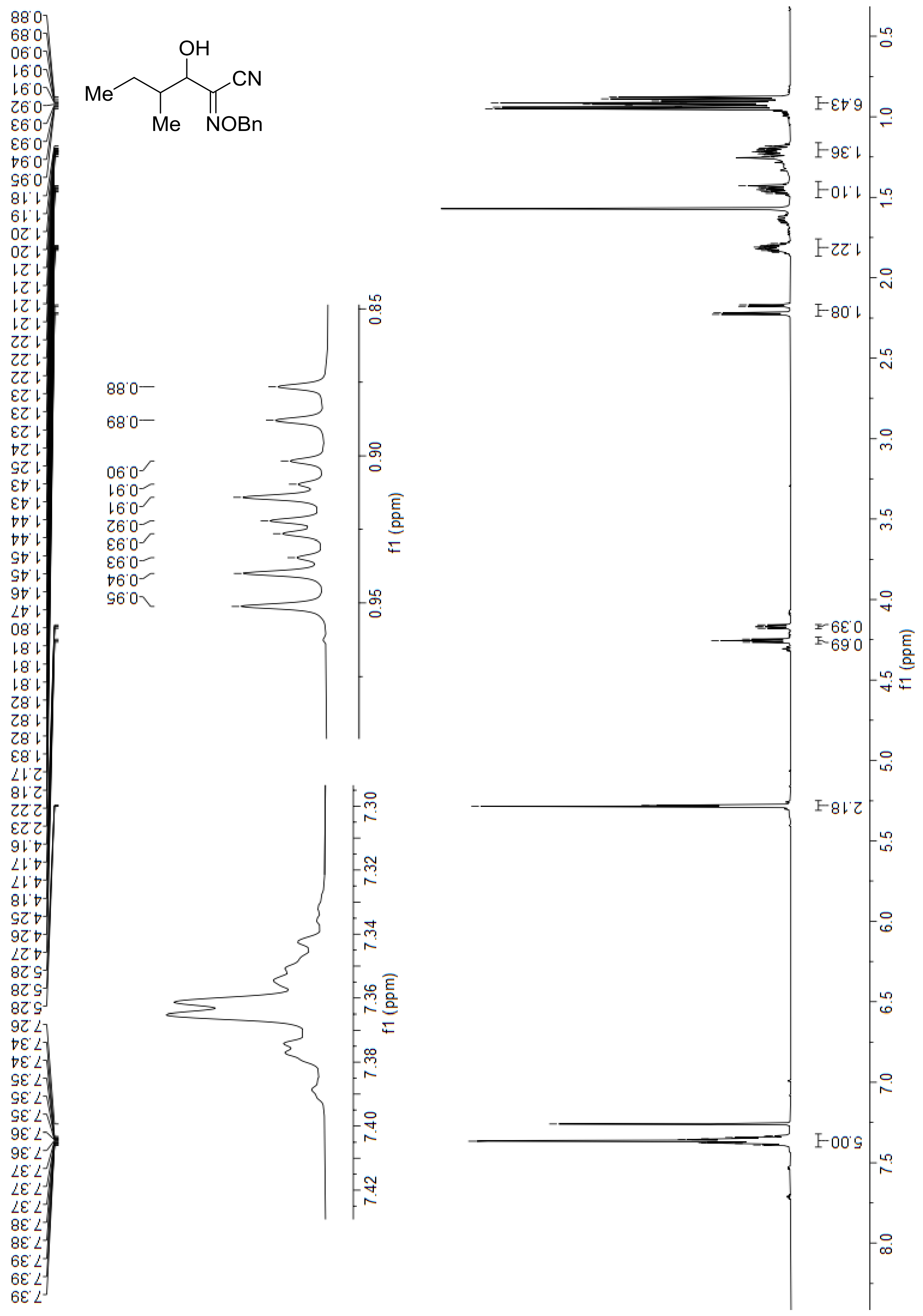

***N*-(benzyloxy)-2-hydroxy-3-methylpentanimidoyl cyanide (3y)**

^{13}C NMR of **3y** ($CDCl_3$, 151 MHz, 25 ºC)

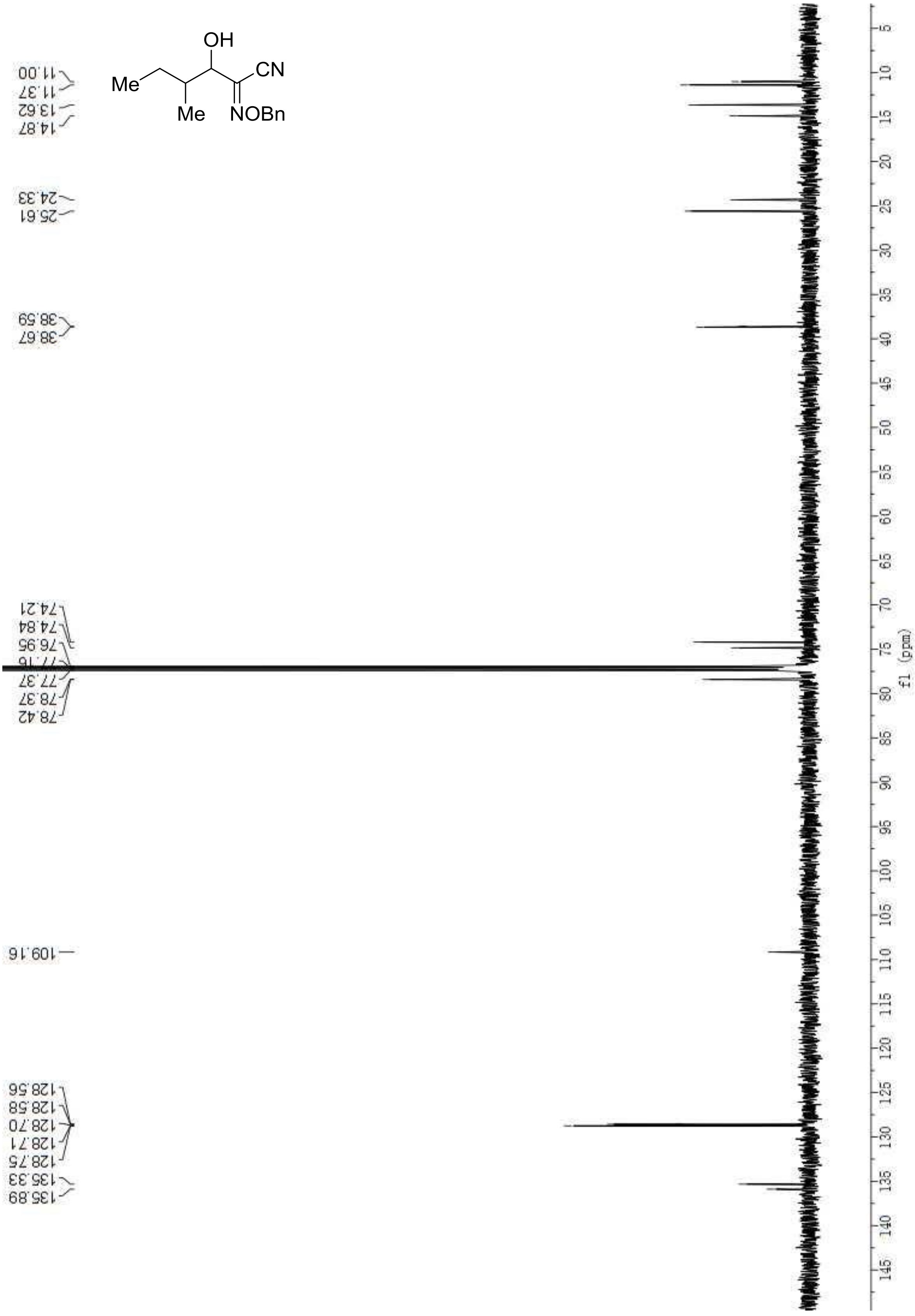

N-(Benzyloxy)-2-ethyl-2-hydroxyhexanimidoyl cyanide (3z)

^{1}H NMR of **3z** ($CDCl_3$, 400 MHz, 25 ºC)

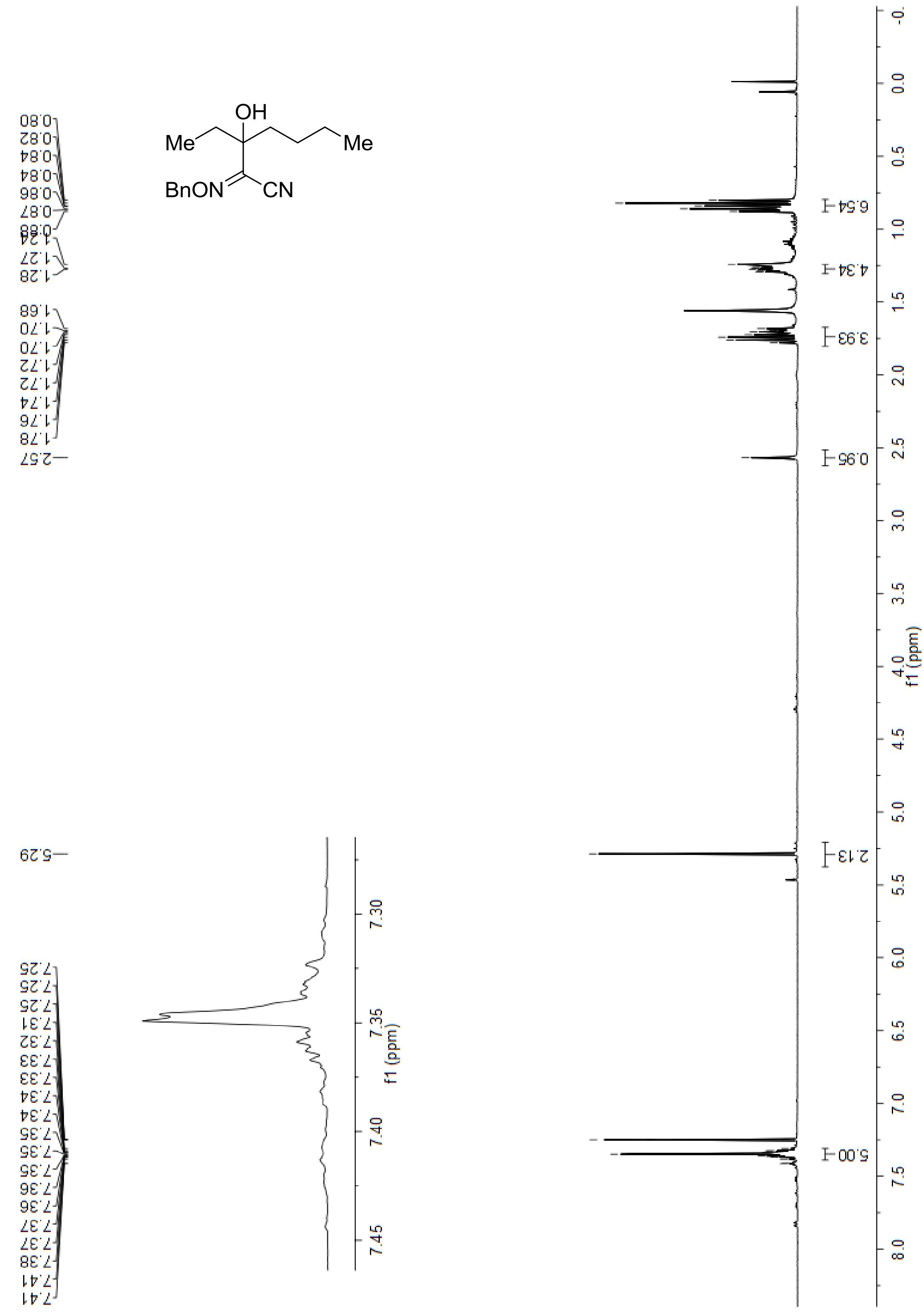

^{13}C NMR of **3z** ($CDCl_3$, 151 MHz, 25 ºC)

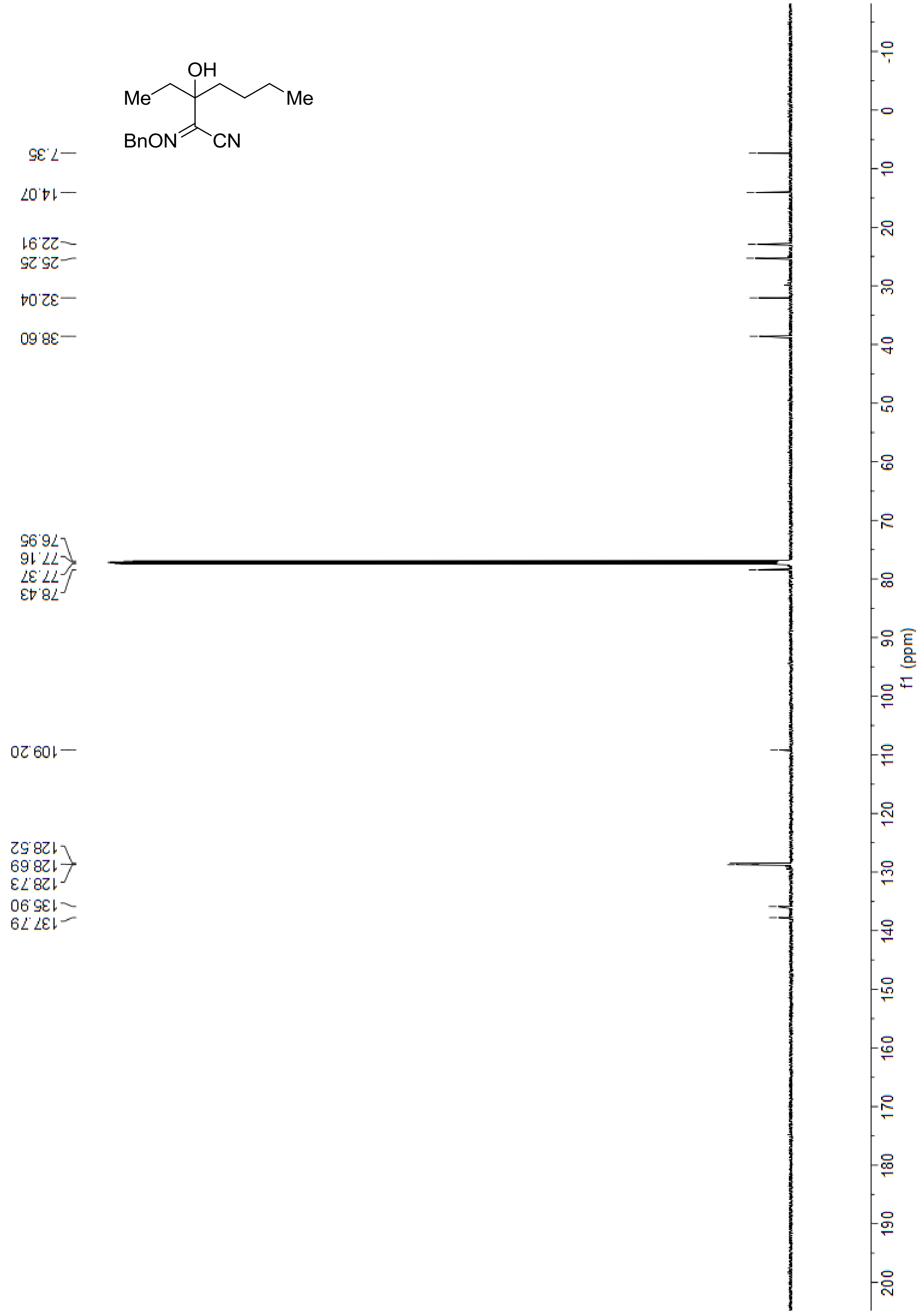

N-(benzyloxy)-2-hydroxy-2,5-dimethylhexanimidoyl cyanide (3aa)

^{1}H NMR of **3aa** ($CDCl_3$, 400 MHz, 25 ºC)

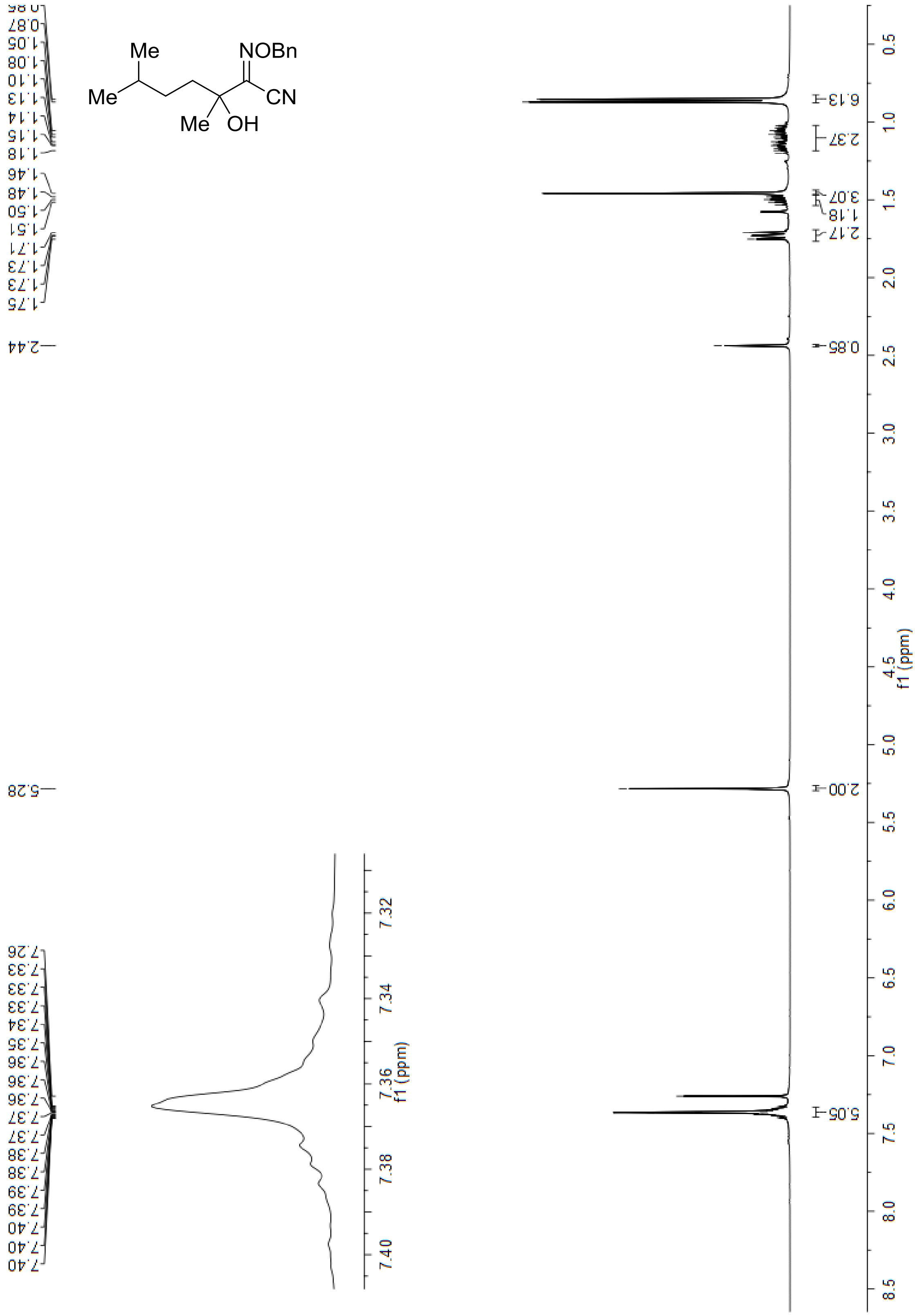

^{13}C NMR of **3aa** ($CDCl_3$, 151 MHz, 25 ºC)

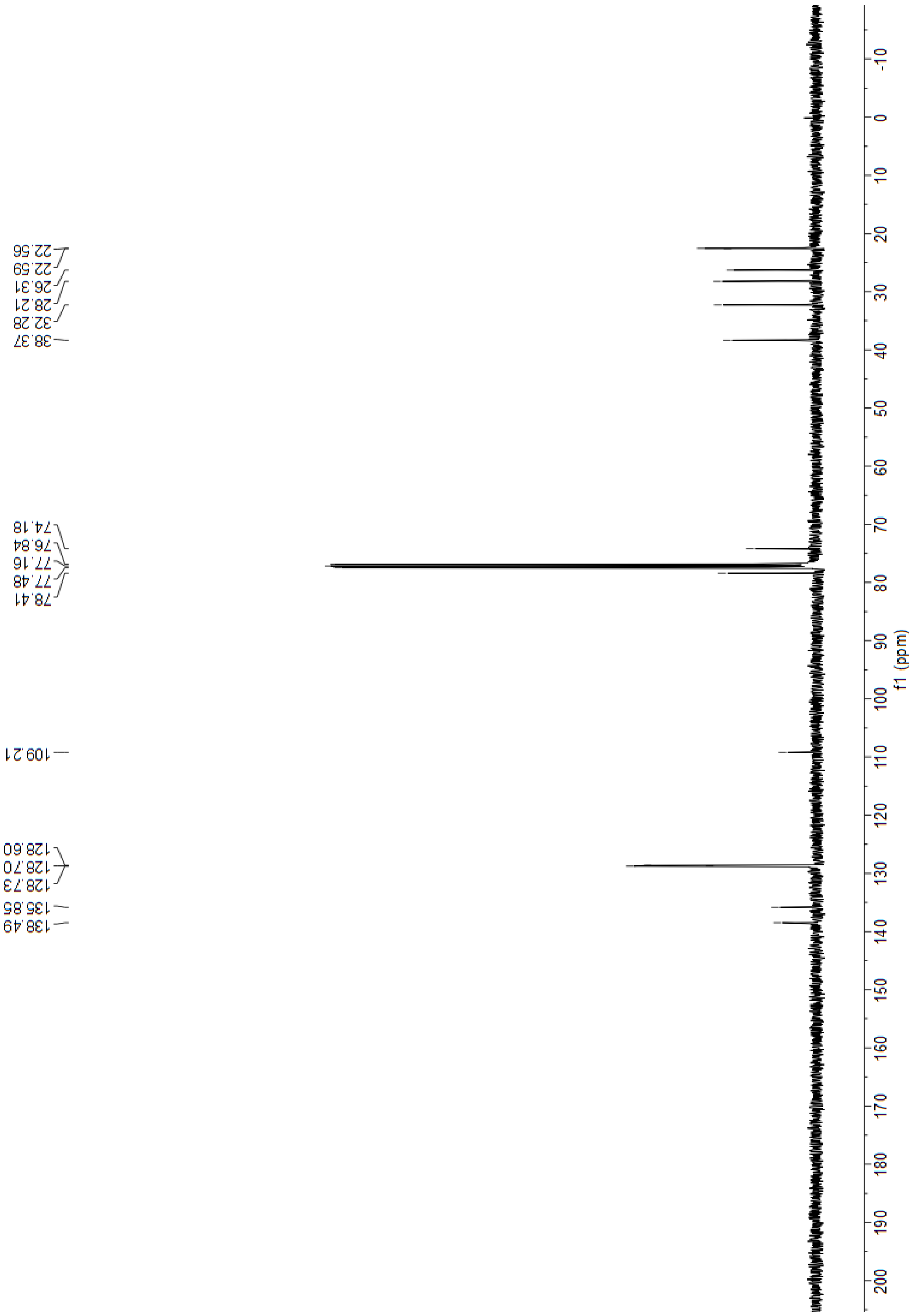

(E)-N-(benzyloxy)-2-hydroxy-2-methylhexanimidoyl cyanide (3ab)

^{1}H NMR of **3ab** ($CDCl_3$, 400 MHz, 25 ºC)

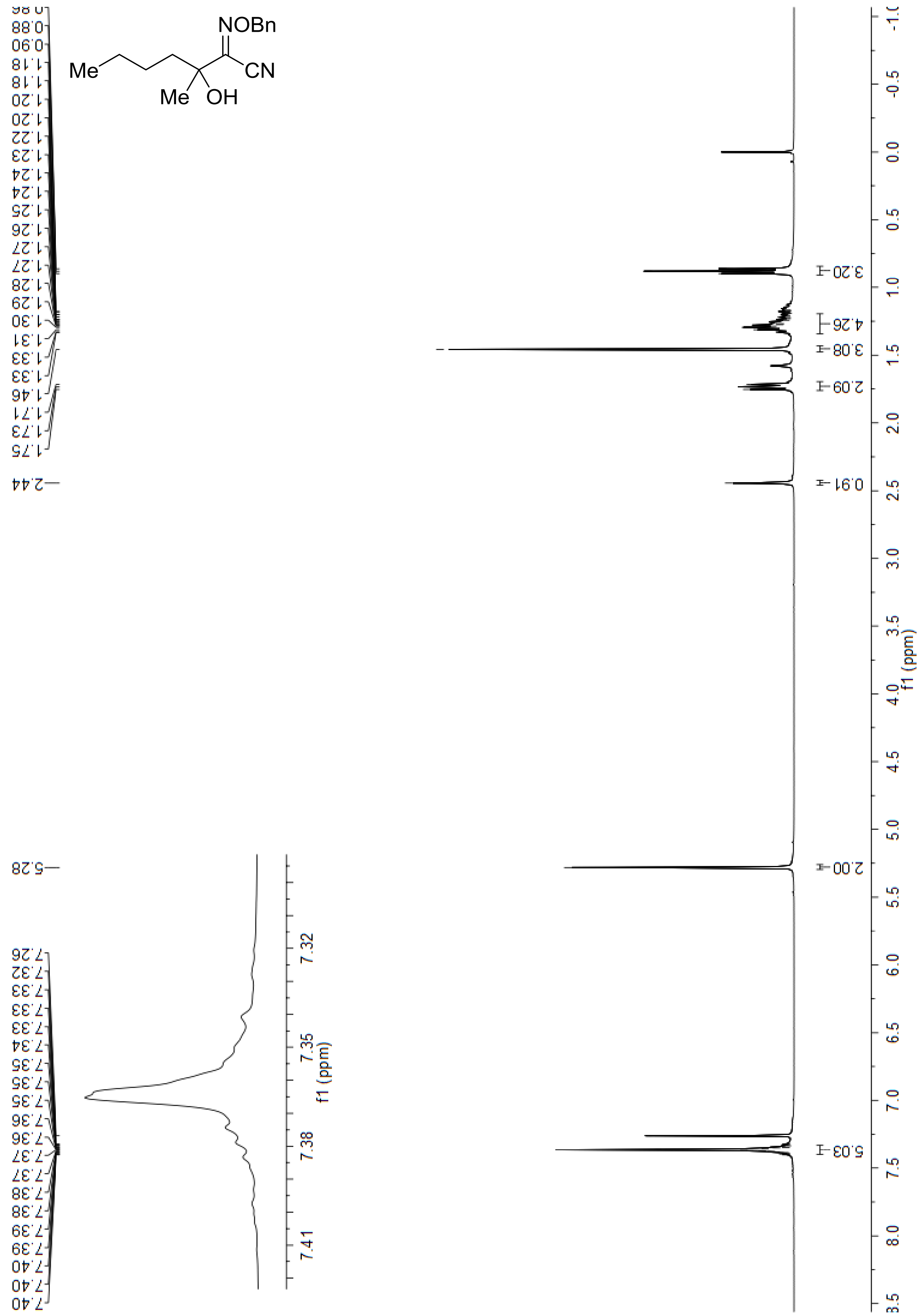

^{13}C NMR of **3ab** ($CDCl_3$, 151 MHz, 25 ºC)

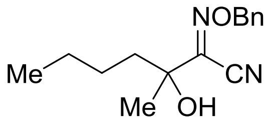

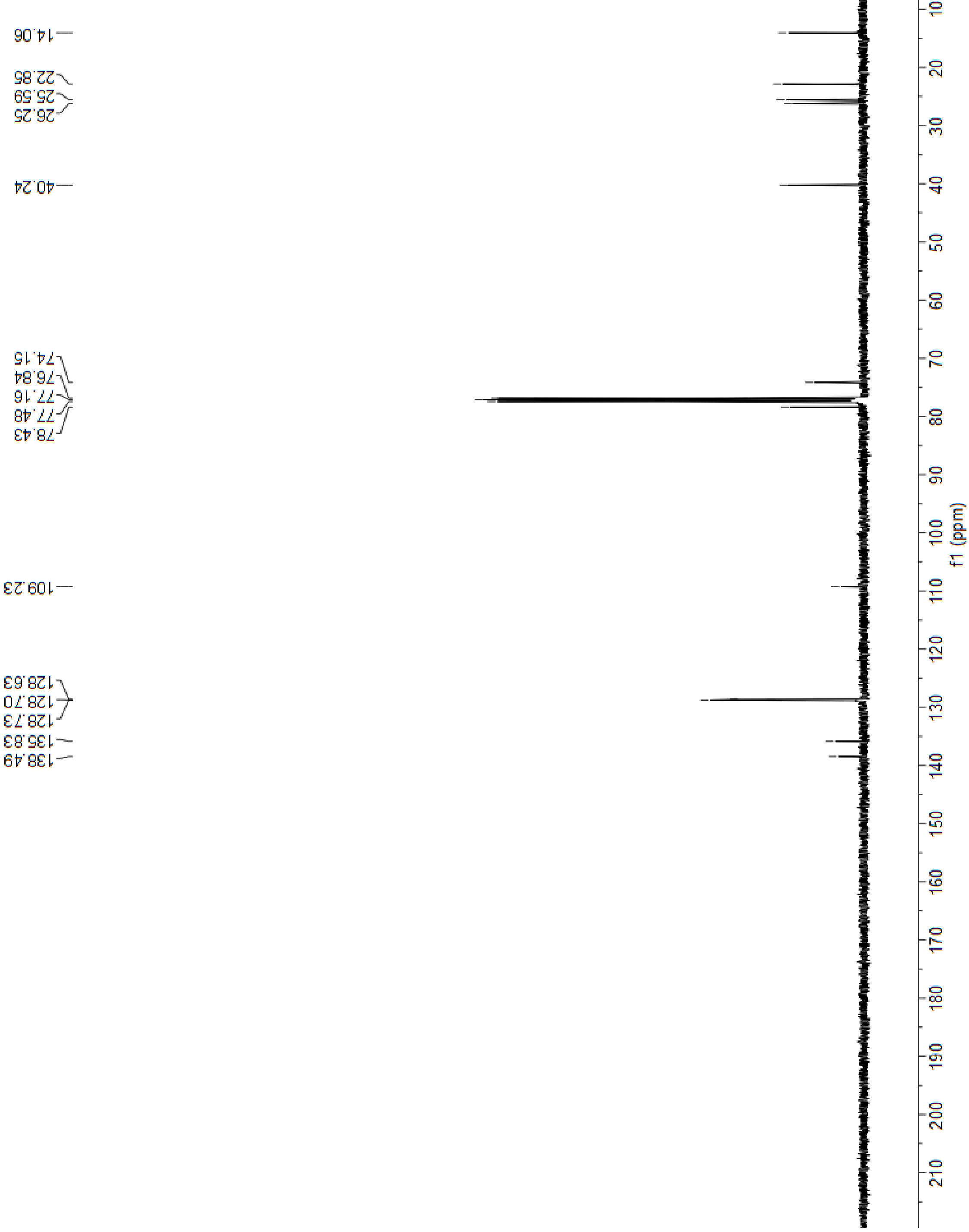

***N*-(benzyloxy)-2-methoxyoctanimidoyl cyanide (3a-2)**

^{1}H NMR of **3a-2** ($CDCl_3$, 400 MHz, 25 ºC)

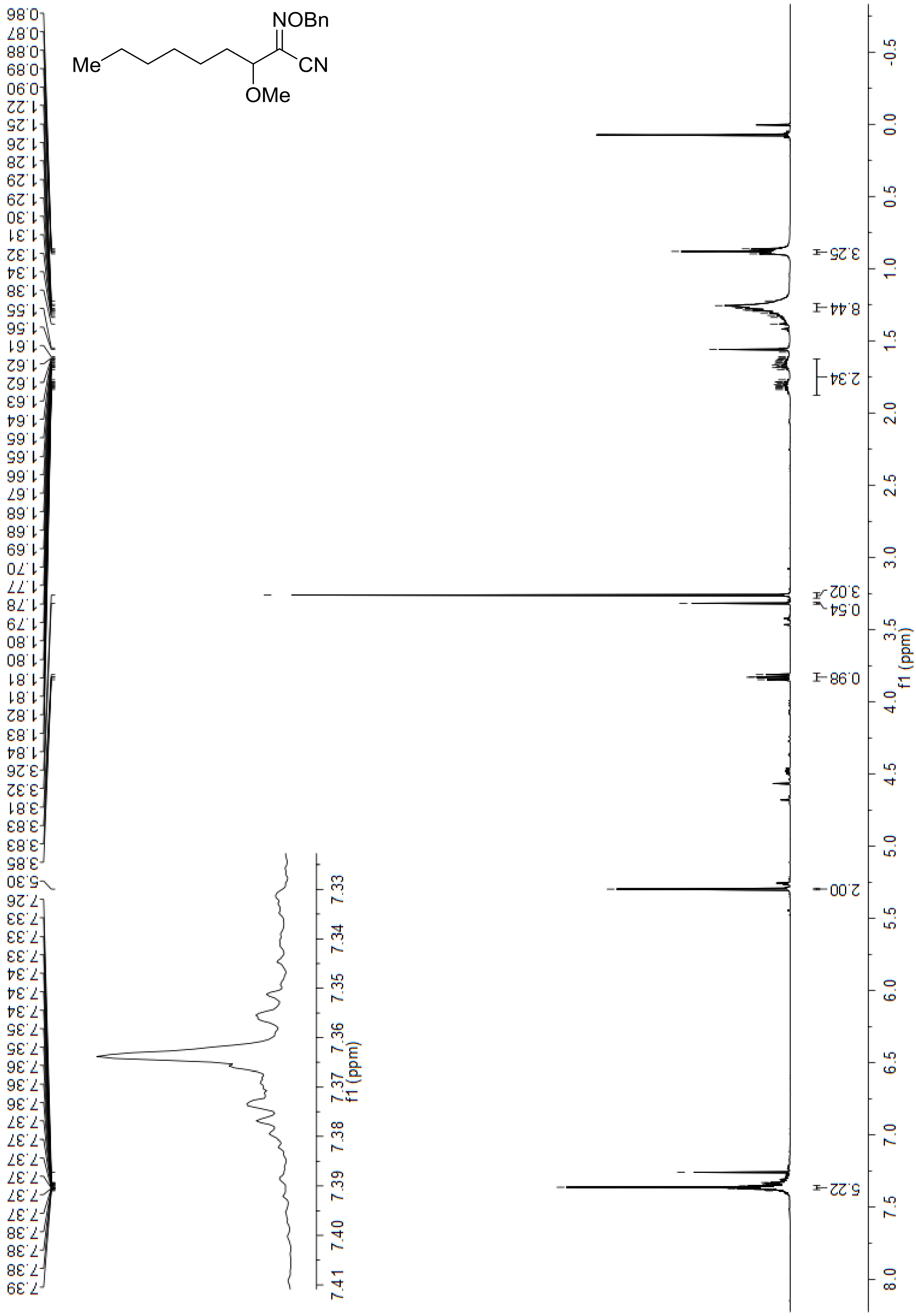

^{13}C NMR of **3a-2** ($CDCl_3$, 101 MHz, 25 ºC)

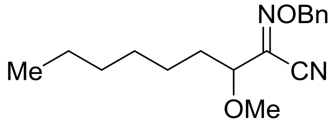

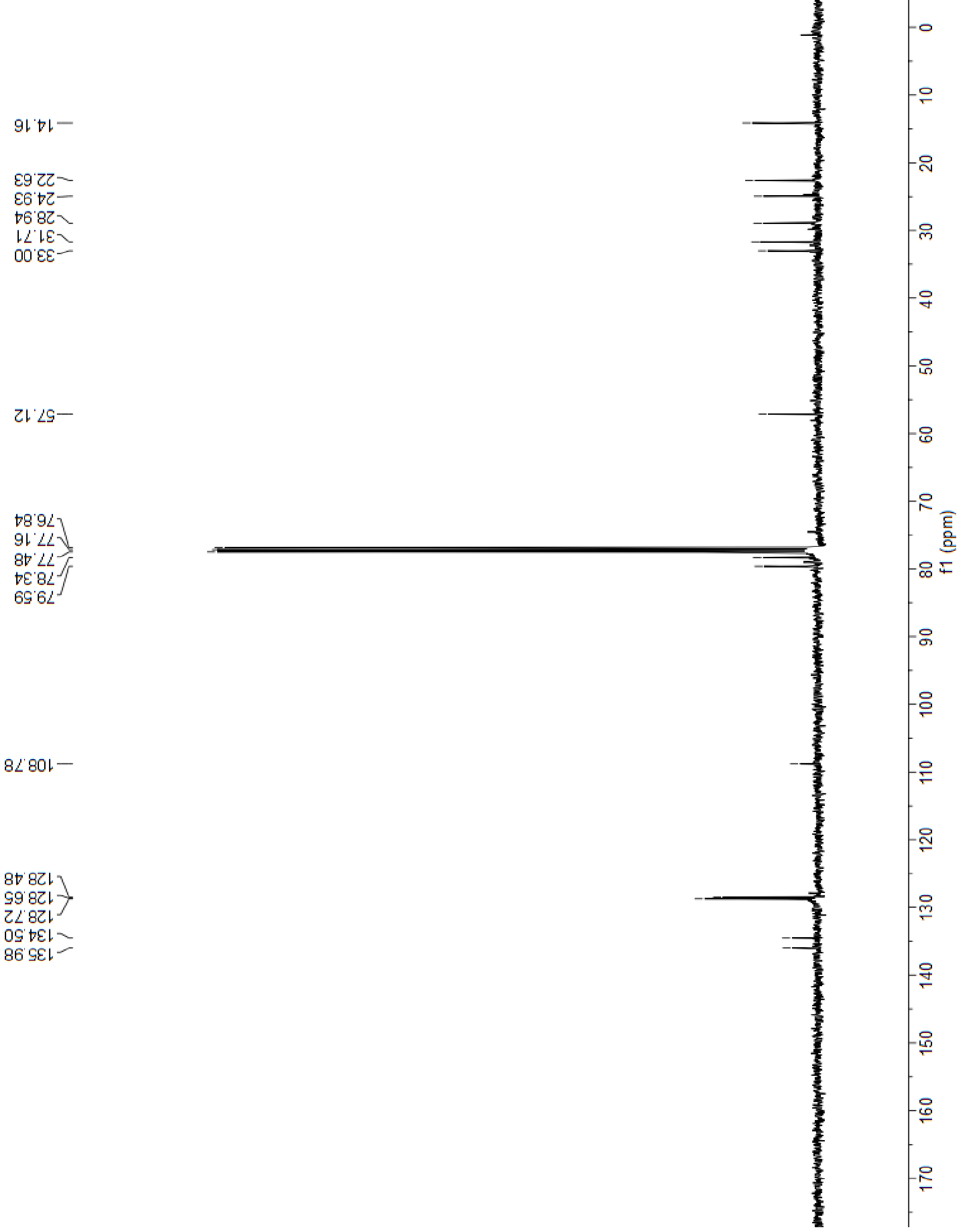

***N*-(Benzyloxy)-2,6-dihydroxyhexanimidoyl cyanide (3a-3)**

^{1}H NMR of **3a-3** ($CDCl_3$, 400 MHz, 25 ºC)

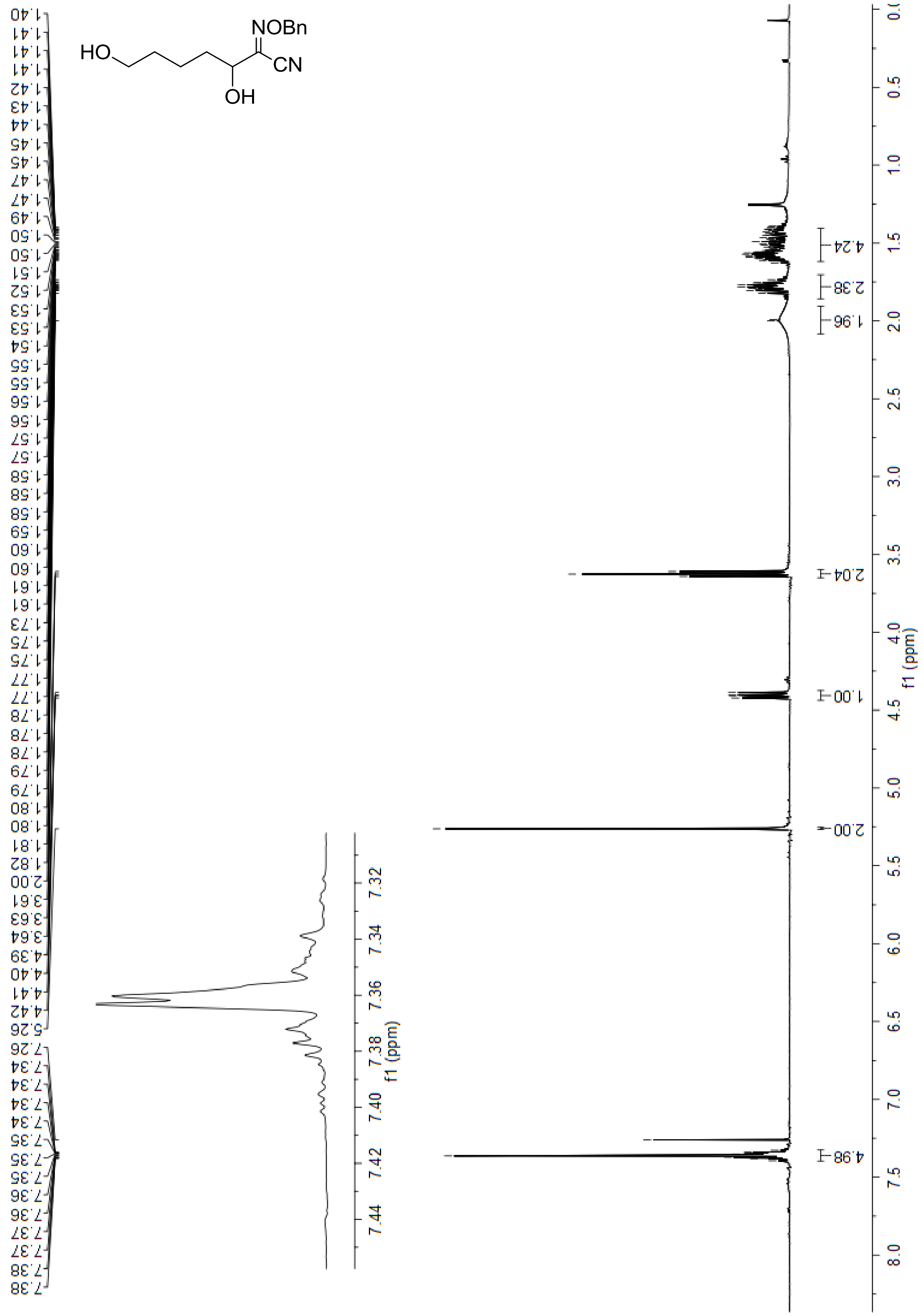

^{13}C NMR of **3a-3** ($CDCl_3$, 101 MHz, 25 ºC)

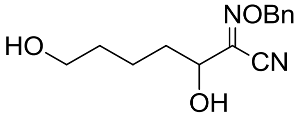

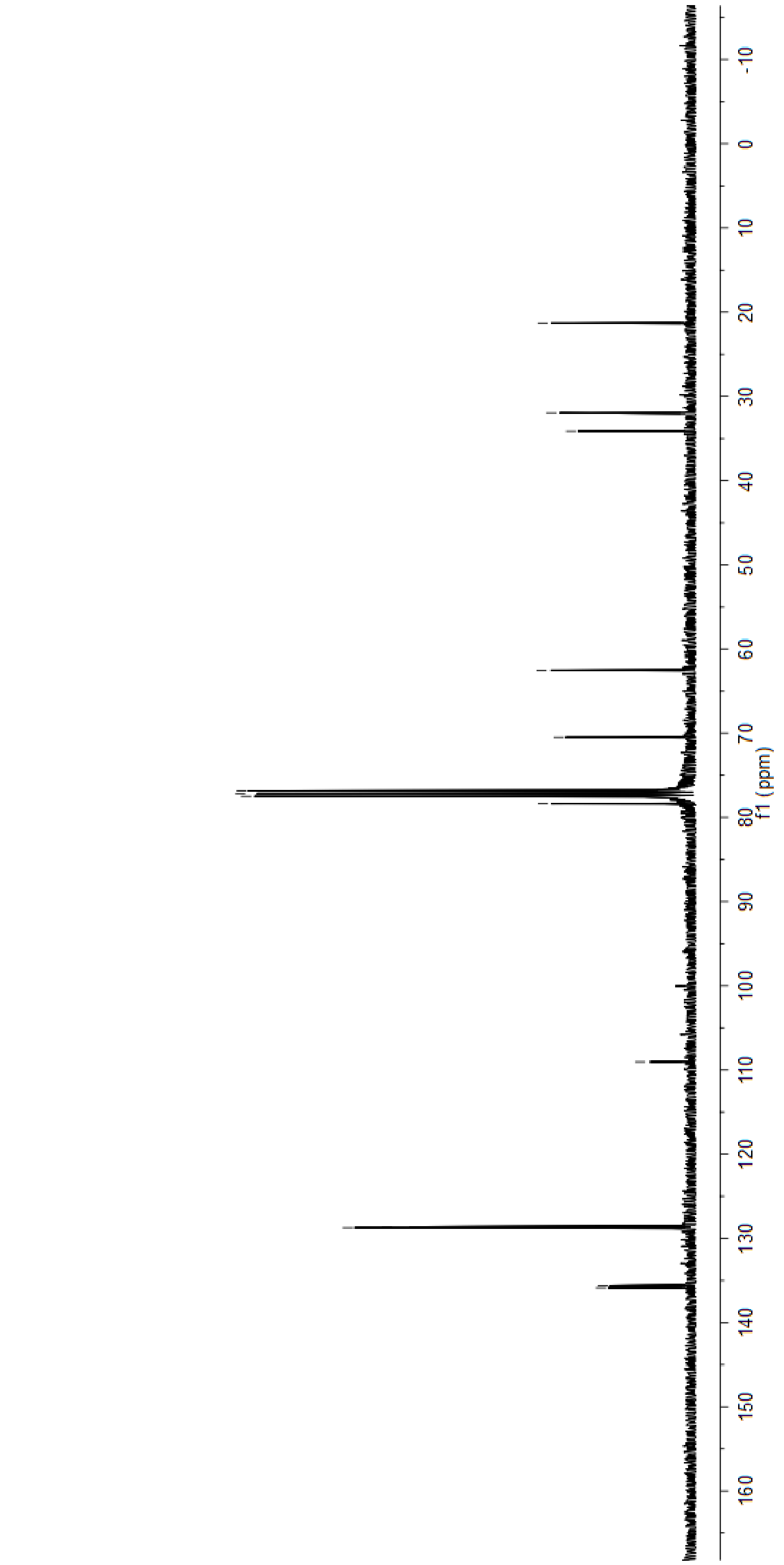

N-(Benzyloxy)-2-((triphenylsilyl)oxy)octanimidoyl cyanide(3a-4)

^{1}H NMR of **3a-4** ($CDCl_3$, 400 MHz, 25 ºC)

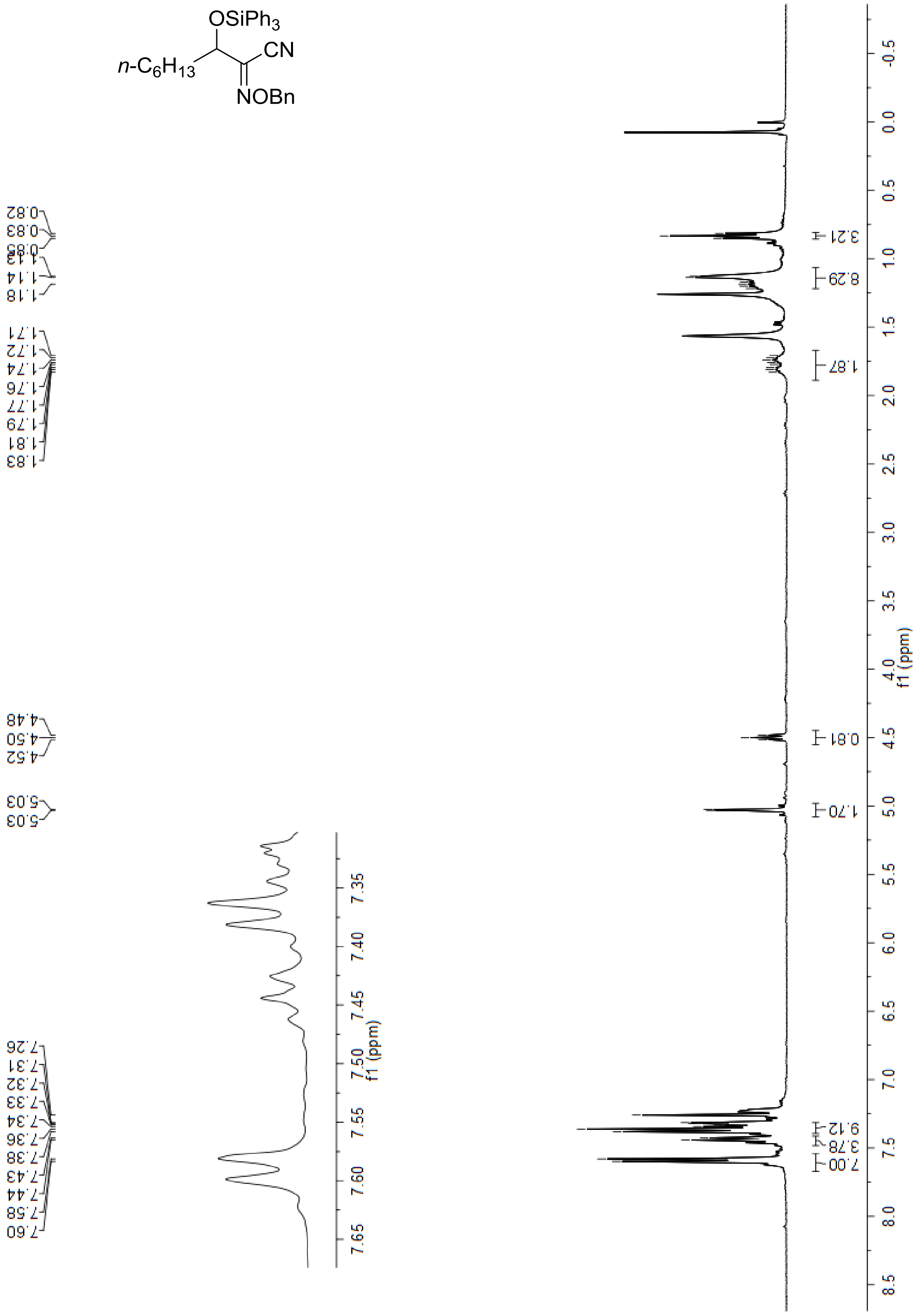

^{13}C NMR of **3a-4** ($CDCl_3$, 151 MHz, 25 ºC)

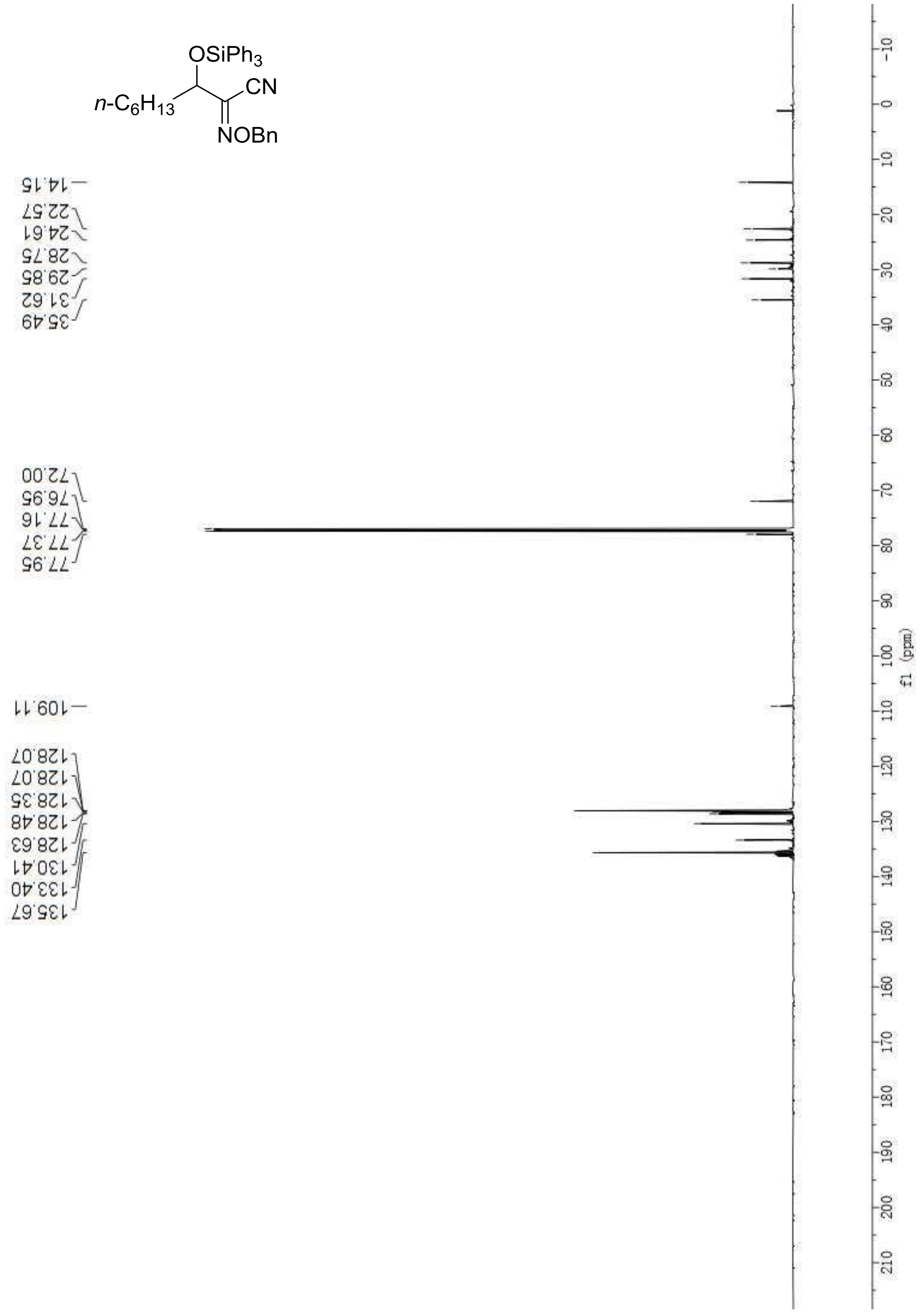

***N*-(Benzyloxy)-2-hydroxyoctanimidoyl cyanide (3a-5)**

^{1}H NMR of **3a-5** ($CDCl_3$, 400 MHz, 25 ºC)

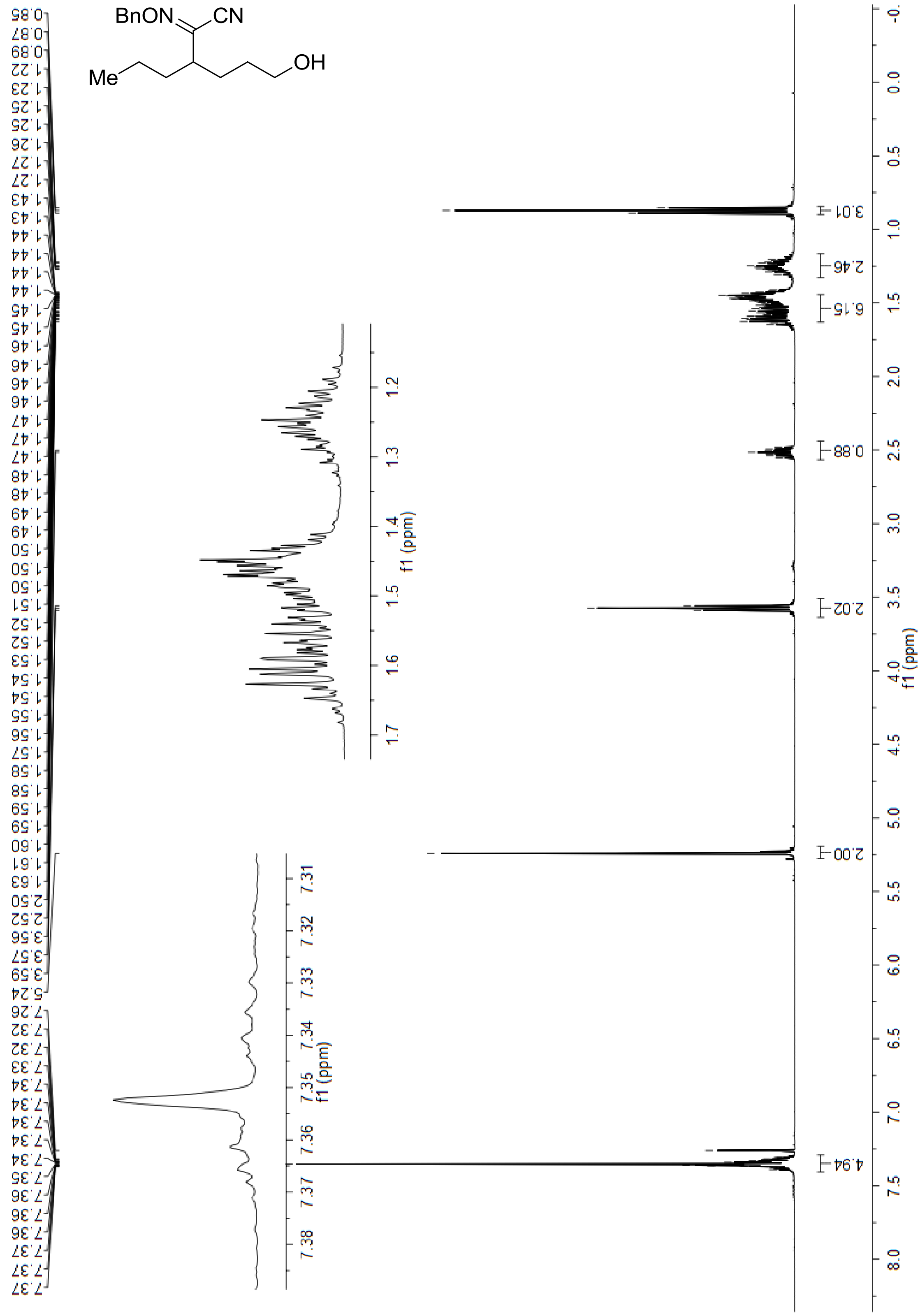

^{13}C NMR of **3a-5** ($CDCl_3$, 101 MHz, 25 ºC)

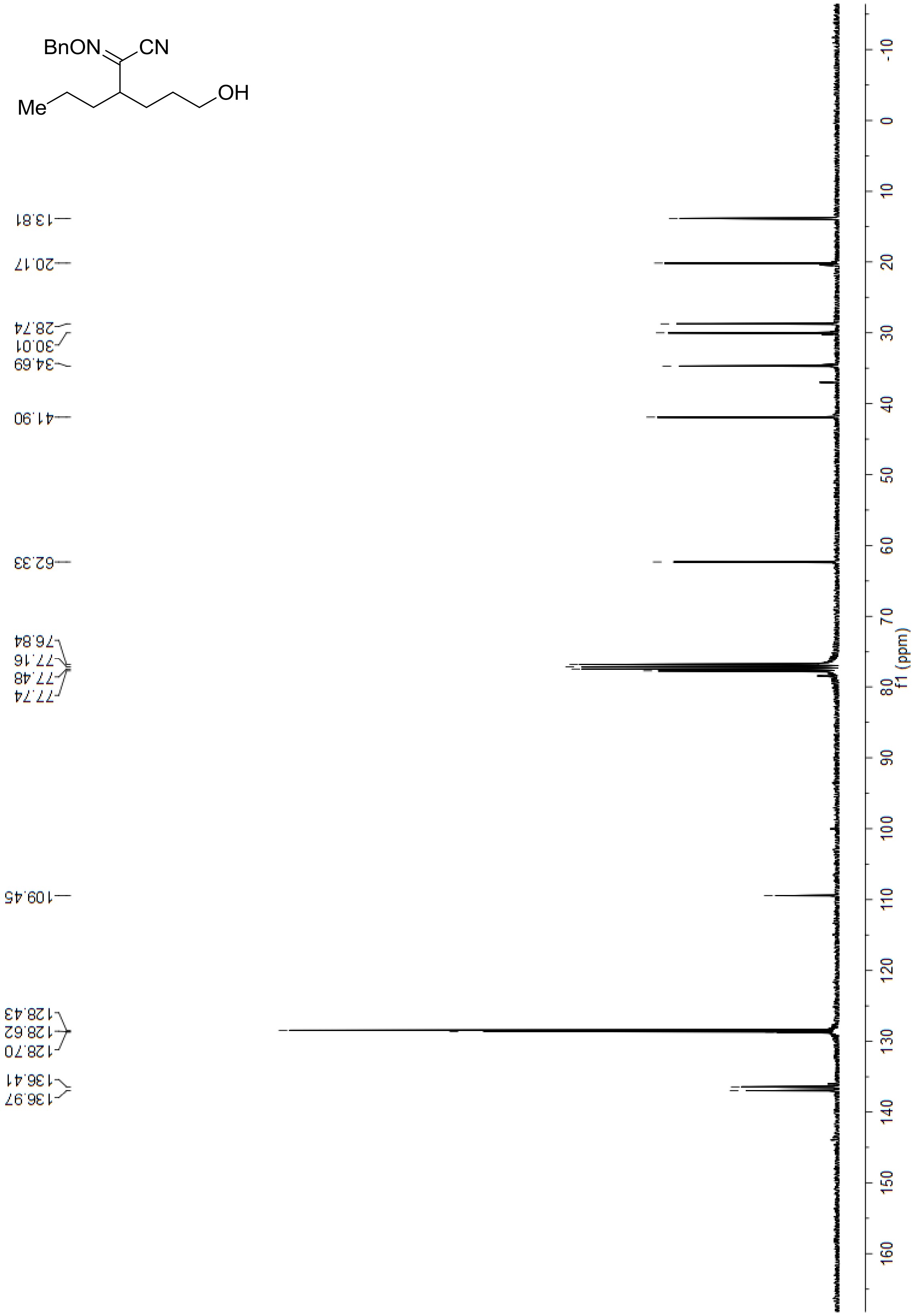

(Z)-2-((benzyloxy)imino)-3-hydroxynonanamide(3a-8)

^{1}H NMR of **3a-8** ($CDCl_3$, 400 MHz, 25 ºC)

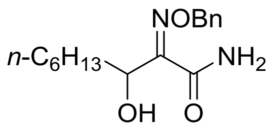

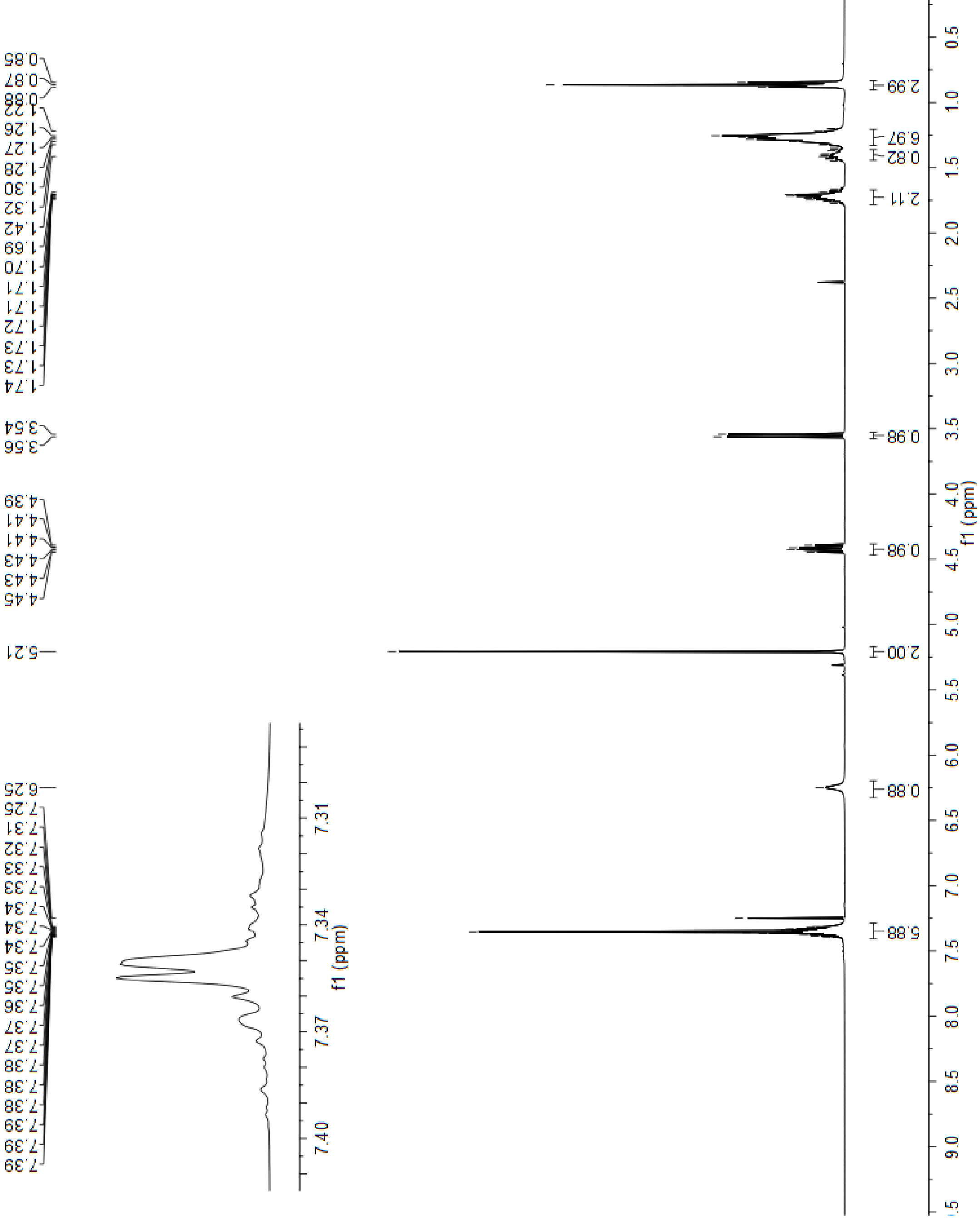

^{13}C NMR of **3a-8** ($CDCl_3$, 151 MHz, 25 ºC)

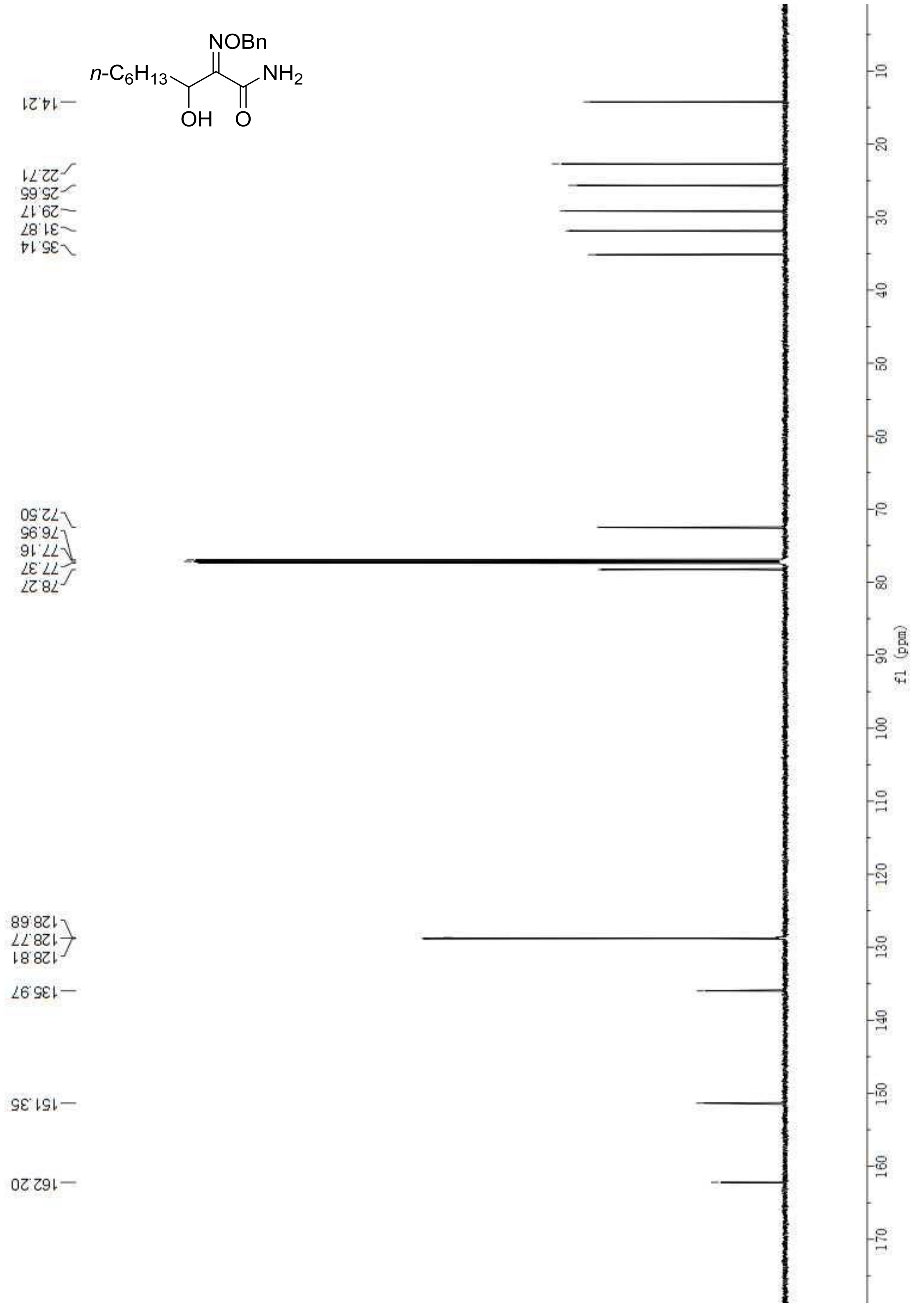

1-(4-Methylquinolin-2-yl)heptan-1-ol (5a)

^{1}H NMR of **5a** ($CDCl_3$, 400 MHz, 25 ºC)

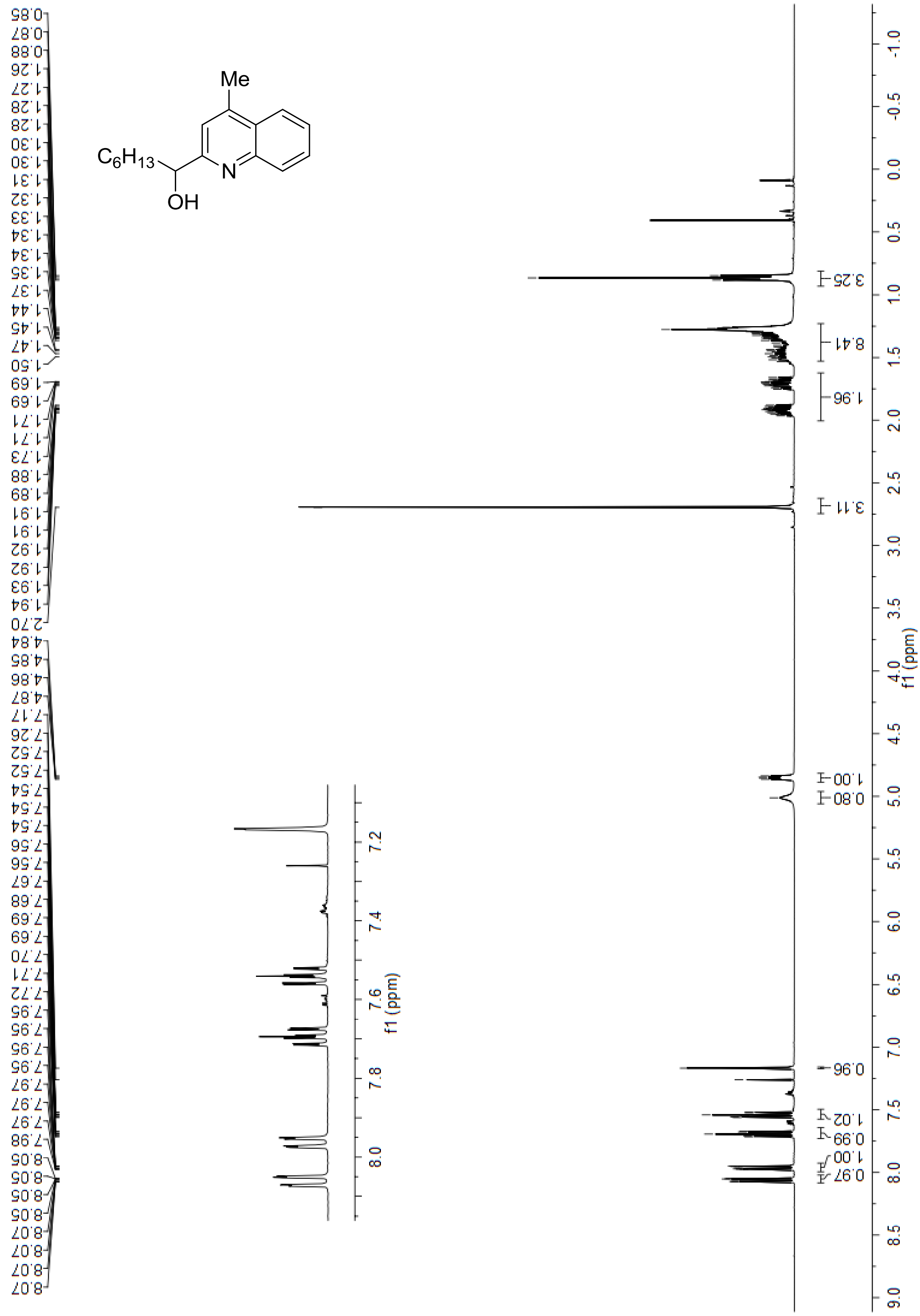

^{13}C NMR of **5a** ($CDCl_3$, 101 MHz, 25 ºC)

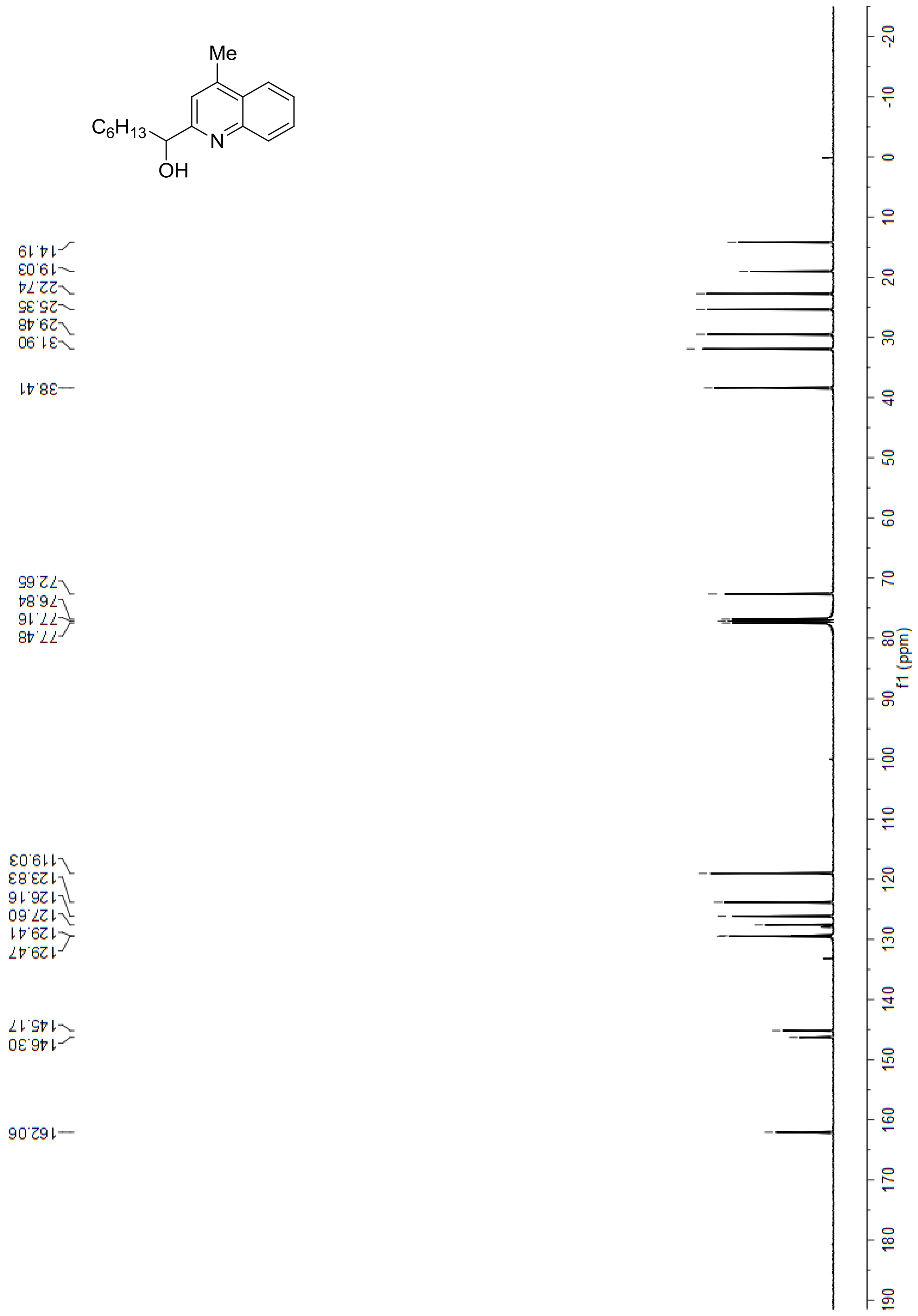

1-(2-Methylquinolin-4-yl)heptan-1-ol (5b)

^{1}H NMR of **5b** ($CDCl_3$, 400 MHz, 25 ºC)

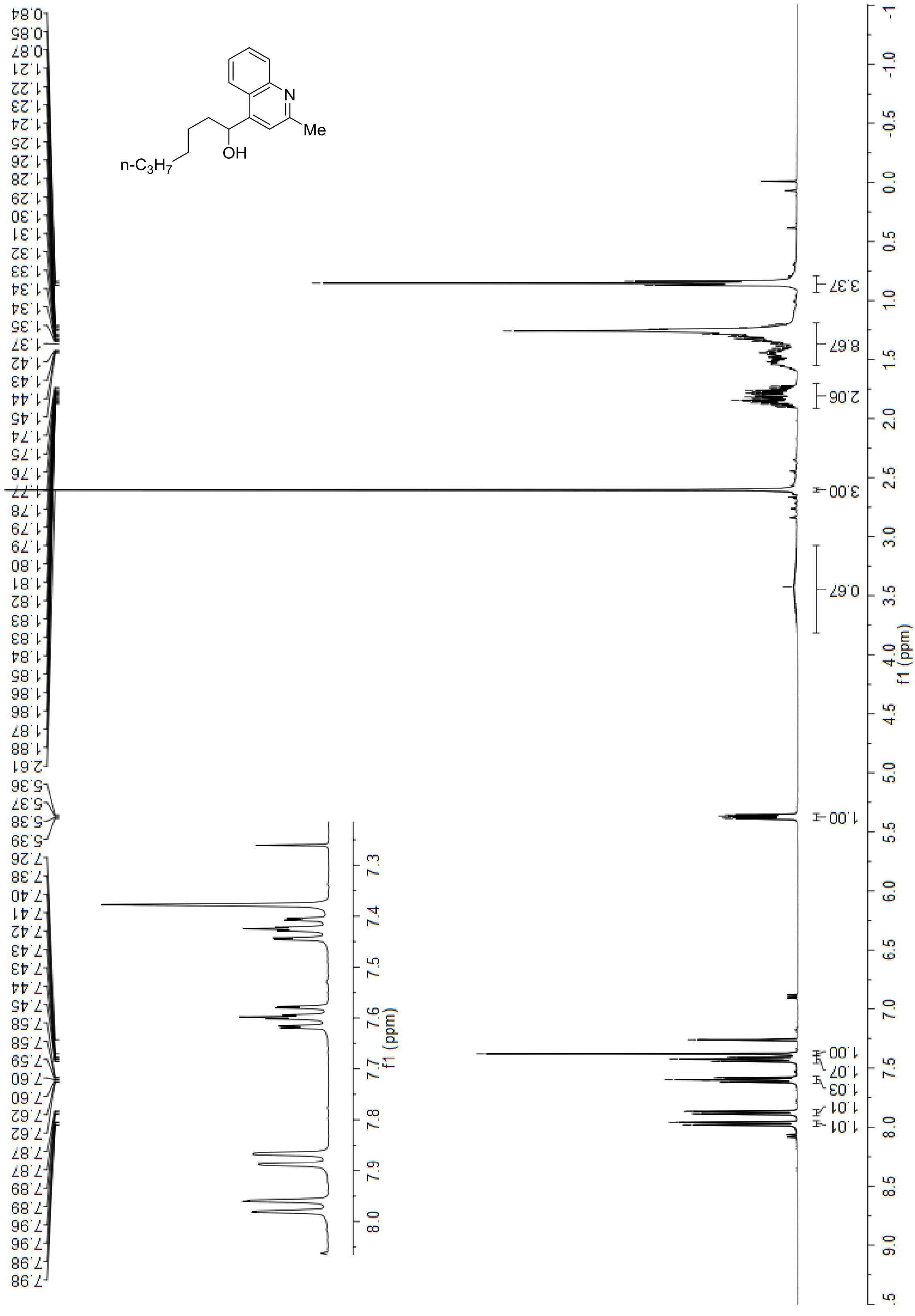

^{13}C NMR of **5b** ($CDCl_3$, 101 MHz, 25 ºC)

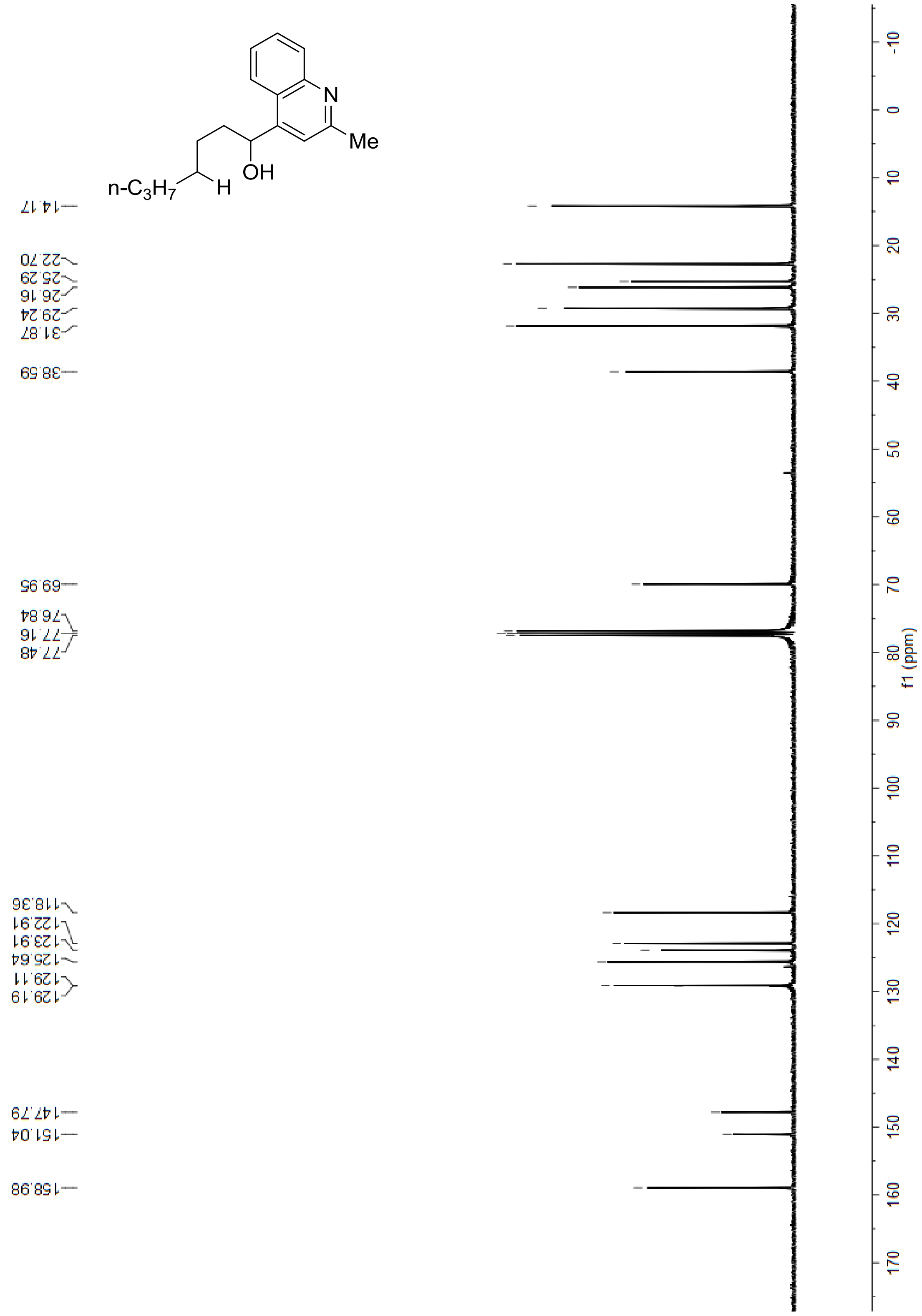

1-(2-phenylquinolin-4-yl)heptan-1-ol (5c)

^{1}H NMR of **5c** ($CDCl_3$, 400 MHz, 25 ºC)

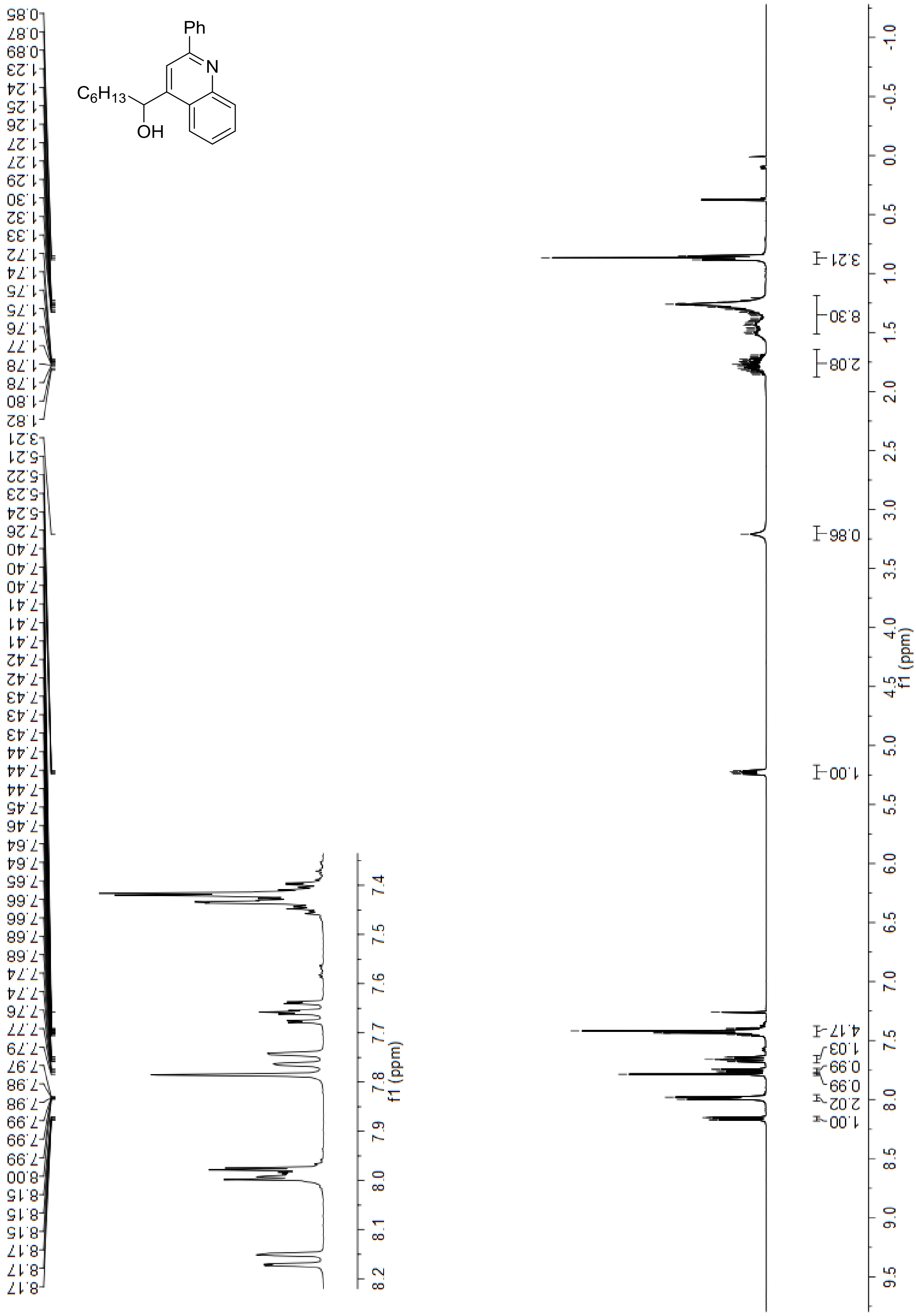

^{13}C NMR of **5c** ($CDCl_3$, 101 MHz, 25 ºC)

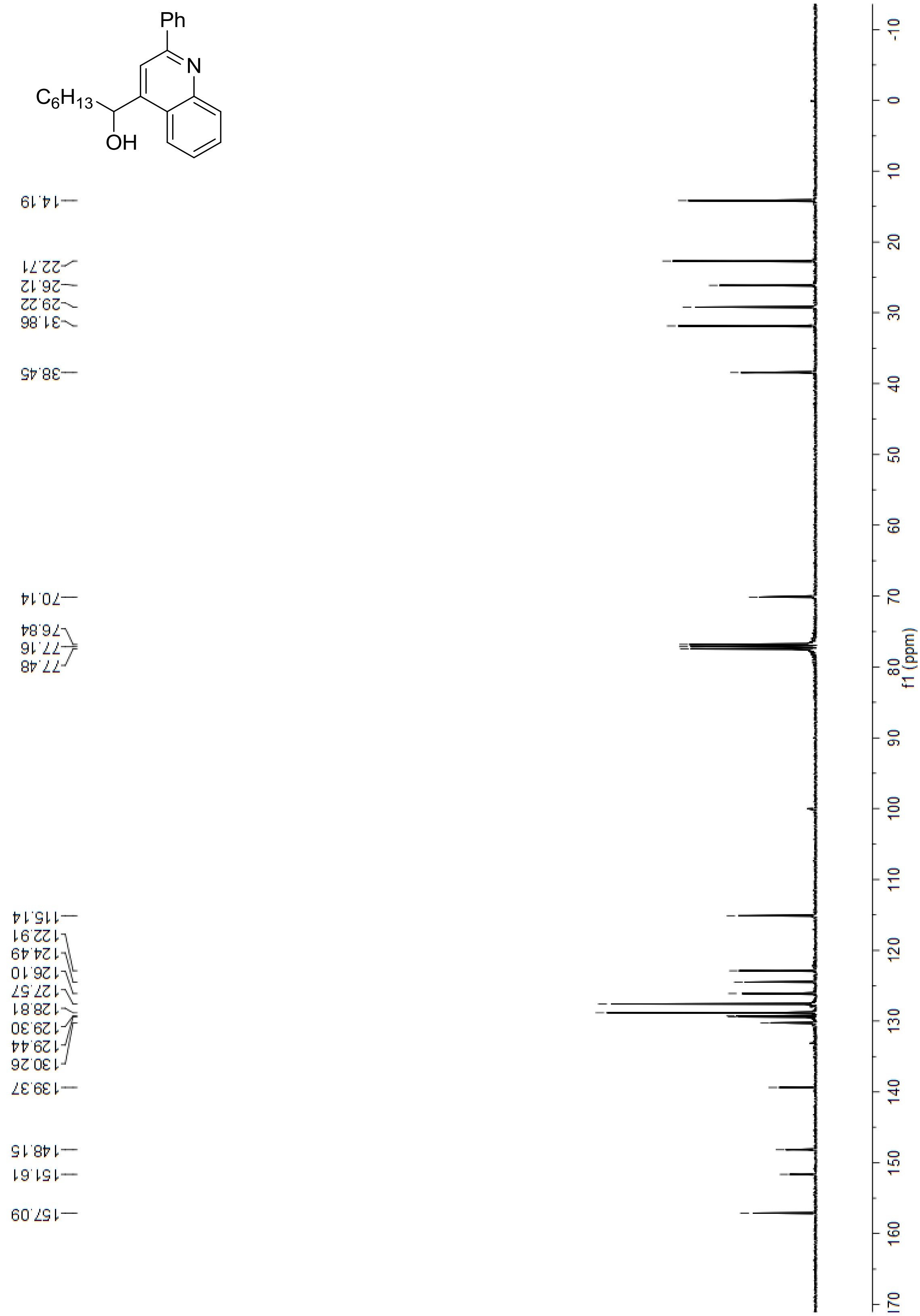

1-(2-(4-fluorophenyl)quinolin-4-yl)heptan-1-ol (5d)

^{1}H NMR of **5d** ($CDCl_3$, 400 MHz, 25 ºC)

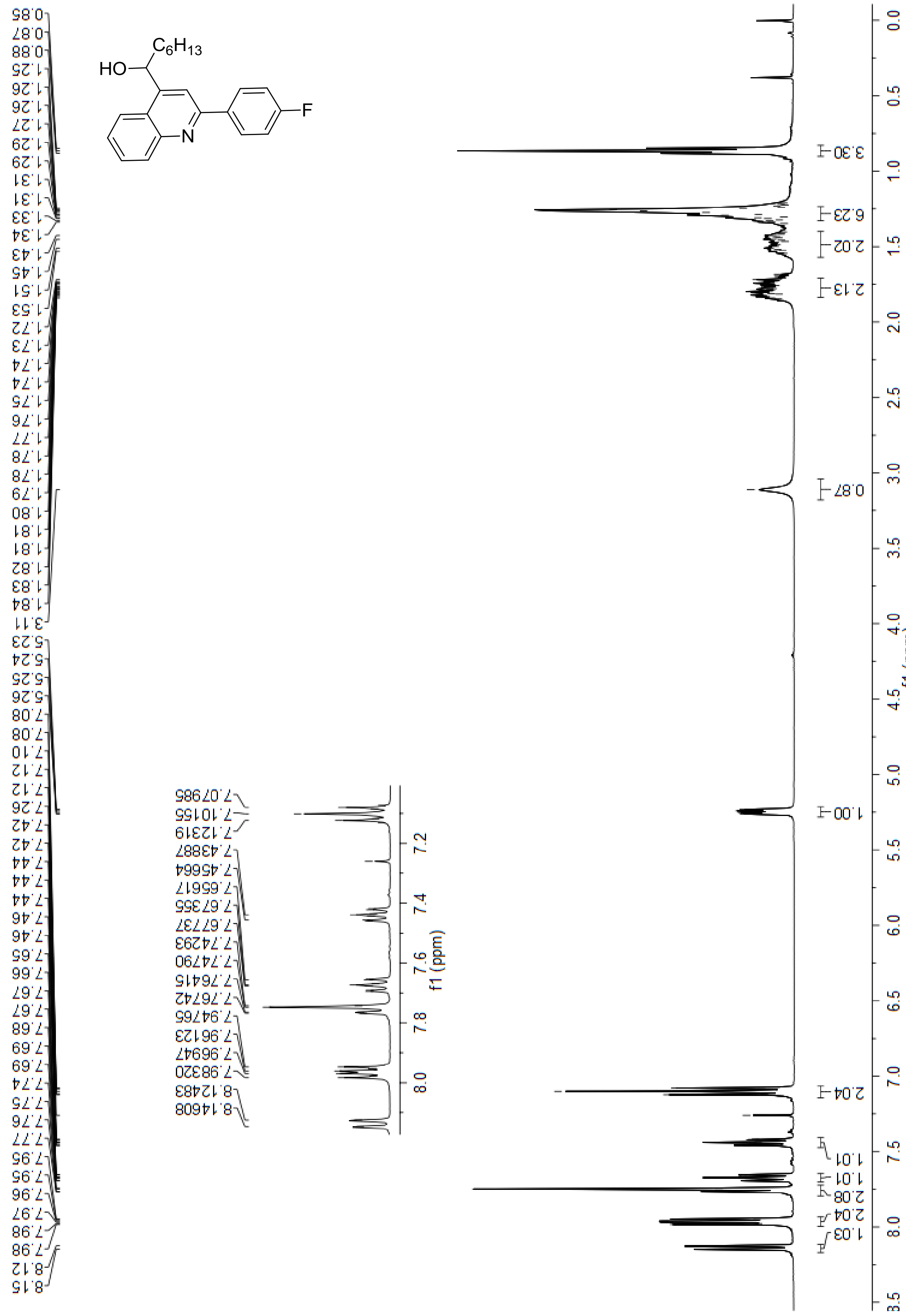

^{13}C NMR of **5d** ($CDCl_3$, 101 MHz, 25 ºC)

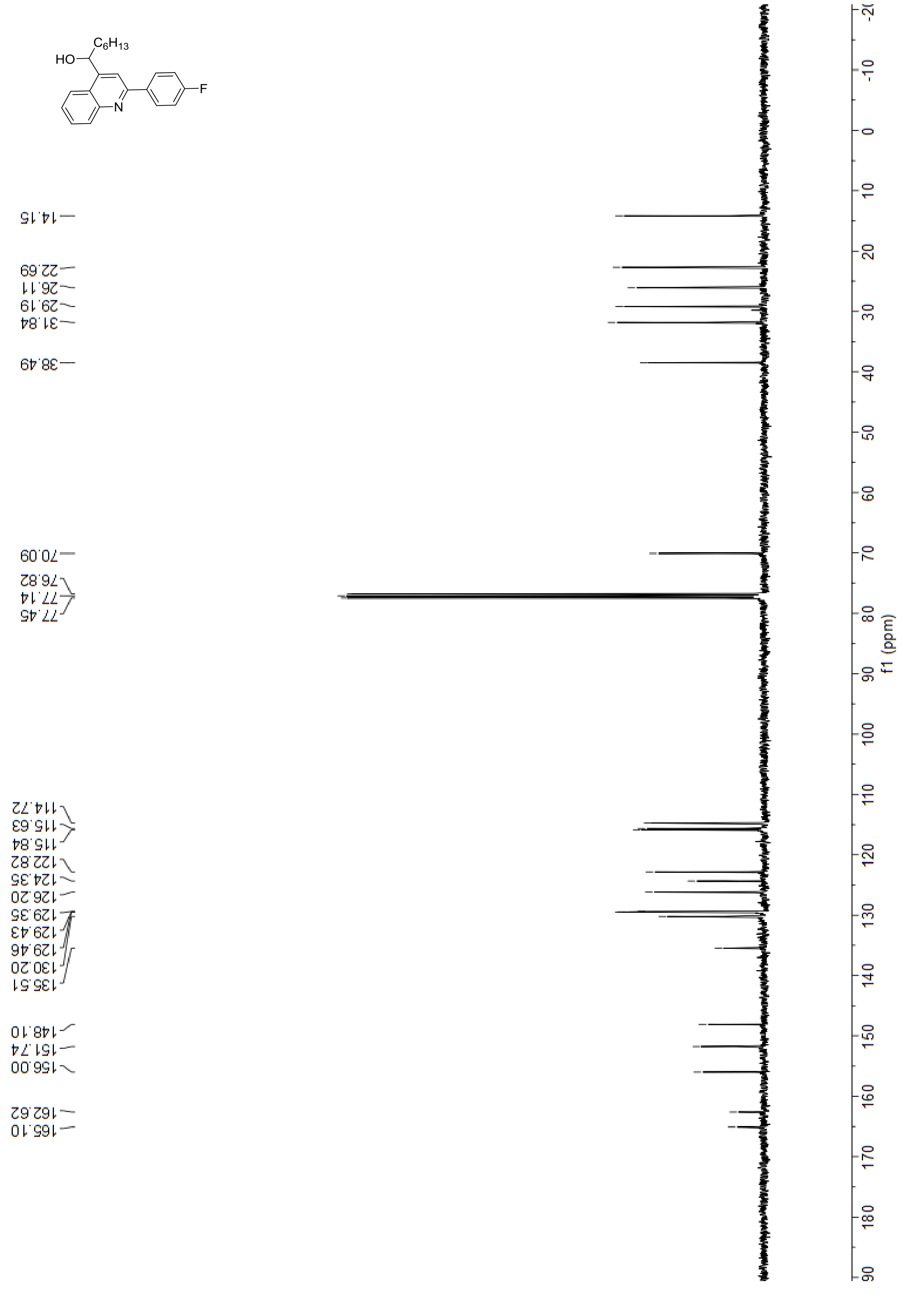

^{13}F NMR of **5d** ($CDCl_3$, 375 MHz, 25 ºC)

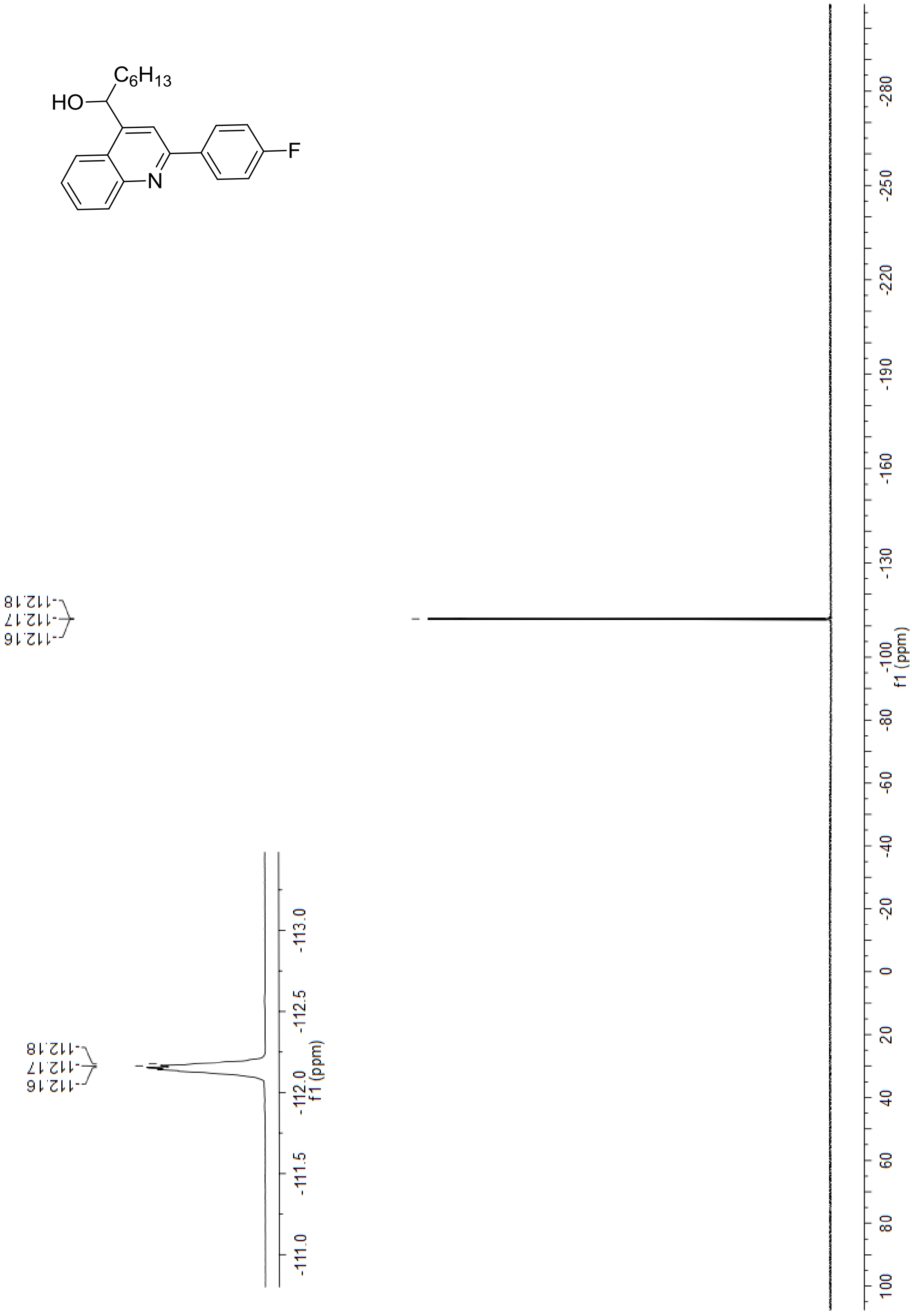

1-(2-(4-chlorophenyl)quinolin-4-yl)heptan-1-ol (5e)

^{1}H NMR of **5e** ($CDCl_3$, 400 MHz, 25 ºC)

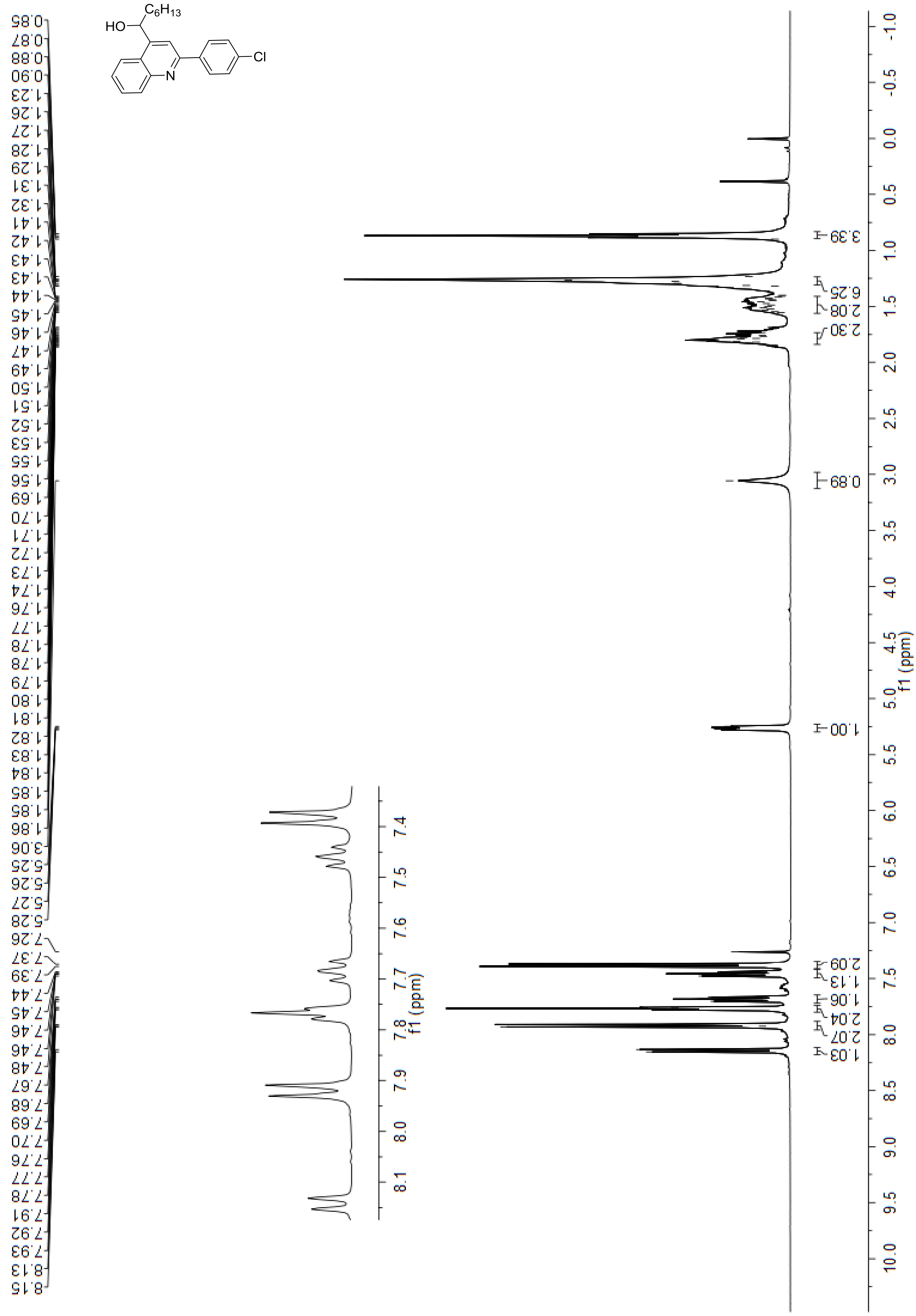

^{13}C NMR of **5e** ($CDCl_3$, 101 MHz, 25 ºC)

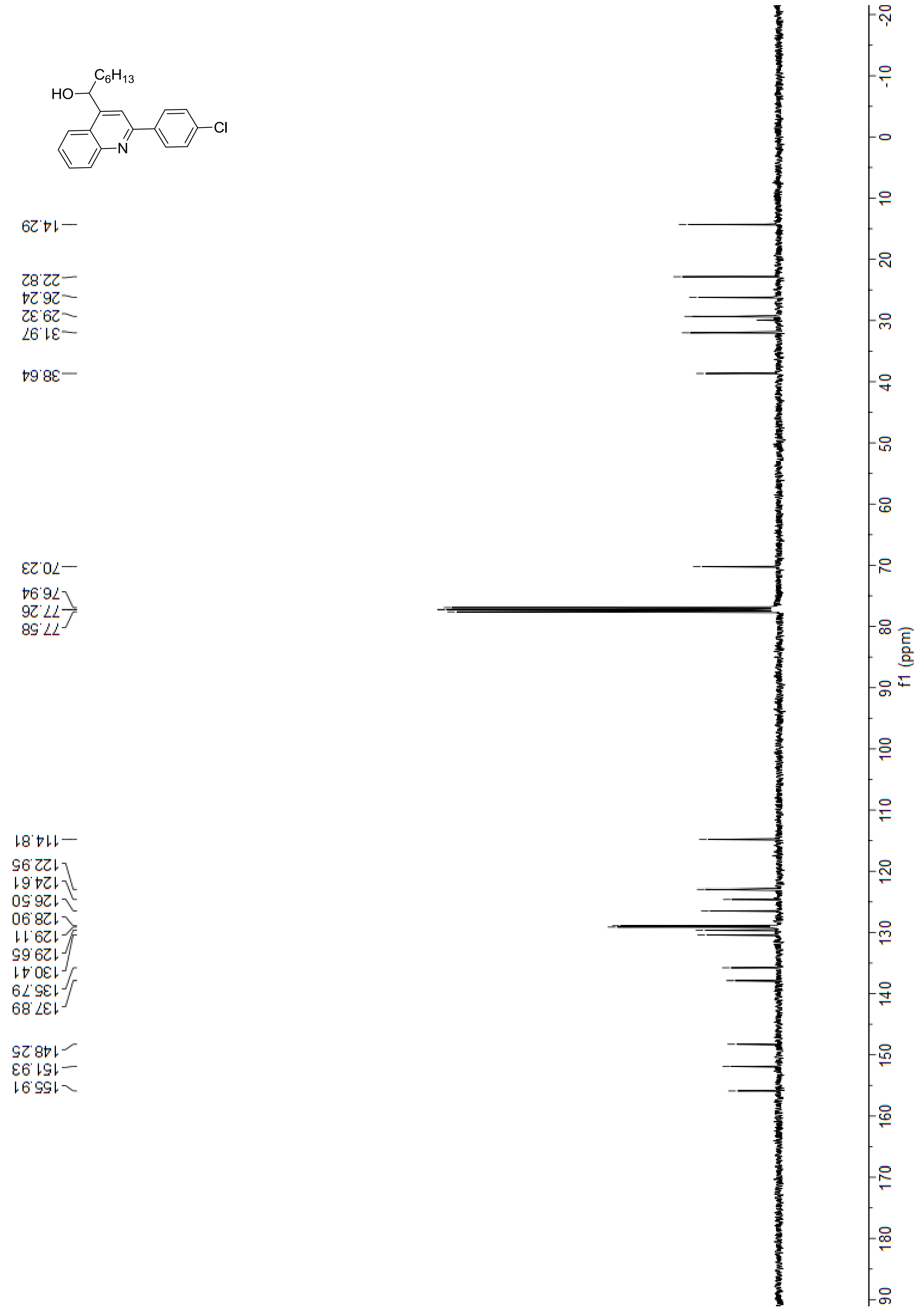

1-(2-(4-bromophenyl)quinolin-4-yl)heptan-1-ol (5f)

^{1}H NMR of **5f** ($CDCl_3$, 400 MHz, 25 ºC)

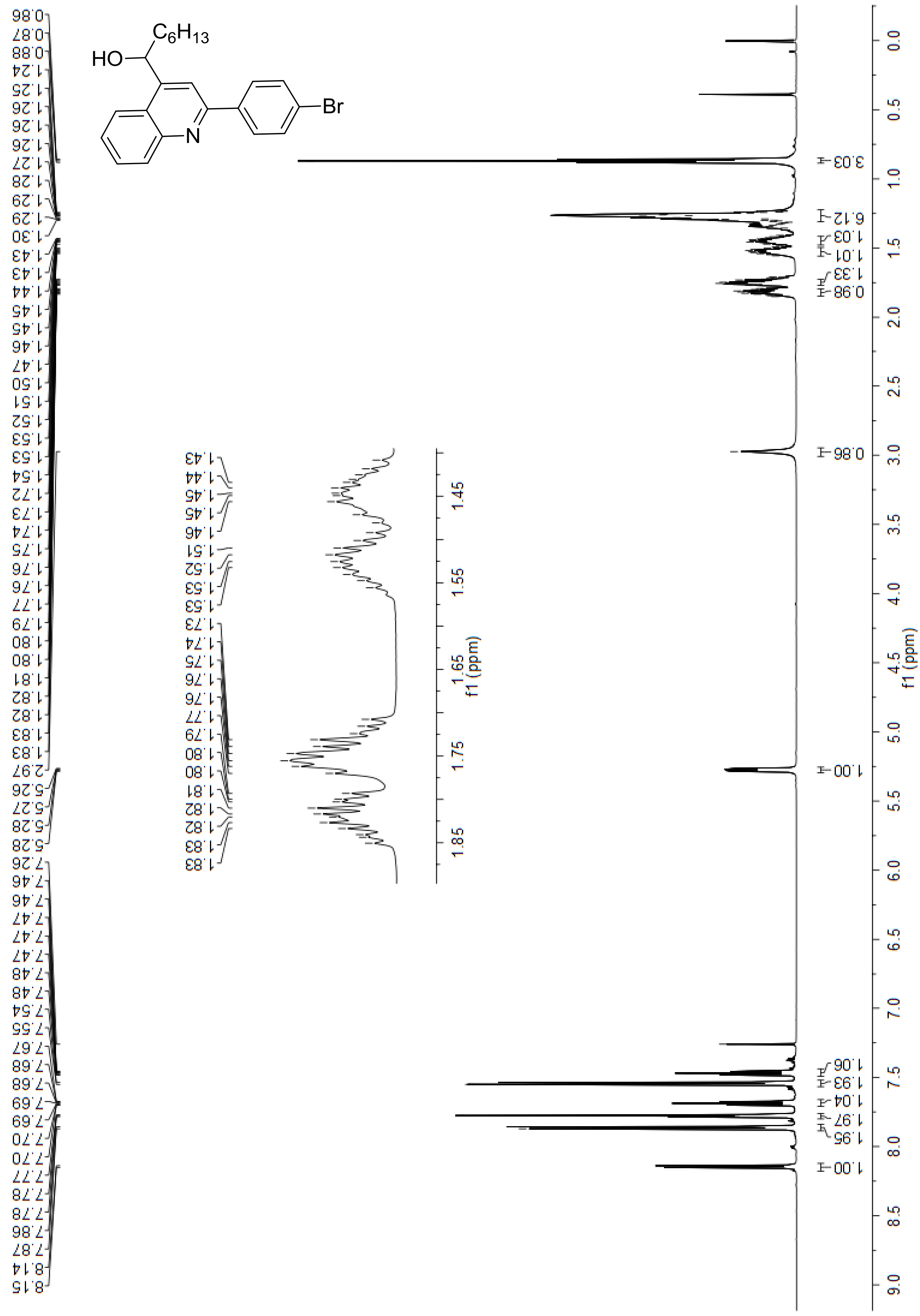

^{13}C NMR of **5f** ($CDCl_3$, 101 MHz, 25 ºC)

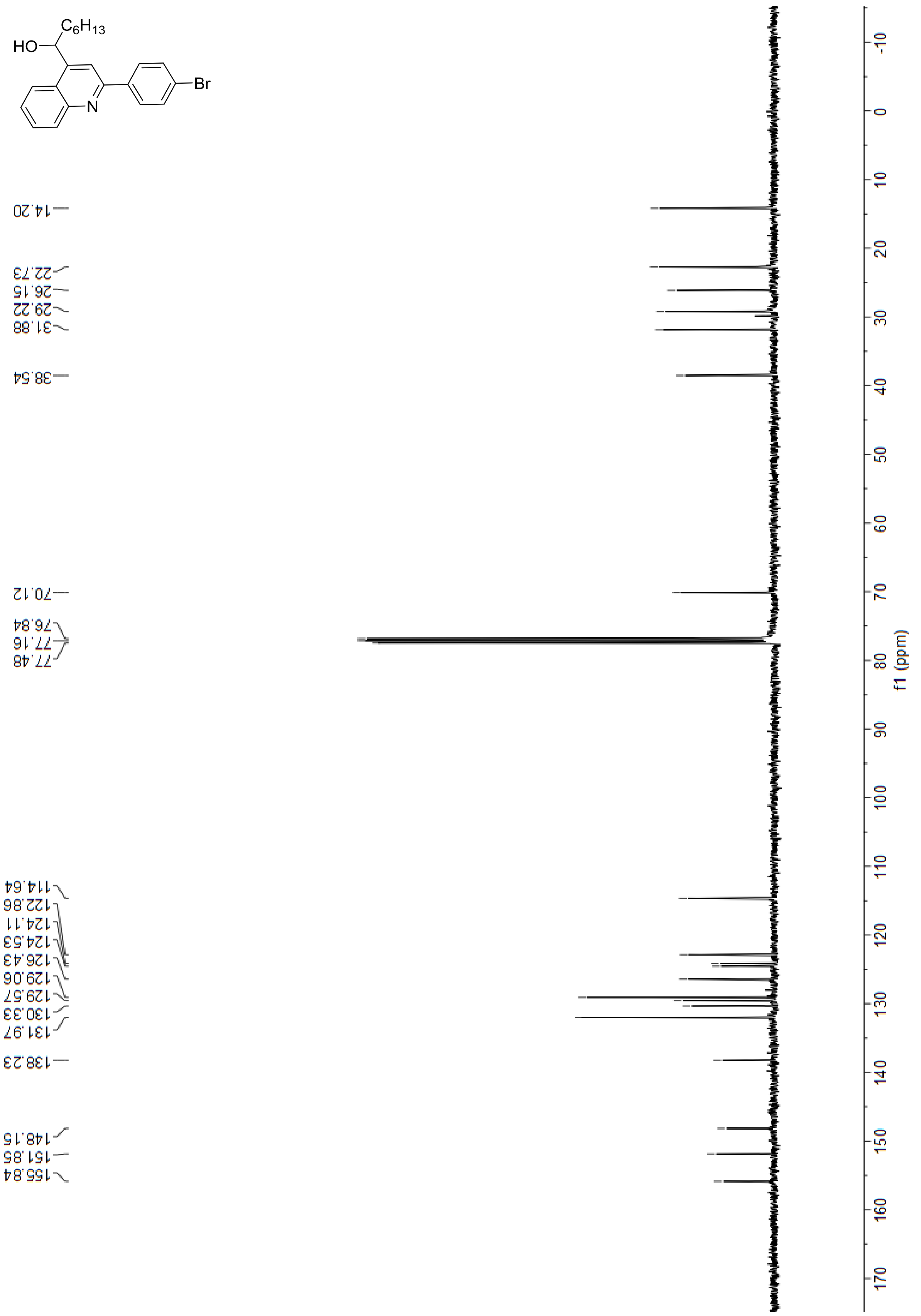

4-(4-(1-hydroxyheptyl)quinolin-2-yl)benzonitrile (5g)

^{1}H NMR of **5g** ($CDCl_3$, 400 MHz, 25 ºC)

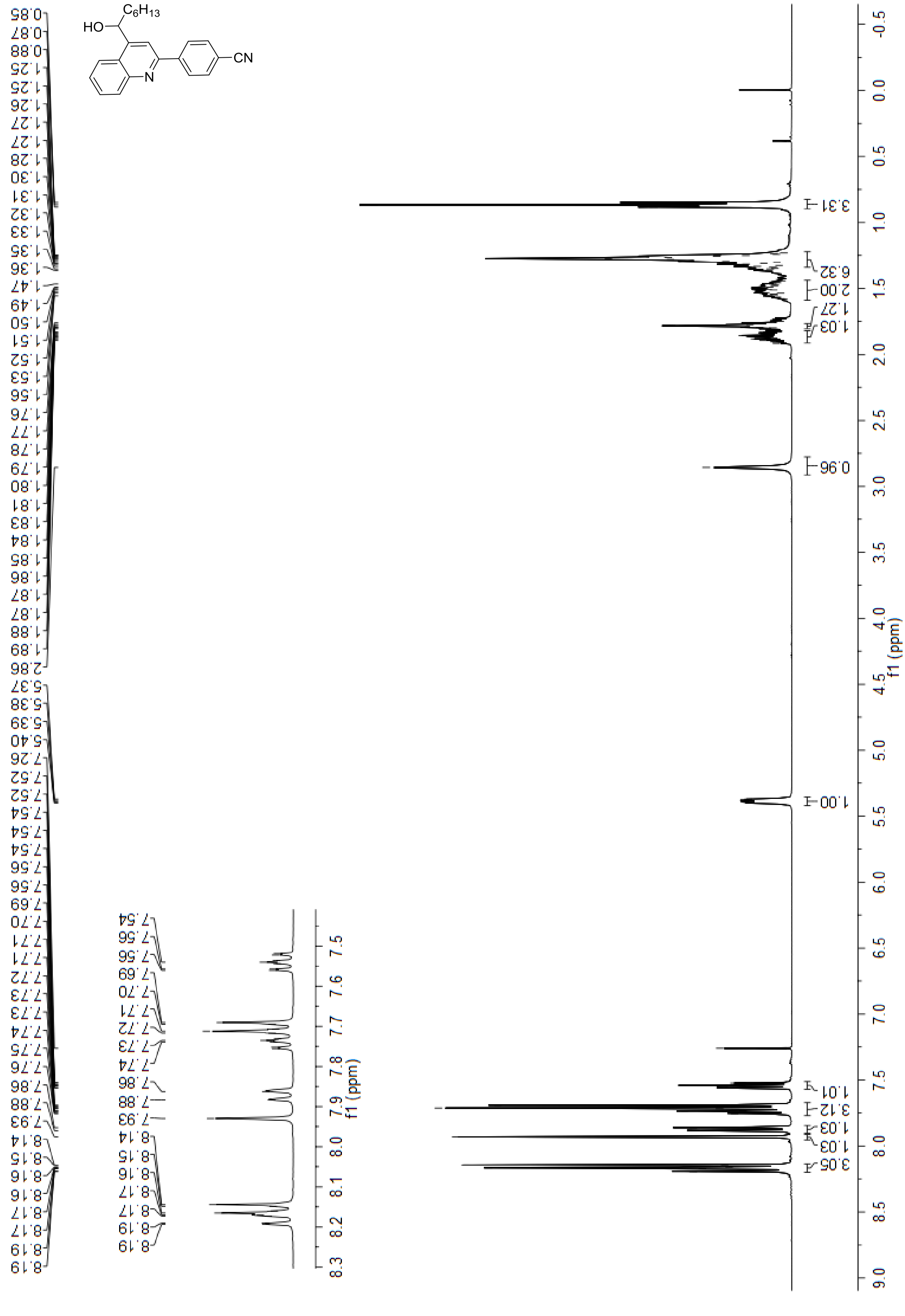

^{13}C NMR of **5g** ($CDCl_3$, 101 MHz, 25 ºC)

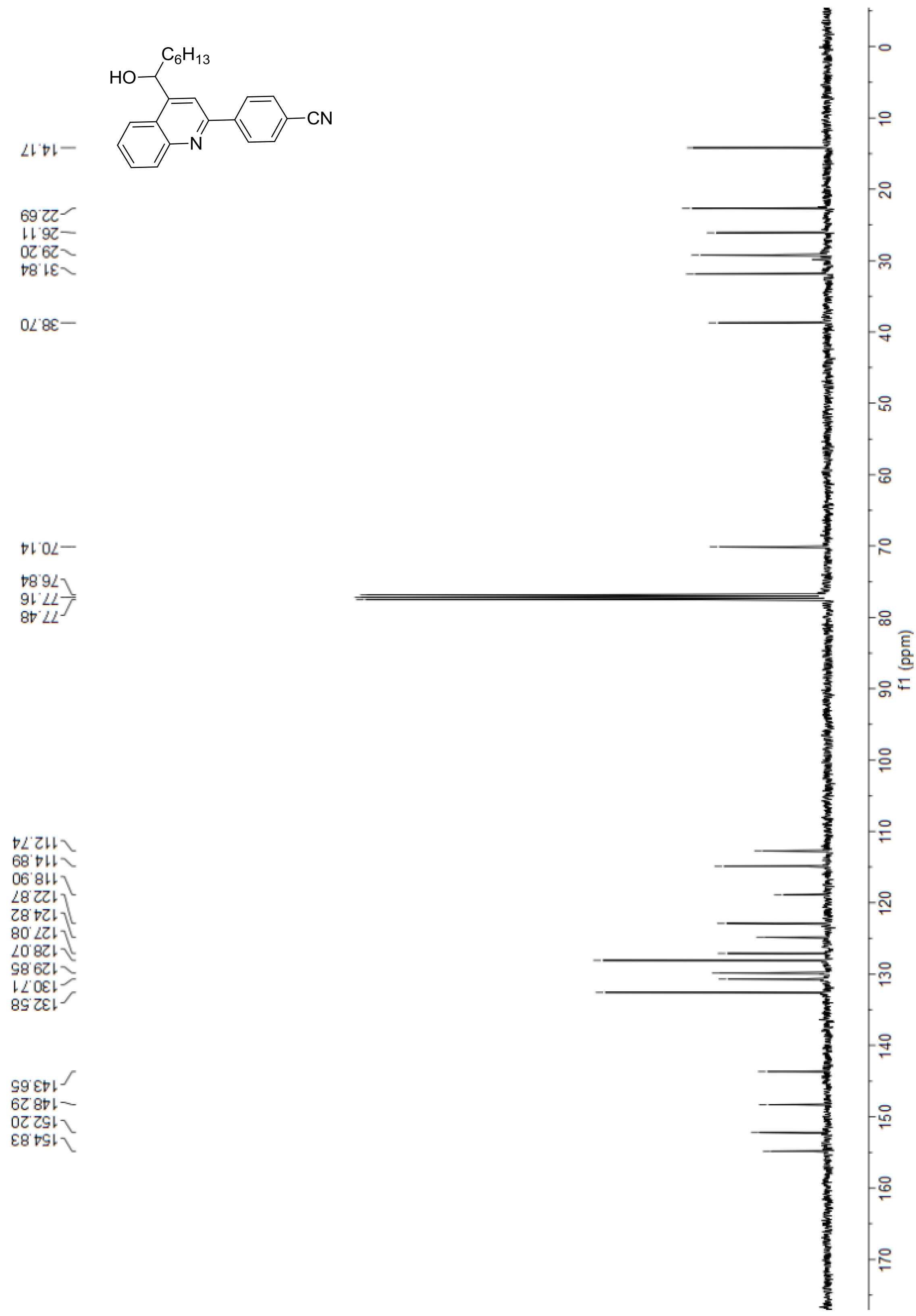

1-(2-(4-methoxyphenyl)quinolin-4-yl)heptan-1-ol (5h)

^{1}H NMR of **5h** ($CDCl_3$, 400 MHz, 25 ºC)

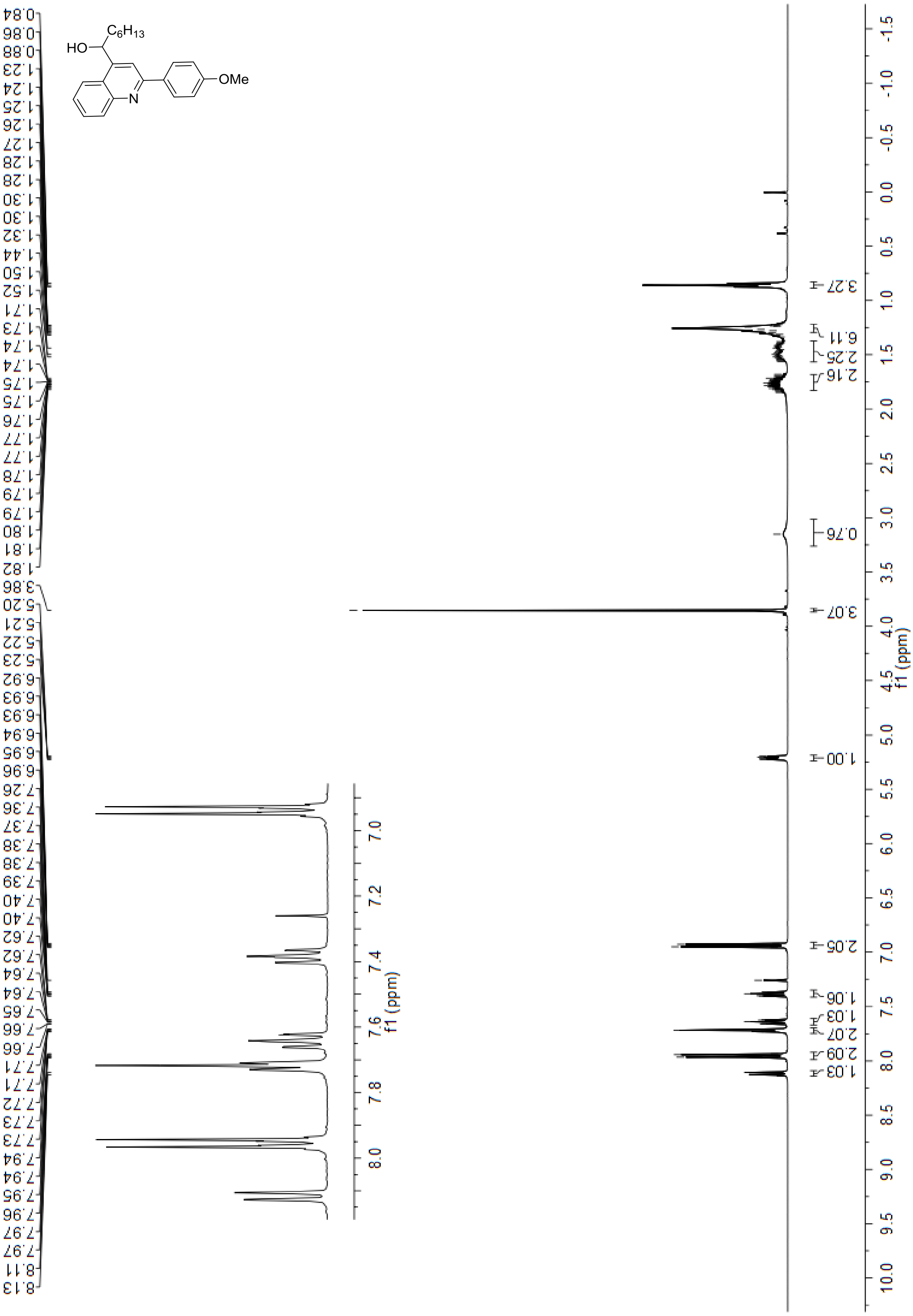

^{13}C NMR of **5h** ($CDCl_3$, 101 MHz, 25 ºC)

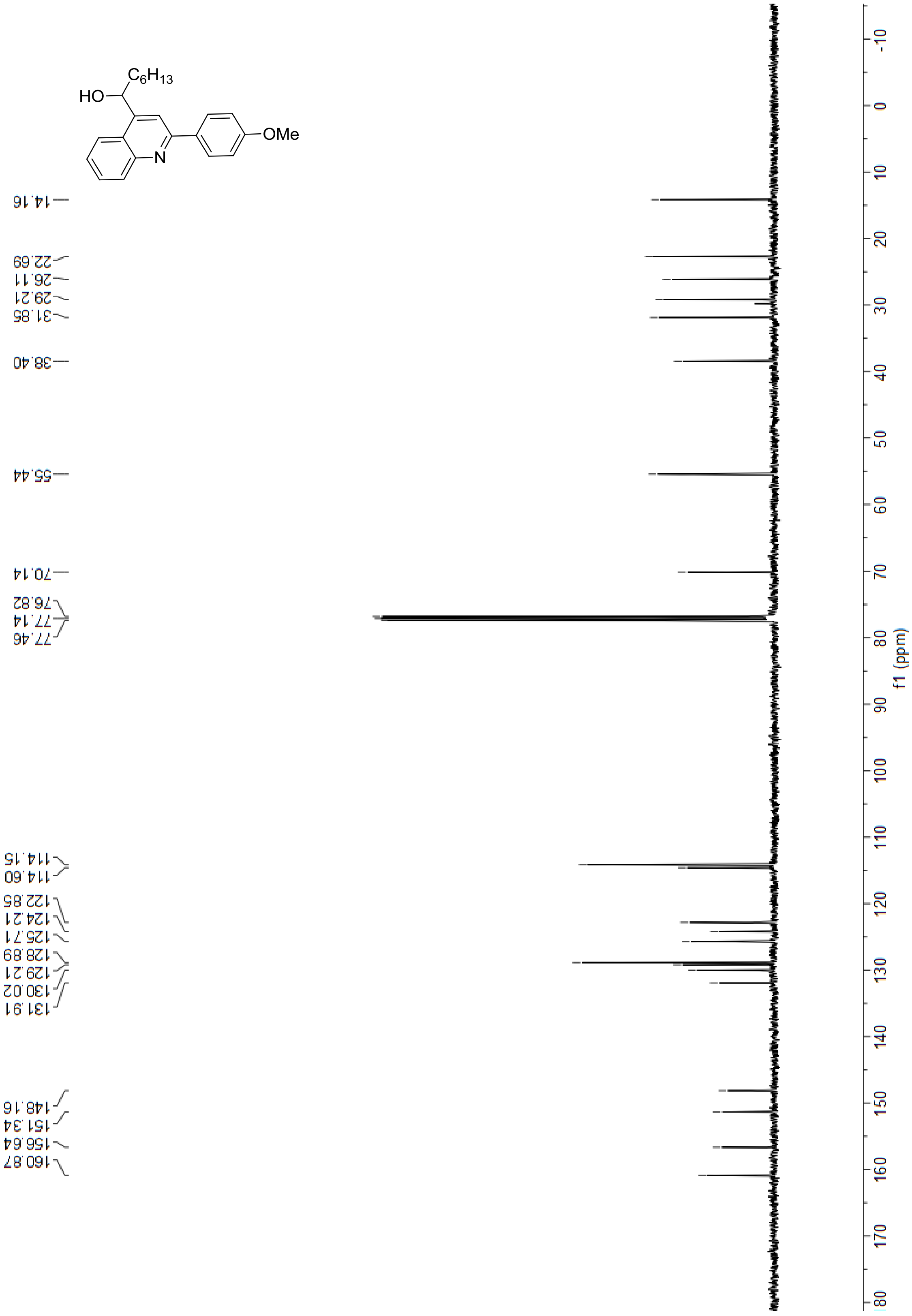

1-(2-(3-methoxyphenyl)quinolin-4-yl)heptan-1-ol (5i)

^{1}H NMR of **5i** ($CDCl_3$, 400 MHz, 25 ºC)

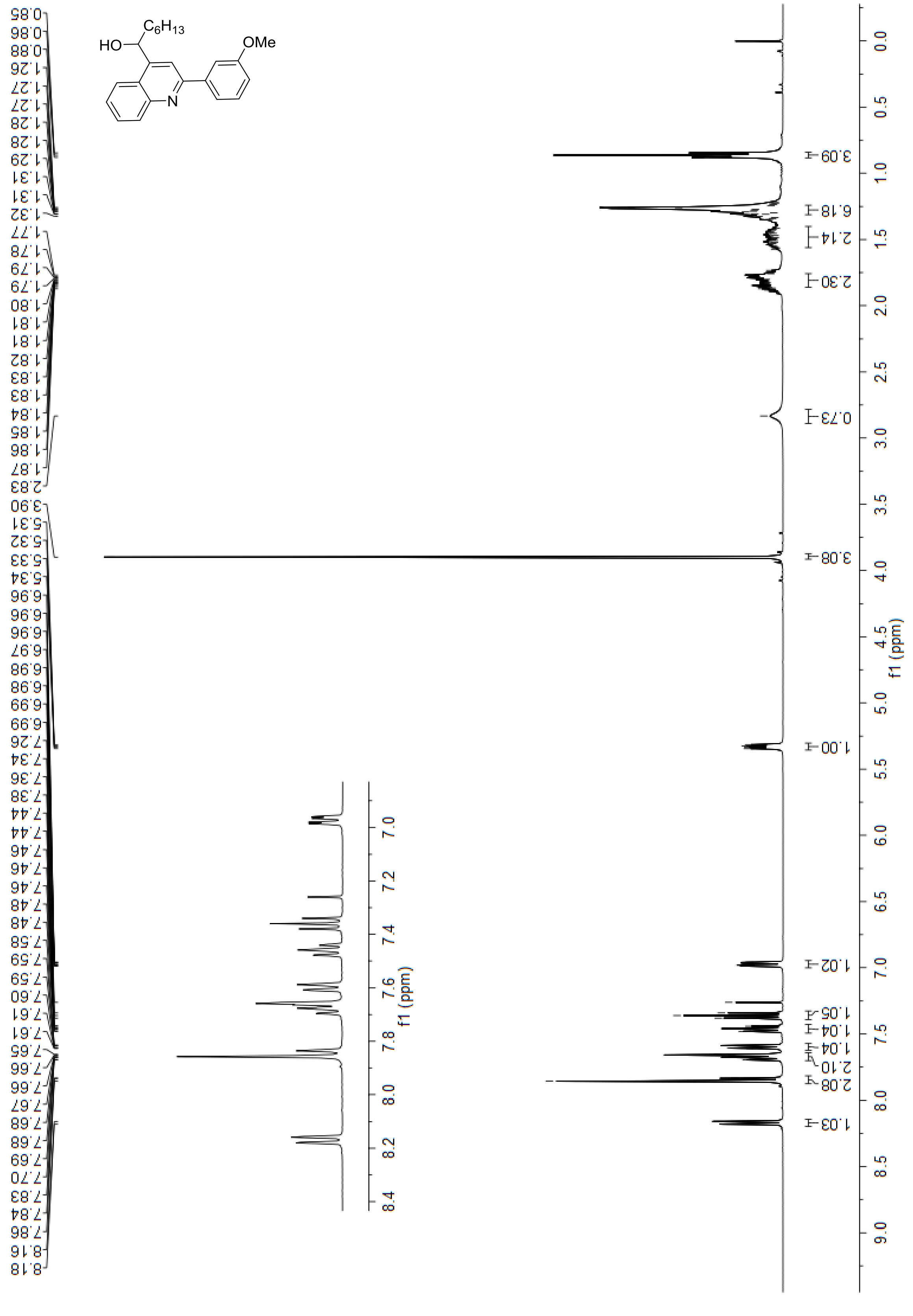

^{13}C NMR of **5i** ($CDCl_3$, 101 MHz, 25 ºC)

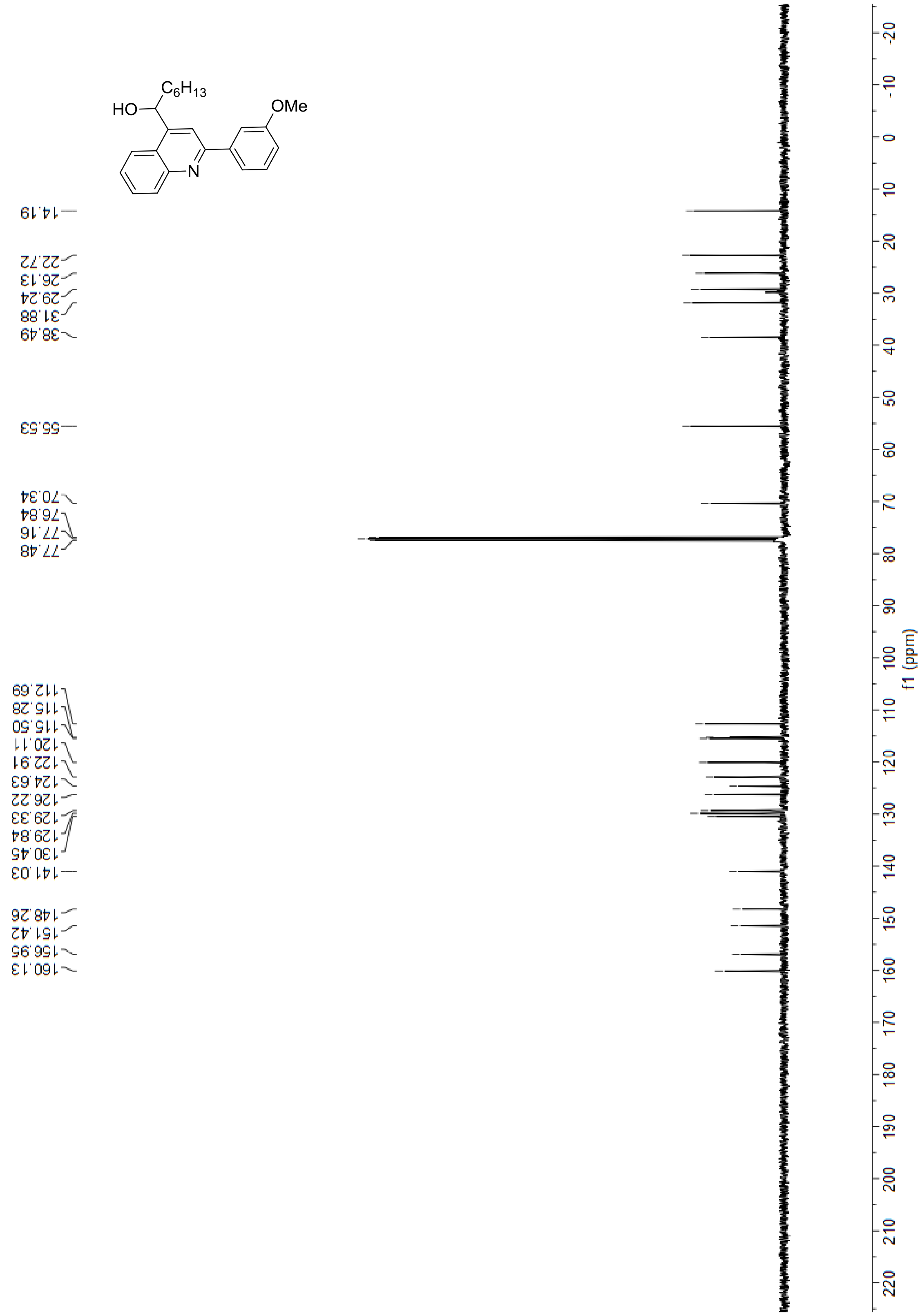

1-(2-(2-Methoxyphenyl)quinolin-4-yl)heptan-1-ol (5j)

^{1}H NMR of **5j** ($CDCl_3$, 400 MHz, 25 ºC)

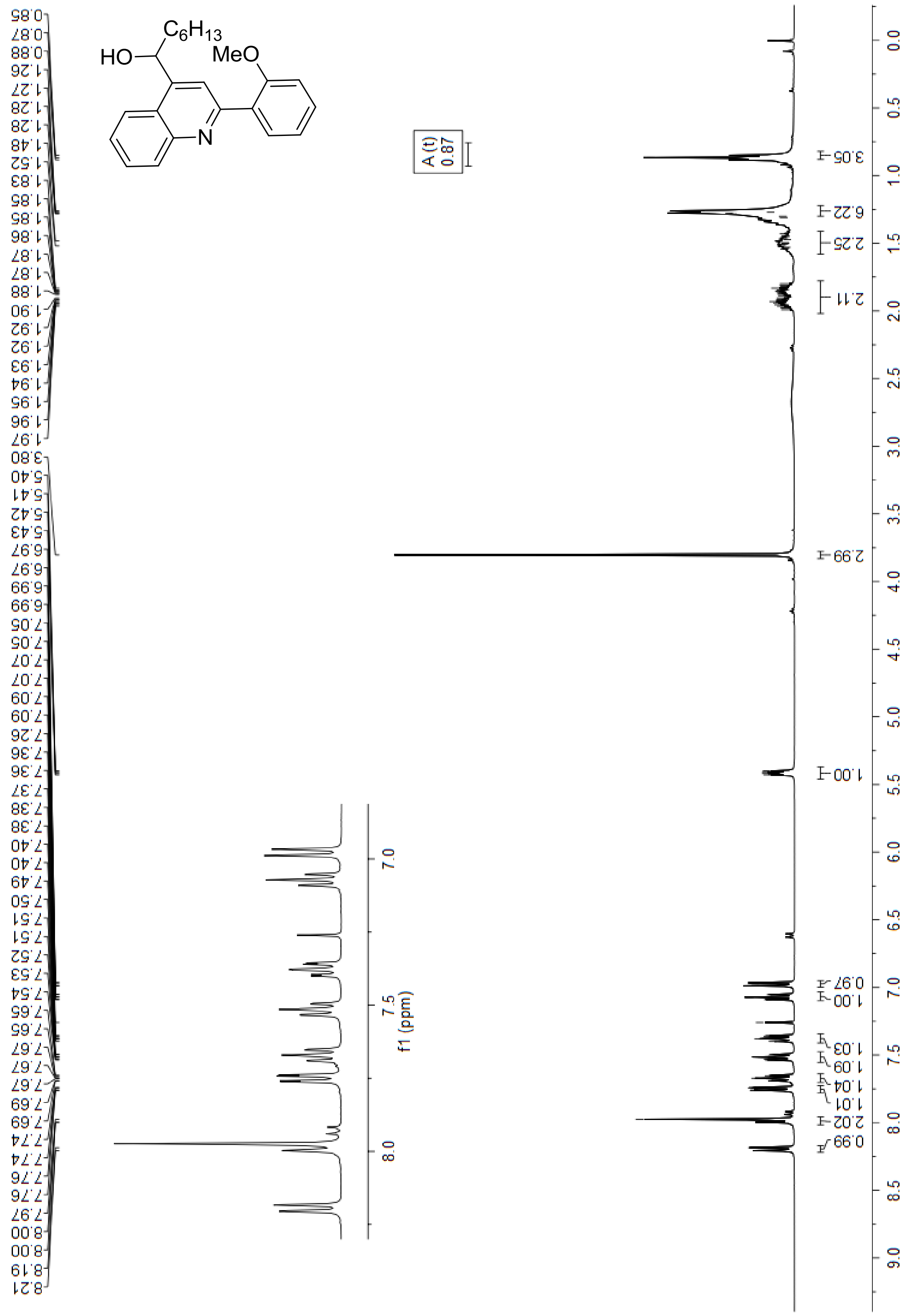

^{13}C NMR of **5j** ($CDCl_3$, 101 MHz, 25 ºC)

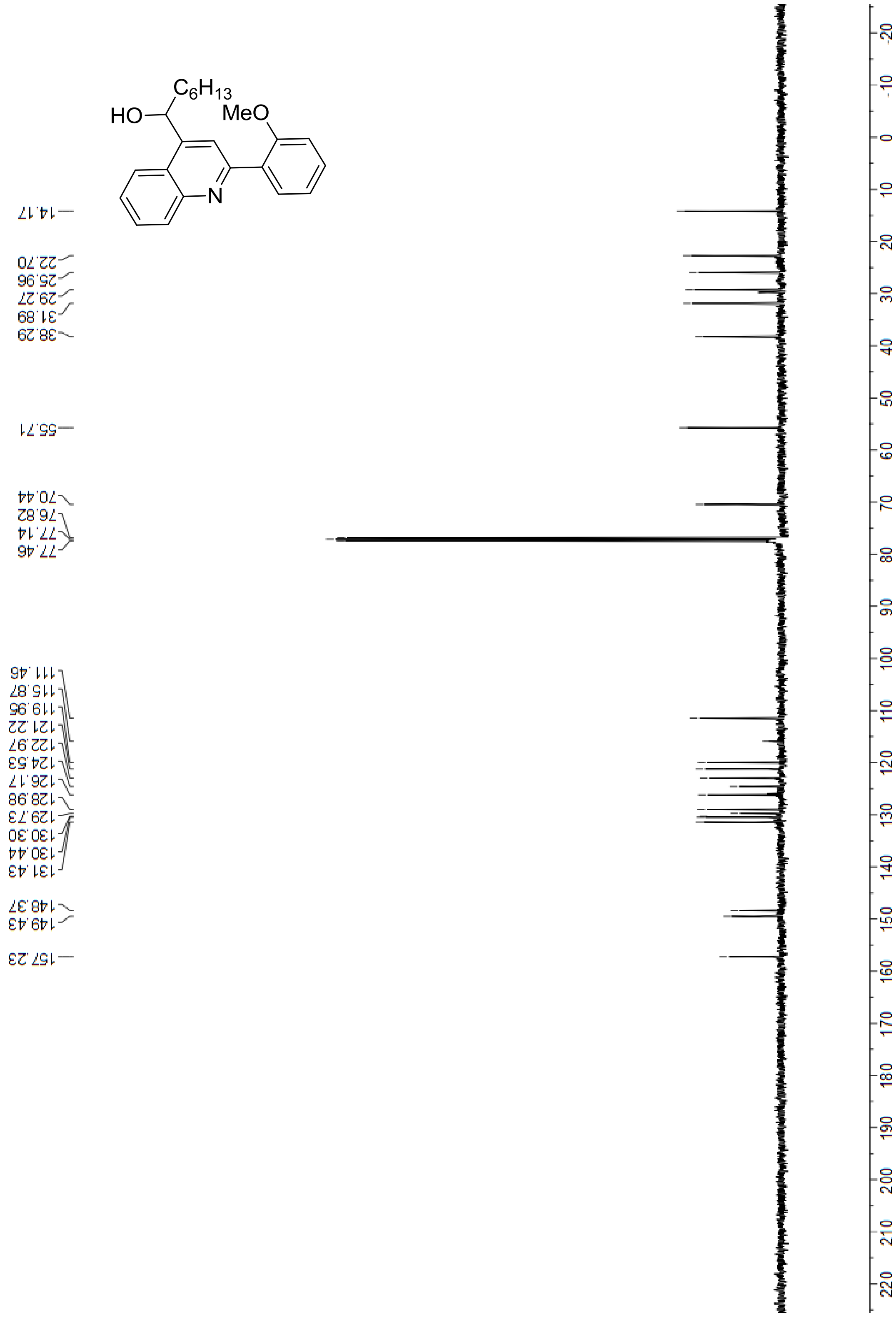

1-(2-(3,5-dimethylphenyl)quinolin-4-yl)heptan-1-ol (5k)

^{1}H NMR of **5k** ($CDCl_3$, 400 MHz, 25 ºC)

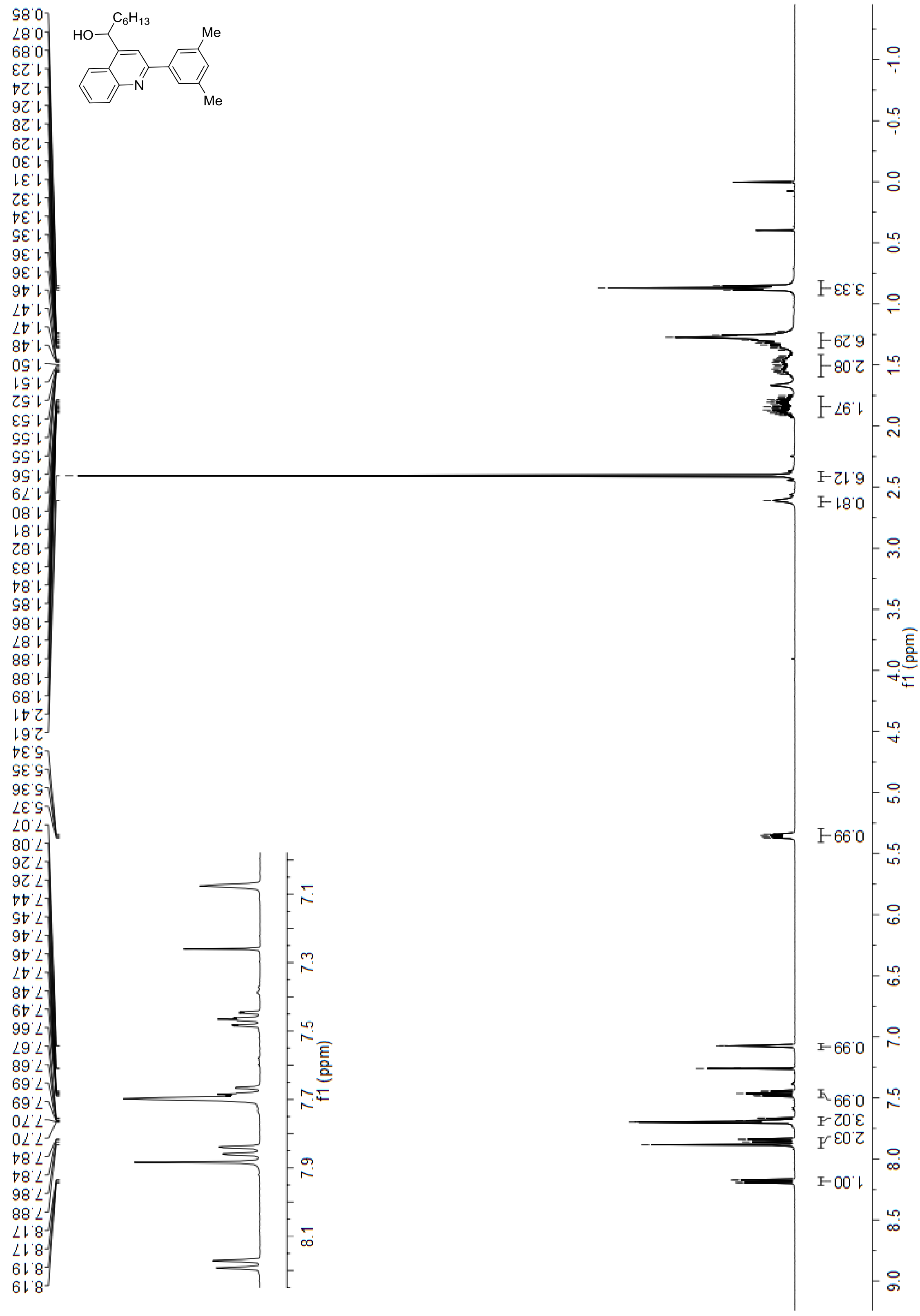

^{13}C NMR of **5k** ($CDCl_3$, 101 MHz, 25 ºC)

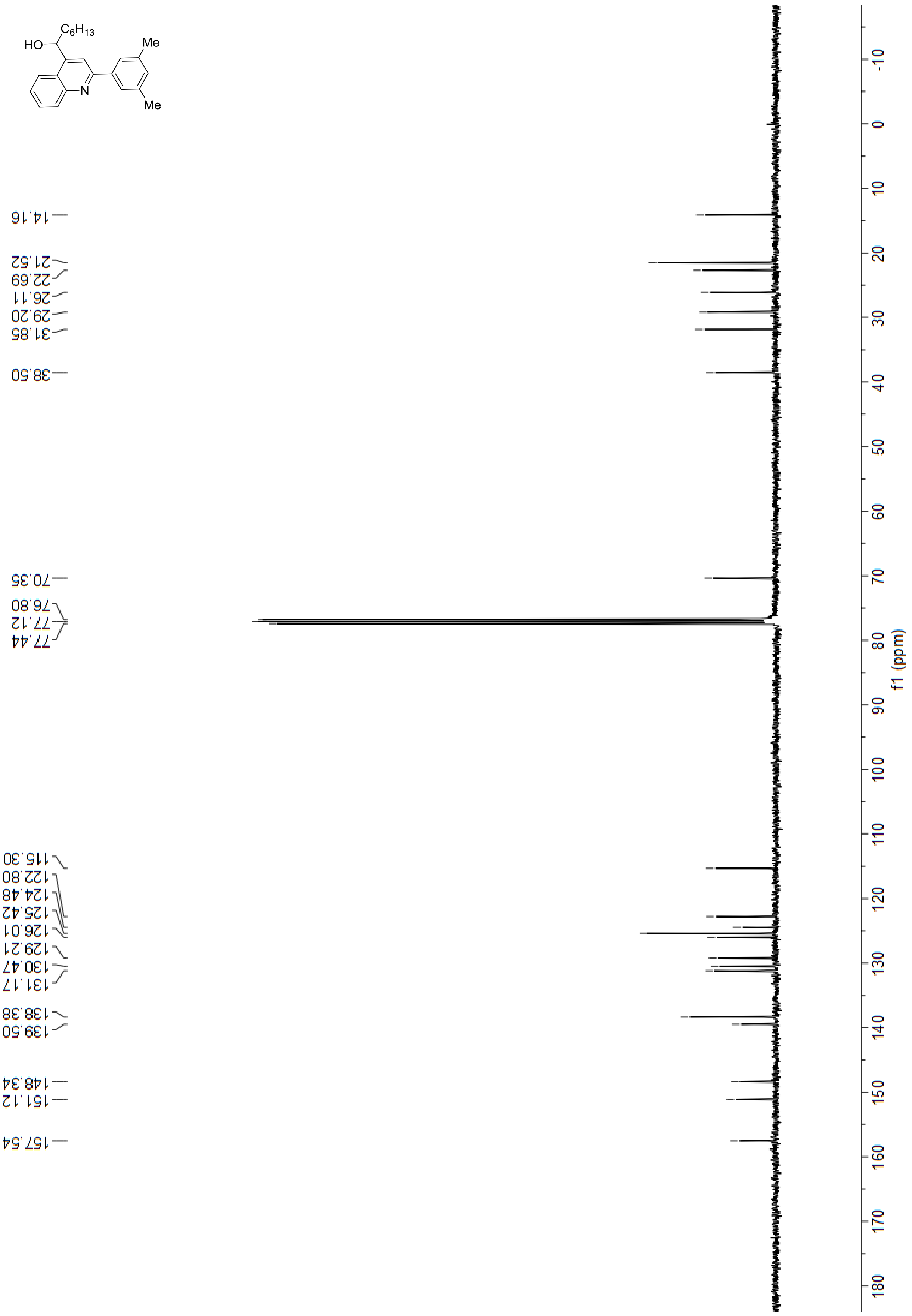

1-(isoquinolin-1-yl)heptan-1-ol (5l)

^{1}H NMR of **5l** ($CDCl_3$, 400 MHz, 25 ºC)

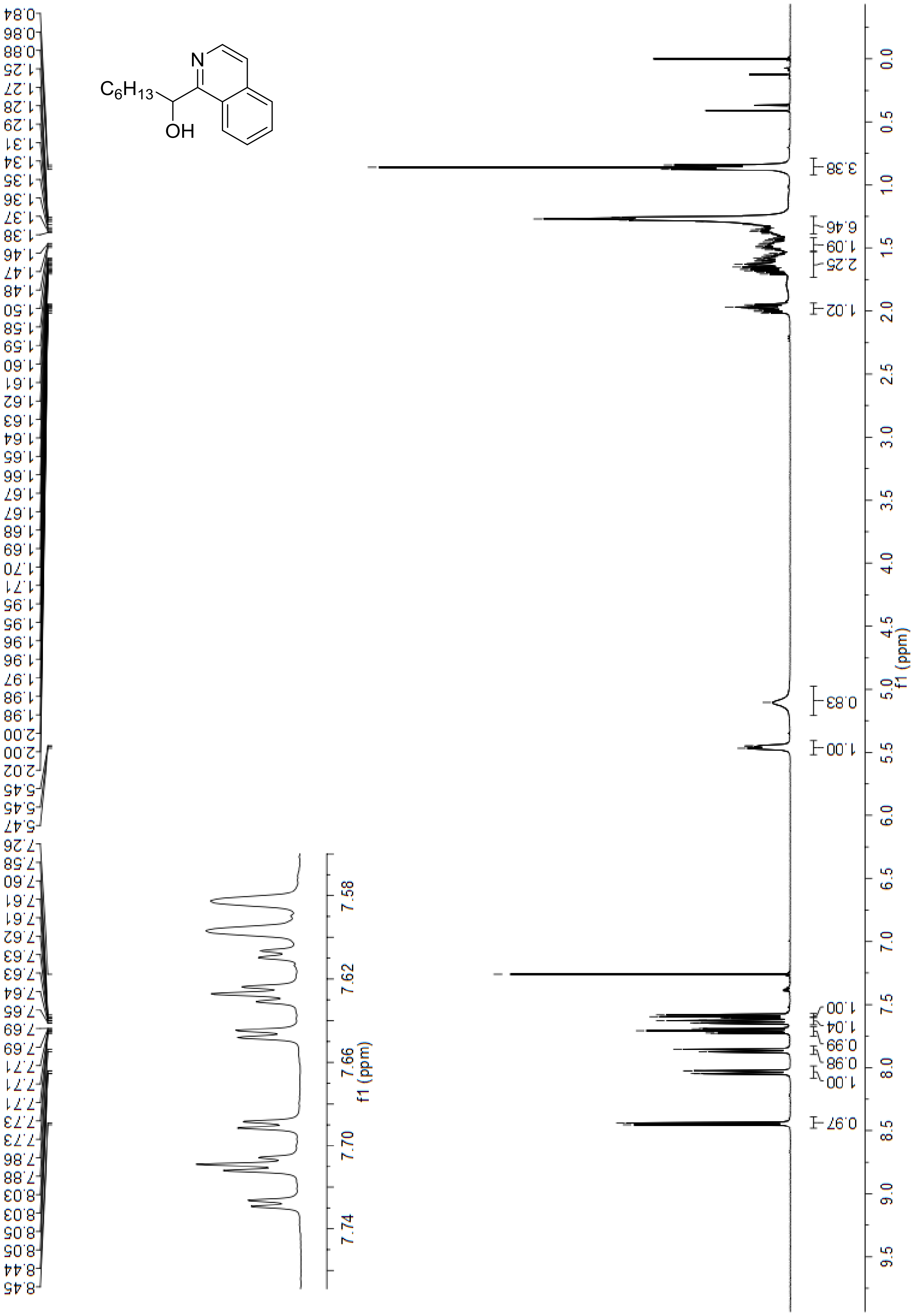

^{13}C NMR of **5l** ($CDCl_3$, 101 MHz, 25 ºC)

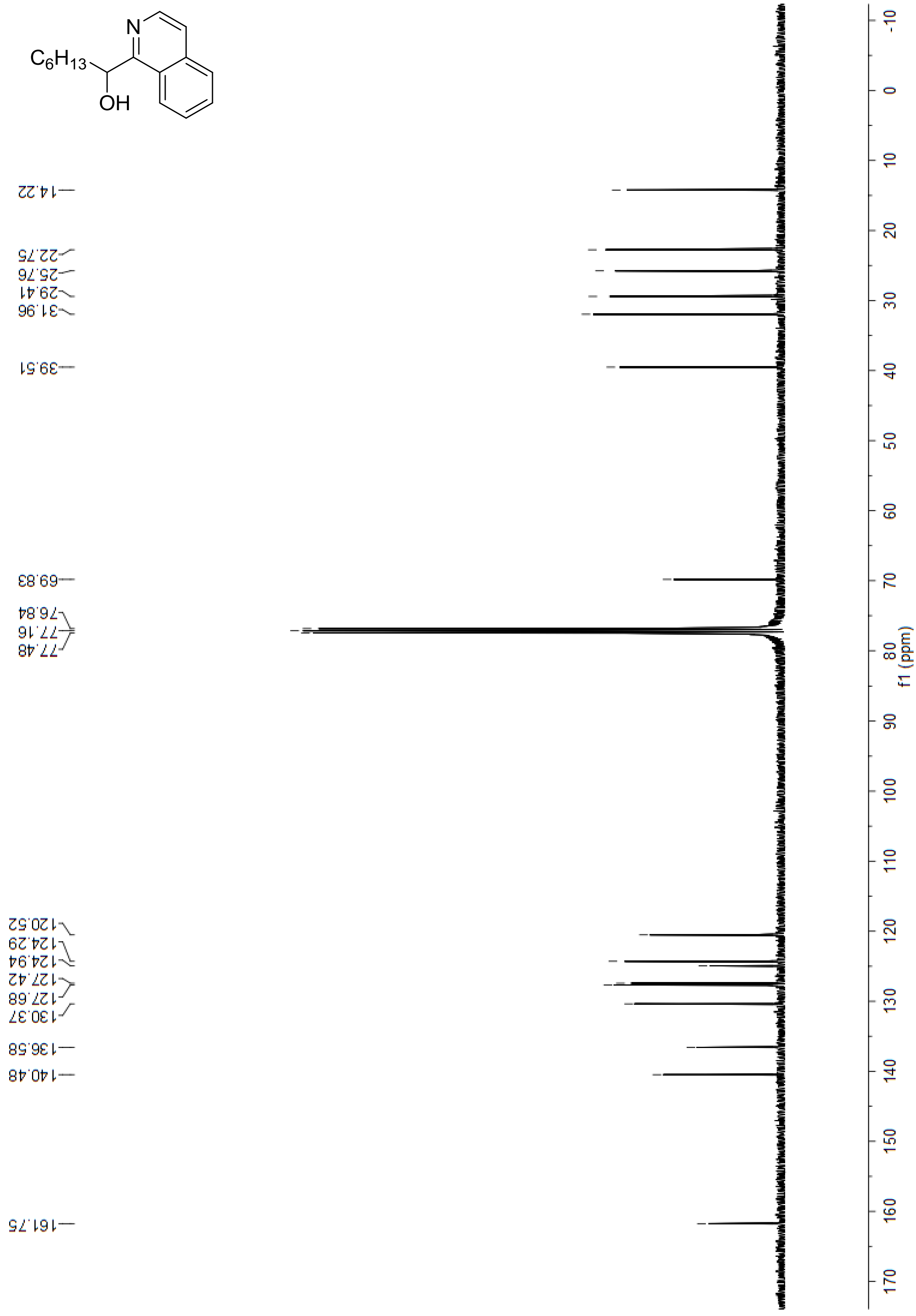

1-(5-bromoisoquinolin-1-yl)heptan-1-ol (5m)

^{1}H NMR of **5m** ($CDCl_3$, 400 MHz, 25 ºC)

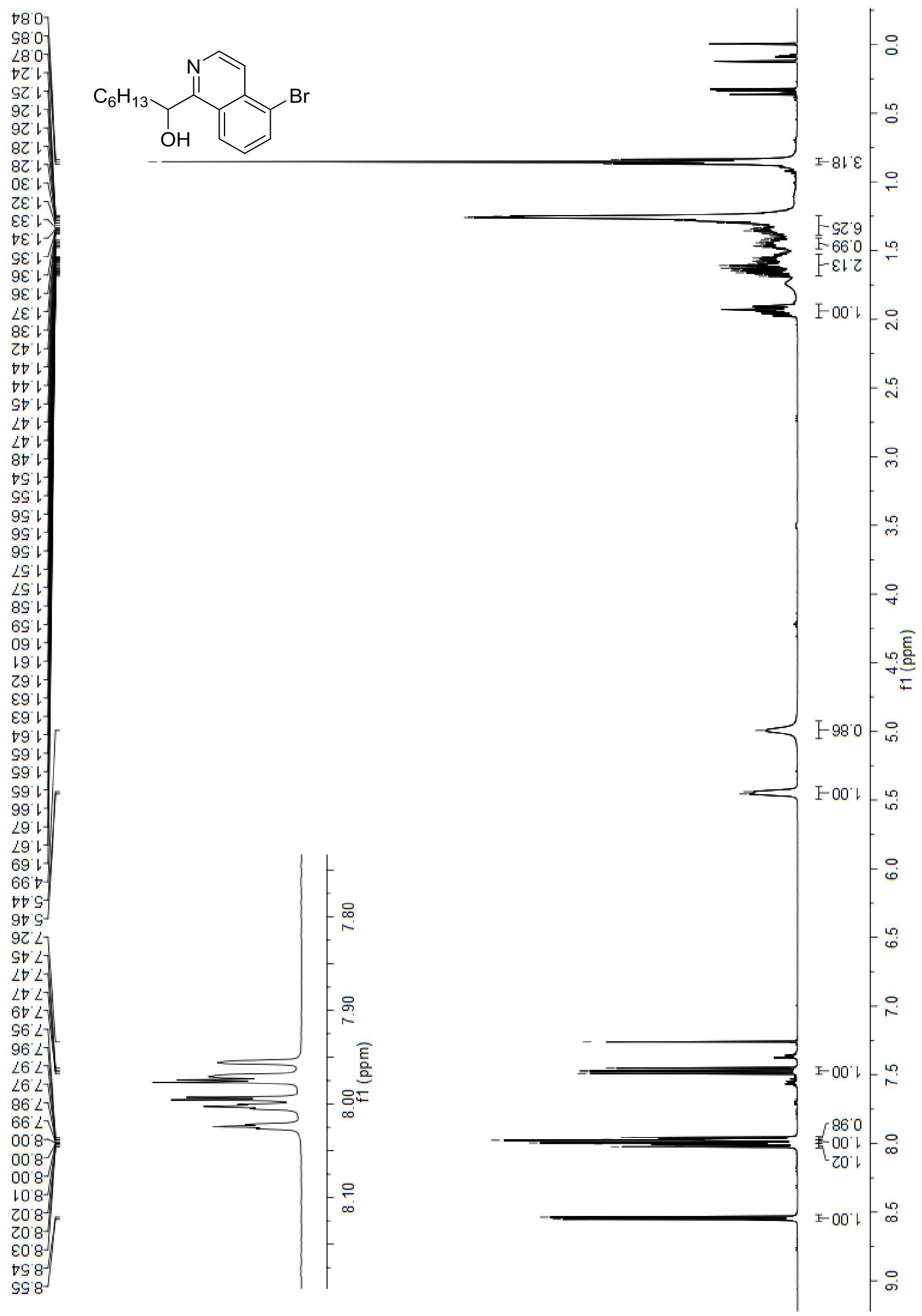

^{13}C NMR of **5m** ($CDCl_3$, 101 MHz, 25 ºC)

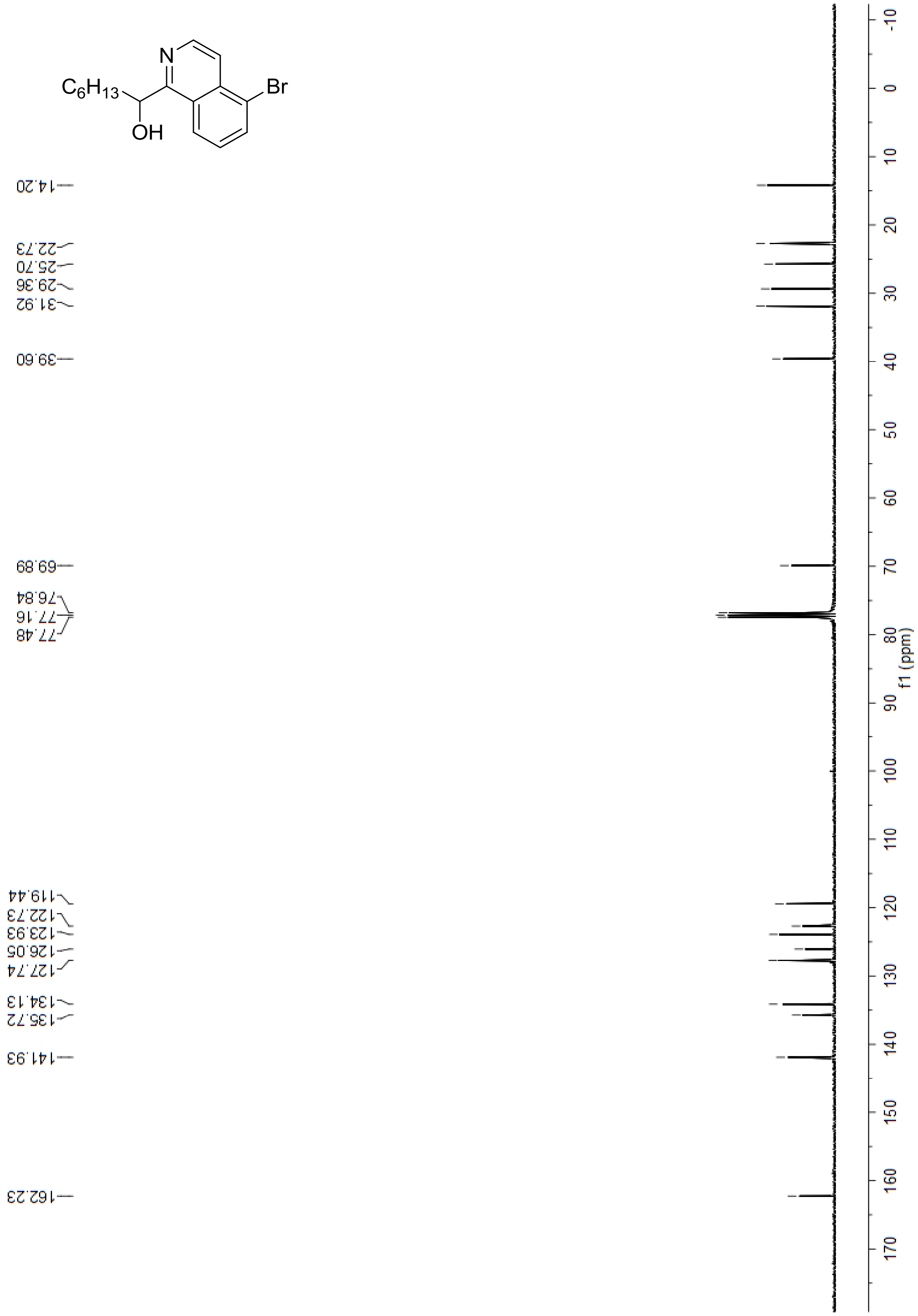

1-(5-methoxyisoquinolin-1-yl)heptan-1-ol (5n)

^{1}H NMR of **5n** ($CDCl_3$, 400 MHz, 25 ºC)

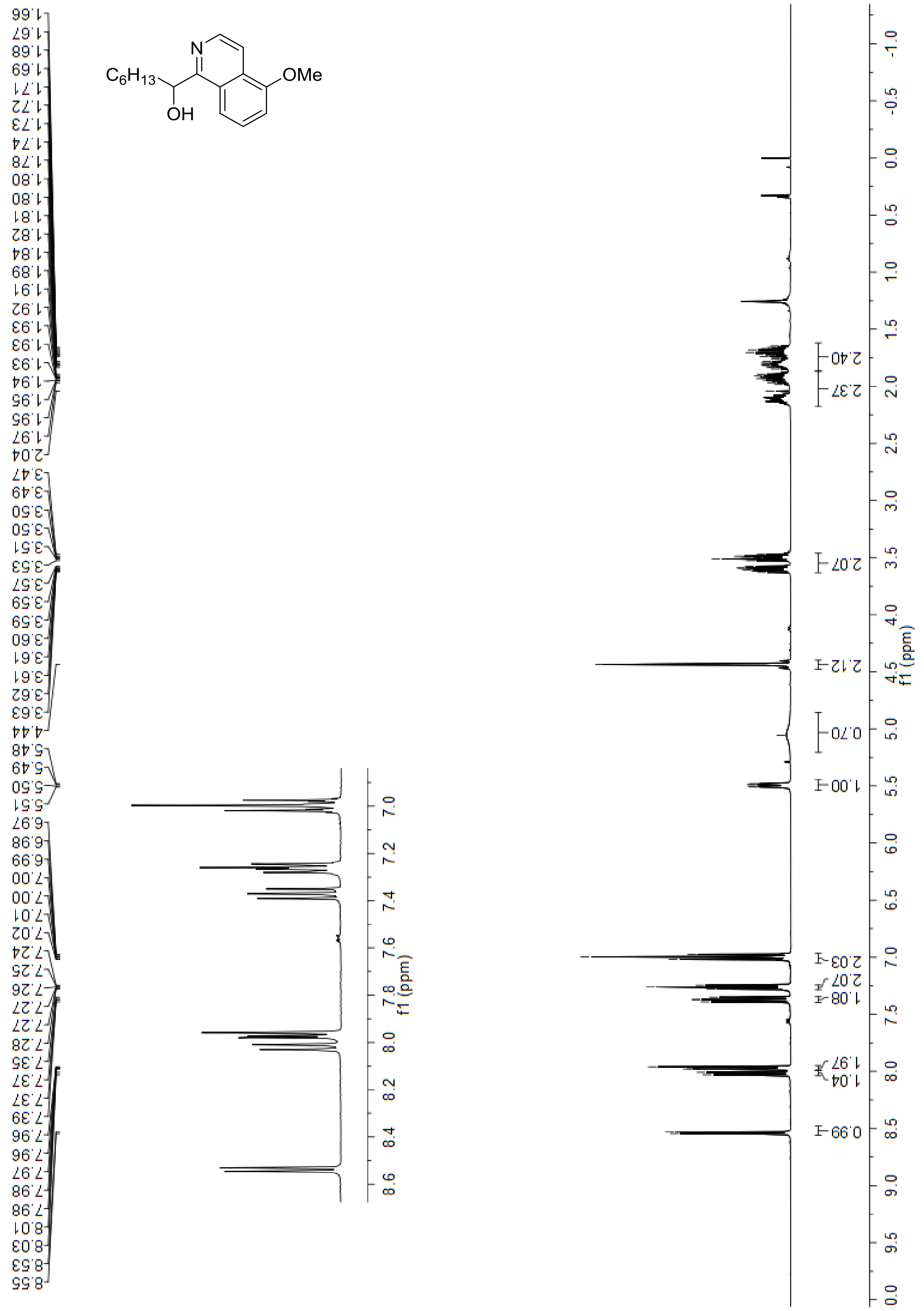

^{13}C NMR of **5n** (CDCl$_3$, 101 MHz, 25 ºC)

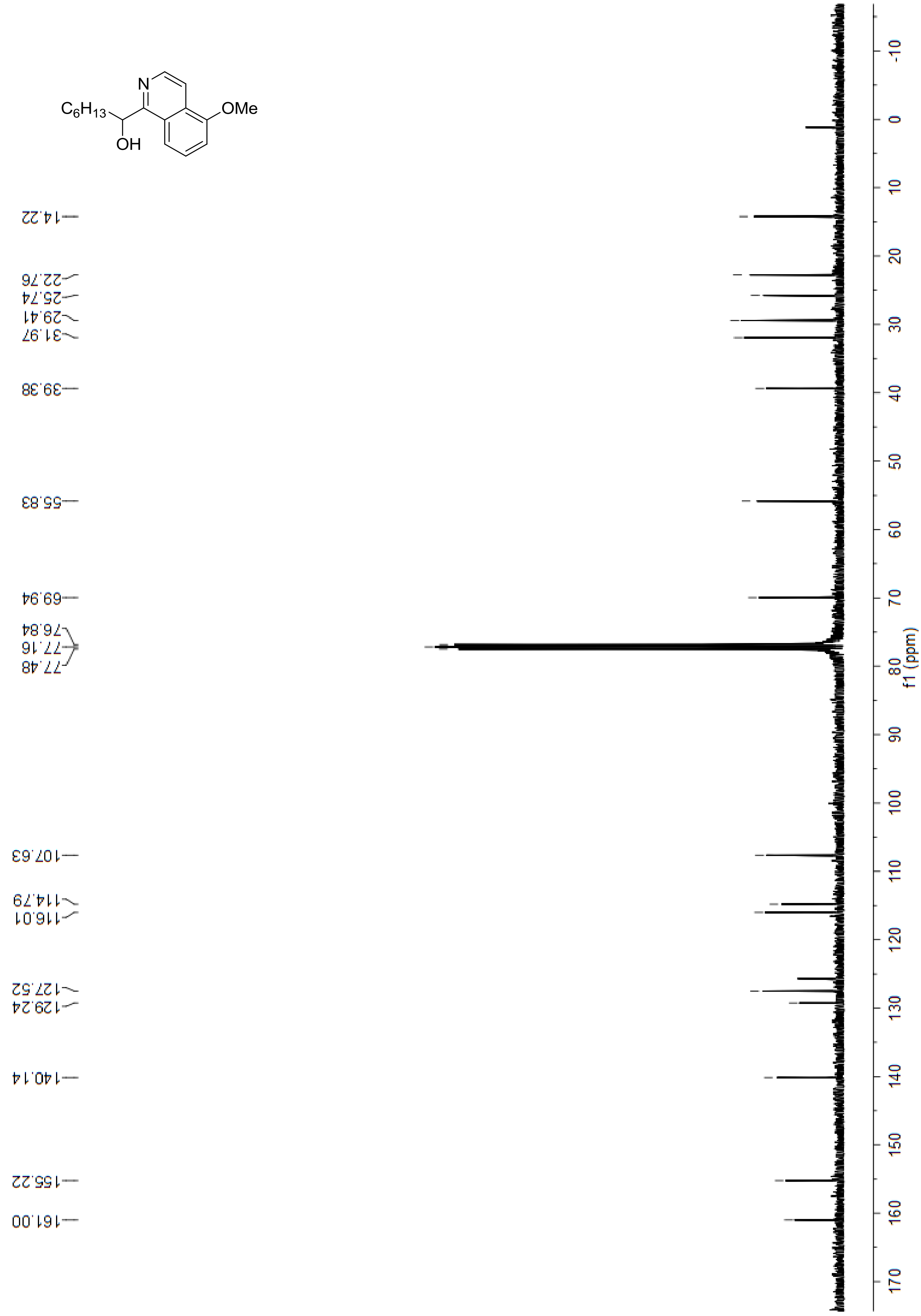

1-(5-(4-methoxyphenyl)isoquinolin-1-yl)heptan-1-ol (5o)

^{1}H NMR of **5o** ($CDCl_3$, 400 MHz, 25 ºC)

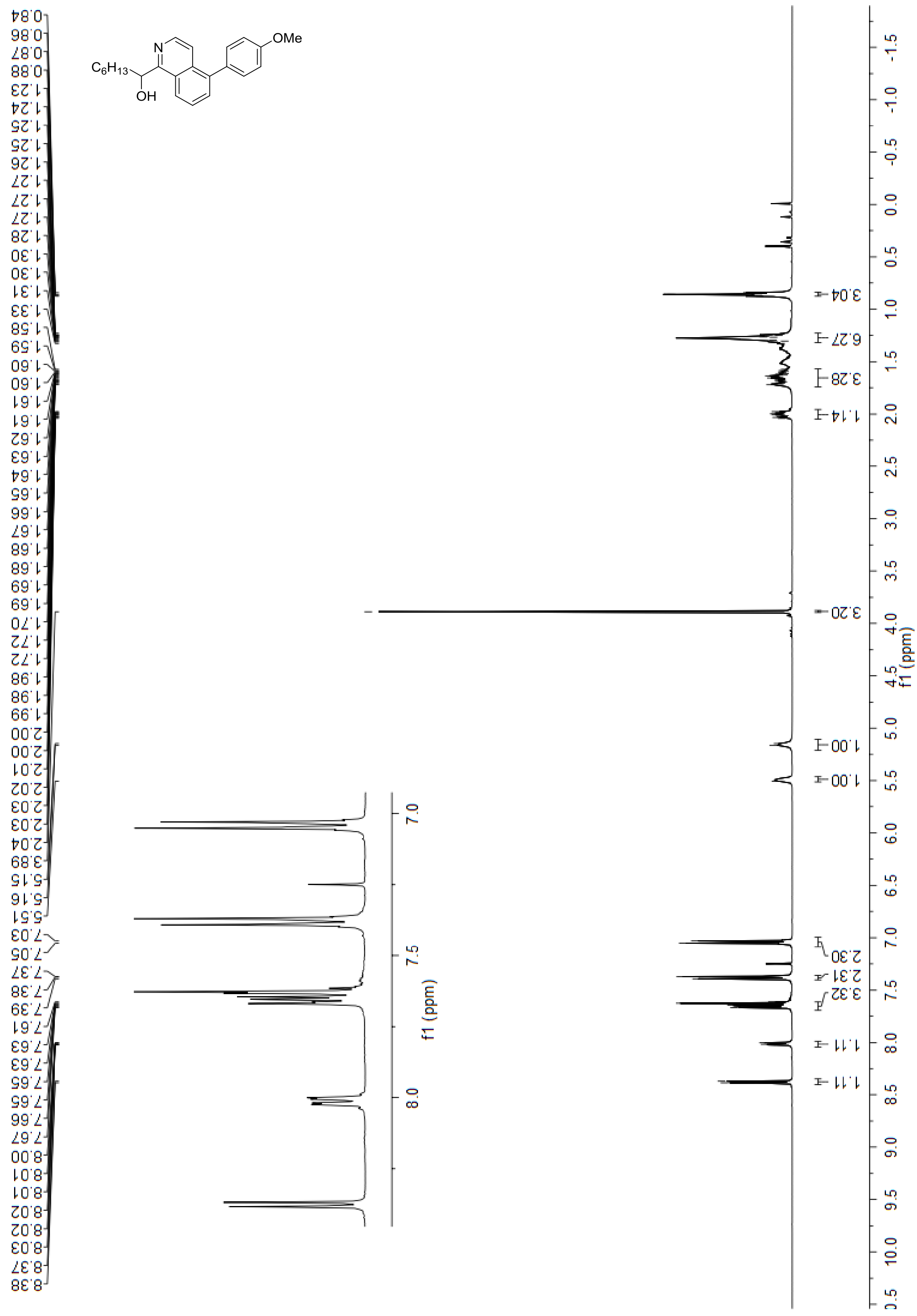

^{13}C NMR of **5o** ($CDCl_3$, 101 MHz, 25 ºC)

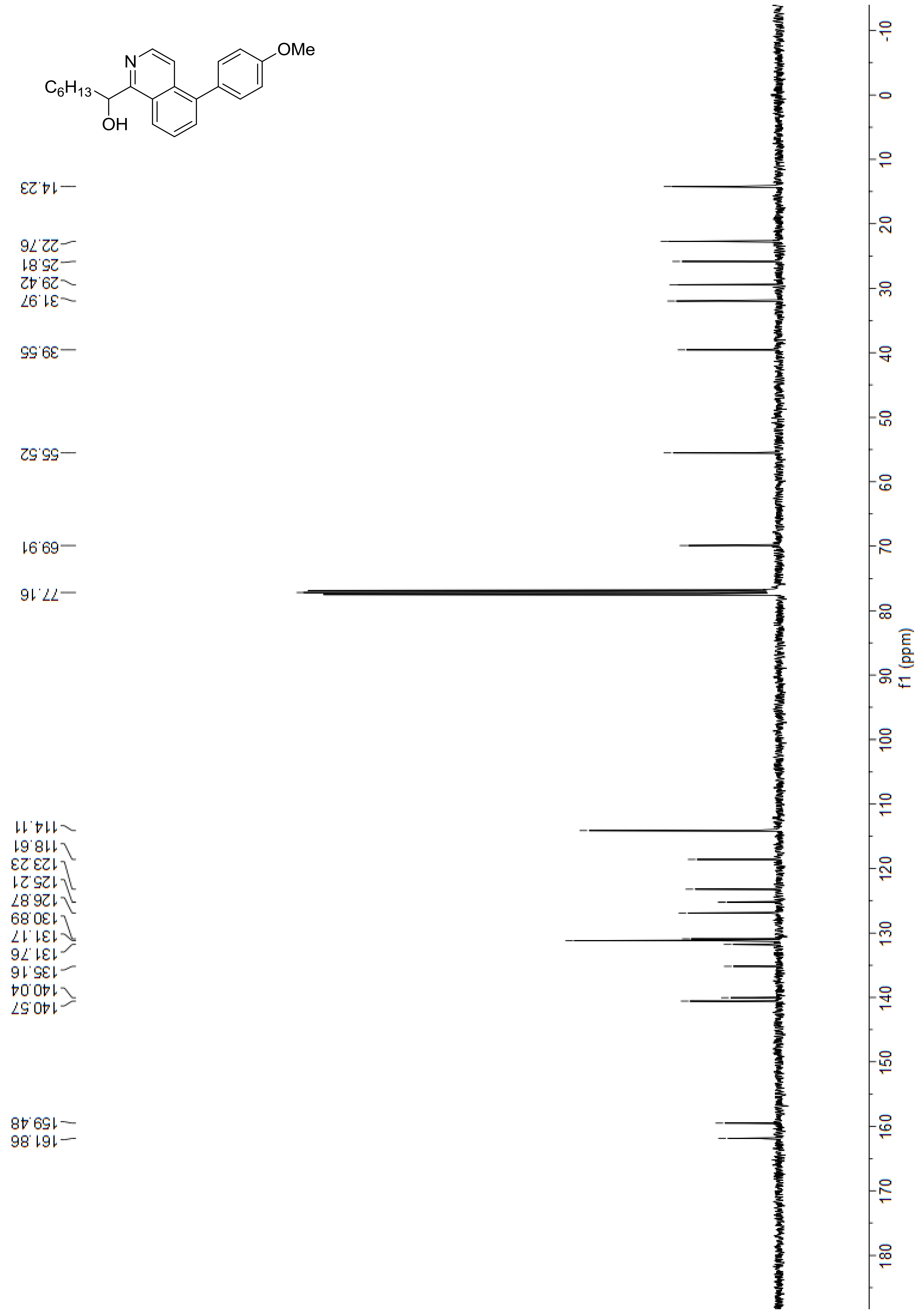

1-(5-(4-chlorophenyl)isoquinolin-1-yl)heptan-1-ol(5p)

^{1}H NMR of **5p** ($CDCl_3$, 400 MHz, 25 ºC)

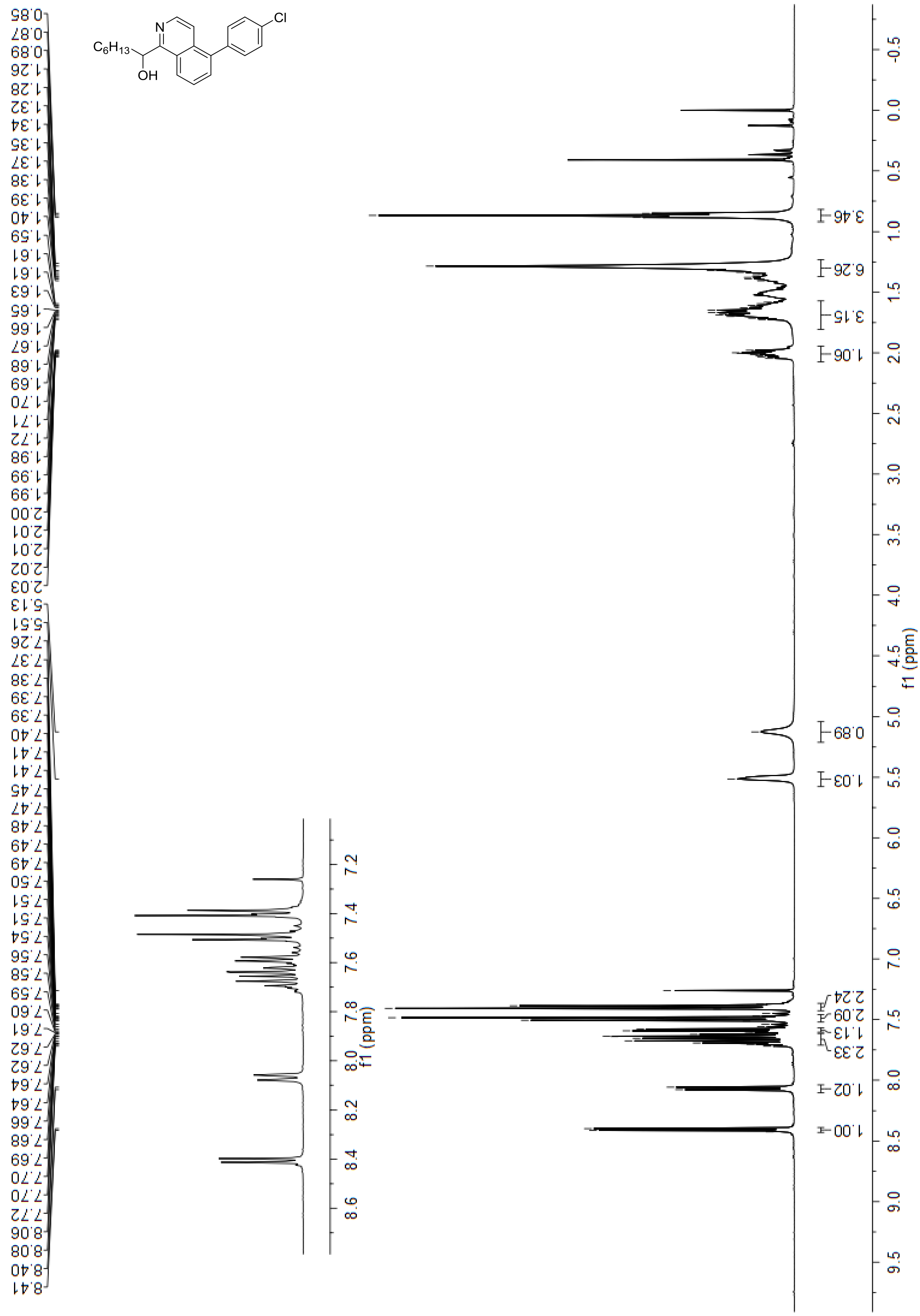

^{13}C NMR of **5p** ($CDCl_3$, 101 MHz, 25 ºC)

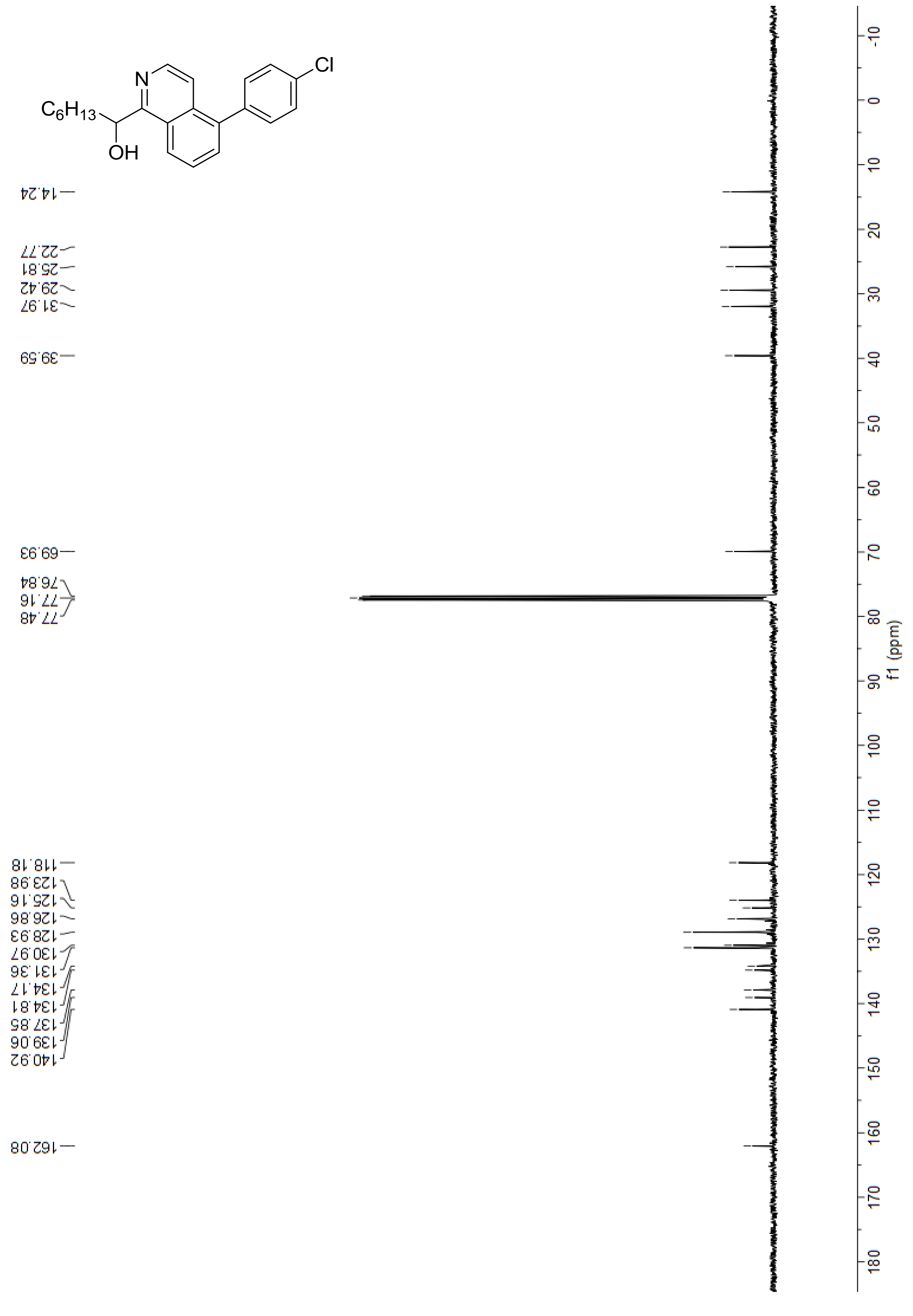

1-(5-([1,1'-biphenyl]-4-yl)isoquinolin-1-yl)heptan-1-ol (5q)

^{1}H NMR of **5q** ($CDCl_3$, 400 MHz, 25 ºC)

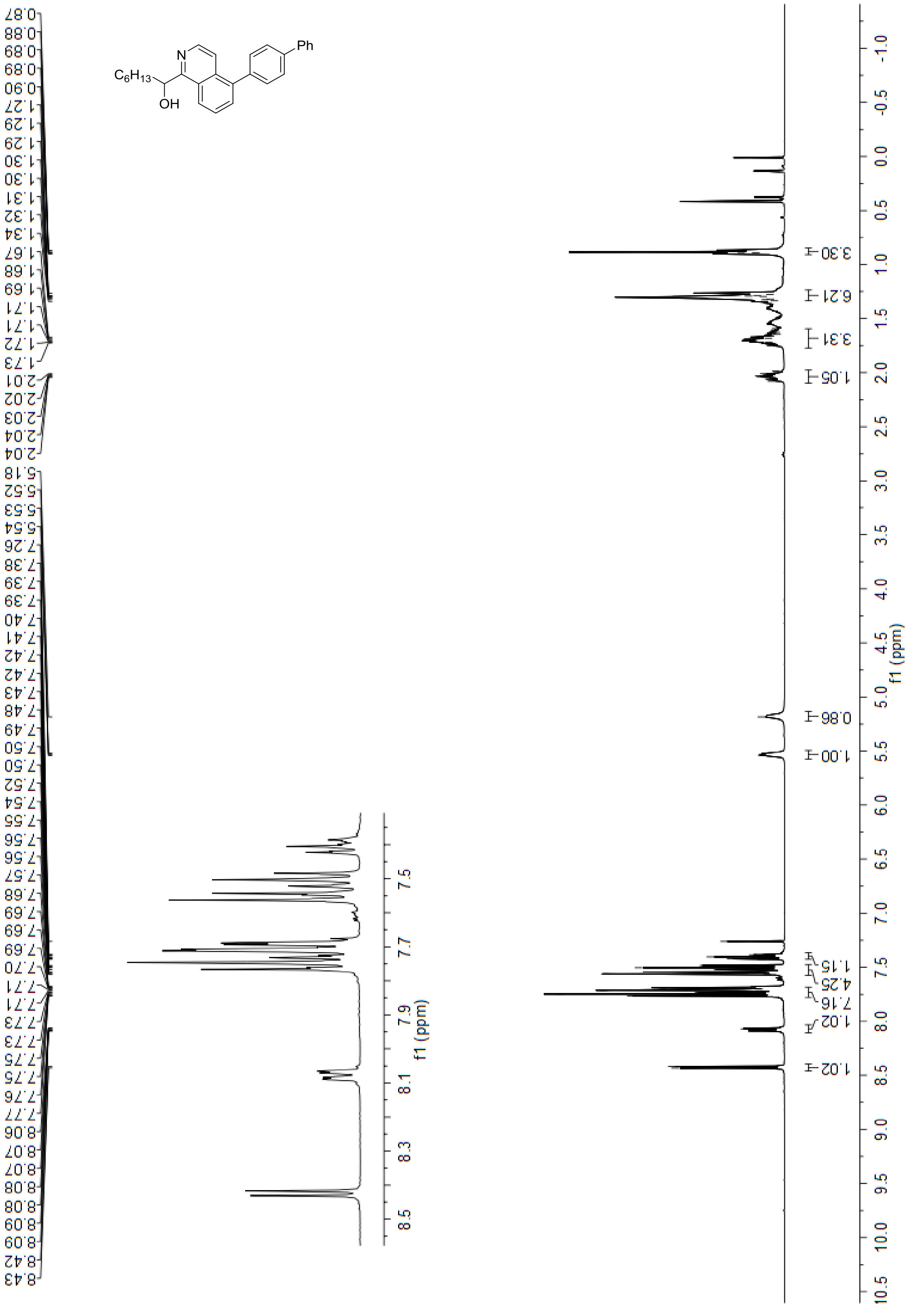

^{13}C NMR of **5q** ($CDCl_3$, 101 MHz, 25 ºC)

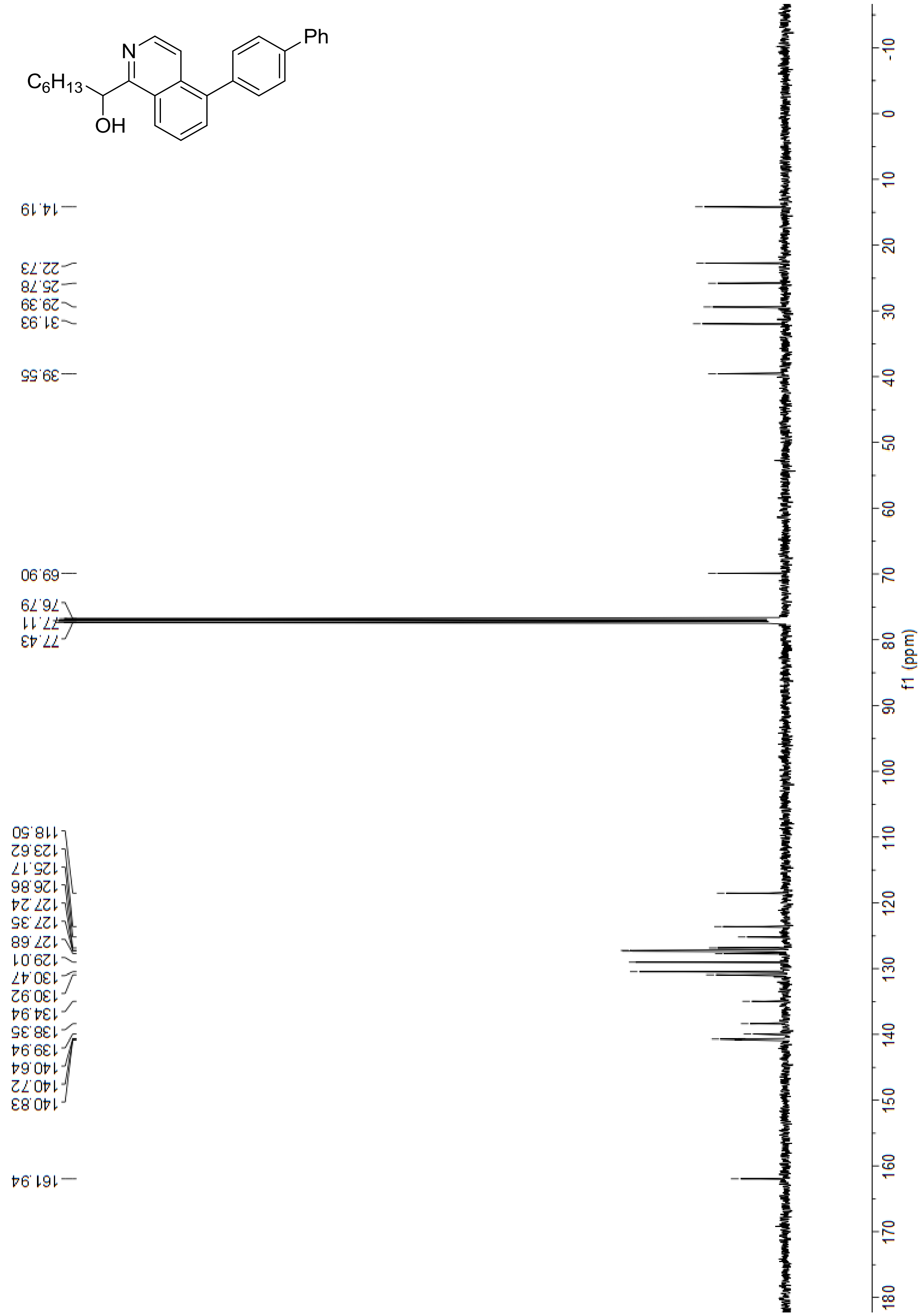

1-(4-bromoisoquinolin-1-yl)dodecan-1-ol (5r)

^{1}H NMR of **5r** ($CDCl_3$, 400 MHz, 25 ºC)

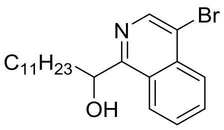

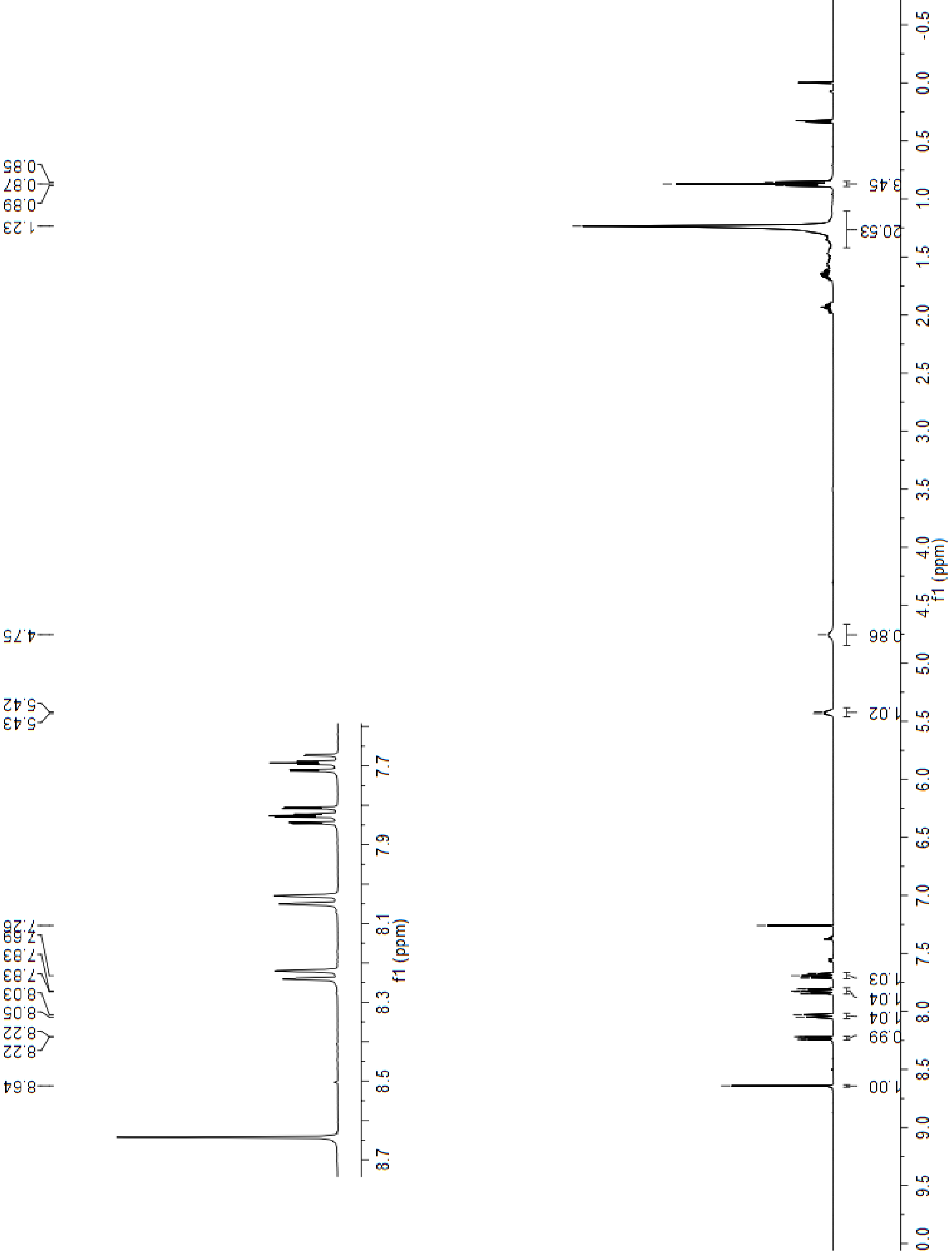

^{13}C NMR of **5r** ($CDCl_3$, 101 MHz, 25 ºC)

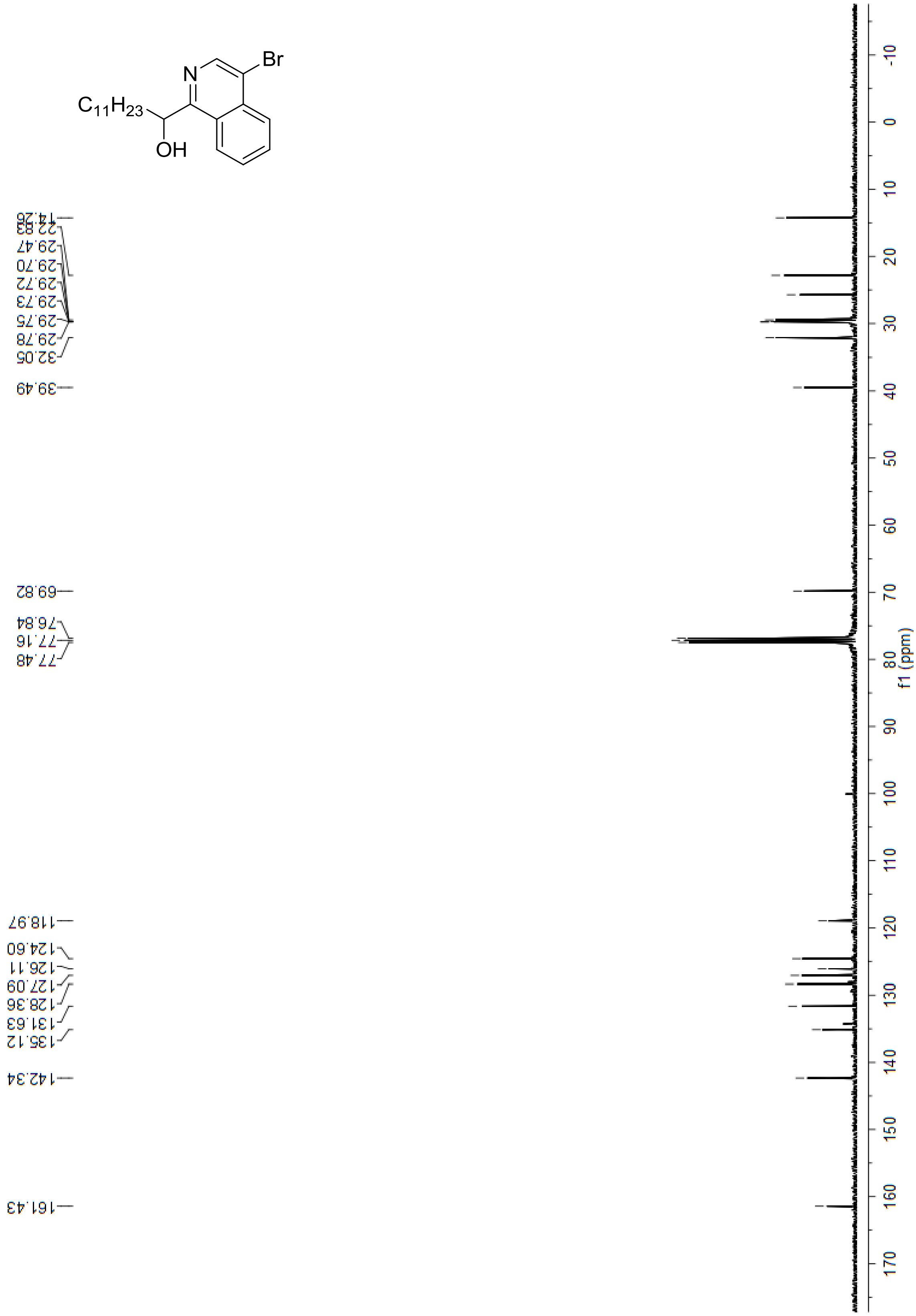

1-(phenanthridin-6-yl)heptan-1-ol (5s)

^{1}H NMR of **5s** ($CDCl_3$, 400 MHz, 25 ºC)

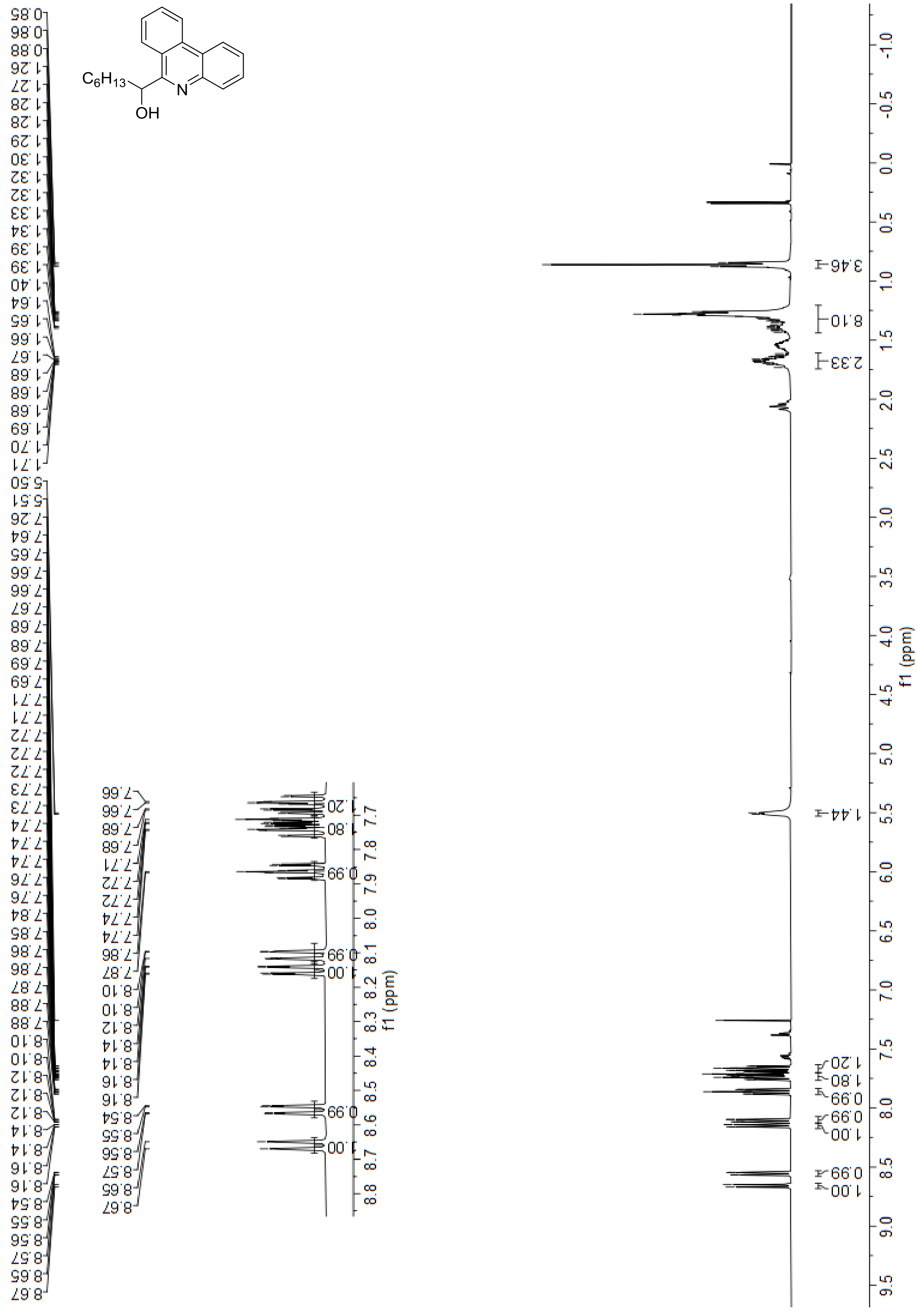

^{13}C NMR of **5s** ($CDCl_3$, 101 MHz, 25 ºC)

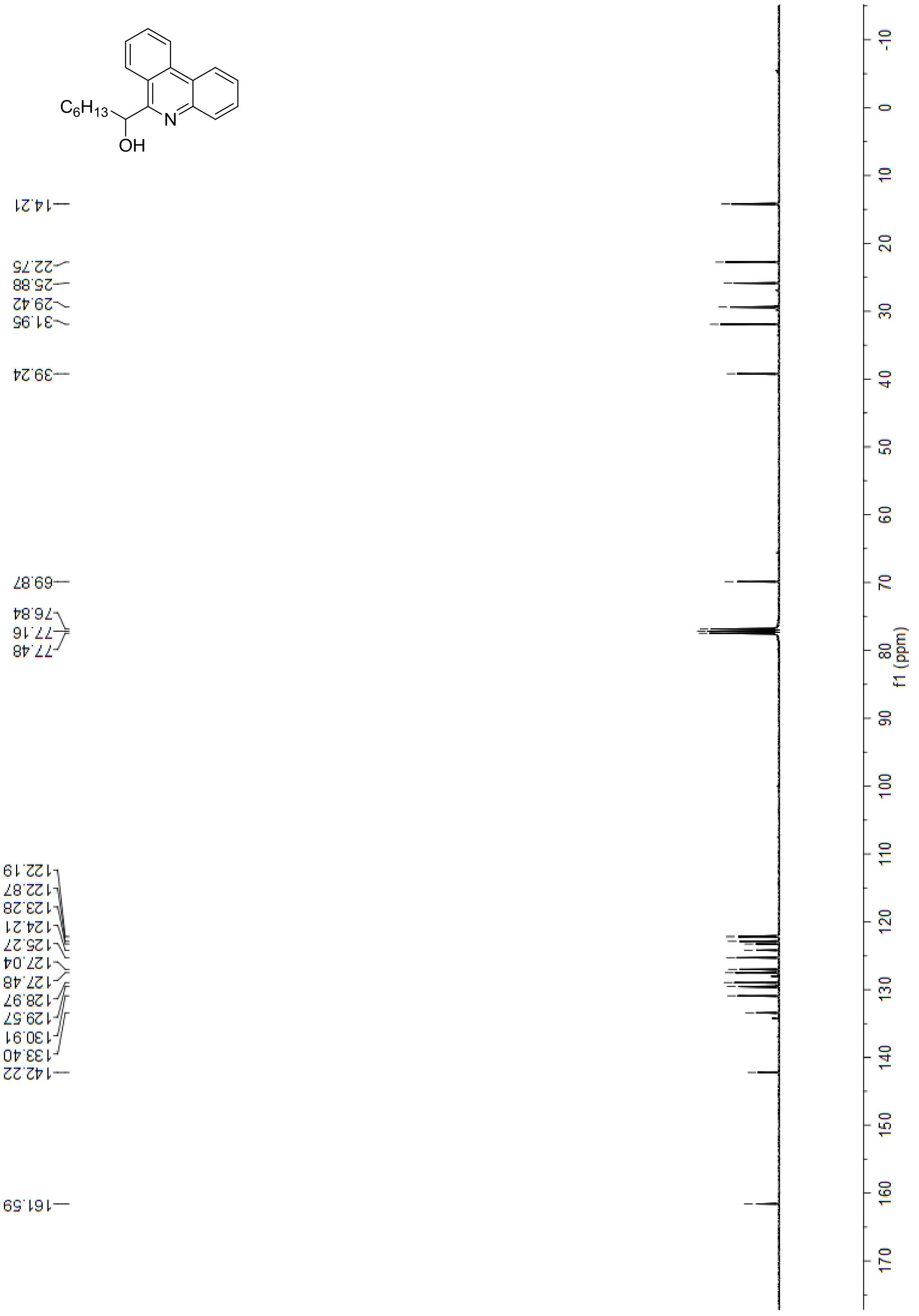

1-(5-bromoisoquinolin-1-yl)dodecan-1-ol (5t)

^{1}H NMR of **5t** ($CDCl_3$, 400 MHz, 25 ºC)

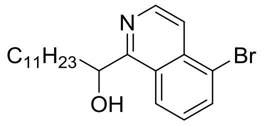

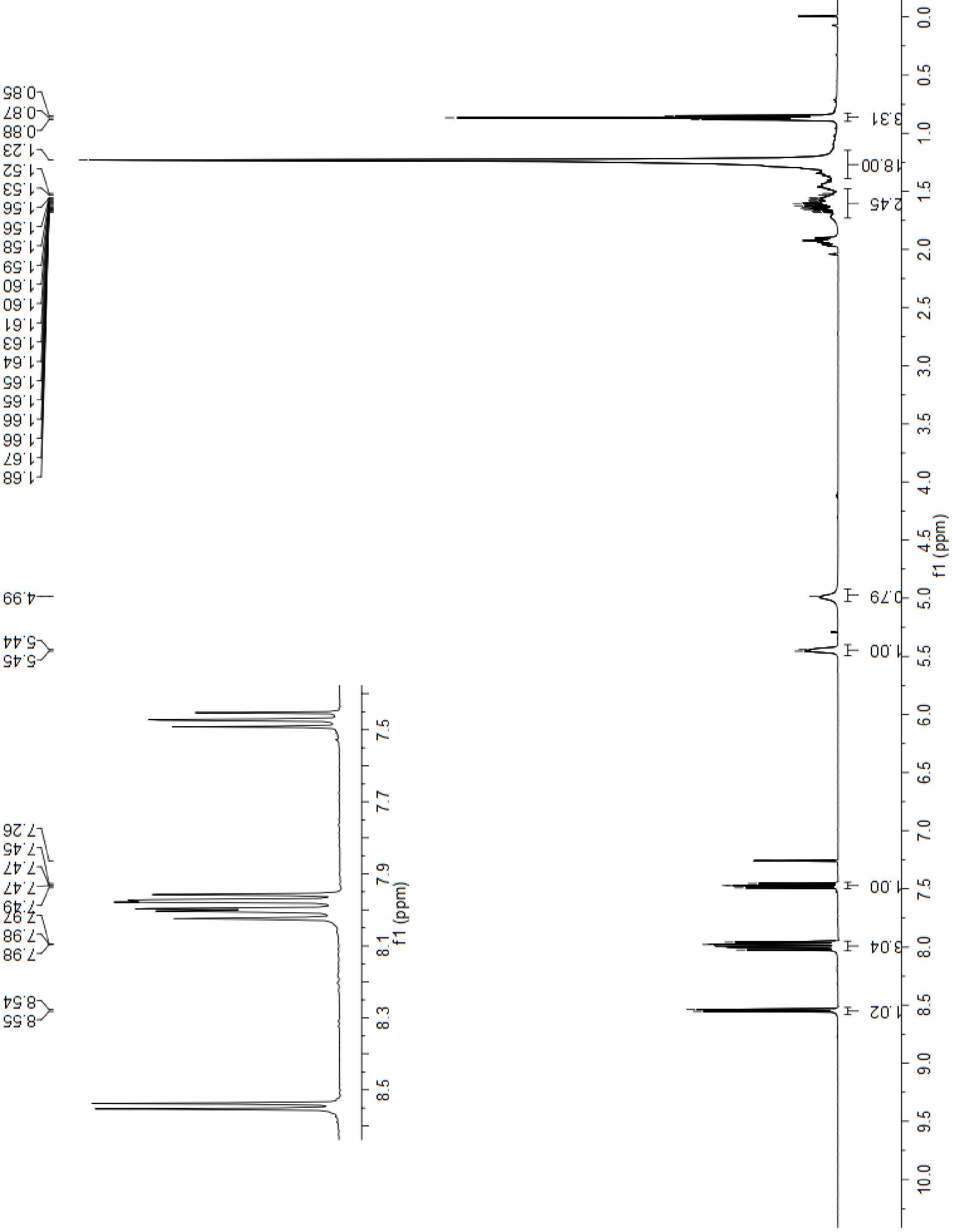

^{13}C NMR of **5t** ($CDCl_3$, 101 MHz, 25 ºC)

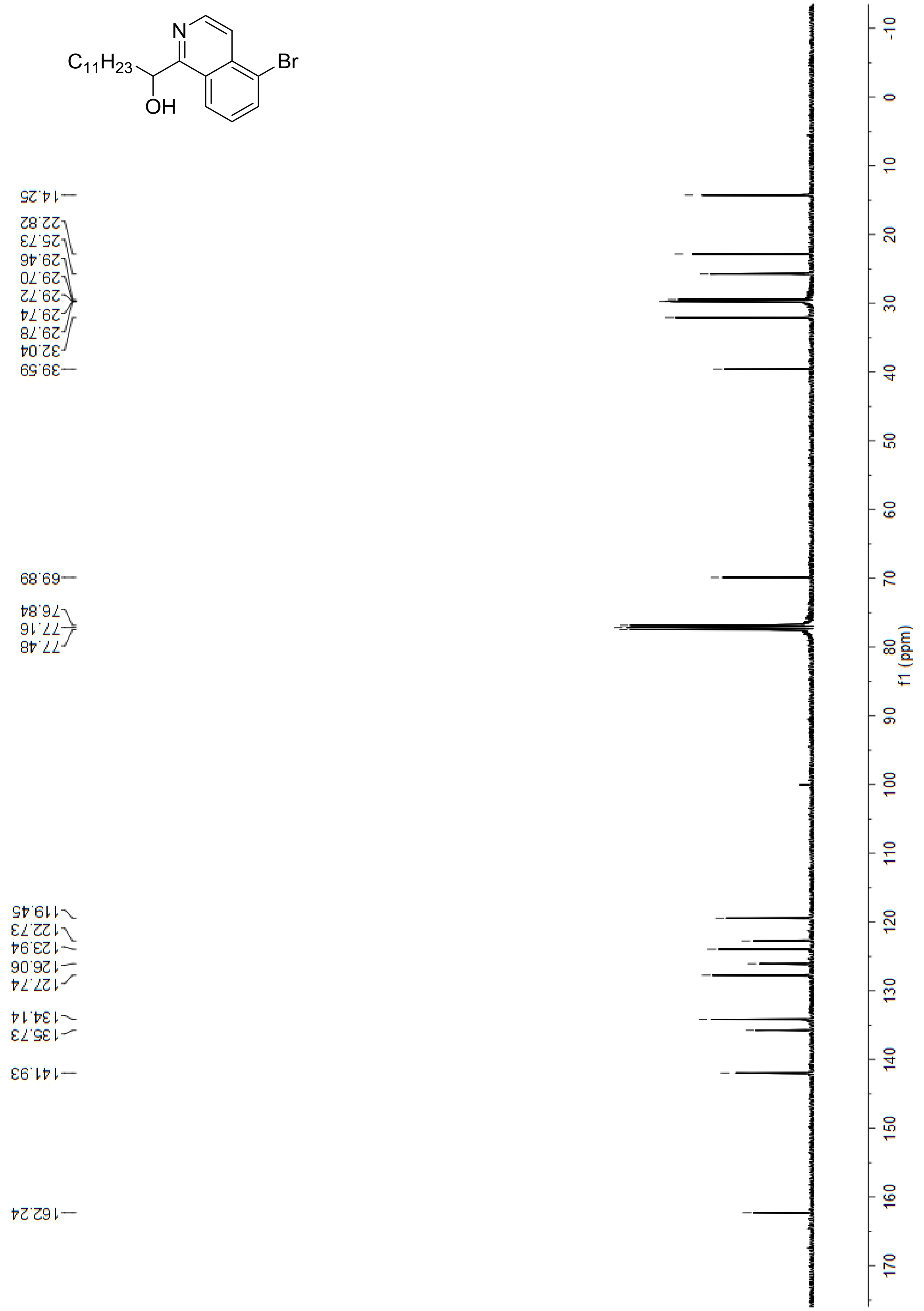

1-(4-bromoisoquinolin-1-yl)heptan-1-ol (5u)

^{1}H NMR of **5u** ($CDCl_3$, 400 MHz, 25 ºC)

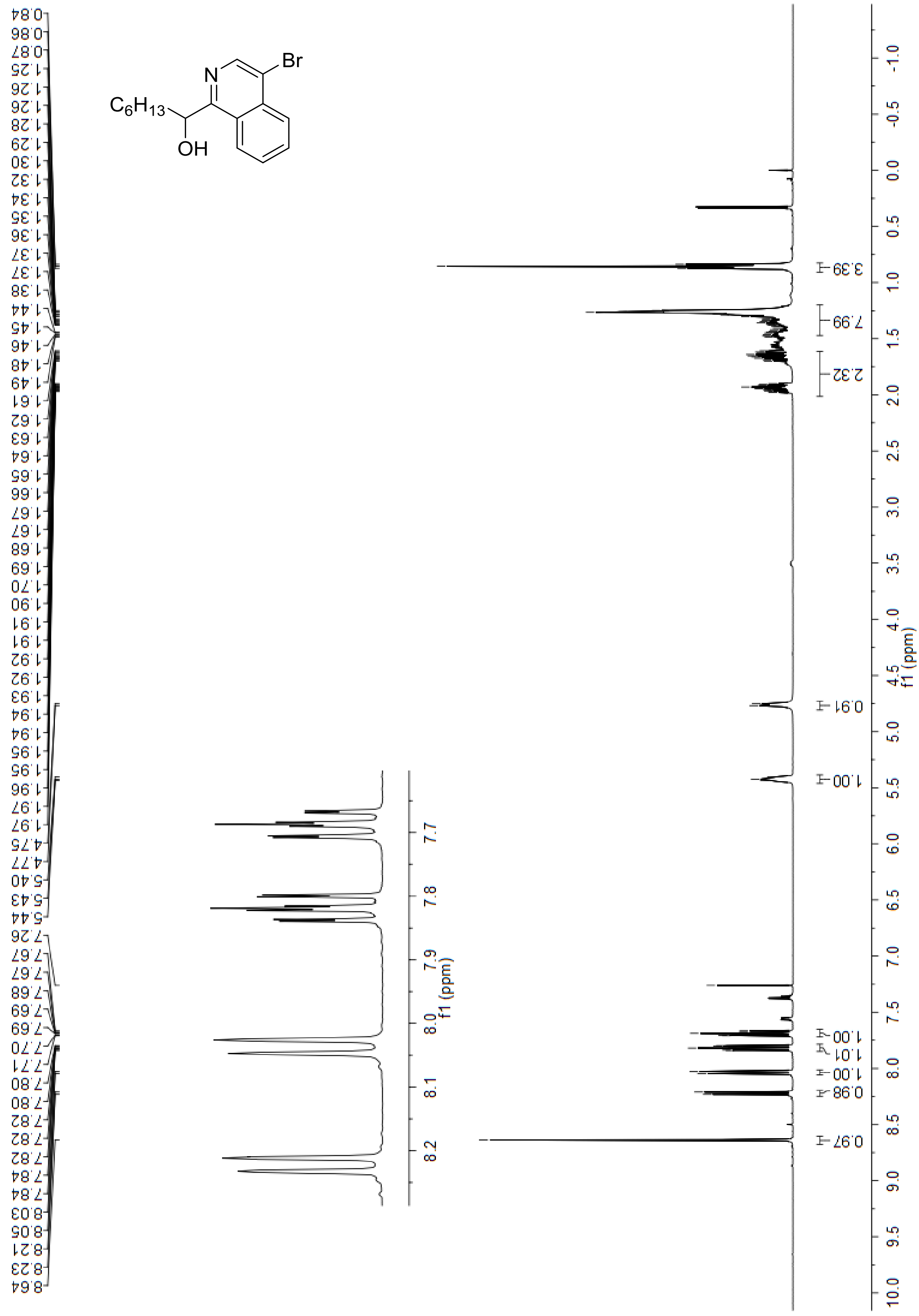

^{13}C NMR of **5u** ($CDCl_3$, 101 MHz, 25 ºC)

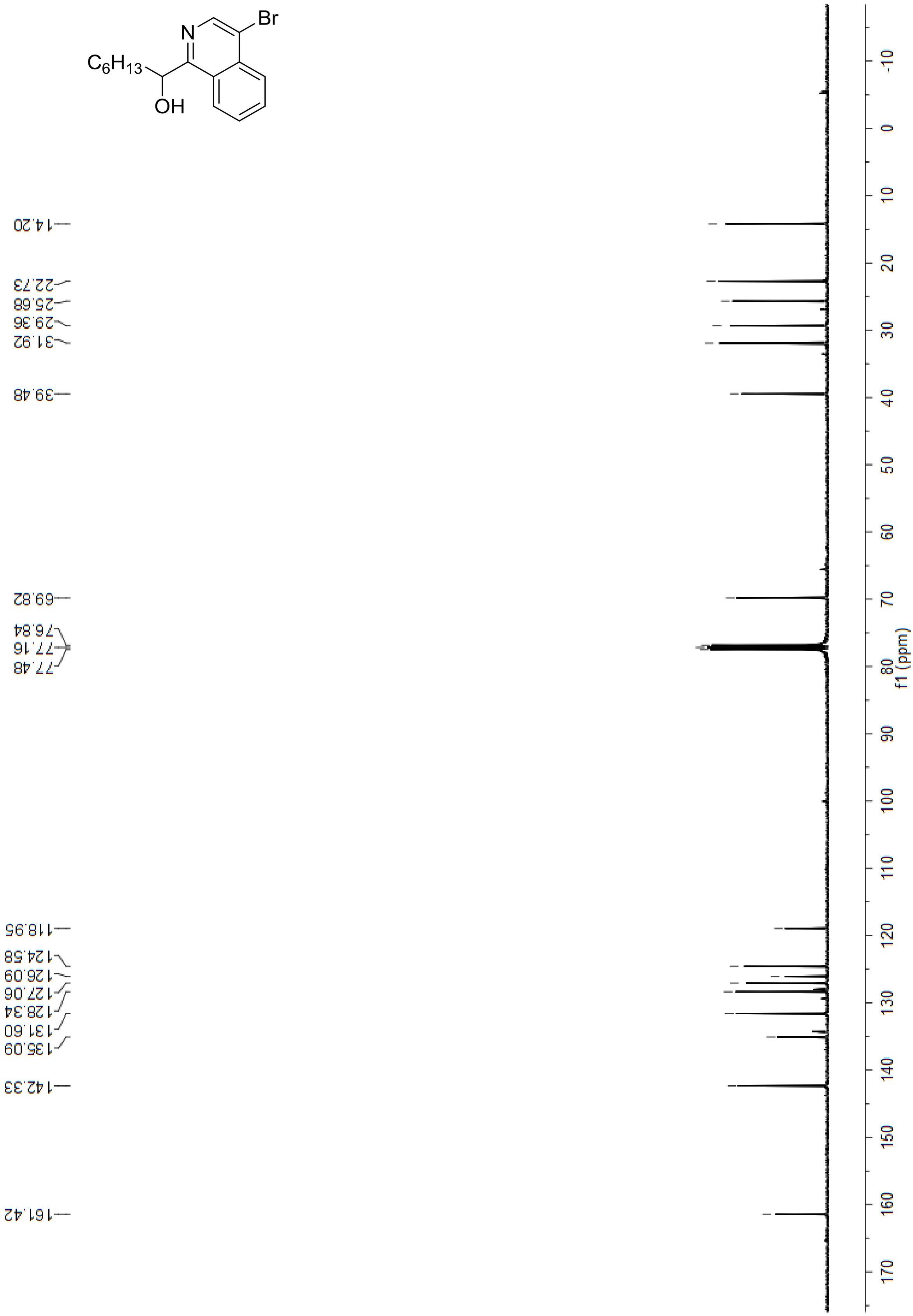

1-(4-bromoisoquinolin-1-yl)-4-((4-fluorobenzyl)oxy)butan-1-ol (5v)

^{1}H NMR of **5v** ($CDCl_3$, 400 MHz, 25 ºC)

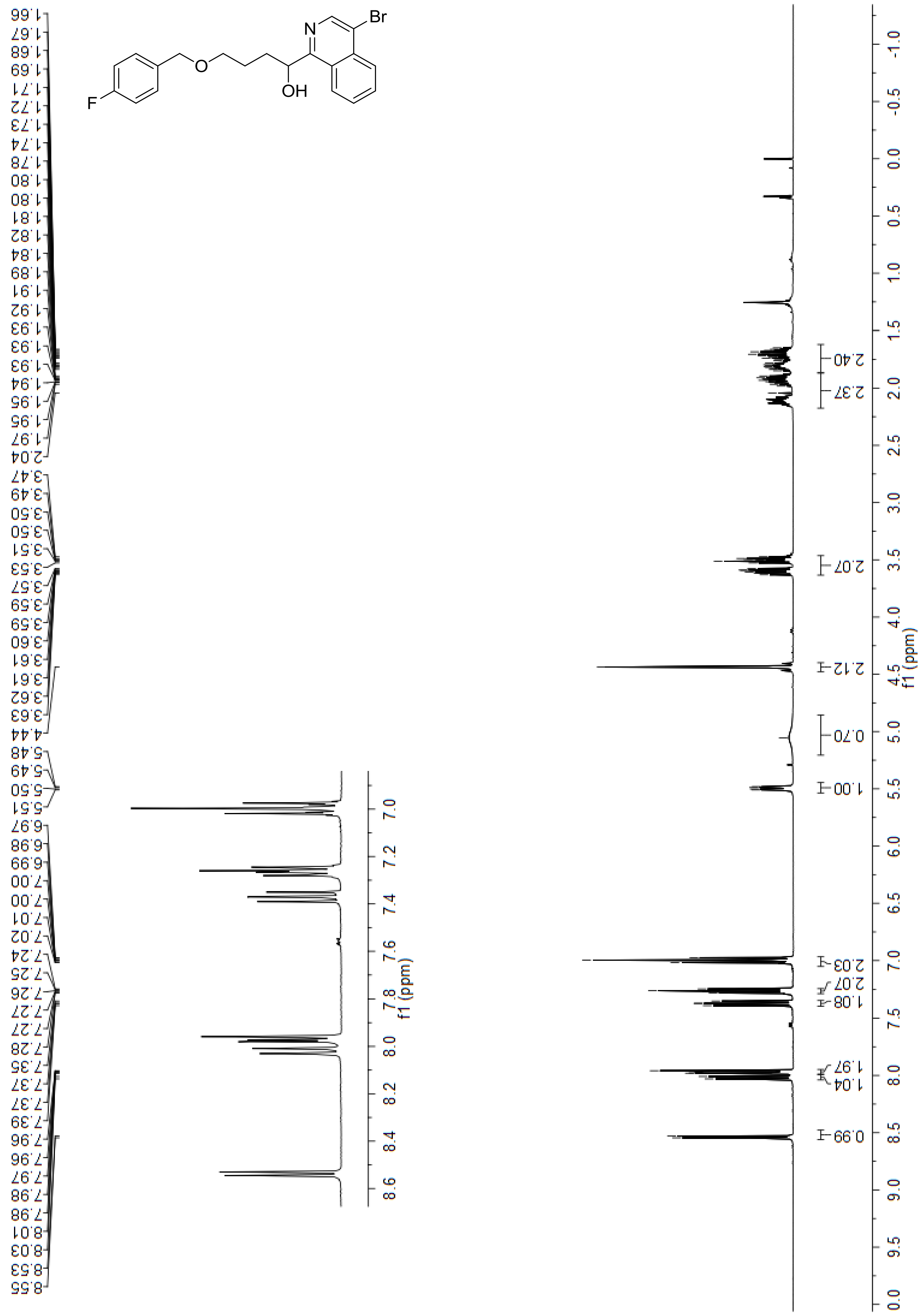

^{13}C NMR of **5v** ($CDCl_3$, 101 MHz, 25 ºC)

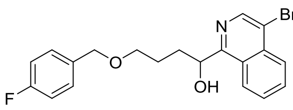

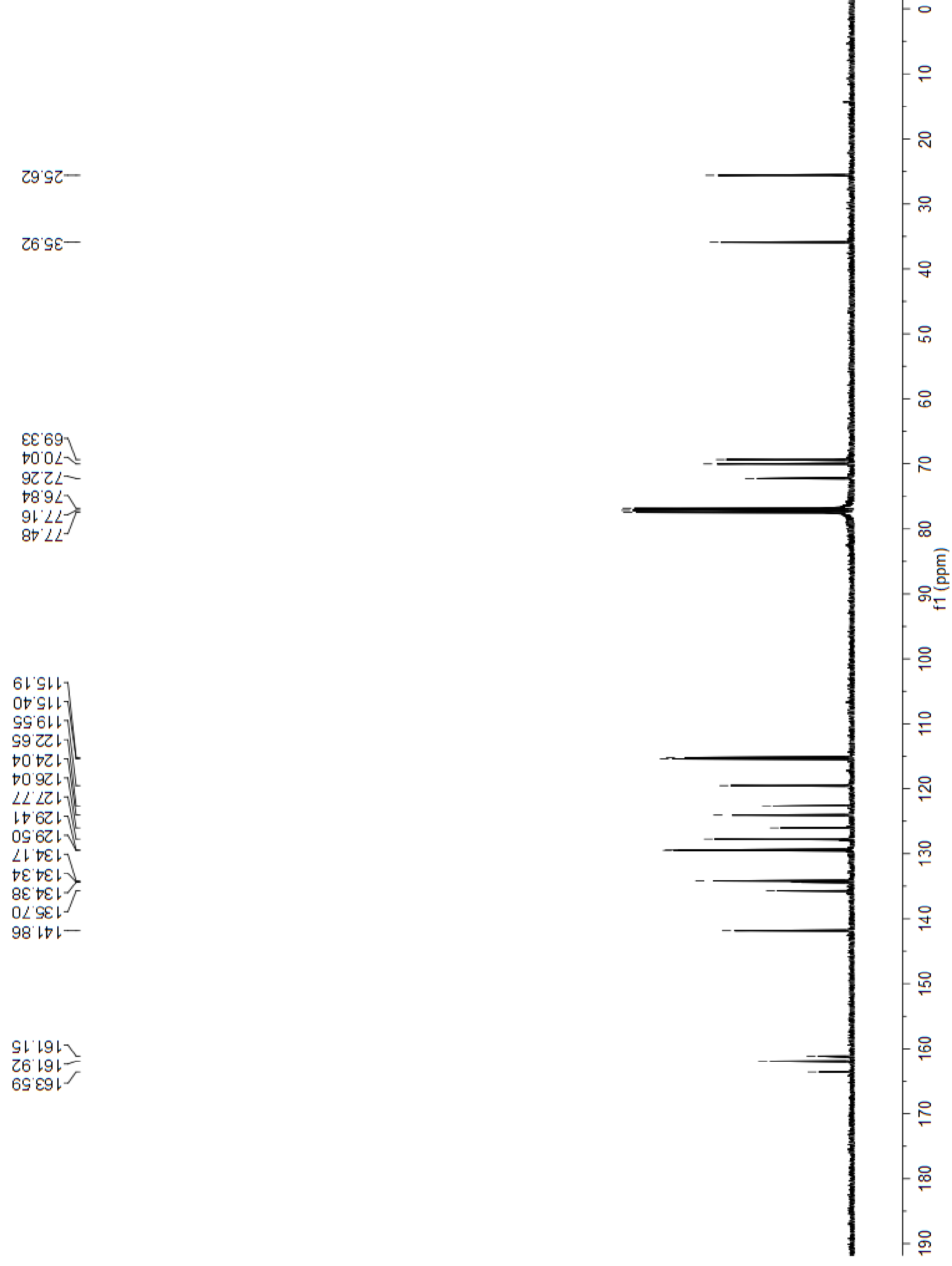

^{19}F NMR of **5v** ($CDCl_3$, 375 MHz, 25 ºC)

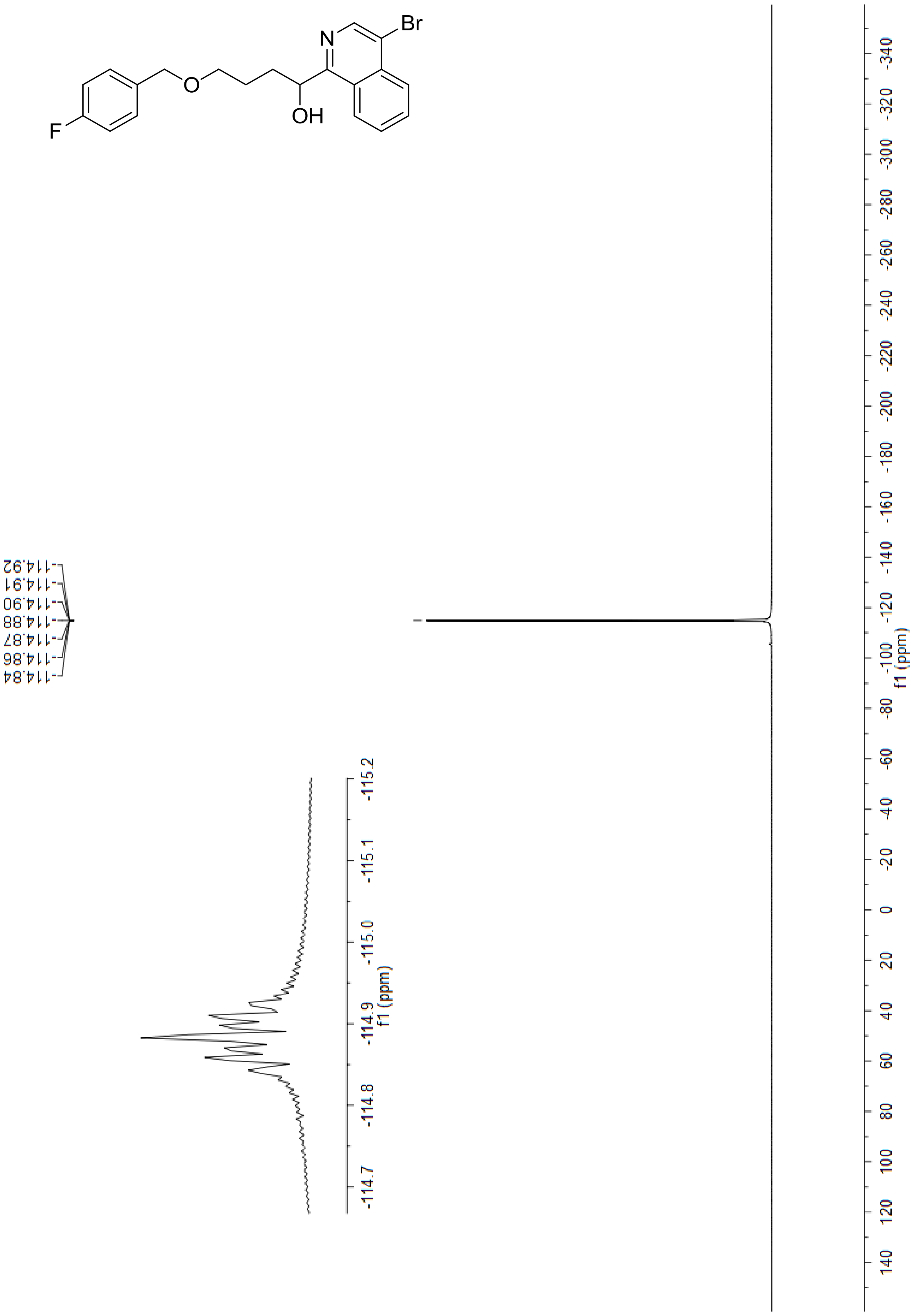

4-((3-chlorobenzyl)oxy)-1-(5-(4-methoxyphenyl)quinolin-2-yl)butan-1-ol (5w)

^{1}H NMR of **5w** ($CDCl_3$, 400 MHz, 25 ºC)

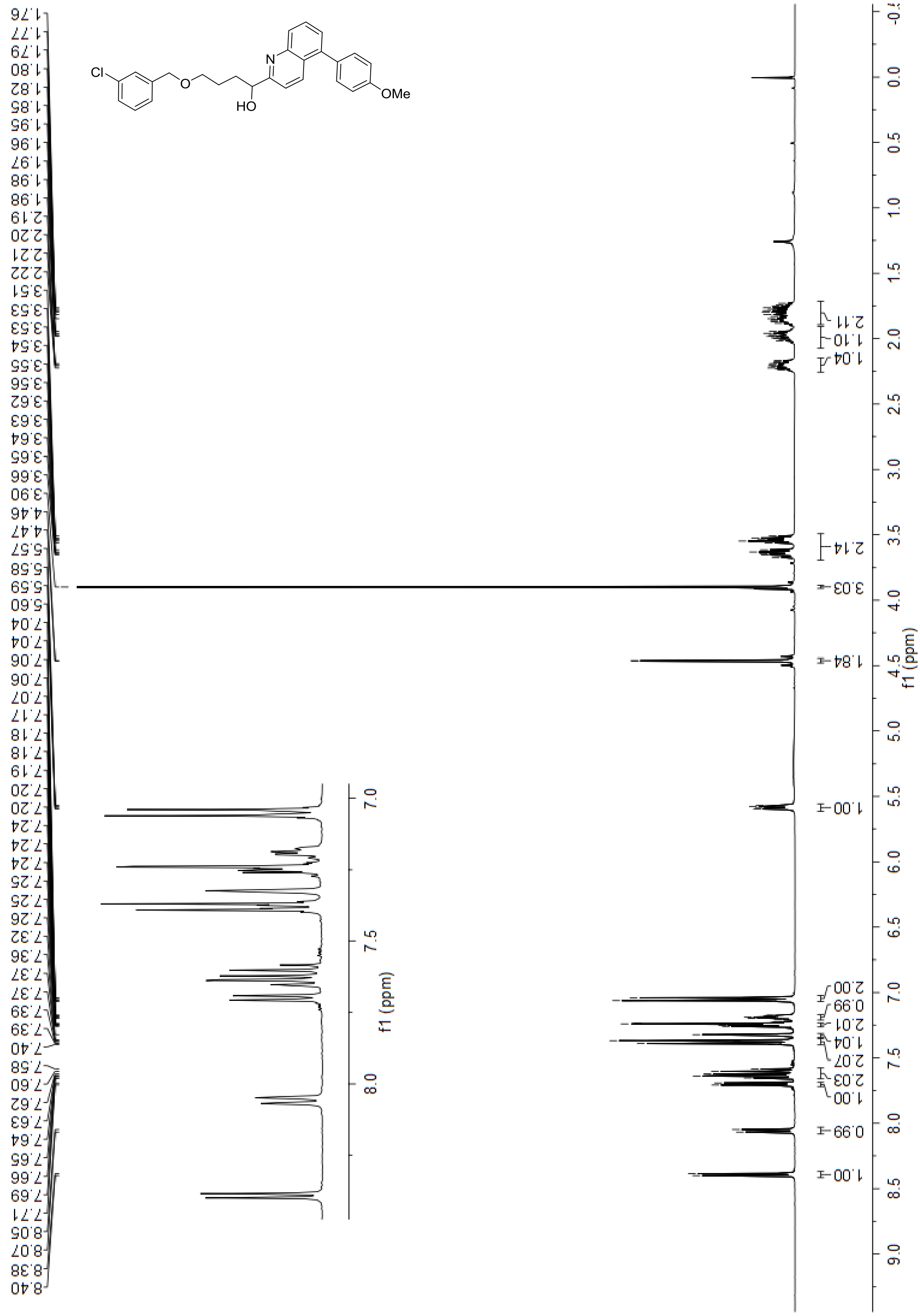

^{13}C NMR of **5w** ($CDCl_3$, 101 MHz, 25 ºC)

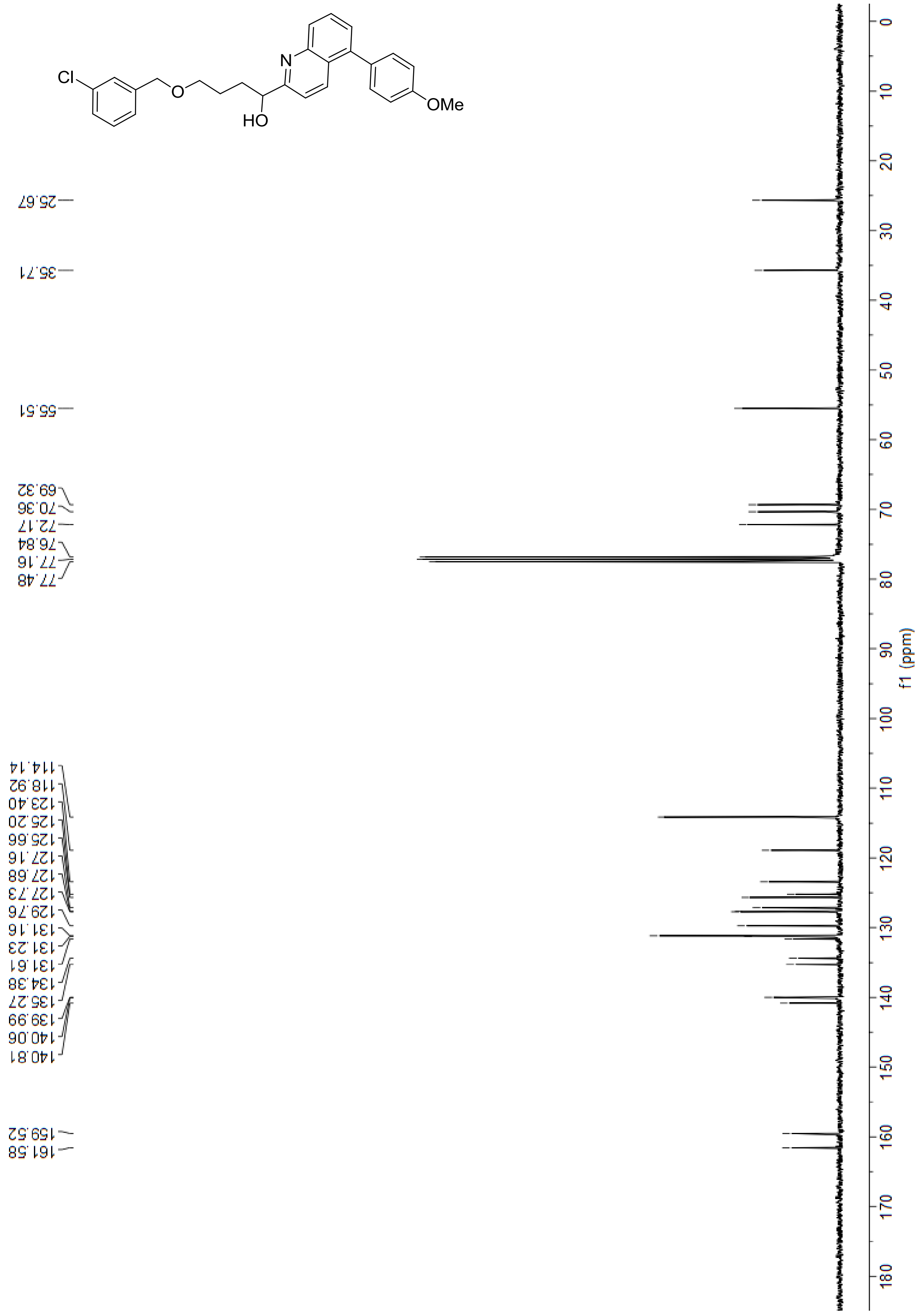

1-(5-bromoisoquinolin-1-yl)-4-(3-phenylpropoxy)butan-1-ol (5x)

^{1}H NMR of **5x** ($CDCl_3$, 400 MHz, 25 ºC)

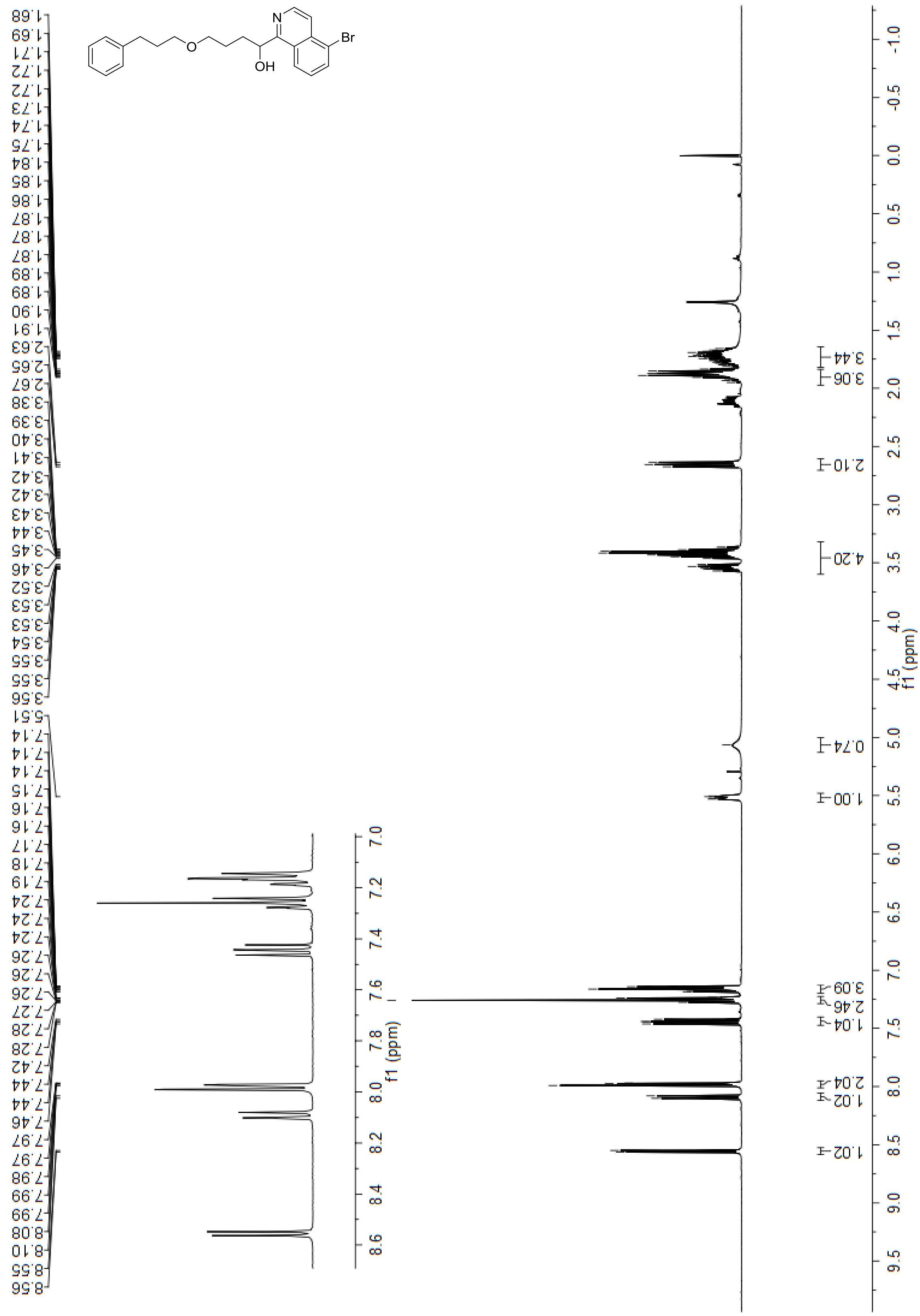

^{13}C NMR of **5x** ($CDCl_3$, 101 MHz, 25 ºC)

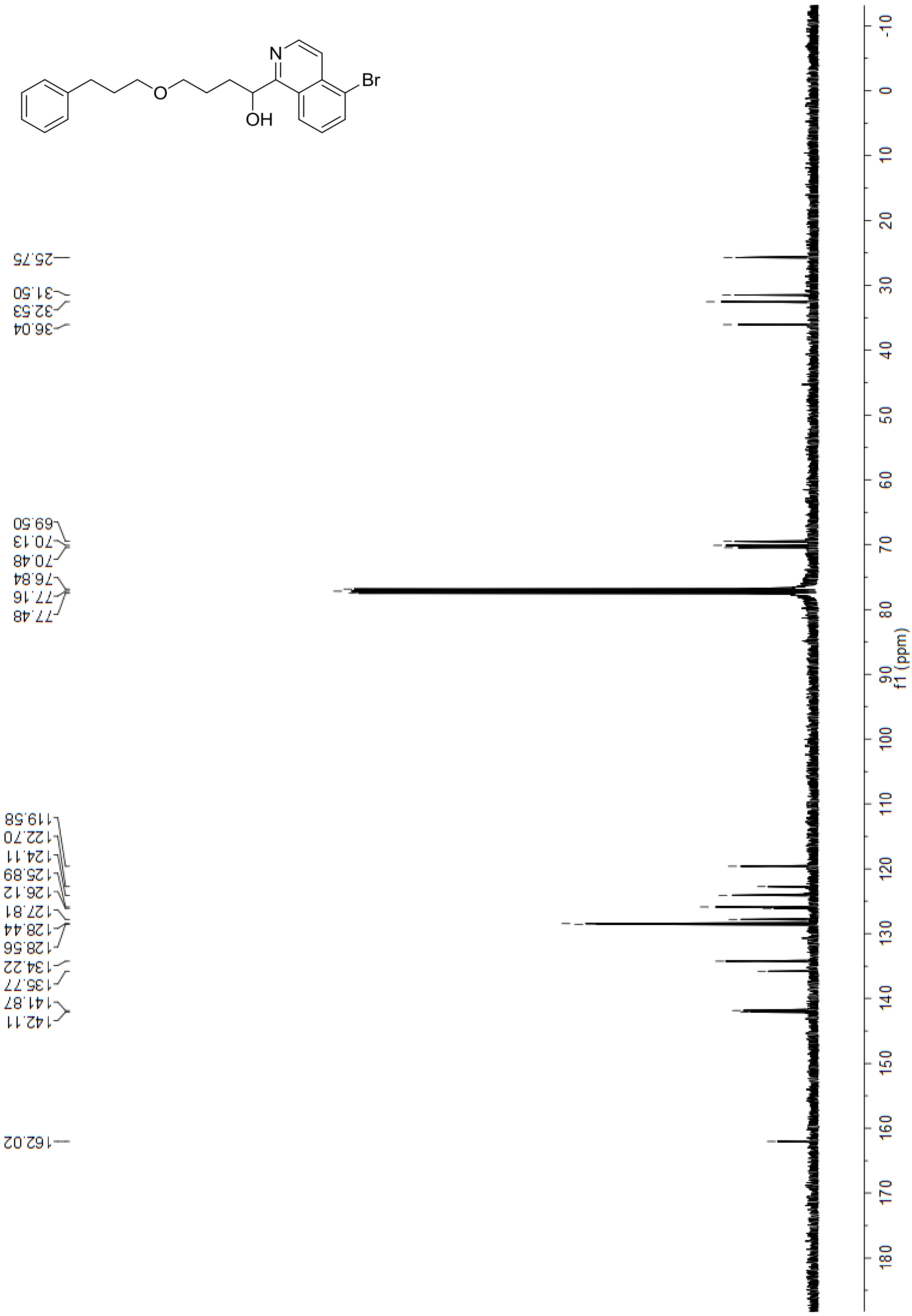

1-(5-bromoisoquinolin-1-yl)-6-((4-(trifluoromethoxy)benzyl)oxy)hexan-1-ol (5y)

^{1}H NMR of **5y** ($CDCl_3$, 400 MHz, 25 ºC)

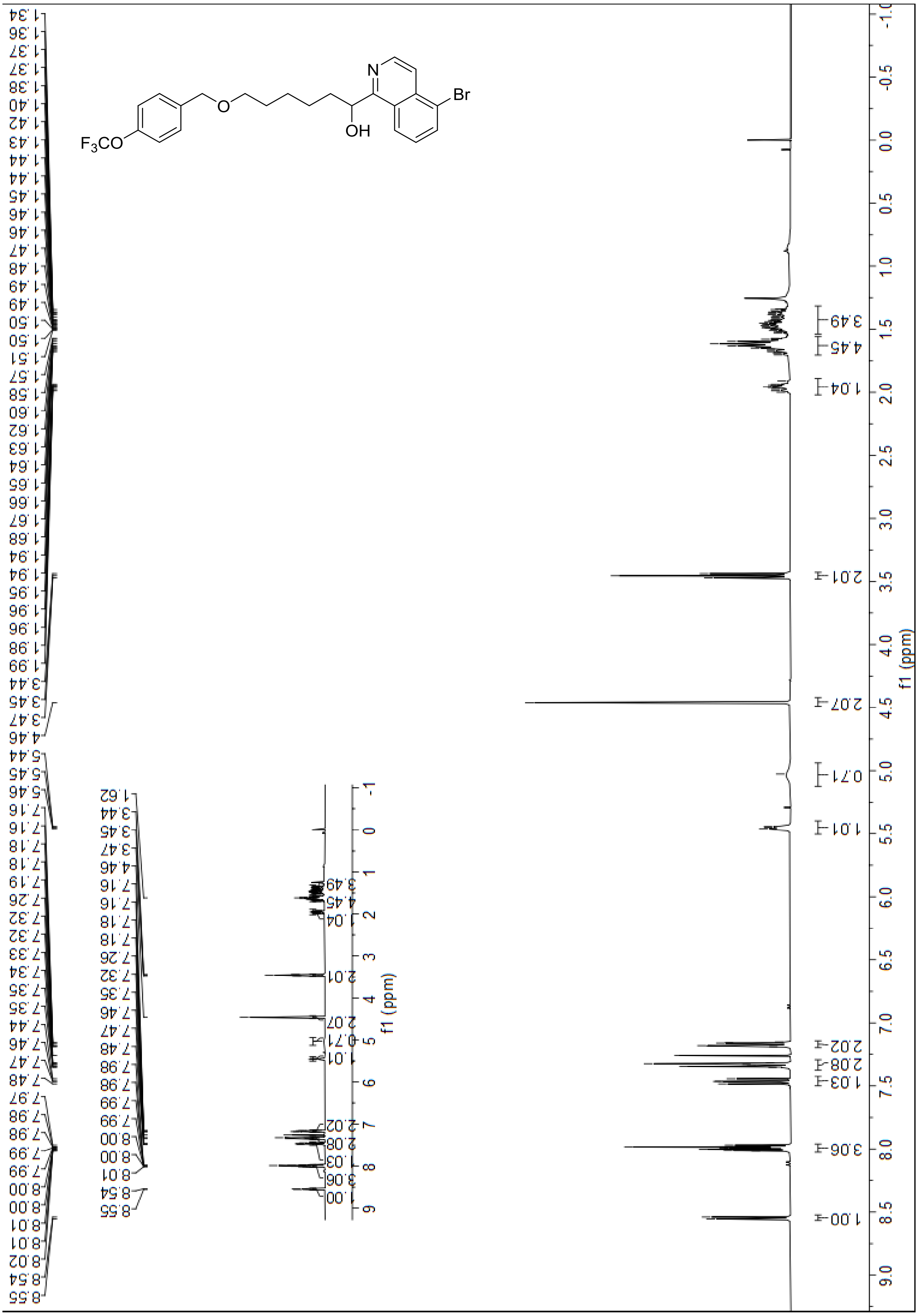

^{13}C NMR of **5y** ($CDCl_3$, 101 MHz, 25 ºC)

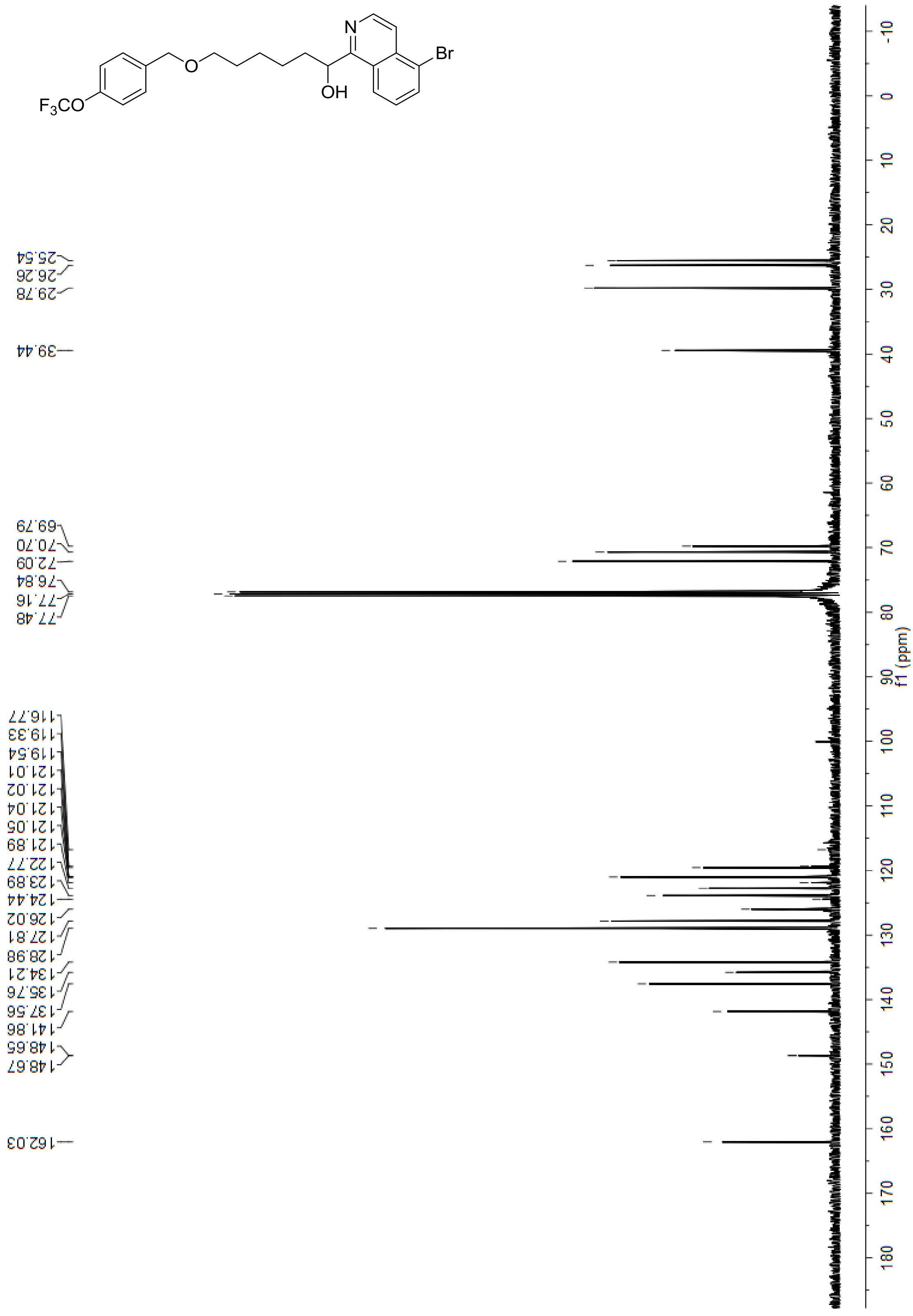

^{19}F NMR of **5y** ($CDCl_3$, 375 MHz, 25 ºC)

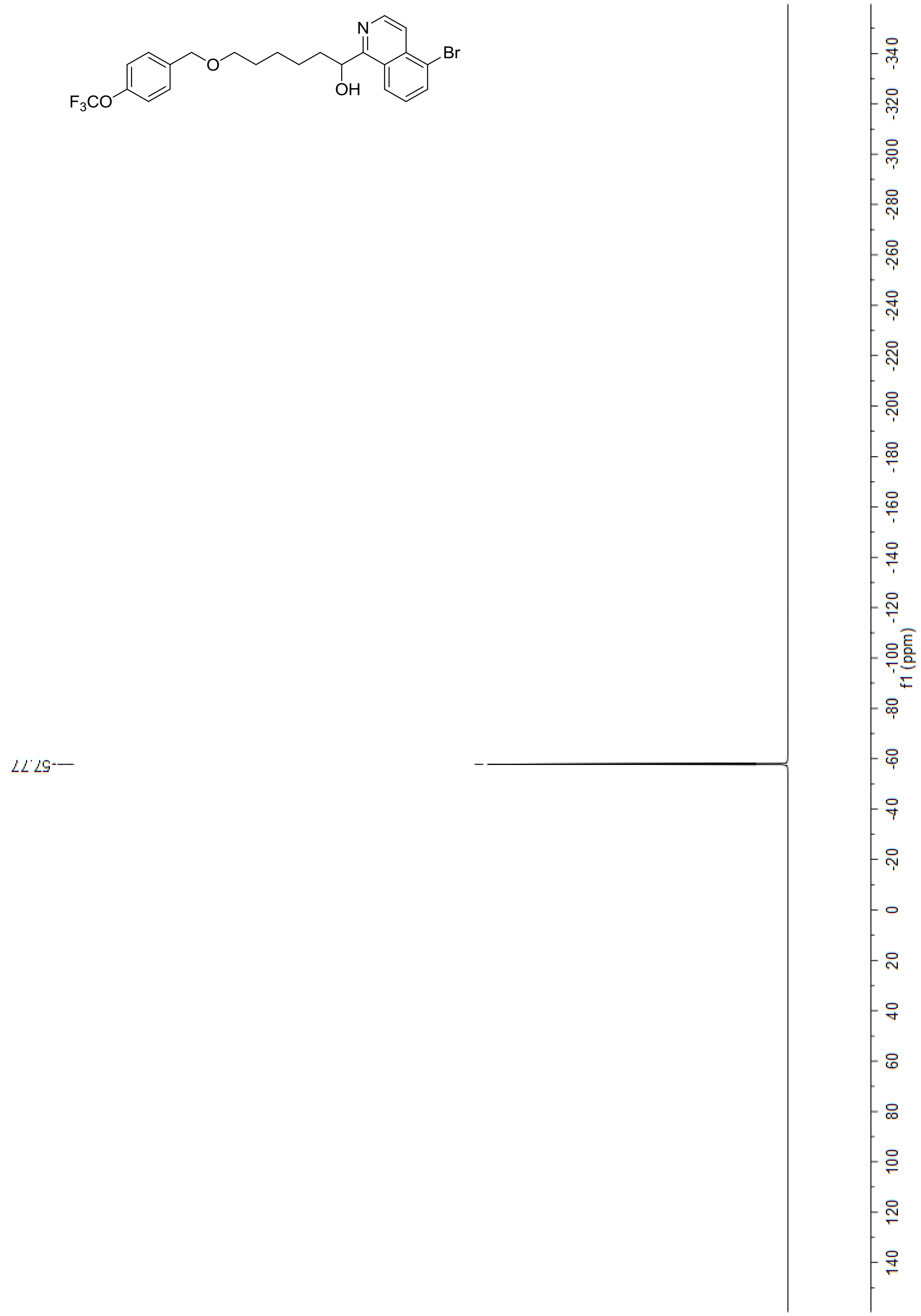

6-(benzyloxy)-1-(2-(4-chlorophenyl)quinolin-4-yl)hexan-1-ol (5z)

^{1}H NMR of **5z** ($CDCl_3$, 400 MHz, 25 ºC)

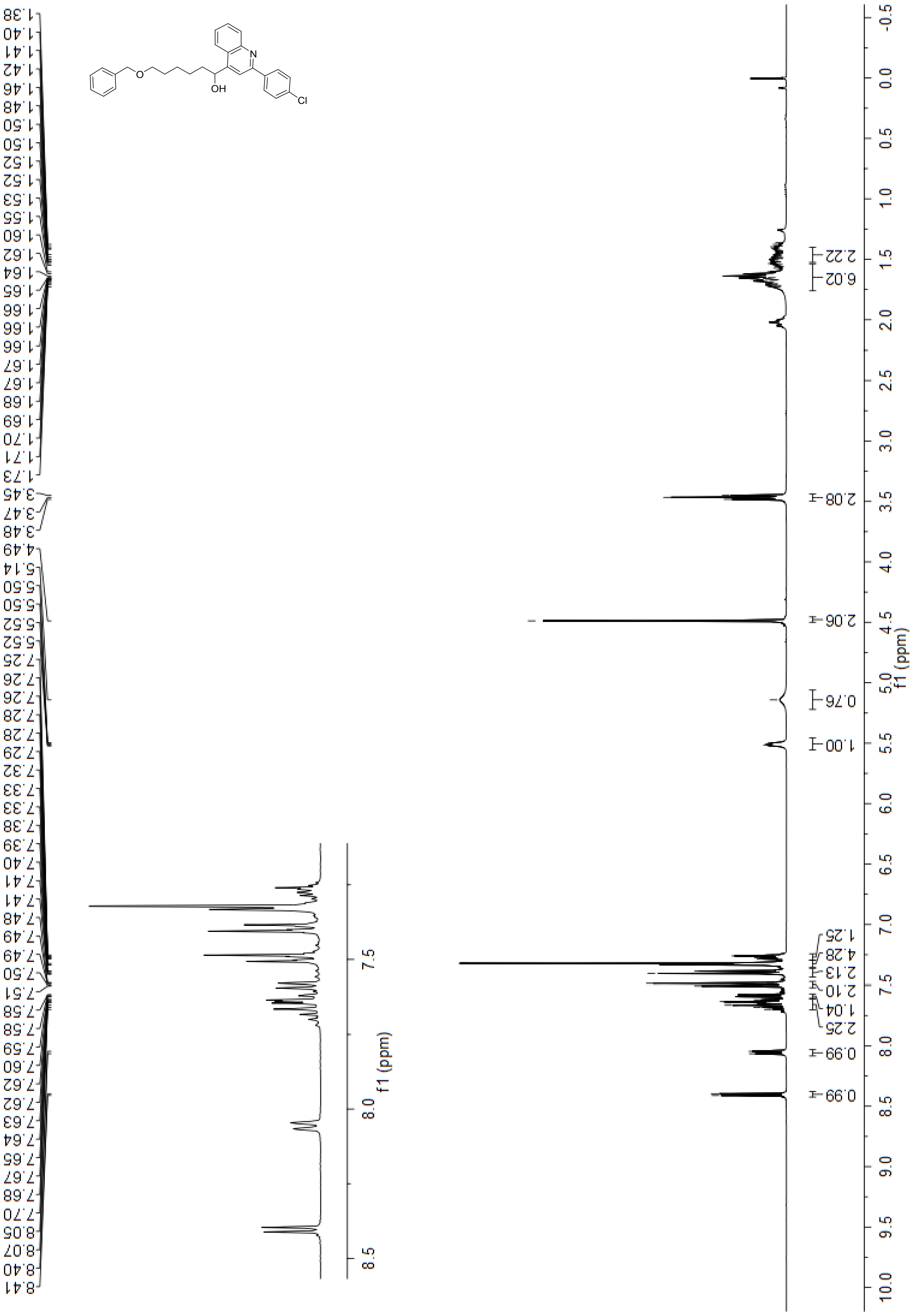

^{13}C NMR of **5z** ($CDCl_3$, 101 MHz, 25 ºC)

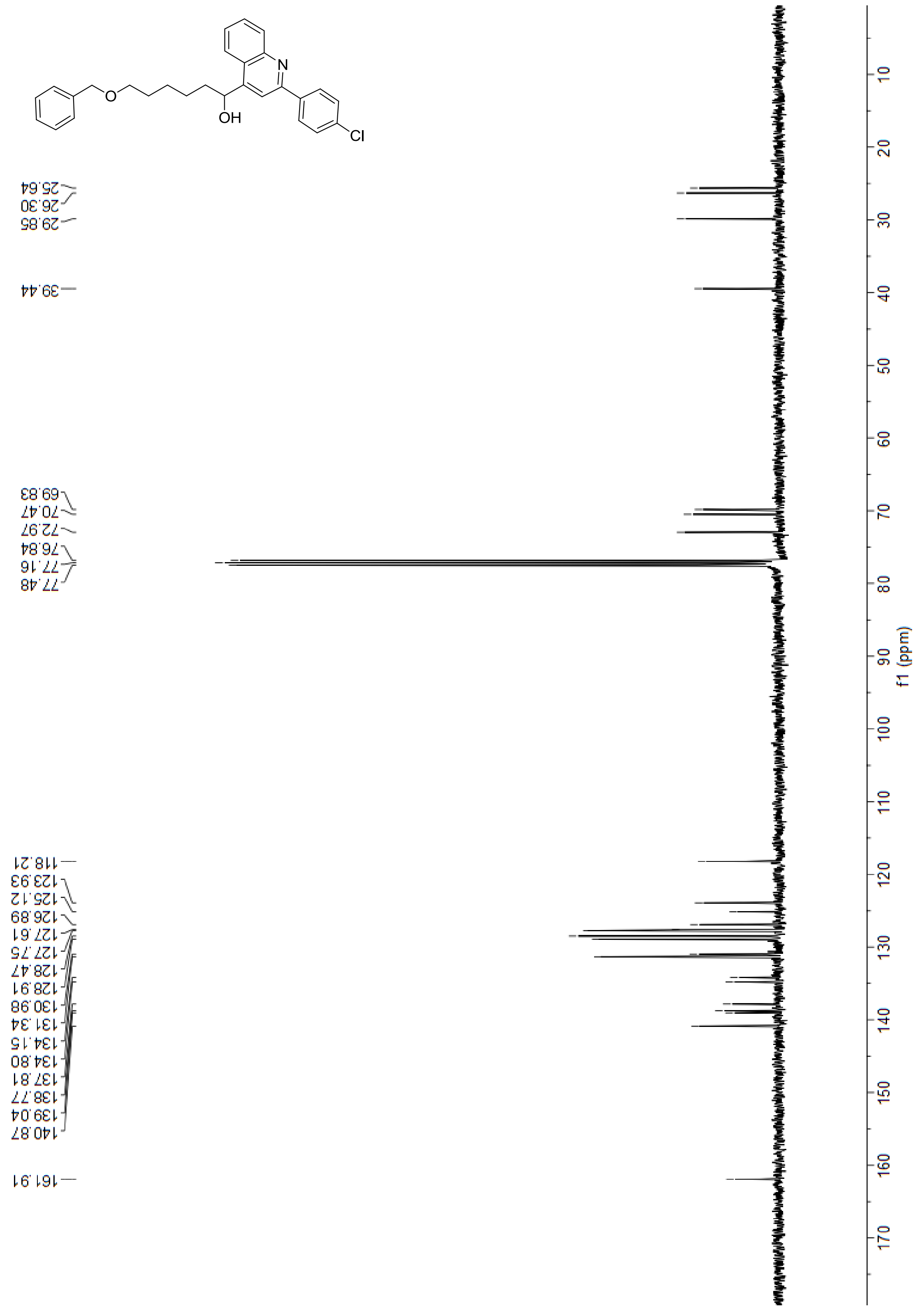

1-(2-(4-(hydroxymethyl)phenyl)quinolin-4-yl)heptan-1-ol (5aa)

^{1}H NMR of **5aa** ($CDCl_3$, 400 MHz, 25 ºC)

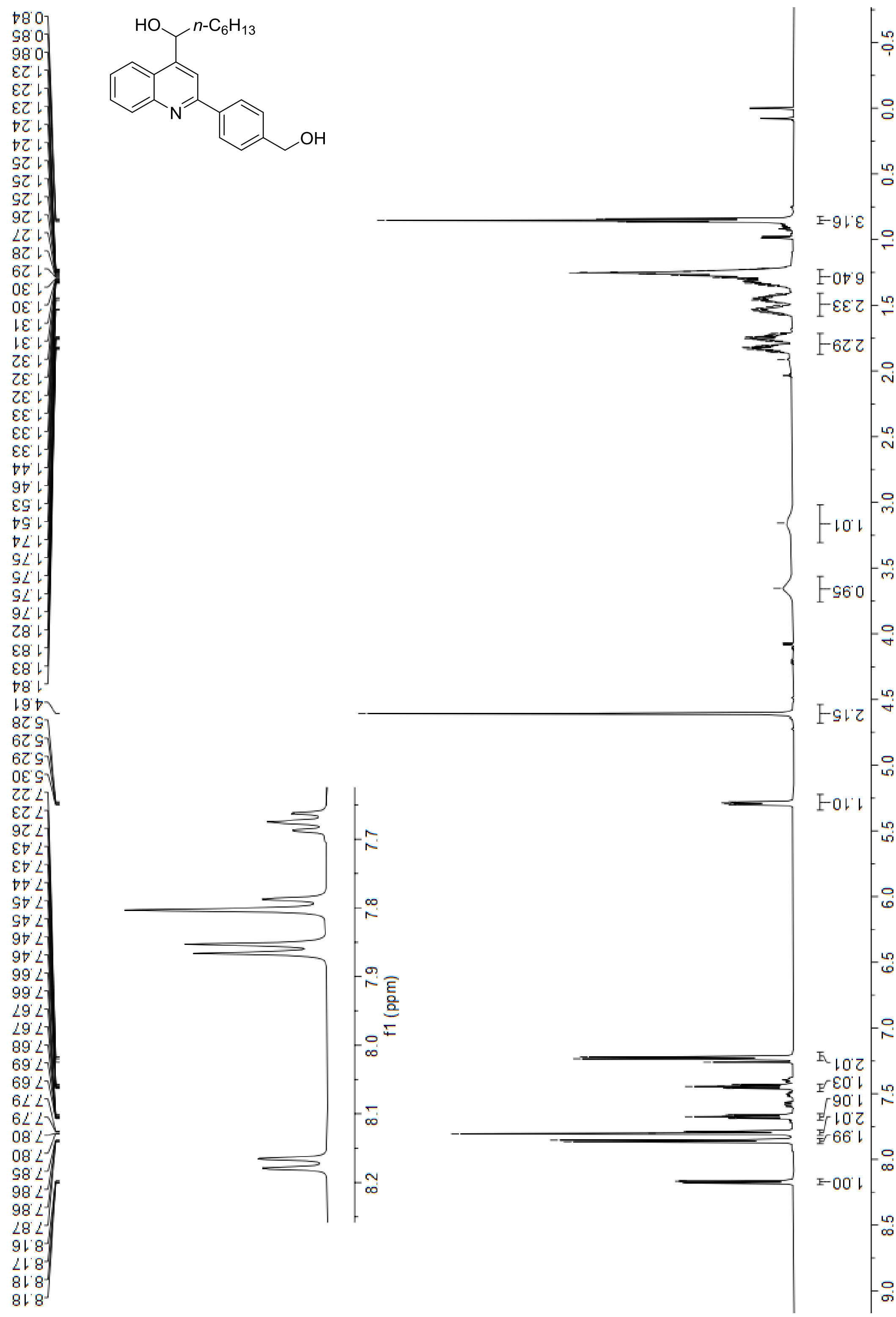

^{13}C NMR of **5aa** ($CDCl_3$, 101 MHz, 25 ºC)

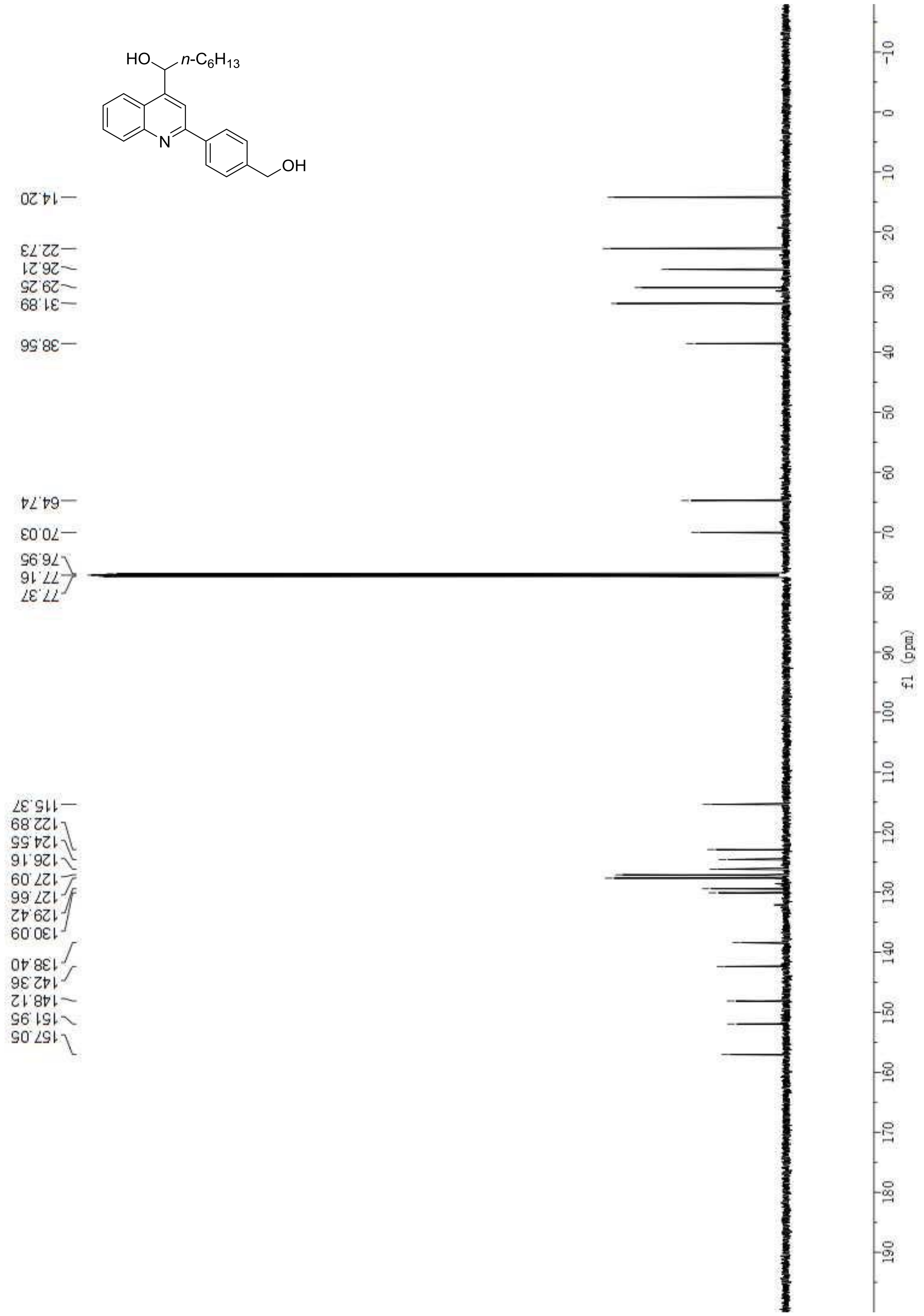

4-((3-chlorobenzyl)oxy)-1-(2-(4-(hydroxymethyl)phenyl)quinolin-4-yl)butan-1-ol (5ab)

^{1}H NMR of **5ab** ($CDCl_3$, 600 MHz, 25 ºC)

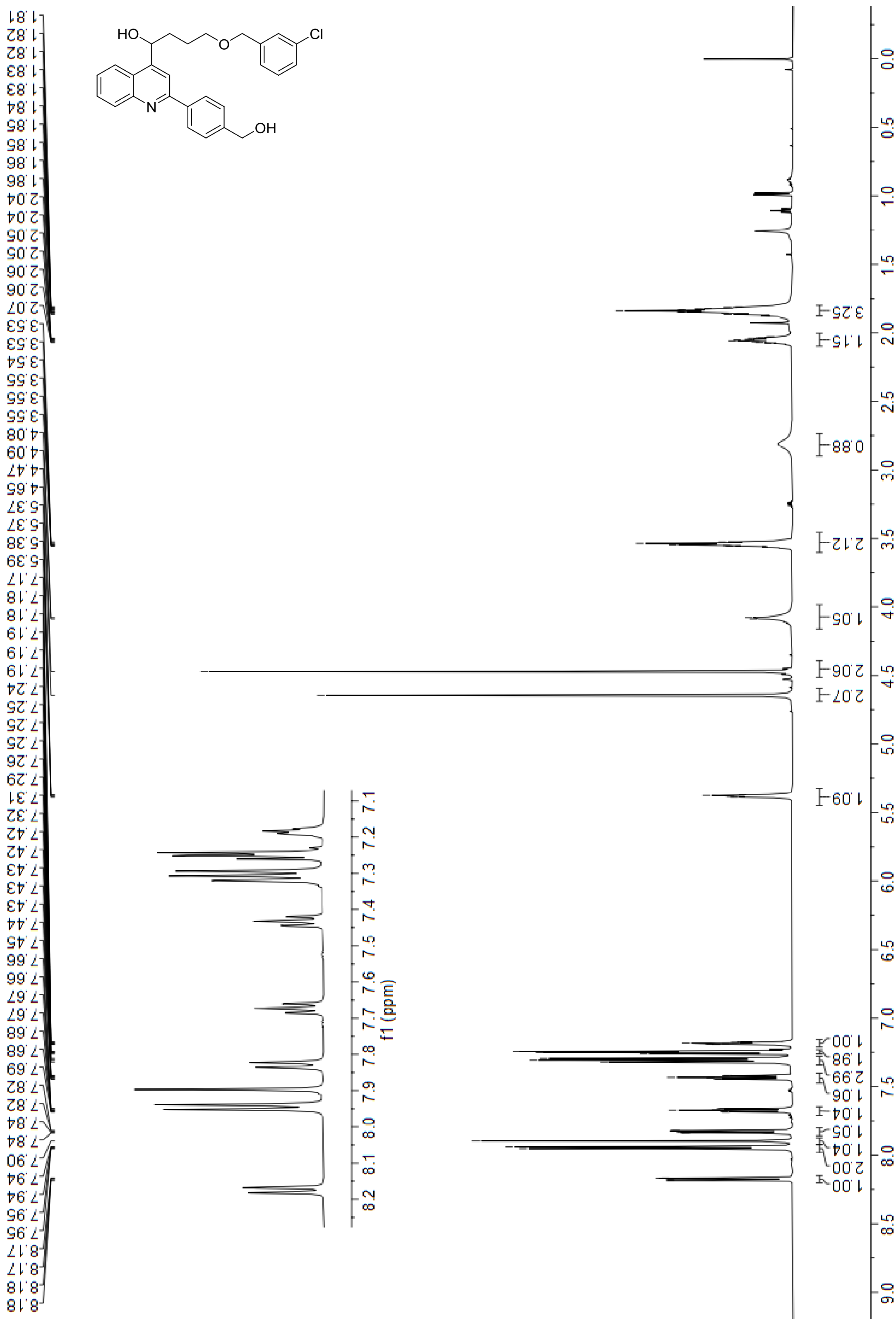

^{13}C NMR of **5ab** ($CDCl_3$, 151 MHz, 25 ºC)

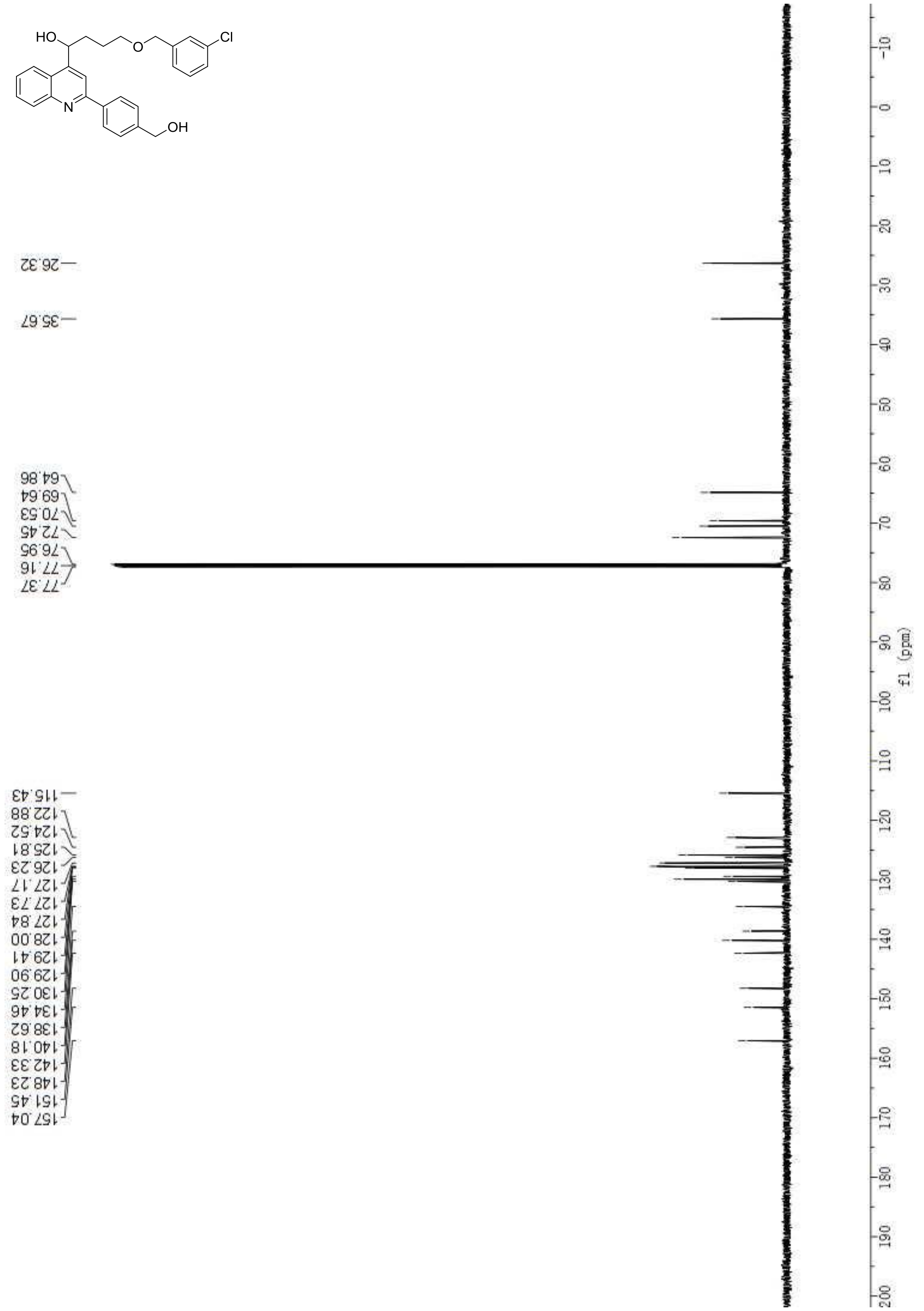

1-(5-bromoisoquinolin-1-yl)-2-methylbutan-1-ol (5ac-Isomer I)

^{1}H NMR of **5ac-Isomer I** ($CDCl_3$, 600 MHz, 25 ºC)

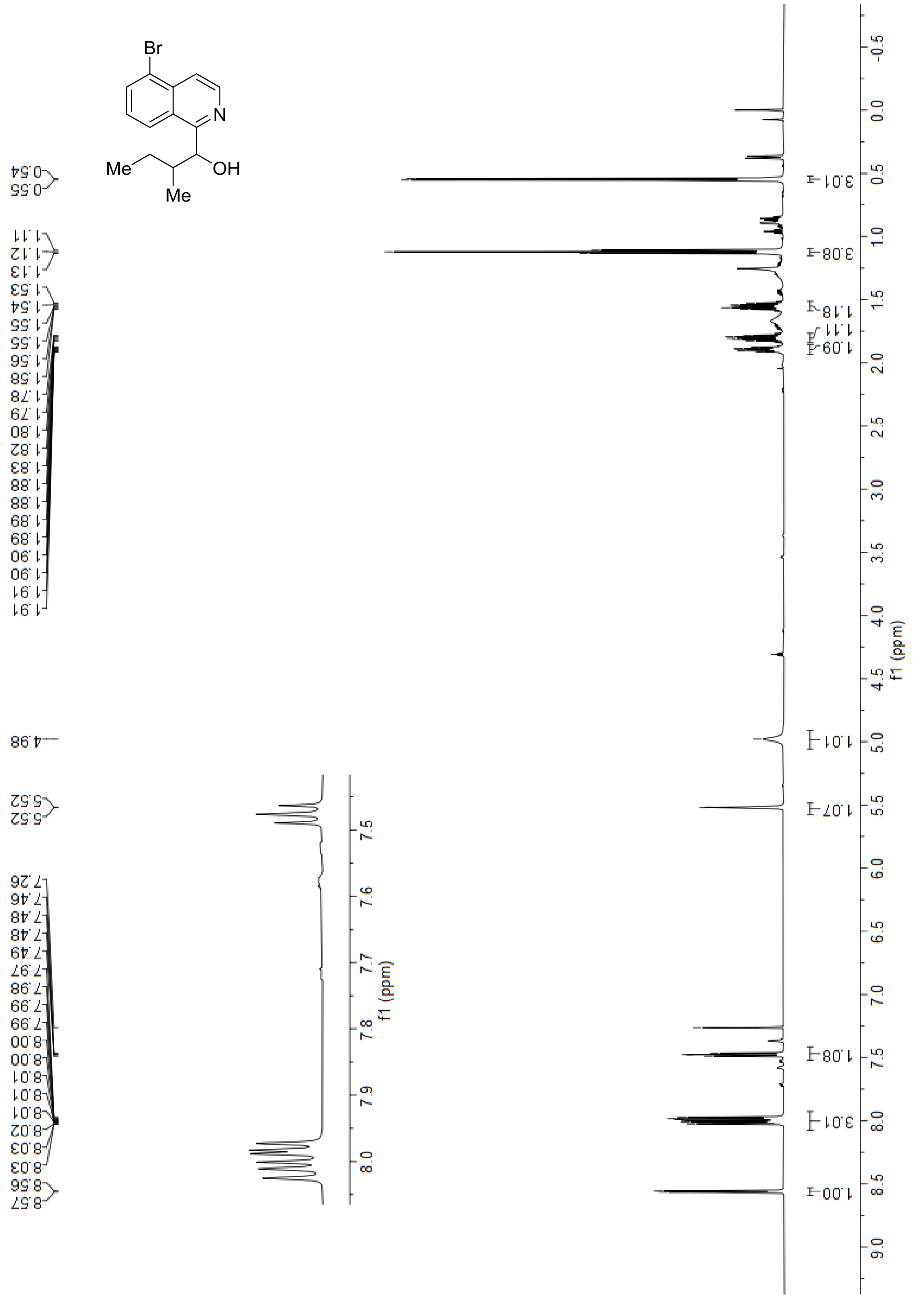

^{13}C NMR of **5ac-Isomer I** ($CDCl_3$, 151 MHz, 25 ºC)

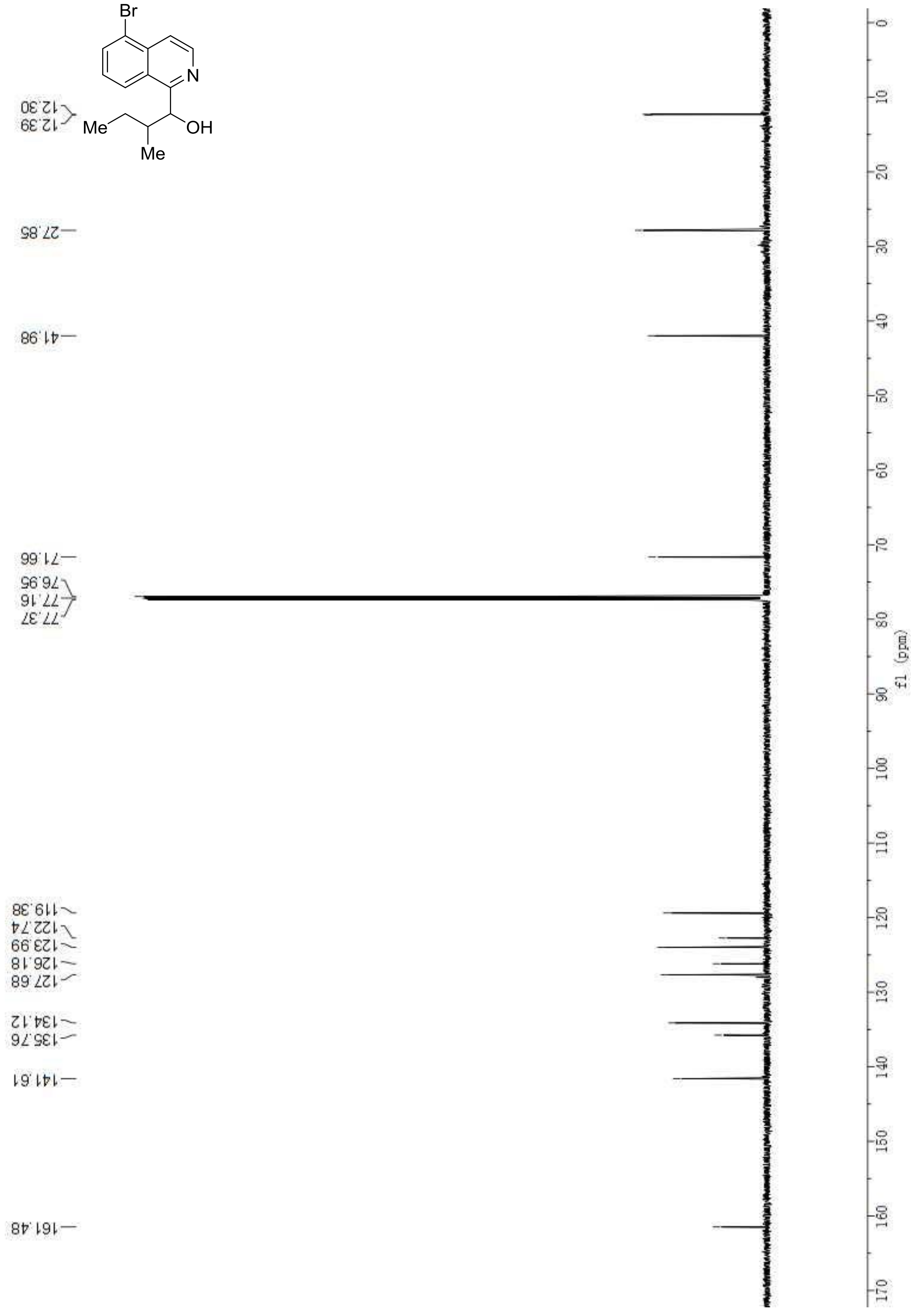

1-(5-bromoisoquinolin-1-yl)-2-methylbutan-1-ol (5ac-Isomer II)

^{1}H NMR of **5ac-Isomer II** ($CDCl_3$, 600 MHz, 25 ºC)

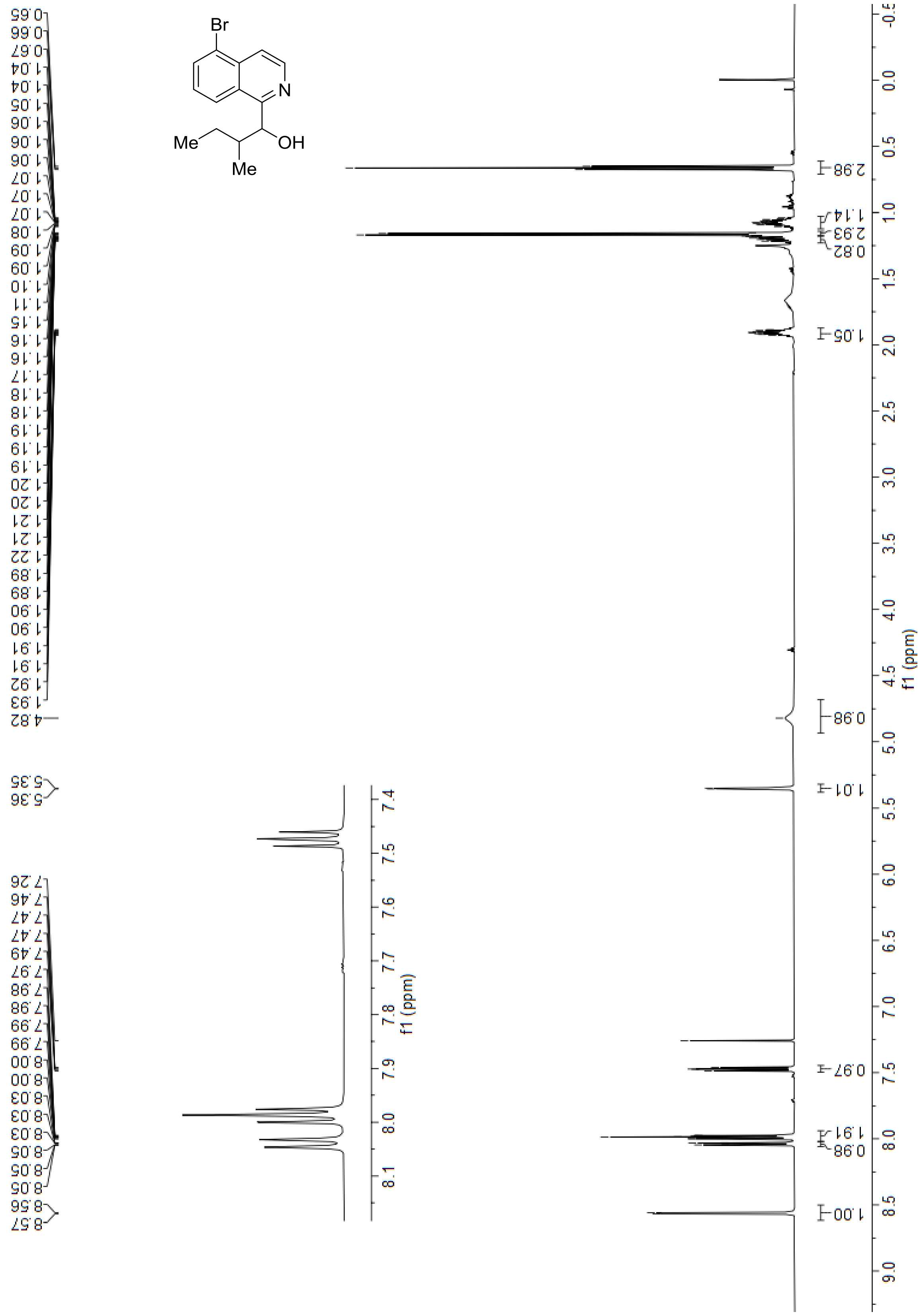

^{13}C NMR of **5ac-Isomer II** ($CDCl_3$, 151 MHz, 25 ºC)

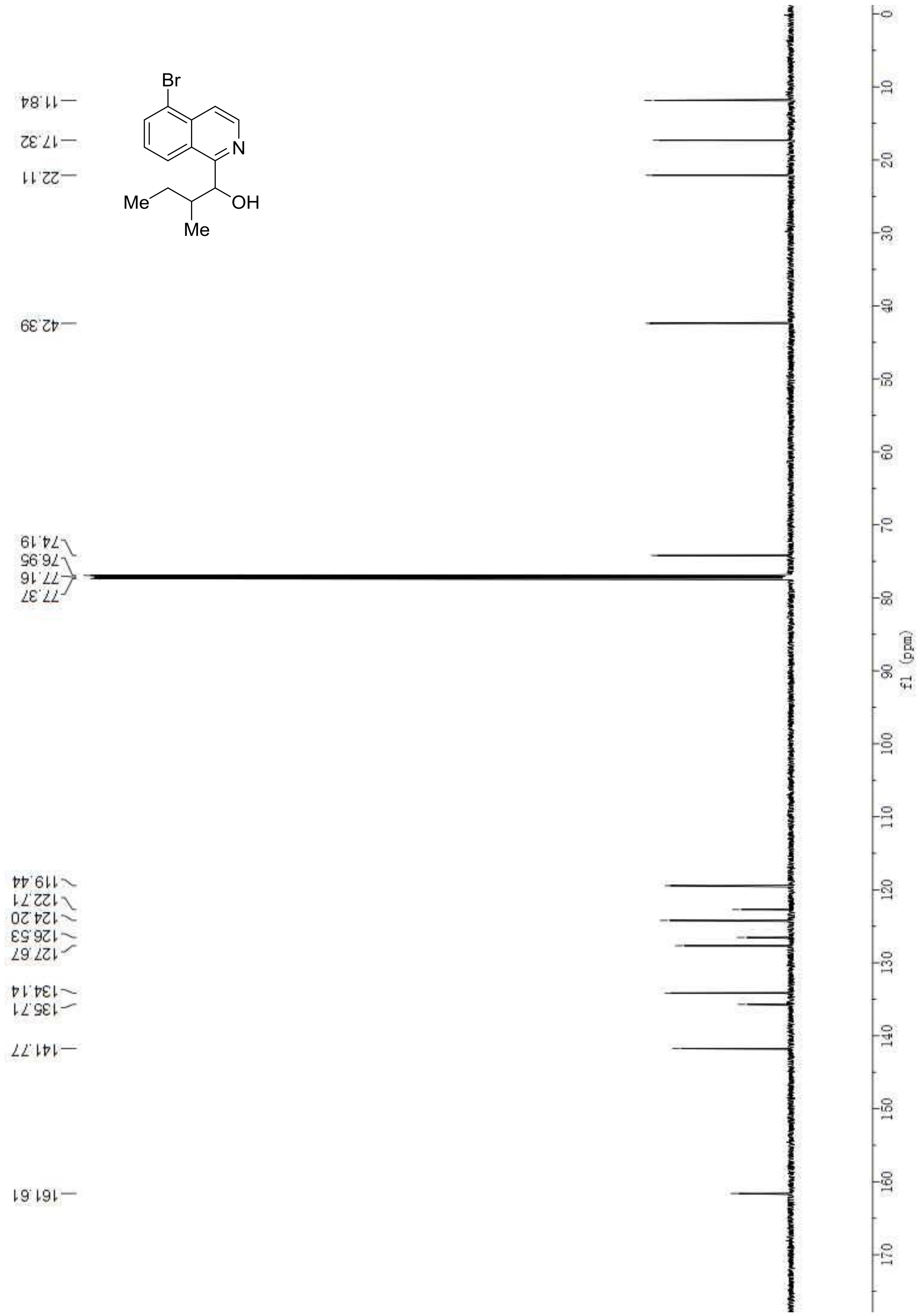

1-(5,6-diphenylpyrazin-2-yl)heptan-1-ol(5ad)

^{1}H NMR of **5ad** ($CDCl_3$, 400 MHz, 25 ºC)

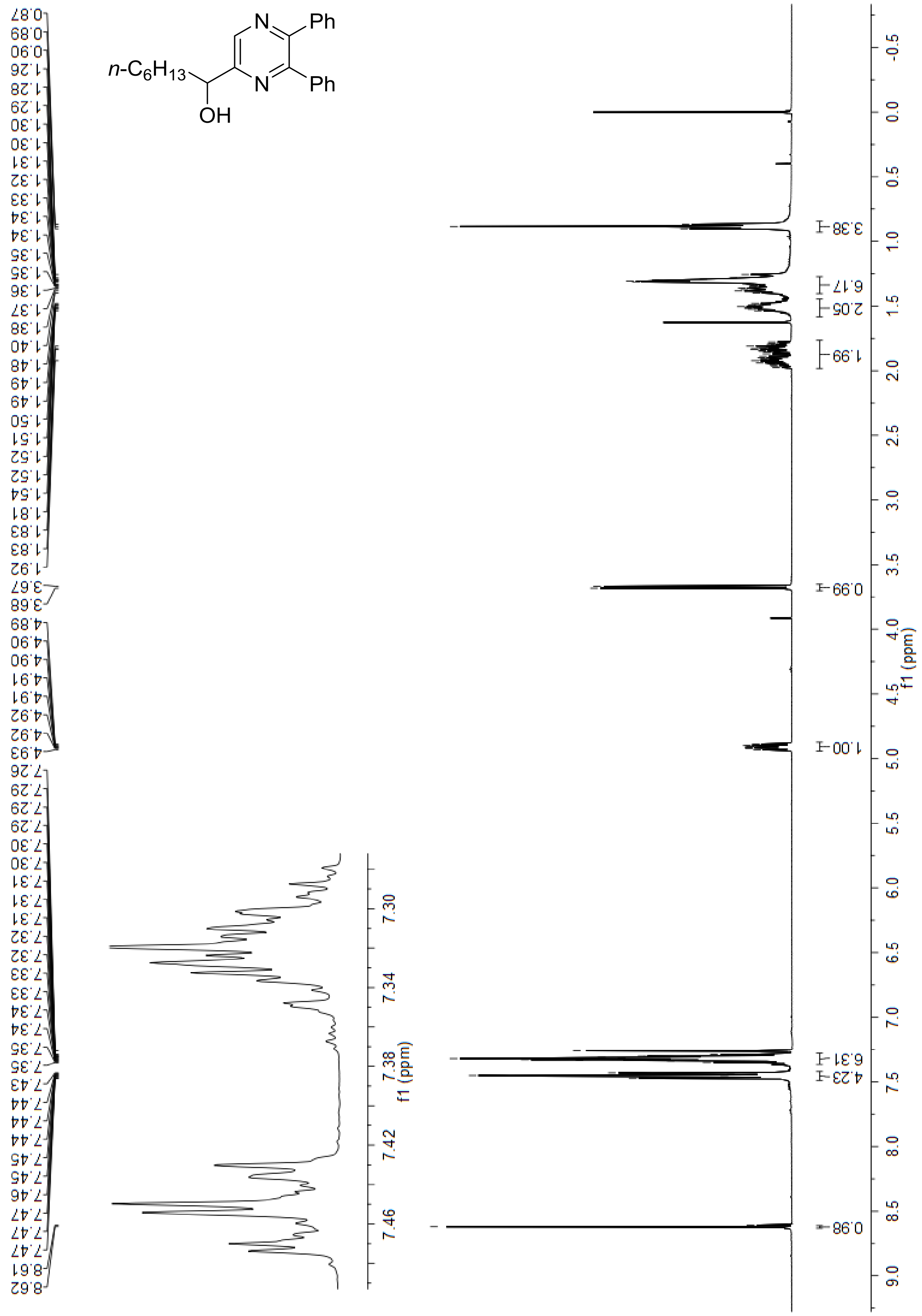

13CNMR of **5ad** ($CDCl_3$, 151 MHz, 25 ºC)

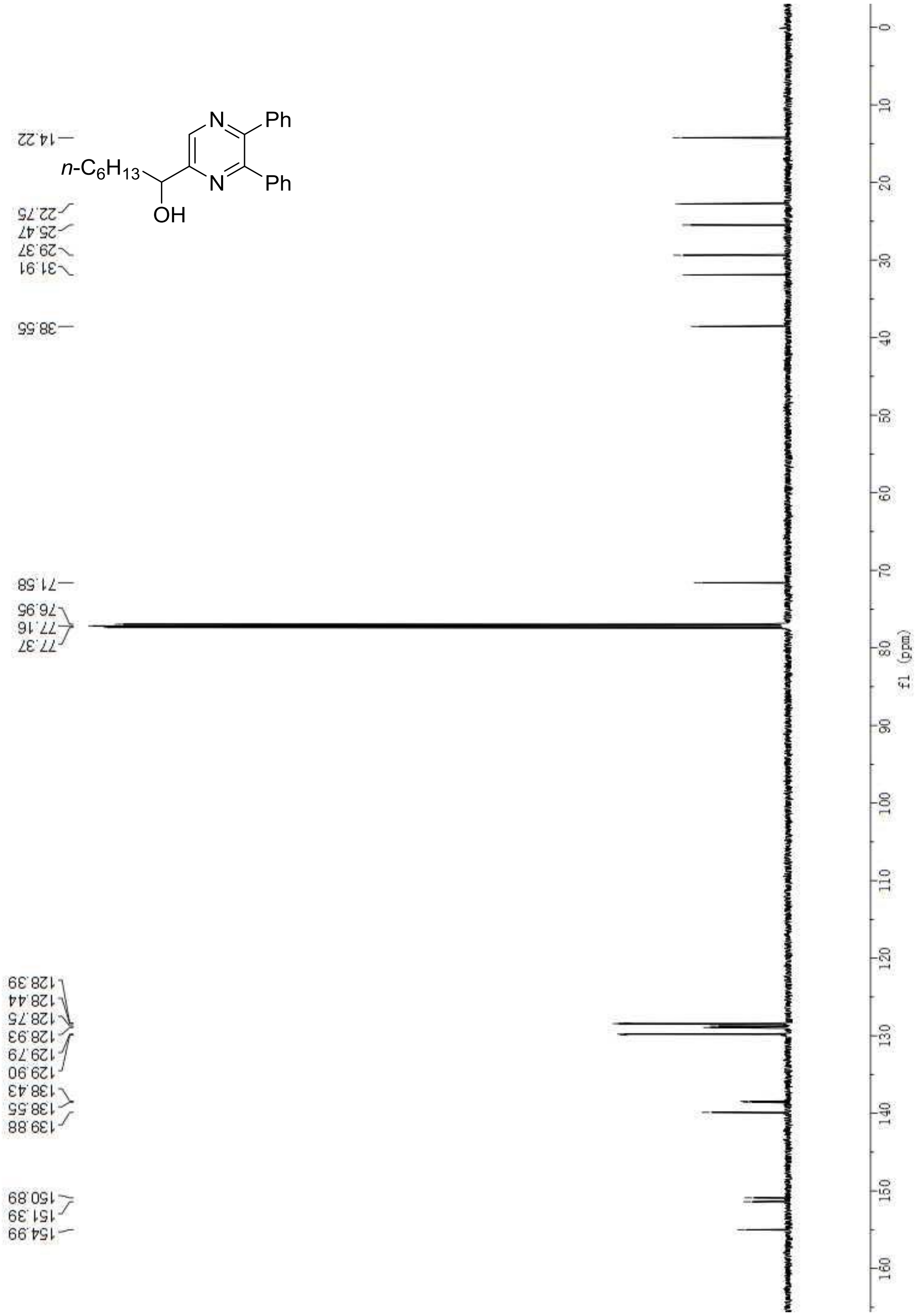

1-(dimethyl(phenyl)silyl)dodecan-1-ol (1d)

^1H NMR of **1d** ($CDCl_3$, 400 MHz, 25 ºC)

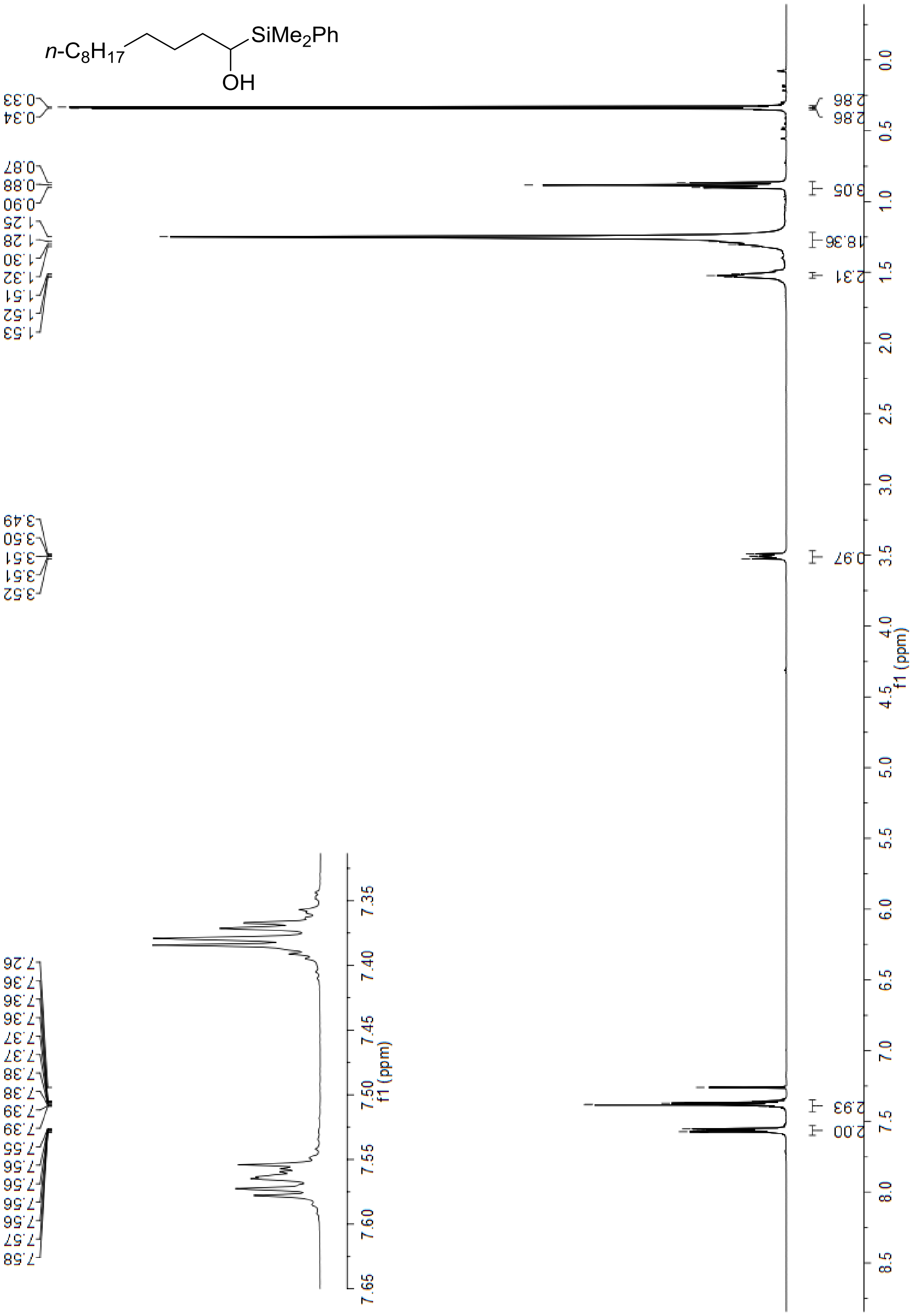

^{13}C NMR of **1d** ($CDCl_3$, 101 MHz, 25 ºC)

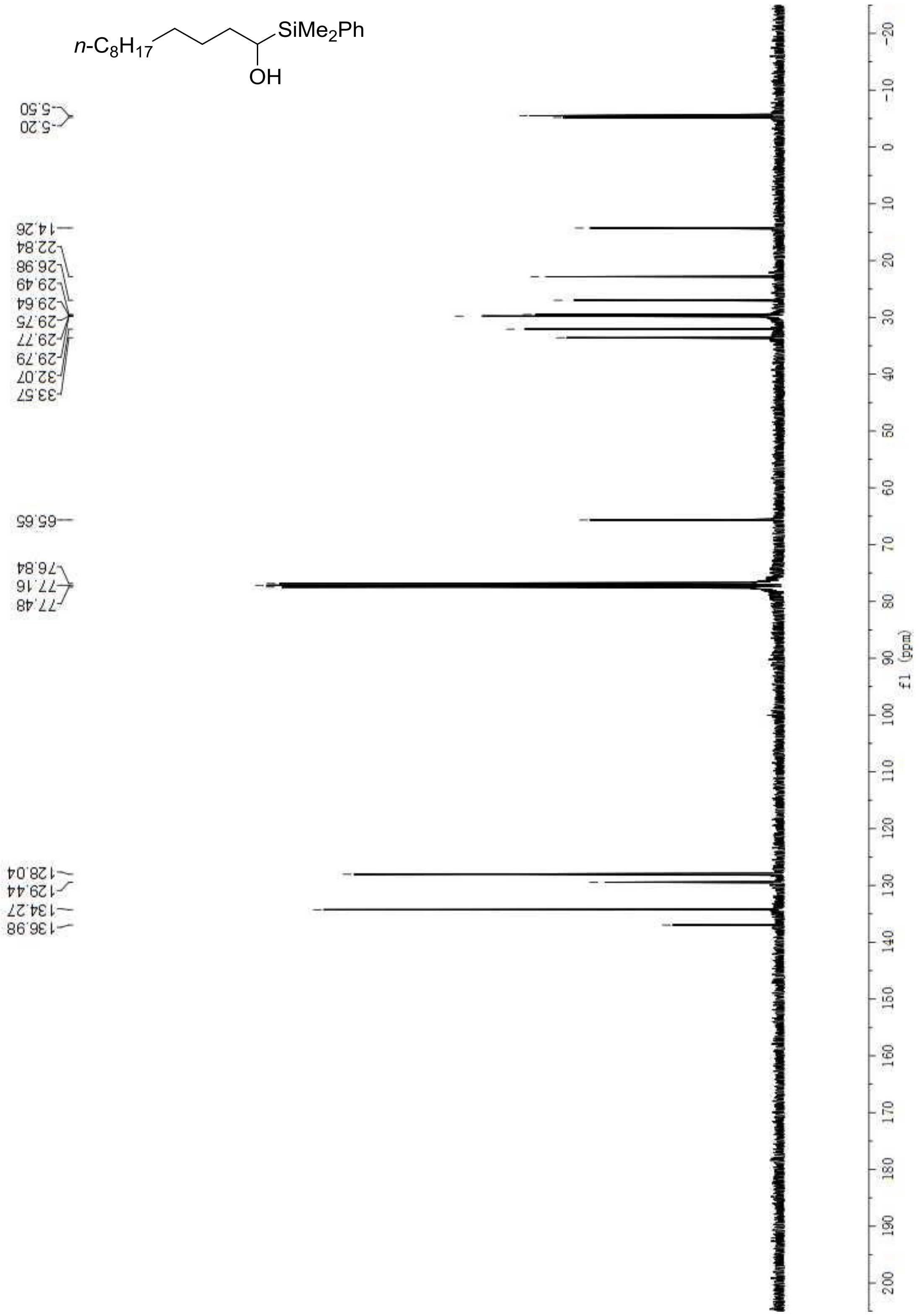

5-azido-1-(dimethyl(phenyl)silyl)pentan-1-ol (1e)

^{1}H NMR of **1e** ($CDCl_3$, 400 MHz, 25 ºC)

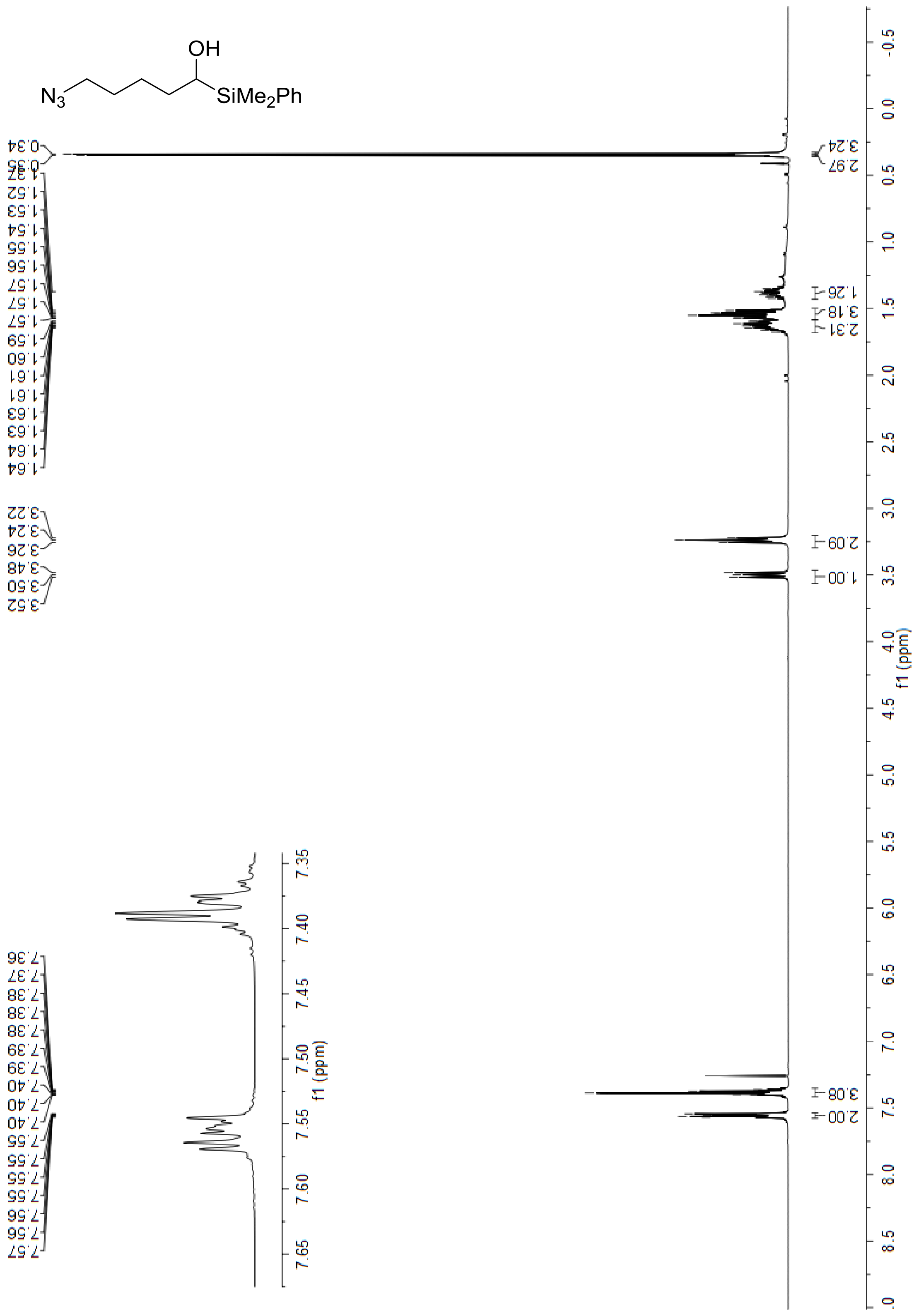

^{13}C NMR of **1e** ($CDCl_3$, 101 MHz, 25 ºC)

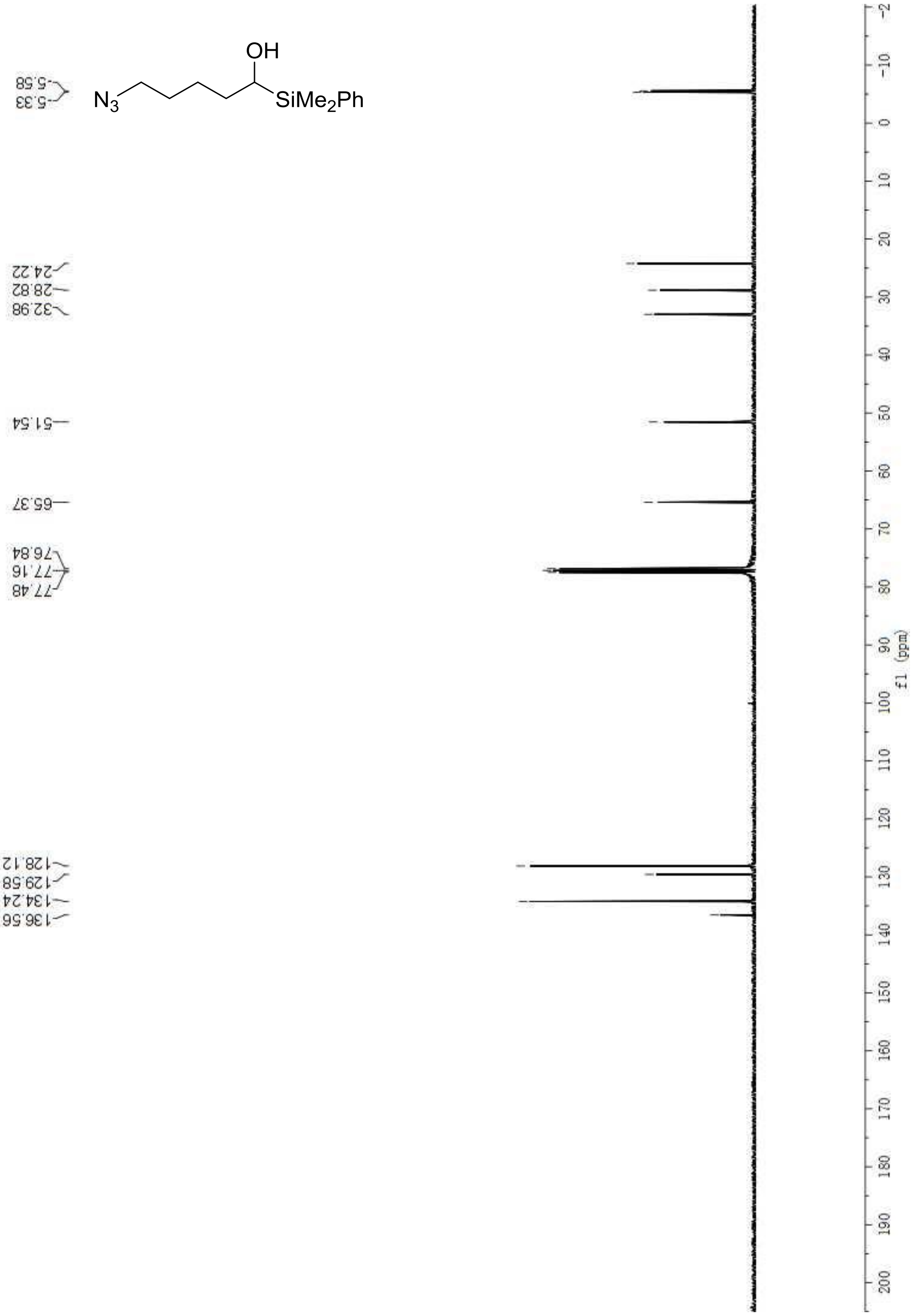

6-bromo-1-(dimethyl(phenyl)silyl)hexan-1-ol (1f)

^{1}H NMR of **1f** ($CDCl_3$, 400 MHz, 25 ºC)

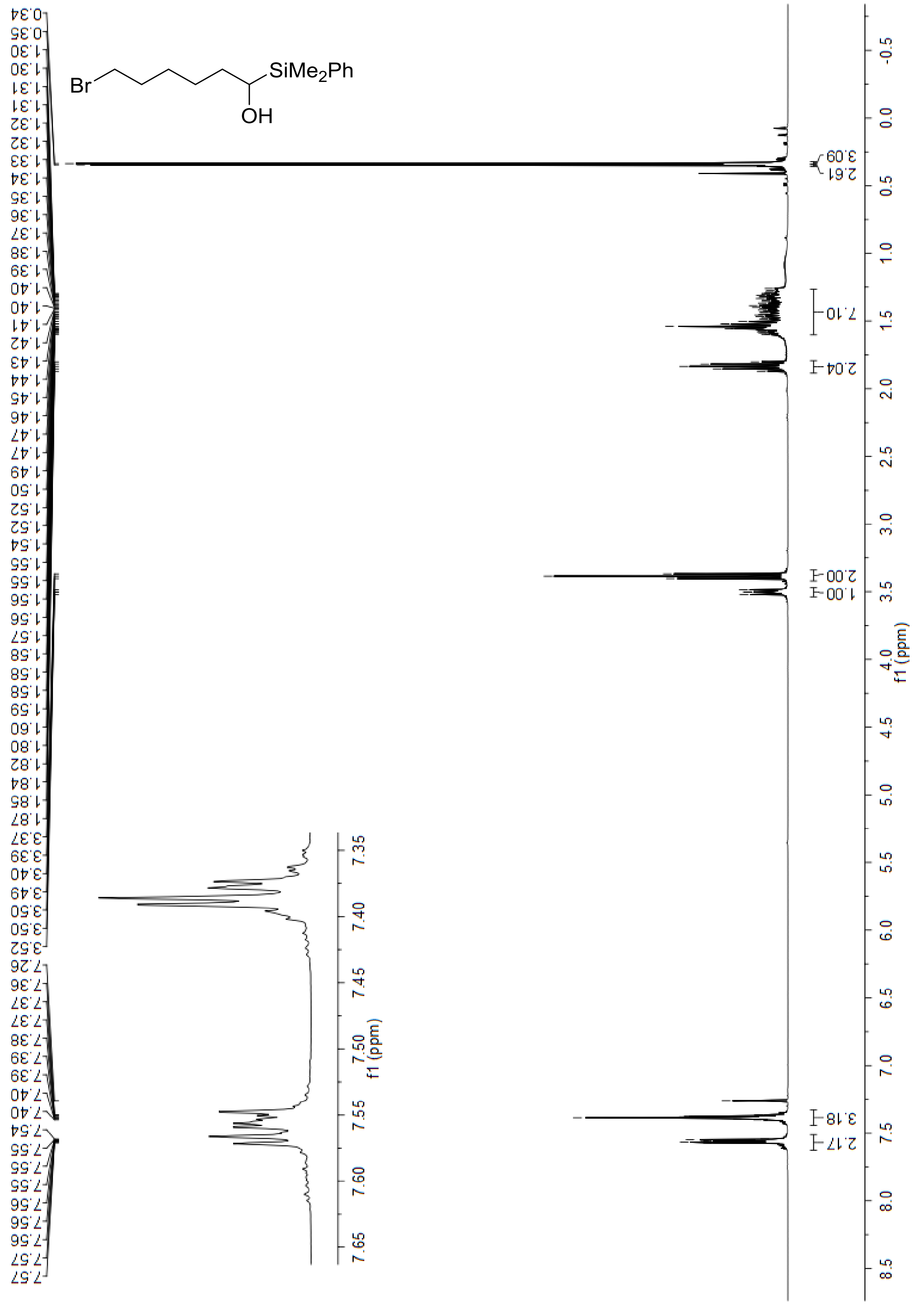

^{13}C NMR of **1f** ($CDCl_3$, 101 MHz, 25 ºC)

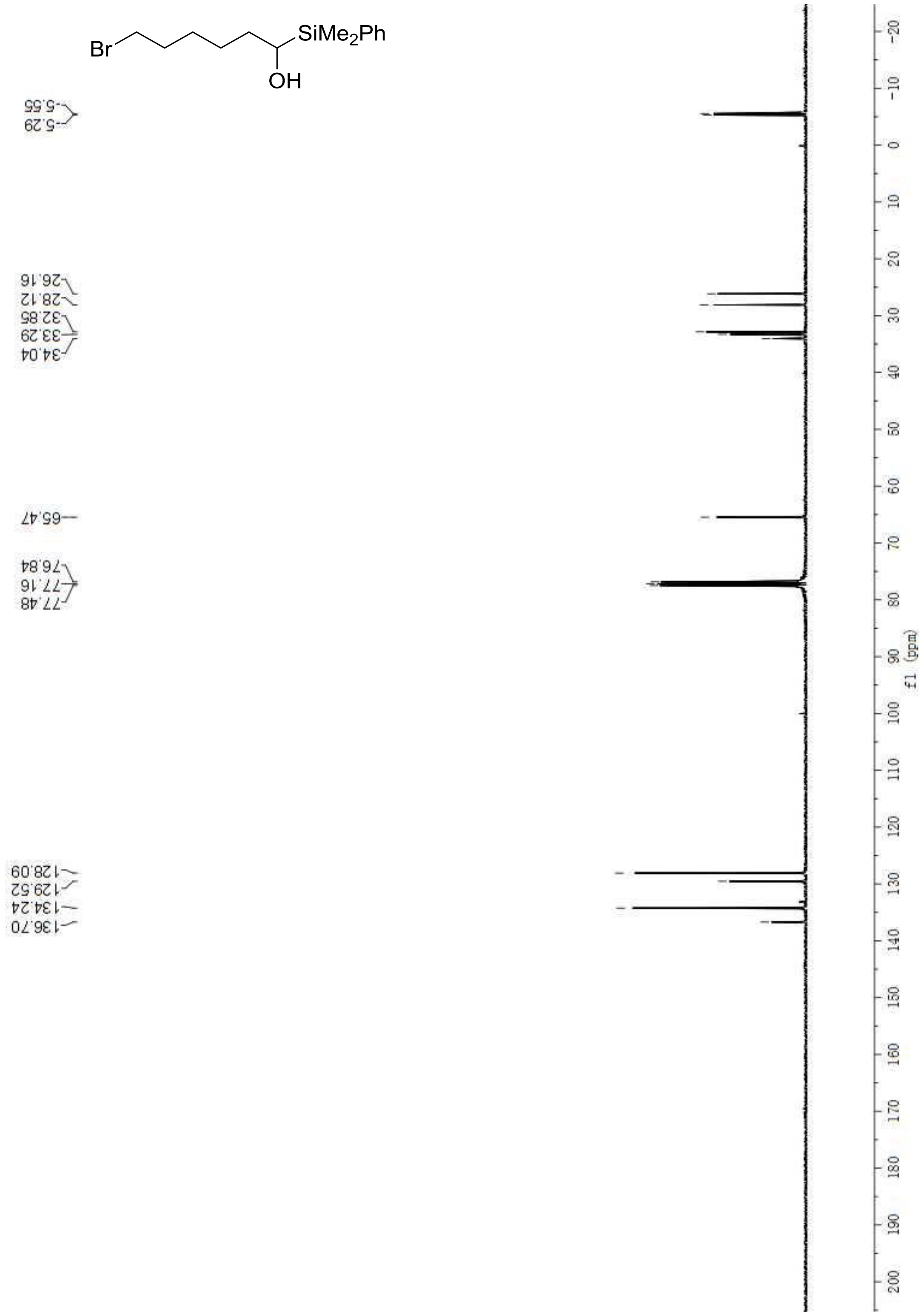

4-(benzyloxy)-1-(dimethyl(phenyl)silyl)butan-1-ol (1g)

^{1}H NMR of **1g** ($CDCl_3$, 400 MHz, 25 ºC)

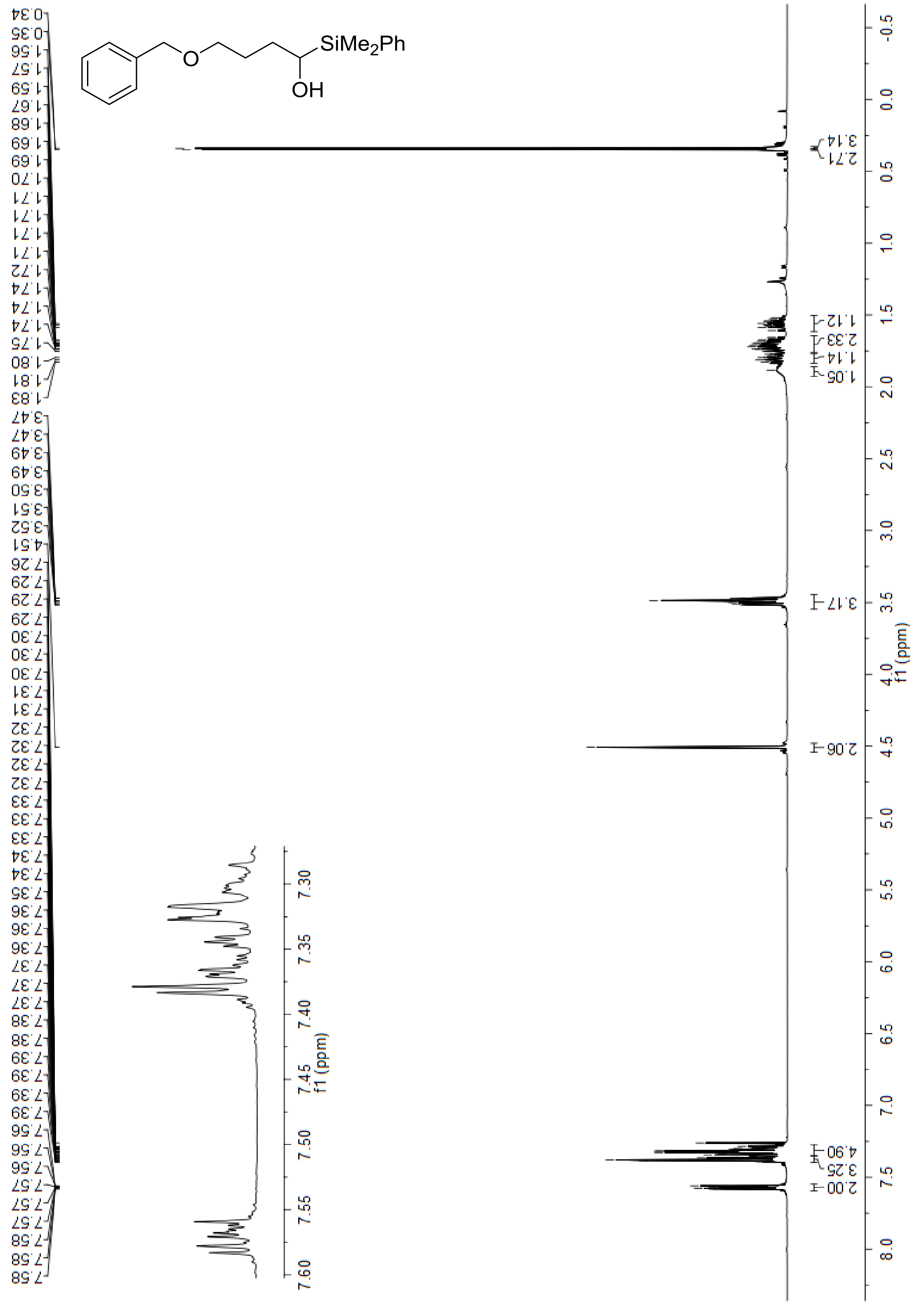

^{13}C NMR of **1g** ($CDCl_3$, 101 MHz, 25 ºC)

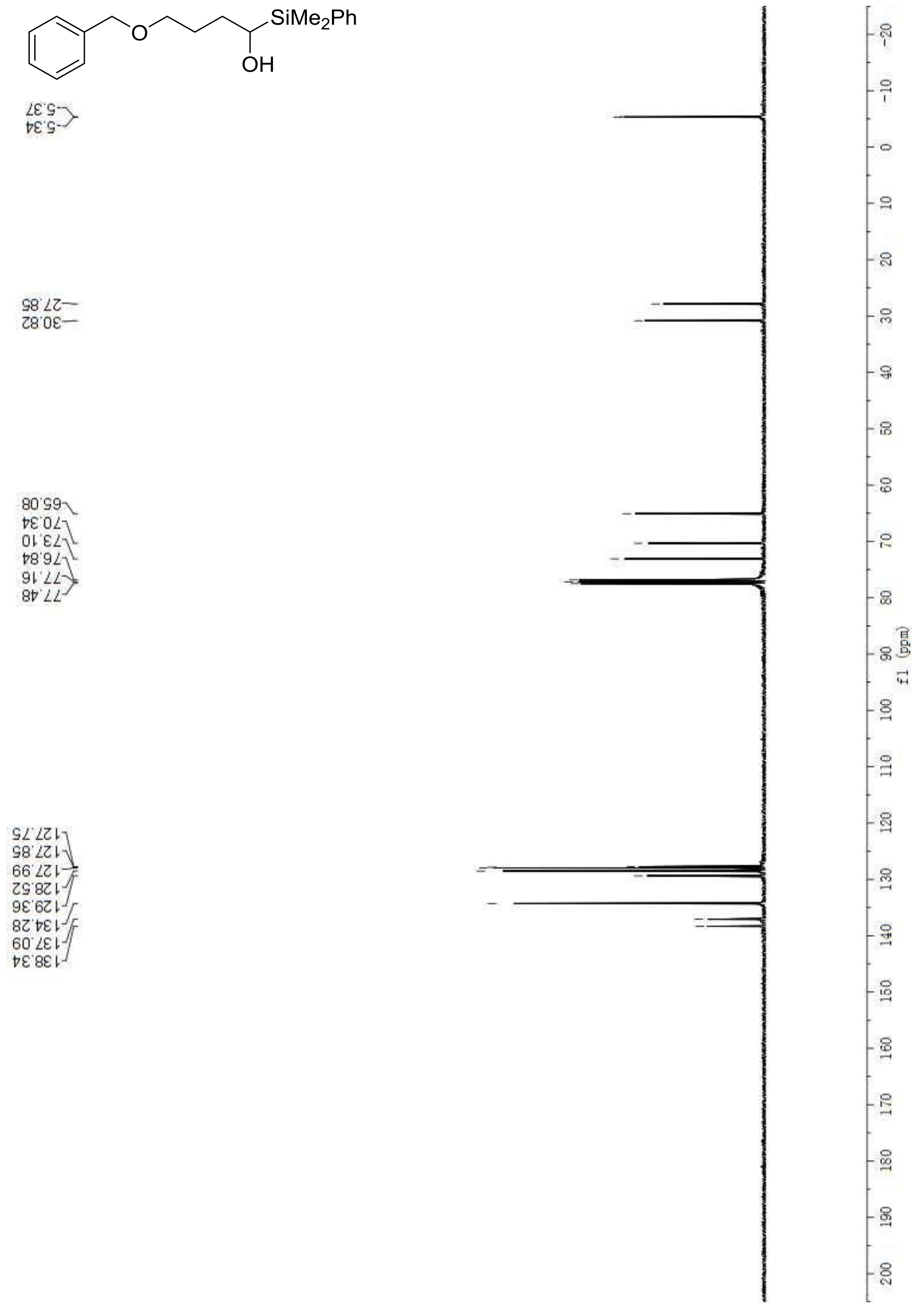

1-(dimethyl(phenyl)silyl)-5-phenoxypentan-1-ol (1h)

^{1}H NMR of **1h** ($CDCl_3$, 400 MHz, 25 ºC)

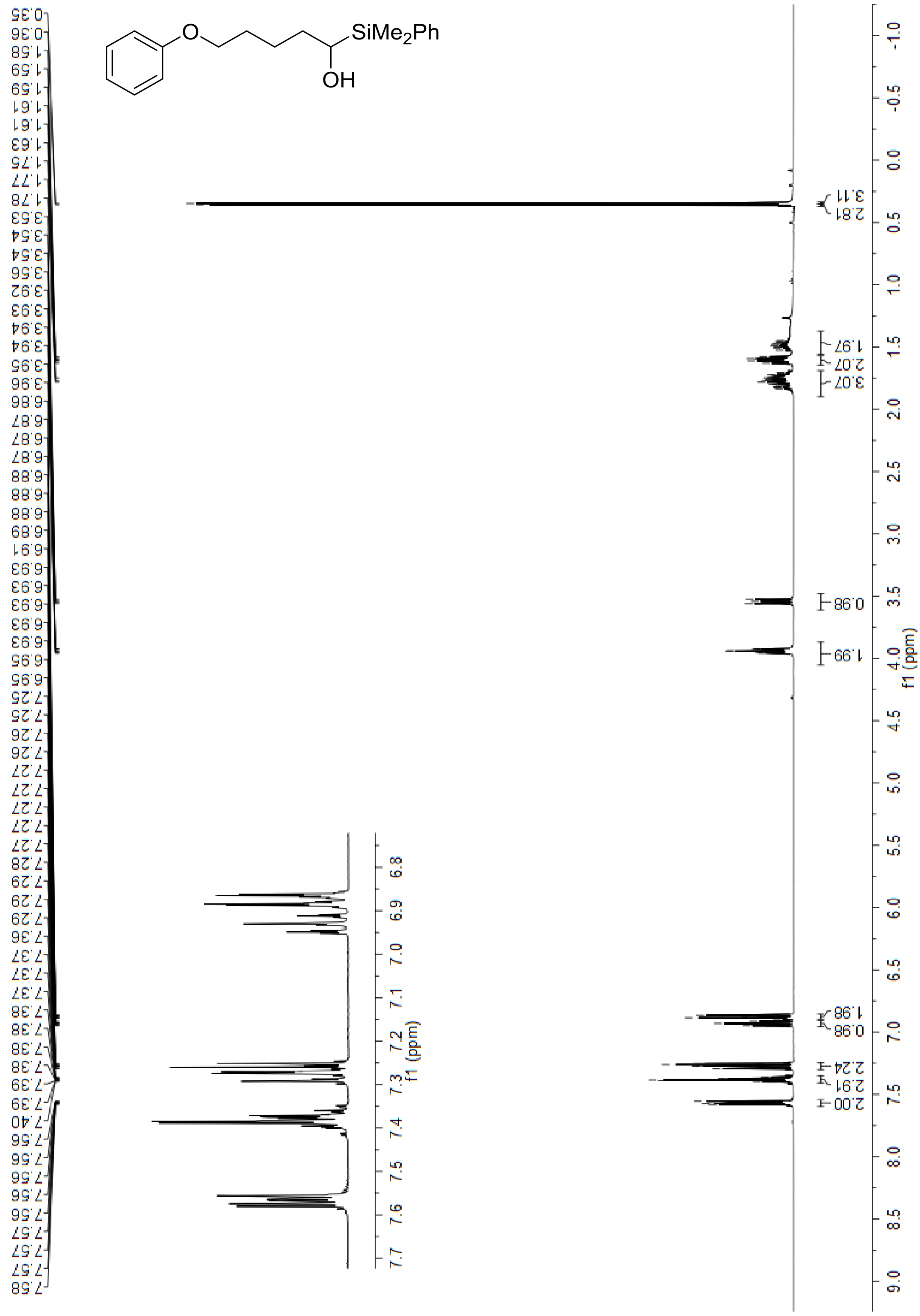

^{13}C NMR of **1h** ($CDCl_3$, 101 MHz, 25 ºC)

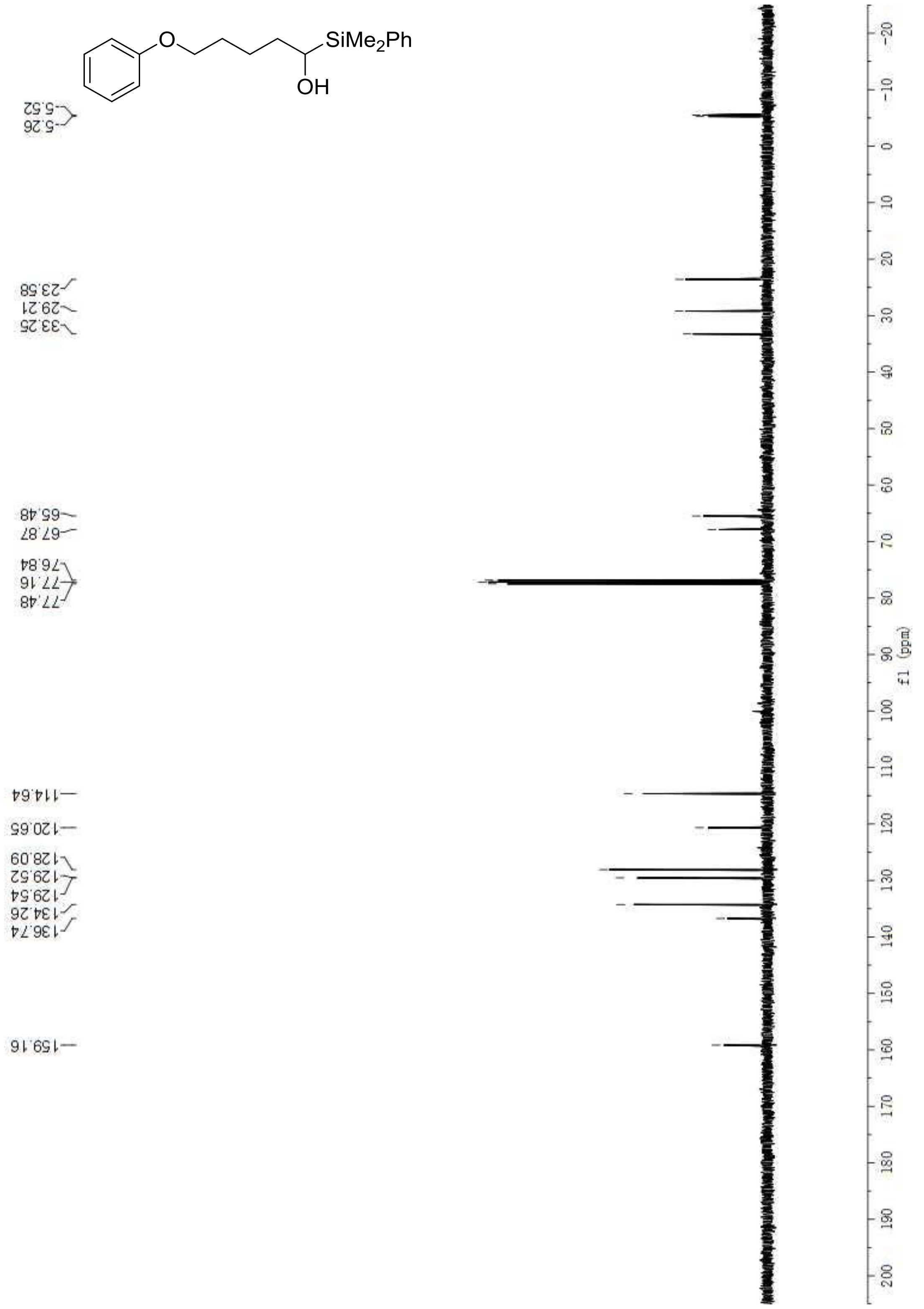

1-(dimethyl(phenyl)silyl)-4-((2-fluorobenzyl)oxy)butan-1-ol (1i)

^{1}H NMR of **1i** ($CDCl_3$, 400 MHz, 25 ºC)

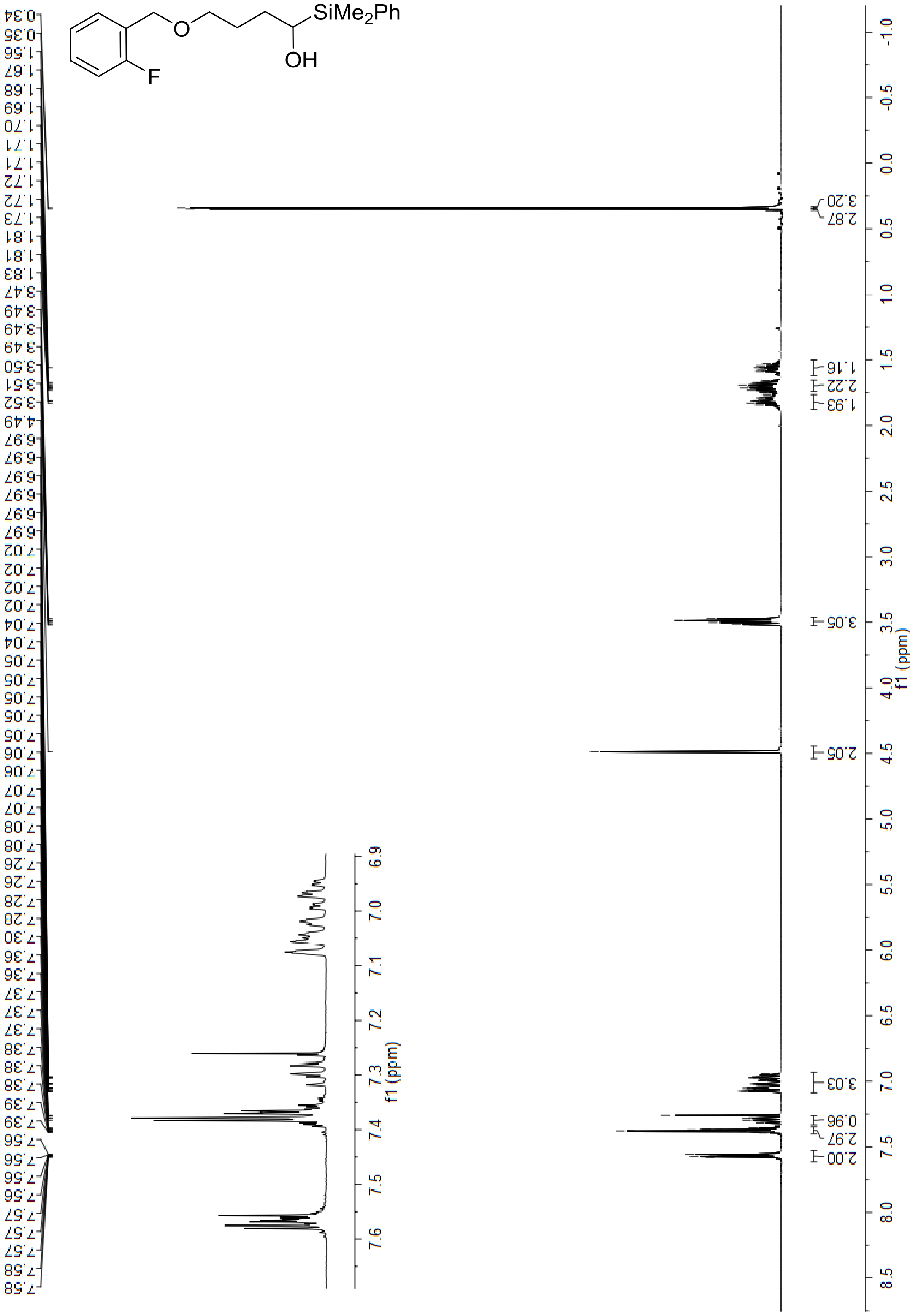

^{13}C NMR of **1i** ($CDCl_3$, 101 MHz, 25 ºC)

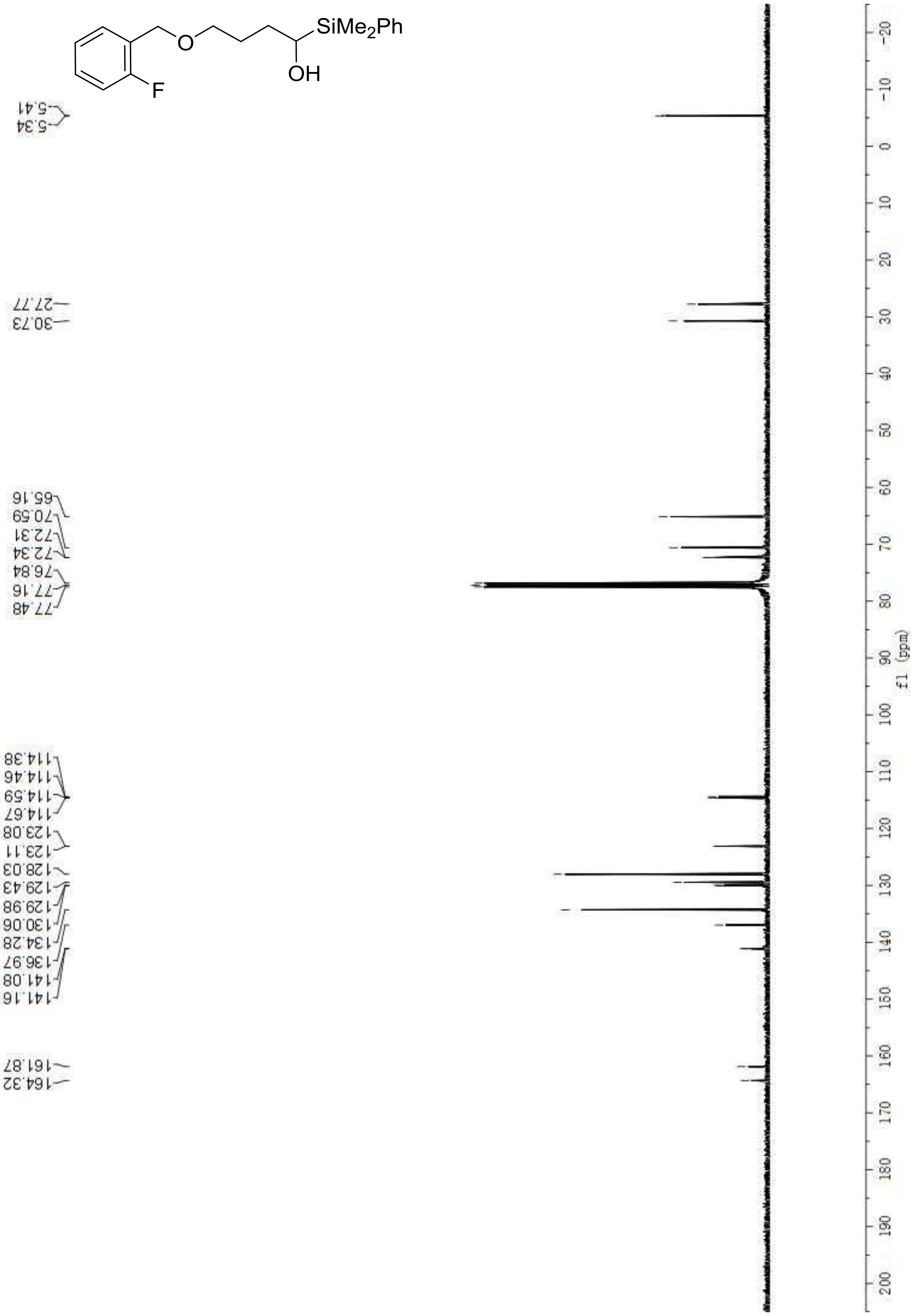

1-(dimethyl(phenyl)silyl)-4-((3-fluorobenzyl)oxy)butan-1-ol (1j)

^{1}H NMR of **1j** ($CDCl_3$, 400 MHz, 25 ºC)

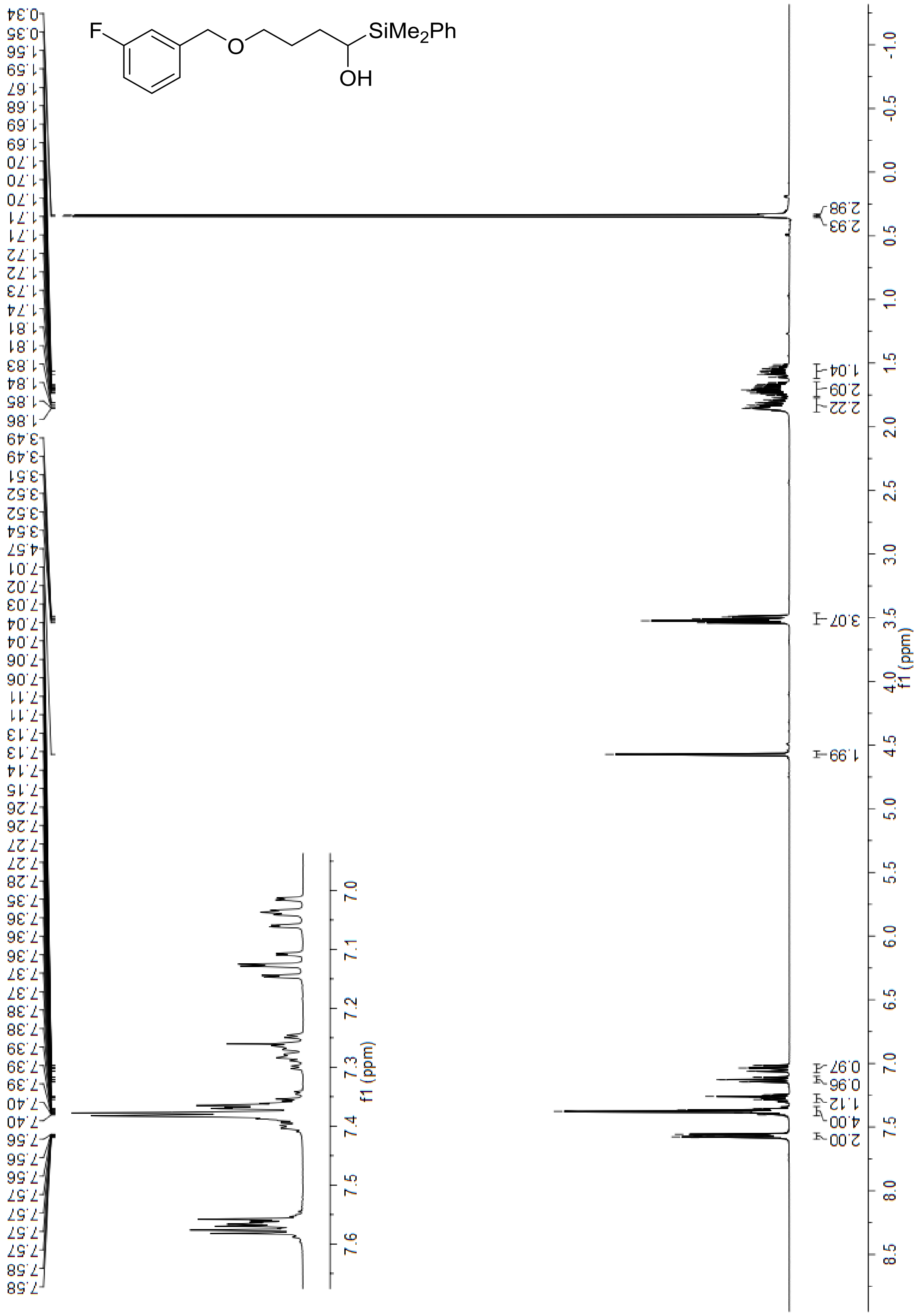

^{13}C NMR of **1j** ($CDCl_3$, 101 MHz, 25 ºC)

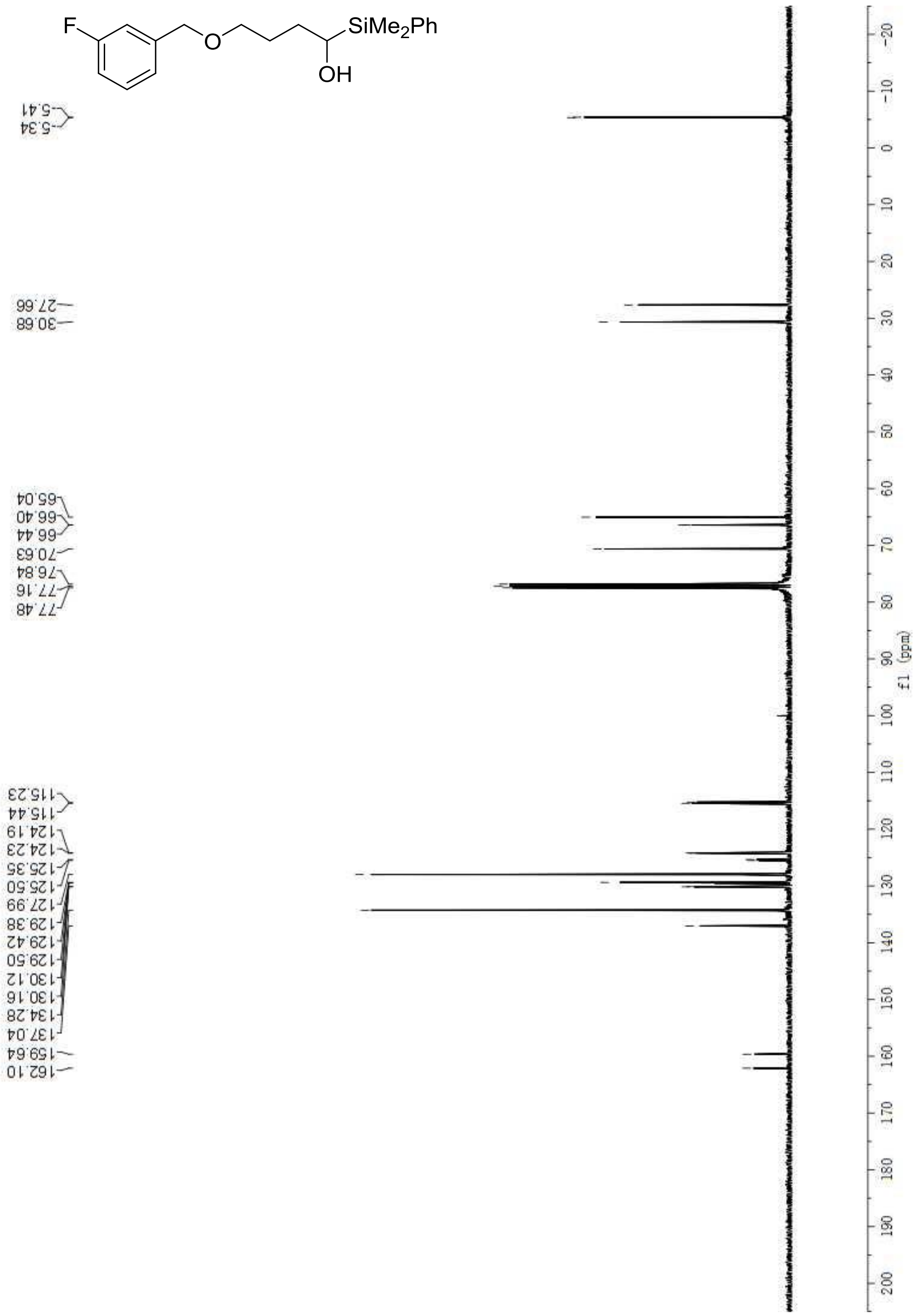

1-(dimethyl(phenyl)silyl)-4-((4-fluorobenzyl)oxy)butan-1-ol (1k)

^{1}H NMR of **1k** ($CDCl_3$, 400 MHz, 25 ºC)

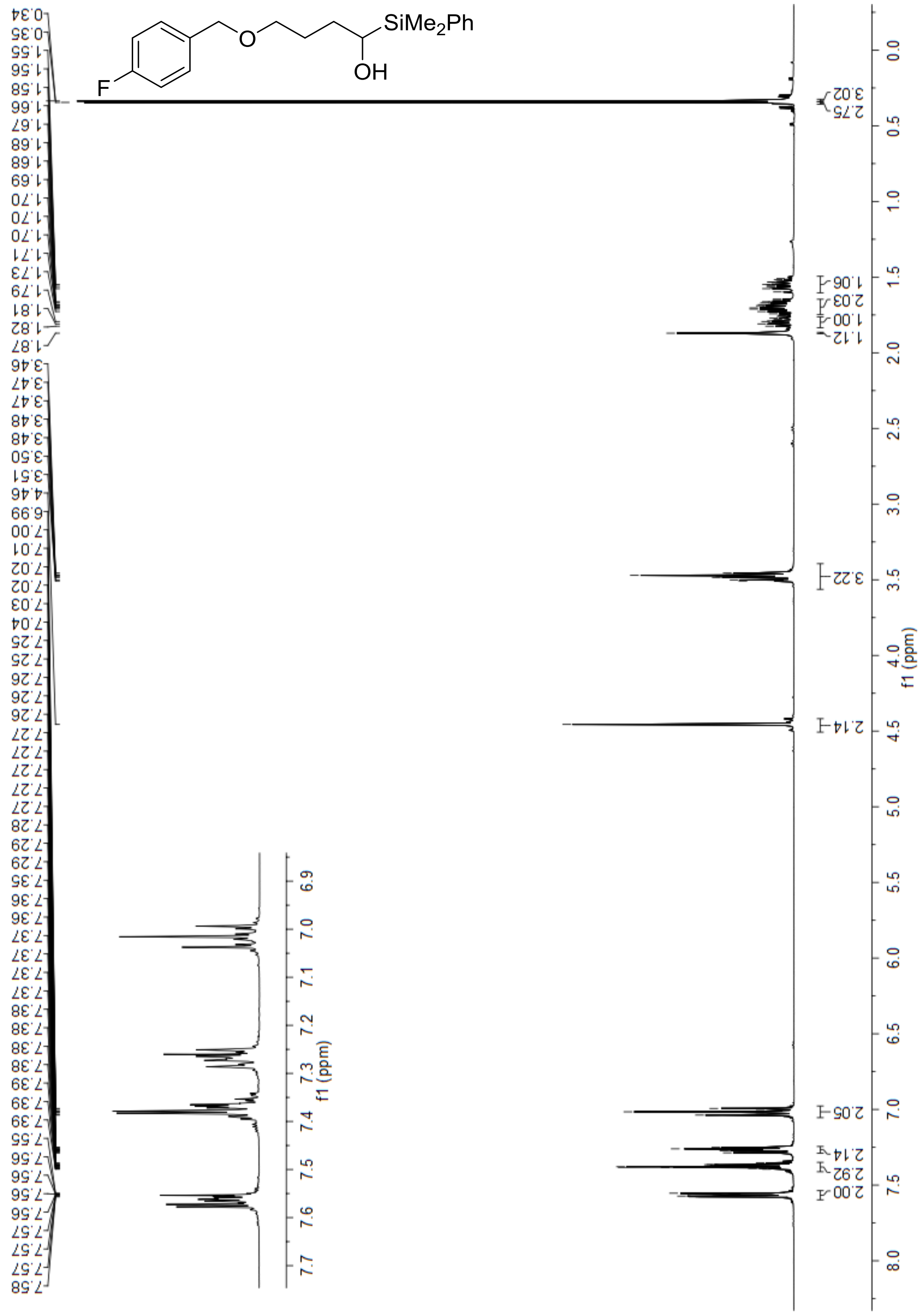

^{13}C NMR of **1k** ($CDCl_3$, 101 MHz, 25 ºC)

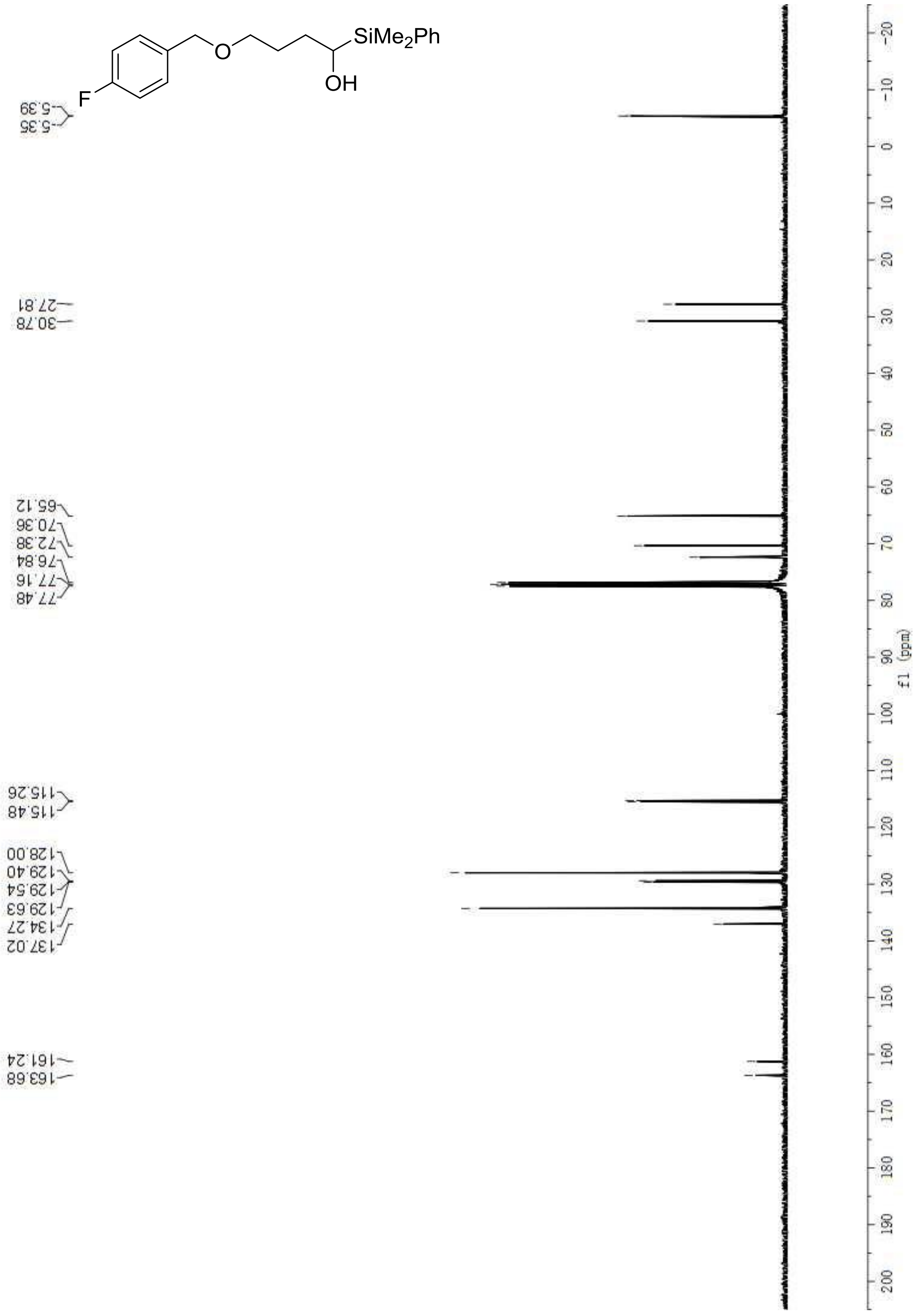

4-((3-chlorobenzyl)oxy)-1-(dimethyl(phenyl)silyl)butan-1-ol (1l)

^{1}H NMR of **1l** ($CDCl_3$, 400 MHz, 25 ºC)

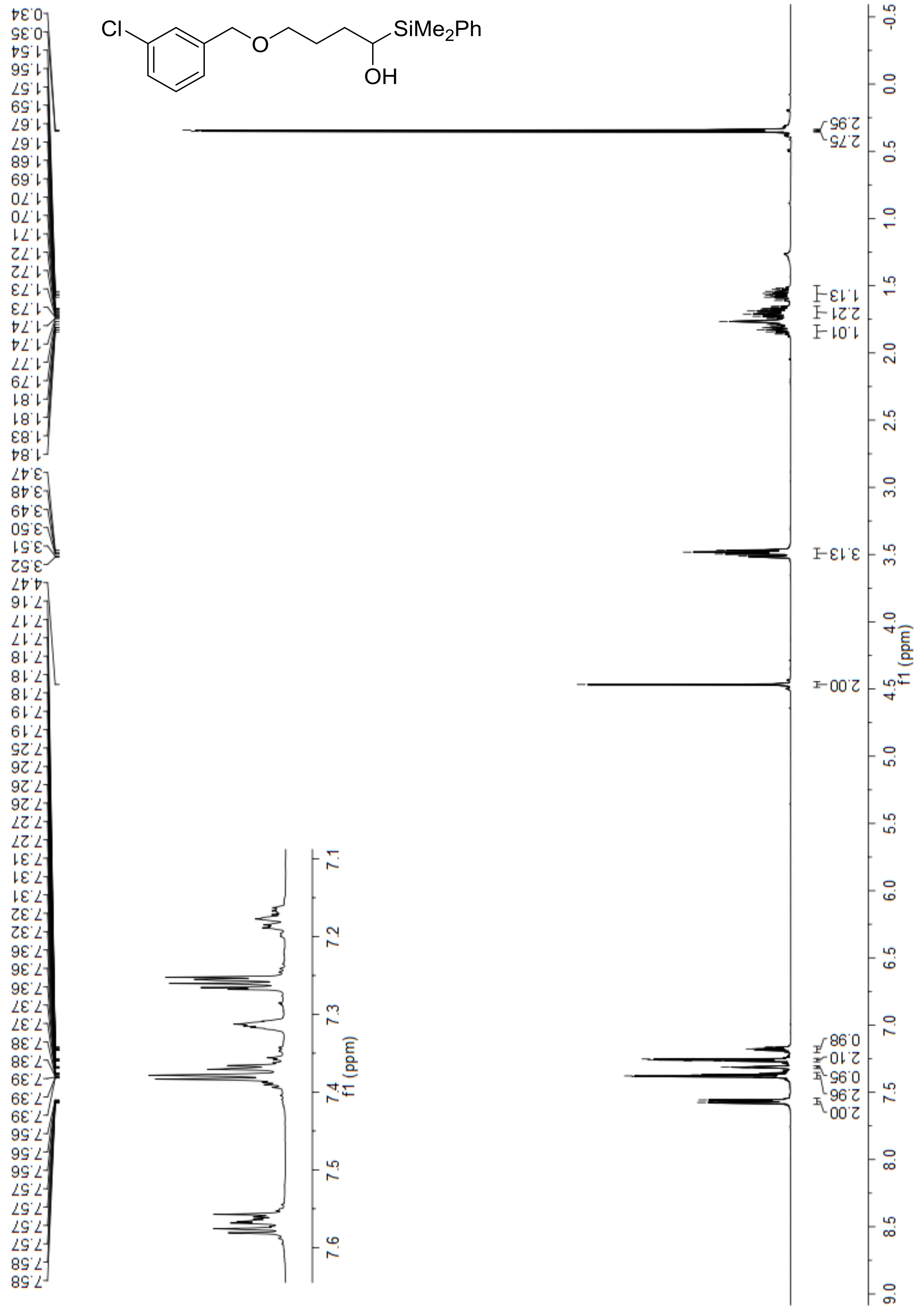

^{13}C NMR of **1l** ($CDCl_3$, 101 MHz, 25 ºC)

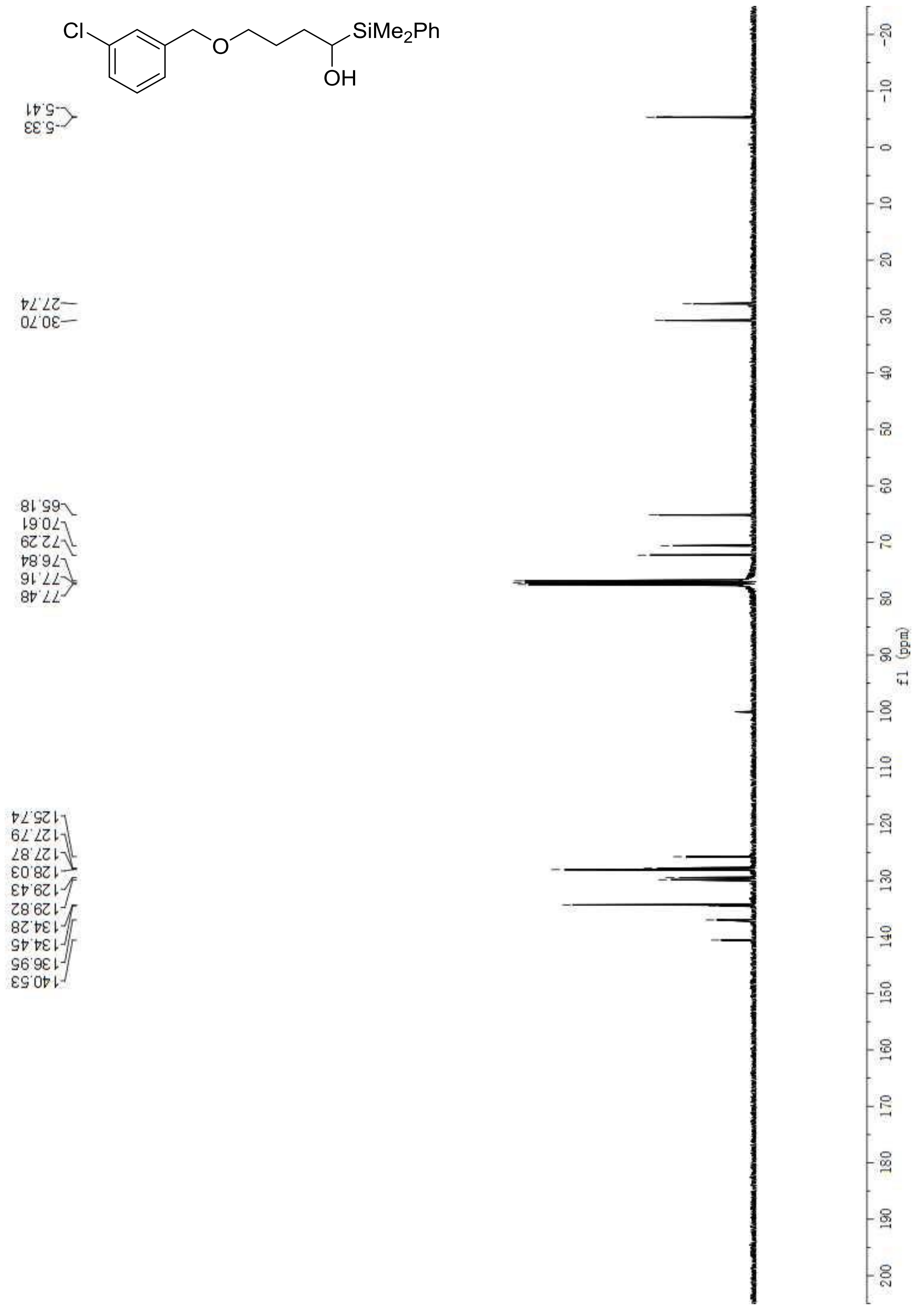

4-((3-bromobenzyl)oxy)-1-(dimethyl(phenyl)silyl)butan-1-ol (1m)

^{1}H NMR of **1m** ($CDCl_3$, 400 MHz, 25 ºC)

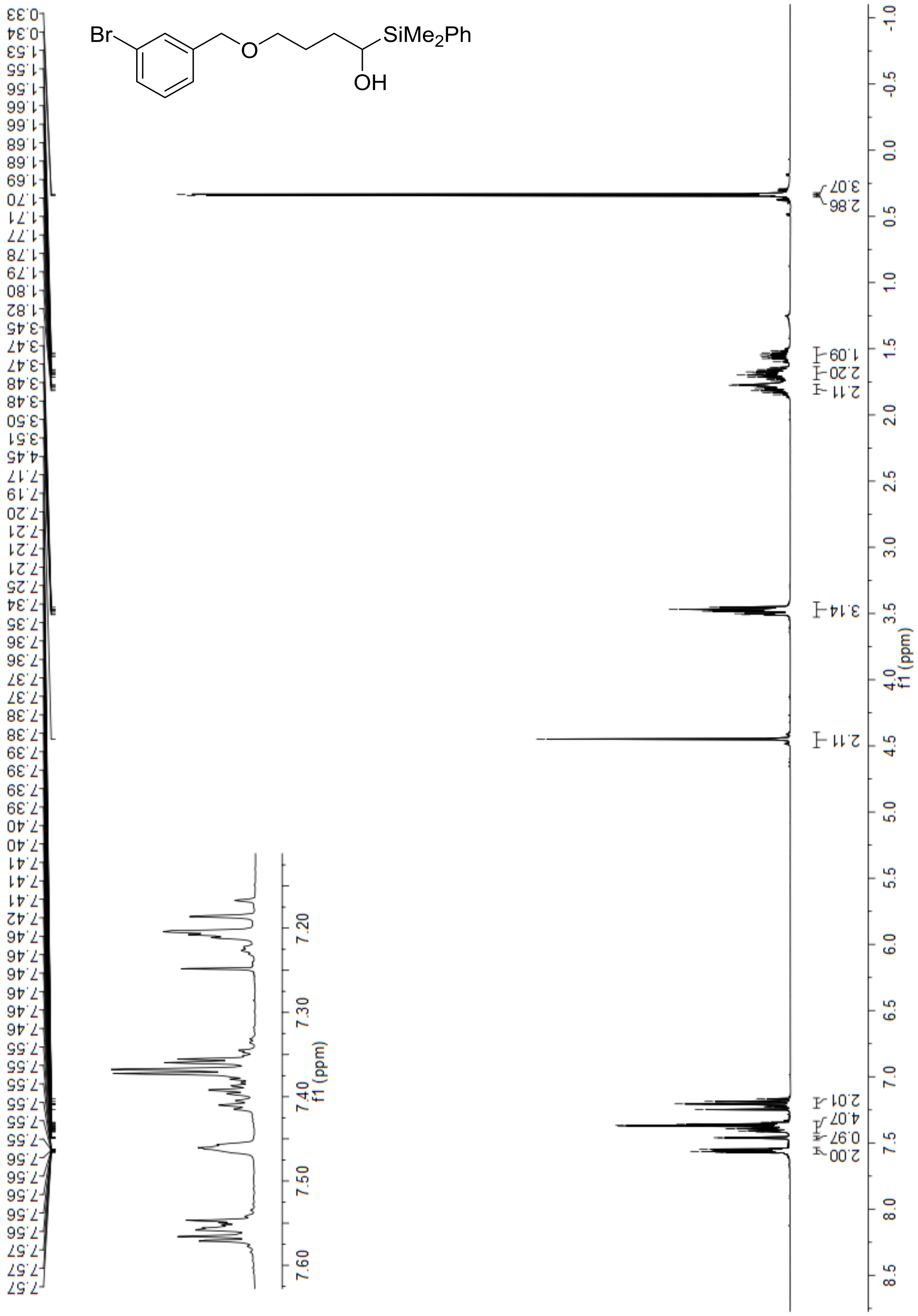

^{13}C NMR of **1m** (CDCl$_3$, 101 MHz, 25 ºC)

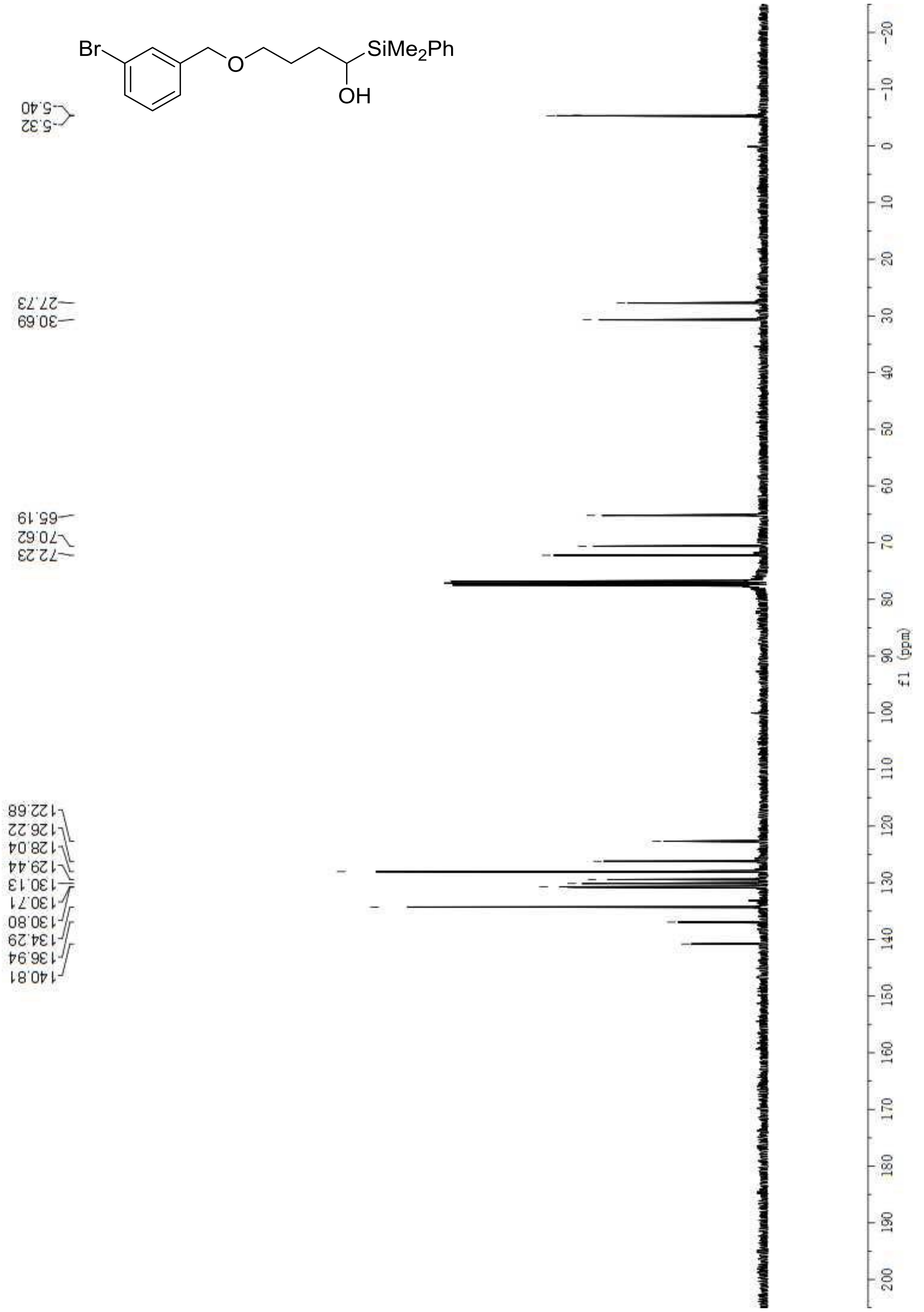

4-((3-bromobenzyl)oxy)-1-(dimethyl(phenyl)silyl)butan-1-ol (1n)

^{1}H NMR of **1n** ($CDCl_3$, 400 MHz, 25 ºC)

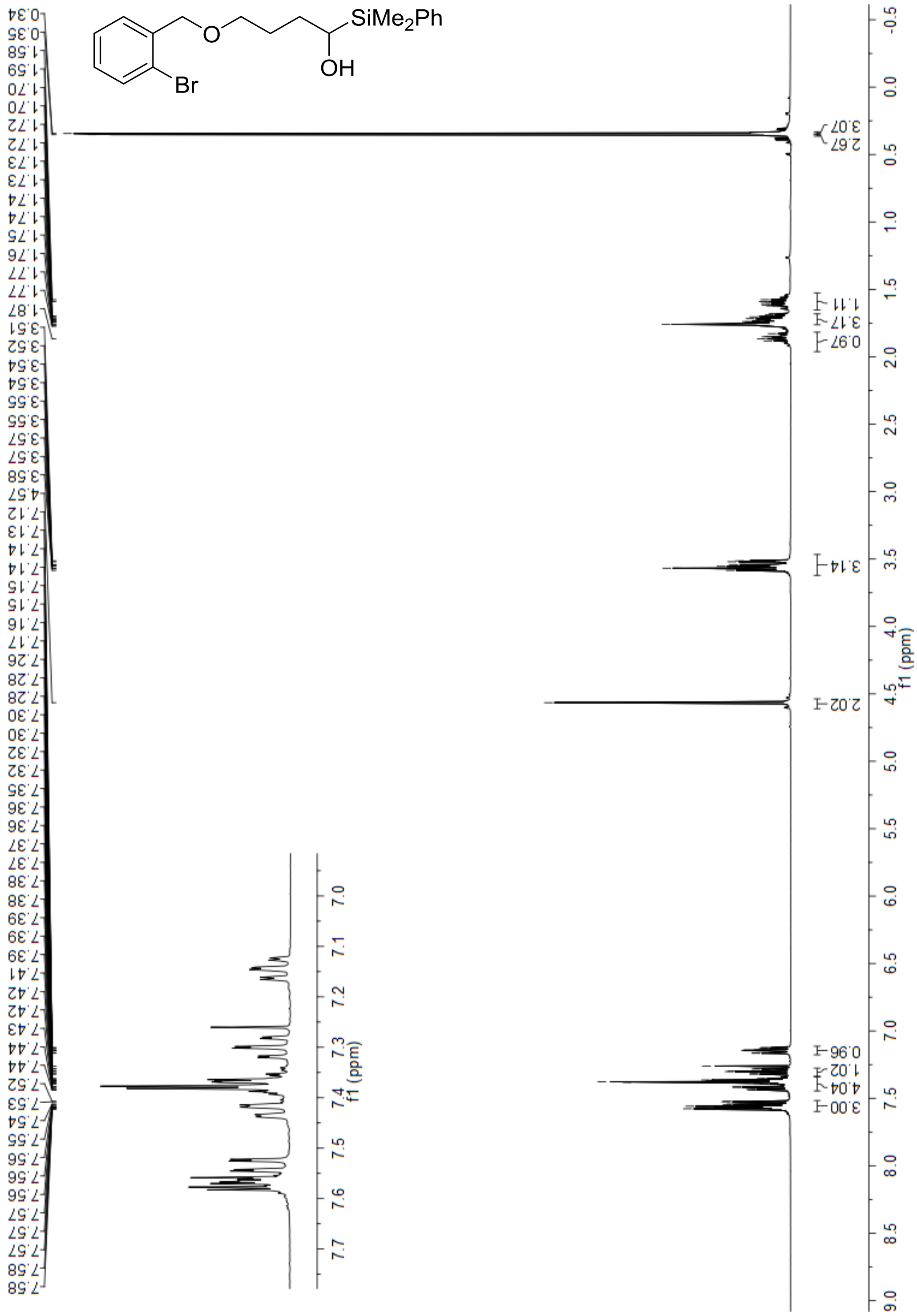

^{13}CNMR of **1n** ($CDCl_3$, 101 MHz, 25 ºC)

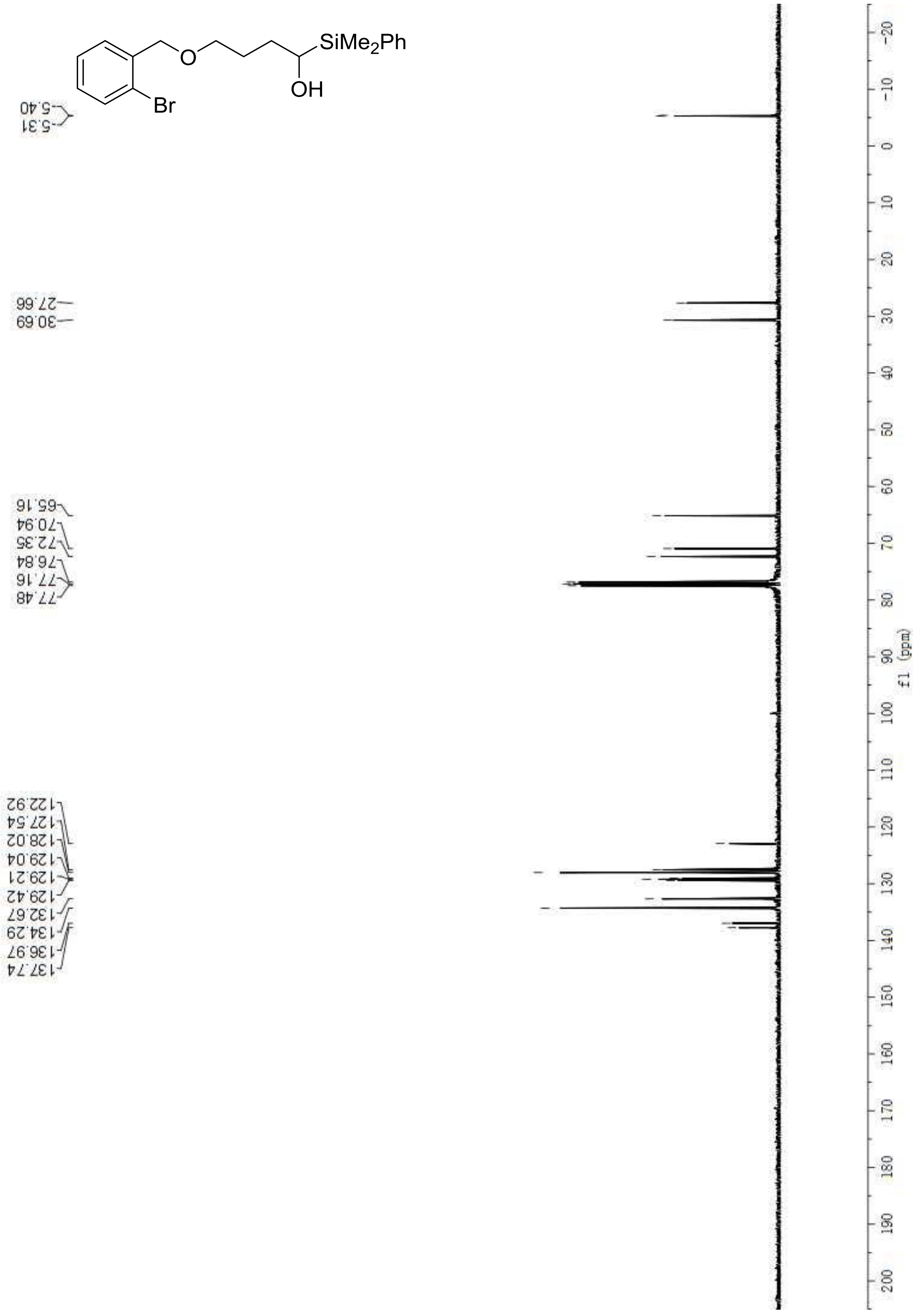

2-((4-(dimethyl(phenyl)silyl)-4-hydroxybutoxy)methyl)benzonitrile (1o)

^{1}H NMR of **1o** ($CDCl_3$, 400 MHz, 25 ºC)

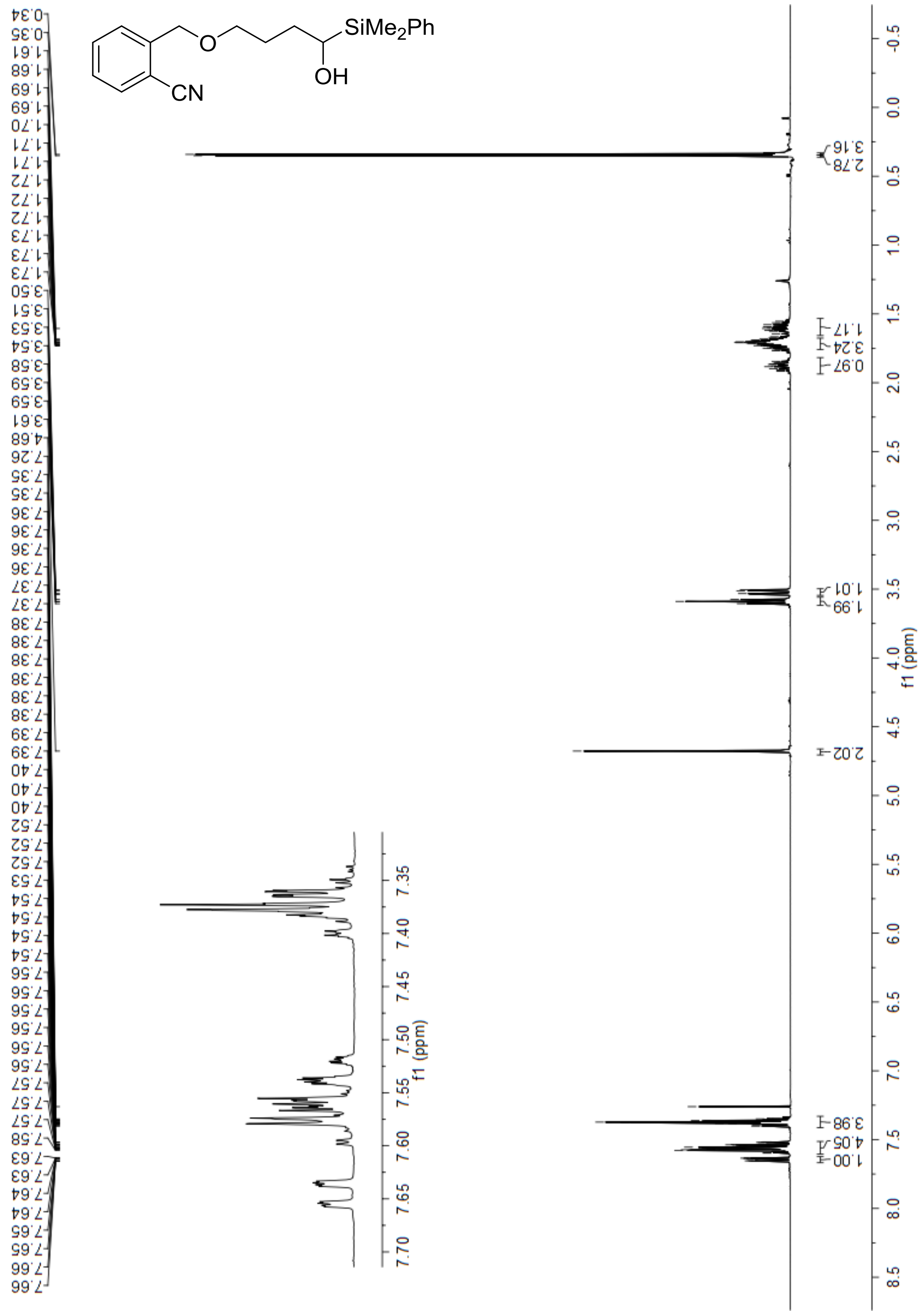

^{13}C NMR of **1o** ($CDCl_3$, 101 MHz, 25 ºC)

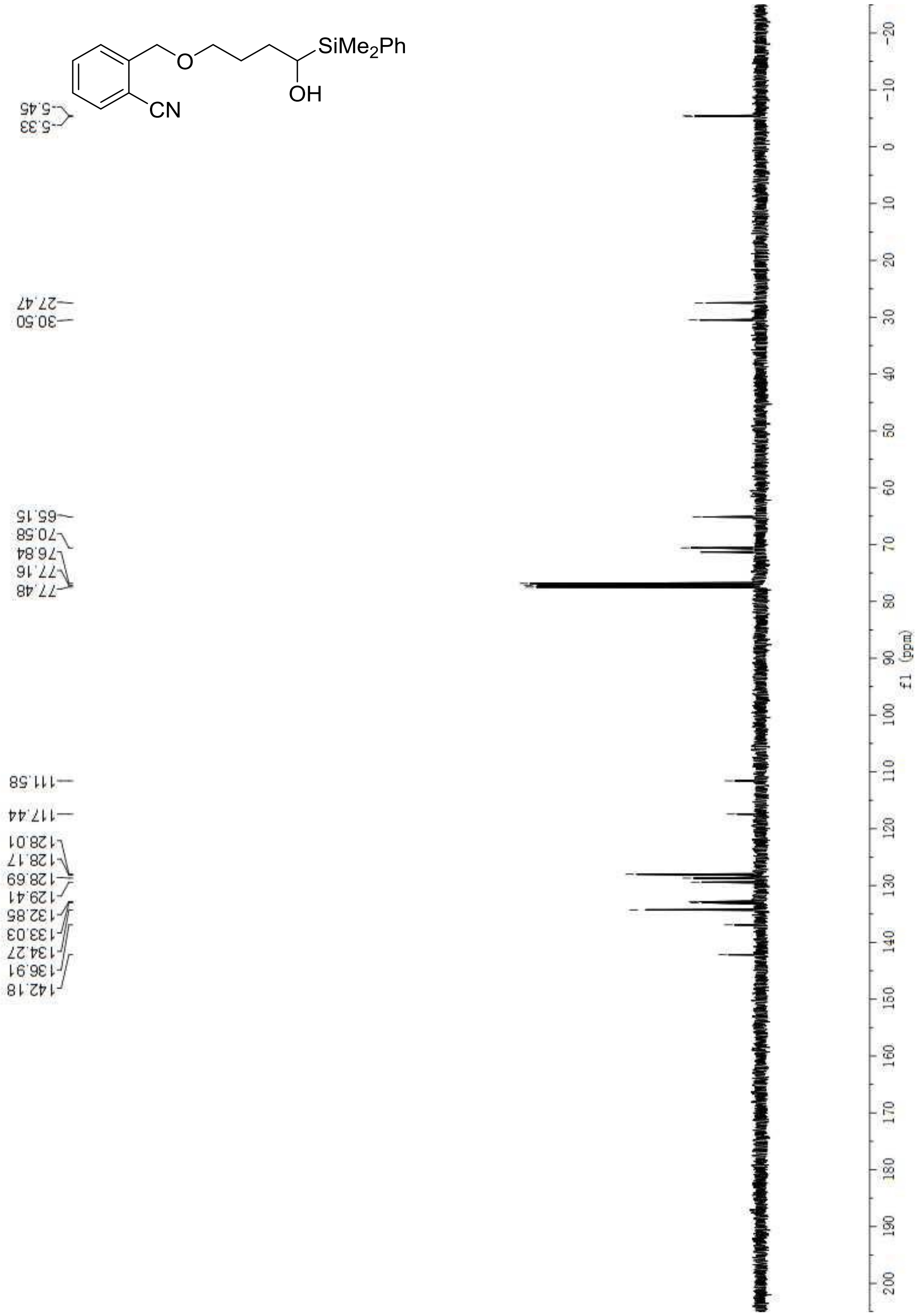

1-(dimethyl(phenyl)silyl)-6-phenoxyhexan-1-ol (1p)

^{1}H NMR of **1p** ($CDCl_3$, 400 MHz, 25 ºC)

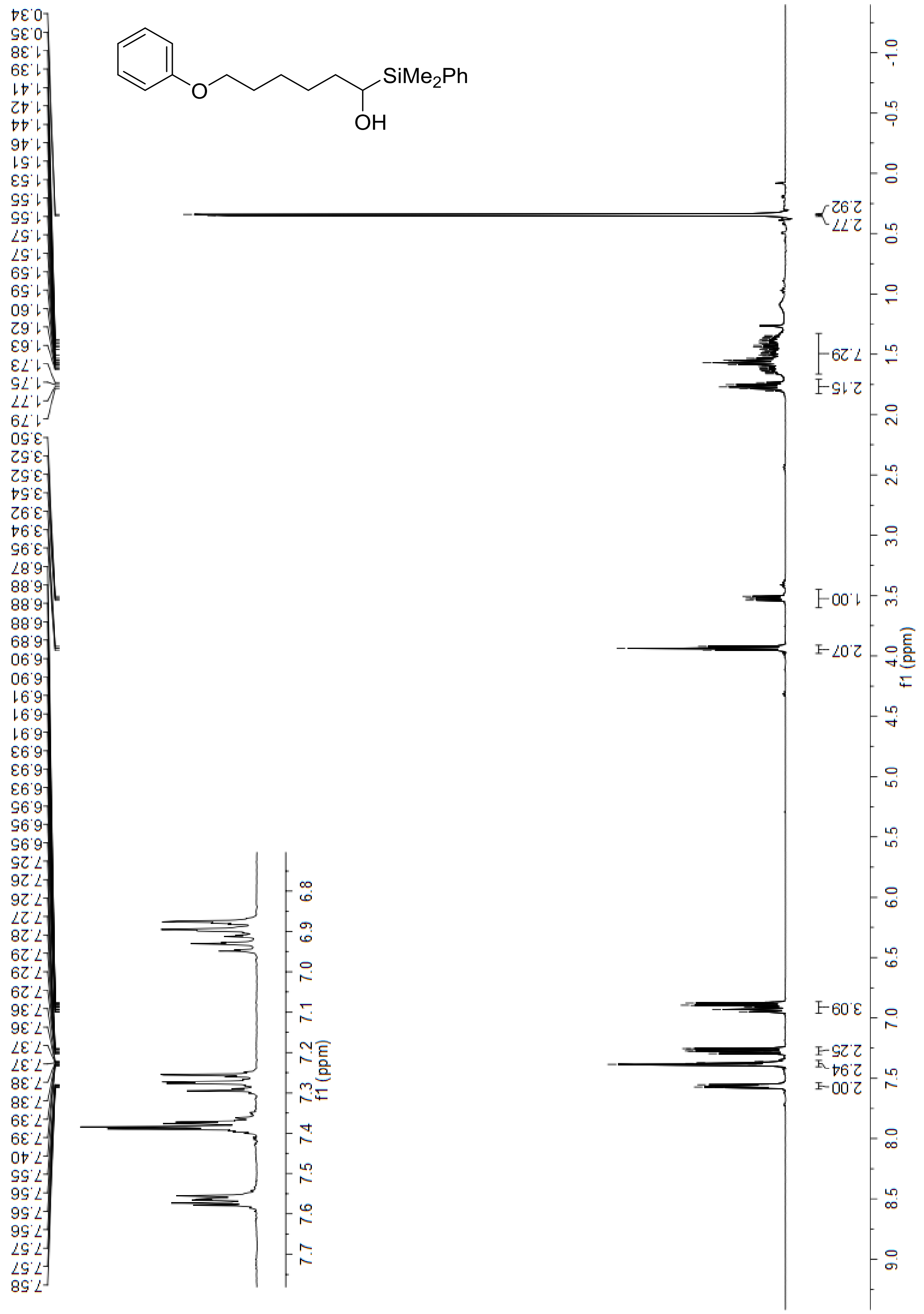

^{13}C NMR of **1p** ($CDCl_3$, 101 MHz, 25 ºC)

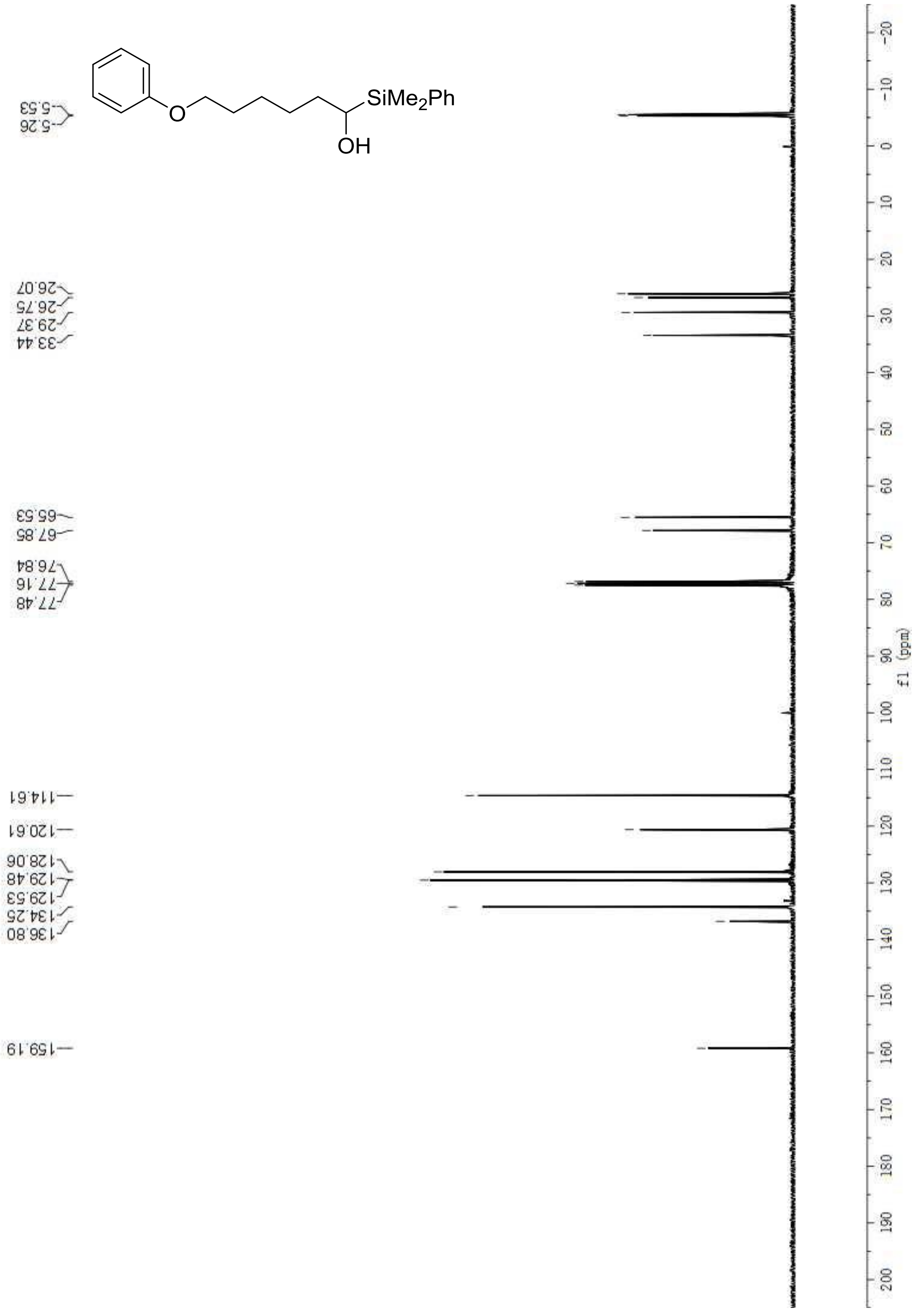

6-(dimethyl(phenyl)silyl)-6-hydroxyhexyl benzoate (1q)

^{1}H NMR of **1q** ($CDCl_3$, 400 MHz, 25 ºC)

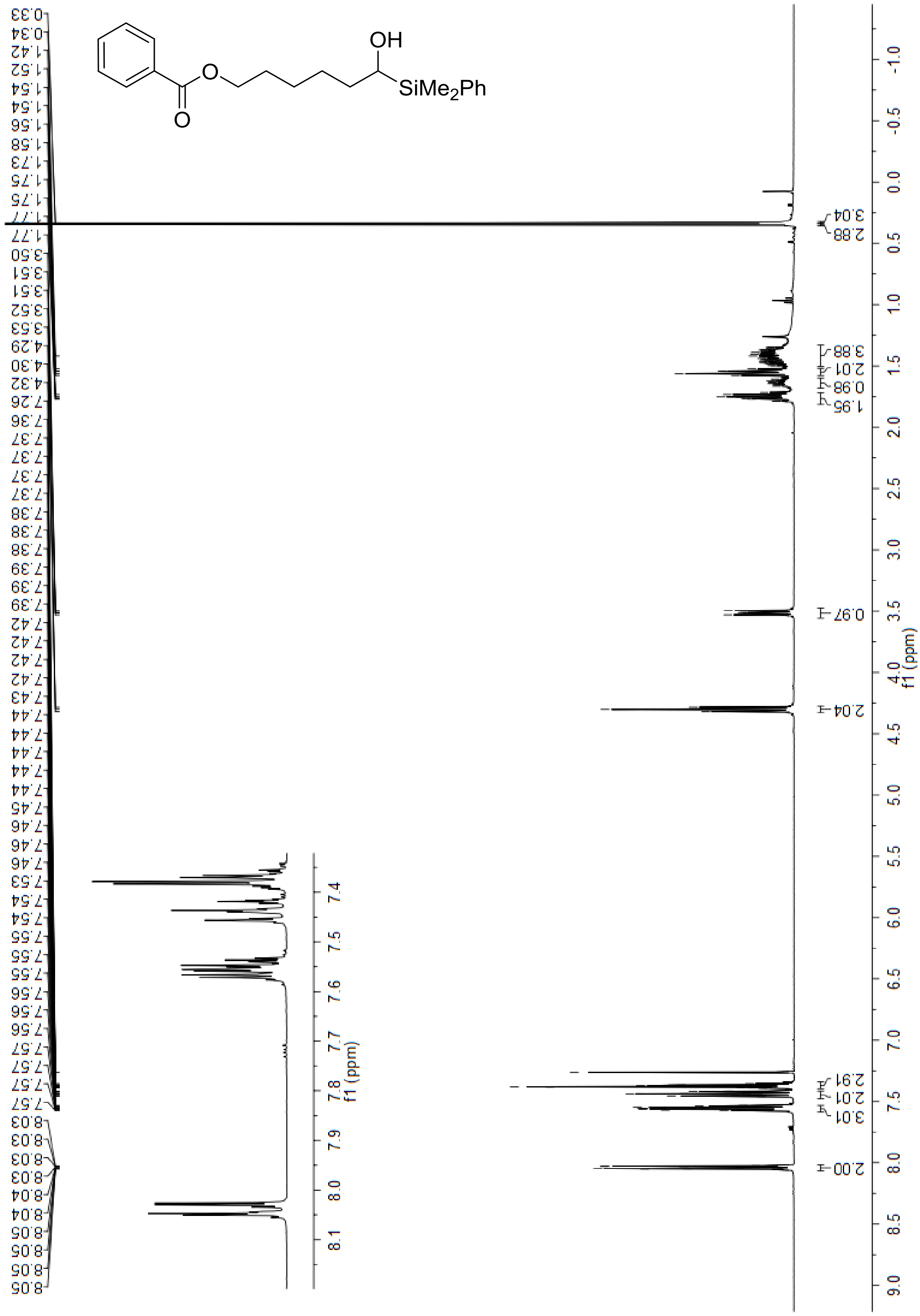

^{13}C NMR of **1q** ($CDCl_3$, 101 MHz, 25 ºC)

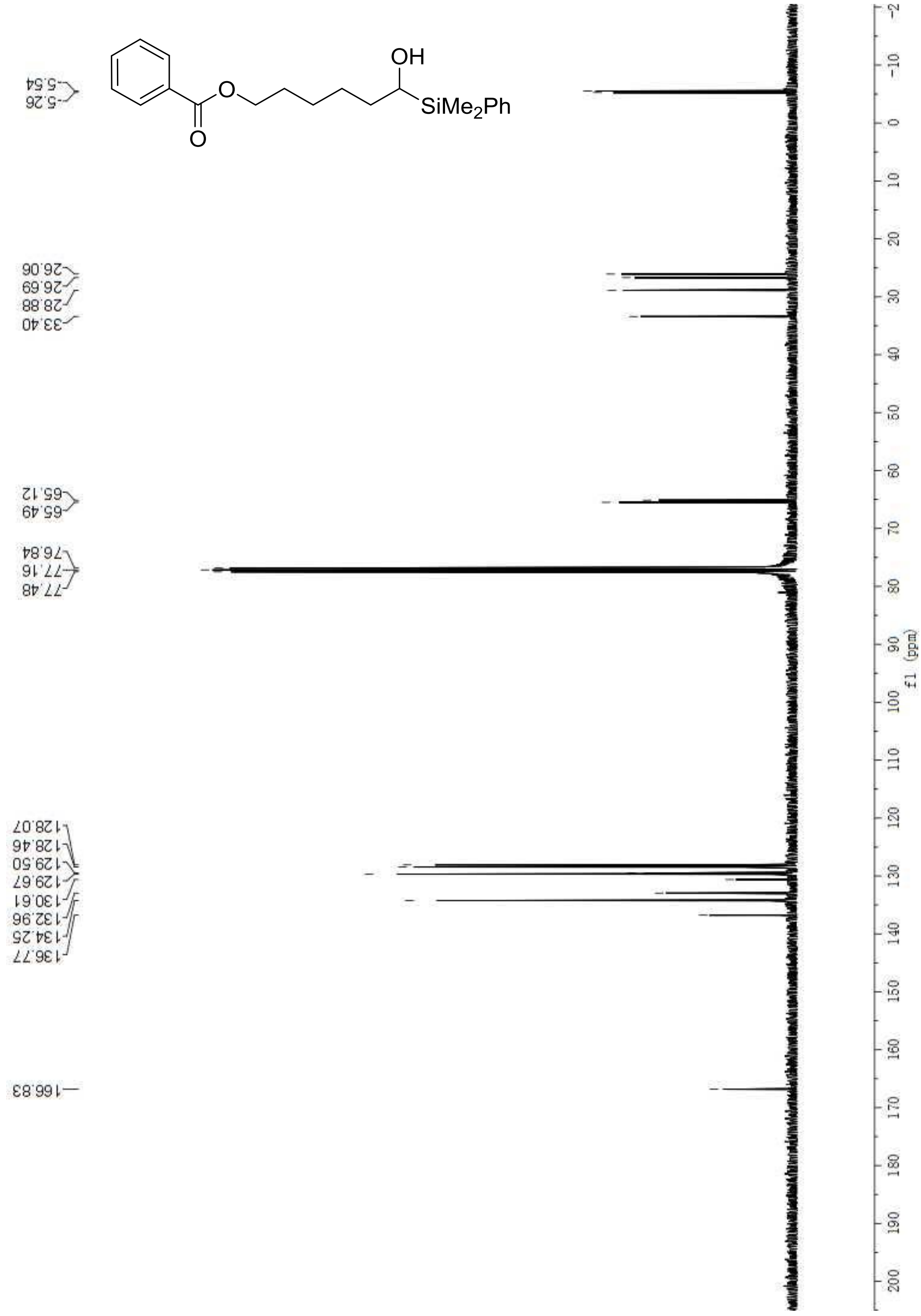

6-(benzyloxy)-1-(dimethyl(phenyl)silyl)hexan-1-ol (1r)

^{1}H NMR of **1r** ($CDCl_3$, 400 MHz, 25 ºC)

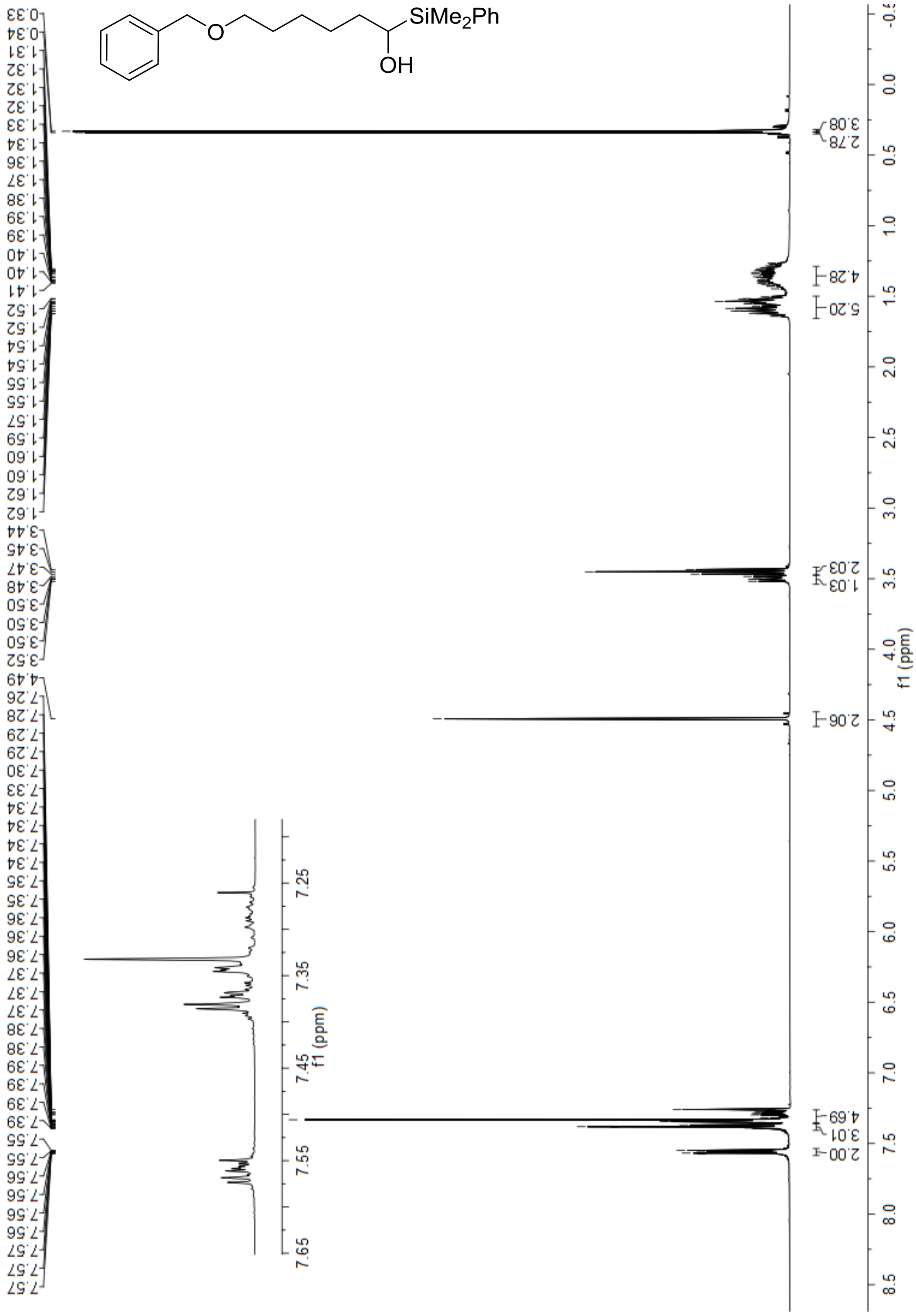

^{13}C NMR of **1r** ($CDCl_3$, 101 MHz, 25 ºC)

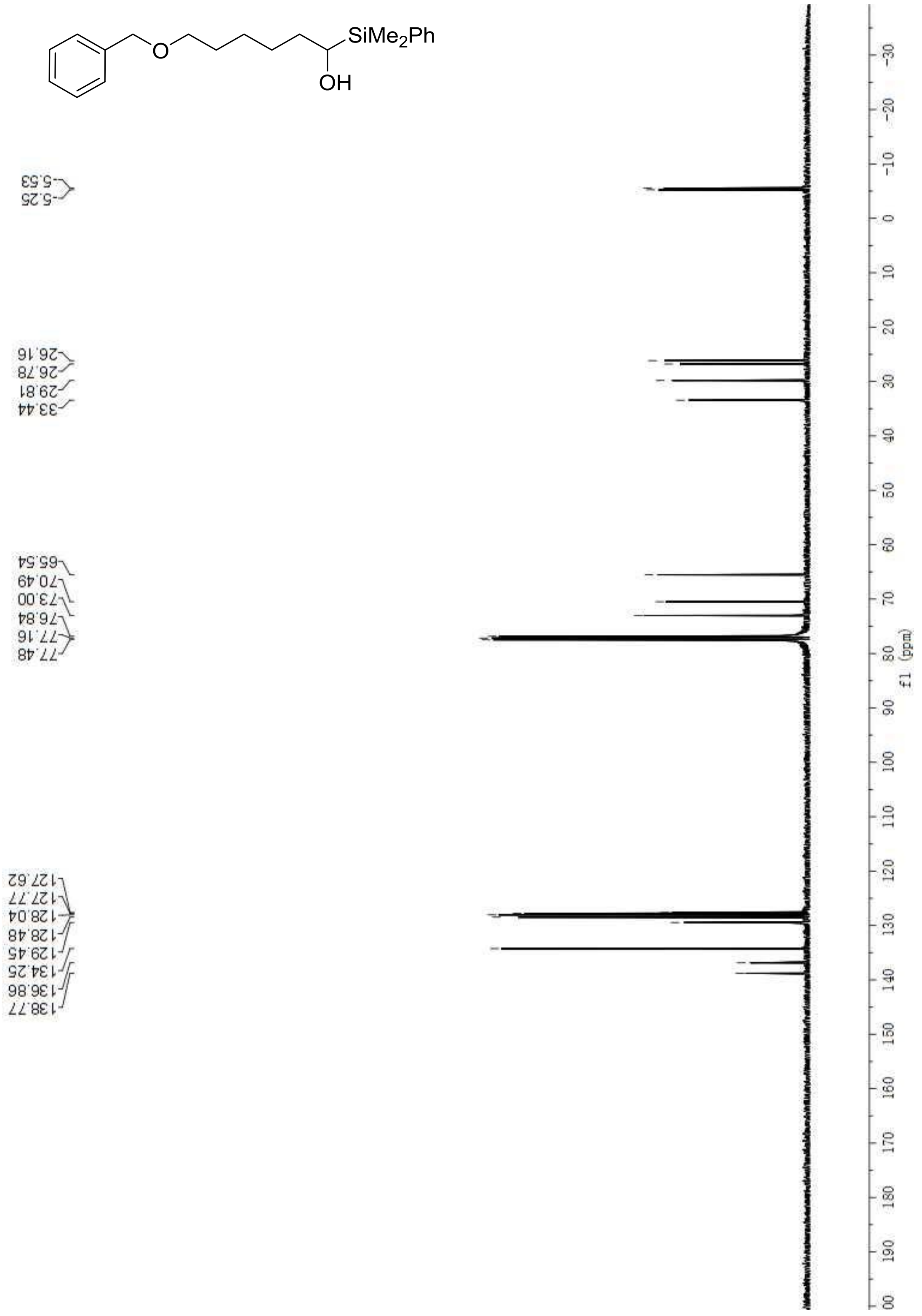

1-(dimethyl(phenyl)silyl)-4-(3-phenylpropoxy)butan-1-ol (1s)

^{1}H NMR of **1s** ($CDCl_3$, 400 MHz, 25 ºC)

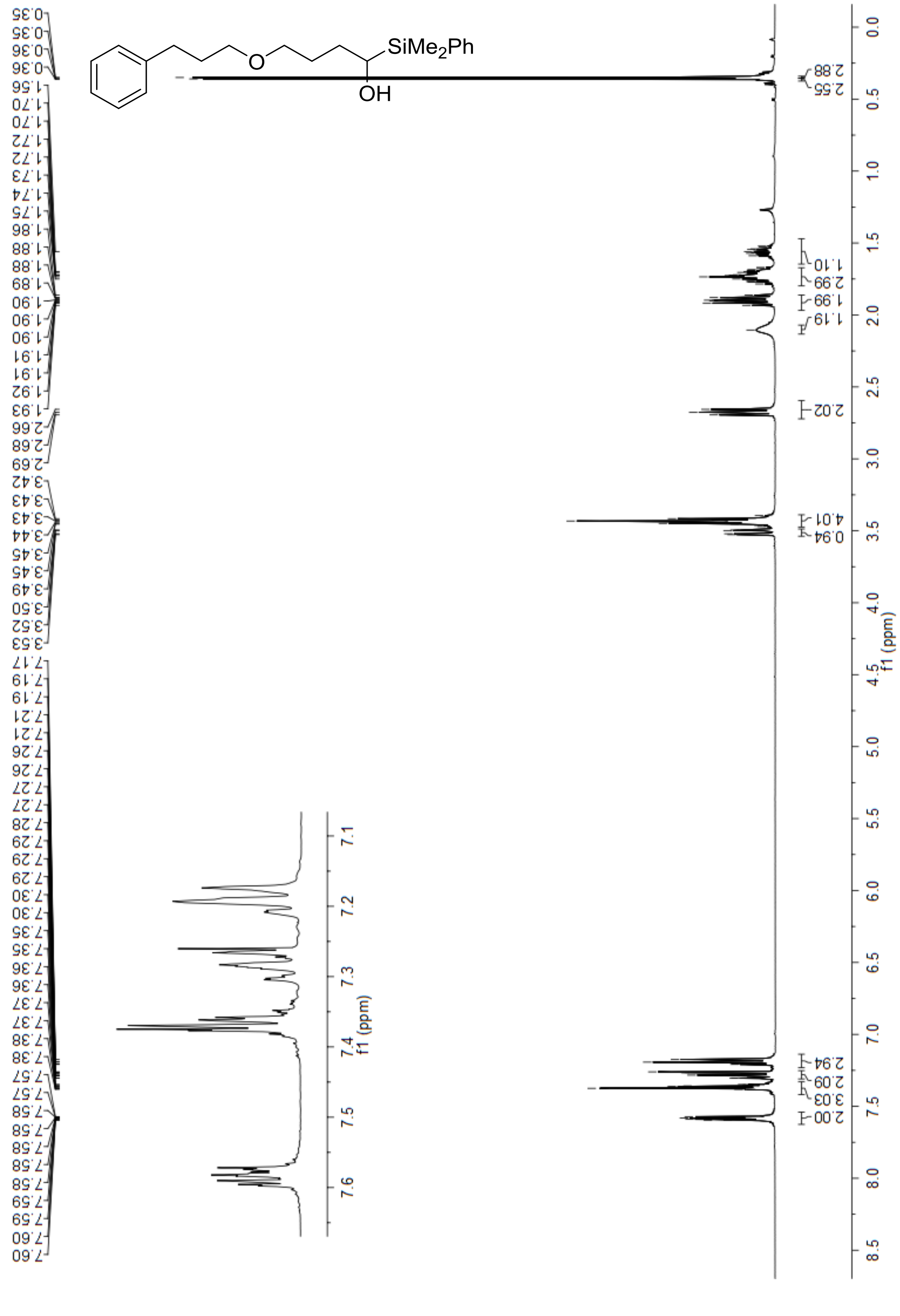

^{13}C NMR of **1s** ($CDCl_3$, 101 MHz, 25 ºC)

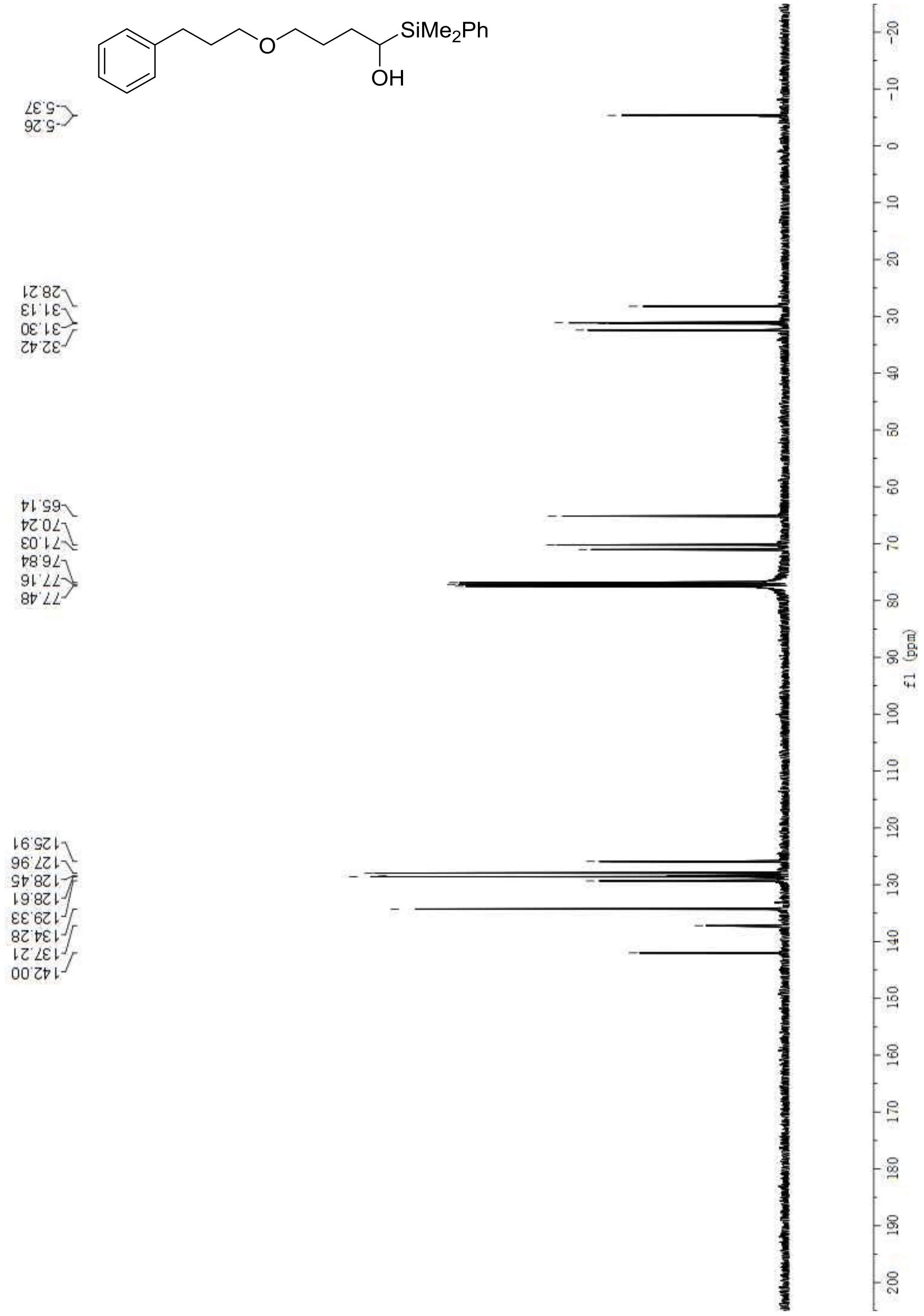

1-(dimethyl(phenyl)silyl)-6-((4-methylbenzyl)oxy)hexan-1-ol (1t)

^{1}H NMR of **1t** (CDCl$_3$, 400 MHz, 25 ºC)

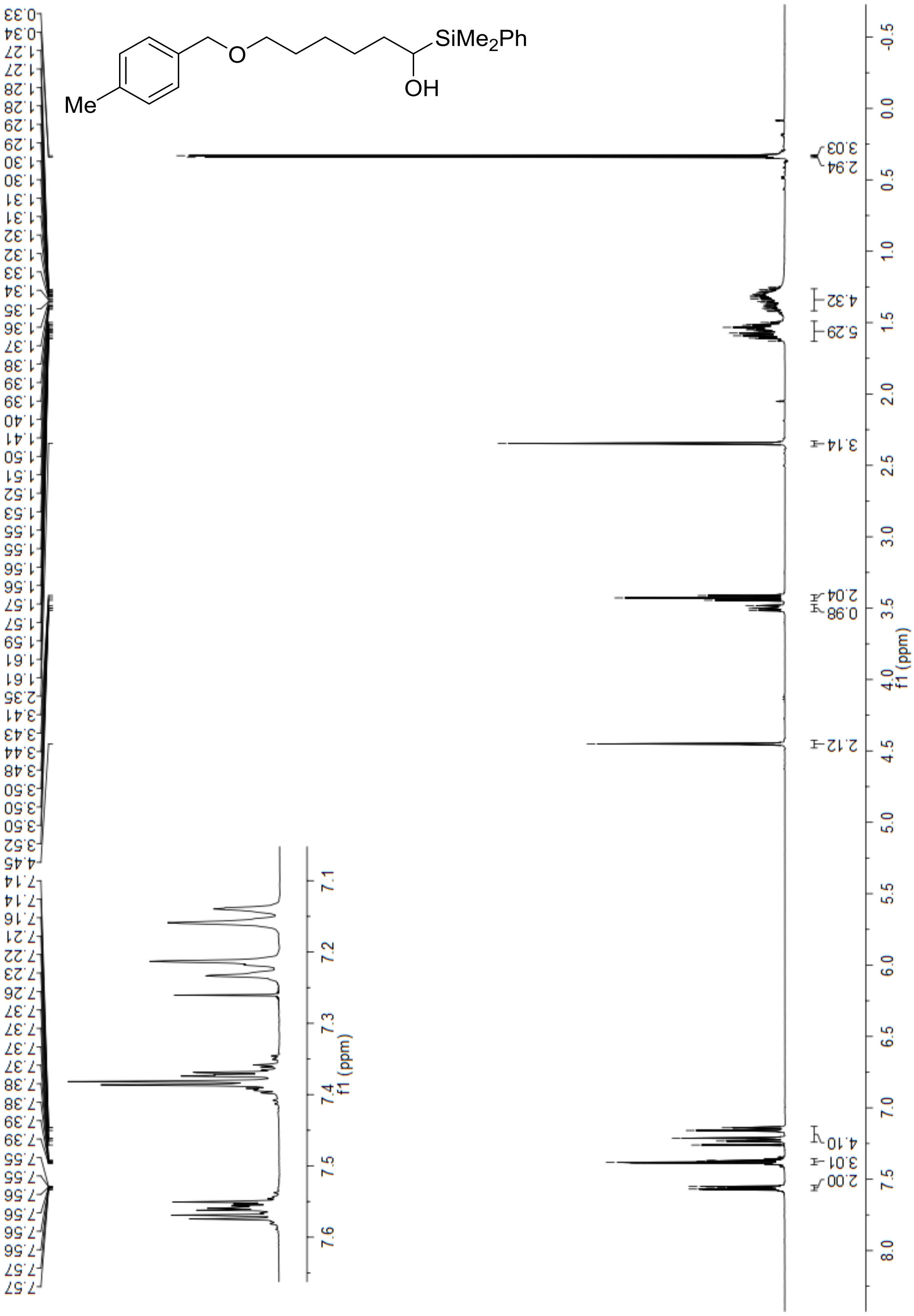

^{13}C NMR of **1t** ($CDCl_3$, 101 MHz, 25 ºC)

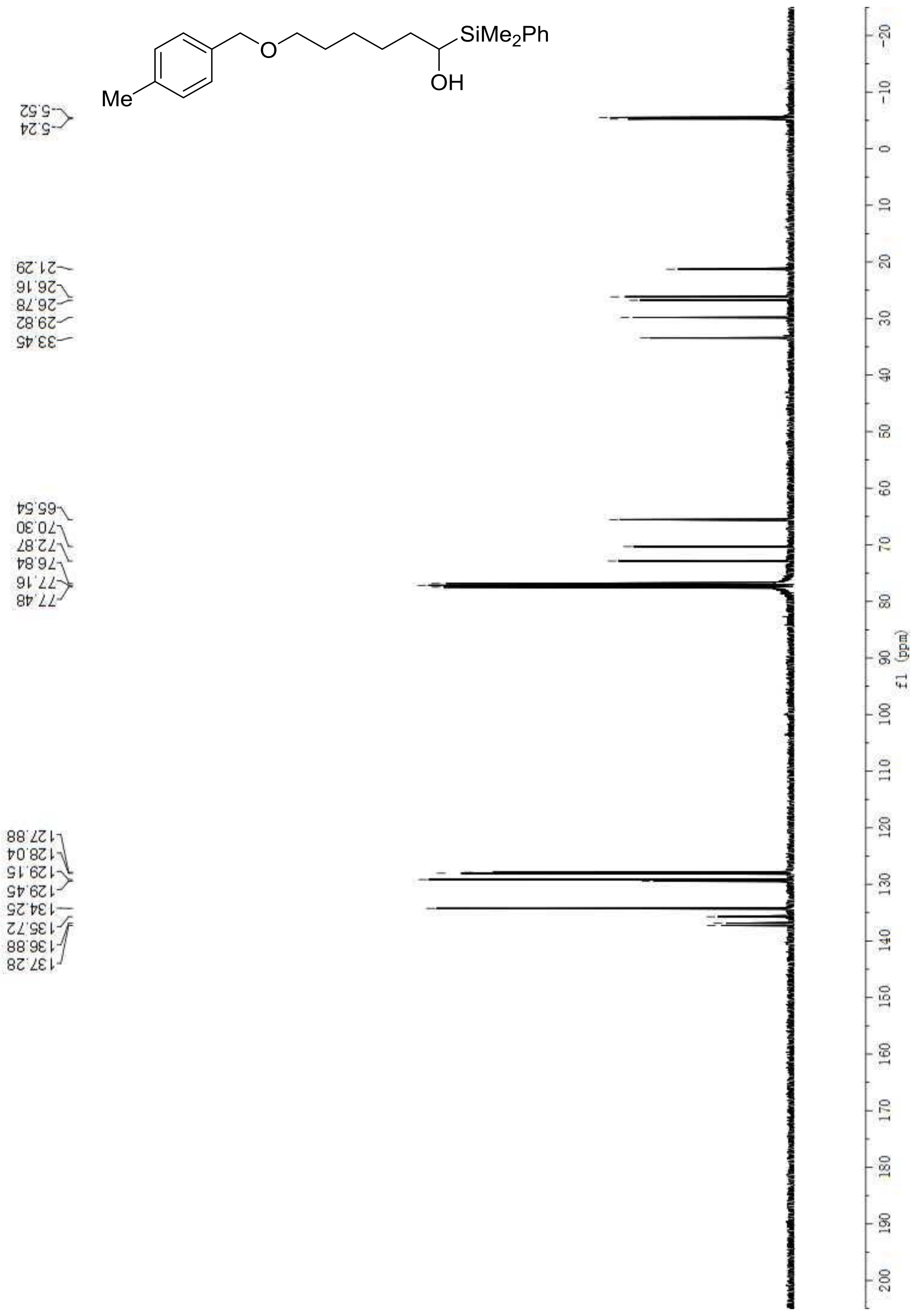

5-(benzyloxy)-1-(dimethyl(phenyl)silyl)pentan-1-ol (1u)

^{1}H NMR of **1u** ($CDCl_3$, 400 MHz, 25 ºC)

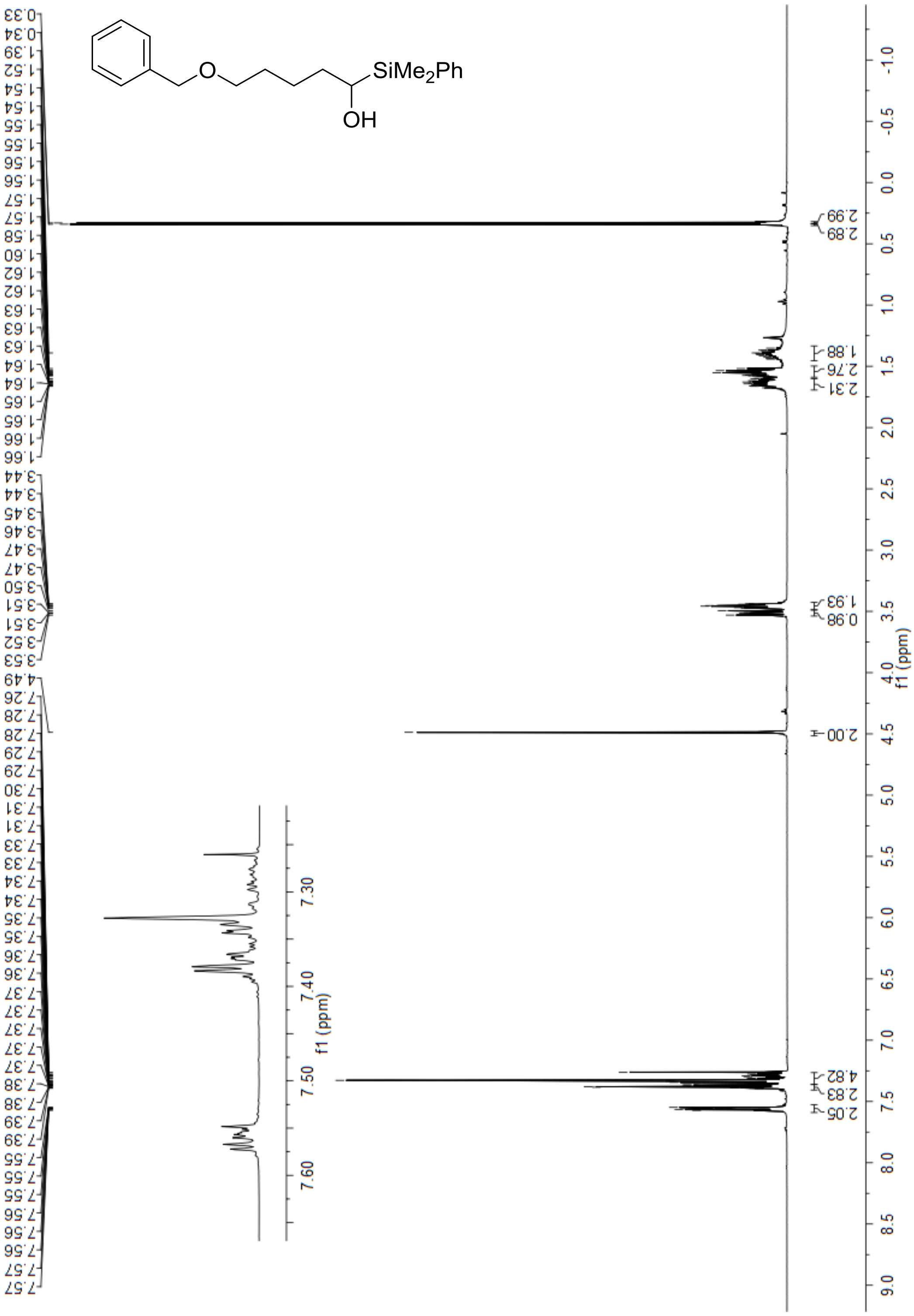

^{13}C NMR of **1u** ($CDCl_3$, 101 MHz, 25 ºC)

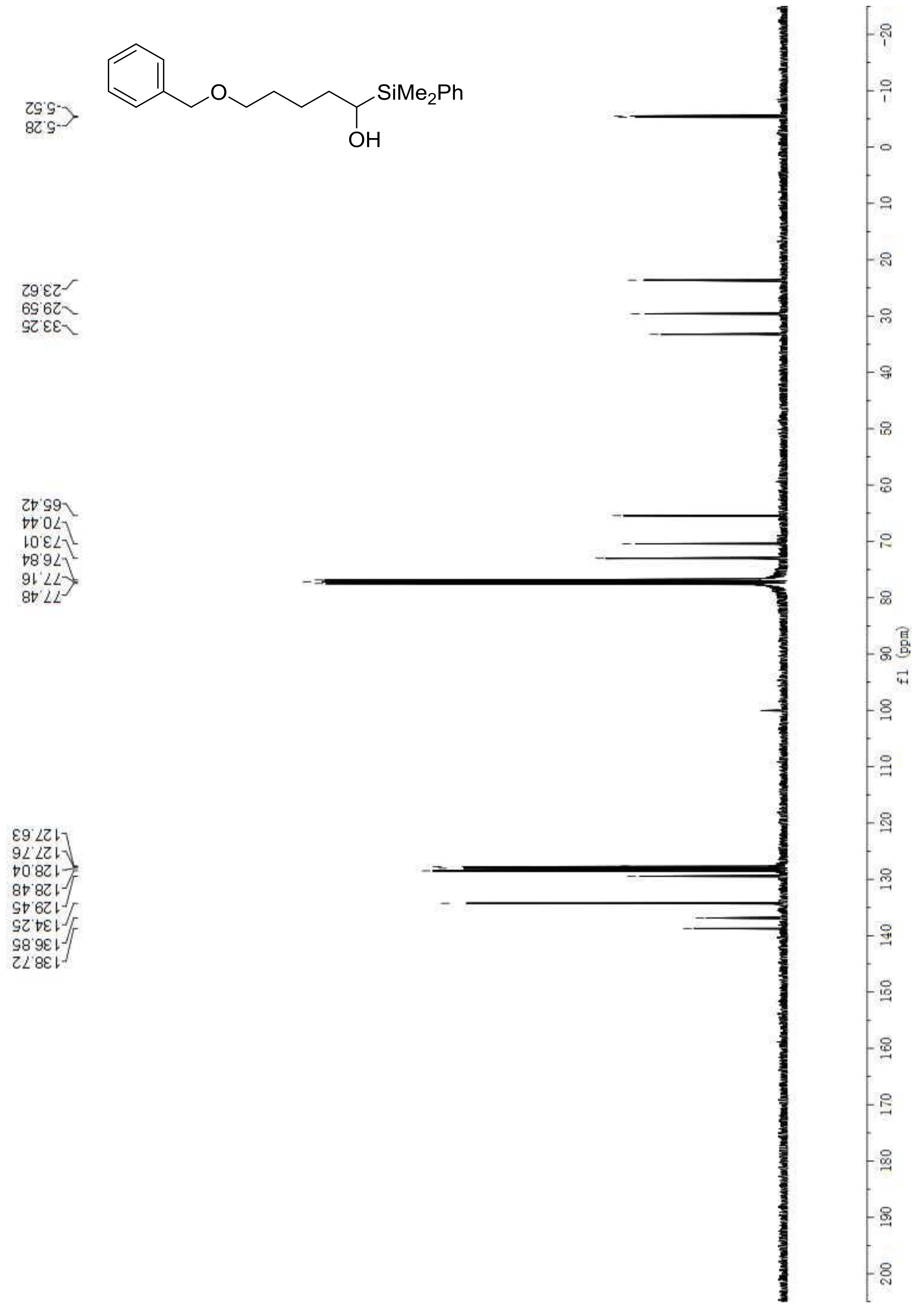

1-(dimethyl(phenyl)silyl)-4-(isopentyloxy)butan-1-ol (1v)

^{1}H NMR of **1y** ($CDCl_3$, 400 MHz, 25 ºC)2

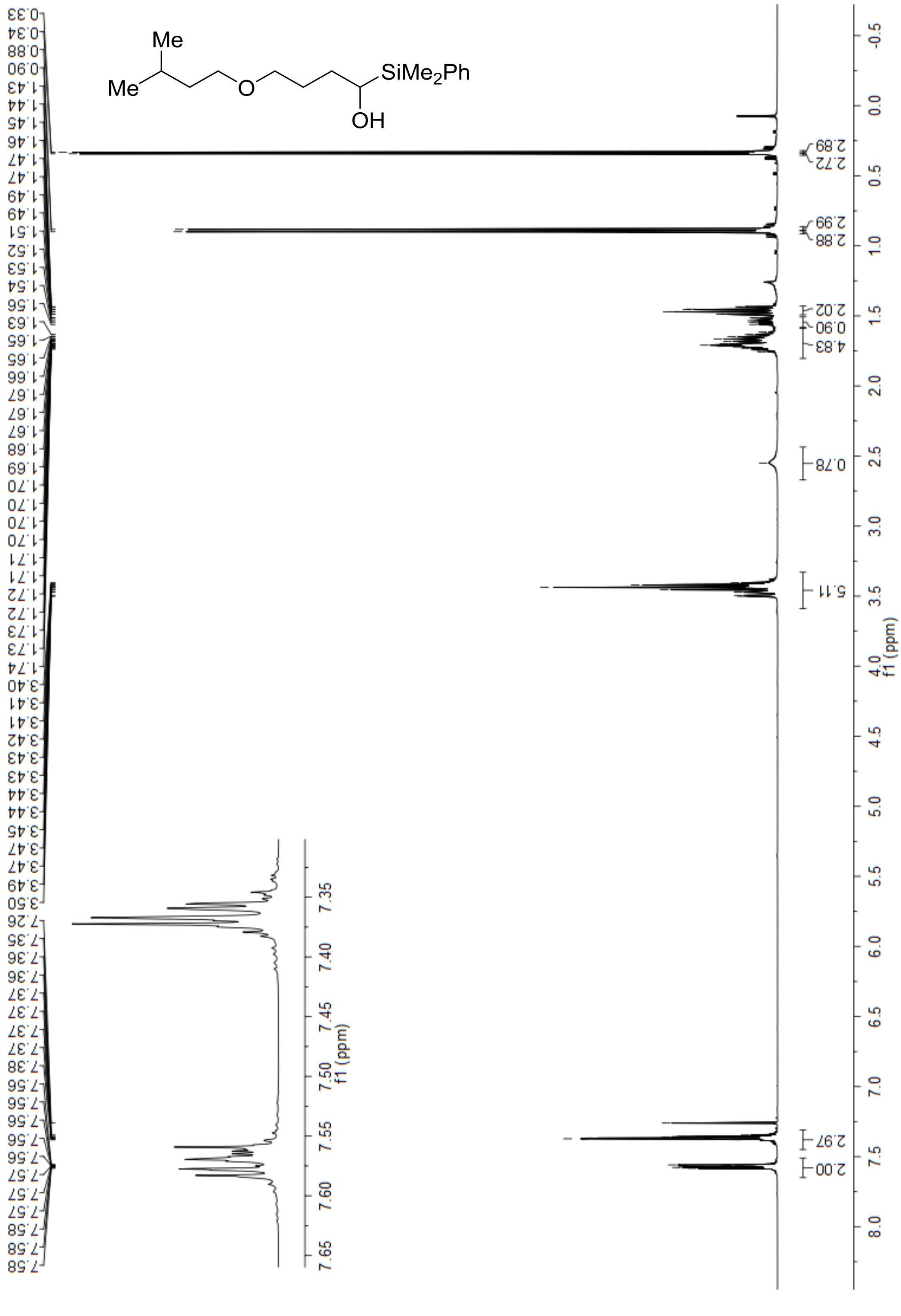

^{13}C NMR of **1u** ($CDCl_3$, 400 MHz, 25 ºC)

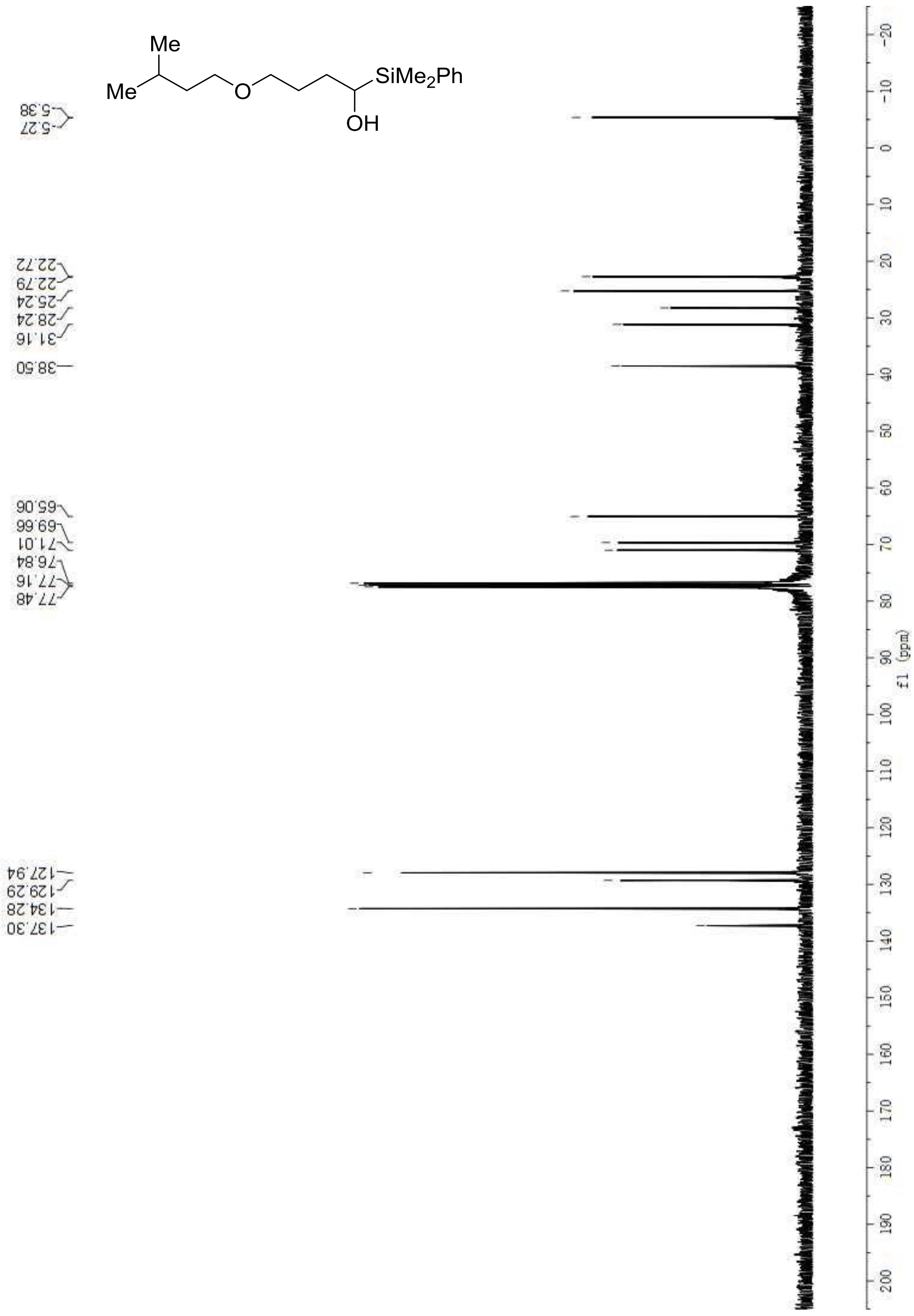

1-(dimethyl(phenyl)silyl)-6-((4-(trifluoromethoxy)benzyl)oxy)hexan-1-ol (1w)

^{1}H NMR of **1w** ($CDCl_3$, 400 MHz, 25 ºC)

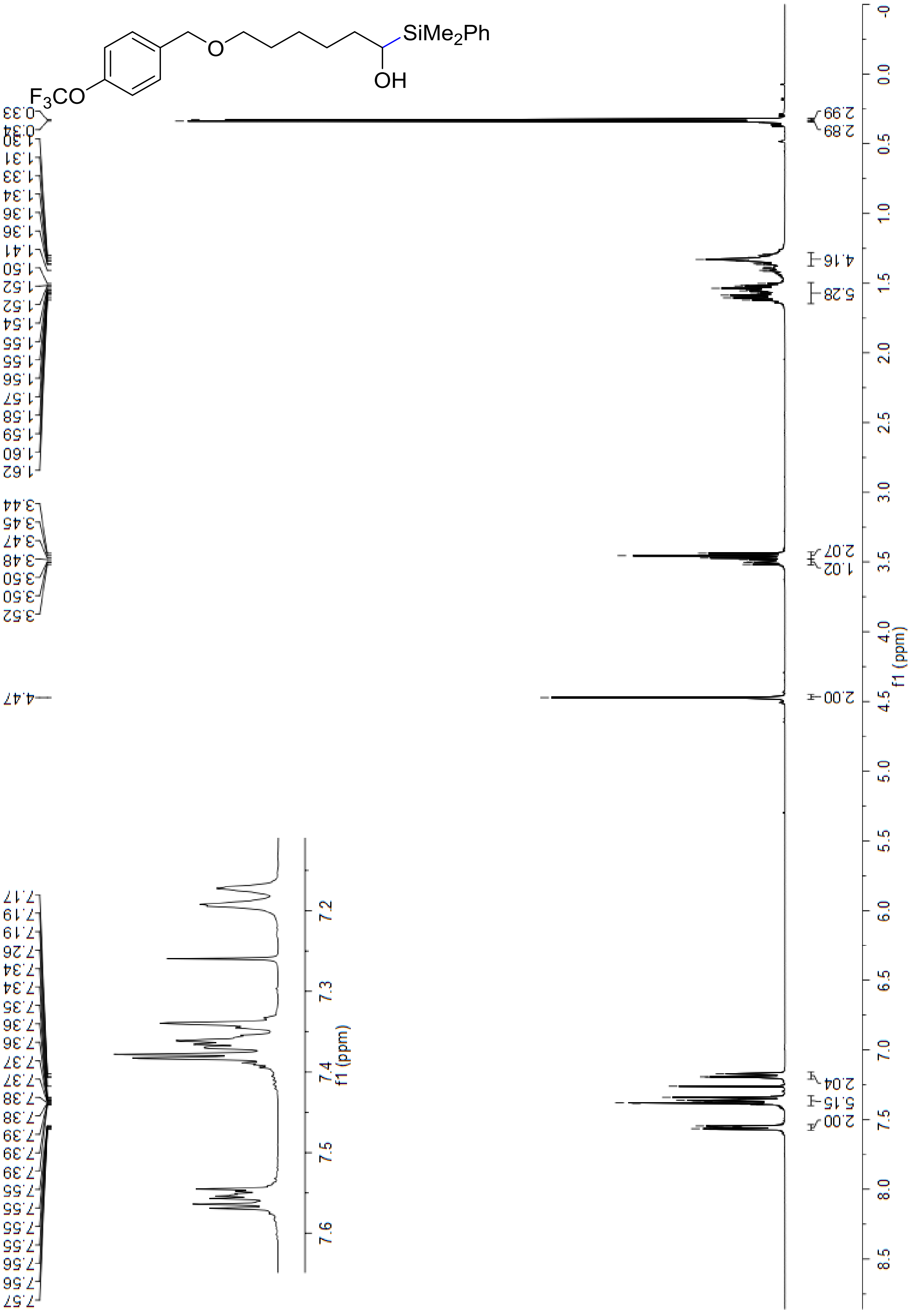

^{13}C NMR of **1w** ($CDCl_3$, 101 MHz, 25 ºC)

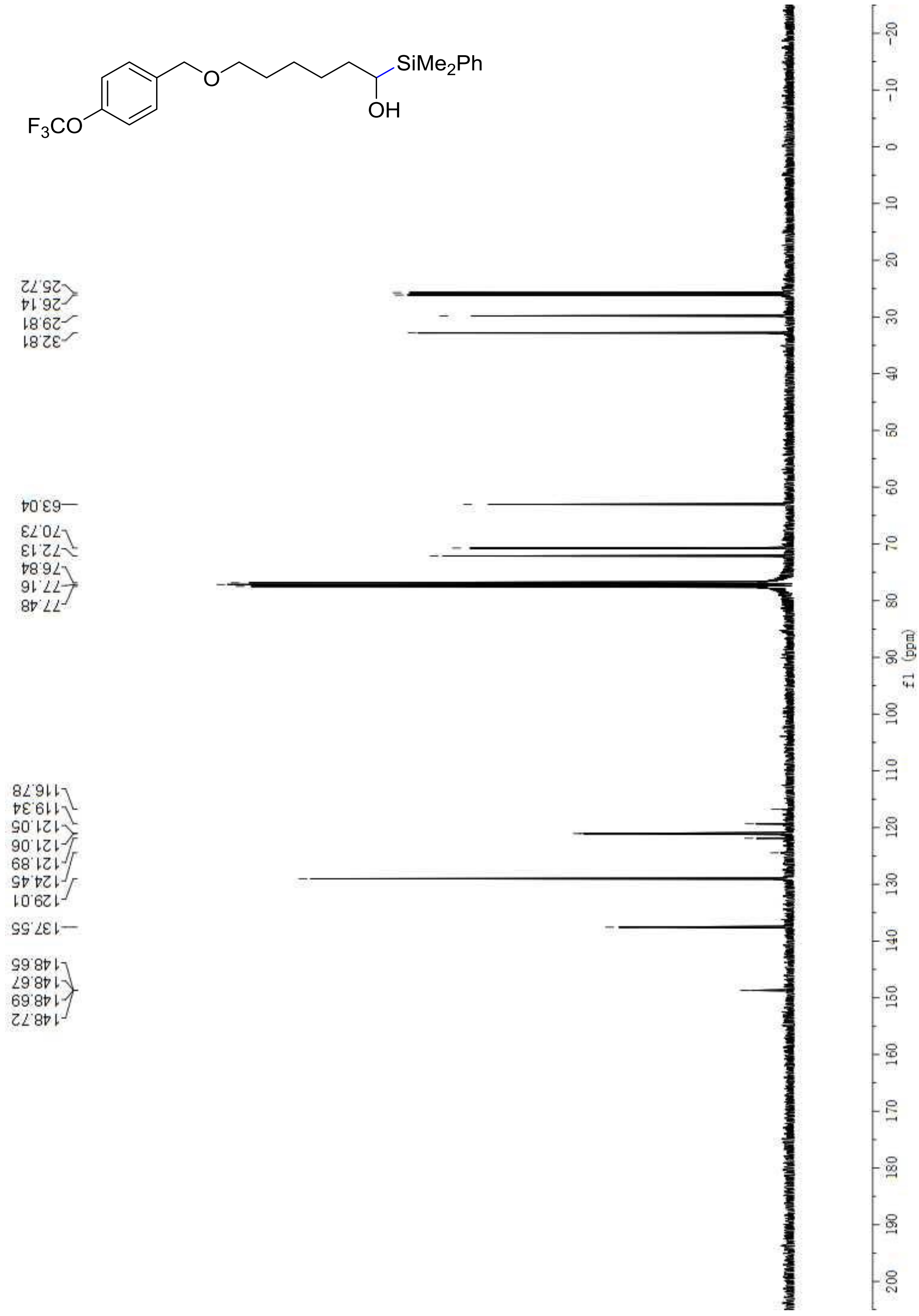

4-(2-(benzyloxy)ethoxy)-1-(dimethyl(phenyl)silyl)butan-1-ol (1x)

^{1}H NMR of **1x**($CDCl_3$, 400 MHz, 25 ºC)

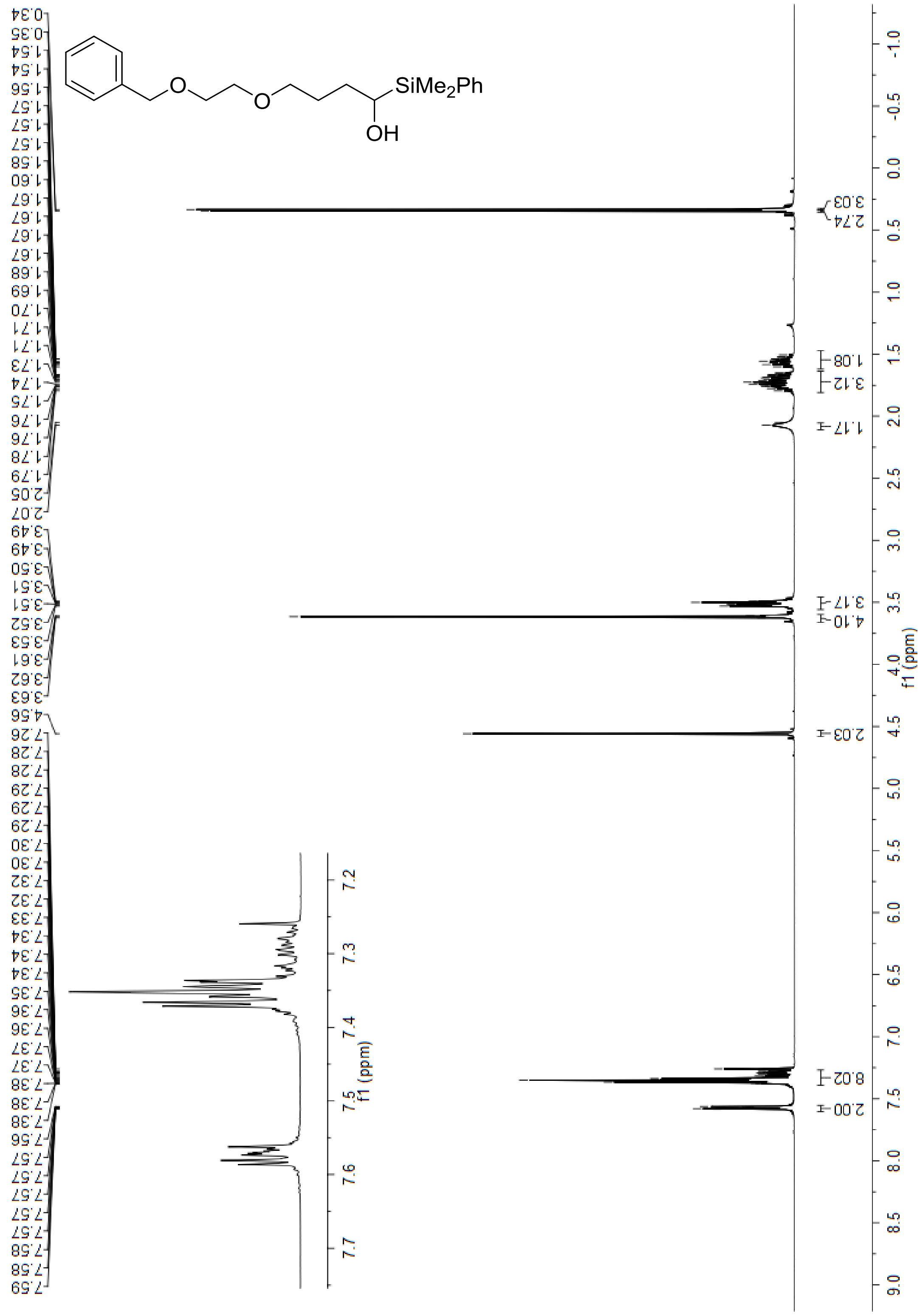

^{13}C NMR of **1x**($CDCl_3$, 101 MHz, 25 ºC)

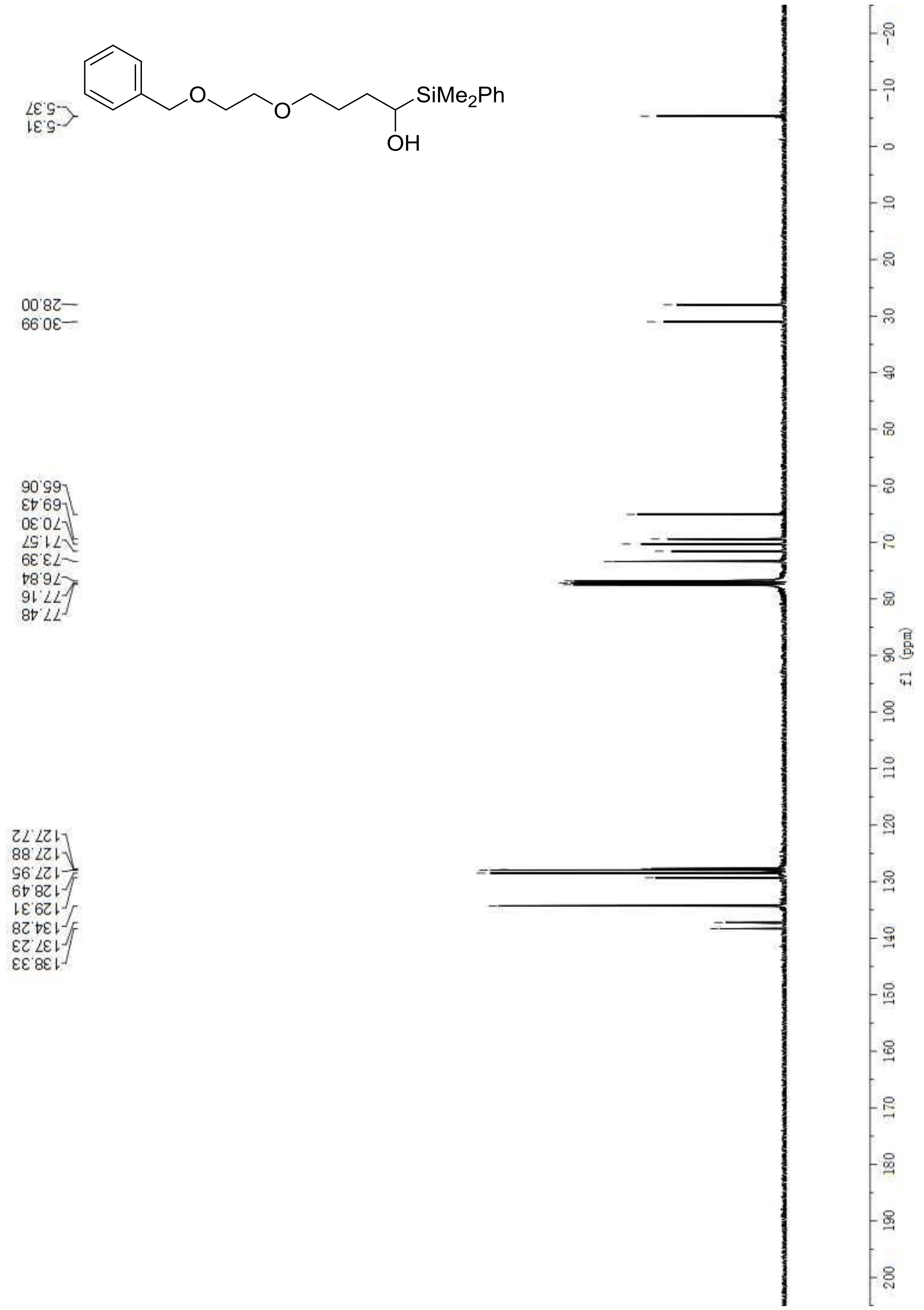

6-chloro-1-(dimethyl(phenyl)silyl)hexan-1-ol (1y)

^{1}H NMR of **1y**($CDCl_3$, 400 MHz, 25 ºC)

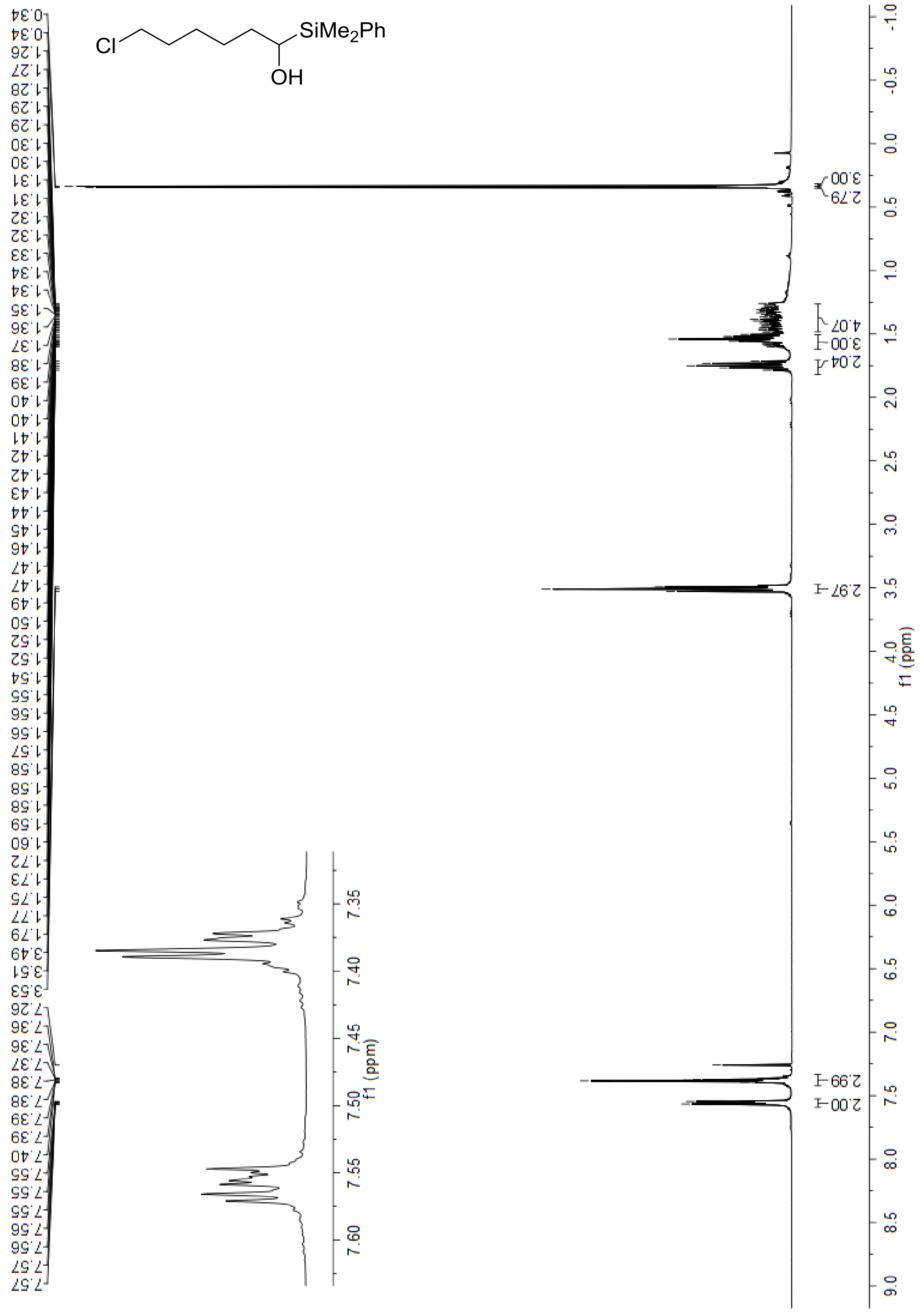

^{1}H NMR of **1y**($CDCl_3$, 101 MHz, 25 ºC)

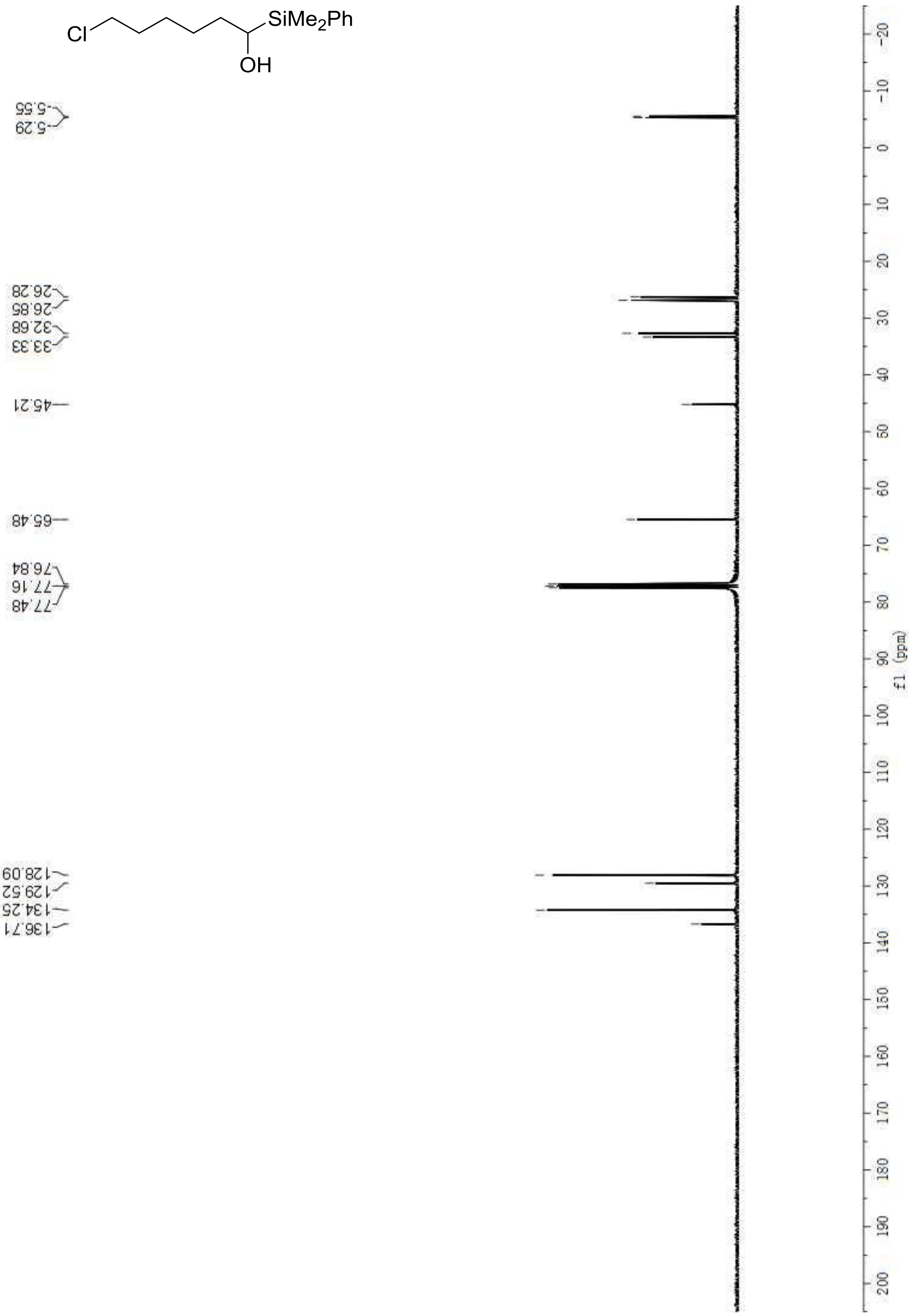

3-(Dimethyl(phenyl)silyl)heptan-3-ol (1z)

^{1}H NMR of **1z**($CDCl_3$, 400 MHz, 25 ºC)

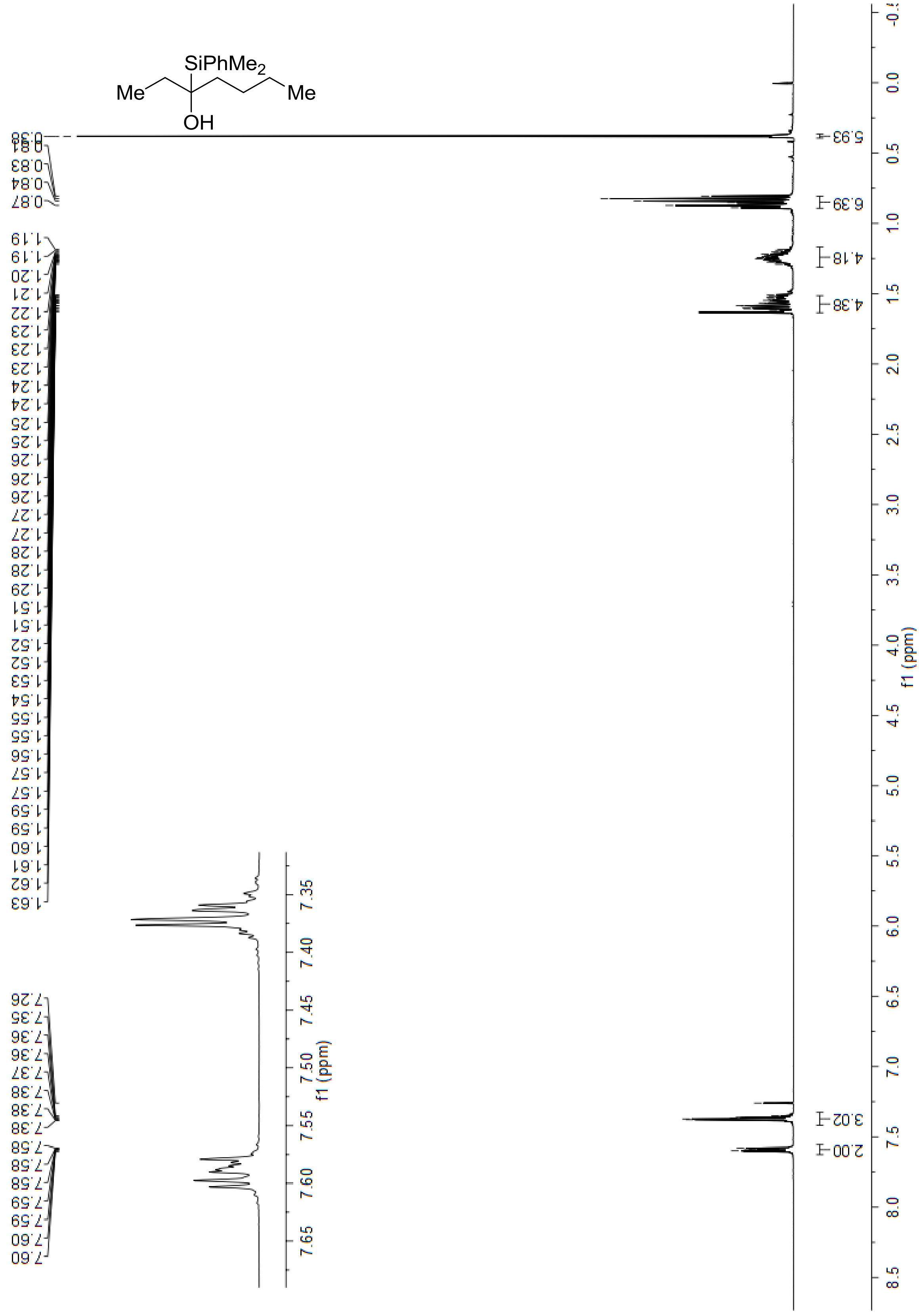

^{13}C NMR of **1x**($CDCl_3$, 101 MHz, 25 ºC)

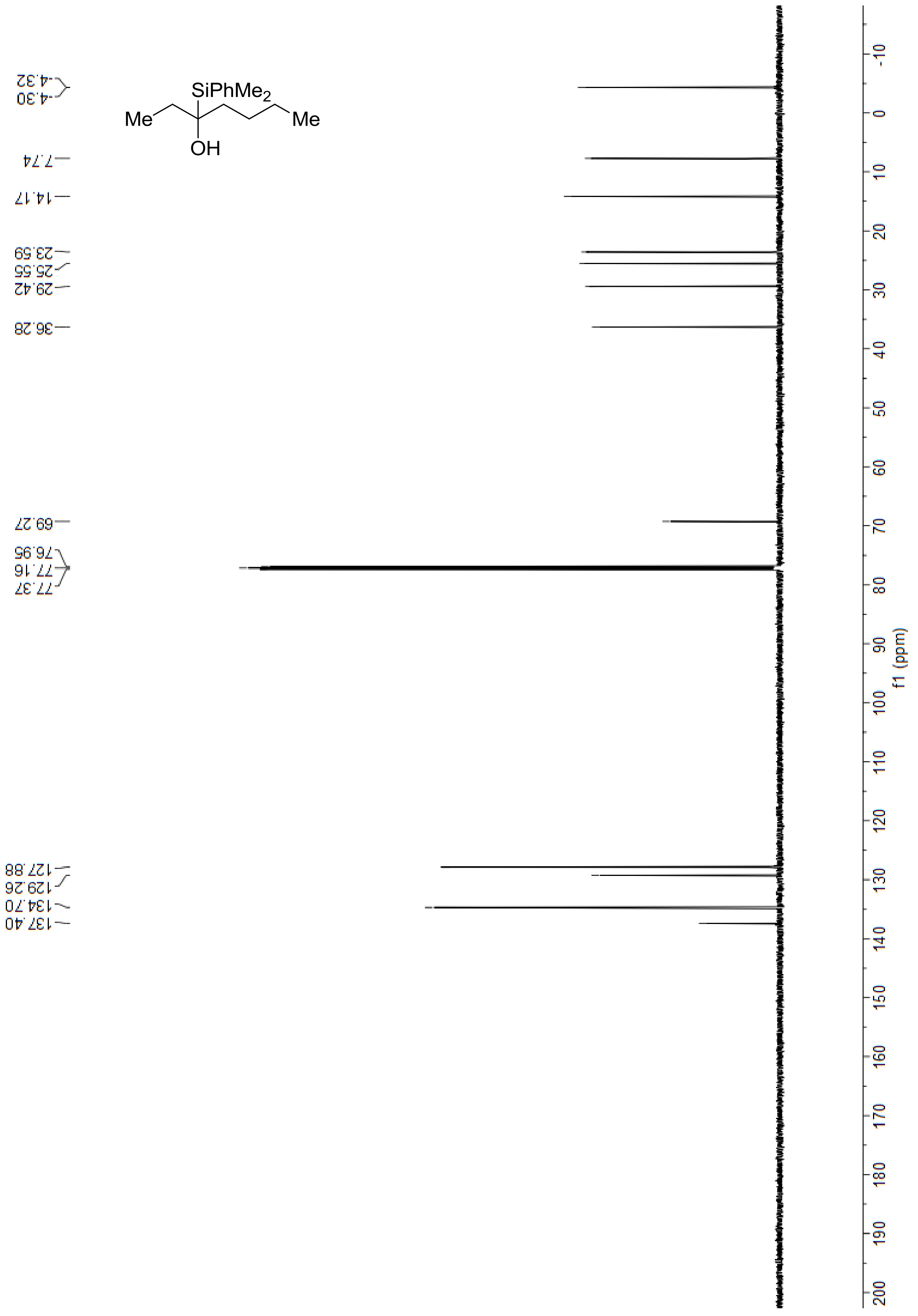

1-(Tert-butyldimethylsilyl)heptan-1-ol (1ac)

^{1}H NMR of **1ac**(CDCl$_3$, 600 MHz, 25 ºC)

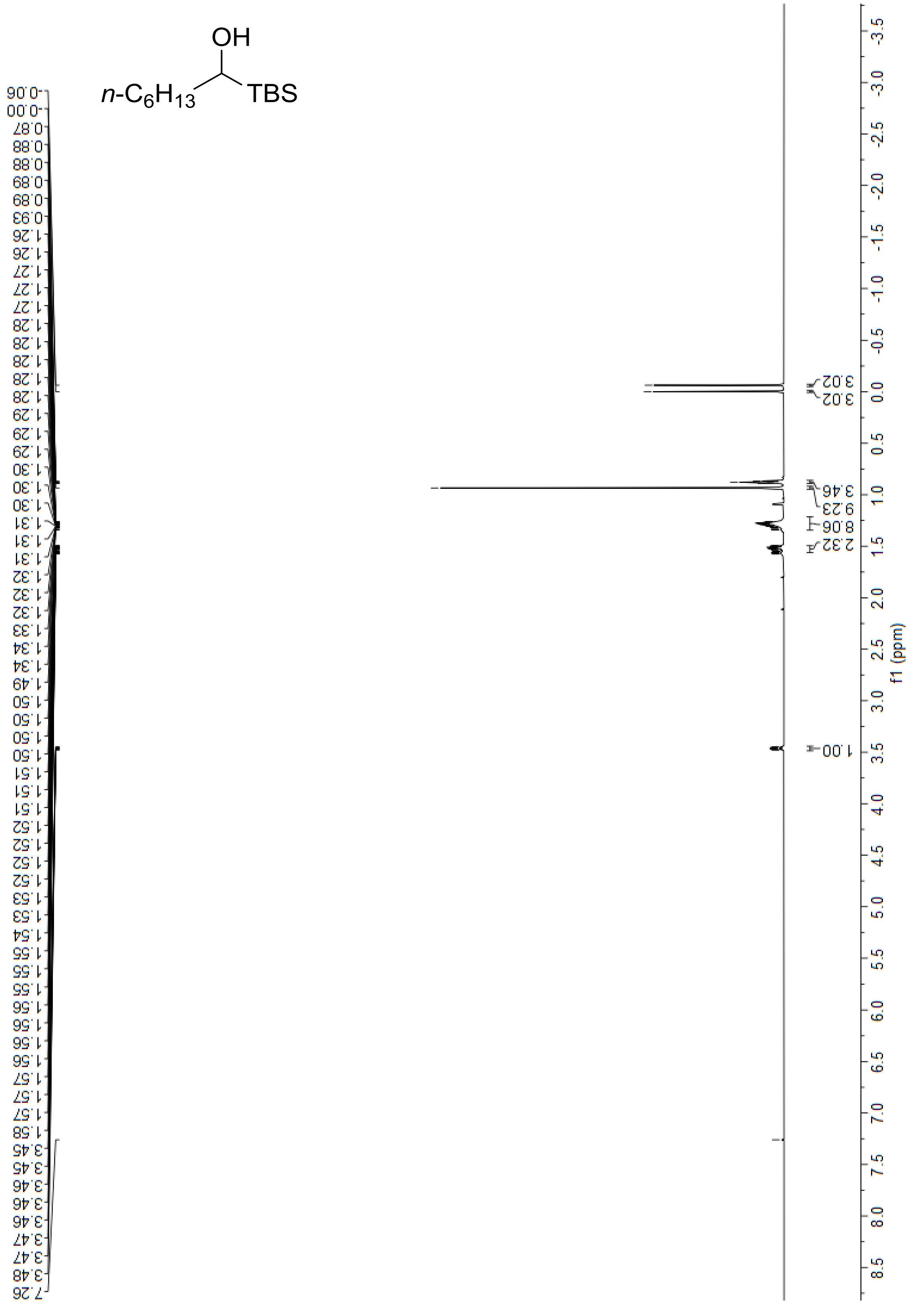

^{13}C NMR of **1ac**($CDCl_3$, 151 MHz, 25 ºC)

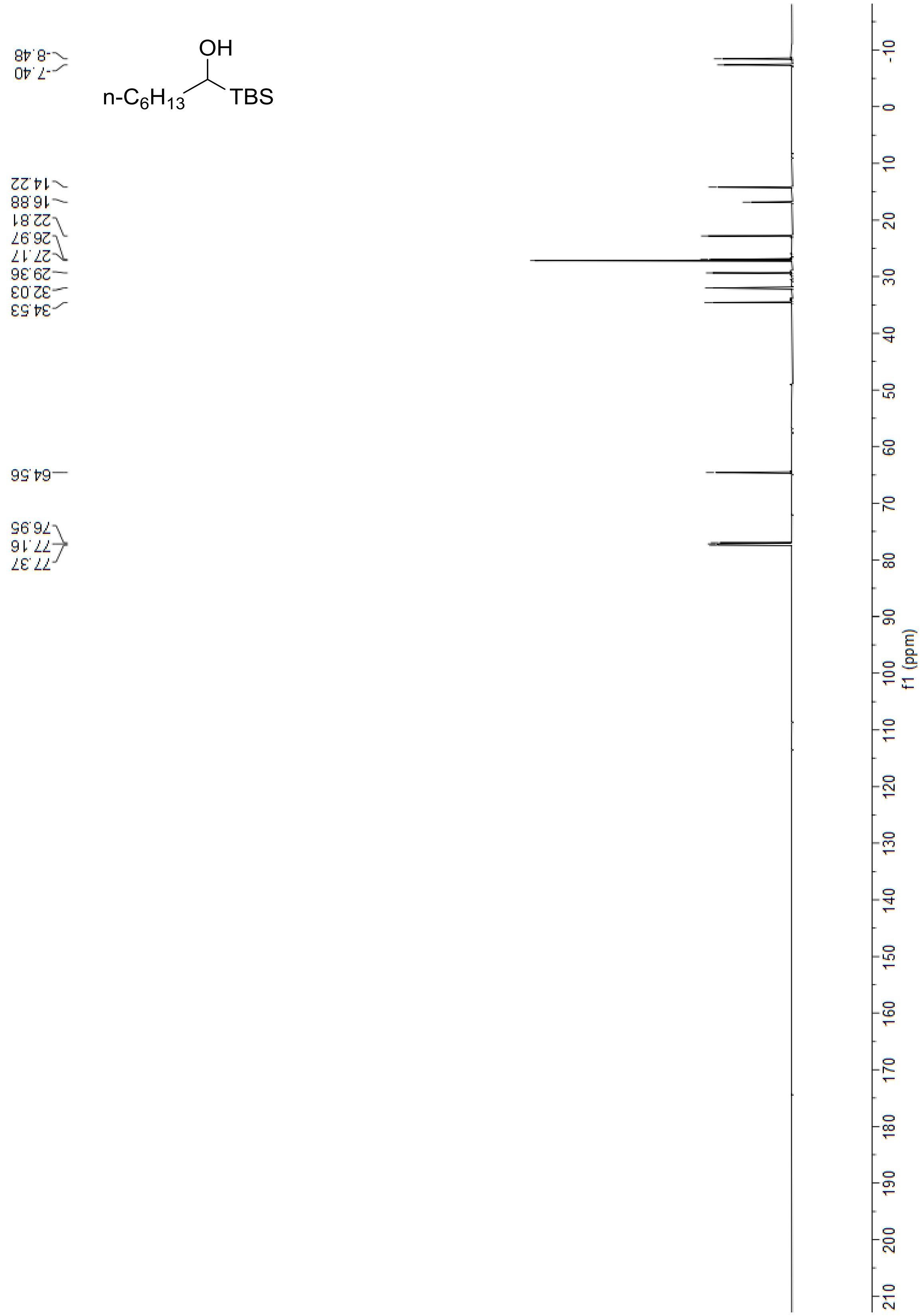

1-(triphenylsilyl)heptan-1-ol (1ae)

^{1}H NMR of **1ae** ($CDCl_3$, 400 MHz, 25 ºC)

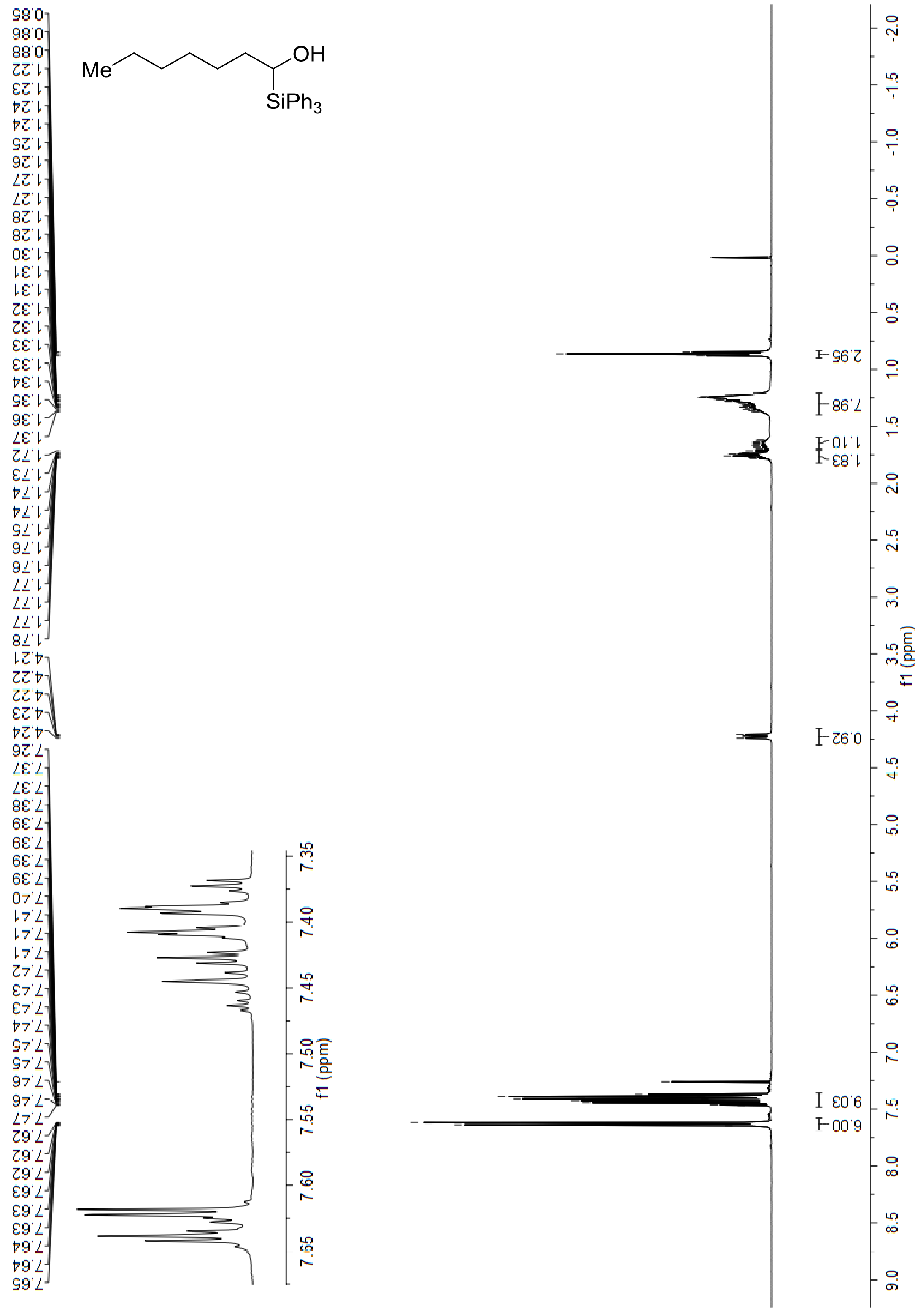

^{13}C NMR of **1ae** ($CDCl_3$, 101 MHz, 25 ºC)

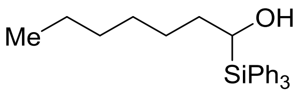

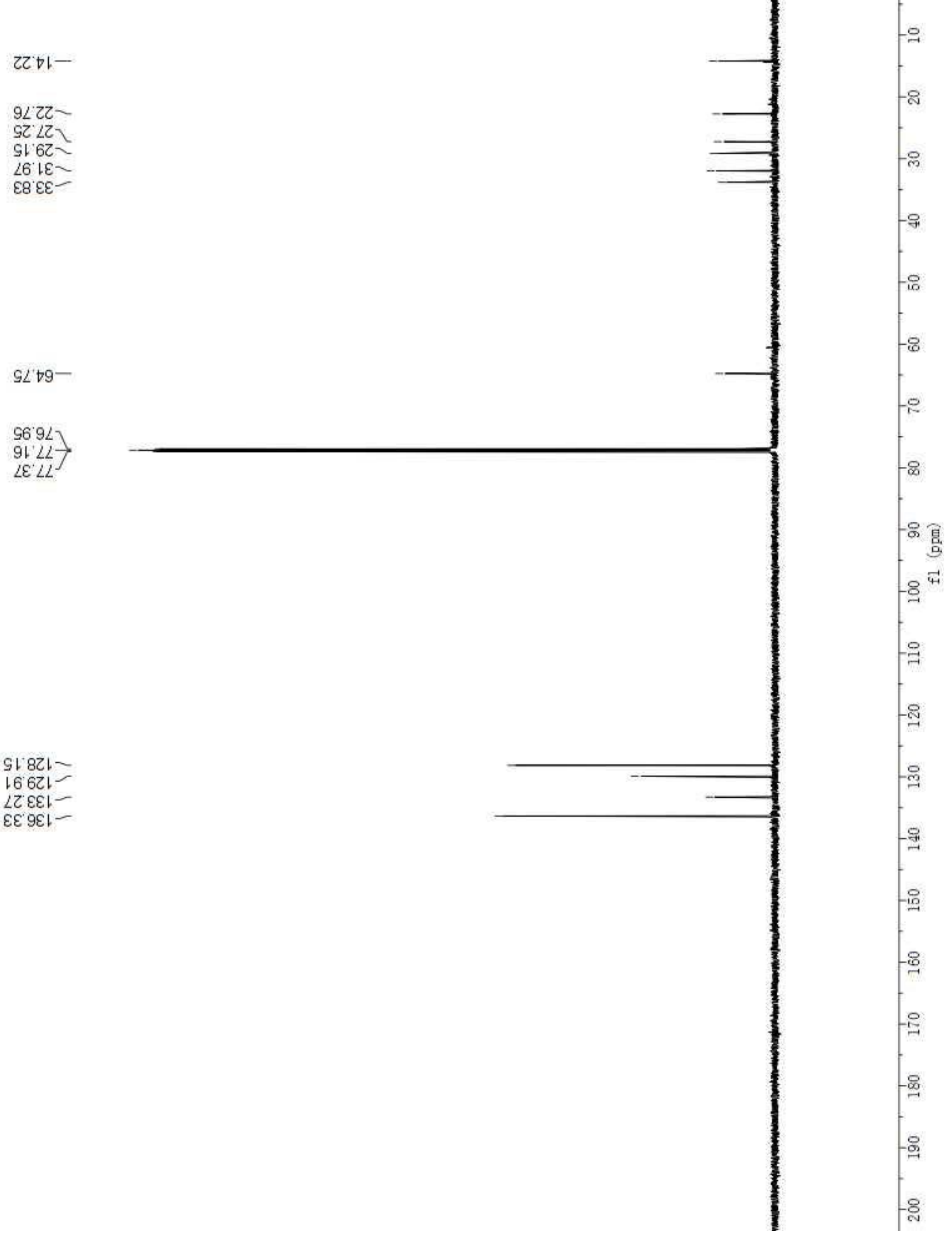

2-(dimethyl(phenyl)silyl)-5-methylhexan-2-ol(1af)

^{1}H NMR of **1af** ($CDCl_3$, 400 MHz, 25 ºC)

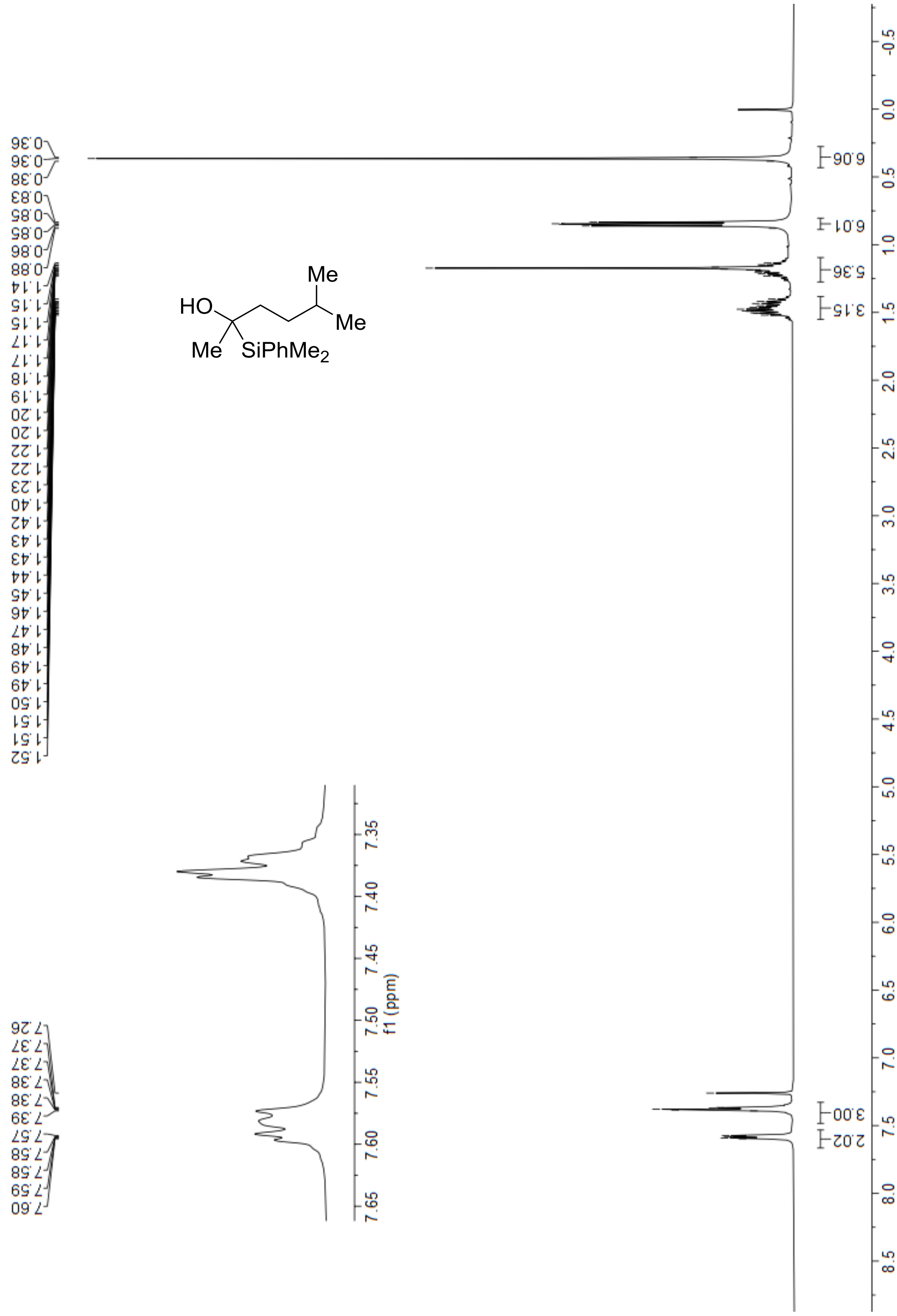

^{13}C NMR of **1af** ($CDCl_3$, 151 MHz, 25 ºC)

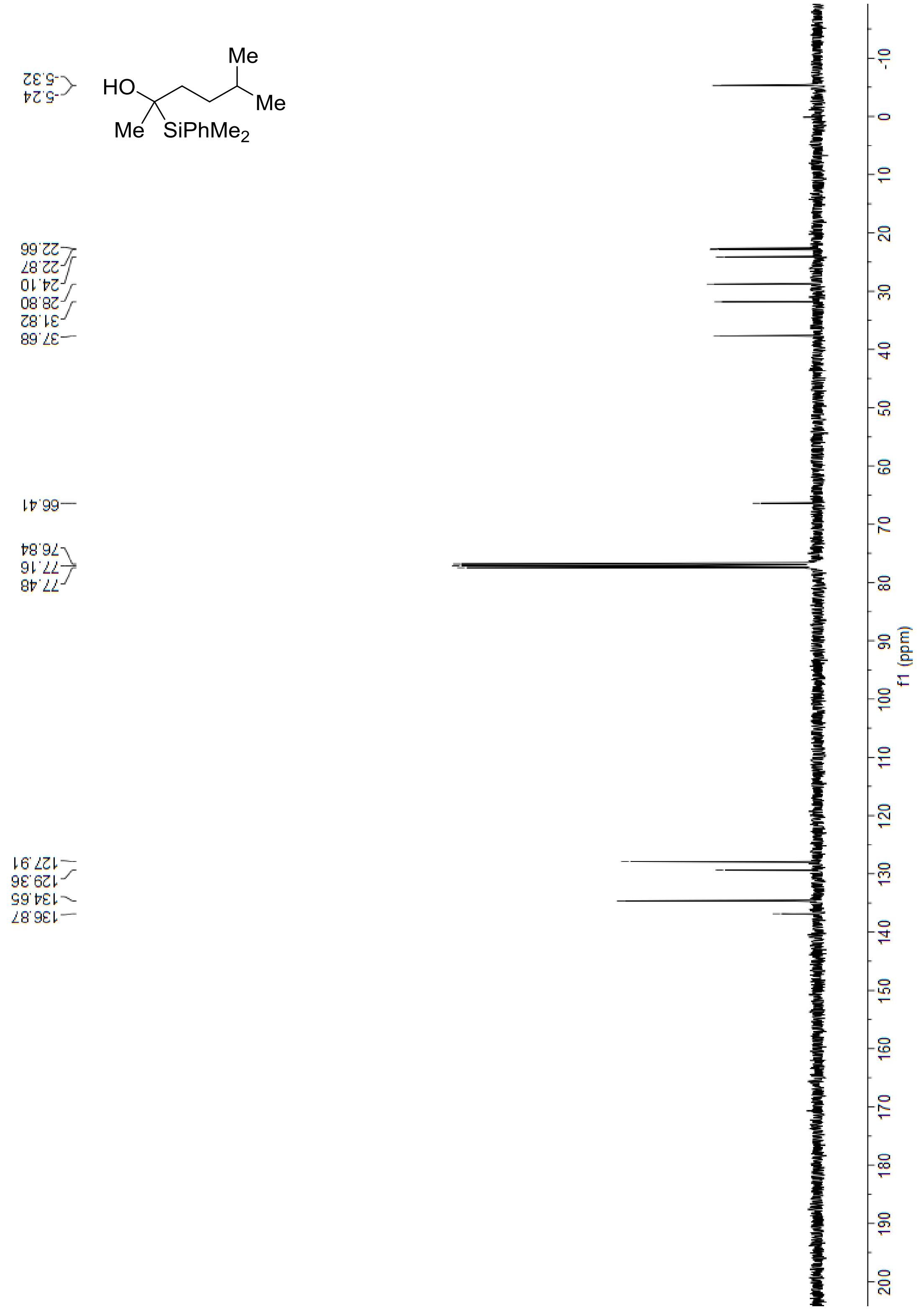

2-(dimethyl(phenyl)silyl)hexan-2-ol(1ag)

^{1}H NMR of **1ag** ($CDCl_3$, 400 MHz, 25 ºC)

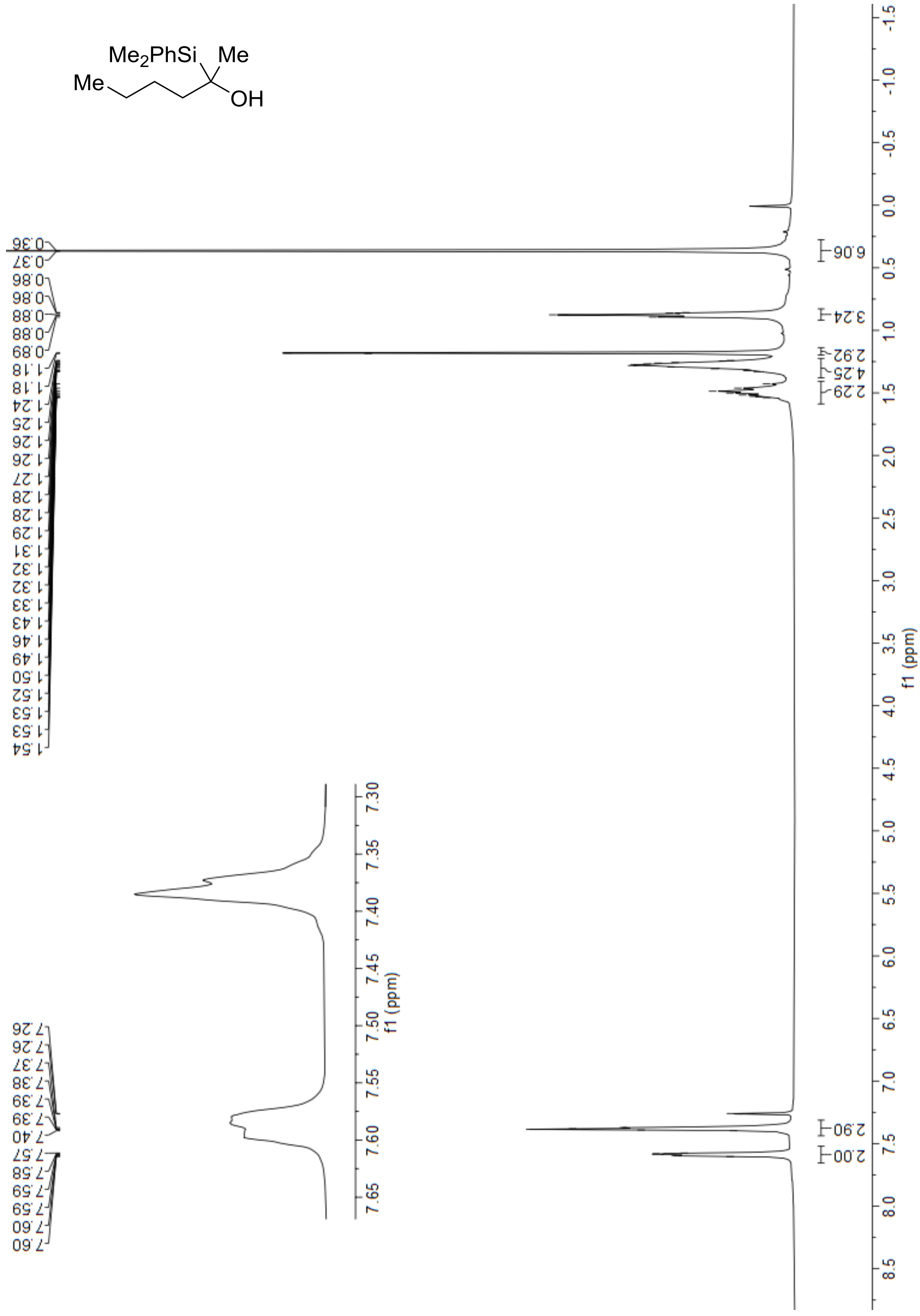

^{1}H NMR of **1ag** ($CDCl_3$, 151 MHz, 25 ºC)

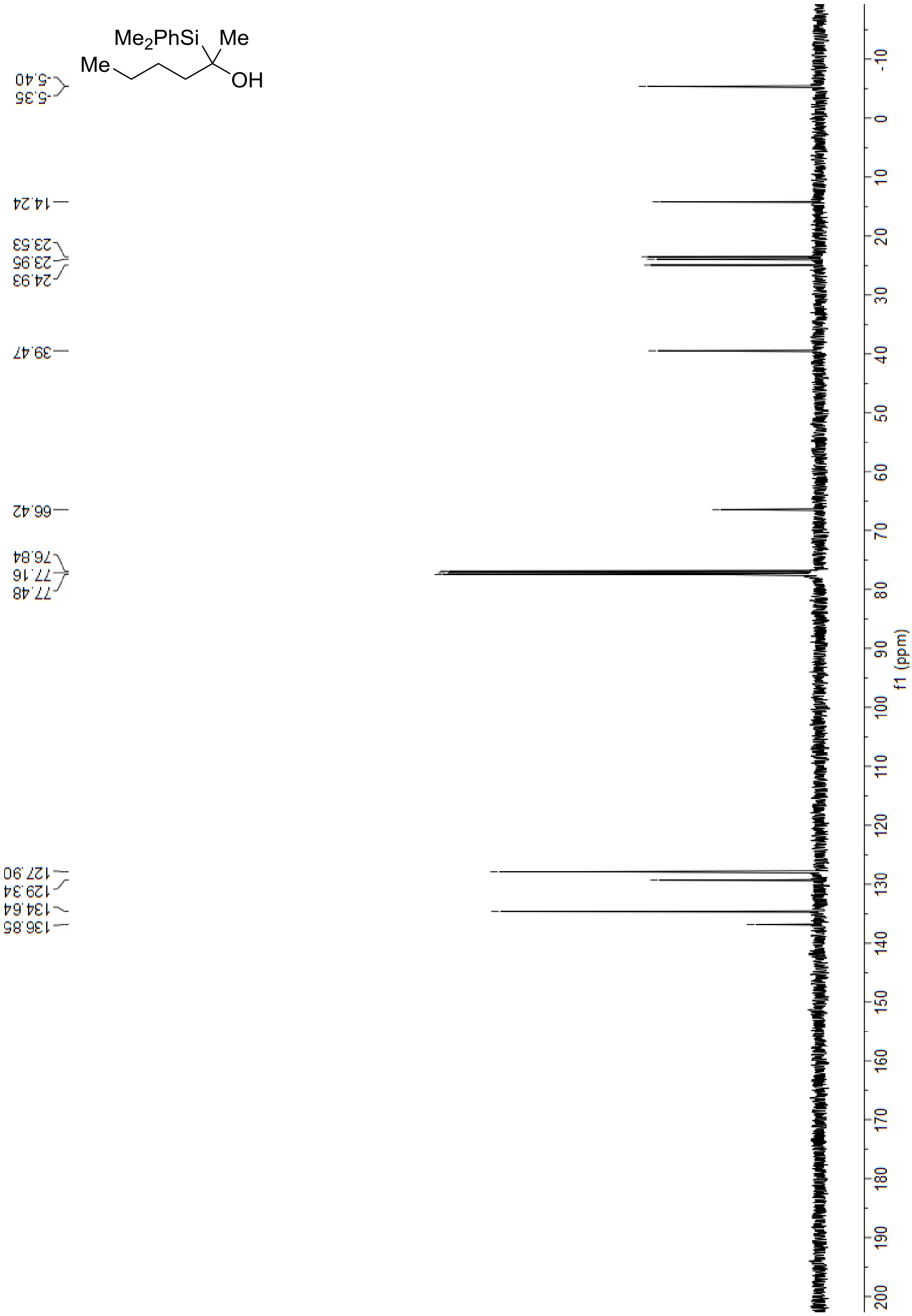

5-((tert-butyldimethylsilyl)oxy)-1-(dimethyl(phenyl)silyl)pentan-1-ol (1a-3)

^{1}H NMR of **1a-3** ($CDCl_3$, 151 MHz, 25 ºC)

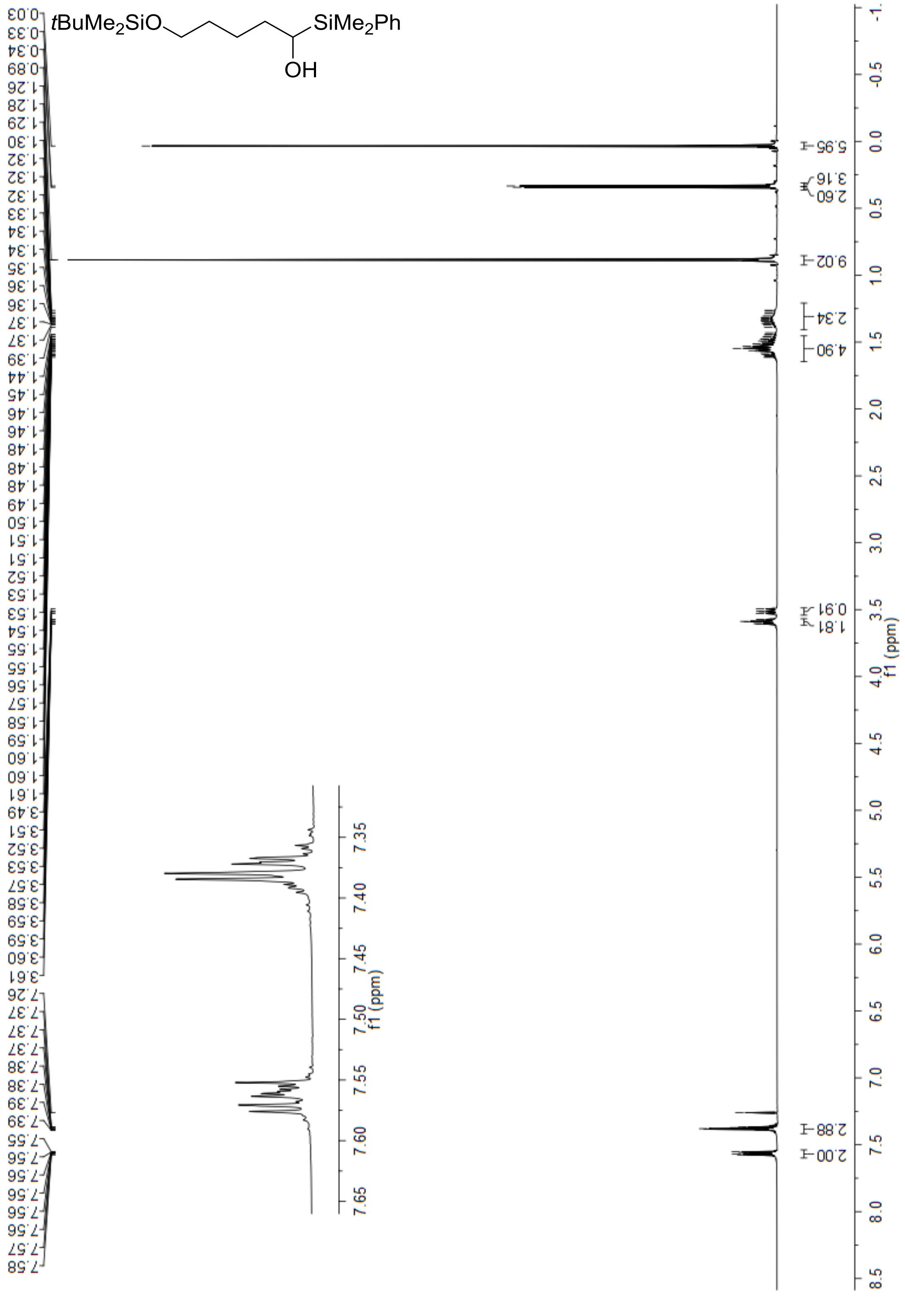

^{13}C NMR of **1a-3** ($CDCl_3$, 101 MHz, 25 ºC)

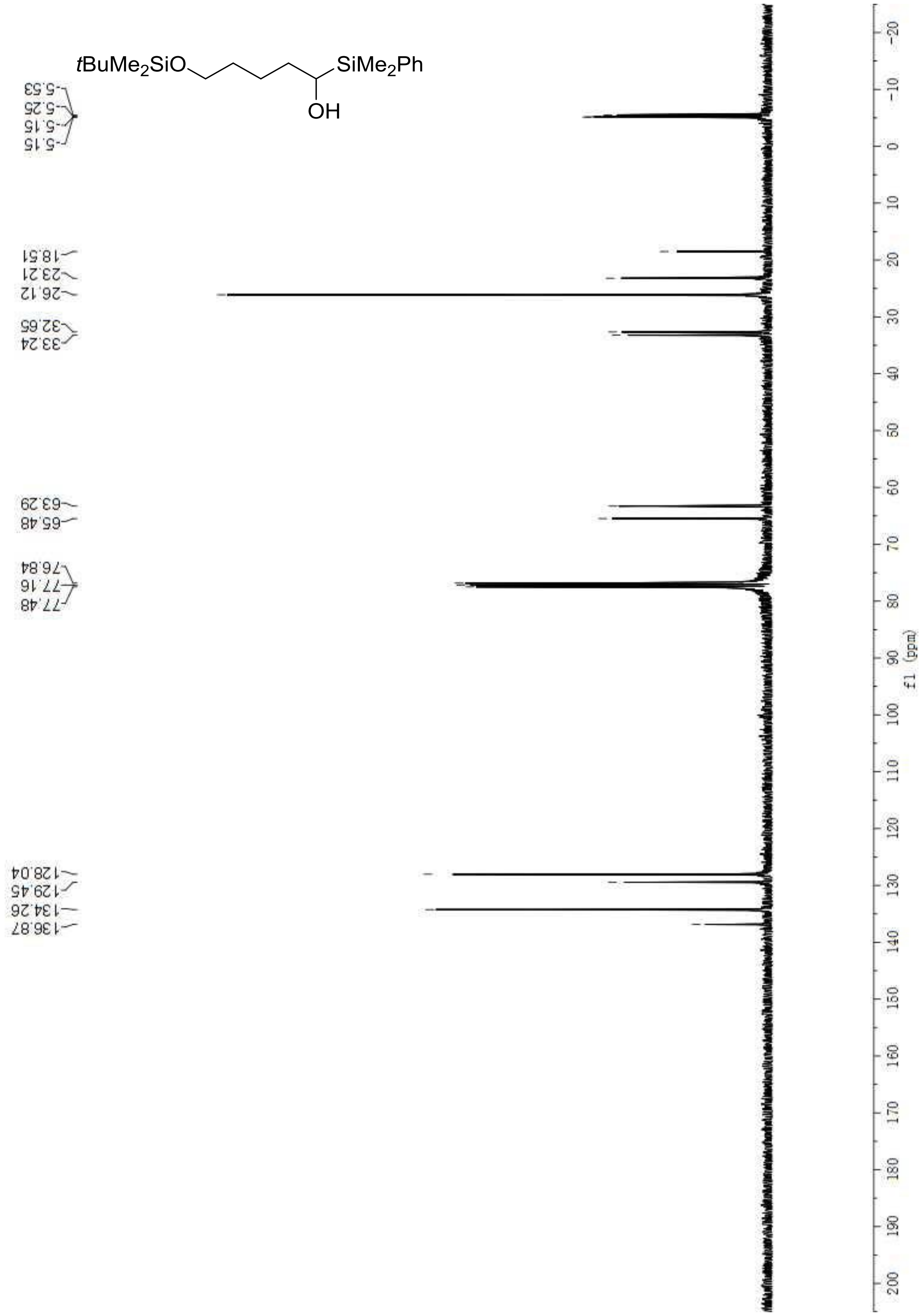

1-(Dimethyl(phenyl)silyl)pentane-1,5-diol (1a-6)

^{1}H NMR of **1a-6** ($CDCl_3$, 600 MHz, 25 ºC)

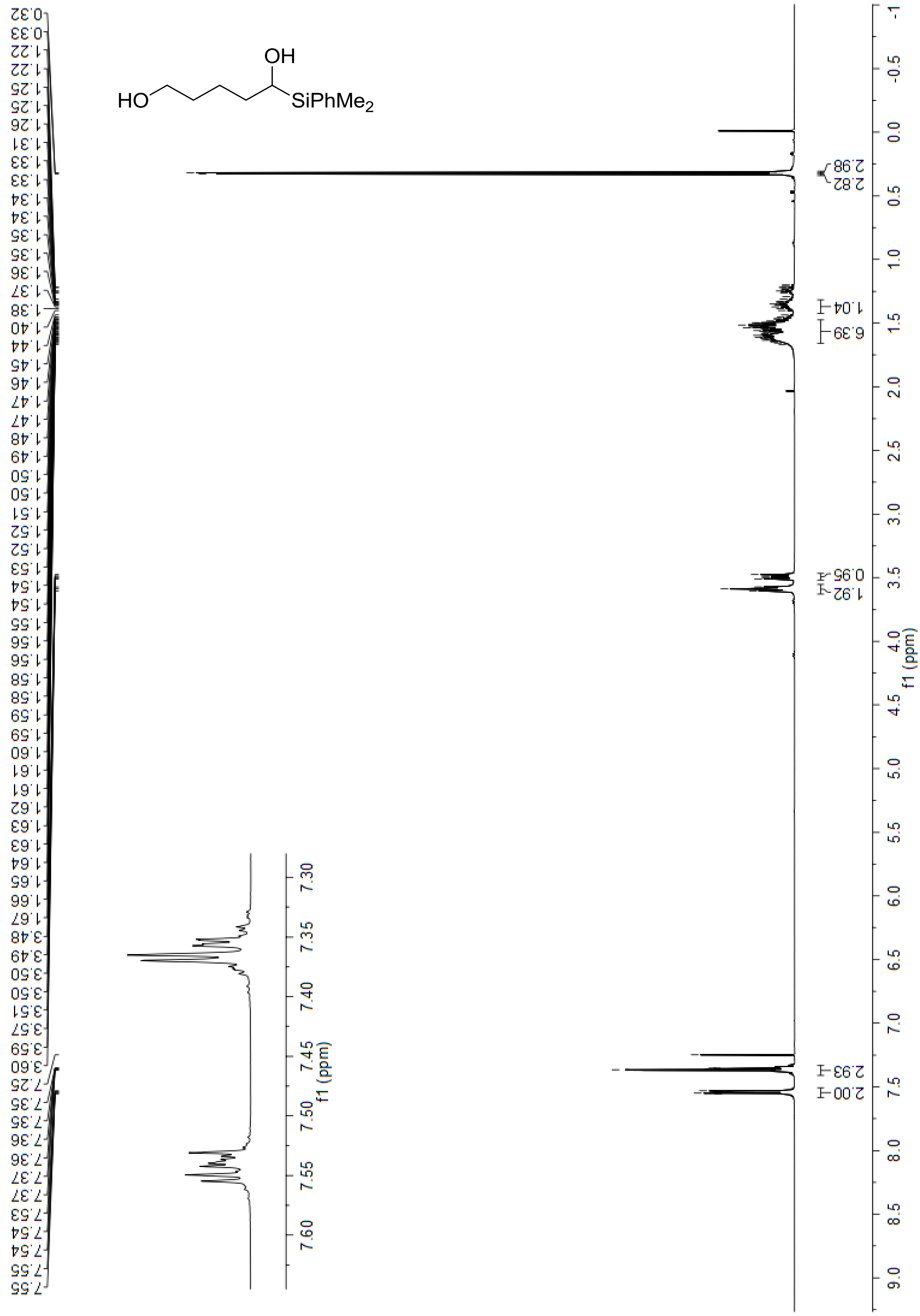

^{13}C NMR of **1a-6** ($CDCl_3$, 151 MHz, 25 ºC)

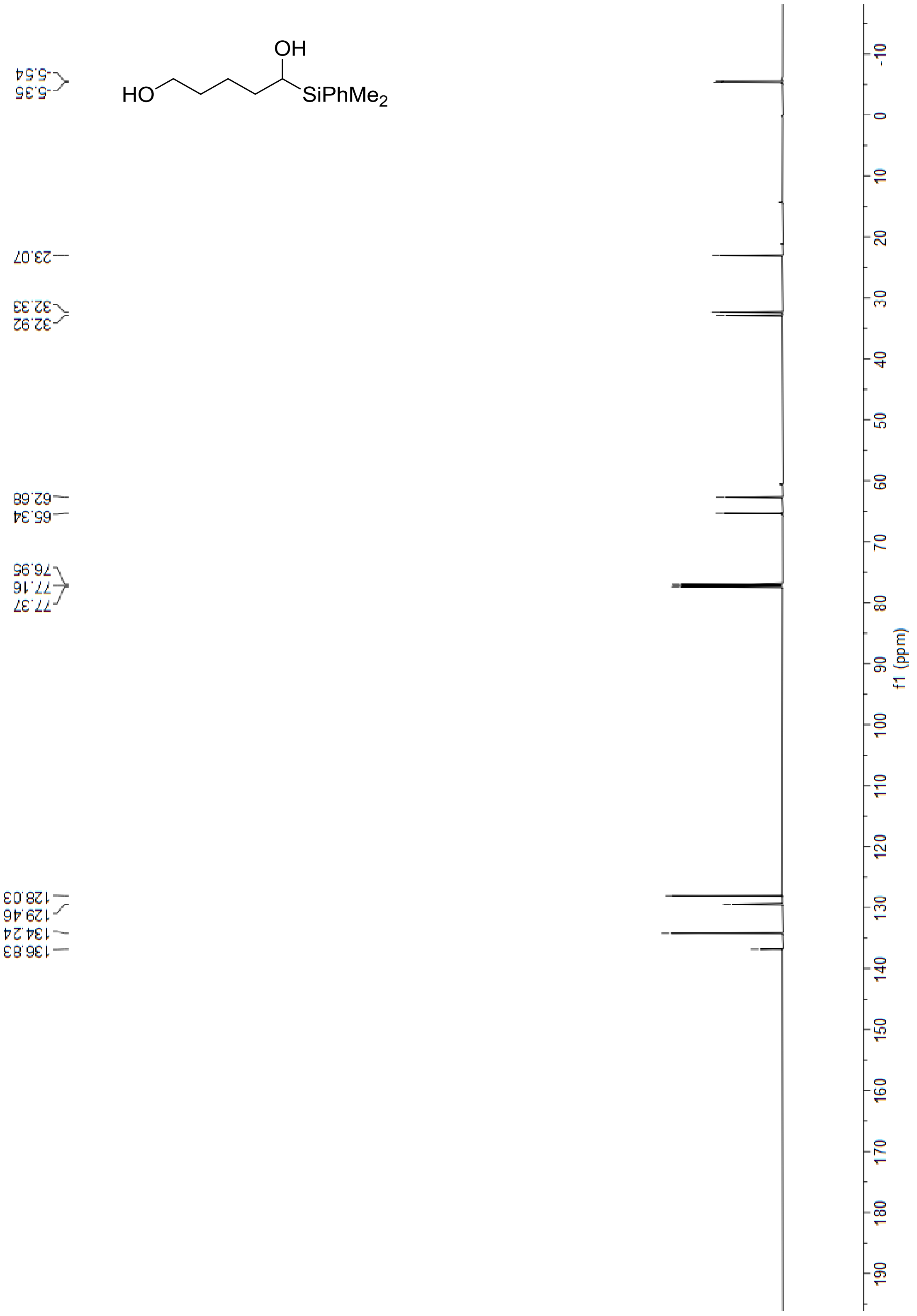
OH
HO
SiPhMe$_2$
-5.35
-5.54
23.07
32.33
32.92
62.68
65.34
76.95
77.16
77.37
128.03
129.46
134.24
136.83
f1 (ppm)
